# Supplementary material for: Integrating I(I)/I(III) catalysis in reaction cascade design enables the synthesis of gem-difluorinated tetralins from cyclobutanols
Source: Nat Commun. 2023 Jun 2;14:3207. doi: 10.1038/s41467-023-38957-w (PMC10238417; doi:10.1038/s41467-023-38957-w)
Supplement: Supplementary file 1 — Supplementary Information [file 41467_2023_38957_MOESM1_ESM.pdf]

Supplementary Information

# Integrating I(I)/I(III) Catalysis in Reaction Cascade Design Enables the Synthesis of *gem*-Difluorinated Tetralins from Cyclobutanols

Joel Häfliger, Louise Ruyet, Nico Stübke, Constantin G. Daniliuc and Ryan Gilmour*

1. **Supplementary Methods** .......... **3**

General Information .......... 3

Synthesis of Cyclobutanones .......... 4

Synthesis of 1,3-Diarylcyclobutan-1-ols .......... 12

Synthesis of (1,3,3-Trifluorobutane-1,4-diyl)dibenzene Derivatives .......... 33

Synthesis of 3,3-Difluoro-1-phenyl-1,2,3,4-tetrahydronaphthalene Derivatives .......... 59

Stepwise Synthesis of **3c** from ***major*-1c** .......... 83

Synthesis of **3c** via Deoxofluorination of Tetralone **S17** .......... 85

Additional Modifications of the Products **2c**, **3q** and **3s** .......... 88

X-Ray Crystallographic Data .......... 101

NMR Spectra .......... 110

2. **Supplementary References** .......... **272**

# 1. Supplementary Methods

## General Information

All reactions were performed by using reagent grade solvents and flame-dried glassware under an argon atmosphere if not stated otherwise. If necessary, solvents were dried by a Grubbs purification system including columns packed with molecular sieves and aluminium oxide. For extractions and purifications, distilled solvents (purchased as technical grade, distilled using rotary evaporator) were used (*n*-pentane, cyclohexane, ethyl acetate, dichloromethane). Diethyl ether and methanol were purchased as reagent grade and were used without further purification. Commercially available chemicals were purchased as reagent grade (suppliers: *abcr*, *Acros*, *Alfa Aesar*, *BLD Pharm*, *Fisher Scientific*, *Fluorochem*, *Sigma Aldrich*, *TCI Europe*) and were used without purification. Purifications by flash column chromatography were performed using $SiO_2$-gel purchased from VWR Chemicals (40-63 µm) and analytical thin layer chromatography was performed using aluminium sheets coated with silica gel 60 $F_{254}$ (Merck). NMR spectra were measured on a *Bruker* AV400, *Agilent* DD2 500 or an *Agilent* DD2 600. The measurements were performed by the NMR service department of the Organisch-Chemisches Institut, Westfälische Wilhelms-Universität Münster. NMR data is reported as following: The chemical shifts (δ) are referenced to the residual solvent signal ($CDCl_3$: $^1$H NMR: 7.26 ppm, $^{13}$C{$^1$H} NMR: 77.16 ppm; DMSO-$d_6$: $^1$H NMR: 2.50 ppm, $^{13}$C{$^1$H} NMR: 39.52 ppm) and are reported in ppm (parts per million) and the identified coupling constants (*J*) are reported in Hertz (Hz). Multiplicities are reported using the following abbreviations: s: singlet, d: doublet, t: triplet, q: quartet, p: quintet, h: sextet, m: multiplet. The assignments of atom numbers were made after considering 2D NMR data (COSY, HMBC and HSQC). HRMS measurement was performed by the mass spectrometry department at the Organisch-Chemisches Institut, Westfälische Wilhelms-Universität Münster. GC-EI analysis was performed using a *Thermo Fisher Scientific* extractive GC-MS. ESI measurements were performed using a *Thermo Fisher Scientific* Orbitrap Velos Pro or a *Thermo Fisher Scientific* Exploris 120 Electrospray Orbitrap. FT-IR spectra were acquired by a Shimadzu IRSpirit ATR FT-IR spectrometer. Absorption maxima are reported in [$cm^{-1}$] and were reported as following: w (weak), m (medium), s (strong), and br (broad). Melting points were determined on a Büchi B-545 melting point apparatus and are reported as a range of 2 ˚C.

## Synthesis of Cyclobutanones

### General Procedure A ([2+2]-Cycloaddition)

The reaction was performed according to a literature procedure.[1]

Dimethylacetamide (1.2 eq.) was dissolved in DCE (0.5 M). $Tf_2O$ (2.0 eq.) was added drop wise and the temperature was kept constant at room temperature by using a water bath. After 15 minutes of stirring, a solution of styrene (1.0 eq.) in a mixture of 2,6-lutidine (2.0 eq.) and DCE (0.2x the amount of DCE used in the first step) was added to the obtained slurry. After complete addition, the reaction mixture was heated to 90 ˚C for the indicated time. The reaction mixture was cooled to room temperature and water (same volume as total volume of DCE) was added. The reaction mixture was heated to 90 ˚C overnight. After cooling to room temperature, the layers were separated and the aqueous layer was extracted with DCM (3x). The combined organic layers were dried over $Na_2SO_4$ and the solvent was removed under reduced pressure. The crude product was purified by flash column chromatography.

### General Procedure B (Wittig)

The reaction was performed according to a literature procedure.[2]

$MePPh_3Br$ (1.5 eq.) was suspended in dry THF (0.5 M). The mixture was cooled to 0 ˚C and *t*-BuOK (1.4 eq.) was added portion wise. The reaction mixture was kept at 0 ˚C for 5 minutes before the cooling was removed. After stirring for 1 h at room temperature, the mixture was cooled to 0 ˚C and the carbonyl compound (1.0 eq.) was added portion wise. After complete addition, the mixture was warmed to room temperature and stirred for the indicated time. The reaction was quenched by adding water unless otherwise stated and the mixture was extracted with the indicated solvent (3x). The combined organic layers were dried over $Na_2SO_4$ and the solvent was removed under reduced pressure. The crude product was purified by flash column chromatography.

**3-Phenylcyclobutan-1-one (S1)**

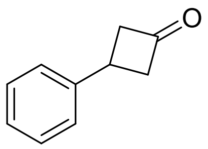

Compound **S1** was prepared following general procedure **A** using styrene (521 mg, 5.0 mmol). The reaction was stirred for 8 h at 90 ˚C. The crude product was purified by flash column chromatography (5-10% $Et_2O$ in *n*-pentane) to yield the product as a brown oil (613 mg, 4.2 mmol, 84%).

$\mathbf{R_f}$ = 0.37 (10% $Et_2O$ in *n*-pentane).

**$^1$H NMR** (400 MHz, $CDCl_3$): $\delta$ [ppm] = 7.40 – 7.33 (m, 2H), 7.33 – 7.23 (m, 3H), 3.69 (p, *J* = 8.6 Hz, 1H), 3.56 – 3.44 (m, 2H), 3.32 – 3.21 (m, 2H).

**GC-EI-MS:** Retention 6.26 min, (*m/z*) requires: $[(C_{10}H_{10}O)^+]$ = 146.07, (*m/z*) found: $[(C_{10}H_{10}O)^+]$ = 146.11.

The analytic data are in good agreement with literature values.[1]

**3-(4-Fluorophenyl)cyclobutan-1-one (S2)**

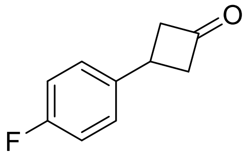

Compound **S2** was prepared following general procedure **A** using 1-fluoro-4-vinylbenzene (878 mg, 7.2 mmol). The reaction was stirred for 8 h at 90 ˚C. The crude product was purified by flash column chromatography (5-10% $Et_2O$ in *n*-pentane) to yield the product as a yellow oil (801 mg, 4.8 mmol, 68%).

$\mathbf{R_f}$ = 0.28 (10% $Et_2O$ in *n*-pentane).

**$^1$H NMR** (400 MHz, $CDCl_3$): $\delta$ [ppm] = 7.30 – 7.22 (m, 2H), 7.08 – 7.01 (m, 2H), 3.74 – 3.60 (m, 1H), 3.56 – 3.43 (m, 2H), 3.27 – 3.15 (m, 2H).

**$^{19}$F NMR** (376 MHz, $CDCl_3$): $\delta$ [ppm] = -116.23 (tt, *J* = 8.6 Hz, *J* = 5.2 Hz, 1F).

**$^{19}$F{$^1$H} NMR** (376 MHz, $CDCl_3$): $\delta$ [ppm] = -116.22 (s, 1F).

**GC-EI-MS:** Retention 6.32 min, (*m/z*) requires: $[(C_{10}H_9FO)^+]$ = 164.06, (*m/z*) found: $[(C_{10}H_9FO)^+]$ = 164.11.

The analytic data are in good agreement with literature values.[1]

**3-(4-Chlorophenyl)cyclobutan-1-one (S3)**

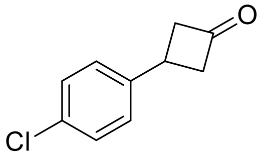

Compound **S3** was prepared following general procedure **A** using 1-chloro-4-vinylbenzene (1.39 g, 10.0 mmol). The reaction was stirred for 9 h at 90 ˚C. The crude product was purified by flash column chromatography (5-10% $Et_2O$ in *n*-pentane) to yield the product as a yellow solid (1.20 g, 6.6 mmol, 66%).

**R$_f$** = 0.23 (10% $Et_2O$ in *n*-pentane).

**$^1$H NMR** (400 MHz, $CDCl_3$): $\delta$ [ppm] = 7.35 – 7.31 (m, 2H), 7.25 – 7.21 (m, 2H), 3.66 (p, *J* = 8.9 Hz, 1H), 3.56 – 3.43 (m, 2H), 3.27 – 3.16 (m, 2H).

**GC-EI-MS:** Retention 7.20 min, (*m/z*) requires: $[(C_{10}H_9ClO)^+]$ = 180.03, (*m/z*) found: $[(C_{10}H_9ClO)^+]$ = 180.06.

The analytic data are in good agreement with literature values.[1]

**1-Bromo-4-vinylbenzene (S4)**

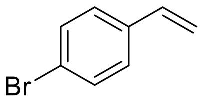


Compound **S4** was prepared following general procedure **B** using 4-bromobenzaldehyde (3.70 g, 20.0 mmol). The reaction was stirred for 16 h at room temperature. The reaction mixture was extracted with *n*-pentane and the crude product was purified by flash column chromatography (100% *n*-pentane) to yield the product as a colorless oil (3.59 g, 19.6 mmol, 98%).

**R$_f$** = 0.77 (100% *n*-pentane).

**$^1$H NMR** (400 MHz, $CDCl_3$): $\delta$ [ppm] = 7.37 – 7.32 (m, 2H), 7.20 – 7.14 (m, 2H), 6.55 (dd, *J* = 17.6 Hz, *J* = 10.9 Hz, 1H), 5.64 (d, *J* = 17.6 Hz, 1H), 5.17 (d, *J* = 10.9 Hz, 1H).

**GC-EI-MS:** Retention 5.53 min, (*m/z*) requires: $[(C_8H_7Br)^+]$ = 181.97, (*m/z*) found: $[(C_8H_7Br)^+]$ = 181.99.

The analytic data are in good agreement with literature values.[3]

**3-(4-Bromophenyl)cyclobutan-1-one (S5)**

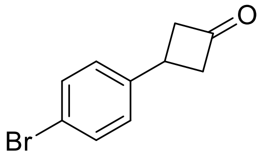


Compound **S5** was prepared following general procedure **A** using 1-bromo-4-vinylbenzene **S4** (2.75 g, 15.0 mmol). The reaction was stirred for 9 h at 90 ˚C. The crude product was purified by flash column chromatography (6-12% $Et_2O$ in *n*-pentane) to yield the product as a yellow solid (1.47 g, 6.5 mmol, 43%).

**R$_f$** = 0.20 (10% $Et_2O$ in *n*-pentane).

**$^1$H NMR** (400 MHz, $CDCl_3$): $\delta$ [ppm] = 7.52 – 7.43 (m, 2H), 7.21 – 7.14 (m, 2H), 3.64 (p, *J* = 8.7 Hz, 1H), 3.57 – 3.44 (m, 2H), 3.27 – 3.15 (m, 2H).

**ESI-MS:** (*m/z*) requires: $[(C_{10}H_9BrONa)^+]$ = 246.97, (*m/z*) found: $[(C_{10}H_9BrONa)^+]$ = 246.97.

The analytic data are in good agreement with literature values.[1]

**1-(Trifluoromethyl)-4-vinylbenzene (S6)**

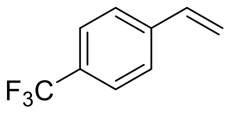


Compound **S6** was prepared following general procedure **B** using 4-(trifluoromethyl)benzaldehyde (1.74 g, 10.0 mmol). The reaction was stirred for 16 h at room temperature. The reaction mixture was extracted with *n*-pentane and the crude product was purified by flash column chromatography (100% *n*-pentane) to yield the product as a colorless oil (789 mg, 4.6 mmol, 46%).

$\mathbf{R_f}$ = 0.82 (100% *n*-pentane).

**$^1$H NMR** (400 MHz, $CDCl_3$): $\delta$ [ppm] = 7.58 (d, *J* = 8.2 Hz, 2H), 7.50 (d, *J* = 8.2 Hz, 2H), 6.75 (dd, *J* = 17.6 Hz, *J* = 10.9 Hz, 1H), 5.85 (d, *J* = 17.5 Hz, 1H), 5.39 (d, *J* = 10.9 Hz, 1H).

**$^{19}$F NMR** (376 MHz, $CDCl_3$): $\delta$ [ppm] = -62.55 (s, 3F).

**$^{19}$F{$^1$H} NMR** (376 MHz, $CDCl_3$): $\delta$ [ppm] = -62.55 (s, 3F).

**GC-EI-MS:** Retention 3.98 min, (*m/z*) requires: [$(C_9H_7F_3)^+$] = 172.05, (*m/z*) found: [$(C_9H_7F_3)^+$] = 172.07.

The analytic data are in good agreement with literature values.[4]

**3-(4-(Trifluoromethyl)phenyl)cyclobutan-1-one (S7)**

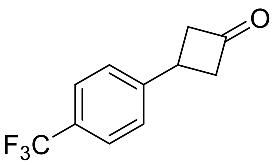


Compound **S7** was prepared following general procedure **A** using 1-(trifluoromethyl)-4-vinylbenzene **S6** (789 mg, 4.6 mmol). The reaction was stirred for 8 h at 90 ˚C. The crude product was purified by flash column chromatography (5-10% $Et_2O$ in *n*-pentane) to yield the product as a yellow oil (393 mg, 1.8 mmol, 39%).

$\mathbf{R_f}$ = 0.20 (10% $Et_2O$ in *n*-pentane).

**$^1$H NMR** (400 MHz, $CDCl_3$): $\delta$ [ppm] = 7.62 (d, *J* = 8.1 Hz, 2H), 7.42 (d, *J* = 8.3 Hz, 2H), 3.75 (p, *J* = 8.0 Hz, 1H), 3.61 – 3.51 (m, 2H), 3.32 – 3.21 (m, 2H).

**$^{19}$F NMR** (376 MHz, $CDCl_3$): $\delta$ [ppm] = -62.49 (s, 3F).

**$^{19}$F{$^1$H} NMR** (376 MHz, $CDCl_3$): $\delta$ [ppm] = -62.49 (s, 3F).

**GC-EI-MS:** Retention 6.40 min, (*m/z*) requires: [$(C_{11}H_9F_3O)^+$] = 214.06, (*m/z*) found: [$(C_{11}H_9F_3O)^+$] = 214.09.

The analytic data are in good agreement with literature values.[5]

**4-Bromo-4'-(trifluoromethyl)-1,1'-biphenyl (S8)**

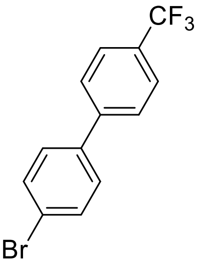


Compound **S8** was prepared following a literature procedure.[6]

A pressure tube was charged with $Pd(PPh_3)_4$ (132 mg, 0.11 mmol, 2 mol%), 4-trifluoromethylphenylboronic acid (1.00 g, 5.2 mmol, 1.0 eq.) and $Na_2CO_3$ (111 mg, 10.4 mmol, 2.0 eq.). The reaction tube was evacuated and backfilled with argon three times. Toluene (12.6 mL) and water (10.5 mL) were added, followed by 1,4-dibromobenzene (4.9 g, 20.8 mmol, 4.0 eq.). The tube was sealed and the reaction mixture was stirred at 110 °C for 16 h. The crude mixture was cooled to room temperature and the aqueous layer was extracted with EtOAc (3x). The organic layer was dried over $Na_2SO_4$ and the volatiles were removed under reduced pressure. The crude product was purified by flash column chromatography (100% *n*-pentane) to yield the product as a colorless solid (552 mg, 1.8 mmol, 35%).

**R*f*** = 0.78 (100% *n*-pentane)

**$^1$H NMR** (500 MHz, $CDCl_3$): $\delta$ [ppm] = 7.73 – 7.63 (m, 4H), 7.61 (d, *J* = 8.4 Hz, 2H), 7.47 (d, *J* = 8.4 Hz, 2H).

**$^{19}$F NMR** (376 MHz, $CDCl_3$): $\delta$ [ppm] = -62.47 (s, 3F).

**$^{19}$F{$^1$H} NMR** (376 MHz, $CDCl_3$): $\delta$ [ppm] = -62.46 (s, 3F).

**GC-EI-MS:** Retention 7.81 min, (*m/z*) requires: $[(C_{13}H_8BrF_3)^+]$ = 301.97, (*m/z*) found: $[(C_{13}H_8BrF_3)^+]$ = 302.01.

The analytic data are in good agreement with literature values.[6]

**4-Formylphenyl trifluoromethanesulfonate (S9)**

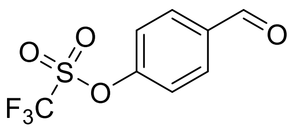


Compound **S9** was prepared following a literature procedure.[4]

4-Hydroxybenzaldehyde (2.44 g, 20.0 mmol, 1.0 eq.) was dissolved in pyridine (20 mL) and the mixture was cooled to 0 ˚C. $Tf_2O$ (6.21 g, 22.0 mmol, 1.1 eq.) was added drop wise. After 10 minutes at 0 ˚C, the reaction mixture was warmed to room temperature. After 16 h, saturated aqueous $NH_4Cl$ (50 mL) and DCM (20 mL) were added and the layers were separated. The organic layer was washed with saturated aqueous $NH_4Cl$ (2x). The organic layer was dried over $Na_2SO_4$ and the solvent was removed under reduced pressure. The crude product was purified by flash column chromatography (10% EtOAc in cyclohexane) to yield the product as a colorless oil, which partially precipitated (4.32 g, 17.0 mmol, 85%).

**R*f*** = 0.35 (20% EtOAc in cyclohexane).

**$^1$H NMR** (400 MHz, $CDCl_3$): $\delta$ [ppm] = 10.05 (s, 1H), 8.04 – 7.97 (m, 2H), 7.50 – 7.44 (m, 2H).

**$^{19}$F NMR** (376 MHz, $CDCl_3$): $\delta$ [ppm] = -72.70 (s, 3F).

**$^{19}$F{$^1$H} NMR** (376 MHz, $CDCl_3$): $\delta$ [ppm] = -72.71 (s, 3F).

**ESI-MS:** (*m/z*) requires: $[(C_8H_5F_3O_4SNa)^+]$ = 276.98, (*m/z*) found: $[(C_8H_5F_3O_4SNa)^+]$ = 277.01.

The analytic data are in good agreement with literature values.[4]

**4-Vinylphenyl trifluoromethanesulfonate (S10)**

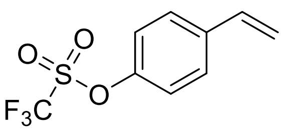


Compound **S10** was prepared following modified general procedure **B** using 4-formylphenyl trifluoromethanesulfonate **S9** (4.32 g, 17.0 mmol). The reaction was stirred for 16 h at room temperature. The reaction mixture was quenched by adding saturated aqueous $NH_4Cl$ and was extracted with EtOAc. The crude product was purified by flash column chromatography (5% EtOAc in cyclohexane) to yield the product as a yellow oil (3.27 g, 13.0 mmol, 76%).

**R*f*** = 0.47 (5% EtOAc in cyclohexane).

**$^1$H NMR** (400 MHz, $CDCl_3$): $\delta$ [ppm] = 7.49 – 7.44 (m, 2H), 7.26 – 7.21 (m, 2H), 6.71 (dd, *J* = 17.5 Hz, *J* = 10.9 Hz, 1H), 5.77 (dd, *J* = 17.6 Hz, *J* = 0.6 Hz, 1H), 5.35 (dd, *J* = 10.9 Hz, *J* = 0.6 Hz, 1H).

**$^{19}$F NMR** (376 MHz, $CDCl_3$): $\delta$ [ppm] = -72.81 (s, 3F).

**$^{19}$F{$^1$H} NMR** (376 MHz, $CDCl_3$): $\delta$ [ppm] = -72.80 (s, 3F).

**GC-EI-MS:** Retention 6.36 min, (*m/z*) requires: $[(C_9H_7F_3O_3S)^+]$ = 252.01, (*m/z*) found $[(C_9H_7F_3O_3S)^+]$ = 252.01.

The analytic data are in good agreement with literature values.[C]

**4-(3-Oxocyclobutyl)phenyl trifluoromethanesulfonate (S11)**

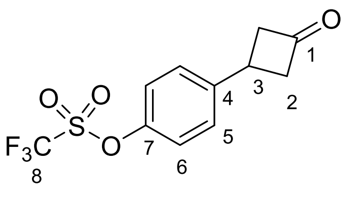


Compound **S11** was prepared following general procedure **A** using 4-vinylphenyl trifluoromethanesulfonate **S10** (3.27 g, 13.0 mmol). The reaction was stirred for 8 h at 90 ˚C. The crude product was purified by flash column chromatography (10-15% EtOAc in cyclohexane) to yield the product as a yellow oil (1.89 g, 6.4 mmol, 49%).

**R*f*** = 0.25 (20% EtOAc in cyclohexane).

**$^{1}$H NMR** (500 MHz, $CDCl_3$): $\delta$ [ppm] = 7.40 – 7.36 (m, 2H, H-C5), 7.28 – 7.24 (m, 2H, H-C6), 3.72 (tt, $^{3}J_{HH}$ = 9.4 Hz, $^{3}J_{HH}$ = 7.4 Hz, 1H, H-C3), 3.58 – 3.50 (m, 2H, $H^a$-C2), 3.28 – 3.20 (m, 2H, $H^b$-C2).

**$^{19}$F NMR** (376 MHz, $CDCl_3$): $\delta$ [ppm] = -72.81 (s, 3F, F-C8).

**$^{19}$F{$^{1}$H} NMR** (376 MHz, $CDCl_3$): $\delta$ [ppm] = -72.81 (s, 3F, F-C8).

**$^{13}$C{$^{1}$H} NMR** (126 MHz, $CDCl_3$): $\delta$ [ppm] = 205.4 (C1), 148.3 (C7), 144.3 (C4), 128.6 (C5), 121.7 (C6), 118.6 (q, $^{1}J_{CF}$ = 320.3 Hz, C8), 54.9 (C2), 28.2 (C3).

**ESI-MS:** (*m/z*) requires: [$(C_{11}H_9F_3O_4SNa)^+$] = 317.0066, (*m/z*) found: [$(C_{11}H_9F_3O_4SNa)^+$] = 317.0066.

**FT-IR** ($\tilde{\nu}$ = $cm^{-1}$)**:** 1785.0 (m), 1502.4 (w), 1419.1 (m), 1249.8 (w),1203.9 (s), 1133.6 (s), 1102.0 (m), 1015.9 (w), 988.7 (w), 881.0 (s), 833.7 (m), 780.6 (w), 756.2 (w), 730.4 (w), 703.1 (w).

**1-Chloro-4-(prop-1-en-2-yl)benzene (S12)**

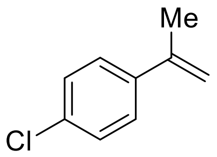


Compound **S12** was prepared following general procedure **B** using 1-(4-chlorophenyl)ethan-1-one (3.10 g, 20.0 mmol). The reaction was stirred for 16 h at room temperature. The reaction mixture was quenched by adding water and was extracted with $Et_2O$. The crude product was purified by flash column chromatography (100% *n*-pentane) to yield the product as a colorless oil (2.78 g, 18.2 mmol, 91%).

**$R_f$** = 0.70 (100% *n*-pentane).

**$^{1}$H NMR** (400 MHz, $CDCl_3$): $\delta$ [ppm] = 7.43 – 7.36 (m, 2H), 7.33 – 7.27 (m, 2H), 5.36 (dq, *J* = 1.6 Hz, *J* = 0.8 Hz, 1H), 5.11 (p, *J* = 1.5 Hz, 1H), 2.14 (dd, *J* = 1.6 Hz, *J* = 0.9 Hz, 3H).

**GC-EI-MS:** Retention 5.90 min, (*m/z*) requires: [$(C_9H_9Cl)^+$] = 152.04, (*m/z*) found [$(C_9H_9Cl)^+$] = 152.05.

The analytic data are in good agreement with literature values.[7]

**3-(4-Chlorophenyl)-3-methylcyclobutan-1-one (S13)**

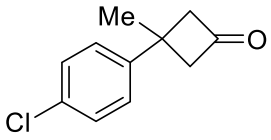


Compound **S13** was prepared following general procedure **A** using 1-chloro-4-(prop-1-en-2-yl)benzene **S12** (2.29 g, 15.0 mmol). The reaction was stirred for 9 h at 90 ˚C. The crude product was purified by flash column chromatography (5-10% $Et_2O$ in *n*-pentane) to yield the product as a brown solid (2.46 g, 12.6 mmol, 84%).

**$R_f$** = 0.41 (10% $Et_2O$ in *n*-pentane).

**$^1$H NMR** (400 MHz, $CDCl_3$): $\delta$ [ppm] = 7.36 – 7.31 (m, 2H), 7.26 – 7.22 (m, 2H), 3.50 – 3.37 (m, 2H), 3.17 – 3.06 (m, 2H), 1.60 (s, 3H).

**GC-EI-MS:** Retention 8.14 min, (*m/z*) requires: [$(C_{11}H_{11}ClO)^+$] = 194.05, (*m/z*) found: [$(C_{11}H_{11}ClO)^+$] = 194.05.

The analytic data are in good agreement with literature values.[8]

**3-(4-Bromophenyl)pyridine (S14)**

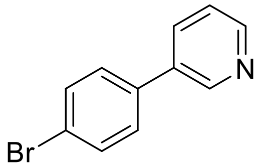


Compound **S14** was prepared following a literature procedure.[9]

A pressure tube was charged with $Pd(PPh_3)_4$ (210 mg, 0.18 mmol, 5 mol%), cesium carbonate (2.30 g, 7.15 mmol, 2.0 eq.), 1,4-dibromobenzene (2.00 g, 8.0 mmol, 2.2 eq.) and 3-pyridylboronic acid (0.44 g, 3.6 mmol, 1.0 eq.). The flask was evacuated under vacuum and backfilled with argon three times. 1,4-Dioxane (48 mL) and water (12 mL) were added. The tube was sealed, and the reaction mixture was stirred at 88 °C for 16 h. The reaction mixture was cooled to room temperature and the organic layer was separated. The aqueous layer was extracted with DCM (3x). The combined organic layers were dried over $Na_2SO_4$ and the volatiles were removed under reduced pressure. The crude product was purified by flash column chromatography (2% EtOH in DCM) to yield the product as a colorless solid (583 mg, 2.5 mmol, 69%).

**$R_f$** = 0.42 (2% EtOH in DCM).

**$^1$H NMR** (400 MHz, $CDCl_3$): $\delta$ [ppm] = 8.81 (d, *J* = 2.4 Hz, 1H), 8.60 (dd, *J* = 4.8 Hz, *J* = 1.6 Hz, 1H), 7.82 (dt, *J* = 7.8 Hz, *J* = 2.1 Hz, 1H), 7.59 (d, *J* = 8.5 Hz, 2H), 7.43 (d, *J* = 8.5 Hz, 2H), 7.35 (dd, *J* = 7.9 Hz, *J* = 4.9 Hz, 1H).

**ESI-MS:** (*m/z*) requires: [$(C_{11}H_8NBrNa)^+$] = 257.97, (*m/z*) found: [$(C_{11}H_8NBrNa)^+$] = 257.97.

The analytic data are in good agreement with literature values.[9]

## Synthesis of 1,3-Diarylcyclobutan-1-ols

### General Procedure C

The reaction was performed according to a literature procedure.[10]

Bromobenzene derivative (1.2 eq.) was dissolved in dry THF (0.5 M) and the reaction mixture was cooled to -78 ˚C. *n*-BuLi (1.6 M in hexanes, 1.2 eq.) was added drop wise and the reaction was stirred for 1 h at -78 ˚C. A solution of cyclobutanone derivative (1.0 eq.) in dry THF (2 M) was added and the temperature was kept at -78 ˚C for another 1 h after complete addition. The reaction was warmed to room temperature and stirred for 2 h. The reaction was quenched by adding saturated aqueous $NH_4Cl$ and the aqueous layer was extracted with EtOAc (3x). The combined organic layers were dried over $Na_2SO_4$ and the solvent was removed under reduced pressure. The crude product was purified by flash column chromatography.

**1,3-Diphenylcyclobutan-1-ol (1a)**

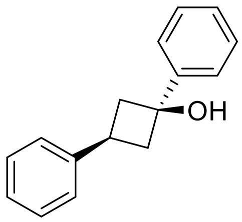


Compound **1a** was prepared following general procedure **C** using bromobenzene (377 mg, 2.40 mmol) and 3-phenylcyclobutan-1-one **S1** (292 mg, 2.00 mmol). The crude product was purified by flash column chromatography (10-15% EtOAc in cyclohexane) to yield product **1a** as a white solid (mixture of diastereoisomers, d.r. = 89:11, 379 mg, 1.69 mmol, 85%).

**$R_f$** = 0.36 (15% EtOAc in cyclohexane).

**$^1$H NMR** (400 MHz, $CDCl_3$): $\delta$ [ppm] = 7.67 – 7.63 (m, 2H), 7.47 – 7.41 (m, 2H), 7.38 – 7.28 (m, 5H), 7.25 – 7.19 (m, 1H), 3.15 – 3.02 (m, 3H), 2.62 – 2.54 (m, 2H), 2.10 (s, 1H).

**ESI-MS:** (*m/z*) requires: [$(C_{16}H_{16}ONa)^+$] = 247.11, (*m/z*) found: [$(C_{16}H_{16}ONa)^+$] = 247.11.

The analytic data are in good agreement with literature values.[11]

**1,3-Bis(4-fluorophenyl)cyclobutan-1-ol (1b)**

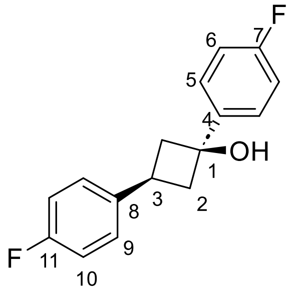


Compound **1b** was prepared following general procedure **C** using 1-bromo-4-fluorobenzene (245 mg, 1.40 mmol) and 3-(4-fluorophenyl)cyclobutan-1-one **S2** (200 mg, 1.22 mmol). The crude product was purified by flash column chromatography (10-20% EtOAc in *n*-pentane) to yield product **1b** as a colorless solid (mixture of diastereoisomers, d.r. = 96:04, 226 mg, 0.90 mmol, 71%).

**$R_f$** = 0.28 (10% EtOAc in *n*-pentane).

**Melting Point:** 76-78°C.

**$^1$H NMR** (599 MHz, $CDCl_3$): $\delta$[ppm] = 7.62 – 7.56 (m, 2H, H-C5), 7.25 – 7.20 (m, 2H, H-C9), 7.14 – 7.08 (m, 2H, H-C6), 7.03 – 6.97 (m, 2H, H-C10), 3.06 – 2.99 (m, 3H, $H^a$-C2, H-C3), 2.56 – 2.47 (m, 2H, $H^b$-C2), 2.09 (s, 1H, H-O).

**$^{19}$F NMR** (376 MHz, $CDCl_3$): $\delta$ [ppm] = -114.83 (tt, $^3J_{FH}$ = 8.6 Hz, $^4J_{FH}$ = 5.3 Hz, 1F, F-C7 or F-C11), -117.12 (tt, $^3J_{FH}$ = 8.7 Hz, $^3J_{FH}$ = 5.4 Hz, 1F, F-C7 or F-C11).

**$^{19}$F{$^1$H} NMR** (376 MHz, $CDCl_3$): $\delta$ [ppm] = -114.80 (s, 1F, F-C7 or F-C11), -117.06 (s, 1F, F-C7 or F-C11).

**$^{13}$C{$^1$H} NMR** (151 MHz, $CDCl_3$): $\delta$ [ppm] = 162.8 (d, $^1J_{CF}$ = 126.6 Hz, C7), 161.2 (d, $^1J_{CF}$ = 124.3 Hz, C11), 141.2 (d, $^4J_{CF}$ = 3.2 Hz, C4), 140.2 (d, $^4J_{CF}$ = 3.1 Hz, C8), 128.2 (d, $^3J_{CF}$ = 7.8 Hz, C5), 127.5 (d,

$^3J_{CF}$ = 8.1 Hz, C9), 115.6 (d, $^2J_{CF}$ = 21.4 Hz, C6), 115.3 (d, $^2J_{CF}$ = 21.1 Hz, C10), 72.2 (C1), 45.0 (C2), 29.5 (C3).

**GC-EI-MS:** Retention 9.93 min**,** (*m/z*) requires: [($C_{16}H_{14}F_2O$)$^+$] = 260.1009, (*m/z*) found: [($C_{16}H_{14}F_2O$)$^+$] = 260.1007.

**FT-IR** ($\tilde{\nu}$ = cm$^{-1}$)**:** 3261.6 (broad), 2976.0 (w), 2360.4 (w), 1601.4 (m), 1508.1 (s), 1457.9 (w), 1427.7 (w), 1277.1 (m), 1245.5 (m), 1226.9 (s), 1156.5 (m), 1081.9 (s), 1070.5 (s), 1033.1 (m), 1014.5 (m), 952.8 (m), 898.3 (w), 838.0 (s), 812.2 (s), 774.9 (s), 668.7 (m), 657.2 (s).

**1,3-Bis(4-chlorophenyl)cyclobutan-1-ol (1c)**

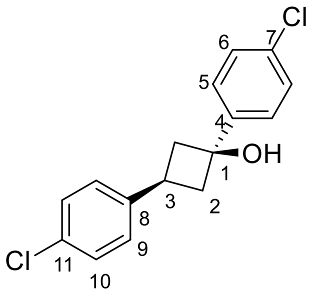


Compound **1c** was prepared following general procedure **C** using 1-bromo-4-chlorobenzene (1.84 mg, 9.60 mmol) and 3-(4-chlorophenyl)cyclobutan-1-one **S3** (1.45 g, 8.00 mmol). The crude product was purified by flash column chromatography (10-15% EtOAc in cyclohexane) to yield product **1c** as a pale-yellow solid (single diastereoisomer, 1.74 g, 5.93 mmol, 74%) and product ***minor*-1c** as a pale-yellow solid (enriched mixture of diastereoisomers, *d.r.* = 11:89, 221 mg, 0.75 mmol, 9%).

***major*-1c**

**R*f*** = 0.41 (20% EtOAc in cyclohexane).

**Melting Point:** 88-90 ˚C.

**$^1$H NMR** (500 MHz, $CDCl_3$): $\delta$[ppm] = 7.57 – 7.53 (m, 2H, H-C6), 7.42 – 7.37 (m, 2H, H-C5), 7.31 – 7.27 (m, 2H, H-C9), 7.22 – 7.18 (m, 2H, H-C10), 3.09 – 2.97 (m, 3H, H$^a$-C2, H-C3), 2.55 – 2.48 (m, 2H, H$^b$-C2), 2.16 (s, 1H, H-O).

**$^{13}$C{$^1$H} NMR** (126 MHz, $CDCl_3$): $\delta$[ppm] = 143.8 (C4), 143.0 (C8), 133.7 (C7), 132.1 (C11), 128.9 (C5), 128.7 (C9), 128.1 (C10), 127.1 (C6), 72.2 (C1), 44.8 (C2), 29.6 (C3).

**ESI-MS:** (*m/z*) requires: [($C_{16}H_{14}Cl_2ONa$)$^+$] = 315.0314, (*m/z*) found: [($C_{16}H_{14}Cl_2ONa$)$^+$] = 315.0314.

**FT-IR** ($\tilde{\nu}$ = cm$^{-1}$)**:** 3291.7 (broad), 2973.1 (w), 2934.4 (w), 1783.6 (w), 1574.1 (w), 1489.4 (m), 1456.4 (w), 1429.2 (w), 1397.6 (w), 1304.3 (w), 1278.5 (w), 1234.0 (m), 1159.4 (m), 1119.2 (w), 1083.4 (s), 1066.1 (m), 1026.0 (w), 1011.6 (m), 954.2 (w), 891.1 (w), 838.0 (s), 825.1 (s), 813.6 (s), 803.6 (m), 743.3 (m), 721.8 (w), 713.2 (m).

***minor*-1c**

**$R_f$** = 0.36 (20% EtOAc in cyclohexane).

**Melting Point:** 114-116 ˚C.

**$^1$H NMR** (599 MHz, $CDCl_3$): $\delta$ [ppm] = 7.36 – 7.31 (m, 4H, H-C5, H-C6), 7.29 – 7.26 (m, 2H, H-C10), 7.16 – 7.12 (m, 2H, H-C9), 3.93 (p, $^3J_{HH}$ = 9.0 Hz, 1H, H-C3), 2.78 – 2.72 (m, 2H, $H^a$-C2), 2.60 – 2.53 (m, 2H, $H^b$-C2), 2.20 (s, 1H, H-O).

**$^{13}$C{$^1$H} NMR** (151 MHz, $CDCl_3$): $\delta$ [ppm] = 145.0 (C4), 143.3 (C8), 133.4 (C7), 131.9 (C11), 128.8 (C5 or C6), 128.6 (C10), 127.9 (C9), 126.4 (C5 or C6), 74.7 (C1), 43.3 (C2), 33.0 (C3).

**GC-EI-MS:** Retention 11.43 min, (*m/z*) requires: [$(C_{16}H_{14}Cl_2O)^+$] = 292.0416, (*m/z*) found: [$(C_{16}H_{14}Cl_2O)^+$] = 292.0415.

**FT-IR** ($\tilde{\nu}$ = $cm^{-1}$)**:** 3278.8 (broad), 2980.3 (w), 2934.4 (w), 1485.1 (m), 1410.5 (w), 1394.7 (w), 1297.2 (w), 1279.9 (m), 1228.3 (m),1170.9 (m), 1126.4 (w), 1086.2 (s), 1066.1 (m), 1008.7 (s), 932.7 (m), 899.7 (m), 832.3 (s), 825.1 (s), 806.4 (m), 743.3 (m), 724.6 (m), 710.3 (m).

Comment: The diastereoisomers were assigned based on the crystal structure of ***minor*-1c** (see below).

**1,3-Bis(4-bromophenyl)cyclobutan-1-ol (1d)**

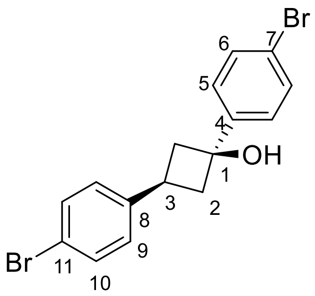


Compound **1d** was prepared following general procedure **C** using 1,4-dibromobenzene (566 mg, 2.40 mmol) and 3-(4-bromophenyl)cyclobutan-1-one **S5** (450 mg, 2.00 mmol). The crude product was purified by flash column chromatography (10-15% EtOAc in cyclohexane) to yield product **1d** as a pale-yellow solid (single diastereoisomer, 538 mg, 1.41 mmol, 71%).

**$R_f$** = 0.39 (20% EtOAc in cyclohexane).

**Melting Point:** 95-97 ˚C.

**$^1$H NMR** (599 MHz, $CDCl_3$): $\delta$ [ppm] = 7.56 – 7.53 (m, 2H, H-C6), 7.50 – 7.47 (m, 2H, H-C5), 7.46 – 7.42 (m, 2H, H-C10), 7.16 – 7.13 (m, 2H, H-C9), 3.07 – 2.97 (m, 3H, $H^a$-C2, H-C3), 2.54 – 2.48 (m, 2H, $H^b$-C2), 2.18 (s, 1H, H-O).

**$^{13}$C{$^1$H} NMR** (151 MHz, $CDCl_3$): $\delta$ [ppm] = 144.3 (C4), 143.5 (C8), 131.9 (C6), 131.6 (C10), 128.6 (C9), 127.4 (C5), 121.8 (C7), 120.1 (C11), 72.2 (C1), 44.7 (C2), 29.6 (C3).

**ESI-MS:** (*m/z*) requires: [($C_{16}H_{14}Br_2ONa)^+$] = 402.9304, (*m/z*) found: [($C_{16}H_{14}Br_2ONa)^+$] = 402.9305.

**FT-IR** ($\tilde{\nu}$ = cm$^{-1}$)**:** 3294.6 (broad), 2967.4 (w), 2933.0 (w), 1486.6 (m), 1429.2 (w), 1394.7 (w), 1232.6 (m), 1159.4 (m), 1119.2 (w), 1103.5 (w), 1073.3 (s), 1027.4 (w), 1007.3 (s), 952.8 (w), 888.2 (w), 836.6 (s), 823.6 (s), 812.2 (s), 800.7 (m), 728.9 (m), 710.3 (m).

**1,3-Bis(4-(trifluoromethyl)phenyl)cyclobutan-1-ol (1e)**

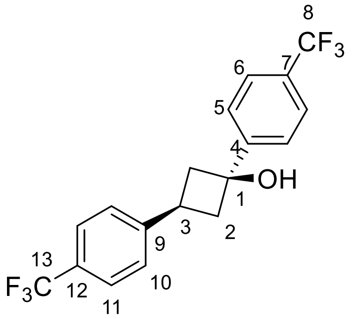


Compound **1e** was prepared following general procedure **C** using 1-bromo-4-(trifluoromethyl)benzene (540 mg, 2.4 mmol) and 3-(4-(trifluoromethyl)phenyl)cyclobutan-1-one **S7** (428 mg, 2.00 mmol). The crude product was purified by flash column chromatography (10-15% EtOAc in cyclohexane) to yield product **1e** as a yellow solid (single diastereoisomer, 516 mg, 1.43 mmol, 72%).

**R*f*** = 0.36 (20% EtOAc in cyclohexane).

**Melting Point:** 90-92 ˚C.

**$^1$H NMR** (500 MHz, $CDCl_3$): $\delta$ [ppm] = 7.75 (d, $^3J_{HH}$ = 8.2 Hz, 2H, H-C5), 7.70 (d, $^3J_{HH}$ = 8.1 Hz, 2H, H-C6), 7.60 (d, $^3J_{HH}$ = 7.9 Hz, 2H, H-C11), 7.40 (d, $^3J_{HH}$ = 8.0 Hz, 2H, H-C10), 3.21 (p, $^3J_{HH}$ = 9.4 Hz, 1H, H-C3), 3.13 – 3.05 (m, 2H, H$^a$-C2), 2.65 – 2.57 (m, 2H, H$^b$-C2), 2.28 (s, 1H, H-O).

**$^{19}$F NMR** (376 MHz, $CDCl_3$): $\delta$ [ppm] = -62.35 (s, 3F, F-C8 or F-C13), -62.54 (s, 3F, F-C8 or F-C13).

**$^{19}$F{$^1$H} NMR** (376 MHz, $CDCl_3$): $\delta$ [ppm] = -62.35 (s, 3F, F-C8 or F-C13), -62.54 (s, 3F, F-C8 or F-C13).

**$^{13}$C{$^1$H} NMR** (126 MHz, $CDCl_3$): $\delta$ [ppm] = 149.2 (q, $^5J_{CF}$ = 1.3 Hz, C4), 148.5 (q, $^5J_{CF}$ = 1.3 Hz, C9), 130.2 (q, $^2J_{CF}$ = 32.5 Hz, C7), 128.8 (q, $^2J_{CF}$ = 32.4 Hz, C12), 127.1 (C10), 125.9 (C5), 125.8 (q, $^3J_{CF}$ = 3.8 Hz, C6), 125.6 (q, $^3J_{CF}$ = 3.8 Hz, C11), 124.4 (q, $^1J_{CF}$ = 271.9 Hz, C13), 124.2 (q, $^1J_{CF}$ = 272.0 Hz, C8), 72.4 (s, C1), 44.7 (C2), 29.9 (C3).

*Comment: HRMS-analysis was inconclusive, therefore [M]-F was reported as an indicative fragment.*

**GC-EI-MS:** Retention 9.90 min, (*m/z*) requires: [($C_{18}H_{14}F_6O$ - F)$^+$] = 341.0959, (*m/z*) found: [($C_{18}H_{14}F_6O$ - F)$^+$] = 341.0959.

**FT-IR** ($\tilde{\nu}$ = cm$^{-1}$)**:** 3337.6 (broad), 1620.0 (w), 1420.6 (w), 1410.5 (w), 1323.0 (s), 1242.6 (w), 1192.4 (w), 1158.0 (m), 1109.2 (s), 1069.0 (s), 1017.4 (m), 954.2 (w), 872.4 (w), 833.7 (s), 767.7 (w), 717.5 (m).

**1-(4-Fluorophenyl)-3-phenylcyclobutan-1-ol (1f)**

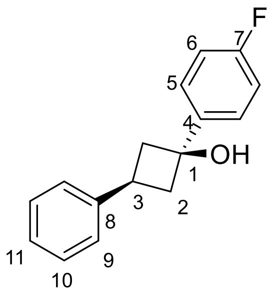


Compound **1f** was prepared following general procedure **C** using 1-bromo-4-fluorobenzene (420 mg, 2.40 mmol) and 3-phenylcyclobutan-1-one **S1** (292 mg, 2.00 mmol). The crude product was purified by flash column chromatography (10-15% EtOAc in cyclohexane) to yield product **1f** as a colorless solid (mixture of diastereoisomers, d.r. = 93:07, 420 mg, 1.73 mmol, 87%).

$\mathbf{R}_f$ = 0.38 (20% EtOAc in cyclohexane).

**Melting Point:** 89-91 ˚C.

**$^1$H NMR** (599 MHz, $CDCl_3$): $\delta$[ppm] = 7.63 – 7.58 (m, 2H, H-C5), 7.36 – 7.32 (m, 2H, H-C10), 7.31 – 7.27 (m, 2H, H-C9), 7.25 – 7.21 (m, 1H, H-C11), 7.14 – 7.09 (m, 2H, H-C6), 3.12 – 3.00 (m, 3H, $H^a$-C2, H-C3), 2.59 – 2.54 (m, 2H, $H^b$-C2), 2.20 (s, 1H, H-O).

**$^{19}$F NMR** (376 MHz, $CDCl_3$): $\delta$[ppm] = -114.95 (tt, $^3J_{FH}$ = 8.5 Hz, $^4J_{FH}$ = 5.3 Hz, 1F, F-C7).

**$^{19}$F{$^1$H} NMR** (376 MHz, $CDCl_3$): $\delta$[ppm] = -114.95 (s, 1F, F-C7).

**$^{13}$C{$^1$H} NMR** (151 MHz, $CDCl_3$): $\delta$ [ppm] = 162.3 (d, $^1J_{CF}$ = 246.2 Hz, C7), 144.5 (C8), 141.3 (d, $^4J_{CF}$ = 3.2 Hz, C4), 128.6 (C10), 127.5 (d, $^3J_{CF}$ = 8.2 Hz, C5), 126.8 (C9), 126.3 (C11), 115.5 (d, $^2J_{CF}$ = 21.3 Hz, C6), 72.3 (C1), 44.9 (C2), 30.0 (C3).

**GC-EI-MS:** Retention 9.89 min, (*m/z*) requires: [$(C_{16}H_{15}FO)^+$] = 242.1101, (*m/z*) found: [$(C_{16}H_{15}FO)^+$] = 242.1103.

**FT-IR** ($\tilde{\nu}$ = $cm^{-1}$)**:** 3265.9 (broad), 1601.4 (w), 1511.0 (m), 1495.2 (w), 1459.3 (w), 1446.4 (w), 1427.7 (w), 1300.0 (w), 1277.1 (w), 1221.1 (m), 1166.6 (w), 1153.7 (m), 1087.7 (m), 1051.8 (w), 1030.3 (w), 1014.5 (w), 955.7 (w), 934.1 (w), 896.8 (w), 838.0 (s), 789.2 (w), 747.6 (s).

**3-Phenyl-1-(4-chlorophenyl)cyclobutan-1-ol (1g)**

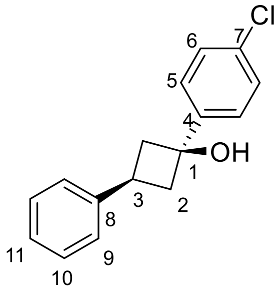


Compound **1g** was prepared following general procedure **C** using 1-bromo-4-chlorobenzene (460 mg, 2.40 mmol) and 3-phenylcyclobutan-1-one **S1** (292 mg, 2.00 mmol). The crude product was purified by flash column chromatography (10-15% EtOAc in cyclohexane) to yield product **1g** as a colorless solid (single diastereoisomer, 405 mg, 1.57 mmol, 79%).

$\mathbf{R}_f$ = 0.33 (20% EtOAc in cyclohexane).

**Melting Point:** 79-81 ˚C.

**$^1$H NMR** (599 MHz, $CDCl_3$): $\delta$ [ppm] = 7.59 – 7.55 (m, 2H, H-C6), 7.42 – 7.38 (m, 2H, H-C5), 7.34 (t, $^3J_{HH}$ = 7.2 Hz, 2H, H-C10), 7.29 (d, $^3J_{HH}$ = 6.9 Hz, 2H, H-C9), 7.23 (tt, $^3J_{HH}$ = 7.3 Hz, $J_{HH}$ = 1.3 Hz, 1H, H-C11), 3.09 (p, $^3J_{HH}$ = 9.0 Hz, 1H, H-C3), 3.04 – 2.98 (m, 2H, $H^a$-C2), 2.59 – 2.54 (m, 2H, $H^b$-C2), 2.20 (s, 1H, H-O).

**$^{13}$C{$^1$H} NMR** (151 MHz, $CDCl_3$): $\delta$[ppm] = 144.5 (C8), 144.1 (C4), 133.6 (C7), 128.9 (C5), 128.6 (C10), 127.1 (C6), 126.8 (C9), 126.4 (C11), 72.3 (C1), 44.9 (C2), 30.0 (C3).

**ESI-MS:** (*m/z*) requires: [($C_{16}H_{15}ClONa)^+$] = 281.0704, (*m/z*) found: [($C_{16}H_{15}ClONa)^+$] = 281.0703.

**FT-IR** ($\tilde{\nu}$ = cm$^{-1}$)**:** 3277.3 (broad), 1599.9 (w), 1492.3 (w), 1446.4 (w), 1231.2 (m), 1156.5 (w), 1094.8 (m), 1053.2 (w), 1030.3 (w), 1011.6 (m), 955.7 (w), 947.0 (w), 896.8 (w), 838.0 (s), 825.1 (m), 802.1 (w), 750.5 (s), 734.7 (w), 720.3 (w).

**3-Phenyl-1-(4-bromophenyl)cyclobutan-1-ol (1h)**

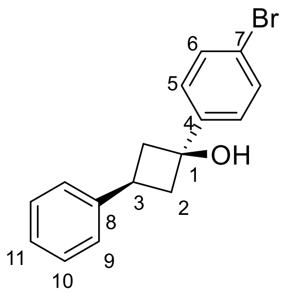


Compound **1h** was prepared following general procedure **C** using 1,4-dibromobenzene (566 mg, 2.40 mmol) and 3-phenylcyclobutan-1-one **S1** (292 mg, 2.00 mmol). The crude product was purified by flash column chromatography (10-15% EtOAc in cyclohexane) to yield product **1h** as a colorless solid (mixture of diastereoisomers, *d.r.* = 97:03, 489 mg, 1.61 mmol, 81%).

**$R_f$** = 0.43 (20% EtOAc in cyclohexane).

**Melting Point:** 82-84 ˚C.

**$^1$H NMR** (599 MHz, $CDCl_3$): $\delta$ [ppm] = 7.57 – 7.54 (m, 2H, H-C5), 7.53 – 7.50 (m, 2H, H-C6), 7.34 (t, $^3J_{HH}$ = 7.6 Hz, 2H, H-C10), 7.29 (d, $^3J_{HH}$ = 7.3 Hz, 2H, H-C9), 7.23 (t, $^3J_{HH}$ = 7.2 Hz, 1H, H-C11), 3.09 (p, $^3J_{HH}$ = 9.2 Hz, 1H, H-C3), 3.04 – 2.98 (m, 2H, $H^a$-C2), 2.59 – 2.53 (m, 2H, $H^b$-C2), 2.14 (s, 1H, H-O).

**$^{13}$C{$^1$H} NMR** (151 MHz, $CDCl_3$): $\delta$ [ppm] = 144.6 (C4), 144.5 (C8), 131.9 (C5), 128.6 (C10), 127.5 (C6), 126.8 (C9), 126.4 (C11), 121.7 (C7), 72.3 (C1), 44.9 (C2), 30.0 (C3).

**ESI-MS:** (*m/z*) requires: [($C_{16}H_{15}BrONa)^+$] = 325.0198, (*m/z*) found: [($C_{16}H_{15}BrONa)^+$] = 325.0198.

**FT-IR** ($\tilde{\nu}$ = cm$^{-1}$)**:** 3293.1 (broad), 1591.3 (w), 1462.2 (w), 1446.4 (w), 1430.6 (w), 1394.7 (w), 1272.8 (w), 1236.9 (m), 1185.2 (w), 1159.4 (m), 1084.8 (m), 1051.8 (w), 1008.7 (s), 958.5 (m), 898.3 (m), 826.5 (s), 802.1 (m), 744.7 (s), 724.6 (m).

**3-Phenyl-1-(4-(trifluoromethoxy)phenyl)cyclobutan-1-ol (1i)**

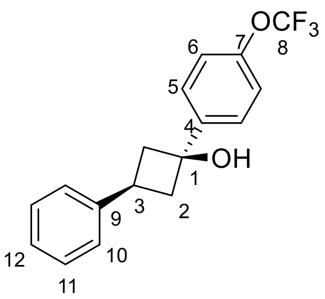


Compound **1i** was prepared following general procedure **C** using 1-bromo-4-(trifluoromethoxy)benzene (578 mg, 2.40 mmol) and 3-phenylcyclobutan-1-one **S1** (292 mg, 2.00 mmol). The crude product was purified by flash column chromatography (10-15% EtOAc in cyclohexane) to yield product **1i** as a pale-yellow solid (single diastereoisomer, 430 mg, 1.40 mmol, 70%).

**R*f*** = 0.47 (20% EtOAc in cyclohexane).

**Melting Point:** 65-67 ˚C.

**$^{1}$H NMR** (500 MHz, $CDCl_3$): $\delta$[ppm] = 7.70 – 7.66 (m, 2H, H-C6), 7.39 – 7.35 (m, 2H, H-C10), 7.34 – 7.28 (m, 4H, H-C5, H-C11), 7.28 – 7.24 (m, 1H, H-C12), 3.14 (p, $^{3}J_{HH}$ = 9.1 Hz, 1H, H-C3), 3.08 – 3.02 (m, 2H, $H^{a}$-C2), 2.63 – 2.56 (m, 2H, $H^{b}$-C2), 2.36 (s, 1H, H-O).

**$^{19}$F NMR** (376 MHz, $CDCl_3$): $\delta$[ppm] = -57.83 (t, $^{5}J_{FH}$ = 1.1 Hz, 3F, F-C8).

**$^{19}$F{$^{1}$H} NMR** (376 MHz, $CDCl_3$): $\delta$[ppm] = -57.83 (s, 3F, F-C8).

**$^{13}$C{$^{1}$H} NMR** (126 MHz, $CDCl_3$): $\delta$[ppm] = 144.7 (q, $^{3}J_{CF}$ = 1.8 Hz, C7), 144.4 (C9), 144.2 (C4), 128.6 (C10), 127.2 (C6), 126.8 (C11), 126.4 (C12), 121.1 (q, $^{5}J_{CF}$ = 1.1 Hz, C5), 120.7 (q, $^{1}J_{CF}$ = 257.2 Hz, C8), 72.2 (C1), 44.9 (C2), 30.0 (C3).

**ESI-MS:** (*m/z*) requires: [$(C_{17}H_{15}F_3O_2Na)^+$] = 331.0916, (*m/z*) found: [$(C_{17}H_{15}F_3O_2Na)^+$] = 331.0916.

**FT-IR** ($\tilde{\nu}$ = $cm^{-1}$)**:** 3288.8 (broad), 1511.0 (w), 1496.6 (w), 1275.6 (m), 1208.2 (s), 1156.5 (s), 1089.1 (m), 1080.5 (m), 1017.4 (m), 952.8 (w), 896.8 (w), 853.8 (m), 820.8 (m), 789.2 (w), 749.0 (s).

**3-Phenyl-1-(4-(trifluoromethyl)phenyl)cyclobutan-1-ol (1j)**

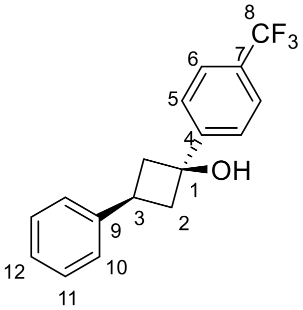


Compound **1j** was prepared following general procedure **C** using 1-bromo-4-(trifluoromethyl)benzene (1.62 g, 7.20 mmol) and 3-phenylcyclobutan-1-one **S1** (876 mg, 6.00 mmol). The crude product was purified by flash column chromatography (10-12.5% EtOAc in cyclohexane) to yield product **1j** as an orange solid (single diastereoisomer, 1.38 g, 4.72 mmol, 79%).

**R*f*** = 0.40 (20% EtOAc in cyclohexane).

**Melting Point:** 61-63 ˚C.

**$^1$H NMR** (599 MHz, $CDCl_3$): $\delta$ [ppm] = 7.77 (d, $^3J_{HH}$ = 8.2 Hz, 2H, H-C5), 7.70 (d, $^3J_{HH}$ = 8.2 Hz, 2H, H-C6), 7.35 (t, $^3J_{HH}$ = 7.4 Hz, 2H, H-C11), 7.30 (d, $^3J_{HH}$ = 7.5 Hz, 2H, H-C10), 7.24 (td, $^3J_{HH}$ = 7.2 Hz, $^4J_{HH}$ = 1.4 Hz, 1H, H-C12), 3.16 (p, $^3J_{HH}$ = 9.1 Hz, 1H, H-C3), 3.05 (ddt, $^2J_{HH}$ = 11.8 Hz, $^3J_{HH}$ = 8.1 Hz, $^4J_{HH}$ = 2.4 Hz, 2H, $H^a$-C2), 2.61 (ddt, $^2J_{HH}$ = 12.0 Hz, $^3J_{HH}$ = 9.4 Hz, $^4J_{HH}$ = 2.5 Hz, 2H, $H^b$-C2), 2.18 (s, 1H, H-O).

**$^{19}$F NMR** (564 MHz, $CDCl_3$): $\delta$ [ppm] = -62.51 (s, 3F, F-C8).

**$^{19}$F{$^1$H} NMR** (376 MHz, $CDCl_3$): $\delta$ [ppm] = -62.52 (s, 3F, F-C8).

**$^{13}$C{$^1$H} NMR** (151 MHz, $CDCl_3$): $\delta$ [ppm] = 149.6 (q, $^5J_{CF}$ = 1.3 Hz, C4), 144.4 (C9), 129.9 (q, $^2J_{CF}$ = 32.4 Hz, C7), 128.6 (C11), 126.8 (C10), 126.5 (C12), 125.9 (C5), 125.8 (q, $^3J_{CF}$ = 3.8 Hz, C6), 124.3 (q, $^1J_{CF}$ = 272.0 Hz, C8), 72.4 (C1), 45.0 (C2), 30.0 (C3).

**GC-EI-MS:** Retention 9.88 min**,** (*m/z*) requires: [$(C_{17}H_{15}F_3O)^+$] = 292.1070, (*m/z*) found: [$(C_{17}H_{15}F_3O)^+$] = 292.1072.

**FT-IR** ($\tilde{\nu}$ = $cm^{-1}$)**:** 3277.3 (broad), 1618.6 (w), 1495.2 (w), 1324.4 (s), 1301.5 (w), 1231.2 (w), 1169.5 (m), 1159.4 (m), 1126.4 (s), 1087.7 (m), 1061.8 (m), 1013.1 (m), 1001.6 (w), 845.2 (s), 815.0 (w), 773.4 (w), 751.9 (s), 743.3 (m).

**4-(1-Hydroxy-3-phenylcyclobutyl)benzonitrile (1k)**

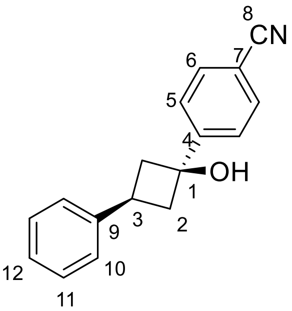


Compound **1k** was prepared following a patent procedure.[12]

4-Iodobenzonitrile (458 mg, 2.00 mmol, 1.0 eq.) was dissolved in dry THF (7 mL). The mixture was cooled to 0 ˚C and *i*-PrMgCl•LiCl in dry THF (1.3 M, 1.54 mL) was added dropwise. After complete addition, the mixture was stirred at room temperature for 1 h. Subsequently, the mixture was cooled to 0 ˚C and 3-phenylcyclobutan-1-one **S1** (292 mg, 2.00 mmol, 1.0 eq.) was added as a solution in dry THF (1 mL). The reaction was warmed to room temperature and stirred for 15 h before it was quenched by adding saturated aqueous $NH_4Cl$ (30 mL). EtOAc (20 mL) was added and the layers were separated. The aqueous layer was extracted with additional EtOAc (2x 30 mL). The combined organic layers were dried over $Na_2SO_4$ and the solvent was removed under reduced pressure. The crude product was purified by flash column chromatography (10-20% EtOAc in cyclohexane) to yield product **1k** as a colorless crystalline solid (single diastereoisomer, 320 mg, 1.28 mmol, 64%).

**R*f*** = 0.30 (30% EtOAc in cyclohexane).

**Melting Point:** 118-120 ˚C.

**$^{1}$H NMR** (599 MHz, $CDCl_3$): $\delta$[ppm] = 7.78 – 7.74 (m, 2H, H-C5), 7.73 – 7.70 (m, 2H, H-C6), 7.37 – 7.33 (m, 2H, H-C11), 7.31 – 7.27 (m, 2H, H-C10), 7.26 – 7.21 (m, 1H, H-C12), 3.18 (p, $^{3}J_{HH}$ = 9.2 Hz, 1H, H-C3), 3.02 (ddt, $^{2}J_{HH}$ = 13.5 Hz, $^{3}J_{HH}$ = 8.0 Hz, $^{4}J_{HH}$ = 2.3 Hz, 2H, $H^{a}$-C2), 2.61 (ddt, $^{2}J_{HH}$ = 12.3 Hz, $^{3}J_{HH}$ = 9.4 Hz, $^{4}J_{HH}$ = 2.4 Hz, 2H, $H^{b}$-C2), 2.32 (s, 1H, H-O).

**$^{13}$C{$^{1}$H} NMR** (151 MHz, $CDCl_3$): $\delta$[ppm] = 151.0 (C4), 144.2 (C9), 132.6 (C6), 128.7 (C11), 126.7 (C10), 126.5 (C12), 126.2 (C5), 118.9 (C8), 111.4 (C7), 72.4 (C1), 45.2 (C2), 30.0 (C3).

**ESI-MS:** (*m/z*) requires: [$(C_{17}H_{14}NO)^{-}$] = 248.1081, (*m/z*) found [$(C_{17}H_{14}NO)^{-}$] = 248.1080.

**FT-IR** ($\tilde{\nu}$ = $cm^{-1}$)**:** 3462.5 (broad), 2235.6 (m), 1602.8 (w), 1492.3 (w), 1445.0 (w), 1310.1 (w), 1242.6 (m), 1160.9 (m), 1093.4 (m), 1077.6 (m), 1054.7 (w), 1034.6 (m), 1014.5 (w), 949.9 (w), 899.7 (w), 882.5 (w), 849.5 (s), 829.4 (s), 819.3 (m), 809.3 (s), 770.6 (m), 744.7 (s).

**3-Phenyl-1-(4'-(trifluoromethyl)-[1,1'-biphenyl]-4-yl)cyclobutan-1-ol (1l)**

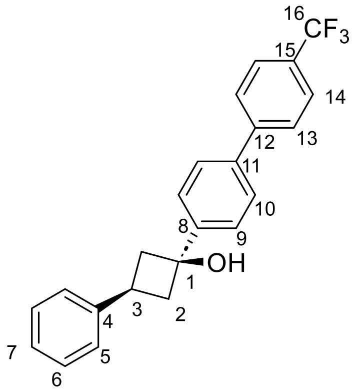


Compound **1l** was prepared following general procedure **C** using 4-bromo-4'-(trifluoromethyl)-1,1'-biphenyl **S8** (384 mg, 1.64 mmol) and 3-phenylcyclobutan-1-one **S1** (200 mg, 1.37 mmol). The crude product was purified by flash column chromatography (20% EtOAc in *n*-pentane) to yield product **1l** as a colorless solid (mixture of diastereoisomers, d.r. = 92:08, 374 mg, 1.01 mmol, 74%).

**$R_f$** = 0.23 (10% EtOAc in *n*-pentane).

**Melting Point:** 121-123 °C.

**$^{1}$H NMR** (500 MHz, $CDCl_3$): $\delta$[ppm] = 7.75 – 7.72 (m, 2H, H-C9), 7.71 – 7.70 (m, 4H, H-C13, H-C14), 7.69 – 7.64 (m, 2H, H-C10), 7.36 – 7.28 (m, 4H, H-C5, H-C6), 7.26 – 7.19 (m, 1H, H-C7), 3.17 – 3.04 (m, 3H, $H^{a}$-C2, H-C3), 2.62 – 2.56 (m, 2H, $H^{b}$-C2), 2.20 (s, 1H, OH).

**$^{19}$F NMR** (376 MHz, $CDCl_3$): $\delta$[ppm] = -62.39 (s, 3F, F-C16).

**$^{19}$F{$^{1}$H} NMR** (376 MHz, $CDCl_3$): $\delta$[ppm] = -62.39 (s, 3F, F-C16).

**$^{13}$C{$^{1}$H} NMR** (126 MHz, $CDCl_3$): $\delta$[ppm] = 145.6 (C8), 144.6 (C4), 144.3 (C11), 139.2 (C12), 129.6 (q, $^{2}J_{CF}$ = 32.7 Hz, C15), 128.6 (C6), 127.7 (C10), 127.5 (C13), 126.8 (C5), 126.4 (C7), 126.3 (C9), 125.9 (q, $^{3}J_{CF}$ = 3.8 Hz, C14), 124.4 (q, $^{1}J_{CF}$ = 271.9 Hz, C16), 72.5 (C1), 44.9 (C2), 30.2 (C3).

**ESI-MS:** (*m/z*) requires: [$(C_{23}H_{18}F_3O)^{-}$] = 367.1315, (*m/z*) found: [$(C_{23}H_{18}F_3O)^{-}$] = 367.1312.

**FT-IR** ($\tilde{\nu}$ = cm$^{-1}$)**:** 2976.01 (broad), 1493.75 (w), 1328.73 (s), 1235.46 (m), 1163.72 (m), 112.06 (s), 1071.88 (s), 830.82 (s), 756.20 (m), 728.94 (w), 698.80 (m).

**1-(3-Bromophenyl)-3-phenylcyclobutan-1-ol (1m)**

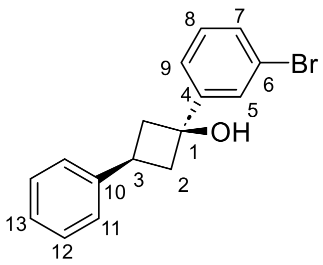


Compound **1m** was prepared following general procedure **C** using 1,3-dibromobenzene (377 mg, 1.60 mmol) and 3-phenylcyclobutan-1-one **S1** (200 mg, 1.36 mmol). The crude product was purified by flash column chromatography (5-20% EtOAc in *n*-pentane) to yield product **1m** as a colorless solid (mixture of diastereoisomers, d.r. = 93:07, 250 mg, 0.82 mmol, 60%).

**R*f*** = 0.38 (10% EtOAc in *n*-pentane).

**Melting Point:** 87-89 °C.

**$^{1}$H NMR** (599 MHz, $CDCl_3$): $\delta$ [ppm] = 7.79 (t, $^{4}J_{HH}$ = 1.9 Hz, 1H, H-C5), 7.57 (ddd, $^{3}J_{HH}$ = 7.8 Hz, $^{4}J_{HH}$ = 1.8 Hz, $J_{HH}$ = 1.0 Hz, 1H, H-C9), 7.48 (ddd, $^{3}J_{HH}$ = 8.0 Hz, $^{4}J_{HH}$ = 2.0 Hz, $J_{HH}$ = 1.0 Hz, 1H, H-C7), 7.36 – 7.28 (m, 5H, H-C8, H-C11, H-C12), 7.23 (td, $^{3}J_{HH}$ = 7.7 Hz, $J_{HH}$ = 1.5 Hz, 1H, H-C13), 3.16 – 3.09 (m, 1H, H-C3), 3.05 – 2.99 (m, 2H, H$^{a}$-C2), 2.60 – 2.53 (m, 2H, H$^{b}$-C2), 2.13 (s, 1H, OH).

**$^{13}$C{$^{1}$H} NMR** (151 MHz, $CDCl_3$): $\delta$ [ppm] = 148.0 (C4), 144.5 (C10), 130.8 (C7), 130.4 (C8), 129.0 (C5), 128.6 (C12), 126.8 (C11), 126.4 (C13), 124.2 (C9), 123.0 (C6), 72.4 (C1), 44.9 (C2), 30.0 (C3).

**GC-EI-MS:** Retention 11.05 min**,** (*m/z*) requires: [($C_{16}H_{15}BrO$)$^{+}$] = 302.0301, (*m/z*) found: [($C_{16}H_{15}BrO$)$^{+}$] = 302.0301.

**FT-IR** ($\tilde{\nu}$ = cm$^{-1}$)**:** 3264.43 (broad), 2360.43 (m), 1495.18 (w), 1407.65 (w), 1267.03 (w), 1241.20 (m), 1080.49 (m), 1064.71 (m), 1028.83 (w), 952.78 (w), 878.17 (w), 793.51 (s), 749.03 (s), 737.55 (s), 695.963 (s), 667.24 (m).

**1-(2-Bromophenyl)-3-phenylcyclobutan-1-ol (1n)**

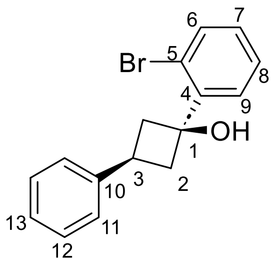


Compound **1n** was prepared following general procedure **C** using 1-bromo-2-iodobenzene (594 mg, 1.60 mmol) and 3-phenylcyclobutan-1-one **S1** (200 mg, 1.36 mmol). The crude product was purified by flash column chromatography (5-10% EtOAc in *n*-pentane) to yield product **1n** as a viscous, colorless oil (single diastereoisomer, 93 mg, 0.30 mmol, 22%).

**R*f*** = 0.45 (10% EtOAc in *n*-pentane).

**$^{1}$H NMR** (599 MHz, $CDCl_3$): $\delta$ [ppm] = 7.70 – 7.65 (m, 2H, H-C6, H-C9), 7.40 (td, $^{3}J_{HH}$ = 7.5 Hz, $^{4}J_{HH}$ = 1.3 Hz, 1H, H-C8), 7.37 – 7.32 (m, 4H, H-C11, H-C12), 7.26 – 7.21 (m, 2H, H-C7, H-C13), 3.31 (s, 1H, O-H), 3.29 – 3.24 (m, 2H, $H^{a}$-C2), 3.01 (p, $^{3}J_{HH}$ = 8.8 Hz, 2H, H-C3), 2.68 – 2.61 (m, 2H, $H^{b}$-C2).

**$^{13}$C{$^{1}$H} NMR** (151 MHz, $CDCl_3$): $\delta$ [ppm] = 144.6 (C10), 142.3 (C4), 134.9 (C9), 129.6 (C7), 128.6 (C12), 127.7 (C8), 127.2 (C6), 126.9 (C11), 126.4 (C13), 123.2 (C5), 74.2 (C1), 43.2 (C2), 30.9 (C3).

**GC-EI-MS:** Retention 11.00 min**,** (*m/z*) requires: [$(C_{16}H_{14}BrO)^{-}$] = 302.0301, (*m/z*) found: [$(C_{16}H_{14}BrO)^{-}$] = 302.0221.

**FT-IR** ($\tilde{\nu}$ = $cm^{-1}$)**:** 3377.79 (broad), 30559.24 (w), 3026.23 (w), 2981.75 (w), 2934.40 (w), 2360.43 (w), 1602.80 (w), 1565.49 (w), 1495.18 (m), 1469.35 (m), 1456.44 (m), 1426.30 (m), 1325.86 (m), 1275.64 (m), 1261.16 (m), 1157.98 (m), 1127.84 (w), 1076.19 (m), 1023.09 (s), 954.22 (w), 941.30 (w), 892.52 (w), 815.03 (m), 749.03 (s), 726.07 (s), 697.37 (s), 668.67 (s), 658.63 (m), 642.84 (m).

**3-(4-Fluorophenyl)-1-phenylcyclobutan-1-ol (1o)**

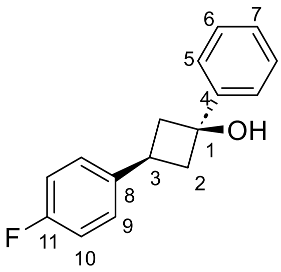


Compound **1o** was prepared following general procedure **C** using bromobenzene (377 mg, 2.40 mmol) and 3-(4-fluorophenyl)cyclobutan-1-one **S2** (328 mg, 2.00 mmol). The crude product was purified by flash column chromatography (10-15% EtOAc in cyclohexane) to yield product **1o** as a colorless solid (single diastereoisomer, 323 mg, 1.33 mmol, 67%).

**$R_f$** = 0.42 (20% EtOAc in cyclohexane).

**Melting Point:** 67-69 ˚C.

**$^{1}$H NMR** (500 MHz, $CDCl_3$): $\delta$ [ppm] = 7.66 – 7.62 (m, 2H, H-C5), 7.48 – 7.40 (m, 2H, H-C6), 7.38 – 7.32 (m, 1H, H-C7), 7.26 – 7.22 (m, 2H, H-C9), 7.06 – 6.97 (m, 2H, H-C10), 3.15 – 2.97 (m, 3H, $H^{a}$-C2, H-C3), 2.64 – 2.45 (m, 2H, $H^{b}$-C2), 2.13 (s, 1H, H-O).

**$^{19}$F NMR** (376 MHz, $CDCl_3$): -117.25 (tt, $^{3}J_{HF}$ = 8.7 Hz, $^{4}J_{HF}$ = 5.3 Hz, 1F, F-C11).

**$^{19}$F{$^{1}$H} NMR** (376 MHz, $CDCl_3$): -117.26 (s, 1F, F-C11).

**$^{13}$C{$^{1}$H} NMR** (126 MHz, $CDCl_3$): $\delta$ [ppm] = 161.5 (d, $^{1}J_{HF}$ = 243.9 Hz, C11), 145.4 (C4), 140.5 (d, $^{4}J_{HF}$ = 3.1 Hz, C8), 128.8 (C6), 128.2 (d, $^{3}J_{HF}$ = 7.8 Hz, C9), 127.9 (C7), 125.6 (C5), 115.3 (d, $^{2}J_{HF}$ = 21.2 Hz, C10), 72.6 (C1), 44.9 (C2), 29.5 (C3).

**GC-EI-MS:** Retention 9.19 min; (*m/z*) requires: [($C_{16}H_{15}FO)^+$] = 242.1101, (*m/z*) found: [($C_{16}H_{15}FO)^+$] = 242.1102.

**FT-IR** ($\tilde{\nu}$ = cm$^{-1}$)**:** 3280.2 (broad), 1605.7 (w), 1509.5 (s), 1457.9 (w), 1446.4 (w), 1278.5 (w), 1242.6 (m), 1222.6 (s), 1186.7 (w), 1159.4 (m), 1073.3 (m), 1048.9 (w), 1027.4 (w), 1017.4 (w), 954.2 (w), 915.5 (w), 898.3 (w), 835.1 (s), 816.5 (m), 792.1 (m), 759.1 (s), 700.2 (s).

**3-(4-Chlorophenyl)-1-phenylcyclobutan-1-ol (1p)**

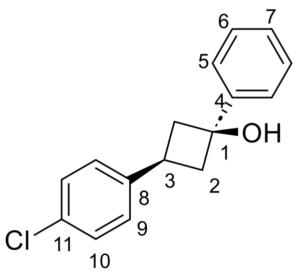


Compound **1p** was prepared following general procedure **C** using bromobenzene (424 mg, 2.70 mmol) and 3-(4-chlorophenyl)cyclobutan-1-one **S3** (400 mg, 2.20 mmol). The crude product was purified by flash column chromatography (10-20% EtOAc in *n*-pentane) to yield product **1p** as a colorless solid (single diastereoisomer, 411 mg, 1.59 mmol, 72%).

**R*f*** = 0.30 (10% EtOAc in *n*-pentane).

**Melting Point**: 72-74 °C

**$^1$H NMR** (599 MHz, $CDCl_3$): $\delta$[ppm] = 7.63 (d, $^3J_{HH}$ = 7.2 Hz, 2H, H-C5), 7.45 (t, $^3J_{HH}$ = 7.9 Hz, 2H, H-C6), 7.38 – 7.34 (m, 1H, H-C7), 7.30 (d, $^3J_{HH}$ = 8.4 Hz, 2H, H-C10), 7.22 (d, $^3J_{HH}$ = 8.5 Hz, 2H, H-C9), 3.10 – 3.01 (m, 3H, H$^a$-C2, H-C3), 2.57 – 2.47 (m, 2H, H$^b$-C2), 2.34 (s, 1H, O-H).

**$^{13}$C{$^1$H} NMR** (151 MHz, $CDCl_3$): $\delta$[ppm] = 145.2 (C4), 143.3 (C8), 131.9 (C11), 128.8 (C6), 128.6 (C10), 128.2 (C9), 127.8 (C7), 125.6 (C5), 72.5 (C1), 44.6 (C2), 29.6 (C3).

**ESI-MS:** (*m/z*) requires: [($C_{16}H_{14}OCl)^-$] = 257.0739, (*m/z*) found: [($C_{16}H_{14}OCl)^-$] = 257.0737.

**FT-IR** ($\tilde{\nu}$ = cm$^{-1}$)**:** 3271.6 (broad), 1492.3 (m), 1445.0 (w), 1400.5 (w), 1278.5 (w), 1241.2 (m), 1162.3 (w), 1090.5 (m), 1079.1 (m), 1048.9 (m), 1027.4 (m), 1013.1 (m), 970.0 (w), 952.8 (w), 915.5 (w), 896.8 (w), 830.8 (s), 802.1 (w), 761.1 (s), 727.5 (m).

**3-(4-Bromophenyl)-1-phenylcyclobutan-1-ol (1q)**

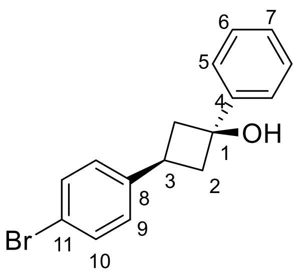


Compound **1q** was prepared following general procedure **C** using bromobenzene (377 mg, 2.40 mmol, 1.2 eq.) and 3-(4-bromophenyl)cyclobutan-1-one **S5** (450 mg, 2.00 mmol, 1.0 eq.). The crude product was purified by flash column chromatography (10-15% EtOAc in cyclohexane) to yield product **1q** as a colorless solid (single diastereoisomer, 367 mg, 1.24 mmol, 62%).

**R*f*** = 0.42 (20% EtOAc in cyclohexane).

**Melting Point:** 84-86 ˚C.

**$^1$H NMR** (599 MHz, $CDCl_3$): $\delta$ [ppm] = 7.64 – 7.61 (m, 2H, H-C5), 7.46 – 7.42 (m, 4H, H-C6, H-C10), 7.37 – 7.33 (m, 1H, H-C7), 7.18 – 7.15 (m, 2H, H-C9), 3.09 – 3.00 (m, 3H, $H^a$-C2, H-C3), 2.56 – 2.49 (m, 2H, $H^b$-C2), 2.15 (s, 1H, H-O).

**$^{13}$C{$^1$H} NMR** (151 MHz, $CDCl_3$): $\delta$ [ppm] = 145.2 (C8), 143.8 (C4), 131.6 (C10), 128.9 (C6), 128.6 (C9), 127.9 (C7), 125.6 (C5), 120.0 (C11), 72.6 (C1), 44.6 (C2), 29.7 (C3).

**GC-EI-MS:** Retention 11.14 min; (*m/z*) requires: [$(C_{16}H_{15}BrO)^+$] = 302.0301, (*m/z*) found: [$(C_{16}H_{15}BrO)^+$] = 302.0301.

**FT-IR** ($\tilde{\nu}$ = $cm^{-1}$)**:** 3287.4 (broad), 1789.3 (w), 1617.2 (w), 1494.2 (w), 1446.4 (w), 1412.0 (w), 1324.4 (s), 1278.5 (w), 1241.2 (w), 1191.0 (w), 1158.0 (m), 1112.1 (s), 1079.1 (m), 1063.3 (s), 1028.8 (w), 1015.9 (m), 971.4 (w), 955.7 (w), 916.9 (w), 898.3 (w), 839.4 (s), 813.6 (w), 772.0 (m), 759.1 (m), 737.6 (w).

**1-Phenyl-3-(4-(trifluoromethyl)phenyl)cyclobutan-1-ol (1r)**

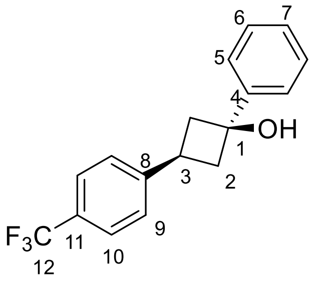


Compound **1r** was prepared following general procedure **C** using bromobenzene (339 mg, 2.16 mmol, 1.2 eq.) and 3-(4-(trifluoromethyl)phenyl)cyclobutan-1-one **S7** (385 mg, 1.80 mmol, 1.0 eq.). The crude product was purified by flash column chromatography (10-15% EtOAc in cyclohexane and 100% DCM to remove further minor impurities) to yield product **1r** as a white solid (single diastereoisomer, 311 mg, 1.06 mmol, 59%).

**R*f*** = 0.33 (20% EtOAc in cyclohexane).

**Melting Point:** 84-86 ˚C.

**$^1$H NMR** (500 MHz, $CDCl_3$): $\delta$ [ppm] = 7.66 – 7.62 (m, 2H, H-C5), 7.59 (d, $^3J_{HH}$ = 8.2 Hz, 2H, H-C10), 7.48 – 7.42 (m, 2H, H-C6), 7.40 (d, $^3J_{HH}$ = 8.0 Hz, 2H, H-C9), 7.37 (tt, $^3J_{HH}$ = 7.4 Hz, $J_{HH}$ = 1.3 Hz, 1H, H-C7), 3.18 – 3.07 (m, 3H, $H^a$-C2, H-C3), 2.62 – 2.54 (m, 2H, $H^b$-C2), 2.23 – 2.20 (m, 1H, H-O).

**$^{19}$F NMR** (376 MHz, $CDCl_3$): $\delta$ [ppm] = -62.33 (s, 3F, F-C12).

**$^{19}$F{$^1$H} NMR** (376 MHz, $CDCl_3$): $\delta$ [ppm] = -62.33 (s, 3F, F-C12).

**$^{13}C\{^1H\}$ NMR** (126 MHz, $CDCl_3$): $\delta$ [ppm] = 148.9 (q, $^5J_{CF}$ = 1.3 Hz, C8), 145.1 (C4), 128.9 (C6), 128.6 (q, $^2J_{CF}$ = 32.4 Hz, C11), 128.0 (C6), 127.2 (C9), 125.6 (C5), 125.5 (q, $^3J_{CF}$ = 3.8 Hz, C10), 124.8 (q, $^1J_{CF}$ = 271.9 Hz, C12), 72.6 (C1), 44.4 (C2), 30.1 (C3).

**ESI-MS:** (*m/z*) requires: [$(C_{17}H_{15}F_3ONa)^+$] = 315.0967, (*m/z*) found: [$(C_{17}H_{15}F_3ONa)^+$] = 315.0965.

**FT-IR** ($\tilde{\nu}$ = $cm^{-1}$)**:** 3297.4 (broad), 1789.3 (w), 1617.2 (w), 1495.2 (w), 1446.4 (w), 1412.0 (w), 1325.9 (s), 1278.5 (w), 1241.2 (w), 1191.0 (w), 1158.0 (m), 1112.1 (s), 1063.3 (s), 1028.8 (w), 1015.9 (m), 971.4 (w), 954.2 (w), 916.9 (w), 839.4 (s), 813.6 (w), 772.0 (w), 759.1 (m), 737.6 (w).

**4-(3-Hydroxy-3-phenylcyclobutyl)phenyl trifluoromethanesulfonate (1s)**

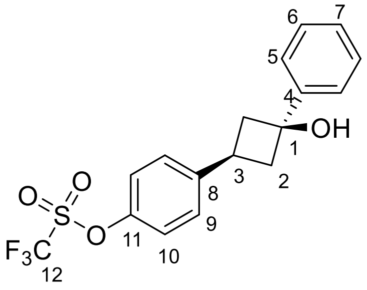


Compound **1s** was prepared following modified general procedure **C**.

4-(3-Oxocyclobutyl)phenyl trifluoromethanesulfonate **S11** (588 mg, 3.00 mmol, 1.0 eq.) dissolved in dry THF (12.0 mL). The reaction mixture was cooled to -78 ˚C and PhMgBr in dry THF (1 M, 3.0 mL, 3.00 mmol, 1.0 eq.) was added. The reaction was stirred for 1 h before it was warmed to room temperature. After stirring for additional 2 h, the reaction was quenched by adding saturated aqueous $NH_4Cl$ and diluted with additional EtOAc. The layers were separated and the aqueous layer was extracted with EtOAc (2x). The combined organic layers were dried over $Na_2SO_4$ and the solvent was removed under reduced pressure. The crude product was purified by flash column chromatography (10-15% EtOAc in cyclohexane) to yield product **1s** as a pale-yellow powder (single diastereoisomer, 593 mg, 1.59 mmol, 53%).

**$R_f$** = 0.36 (30% EtOAc in cyclohexane).

**Melting Point:** 68-70 ˚C.

**$^1H$ NMR** (500 MHz, $CDCl_3$): $\delta$ [ppm] = 7.65 – 7.60 (m, 2H, H-C5), 7.48 – 7.42 (m, 2H, H-C6), 7.39 – 7.33 (m, 3H, H-C7, H-C9), 7.25 – 7.20 (m, 2H, H-C10), 3.15 – 3.05 (m, 3H, $H^a$-C2, H-C3), 2.60 – 2.49 (m, 2H, $H^b$-C2), 2.21 (s, 1H, H-O).

**$^{19}F$ NMR** (376 MHz, $CDCl_3$): $\delta$ [ppm] = -72.85 (s, 3F, F-C12).

**$^{19}F\{^1H\}$ NMR** (376 MHz, $CDCl_3$): $\delta$ [ppm] = -72.85 (s, 3F, F-C12).

**$^{13}C\{^1H\}$ NMR** (126 MHz, $CDCl_3$): $\delta$ [ppm] = 148.0 (C11), 145.4 (C8), 145.1 (C4), 128.9 (C6), 128.6 (C9), 128.0 (C7), 125.6 (C5), 121.3 (C10), 118.9 (q, $^1J_{HF}$ = 320.9 Hz, C12), 72.57 (C1), 44.6 (C2), 29.7 (C3).

**ESI-MS:** (*m/z*) requires: [$(C_{17}H_{15}F_3O_4SNa)^+$] = 395.0535, (*m/z*) found: [$(C_{17}H_{15}F_3O_4SNa)^+$] = 395.0532.

**FT-IR** ($\tilde{\nu}$ = cm$^{-1}$)**:** 3306.0 (broad), 1503.8 (w), 1427.7 (s), 1249.8 (m), 1221.1 (s), 1182.4 (w), 1162.3 (w), 1135.0 (s), 1073.3 (m), 1030.3 (w), 1018.8 (w), 886.8 (s), 846.6 (m), 830.8 (w), 816.5 (w), 782.0 (w), 767.7 (m), 747.6 (m), 713.2 (w), 701.7 (m).

**1-(4-Bromophenyl)-3-(4-fluorophenyl)cyclobutan-1-ol (1t)**

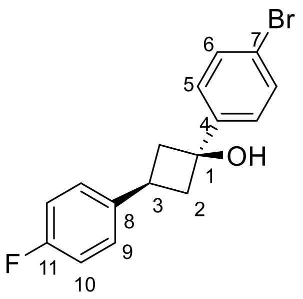


Compound **1t** was prepared following general procedure **C** using 1,4-dibromobenzene (566 mg, 2.40 mmol) and 3-(4-fluorophenyl)cyclobutan-1-one **S2** (329 mg, 2.00 mmol, 1.0 eq.). The crude product was purified by flash column chromatography (10-15% EtOAc in cyclohexane) to yield product **1t** as a pale-yellow solid (single diastereoisomer, 361 mg, 1.12 mmol, 56%).

**R*f*** = 0.45 (20% EtOAc in cyclohexane).

**Melting Point:** 69-71 ˚C.

**$^1$H NMR** (500 MHz, $CDCl_3$): $\delta$[ppm] = 7.57 – 7.53 (m, 2H, H-C6), 7.51 – 7.47 (m, 2H, H-C5), 7.25 – 7.20 (m, 2H, H-C9), 7.04 – 6.98 (m, 2H, H-C10), 3.11 – 2.95 (m, 3H, H$^a$-C2, H-C3), 2.54 – 2.47 (m, 2H, H$^b$-C2), 2.27 (s, 1H, H-O).

**$^{19}$F NMR** (376 MHz, $CDCl_3$): $\delta$[ppm] = -116.96 (tt, $^3J_{CF}$ = 8.9 Hz, $^3J_{CF}$ = 5.4 Hz ,1F, F-C11).

**$^{19}$F{$^1$H} NMR** (376 MHz, $CDCl_3$): $\delta$[ppm] = -116.96 (s, 1F, F-C11).

**$^{13}$C{$^1$H} NMR** (126 MHz, $CDCl_3$): $\delta$ [ppm] = 161.5 (d, $^1J_{CF}$ = 244.1 Hz, C11), 144.4 (C4), 140.1 (d, $^4J_{CF}$ = 3.1 Hz, C8), 131.9 (C6), 128.2 (d, $^3J_{CF}$ = 7.8 Hz, C9), 127.4 (C5), 121.8 (C7), 115.3 (d, $^2J_{CF}$ = 21.1 Hz, C10), 72.2 (C1), 44.9 (C2), 29.4 (C3).

**GC-EI-MS:** Retention 11.12 min, (*m/z*) requires: [($C_{16}H_{14}BrFO)^+$] = 322.0187, (*m/z*) found: [($C_{16}H_{14}BrFO)^+$] = 322.0183.

**FT-IR** ($\tilde{\nu}$ = cm$^{-1}$)**:** 3265.9 (broad), 1604.2 (w), 1508.1 (s), 1489.4 (w), 1394.7 (w), 1337.3 (w), 1272.8 (w), 1222.6 (m), 1185.2 (w), 1156.5 (m), 1083.4 (m), 1036.0 (w), 1008.7 (m), 957.1 (w), 899.7 (w), 840.9 (s), 823.6 (s), 815.0 (s), 784.9 (s), 760.5 (w), 723.2 (m), 701.7 (w).

**1-(4-Bromophenyl)-3-(4-chlorophenyl)cyclobutan-1-ol (1u)**

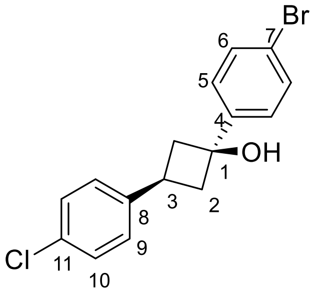

Compound **1u** was prepared following general procedure **C** using 1,4-dibromobenzene (566 mg, 2.40 mmol) and 3-(4-chlorophenyl)cyclobutan-1-one **S3** (361 mg, 2.00 mmol, 1.0 eq.). The crude product was purified by flash column chromatography (10-15% EtOAc in cyclohexane) to yield product **1u** as a pale-yellow solid (single diastereoisomer, 423 mg, 1.25 mmol, 63%).

**R*f*** = 0.42 (20% EtOAc in cyclohexane).

**Melting Point:** 85-87 ˚C.

**$^1$H NMR** (500 MHz, $CDCl_3$): $\delta$[ppm] = 7.57 – 7.53 (m, 2H, H-C6), 7.52 – 7.47 (m, 2H, H-C5), 7.31 – 7.27 (m, 2H, H-C10), 7.23 – 7.18 (m, 2H, H-C9), 3.09 – 2.96 (m, 3H, $H^a$-C2, H-C3), 2.56 – 2.47 (m, 2H, $H^b$-C2), 2.13 (s, 1H, H-O).

**$^{13}$C{$^1$H} NMR** (126 MHz, $CDCl_3$): $\delta$[ppm] = 144.4 (C4), 143.0 (C8), 132.1 (C11), 131.9 (C6), 128.7 (C10), 128.2 (C9), 127.4 (C5), 121.8 (C7), 72.3 (C1), 44.8 (C2), 29.6 (C3).

**GC-EI-MS:** Retention 12.08 min, (*m/z*) requires: $[(C_{16}H_{14}BrClO)^+]$ = 335.9911, (*m/z*) found: $[(C_{16}H_{14}BrClO)^+]$ = 335.9915.

**FT-IR** ($\tilde{\nu}$ = cm$^{-1}$)**:** 3290.3 (broad), 2968.8 (w), 2933.0 (w), 1486.0 (m), 1432.0 (w), 1394.7 (w), 1234.0 (m), 1159.4 (w), 1074.8 (s), 1007.3 (s), 952.8 (w), 889.7 (w), 836.6 (m), 825.1 (s), 802.1 (m), 736.1 (m), 713.2 (w).

**1-(4-Bromophenyl)-3-(4-(trifluoromethyl)phenyl)cyclobutan-1-ol (1v)**

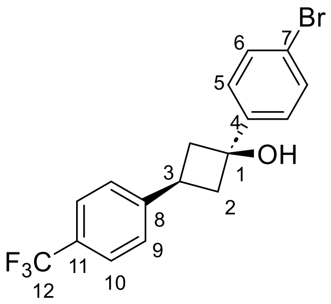

Compound **1v** was prepared following general procedure **C** using 1,4-dibromobenzene (566 mg, 2.40 mmol, 1.2 eq.) and 3-(4-(trifluoromethyl)phenyl)cyclobutan-1-one **S7** (426 mg, 2.00 mmol, 1.0 eq.). The crude product was purified by flash column chromatography (10-15% EtOAc in cyclohexane) to yield product **1v** as a yellow solid (single diastereoisomer, 352 mg, 0.95 mmol, 48%).

**R*f*** = 0.28 (15% EtOAc in cyclohexane).

**Melting Point:** 64-66 ˚C.

**$^{1}$H NMR** (500 MHz, $CDCl_3$): $\delta$ [ppm] = 7.58 (d, $^{3}J_{HH}$ = 8.1 Hz, 2H, H-C10), 7.57 – 7.54 (m, 2H, H-C6), 7.52 – 7.48 (m, 2H, H-C5), 7.39 (d, $^{3}J_{HH}$ = 8.1 Hz, 2H, H-C9), 3.11 (p, $^{3}J_{HH}$ = 9.2 Hz, 1H, H-C3), 3.08 – 3.00 (m, 2H, $H^{a}$-C2), 2.61 – 2.53 (m, 2H, $H^{b}$-C2), 2.17 (s, 1H, H-O).

**$^{19}$F NMR** (470 MHz, $CDCl_3$): $\delta$ [ppm] = -62.35 (s, 3F, F-C12).

**$^{19}$F{$^{1}$H} NMR** (470 MHz, $CDCl_3$): $\delta$ [ppm] = -62.35 (s, 3F, F-C12).

**$^{13}$C{$^{1}$H} NMR** (126 MHz, $CDCl_3$): $\delta$ [ppm] = 148.6 (q, $^{5}J_{CF}$ = 1.2 Hz, C8), 144.3 (C4), 132.0 (C6), 128.7 (q, $^{2}J_{CF}$ = 32.4 Hz, C11), 127.4 (C5), 127.1 (C9), 125.5 (q, $^{3}J_{CF}$ = 3.8 Hz, C10), 124.4 (q, $^{1}J_{CF}$ = 271.8 Hz, C12), 121.9 (C7), 72.4 (C1), 44.5 (C2), 30.0 (C3).

*Comment: HRMS-analysis was inconclusive, therefore [M]-F was reported as an indicative fragment.*

**GC-EI-MS:** Retention 11.07 min, (*m/z*) requires: [$(C_{17}H_{14}BrF_3O$ - $F)^+$] = 351.0191, (*m/z*) found: [$(C_{17}H_{14}BrF_3O$ - $F)^+$] = 351.0190.

**FT-IR** ($\tilde{\nu}$ = $cm^{-1}$)**:** 3529.9 (broad), 2980.3 (w), 2943.0 (w), 1615.7 (w), 1485.1 (w), 1433.5 (w), 1407.7 (w), 1396.2 (w), 1320.1 (s), 1284.3 (w), 1241.2 (m), 1211.1 (w), 1158.0 (s), 1117.8 (s), 1081.9 (s), 1059.0 (s), 1015.9 (s), 1007.3 (s), 974.3 (w), 948.5 (w), 888.2 (w), 840.9 (s), 815.0 (m), 803.6 (m), 736.1 (w), 721.8 (m), 716.0 (w).

**4-(3-(4-Bromophenyl)-3-hydroxycyclobutyl)phenyl trifluoromethanesulfonate (1w)**

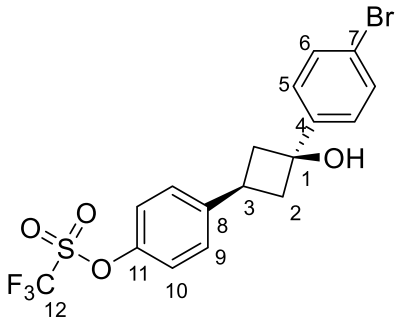


Compound **1w** was prepared following modified general procedure **C** using 1,4-dibromobenzene (566 mg, 2.40 mmol, 1.2 eq.) and 4-(3-oxocyclobutyl)phenyl trifluoromethanesulfonate **S11** (588 mg, 2.00 mmol, 1.0 eq.). The concentration of the reaction mixture was set to 0.25 M (half concentration). The crude product was purified by flash column chromatography (10-15% EtOAc in cyclohexane) to yield product **1w** as a pale-yellow oil (single diastereoisomer, 657 mg, 1.46 mmol, 73%).

**$R_f$** = 0.35 (25% EtOAc in cyclohexane).

**$^{1}$H NMR** (599 MHz, $CDCl_3$): $\delta$ [ppm] = 7.57 – 7.54 (m, 2H, H-C6), 7.51 – 7.43 (m, 2H, H-C5), 7.37 – 7.33 (m, 2H, H-C9), 7.25 – 7.20 (m, 2H, H-C10), 3.11 (p, $^{3}J_{HH}$ = 9.2 Hz, 1H, H-C3), 3.05 – 3.00 (m, 2H, $H^{a}$-C2), 2.57 – 2.50 (m, 2H, $H^{b}$-C2), 2.15 (s, 1H, H-O).

**$^{19}$F NMR** (376 MHz, $CDCl_3$): $\delta$ [ppm] = -72.84 (s, 3F, F-C12).

**$^{19}F\{^1H\}$ NMR** (376 MHz, $CDCl_3$): $\delta$ [ppm] = -72.84 (s, 3F, F-C12).

**$^{13}C\{^1H\}$ NMR** (151 MHz, $CDCl_3$): $\delta$ [ppm] = 148.1 (C11), 145.1 (C8), 144.2 (C4), 132.0 (C6), 128.6 (C9), 127.4 (C5), 122.0 (C7), 121.4 (C10), 118.9 (q, $^1J_{CF}$ = 320.8 Hz, C12), 72.3 (C1), 44.7 (C2), 29.6 (C3).

**ESI-MS:** (*m/z*) requires: [($C_{17}H_{14}BrF_3O_4SNa)^+$] = 472.9640, (*m/z*) found: [($C_{17}H_{14}BrF_3O_4SNa)^+$] = 472.9636.

**FT-IR** ($\tilde{\nu}$ = $cm^{-1}$)**:** 3352.0 (broad), 2978.9 (w), 2940.1 (w), 1592.8 (w), 1502.4 (m), 1417.7 (m), 1304.3 (w), 1248.4 (m), 1205.3 (s), 1135.0 (s), 1077.6 (m), 1015.9 (m), 1008.7 (m), 941.3 (w), 882.5 (s), 832.3 (s), 805.0 (m), 776.3 (w), 749.0 (m), 704.5 (w).

**1-(4-Bromophenyl)-3-(4-chlorophenyl)-3-methylcyclobutan-1-ol (1x)**

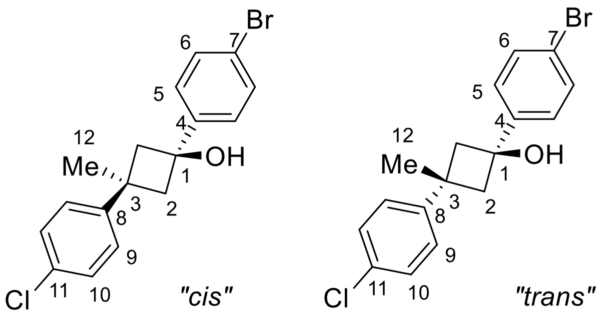


Compounds *cis*-**1x** and *trans*-**1x** were prepared following general procedure **C** using 1,4-dibromobenzene (566 mg, 2.40 mmol, 1.2 eq.) and 3-(4-chlorophenyl)-3-methylcyclobutan-1-one **S13** (396 mg, 2.00 mmol, 1.0 eq.). The crude product was purified by flash column chromatography (10-15% EtOAc in cyclohexane) to yield *cis*-**1x** as a colorless solid (single diastereoisomer, 321 mg, 0.91 mmol, 46%) and *trans*-**1x** as a colorless solid (single diastereoisomers, 323 mg, 0.92 mmol, 46%).

Comment: The diastereoisomers were assigned according to *Uemura et. al.*[11]

*cis*-**1x**

**$R_f$** = 0.48 (20% EtOAc in cyclohexane).

**Melting Point:** 119-121 ˚C.

**$^1H$ NMR** (599 MHz, $CDCl_3$): $\delta$ [ppm] = 7.55 – 7.51 (m, 2H, H-C6), 7.42 – 7.37 (m, 2H, H-C5), 7.33 – 7.29 (m, 2H, H-C10), 7.25 – 7.21 (m, 2H, H-C9), 2.90 – 2.86 (m, 2H, $H^a$-C2), 2.84 – 2.79 (m, 2H, $H^b$-C2), 1.90 (s, 1H, H-O), 1.25 (s, 3H, H-C12).

**$^{13}C\{^1H\}$ NMR** (151 MHz, $CDCl_3$): $\delta$ [ppm] = 149.9 (C4), 145.4 (C8), 131.9 (C6), 131.5 (C11), 128.6 (C10), 127.6 (C5), 126.9 (C9), 121.7 (C7), 72.1 (C1), 45.0 (C2), 34.2 (C3), 31.5 (C12).

*Comment: HRMS-analysis was inconclusive due to decomposition.*

**FT-IR** ($\tilde{\nu}$ = cm$^{-1}$)**:** 3557.2 (broad), 2971.7 (w), 2953.1 (w), 1777.9 (w), 1588.5 (w), 1482.3 (m), 1420.6 (w), 1397.6 (w), 1371.8 (w), 1297.2 (w), 1242.6 (w), 1209.6 (w), 1117.8 (w), 1106.3 (m), 1081.9 (w), 1071.9 (w), 1036.0 (m), 1008.7 (s), 938.4 (w), 895.4 (m), 823.6 (s), 784.9 (w), 734.7 (m), 724.6 (m), 718.9 (m).

*trans*-**1x**

**R*f*** = 0.38 (20% EtOAc in cyclohexane).

**Melting Point:** 112-114 ˚C.

**$^1$H NMR** (599 MHz, $CDCl_3$): $\delta$[ppm] = 7.43 – 7.39 (m, 2H, H-C6), 7.28 – 7.25 (m, 2H, H-C10), 7.20 – 7.16 (m, 2H, H-C5), 7.12 – 7.08 (m, 2H, H-C9), 2.93 – 2.88 (m, 2H, H$^a$-C2), 2.63 – 2.58 (m, 2H, H$^b$-C2), 1.98 (s, 1H, H-O), 1.68 (s, 3H, H-C12).

**$^{13}$C{$^1$H} NMR** (151 MHz, $CDCl_3$): $\delta$[ppm] = 149.9 (C4), 146.3 (C8), 131.6 (C6), 131.4 (C11), 128.6 (C10), 126.74 (C9), 126.71 (C5), 121.3 (C7), 72.5 (C1), 48.8 (C2), 35.8 (C3), 32.8 (C12).

*Comment: HRMS-analysis was inconclusive due to decomposition.*

**FT-IR** ($\tilde{\nu}$ = cm$^{-1}$)**:** 3303.2 (broad), 2974.6 (w), 1488.0 (m), 1449.3 (w), 1397.6 (w), 1301.5 (w), 1275.6 (w), 1244.1 (w), 1191.0 (w), 1166.6 (w), 1093.4 (m), 1079.1 (w), 1013.1 (m), 1005.9 (m), 952.8 (w), 939.9 (w), 896.8 (m), 850.9 (w), 828.0 (m), 823.6 (s), 816.5 (s), 731.8 (m), 718.9 (w).

**3-Phenyl-1-(4-(pyridin-3-yl)phenyl)cyclobutanol (1y)**

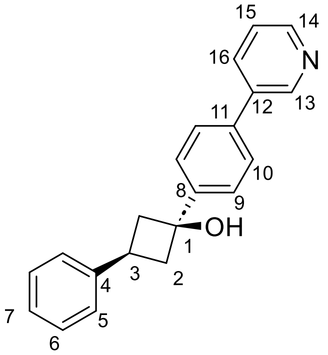


Compound **1y** was prepared following general procedure **C** using 3-(4-bromophenyl)pyridine **S14** (384 mg, 1.64 mmol, 1.2 eq.) and 3-phenylcyclobutan-1-one **S1** (200 mg, 1.37 mmol, 1.0 eq.). The crude product was purified by flash column chromatography (0-30% EtOAc in DCM) to yield product **1y** as a colorless solid (single diastereoisomer, 220 mg, 0.73 mmol, 53%).

**R*f*** = 0.22 (30% EtOAc in DCM).

**Melting Point:** 128-130 °C.

**$^1$H NMR** (500 MHz, $CDCl_3$): $\delta$ [ppm] = 8.73 (dd, $^4J_{HH}$ = 2.4 Hz, $J_{HH}$ = 0.8 Hz, 1H, H-C13), 8.58 (dd, $^3J_{HH}$ = 4.8 Hz, $^4J_{HH}$ = 1.6 Hz, 1H, H-C16), 7.89 (ddd, $^3J_{HH}$ = 7.9 Hz, $^4J_{HH}$ = 2.4 Hz, $^4J_{HH}$ = 1.6 Hz, 1H, H-C14), 7.75 (d, $^3J_{HH}$ = 8.5 Hz, 2H, H-C10), 7.62 (d, $^3J_{HH}$ = 8.5 Hz, 2H, H-C9), 7.38 (ddd, $^3J_{HH}$ = 7.9 Hz, $^3J_{HH}$ = 4.8 Hz, $J_{HH}$ = 0.8 Hz, 1H, H-C15), 7.36 – 7.31 (m, 4H, H-C5, H-C6), 7.24 – 7.21 (m, 1H, H-C7), 3.17 – 3.06 (m, 3H, H$^a$-C2, H-C3), 2.85 (s, 1H, O-H), 2.65 – 2.60 (m, 2H, H$^b$-C2).

**$^{13}C\{^{1}H\}$ NMR** (126 MHz, $CDCl_3$): $\delta$ [ppm] = 148.6 (C16), 148.4 (C13), 145.7 (C8), 144.7 (C4), 137.2 (C12), 136.4 (C11), 134.5 (C14), 128.6 (C6), 127.5 (C9), 126.8 (C5), 126.5 (C10), 126.3 (C7), 123.8 (C15), 72.3 (C1), 44.9 (C2), 30.2 (C3).

**ESI-MS:** (*m/z*) requires: $[(C_{21}H_{19}NONa)^{+}]$ = 324.1358, (*m/z*) found: $[(C_{21}H_{19}NONa)^{+}]$ = 324.1359.

**FT-IR** ($\tilde{\nu}$ = $cm^{-1}$)**:** 3320.39 (broad), 2980.32 (broad), 1493.75 (w), 1475.09 (m), 1427.74 (m), 1389.00 (m), 1269.90 (m), 1238.33 (m), 1160.85 (w), 1094.84 (m), 1057.53 (m), 1028.83 (m), 1007.31 (m), 901.13 (w), 876.73 (w), 842.29 (s), 797.81 (s), 756.20 (s), 747.59 (s), 710.28 (s), 695.93 (s), 629.93 (m).

## Synthesis of (1,3,3-Trifluorobutane-1,4-diyl)dibenzene Derivatives

### General Procedure D

Cyclobutanol derivative **1** (0.20 mmol, 1.0 eq.) and *p*-TolI (8.7 mg, 0.04 mmol, 20 mol%) were dissolved in $CHCl_3$ (0.5 mL) in a Teflon®-vial (5 mL total volume). Subsequently, $NEt_3$•3HF and Olah's reagent were added with the appropriate ratio (0.5 mL total volume, for more information, see below). Finally, Selectfluor® (106 mg, 0.3 mmol, 1.5 eq.) was added to the reaction mixture in one portion. The reaction mixture was stirred for 18 h at room temperature. The reaction mixture was diluted with DCM (2 mL) and poured in saturated aqueous $NaHCO_3$ (100 mL). The aqueous layer was extracted with DCM (3x 30 mL). The combined organic layers were dried over $Na_2SO_4$ and the solvent was removed under reduced pressure. The crude product was purified by flash column chromatography.

The amine:HF mixtures are prepared in accordance to a previous publication of this group.[7]

**1:4.5:** 0.34 mL $NEt_3$•3HF and 0.16 mL Olah's reagent

**1:5.0:** 0.29 mL $NEt_3$•3HF and 0.21 mL Olah's reagent

**1:5.5:** 0.25 mL $NEt_3$•3HF and 0.25 mL Olah's reagent

**1:6.5:** 0.17 mL $NEt_3$•3HF and 0.33 mL Olah's reagent

***Caution:*** *Olah's reagent is highly toxic and corrosive. Direct exposure should be avoided. In the case of skin exposure, immediate treatment of the affected skin area with calcium gluconate gel is necessary to prevent serious chemical burns.*

**(1,3,3-Trifluorobutane-1,4-diyl)dibenzene (2a)**

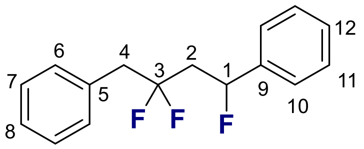


Compound **2a** was prepared according to general procedure **D** (amine:HF ratio = 1:4.5) using 1,3-diphenylcyclobutan-1-ol **1a** (44.9 mg, 0.200 mmol). The crude product was purified by flash column chromatography (5-10% DCM in cyclohexane) to yield the title compound as a colorless solid (30.0 mg, 0.114 mmol, 57%).

**R*f*** = 0.20 (10% DCM in cyclohexane).

**Melting Point:** 42-44 ˚C.

**$^1$H NMR** (500 MHz, $CDCl_3$): $\delta$ [ppm] = 7.43 – 7.28 (m, 10H, H-C6, H-C7, H-C8, H-C10, H-C11, H-C12), 5.81 (ddd, $^2J_{HF}$ = 48.0 Hz, $^3J_{HH}$ = 9.9 Hz, $^3J_{HH}$ = 2.5 Hz, 1H, H-C1), 3.38 – 3.21 (m, 2H, H-C4), 2.55 (dtdd, $^3J_{HF}$ = 18.5 Hz $^2J_{HH}$ = $^3J_{HF}$ = 15.6 Hz, $^3J_{HF}$ = 13.8 Hz, $^3J_{HH}$ = 9.5 Hz, 1H, $H^a$-C2), 2.23 (dtdd, $^3J_{HF}$ = 34.7 Hz, $^2J_{HH}$ = $^3J_{HF}$ = 16.0 Hz, $^3J_{HF}$ = 12.0 Hz, $^3J_{HH}$ = 2.6 Hz, 1H, $H^b$-C2).

**$^{19}$F NMR** (470 MHz, $CDCl_3$): $\delta$ [ppm] = -92.70 (dttd, $^2J_{FF}$ = 247.3 Hz, $^3J_{FH}$ = 18.3 Hz, $^3J_{FH}$ = 13.3 Hz, $^4J_{FF}$ = 4.5 Hz, 1F, $F^a$-C3), -95.89 (dpd, $^2J_{FF}$ = 247.3 Hz, $^3J_{FH}$ = 15.8 Hz, $^4J_{FF}$ = 8.1 Hz, 1F, $F^b$-C3), -173.75 (ddddd, $^2J_{FH}$ = 48.2 Hz, $^3J_{FH}$ = 34.9 Hz, $^3J_{FH}$ = 15.1 Hz, $^4J_{FF}$ = 7.7 Hz, $^4J_{FF}$ = 4.5 Hz, 1F, F-C1).

**$^{19}$F{$^1$H} NMR** (470 MHz, $CDCl_3$): $\delta$ [ppm] = -92.70 (dd, $^2J_{FF}$ = 247.3 Hz, $^4J_{FF}$ = 4.3 Hz, 1F, $F^a$-C3), -95.89 (dd, $^2J_{FF}$ = 247.2 Hz, $^4J_{FF}$ = 8.1 Hz, 1F, $F^b$-C3), -173.75 (dd, $^4J_{FF}$ = 8.1 Hz, $^4J_{FF}$ = 4.4 Hz, 1F, F-C1).

**$^{13}$C{$^1$H} NMR** (126 MHz, $CDCl_3$): $\delta$ [ppm] = 139.4 (d, $^2J_{CF}$ = 19.8 Hz, C9), 133.1 (dd, $^3J_{CF}$ = 6.5 Hz, $^3J_{CF}$ = 3.1 Hz, C5), 130.7 (C6), 128.9 (d, $^5J_{CF}$ = 2.0 Hz, C12), 128.8 (C7 or C11), 128.6 (C7 or C11), 127.6 (C8), 125.6 (d, $^3J_{CF}$ = 6.7 Hz, C10), 122.7 (dd, $^1J_{CF}$ = 243.9 Hz, $^1J_{CF}$ = 242.9 Hz, C3), 89.6 (ddd, $^1J_{CF}$ = 171.9 Hz, $^3J_{CF}$ = 7.8 Hz, $^3J_{CF}$ = 3.8 Hz, C1), 43.7 – 43.0 (C2 and C4).

**GC-EI-MS:** Retention 9.06 min, (*m/z*) requires: $[(C_{16}H_{15}F_3)^+]$ = 264.1120, (*m/z*) found: $[(C_{16}H_{15}F_3)^+]$ = 264.1121.

**FT-IR** ($\tilde{\nu}$ = $cm^{-1}$)**:** 3065.0 (w), 3034.8 (w), 1604.2 (w), 1495.2 (m), 1457.9 (m), 1437.8 (w), 1424.9 (w), 1401.9 (m), 1361.7 (m), 1343.1 (w), 1330.2 (w), 1287.1 (m), 1269.9 (w), 1211.1 (m), 1182.4 (w), 1158.0 (m), 1136.5 (m), 1106.3 (w), 1087.7 (m), 1059.0 (m), 1027.4 (s), 1001.6 (m), 955.7 (m), 915.5 (m), 894.0 (m), 859.5 (m), 843.7 (m), 817.9 (m), 759.1 (s), 734.7 (s).

**4,4'-(1,3,3-Trifluorobutane-1,4-diyl)bis(fluorobenzene) (2b)**

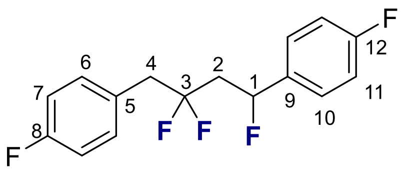


Compound **2b** was prepared according to general procedure **D** (amine:HF ratio = 1:4.5) using 1,3-bis(4-fluorophenyl)-cyclobutan-1-ol **1b** (52.1 mg, 0.200 mmol). The crude product was purified by flash column chromatography (0-5% DCM in *n*-pentane) to yield the title compound as a colorless solid (30.0 mg, 0.100 mmol, 50%).

$\mathbf{R}_f$ = 0.30 (5% DCM in *n*-pentane).

**Melting Point:** 54-56 ˚C.

**$^{1}$H NMR** (599 MHz, $CDCl_3$): $\delta$ [ppm] = 7.31 – 7.25 (m, 4H, H-C6, H-C10), 7.07 (t, $^{3}J_{HH}$ = $^{3}J_{HF}$ = 8.3 Hz, 1H, H-C11), 7.03 (t, $^{3}J_{HH}$ = $^{3}J_{HF}$ = 8.7 Hz, 2H, H-C7), 5.77 (ddd, $^{2}J_{HF}$ = 47.7 Hz, $^{3}J_{HH}$ = 9.3 Hz, $^{3}J_{HH}$ = 2.8 Hz, 1H, H-C1), 3.32 – 3.18 (m, 2H, H-C4), 2.51 (dtdd, $^{3}J_{HF}$ = 19.2 Hz $^{2}J_{HH}$ = $^{3}J_{HF}$ = 15.5 Hz, $^{3}J_{HF}$ = 13.3 Hz, $^{3}J_{HH}$ = 9.4 Hz, 1H, $H^{a}$-C2), 2.20 (dtdd, $^{3}J_{HF}$ = 34.3 Hz, $^{2}J_{HH}$ = $^{3}J_{HF}$ = 15.4 Hz, $^{3}J_{HF}$ = 11.6 Hz, $^{3}J_{HH}$ = 2.8 Hz, 1H, $H^{b}$-C2).

**$^{19}$F NMR** (564 MHz, $CDCl_3$): $\delta$ [ppm] = -92.98 (ddtdd, $^{2}J_{FF}$ = 248.1 Hz, $^{3}J_{FH}$ = 18.4 Hz, $^{3}J_{FH}$ = 15.8 Hz, $^{3}J_{FH}$ = 12.5 Hz, $^{4}J_{FF}$ = 3.9 Hz, 1F, $F^{a}$-C3), -96.66 (ddtdd, $^{2}J_{FF}$ = 248.9 Hz, $^{3}J_{FH}$ = 19.6 Hz, $^{3}J_{FH}$ = 15.7 Hz, $^{3}J_{FH}$ = 12.8 Hz , $^{4}J_{FF}$ = 7.4 Hz, 1F, $F^{b}$-C3), -112.70 (ttd, $^{3}J_{FH}$ = 8.6 Hz, $^{4}J_{FH}$ = 5.2 Hz, $^{6}J_{FF}$ = 3.4 Hz, 1F, F-C12), -115.18 (tt, $^{3}J_{FH}$ = 8.7 Hz, $^{4}J_{FH}$ = 5.4 Hz, F-C8), -171.39 – -171.63 (m, 1F, F-C1).

**$^{19}$F{$^{1}$H} NMR** (564 MHz, $CDCl_3$): $\delta$ [ppm] = -92.96 (dd, $^{2}J_{FF}$ = 248.1 Hz, $^{4}J_{FF}$ = 4.1 Hz, 1F, $F^{a}$-C3), -96.58 (dd, $^{2}J_{FF}$ = 248.1 Hz, $^{4}J_{FF}$ = 7.3 Hz, 1F, $F^{b}$-C3), -112.70 (d, $^{6}J_{FF}$ = 3.4 Hz, 1F, F-C12), -115.15 (s, F-C8), -171.47 (dt, $^{4}J_{FF}$ = 7.5 Hz, $^{4}J_{FF}$ = $^{6}J_{FF}$ = 3.8 Hz, 1F, F-C1).

**$^{13}$C{$^{1}$H} NMR** (151 MHz, $CDCl_3$): $\delta$ [ppm] = 163.0 (dd, $^{1}J_{CF}$ = 247.7 Hz, $^{5}J_{CF}$ = 2.4 Hz, C12), 162.5 (d, $^{1}J_{CF}$ = 246.0 Hz, C8), 135.0 (dd, $^{2}J_{CF}$ = 20.4 Hz, $^{4}J_{CF}$ = 3.2 Hz, C9), 132.3 (d, $^{3}J_{CF}$ = 8.0 Hz, C6), 128.7 (dd, $^{3}J_{CF}$ = 6.6 Hz, $^{3}J_{CF}$ = 3.2 Hz, C5), 127.6 (dd, $^{3}J_{CF}$ = 8.3 Hz, $^{3}J_{CF}$ = 6.5 Hz, C10), 124.4 (ddd, $^{1}J_{CF}$ = 244.1 Hz, $^{1}J_{CF}$ = 242.7 Hz, $^{3}J_{CF}$ = 1.7 Hz, C3), 115.9 (d, $^{2}J_{CF}$ = 21.8 Hz, C11), 115.5 (d, $^{2}J_{CF}$ = 21.4 Hz, C7), 89.0 (ddd, $^{1}J_{CF}$ = 172.0 Hz, $^{3}J_{CF}$ = 8.1 Hz, $^{3}J_{CF}$ = 3.8 Hz, C1), 43.3 (q, $^{2}J_{CF}$ = 24.8 Hz, C2), 42.6 (td, $^{2}J_{CF}$ = 25.1 Hz, $^{4}J_{CF}$ = 2.9 Hz, C4).

**GC-EI-MS:** Retention 8.97 min, (*m/z*) requires: $[(C_{16}H_{13}F_5)^+]$ = 300.0932, (*m/z*) found: $[(C_{16}H_{13}F_5)^+]$ = 300.0932.

**FT-IR** ($\tilde{\nu}$ = $cm^{-1}$)**:** 1608.5 (m), 1511.0 (s), 1433.5 (w), 1386.1 (w), 1224.0 (s), 1179.5 (w), 1158.0 (m), 1114.9 (m), 1096.3 (m), 1070.5 (m), 1038.9 (m), 1014.5 (w), 889.7 (w), 869.6 (w), 836.6 (s), 812.2 (s), 782.0 (m), 772.0 (s), 721.8 (w).

**4,4'-(1,3,3-Trifluorobutane-1,4-diyl)bis(chlorobenzene) (2c)**

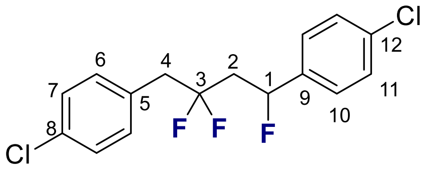


Compound **2c** was prepared according to general procedure **D** (amine:HF ratio = 1:5.5) using 1,3-bis(4-chlorophenyl)-cyclobutan-1-ol ***major*-1c** (58.6 mg, 0.200 mmol). The crude product was purified by flash column chromatography (5-10% DCM in cyclohexane) to yield the title compound as a colorless solid (49.2 mg, 0.148 mmol, 74%).

**$R_f$** = 0.22 (10% DCM in cyclohexane).

**Melting Point:** 73-75 ˚C.

**$^{1}$H NMR** (500 MHz, $CDCl_3$): $\delta$[ppm] = 7.38 – 7.35 (m, 2H, H-C10), 7.34 – 7.30 (m, 2H, H-C6), 7.27 – 7.22 (m, 4H, H-C7, H-C11), 5.78 (ddd, $^{2}J_{HF}$ = 47.9 Hz, $^{3}J_{HH}$ = 9.4 Hz, $^{3}J_{HH}$ = 2.7 Hz, 1H, H-C1), 3.35 – 3.16 (m, 2H, H-C4), 2.51 (dtdd, $^{3}J_{HF}$ = 19.1 Hz $^{2}J_{HH}$ = $^{3}J_{HF}$ = 15.6 Hz, $^{3}J_{HF}$ = 13.3 Hz, $^{3}J_{HH}$ = 9.5 Hz, 1H, $H^{a}$-C2), 2.21 (dtdd, $^{3}J_{HF}$ = 34.5 Hz, $^{2}J_{HH}$ = $^{3}J_{HF}$ = 15.7 Hz, $^{3}J_{HF}$ = 11.4 Hz, $^{3}J_{HH}$ = 2.7 Hz, 1H, $H^{b}$-C2).

**$^{19}$F NMR** (376 MHz, $CDCl_3$): $\delta$ [ppm] = -92.75 (ddtdd, $^{2}J_{FF}$ = 248.5 Hz, $^{3}J_{FH}$ = 18.0 Hz, $^{3}J_{FH}$ = 15.4 Hz $^{3}J_{FH}$ = 12.7 Hz, $^{4}J_{FF}$ = 3.9 Hz, 1F, $F^{a}$-C3), -96.44 (ddtdd, $^{2}J_{FF}$ = 248.2 Hz, $^{3}J_{FH}$ = 19.6 Hz, $^{3}J_{FH}$ = 15.8 Hz, $^{3}J_{FH}$ = 12.6 Hz, $^{4}J_{FF}$ = 7.4 Hz, 1F, $F^{b}$-C3), -173.86 (ddddd, $^{2}J_{FH}$ = 47.9 Hz, $^{3}J_{FH}$ = 34.6 Hz, $^{3}J_{FH}$ = 15.6 Hz, $^{4}J_{FF}$ = 7.2 Hz, $^{4}J_{FF}$ = 4.1 Hz, 1F, F-C1).

**$^{19}$F{$^{1}$H} NMR** (376 MHz, $CDCl_3$): $\delta$[ppm] = -92.74 (dd, $^{2}J_{FF}$ = 248.3 Hz, $^{4}J_{FF}$ = 3.9 Hz, 1F, $F^{a}$-C3), -96.43 (dd, $^{2}J_{FF}$ = 248.2 Hz, $^{4}J_{FF}$ = 7.4 Hz, 1F, $F^{b}$-C3), -173.86 (dd, $^{4}J_{FF}$ = 7.5 Hz, $^{4}J_{FF}$ = 3.9 Hz, 1F, F-C1).

**$^{13}$C{$^{1}$H} NMR** (126 MHz, $CDCl_3$): $\delta$[ppm] = 137.6 (d, $^{2}J_{CF}$ = 20.3 Hz, C9), 125.0 (d, $^{5}J_{CF}$ = 2.4 Hz, C12), 132.7 (C8), 132.1 (overlying signals, C7, C11), 131.4 (dd, $^{3}J_{CF}$ = 6.5 Hz, $^{3}J_{CF}$ = 2.7 Hz, C5), 128.8 (C6), 127.0 (d, $^{3}J_{CF}$ = 6.7 Hz, C10), 122.3 (dd, $^{1}J_{CF}$ = 244.4 Hz, $^{1}J_{CF}$ = 242.9 Hz, C3), 88.9 (ddd, $^{1}J_{CF}$ = 172.8 Hz, $^{3}J_{CF}$ = 8.1 Hz, $^{3}J_{CF}$ = 3.8 Hz, C1), 43.3 (dt, $^{2}J_{CF}$ = 26.1 Hz, $^{2}J_{CF}$ = 24.2 Hz, C2), 42.7 (ddd, $^{2}J_{CF}$ = 26.3 Hz, $^{2}J_{CF}$ = 24.7 Hz, $^{4}J_{CF}$ = 2.7 Hz, C4).

**GC-EI-MS:** Retention 10.66 min, (*m/z*) requires: [$(C_{16}H_{13}Cl_2F_3)^{+}$] = 332.0341, (*m/z*) found: [$(C_{16}H_{13}Cl_2F_3)^{+}$] = 332.0341.

**FT-IR** ($\tilde{\nu}$ = $cm^{-1}$)**:** 2988.9 (w), 1595.6 (w), 1492.3 (m), 1409.1 (w), 1376.1 (w), 1343.1 (w), 1315.8 (w), 1294.3 (w), 1261.3 (w), 1218.2 (w), 1208.2 (w), 1156.5 (s), 1140.8 (m), 1103.5 (m), 1064.7 (s), 1028.8 (m), 1014.5 (s), 975.7 (m), 896.8 (m), 869.6 (m), 853.8 (m), 838.0 (s), 823.6 (s), 783.5 (s), 733.2 (m), 716.0 (w).

**4,4'-(1,3,3-Trifluorobutane-1,4-diyl)bis(bromobenzene) (2d)**

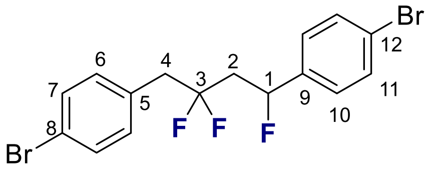


Compound **2d** was prepared according to general procedure **D** (amine:HF ratio = 1:5.5) using 1,3-bis(4-bromophenyl)-cyclobutan-1-ol **1d** (76.4 mg, 0.200 mmol). The crude product was purified by flash column chromatography (5-10% DCM in cyclohexane) to yield the title compound as a colorless solid (71.0 mg, 0.168 mmol, 84%).

$\mathbf{R}_f$ = 0.23 (10% DCM in cyclohexane).

**Melting Point:** 98-100 ˚C.

**$^{1}$H NMR** (500 MHz, $CDCl_3$): $\delta$[ppm] = 7.54 – 7.50 (m, 2H, H-C10), 7.49 – 7.44 (m, 2H, H-C6), 7.21 – 7.15 (m, 4H, H-C7, H-C11), 5.75 (ddd, $^{2}J_{HF}$ = 47.9 Hz, $^{3}J_{HH}$ = 9.4 Hz, $^{3}J_{HH}$ = 2.6 Hz, 1H, H-C1), 3.32 – 3.15 (m, 2H, H-C4), 2.48 (dtdd, $^{3}J_{HF}$ = 19.1 Hz $^{2}J_{HH}$ = $^{3}J_{HF}$ = 15.7 Hz, $^{3}J_{HF}$ = 13.4 Hz, $^{3}J_{HH}$ = 9.5 Hz, 1H, $H^{a}$-C2), 2.19 (dtdd, $^{3}J_{HF}$ = 34.5 Hz, $^{2}J_{HH}$ = $^{3}J_{HF}$ = 15.7 Hz, $^{3}J_{HF}$ = 11.3 Hz, $^{3}J_{HH}$ = 2.7 Hz, 1H, $H^{b}$-C2).

**$^{19}$F NMR** (376 MHz, $CDCl_3$): $\delta$ [ppm] = -92.72 (ddtdd, $^{2}J_{FF}$ = 249.2 Hz, $^{3}J_{FH}$ = 18.7 Hz, $^{3}J_{FH}$ = 16.0 Hz, $^{3}J_{FH}$ = 12.8 Hz, $^{4}J_{FF}$ = 4.0 Hz, 1F, $F^{a}$-C3), -96.42 (ddtdd, $^{2}J_{FF}$ = 248.3 Hz, $^{3}J_{FH}$ = 19.7 Hz, $^{3}J_{FH}$ = 15.7 Hz, $^{3}J_{FH}$ = 12.6 Hz, $^{4}J_{FF}$ = 7.5 Hz, 1F, $F^{b}$-C3), -174.36 (ddddd, $^{2}J_{FH}$ = 47.8 Hz, $^{3}J_{FH}$ = 34.6 Hz, $^{3}J_{FH}$ = 15.6 Hz, $^{4}J_{FF}$ = 7.4 Hz, $^{4}J_{FF}$ = 3.8 Hz, 1F, F-C1).

**$^{19}$F{$^{1}$H} NMR** (376 MHz, $CDCl_3$): $\delta$[ppm] = -92.71 (dd, $^{2}J_{FF}$ = 248.3 Hz, $^{4}J_{FF}$ = 3.9 Hz, 1F, $F^{a}$-C3), -96.41 (dd, $^{2}J_{FF}$ = 248.4 Hz, $^{4}J_{FF}$ = 7.5 Hz, 1F, $F^{b}$-C3), -174.36 (dd, $^{4}J_{FF}$ = 7.6 Hz, $^{4}J_{FF}$ = 3.8 Hz, 1F, F-C1).

**$^{13}$C{$^{1}$H} NMR** (126 MHz, $CDCl_3$): $\delta$[ppm] = 138.1 (d, $^{2}J_{CF}$ = 20.3 Hz, C9), 132.2 (C7), 132.1 (C11), 131.9 (dd, $^{3}J_{CF}$ = 6.6 Hz, $^{3}J_{CF}$ = 2.4 Hz, C5), 131.8 (C6), 127.2 (d, $^{3}J_{CF}$ = 6.7 Hz, C10), 123.0 (d, $^{5}J_{CF}$ = 2.5 Hz, C12), 122.2 (dd, $^{1}J_{CF}$ = 244.4 Hz, $^{1}J_{CF}$ = 243.0 Hz, C3), 121.9 (C8), 89.9 (ddd, $^{1}J_{CF}$ = 172.9 Hz, $^{3}J_{CF}$ = 8.1 Hz, $^{3}J_{CF}$ = 3.7 Hz, C1), 43.3 (dt, $^{2}J_{CF}$ = 26.1 Hz, $^{2}J_{CF}$ = 24.1 Hz, C2), 42.8 (ddd, $^{2}J_{CF}$ = 26.3 Hz, $^{2}J_{CF}$ = 24.7 Hz, $^{4}J_{CF}$ = 2.7 Hz, C4).

**GC-EI-MS:** Retention 11.35 min, (*m/z*) requires: $[(C_{16}H_{13}Br_2F_3)^+]$ = 421.9311, (*m/z*) found: $[(C_{16}H_{13}Br_2F_3)^+]$ = 421.9309.

**FT-IR** ($\tilde{\nu}$ = cm$^{-1}$)**:** 2970.3 (w), 2917.2 (w), 1592.8 (w), 1488.0 (m), 1406.2 (w), 1377.5 (w), 1343.1 (w), 1314.4 (w), 1294.3 (w), 1218.2 (w), 1155.1 (m), 1140.8 (m), 1106.3 (w), 1066.1 (s), 1030.3 (m), 1010.2 (s), 975.7 (w), 896.8 (w), 869.6 (w), 852.3 (m), 836.6 (m), 820.8 (m), 780.6 (s), 726.1 (m).

**4,4'-(1,3,3-Trifluorobutane-1,4-diyl)bis((trifluoromethyl)benzene) (2e)**

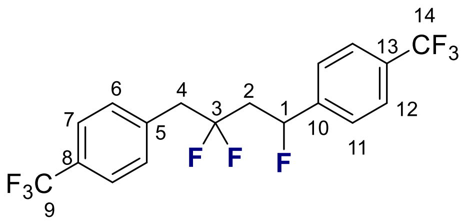


Compound **2e** was prepared according to modified general procedure **D** (amine:HF ratio = 1:6.5) using 1,3-bis(4-(trifluoromethyl)phenyl)cyclobutan-1-ol **1e** (72.1 mg, 0.200 mmol). The reaction was stirred for 42 h. The crude product was purified by flash column chromatography (5-10% DCM in cyclohexane) to yield the title compound as a colorless solid (53.4 mg, 0.133 mmol, 67%).

**R*f*** = 0.27 (10% DCM in cyclohexane)

**Melting Point:** 76-78 ˚C.

**$^{1}$H NMR** (500 MHz, $CDCl_3$): $\delta$ [ppm] = 7.66 (d, $^{3}J_{HH}$ = 8.1 Hz, 2H, H-C12), 7.61 (d, $^{3}J_{HH}$ = 8.1 Hz, 2H, H-C7), 7.48 – 7.41 (m, 4H, H-C6, H-C11), 5.88 (ddd, $^{2}J_{HF}$ = 48.0 Hz, $^{3}J_{HH}$ = 9.5 Hz, $^{3}J_{HH}$ = 2.5 Hz, 1H, H-C1), 3.49 – 3.25 (m, 2H, H-C4), 2.51 (dtdd, $^{3}J_{HF}$ = 19.2 Hz, $^{2}J_{HH}$ = $^{3}J_{HF}$ = 15.9 Hz, $^{3}J_{HF}$ = 13.2 Hz, $^{3}J_{HH}$ = 9.5 Hz, 1H, H$^{a}$-C2), 2.26 (dtdd, $^{3}J_{HF}$ = 34.6 Hz, $^{2}J_{HH}$ = $^{3}J_{HF}$ = 15.8 Hz, $^{3}J_{HF}$ = 11.1 Hz, $^{3}J_{HH}$ = 2.6 Hz, 1H, H$^{b}$-C2).

**$^{19}$F NMR** (376 MHz, $CDCl_3$): $\delta$ [ppm] = -62.65 (s, 3F, F-C9), -62.78 (s, 3F, F-C14), -92.53 (ddtdd, $^{2}J_{FF}$ = 249.2 Hz, $^{3}J_{FH}$ = 18.3 Hz, $^{3}J_{FH}$ = 15.0 Hz, $^{3}J_{FH}$ = 13.8 Hz, $^{4}J_{FF}$ = 3.8 Hz, 1F, F$^{a}$-C3), -96.45 (ddtdd, $^{2}J_{FF}$ = 249.2 Hz, $^{3}J_{FH}$ = 18.6 Hz, $^{3}J_{FH}$ = 15.5 Hz, $^{3}J_{FH}$ = 11.9 Hz, $^{4}J_{FF}$ = 7.6 Hz, 1F, F$^{b}$-C3), -174.24 (ddddd, $^{2}J_{FH}$ = 48.1 Hz, $^{3}J_{FH}$ = 34.4 Hz, $^{3}J_{FH}$ = 15.9 Hz, $^{4}J_{FF}$ = 6.6 Hz, $^{4}J_{FF}$ = 3.3 Hz, 1F, F-C1).

**$^{19}$F{$^{1}$H} NMR** (376 MHz, $CDCl_3$): $\delta$ [ppm] = -62.65 (s, 3F, F-C9), -62.79 (d, $^{7}J_{FF}$ = 1.1 Hz, 3F, F-C14), -92.53 (dd, $^{2}J_{FF}$ = 249.2 Hz, $^{4}J_{FF}$ = 3.8 Hz, 1F, F$^{a}$-C3), -96.45 (dd, $^{2}J_{FF}$ = 249.2 Hz, $^{4}J_{FF}$ = 7.5 Hz, 1F, F$^{b}$-C3), -177.24 (dd, $^{4}J_{FF}$ = 7.5 Hz, $^{4}J_{FF}$ = 3.9 Hz, 1F, F-C1).

**$^{13}$C{$^{1}$H} NMR** (126 MHz, $CDCl_3$): $\delta$ [ppm] = 143.0 (d, $^{2}J_{CF}$ = 20.3 Hz, C10), 136.8 (d, $^{3}J_{CF}$ = 6.2 Hz, C5), 131.2 (qd, $^{2}J_{CF}$ = 32.8 Hz, $^{5}J_{CF}$ = 1.6 Hz, C13), 131.1 (C6), 130.1 (q, $^{2}J_{CF}$ = 32.6 Hz, C8), 125.9 (q, $^{3}J_{CF}$ = 3.8 Hz, C12), 125.7 (d, $^{3}J_{CF}$ = 7.3 Hz, C11), 125.6 (q, $^{3}J_{CF}$ = 3.8 Hz, C7), 124.2 (q, $^{1}J_{CF}$ = 272.0 Hz, C9), 124.0 (q, $^{1}J_{CF}$ = 272.1 Hz, C14), 122.2 (dd, $^{1}J_{CF}$ = 244.3 Hz, $^{1}J_{CF}$ = 243.8 Hz, C3), 88.8 (ddd, $^{1}J_{CF}$ = 174.1 Hz, $^{3}J_{CF}$ = 8.3 Hz, $^{3}J_{CF}$ = 3.7 Hz, C1), 43.6 (dt, $^{2}J_{CF}$ = 26.3 Hz, $^{2}J_{CF}$ = 24.1 Hz, C2), 43.2 (ddd, $^{2}J_{CF}$ = 27.0 Hz, $^{2}J_{CF}$ = 25.2 Hz, $^{4}J_{CF}$ = 2.6 Hz, C4).

**GC-EI-MS:** Retention 8.85 min, (*m/z*) requires: [$(C_{18}H_{13}F_9)^+$] = 400.0868, (*m/z*) found: [$(C_{18}H_{13}F_9)^+$] = 400.0879.

**FT-IR** ($\tilde{\nu}$ = cm$^{-1}$)**:** 1621.5 (w), 1417.7 (w), 1383.3 (w), 1320.1 (m), 1170.9 (m), 1155.1 (m), 1123.5 (s), 1110.6 (s), 1064.7 (s), 1041.7 (m), 1017.4 (m), 981.5 (w), 961.4 (w), 901.1 (w), 863.8 (m), 849.5 (m), 835.1 (m), 793.5 (m), 772.0 (w), 759.1 (w), 743.3 (w), 730.4 (w).

**1-Fluoro-4-(2,2,4-trifluoro-4-phenylbutyl)benzene (2f)**

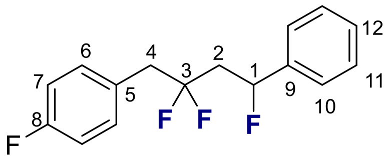


Compound **2f** was prepared according to general procedure **D** (amine:HF ratio = 1:4.5) using 1-(4-fluorophenyl)-3-phenylcyclobutan-1-ol **1f** (48.5 mg, 0.200 mmol). The crude product was purified by flash column chromatography (5-10% DCM in cyclohexane) to yield the title compound as a colorless solid (31.7 mg, 0.111 mmol, 56%).

**R*f*** = 0.29 (10% DCM in cyclohexane).

**Melting Point:** 59-61 ˚C.

**$^1$H NMR** (599 MHz, CDCl$_3$): $\delta$ [ppm] = 7.42 – 7.34 (m, 3H, H-C11, H-C12), 7.34 – 7.31 (m, 2H, H-C10), 7.31 – 7.27 (m, 2H, H-C6), 7.06 – 7.01 (m, 2H, H-C7), 5.81 (ddd, $^2J_{HF}$ = 48.2 Hz, $^3J_{HH}$ = 9.7 Hz, $^3J_{HH}$ = 2.5 Hz, 1H, H-C1), 3.34 – 3.21 (m, 2H, H-C4), 2.53 (dtdd, $^3J_{HF}$ = 19.9 Hz, $^2J_{HH}$ = $^3J_{HF}$ = 15.5 Hz, $^3J_{HF}$ = 12.5 Hz, $^3J_{HH}$ = 9.6 Hz, 1H, H$^a$-C2), 2.23 (dtdd, $^3J_{HF}$ = 35.5 Hz, $^2J_{HH}$ = $^3J_{HF}$ = 15.3 Hz, $^3J_{HF}$ = 11.9 Hz, $^3J_{HH}$ = 2.5 Hz, 1H, H$^b$-C2).

**$^{19}$F NMR** (564 MHz, CDCl$_3$): $\delta$ [ppm] = -92.87 (ddtdd, $^2J_{FF}$ = 247.9 Hz, $^3J_{FH}$ = 19.5 Hz, $^3J_{FH}$ = 15.7 Hz, $^3J_{FH}$ = 12.3 Hz, $^4J_{FF}$ = 4.1 Hz, 1F, F$^a$-C3), -96.51 (ddtdd, $^2J_{FF}$ = 247.9 Hz, $^3J_{FH}$ = 20.1 Hz, $^3J_{FH}$ = 15.4 Hz, $^3J_{FH}$ = 12.8 Hz, $^4J_{FF}$ = 7.5 Hz, 1F, F$^b$-C3), -115.28 (tt, $^3J_{FH}$ = 8.6 Hz, $^4J_{FH}$ = 5.3 Hz, 1F, F-C8), -173.74 (ddddd, $^2J_{FH}$ = 48.5 Hz, $^3J_{FH}$ = 35.4 Hz, $^3J_{FH}$ = 15.4 Hz, $^4J_{FF}$ = 7.0 Hz, $^4J_{FF}$ = 3.9 Hz, 1F, F-C1).

**$^{19}$F{$^1$H} NMR** (564 MHz, CDCl$_3$): $\delta$ [ppm] = -92.87 (dd, $^2J_{FF}$ = 247.9 Hz, $^4J_{FF}$ = 4.1 Hz, 1F, F$^a$-C3), -96.51 (dd, $^2J_{FF}$ = 247.9 Hz, $^4J_{FF}$ = 7.5 Hz, 1F, F$^b$-C3), -115.28 (s, 1F, F-C8), -173.74 (dd, $^4J_{FF}$ = 7.6 Hz, $^4J_{FF}$ = 4.1 Hz, 1F, F-C1).

**$^{13}$C{$^1$H} NMR** (151 MHz, CDCl$_3$): $\delta$ [ppm] = 162.4 (d, $^1J_{CF}$ = 245.9 Hz, C8), 139.2 (d, $^2J_{CF}$ = 19.9 Hz, C9), 132.3 (d, $^3J_{CF}$ = 8.0 Hz, C6), 129.0 (d, $^5J_{CF}$ = 2.1 Hz, C12), 128.9 (C11), 128.8 (dt, $^3J_{CF}$ = 6.4 Hz, $^3J_{CF}$ = $^4J_{CF}$ = 2.9 Hz, C5), 125.6 (d, $^3J_{CF}$ = 6.7 Hz, C10), 122.5 (ddd, $^1J_{CF}$ = 244.2 Hz, $^1J_{CF}$ = 242.5 Hz, $^3J_{CF}$ = 1.4 Hz, C3), 115.5 (d, $^2J_{CF}$ = 21.3 Hz, C7), 89.6 (ddd, $^1J_{CF}$ = 171.9 Hz, $^3J_{CF}$ = 8.3 Hz, $^3J_{CF}$ = 3.7 Hz, C1), 43.3 (dt, $^2J_{CF}$ = 26.2 Hz, $^2J_{CF}$ = 24.3 Hz, C2), 42.5 (ddd, $^2J_{CF}$ = 26.8 Hz, $^2J_{CF}$ = 24.8 Hz, $^4J_{CF}$ = 2.8 Hz, C4).

**GC-EI-MS:** Retention 8.39 min, (*m/z*) requires: [$(C_{16}H_{14}F_4)^+$] = 282.1026, (*m/z*) found: [$(C_{16}H_{14}F_4)^+$] = 282.1025.

**FT-IR** ($\tilde{\nu}$ = cm$^{-1}$)**:** 1608.5 (w), 1509.5 (m), 1457.9 (w), 1439.2 (w), 1404.8 (w), 1363.2 (w), 1304.3 (w), 1287.1 (w), 1269.9 (w), 1222.6 (m), 1211.1 (m), 1156.5 (m), 1135.0 (m), 1097.7 (m), 1084.8 (m), 1059.0 (m), 1030.3 (s), 1014.5 (m), 1001.6 (m), 955.7 (m), 914.0 (w), 895.4 (m), 859.5 (m), 840.9 (m), 829.4 (m), 793.5 (m), 774.9 (s), 757.6 (s).

**1-Chloro-4-(2,2,4-trifluoro-4-phenylbutyl)benzene (2g)**

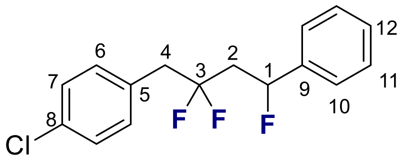


Compound **2g** was prepared according to general procedure **D** (amine:HF ratio = 1:5.5) using 1-(4-chlorophenyl)-3-phenylcyclobutan-1-ol **1g** (51.7 mg, 0.200 mmol). The crude product was purified by flash column chromatography (5-10% DCM in cyclohexane) to yield the title compound as a colorless solid (37.0 mg, 0.124 mmol, 62%).

**$R_f$** = 0.23 (10% DCM in cyclohexane).

**Melting Point:** 64-66 ˚C.

**$^1$H NMR** (500 MHz, $CDCl_3$): $\delta$ [ppm] = 7.42 – 7.35 (m, 3H, H-C10, H-C12), 7.34 – 7.30 (m, 4H, H-C6, H-C11), 7.25 (d, $^3J_{HH}$ = 8.5 Hz, 2H, H-C7), 5.80 (ddd, $^2J_{HF}$ = 48.4 Hz, $^3J_{HH}$ = 9.6 Hz, $^3J_{HH}$ = 2.4 Hz, 1H, H-C1), 3.34 – 3.19 (m, 2H, H-C4), 2.53 (dtdd, $^3J_{HF}$ = 20.0 Hz $^2J_{HH}$ = $^3J_{HF}$ = 15.5 Hz, $^3J_{HF}$ = 12.3 Hz, $^3J_{HH}$ = 9.7 Hz, 1H, $H^a$-C2), 2.23 (dtdd, $^3J_{HF}$ = 35.3 Hz, $^2J_{HH}$ = $^3J_{HF}$ = 15.1 Hz, $^3J_{HF}$ = 12.0 Hz, $^3J_{HH}$ = 2.5 Hz, 1H, $H^b$-C2).

**$^{19}$F NMR** (470 MHz, $CDCl_3$): $\delta$ [ppm] = -92.58 (ddtdd, $^2J_{FF}$ = 248.3 Hz, $^3J_{FH}$ = 19.2 Hz, $^3J_{FH}$ = 15.7 Hz, $^3J_{FH}$ = 12.2 Hz, $^4J_{FF}$ = 4.1 Hz, 1F, $F^a$-C3), -96.30 (ddtdd, $^2J_{FF}$ = 248.0 Hz, $^3J_{FH}$ = 20.1 Hz, $^3J_{FH}$ = 15.4 Hz, $^3J_{FH}$ = 12.8 Hz, $^4J_{FF}$ = 7.4 Hz, 1F, $F^b$-C3), -173.61 (ddddd, $^2J_{FH}$ = 48.4 Hz, $^3J_{FH}$ = 35.4 Hz, $^3J_{FH}$ = 15.3 Hz, $^4J_{FF}$ = 6.9 Hz, $^4J_{FF}$ = 4.1 Hz, 1F, F-C1).

**$^{19}$F{$^1$H} NMR** (470 MHz, $CDCl_3$): $\delta$ [ppm] = -92.58 (dd, $^2J_{FF}$ = 248.3 Hz, $^4J_{FF}$ = 4.0 Hz, 1F, $F^a$-C3), -96.30 (dd, $^2J_{FF}$ = 248.3 Hz, $^4J_{FF}$ = 7.4 Hz, 1F, $F^b$-C3), -173.67 (dd, $^4J_{FF}$ = 7.4 Hz, $^4J_{FF}$ = 4.0 Hz, 1F, F-C1).

**$^{13}$C{$^1$H} NMR** (126 MHz, $CDCl_3$): $\delta$ [ppm] = 139.1 (d, $^2J_{CF}$ = 19.8 Hz, C9), 133.6 (C8), 132.1 (C7), 131.5 (dd, $^3J_{CF}$ = 6.5 Hz, $^3J_{CF}$ = 2.6 Hz, C5), 129.0 (d, $^5J_{CF}$ = 2.1 Hz, C12), 128.9 (C11), 128.8 (C6), 125.6 (d, $^3J_{CF}$ = 6.6 Hz, C10), 122.4 (t, $^1J_{CF}$ = 244.2 Hz, C3), 89.6 (ddd, $^1J_{CF}$ = 172.1 Hz, $^3J_{CF}$ = 8.4 Hz, $^3J_{CF}$ = 3.8 Hz, C1), 43.4 (dt, $^2J_{CF}$ = 25.9 Hz, $^2J_{CF}$ = 24.5 Hz, C2), 42.7 (ddd, $^2J_{CF}$ = 26.5 Hz, $^2J_{CF}$ = 24.6 Hz, $^4J_{CF}$ = 2.8 Hz, C4).

**GC-EI-MS:** Retention 9.84 min, (*m/z*) requires: [$(C_{16}H_{14}ClF_3)^+$] = 298.0731, (*m/z*) found: [$(C_{16}H_{14}ClF_3)^+$] = 298.0730.

**FT-IR** ($\tilde{\nu}$ = $cm^{-1}$)**:** 1598.5 (w), 1492.3 (m), 1457.9 (w), 1439.2 (w), 1424.9 (w), 1409.1 (w), 1363.2 (w), 1341.6 (w), 1304.4 (w), 1267.0 (w), 1212.5 (m), 1160.9 (m), 1135.0 (m), 1109.2 (w), 1084.8 (m), 1059.0 (m), 1030.3 (s), 1015.9 (s), 1001.6 (w), 954.2 (m), 915.5 (w), 895.4 (m), 859.5 (m), 833.7 (s), 782.0 (s), 760.5 (s), 724.6 (m).

**1-Bromo-4-(2,2,4-trifluoro-4-phenylbutyl)benzene (2h)**

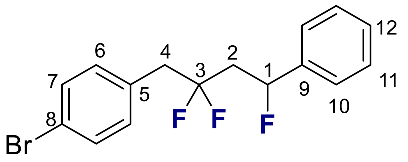


Compound **2h** was prepared according to general procedure **D** (amine:HF ratio = 1:5.5) using 1-(4-bromophenyl)-3-phenyl-cyclobutan-1-ol **1h** (60.6 mg, 0.200 mmol). The crude product was purified by flash column chromatography (5-10% DCM in cyclohexane) to yield the title compound as a colorless solid (49.4 mg, 0.144 mmol, 72%).

**R*f*** = 0.26 (10% DCM in cyclohexane).

**Melting Point:** 73-75 ˚C.

**$^1$H NMR** (500 MHz, $CDCl_3$): $\delta$ [ppm] = 7.50 – 7.46 (m, 2H, H-C7), 7.43 – 7.34 (m, 3H, H-C11, H-C12), 7.34 – 7.30 (m, 2H, H-C10), 7.20 (d, $^3J_{HH}$ = 8.3 Hz, 2H, H-C6), 5.80 (ddd, $^2J_{HF}$ = 48.2 Hz, $^3J_{HH}$ = 9.6 Hz, $^3J_{HH}$ = 2.3 Hz, 1H, H-C1), 3.34 – 3.17 (m, 2H, H-C4), 2.53 (dtdd, $^3J_{HF}$ = 20.2 Hz $^2J_{HH}$ = $^3J_{HF}$ = 15.5 Hz, $^3J_{HF}$ = 12.3 Hz, $^3J_{HH}$ = 9.7 Hz, 1H, H$^a$-C2), 2.23 (dtdd, $^3J_{HF}$ = 35.4 Hz, $^2J_{HH}$ = $^3J_{HF}$ = 15.1 Hz, $^3J_{HF}$ = 11.9 Hz, $^3J_{HH}$ = 2.5 Hz, 1H, H$^b$-C2).

**$^{19}$F NMR** (376 MHz, $CDCl_3$): $\delta$ [ppm] = -92.53 (ddtdd, $^2J_{FF}$ = 248.1 Hz, $^3J_{FH}$ = 19.3 Hz, $^3J_{FH}$ = 15.8 Hz, $^3J_{FH}$ = 12.1 Hz, $^4J_{FF}$ = 4.0 Hz, 1F, F$^a$-C3), -96.30 (ddtdd, $^2J_{FF}$ = 248.3 Hz, $^3J_{FH}$ = 20.3 Hz, $^3J_{FH}$ = 15.3 Hz, $^3J_{FH}$ = 12.8 Hz, $^4J_{FF}$ = 7.4 Hz, 1F, F$^b$-C3), -173.63 (ddddd, $^2J_{FH}$ = 48.2 Hz, $^3J_{FH}$ = 35.3 Hz, $^3J_{FH}$ = 15.3 Hz, $^4J_{FF}$ = 7.3 Hz, $^4J_{FF}$ = 3.8 Hz, 1F, F-C1).

**$^{19}$F{$^1$H} NMR** (376 MHz, $CDCl_3$): $\delta$ [ppm] = -92.53 (dd, $^2J_{FF}$ = 248.3 Hz, $^4J_{FF}$ = 4.0 Hz, 1F, F$^a$-C3), -96.30 (dd, $^2J_{FF}$ = 248.2 Hz, $^4J_{FF}$ = 7.4 Hz, 1F, F$^b$-C3), -173.63 (dd, $^4J_{FF}$ = 7.4 Hz, $^4J_{FF}$ = 3.9 Hz, 1F, F-C1).

**$^{13}$C{$^1$H} NMR** (126 MHz, $CDCl_3$): $\delta$ [ppm] = 139.1 (d, $^2J_{CF}$ = 20.0 Hz, C9), 132.4 (C6), 132.0 (dd, $^3J_{CF}$ = 6.5 Hz, $^3J_{CF}$ = 2.6 Hz, C5), 131.8 (C7), 129.0 (d, $^5J_{CF}$ = 2.1 Hz, C12), 128.9 (C11), 125.6 (d, $^3J_{CF}$ = 6.6 Hz, C10), 122.3 (dd, $^1J_{CF}$ = 244.2 Hz, $^1J_{CF}$ = 242.8 Hz, C3), 121.8 (C8), 89.6 (ddd, $^1J_{CF}$ = 172.0 Hz, $^3J_{CF}$ = 8.4 Hz, $^3J_{CF}$ = 3.7 Hz, C1), 43.4 (dt, $^2J_{CF}$ = 26.1 Hz, $^2J_{CF}$ = 24.2 Hz, C2), 42.8 (ddd, $^2J_{CF}$ = 26.2 Hz, $^2J_{CF}$ = 24.7 Hz, $^4J_{CF}$ = 2.9 Hz, C4).

**GC-EI-MS:** Retention 10.23 min, (*m/z*) requires: [$(C_{16}H_{14}BrF_3)^+$] = 342.0226, (*m/z*) found: [$(C_{16}H_{14}BrF_3)^+$] = 342.0225.

**FT-IR** ($\tilde{\nu}$ = $cm^{-1}$)**:** 1594.2 (w), 1488.0 (m), 1453.6 (w), 1436.4 (w), 1386.1 (w), 1360.3 (w), 1261.3 (w), 1211.1 (w), 1198.2 (m), 1178.1 (w), 1070.5 (m), 1037.4 (m), 1013.1 (m), 960.0 (w), 915.5 (w), 886.8 (w), 862.4 (m), 833.7 (m), 784.9 (s), 756.2 (m), 724.6 (s).

**1-(2,2,4-Trifluoro-4-phenylbutyl)-4-(trifluoromethoxy)benzene (2i)**

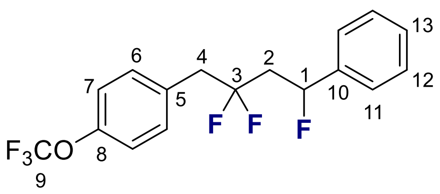


Compound **2i** was prepared according to general procedure **D** (amine:HF ratio = 1:5.5) using 3-phenyl-1-(4-(trifluoromethoxy)-phenyl)cyclobutan-1-ol **1i** (61.7 mg, 0.200 mmol). The crude product was purified by flash column chromatography (5-7.5% DCM in cyclohexane) to yield the title compound as a colorless solid (53.4 mg, 0.153 mmol, 77%).

$\mathbf{R}_f$ = 0.32 (10% DCM in cyclohexane).

**Melting Point:** 75-77 ˚C.

**$^1$H NMR** (500 MHz, $CDCl_3$): $\delta$ [ppm] = 7.43 – 7.38 (m, 2H, H-C12), 7.38 – 7.30 (m, 5H, H-C7, H-C11, H-C13), 7.23 – 7.17 (m, 2H, H-C6), 5.81 (dd, $^2J_{HF}$ = 48.2 Hz, $^3J_{HH}$ = 9.9 Hz, 1H, H-C1), 3.39 – 3.21 (m, 2H, H-C4), 2.64 – 2.46 (m, 1H, $H^a$-C2), 2.35 – 2.17 (m, 1H, $H^b$-C2).

**$^{19}$F NMR** (470 MHz, $CDCl_3$): $\delta$ [ppm] = -57.85 (s, 3F, F-C9), -92.60 (dttd, $^2J_{FF}$ = 248.6 Hz, $^3J_{FH}$ = 19.4 Hz, $^3J_{FH}$ = 12.3 Hz, $^4J_{FF}$ = 4.3 Hz, 1F, $F^a$-C3), -96.40 (dttd, $^2J_{FF}$ = 248.5 Hz, $^3J_{FH}$ = 15.3 Hz, $^3J_{FH}$ = 12.5 Hz, $^4J_{FF}$ = 7.5 Hz, 1F, $F^b$-C3), -173.51 – -173.84 (m, 1F, F-C1).

**$^{19}$F{$^1$H} NMR** (470 MHz, $CDCl_3$): $\delta$ [ppm] = -57.85 (s, 3F, F-C9), -92.60 (dd, $^2J_{FF}$ = 248.6 Hz, $^4J_{FF}$ = 4.0 Hz, 1F, $F^a$-C3), -96.40 (dd, $^2J_{FF}$ = 248.5 Hz, $^4J_{FF}$ = 7.3 Hz, 1F, $F^b$-C3), -173.63 – -173.70 (m, 1F, F-C1).

**$^{13}$C{$^1$H} NMR** (126 MHz, $CDCl_3$): $\delta$ [ppm] = 148.9 (q, $^3J_{CF}$ = 1.8 Hz, C8), 139.1 (d, $^2J_{CF}$ = 19.8 Hz, C10), 132.2 (C7), 131.8 (dd, $^3J_{CF}$ = 6.5 Hz, $^3J_{CF}$ = 2.4 Hz, C5), 129.0 (d, $^5J_{CF}$ = 2.0 Hz, C13), 128.9 (s, C12), 125.6 (d, $^3J_{CF}$ = 6.6 Hz, C11), 122.4 (t, $^1J_{CF}$ = 244.0 Hz, C3), 121.0 (C6), 120.0 (q, $^1J_{CF}$ = 257.5 Hz, C9), 89.6 (ddd, $^1J_{CF}$ = 171.9 Hz, $^3J_{CF}$ = 8.5 Hz, $^3J_{CF}$ = 3.8 Hz, C1), 43.5 (dt, $^2J_{CF}$ = 26.4 Hz, $^2J_{CF}$ = 24.3 Hz, C2), 42.6 (ddd, $^2J_{CF}$ = 26.8 Hz, $^2J_{CF}$ = 24.8 Hz, $^4J_{CF}$ = 3.0 Hz, C4).

**GC-EI-MS:** Retention 8.93 min, (*m/z*) requires: [$(C_{17}H_{14}F_6O)^+$] = 348.0943, (*m/z*) found: [$(C_{17}H_{14}F_6O)^+$] = 348.0941.

**FT-IR** ($\tilde{\nu}$ = $cm^{-1}$)**:** 1509.5 (w), 1459.3 (w), 1403.4 (w), 1211.1 (m), 1199.6 (m), 1153.7 (s), 1132.2 (s), 1106.3 (m), 1084.8 (w), 1063.3 (w), 1020.2 (m), 1001.6 (w), 954.2 (w), 921.2 (w), 915.5 (w), 886.8 (w), 856.6 (m), 823.6 (w), 805.0 (w), 773.4 (m), 761.9 (s), 736.1 (w).

**1-(2,2,4-Trifluoro-4-phenylbutyl)-4-(trifluoromethyl)benzene (2j)**

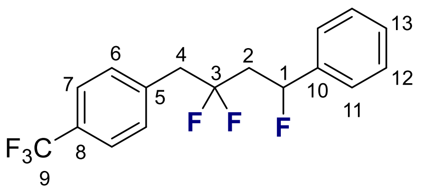


Compound **2j** was prepared according to general procedure **D** (amine:HF ratio = 1:6.5) using 3-phenyl-1-(4-(trifluoromethyl)-phenyl)cyclobutan-1-ol **1j** (58.5 mg, 0.200 mmol). The crude product was purified by flash column chromatography (5-7.5% DCM in cyclohexane) to yield the title compound as a colorless solid (44.6 mg, 0.134 mmol, 67%).

**$R_f$** = 0.31 (10% DCM in cyclohexane).

**Melting Point:** 91-93 ˚C.

**$^1$H NMR** (500 MHz, $CDCl_3$): $\delta$ [ppm] = 7.61 (d, $^3J_{HH}$ = 8.1 Hz, 2H, H-C7), 7.45 (d, $^3J_{HH}$ = 8.0 Hz, 2H, H-C6), 7.43 – 7.35 (m, 3H, H-C12, H-C13), 7.33 (d, $^3J_{HH}$ = 8.0 Hz, 2H, H-C11), 5.82 (ddd, $^2J_{HF}$ = 48.4 Hz, $^3J_{HH}$ = 9.7 Hz, $^3J_{HH}$ = 2.4 Hz, 1H, H-C1), 3.45 – 3.28 (m, 2H, H-C4), 2.55 (dtdd, $^3J_{HF}$ = 21.1 Hz, $^2J_{HH}$ = $^3J_{HF}$ = 15.5 Hz, $^3J_{HF}$ = 11.6 Hz, $^3J_{HH}$ = 9.7 Hz, 1H, $H^a$-C2), 2.27 (dtdd, $^3J_{HF}$ = 35.5 Hz, $^2J_{HH}$ = $^3J_{HF}$ = 15.0 Hz, $^3J_{HF}$ = 12.0 Hz, $^3J_{HH}$ = 2.5 Hz, 1H, $H^b$-C2).

**$^{19}$F NMR** (470 MHz, $CDCl_3$): $\delta$ [ppm] = -62.59 (s, 3F, F-C9), -92.25 (ddtdd, $^2J_{FF}$ = 249.1 Hz, $^3J_{FH}$ = 19.4 Hz, $^3J_{FH}$ = 15.6 Hz, $^3J_{FH}$ = 11.8 Hz, $^4J_{FF}$ = 3.9 Hz, 1F, $F^a$-C3), -96.23 (ddtdd, $^2J_{FF}$ = 249.1 Hz, $^3J_{FH}$ = 21.6 Hz, $^3J_{FH}$ = 15.8 Hz, $^3J_{FH}$ = 11.8 Hz, $^4J_{FF}$ = 7.0 Hz, 1F, $F^b$-C3), -173.62 (ddddd, $^2J_{FH}$ = 48.5 Hz, $^3J_{FH}$ = 35.3 Hz, $^3J_{FH}$ = 15.8 Hz, $^4J_{FF}$ = 7.1 Hz, $^4J_{FF}$ = 4.0 Hz, 1F, F-C1).

**$^{19}$F{$^1$H} NMR** (470 MHz, $CDCl_3$): $\delta$ [ppm] = -62.59 (s, 3F, F-C9), -92.25 (dd, $^2J_{FF}$ = 249.1 Hz, $^4J_{FF}$ = 3.9 Hz, 1F, $F^a$-C3), -96.23 (dd, $^2J_{FF}$ = 249.1 Hz, $^4J_{FF}$ = 7.0 Hz, 1F, $F^b$-C3), -173.62 (dd, $^4J_{FF}$ = 7.1 Hz, $^4J_{FF}$ = 3.9 Hz, 1F, F-C1).

**$^{13}$C{$^1$H} NMR** (126 MHz, $CDCl_3$): $\delta$ [ppm] = 139.0 (d, $^2J_{CF}$ = 19.8 Hz, C10), 137.8 (d, $^3J_{CF}$ = 6.1 Hz, C5), 131.1 (q, $^4J_{CF}$ = 1.1 Hz, C6), 130.0 (q, $^2J_{CF}$ = 32.5 Hz, C8), 129.1 (d, $^5J_{CF}$ = 2.1 Hz, C13), 128.9 (C12), 125.6 (d, $^3J_{CF}$ = 6.6 Hz, C11), 125.7 (q, $^3J_{CF}$ = 3.7 Hz, C7), 124.3 (q, $^1J_{CF}$ = 272.0 Hz, C9), 122.3 (t, $^1J_{CF}$ = 243.8 Hz, C3), 89.6 (ddd, $^1J_{CF}$ = 171.8 Hz, $^3J_{CF}$ = 8.6 Hz, $^3J_{CF}$ = 3.7 Hz, C1), 43.6 (dt, $^2J_{CF}$ = 26.1 Hz, $^2J_{CF}$ = 24.2 Hz, C2), 43.2 (ddd, $^2J_{CF}$ = 26.5 Hz, $^2J_{CF}$ = 24.6 Hz, $^4J_{CF}$ = 3.1 Hz, C4).

**GC-EI-MS:** Retention 8.93 min, (*m/z*) requires: [$(C_{17}H_{14}F_6)^+$] = 332.0994, (*m/z*) found: [$(C_{17}H_{14}F_6)^+$] = 332.0994.

**FT-IR** ($\tilde{\nu}$ = $cm^{-1}$)**:** 1621.5 (w), 1495.2 (w), 1459.3 (w), 1432.0 (w), 1417.7 (w), 1400.5 (w), 1366.0 (w), 1324.4 (m), 1284.3 (w), 1211.1 (w), 1191.0 (w), 1119.2 (s), 1110.6 (s), 1084.8 (w), 1064.7 (s), 1028.8 (m), 1018.8 (m), 1001.6 (w), 952.8 (w), 915.5 (w), 886.8 (w), 856.6 (m), 840.9 (m), 823.6 (w), 790.6 (m), 769.1 (m), 759.1 (s), 728.9 (m).

**4-(2,2,4-Trifluoro-4-phenylbutyl)benzonitrile (2k)**

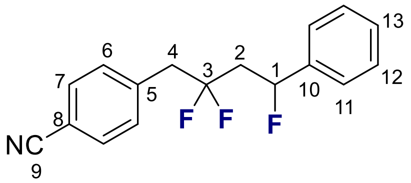


Compound **2k** was prepared according to general procedure **D** (amine:HF ratio = 1:6.5) using 4-(1-hydroxy-3-phenylcyclobutyl)-benzonitrile **1k** (49.9 mg, 0.200 mmol). The crude product was purified by flash column chromatography (5% EtOAc in cyclohexane) to yield the title compound as a yellow oil (26.0 mg, 0.090 mmol, 45%).

**$R_f$** = 0.23 (10% EtOAc in cyclohexane).

**Melting Point:** 51-53 ˚C.

**$^1$H NMR** (500 MHz, $CDCl_3$): $\delta$ [ppm] = 7.66 – 7.62 (m, 2H, H-C7), 7.44 (d, $^3J_{HH}$ = 8.0 Hz, 2H, H-C6), 7.42 – 7.35 (m, 3H, H-C12, H-C13), 7.34 – 7.30 (m, 2H, H-C11), 5.80 (ddd, $^2J_{HF}$ = 48.3 Hz, $^3J_{HH}$ = 10.1 Hz, $^3J_{HH}$ = 2.5 Hz, 1H, H-C1), 3.46 – 3.26 (m, 2H, H-C4), 2.53 (dtdd, $^3J_{HF}$ = 21.8 Hz, $^2J_{HH}$ = $^3J_{HF}$ = 15.4 Hz, $^3J_{HF}$ = 10.8 Hz, $^3J_{HH}$ = 9.6 Hz, 1H, $H^a$-C2), 2.27 (dtdd, $^3J_{HF}$ = 36.0 Hz, $^2J_{HH}$ = $^3J_{HF}$ = 14.7 Hz, $^3J_{HF}$ = 12.6 Hz, $^3J_{HH}$ = 2.5 Hz, 1H, $H^b$-C2).

**$^{19}$F NMR** (470 MHz, $CDCl_3$): $\delta$ [ppm] = -91.99 (ddtdd, $^2J_{FF}$ = 249.1 Hz, $^3J_{FH}$ = 22.0 Hz, $^3J_{FH}$ = 15.4 Hz, $^3J_{FH}$ = 11.7 Hz, $^4J_{FF}$ = 3.8 Hz, 1F, $F^a$-C3), -96.30 (dddddd, $^2J_{FF}$ = 249.8 Hz, $^3J_{FH}$ = 21.3 Hz, $^3J_{FH}$ = 17.3 Hz, $^3J_{FH}$ = 14.2 Hz, $^3J_{FH}$ = 10.8 Hz, $^4J_{FF}$ = 7.0 Hz, 1F, $F^b$-C3), -173.60 (ddddd, $^2J_{FH}$ = 47.0 Hz, $^3J_{FH}$ = 35.7 Hz, $^3J_{FH}$ = 15.6 Hz, $^4J_{FF}$ = 6.6 Hz, $^4J_{FF}$ = 3.8 Hz, 1F, F-C1).

**$^{19}$F{$^1$H} NMR** (470 MHz, $CDCl_3$): $\delta$ [ppm] = -91.99 (dd, $^2J_{FF}$ = 249.8 Hz, $^4J_{FF}$ = 3.8 Hz, 1F, $F^a$-C3), -96.30 (dd, $^2J_{FF}$ = 249.8 Hz, $^4J_{FF}$ = 6.6 Hz, 1F, $F^b$-C3), -173.60 (dd, $^4J_{FF}$ = 6.6 Hz, $^4J_{FF}$ = 3.8 Hz, 1F, F-C1).

**$^{13}$C{$^1$H} NMR** (126 MHz, $CDCl_3$): $\delta$ [ppm] = 138.8 (d, $^2J_{CF}$ = 19.8 Hz, C10), 138.4 (dd, $^3J_{CF}$ = 6.1 Hz, $^3J_{CF}$ = 2.2 Hz, C5), 132.3 (C7), 131.5 (d, $^4J_{CF}$ = 1.1 Hz, C6), 129.1 (d, $^5J_{CF}$ = 2.1 Hz, C13), 128.9 (C12), 125.5 (d, $^3J_{CF}$ = 6.7 Hz, C11), 122.0 (dd, $^1J_{CF}$ = 244.7 Hz, $^1J_{CF}$ = 243.1 Hz, C3), 118.7 (C9), 111.7 (C8), 89.5 (ddd, $^1J_{CF}$ = 171.9 Hz, $^3J_{CF}$ = 8.9 Hz, $^3J_{CF}$ = 3.6 Hz, C1), 43.7 (dt, $^2J_{CF}$ = 26.2 Hz, $^2J_{CF}$ = 24.3 Hz, C2), 43.3 (ddd, $^2J_{CF}$ = 26.5 Hz, $^2J_{CF}$ = 24.6 Hz, $^4J_{CF}$ = 3.3 Hz, C4).

**GC-EI-MS:** Retention 10.33 min, (*m/z*) requires: [$(C_{17}H_{14}F_3N)^+$] = 289.1073, (*m/z*) found: [$(C_{17}H_{14}F_3N)^+$] = 289.1072.

**FT-IR** ($\tilde{\nu}$ = $cm^{-1}$)**:** 3066.4 (w), 3033.4 (w), 2943.0 (w), 2237.0 (w), 2227.0 (w), 1611.4 (w), 1506.7 (w), 1455.0 (w), 1436.4 (w), 1416.3 (w), 1387.6 (m), 1357.4 (m), 1284.3 (w), 1264.2 (w), 1213.9 (m), 1196.8 (s), 1179.5 (m), 1158.0 (w), 1136.5 (w), 1116.4 (s), 1086.2 (w), 1067.6 (m), 1041.8 (s), 1001.6 (w), 965.7 (w), 915.5 (w), 891.1 (m), 865.3 (m), 839.4 (s), 793.5 (s), 764.8 (m), 744.7 (s).

**4-(2,2,4-Trifluoro-4-phenylbutyl)-4'-(trifluoromethyl)-1,1'-biphenyl (2l)**

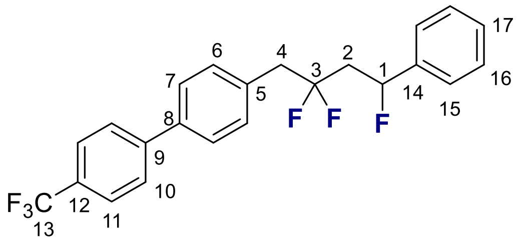


Compound **2l** was prepared according to general procedure **D** (amine:HF ratio = 1:5.5) using 3-phenyl-1-(4'-(trifluoromethyl)-[1,1'-biphenyl]-4-yl)cyclobutan-1-ol **1l** (73.7 mg, 0.200 mmol). The crude product was purified by flash column chromatography (0-7% DCM in *n*-pentane) to yield the title compound as a colorless solid (48.1 mg, 0.118 mmol, 59%).

**R*f*** = 0.18 (5% DCM in *n*-pentane).

**Melting Point:** 154-156 °C.

**$^1$H NMR** (500 MHz, $CDCl_3$): $\delta$ [ppm] = 7.77 – 7.68 (m, 4H, H-C10, H-C11), 7.63 – 7.55 (m, 2H, H-C7), 7.50 – 7.33 (m, 7H, H-C6, H-C15, H-C16, H-C17), 5.85 (ddd, $^2J_{HF}$ = 48.2 Hz, $^3J_{HH}$ = 9.6 Hz, $^3J_{HH}$ = 2.5 Hz, 1H, H-C1), 3.48 – 3.26 (m, 2H, H-C4), 2.60 (dtdd, $^3J_{HF}$ = 19.5 Hz, $^2J_{HH}$ = $^3J_{HF}$ = 15.6 Hz, $^3J_{HF}$ = 12.9 Hz, $^3J_{HH}$ = 9.6 Hz, 1H, $H^a$-C2), 2.27 (dtdd, $^3J_{HF}$ = 34.1 Hz, $^2J_{HH}$ = $^3J_{HF}$ = 15.3 Hz, $^3J_{HF}$ = 12.0 Hz, $^3J_{HH}$ = 2.6 Hz, 1H, $H^b$-C2).

**$^{19}$F NMR** (470 MHz, $CDCl_3$): $\delta$ [ppm] = -62.41 (s, 3F, F-C12), -92.47 (ddtdd, $^2J_{FF}$ = 247.5 Hz, $^3J_{FH}$ = 17.8 Hz, $^3J_{FH}$ = 15.8 Hz, $^3J_{FH}$ = 12.3 Hz, $^4J_{FF}$ = 4.1 Hz, 1F, $F^a$-C3), -96.09 (ddtdd, $^2J_{FF}$ = 247.7 Hz, $^3J_{FH}$ = 20.1 Hz, $^3J_{FH}$ = 15.7 Hz, $^3J_{FH}$ = 13.3 Hz, $^4J_{FF}$ = 7.4 Hz, 1F, $F^b$-C3), -173.60 (ddddd, $^2J_{FH}$ = 47.4 Hz, $^3J_{FH}$ = 35.8 Hz, $^3J_{FH}$ = 15.1 Hz, $^4J_{FF}$ = 6.8 Hz, $^4J_{FF}$ = 3.7 Hz, 1F, F-C1).

**$^{19}$F{$^1$H} NMR** (470 MHz, $CDCl_3$): $\delta$ [ppm] = -62.41 (s, 3F, F-C12), -92.47 (dd, $^2J_{FF}$ = 247.7 Hz, $^4J_{FF}$ = 4.1 Hz, 1F, $F^a$-C3), -96.09 (dd, $^2J_{FF}$ = 247.8 Hz, $^4J_{FF}$ = 7.7 Hz, 1F, $F^b$-C3), -173.60 (dd, $^4J_{FF}$ = 7.6 Hz, $^4J_{FF}$ = 4.1 Hz, 1F, F-C1).

**$^{13}$C{$^1$H} NMR** (126 MHz, $CDCl_3$): $\delta$ [ppm] = 144.4 (C9), 139.2 (d, $^2J_{CF}$ = 19.8 Hz, C14), 139.0 (C8), 133.2 (dd, $^3J_{CF}$ = 6.3 Hz, $^3J_{CF}$ = 2.7 Hz, C5), 131.4 (C6), 129.6 (q, $^2J_{CF}$ = 32.5 Hz, C12), 128.9 (C16), 127.5 (C7 and C10), 125.9 (q, $^3J_{CF}$ = 3.8 Hz, C11), 125.6 (d, $^3J_{CF}$ = 6.6 Hz, C15), 122.6 (dd, $^1J_{CF}$ = 244.2 Hz, $^1J_{CF}$ = 242.7 Hz, C3), 124.4 (q, $^1J_{CF}$ = 272.5 Hz, C13), 89.6 (ddd, $^1J_{CF}$ = 171.9 Hz, $^3J_{CF}$ = 8.1 Hz, $^3J_{CF}$ = 3.7 Hz, C1), 43.5 (dt, $^2J_{CF}$ = 26.0 Hz, $^2J_{CF}$ = 24.3 Hz, C2), 43.3 (ddd, $^2J_{CF}$ = 26.9 Hz, $^2J_{CF}$ = 24.8 Hz, $^4J_{CF}$ = 2.6 Hz, C4).

**GC-EI-MS:** Retention 10.77 min, (*m/z*) requires: [$(C_{23}H_{18}F_6)^+$] = 408.1307, (*m/z*) found: [$(C_{23}H_{18}F_6)^+$] = 408.1307.

**FT-IR** ($\tilde{\nu}$ = $cm^{-1}$)**:** 2925.8 (broad), 2360.4 (broad), 1338.8 (s), 1325.9 (m), 1275.6 (w), 1159.4 (w), 1123.5 (m), 1077.6 (m), 1024.5 (w), 859.5 (w), 832.3 (w), 789.2 (w), 763.4 (w), 744.7 (w), 697.4 (w).

**1-Bromo-3-(2,2,4-trifluoro-4-phenylbutyl)benzene (2m)**

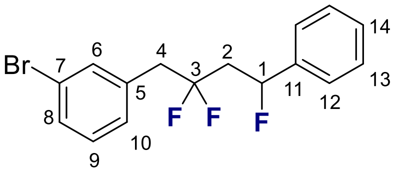


Compound **2m** was prepared according to general procedure **D** (amine:HF ratio = 1:5.5) using 1-(3-bromophenyl)-3-phenylcyclobutan-1-ol **1m** (60.6 mg, 0.200 mmol). The crude product was purified by flash column chromatography (0-2% DCM in *n*-pentane) to yield the title compound as a colorless solid (48.1 mg, 0.140 mmol, 70%).

$\mathbf{R_f}$ = 0.24 (5% DCM in *n*-pentane).

**Melting point:** 37-39 °C.

**$^{1}$H NMR** (500 MHz, $CDCl_3$): $\delta$ [ppm] = 7.51 – 7.47 (m, 1H, H-C6), 7.45 (dt, $^{3}J_{HH}$ = 7.7 Hz, $^{4}J_{HH}$ = 1.7 Hz, 1H, H-C8), 7.43 – 7.36 (m, 3H, H-C10, H-C13), 7.35 – 7.32 (m, 2H, H-C12), 7.28 – 7.24 (m, 1H, H-C14), 7.22 (t, $^{3}J_{HH}$ = 7.7 Hz, 1H, H-C9), 5.81 (ddd, $^{2}J_{HF}$ = 48.2 Hz, $^{3}J_{HH}$ = 9.6 Hz, $^{3}J_{HH}$ = 2.5 Hz, 1H, H-C1), 3.48 – 3.26 (m, 2H, H-C4), 2.55 (dtdd, $^{3}J_{HF}$ = 20.0 Hz, $^{2}J_{HH}$ = $^{3}J_{HF}$ = 15.5 Hz, $^{3}J_{HF}$ = 12.3 Hz, $^{3}J_{HH}$ = 9.6 Hz, 1H, $H^{a}$-C2), 2.25 (ddddd, $^{3}J_{HF}$ = 34.3 Hz, $^{3}J_{HF}$ = 17.3 Hz, $^{2}J_{HH}$ = 14.7 Hz, $^{3}J_{HF}$ = 11.7 Hz, $^{3}J_{HH}$ = 2.6 Hz, 1H, $H^{b}$-C2).

**$^{19}$F NMR** (376 MHz, $CDCl_3$): $\delta$ [ppm] = -92.33 (ddtdd, $^{2}J_{FF}$ = 248.8 Hz, $^{3}J_{FH}$ = 18.5 Hz, $^{3}J_{FH}$ = 15.5 Hz, $^{3}J_{FH}$ = 12.3 Hz, $^{4}J_{FF}$ = 4.0 Hz, 1F, $F^{a}$-C3), -96.13 (ddtdd, $^{2}J_{FF}$ = 248.8 Hz, $^{3}J_{FH}$ = 20.0 Hz, $^{3}J_{FH}$ = 15.5 Hz, $^{3}J_{FH}$ = 12.7 Hz, $^{4}J_{FF}$ = 7.6 Hz, 1F, $F^{b}$-C3), -173.65 (ddddd, $^{2}J_{FH}$ = 47.7 Hz, $^{3}J_{FH}$ = 34.9 Hz, $^{3}J_{FH}$ = 15.4 Hz, $^{4}J_{FF}$ = 7.6 Hz, $^{4}J_{FF}$ = 4.0 Hz, 1F, F-C1).

**$^{19}$F{$^{1}$H} NMR** (376 MHz, $CDCl_3$): $\delta$ [ppm] = -92.33 (dd, $^{2}J_{FF}$ = 248.8 Hz, $^{4}J_{FF}$ = 4.1 Hz, 1F, $F^{a}$-C3), -96.13 (dd, $^{2}J_{FF}$ = 248.8 Hz, $^{4}J_{FF}$ = 7.4 Hz, 1F, $F^{b}$-C3), -173.65 (dd, $^{4}J_{FF}$ = 7.4 Hz, $^{4}J_{FF}$ = 4.0 Hz, 1F, F-C1).

**$^{13}$C{$^{1}$H} NMR** (126 MHz, $CDCl_3$): $\delta$ [ppm] = 139.1 (d, $^{2}J_{CF}$ = 19.9 Hz, C11), 135.3 (dd, $^{3}J_{CF}$ = 6.3 Hz, $^{3}J_{CF}$ = 2.7 Hz, C5), 133.7 (d, $^{4}J_{CF}$ = 1.1 Hz, C6), 130.8 (C8), 130.1 (C9), 129.4 (C14), 129.0 (d, $^{4}J_{CF}$ = 2.1 Hz, C10), 128.9 (C13), 125.6 (d, $^{3}J_{CF}$ = 6.7 Hz, C12), 122.6 (C7), 122.3 (dd, $^{1}J_{CF}$ = 244.4 Hz, $^{1}J_{CF}$ = 242.9 Hz, C3), 89.6 (ddd, $^{1}J_{CF}$ = 171.9 Hz, $^{3}J_{CF}$ = 8.3 Hz, $^{3}J_{CF}$ = 3.8 Hz, C1), 43.5 (dt, $^{2}J_{CF}$ = 26.0 Hz, $^{2}J_{CF}$ = 23.7 Hz, C2), 42.9 (ddd, $^{2}J_{CF}$ = 26.5 Hz, $^{2}J_{CF}$ = 24.8 Hz, $^{4}J_{CF}$ = 3.0 Hz, C4).

**GC-EI-MS:** Retention 10.15 min, (*m/z*) requires: [$(C_{16}H_{14}BrF_3)^{+}$] = 342.0226, (*m/z*) found: [$(C_{16}H_{14}BrF_3)^{+}$] = 342.0224.

**FT-IR** ($\tilde{\nu}$ = $cm^{-1}$)**:** 2340.4 (broad), 1597.1 (w), 1569.8 (w), 1476.5 (w), 1456.4 (w), 1429.2 (w), 1211.1 (m), 1153.7 (m), 1117.8 (m), 1094.8 (m),1073.3 (s), 1061.8 (s), 1037.4 (s), 997.3 (w), 972.9 (w), 896.8 (w), 859.5 (m), 774.9 (s), 756.2 (s), 717.8 (m).

**1-Bromo-2-(2,2,4-trifluoro-4-phenylbutyl)benzene (2n)**

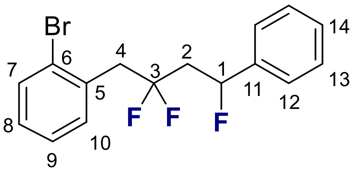


Compound **2n** was prepared according to modified general procedure **D** (amine:HF ratio = 1:5.5) using 1-(2-bromophenyl)-3-phenylcyclobutan-1-ol **1n** (60.6 mg, 0.200 mmol). The reaction time was elongated to 42 h. The crude product was purified by flash column chromatography (5-10% DCM in *n*-pentane) to yield the title compound as a colorless oil (35.1 mg, 0.102 mmol, 51%).

**R*f*** = 0.24 (5% DCM in *n*-pentane).

**$^{1}$H NMR** (500 MHz, $CDCl_3$): $\delta$ [ppm] = 7.59 (dd, $^{3}J_{HH}$ = 8.0 Hz, $^{4}J_{HH}$ = 1.3 Hz, 1H, H-C7), 7.44 – 7.41 (m, 1H, H-C10), 7.41 – 7.33 (m, 5H, H-C12, H-C13, H-C14), 7.30 (td, $^{3}J_{HH}$ = 7.5 Hz, $^{4}J_{HH}$ = 1.3 Hz, 1H, H-C9), 7.16 (td, $^{3}J_{HH}$ = 7.7 Hz, $^{4}J_{HH}$ = 1.7 Hz ,1H, H-C8), 5.84 (ddd, $^{2}J_{HF}$ = 48.1 Hz, $^{3}J_{HH}$ = 9.2 Hz, $^{3}J_{HH}$ = 2.6 Hz, 1H, H-C1), 3.65 – 3.40 (m, 2H, H-C4), 2.66 (ddtd, $^{3}J_{HF}$ = 17.7 Hz, $^{3}J_{HF}$ = 16.4 Hz, $^{2}J_{HH}$ = $^{3}J_{HF}$ = 15.4 Hz, $^{3}J_{HH}$ = 9.2 Hz, 1H, $H^{a}$-C2), 2.25 (ddddd, $^{3}J_{HF}$ = 33.3 Hz, $^{3}J_{HF}$ = 18.3 Hz, $^{2}J_{HH}$ = 15.6 Hz, $^{3}J_{HF}$ = 10.5 Hz, $^{3}J_{HH}$ = 2.7 Hz, 1H, $H^{b}$-C2).

**$^{19}$F NMR** (470 MHz, $CDCl_3$): $\delta$ [ppm] = -93.53 (dddddd, $^{2}J_{FF}$ = 246.6 Hz, $^{3}J_{FH}$ = 21.3 Hz, $^{3}J_{FH}$ = 17.9 Hz, $^{3}J_{FH}$ = 14.4 Hz, $^{3}J_{FH}$ = 10.7 Hz, $^{4}J_{FF}$ = 4.4 Hz, 1F, $F^{a}$-C3), -95.98 – -96.70 (m, 1F, $F^{b}$-C3), -172.83 (ddddd, $^{2}J_{FH}$ = 48.5 Hz, $^{3}J_{FH}$ = 32.3 Hz, $^{3}J_{FH}$ = 15.2 Hz, $^{4}J_{FF}$ = 10.1 Hz, $^{4}J_{FF}$ = 4.2 Hz, 1F, F-C1).

**$^{19}$F{$^{1}$H} NMR** (470 MHz, $CDCl_3$): $\delta$ [ppm] = -93.53 (dd, $^{2}J_{FF}$ = 246.6 Hz, $^{4}J_{FF}$ = 4.3 Hz, 1F, $F^{a}$-C3), -96.33 (dd, $^{2}J_{FF}$ = 248.8 Hz, $^{4}J_{FF}$ = 10.1 Hz, 1F, $F^{b}$-C3), -172.83 (dd, $^{4}J_{FF}$ = 10.2 Hz, $^{4}J_{FF}$ = 4.2 Hz, 1F, F-C1).

**$^{13}$C{$^{1}$H} NMR** (126 MHz, $CDCl_3$): $\delta$ [ppm] = 139.4 (d, $^{2}J_{CF}$ = 19.8 Hz, C11), 133.3 (C7), 132.9 (dd, $^{3}J_{CF}$ = 7.3 Hz, $^{3}J_{CF}$ = 3.8 Hz, C5), 132.6 (C10), 129.3 (C8), 128.9 (d, $^{4}J_{CF}$ = 2.1 Hz, C13), 128.8 (C14), 127.6 (C9), 126.0 (C6), 125.7 (d, $^{3}J_{CF}$ = 6.6 Hz, C12), 122.4 (dd, $^{1}J_{CF}$ = 243.7 Hz, $^{1}J_{CF}$ = 243.0 Hz, C3), 89.3 (ddd, $^{1}J_{CF}$ = 172.3 Hz, $^{3}J_{CF}$ = 6.8 Hz, $^{3}J_{CF}$ = 3.5 Hz, C1), 44.0 (q, $^{2}J_{CF}$ = 24.5 Hz, C2), 42.6 (td, $^{2}J_{CF}$ = 25.4 Hz, $^{4}J_{CF}$ = 2.4 Hz, C4).

**GC-EI-MS:** Retention 9.41 min, (*m/z*) requires: $[(C_{16}H_{14}BrF_3)^{+}]$ = 342.0226, (*m/z*) found: $[(C_{16}H_{14}BrF_3)^{+}]$ = 342.0225.

**FT-IR** ($\tilde{\nu}$ = $cm^{-1}$)**:** 2363.3 (broad), 1341.6 (w), 1277.1 (w), 1269.6 (w), 1114.9 (m), 1083.4 (m), 1061.8 (m), 1041.8 (m), 1015.9 (w), 849.5 (w), 822.2 (w), 749.0 (s), 720.3 (w).

**1-Fluoro-4-(1,3,3-trifluoro-4-phenylbutyl)benzene (2o)**

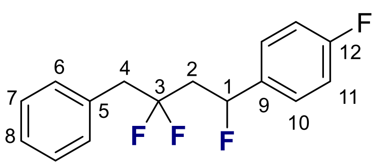


Compound **2o** was prepared according to general procedure **D** (amine:HF ratio = 1:4.5) using 3-(4-fluorophenyl)-1-phenyl-cyclobutan-1-ol **1o** (48.5 mg, 0.200 mmol). The crude product was purified by flash column chromatography (5-10% DCM in cyclohexane) to yield the title compound as a colorless solid (27.8 mg, 0.098 mmol, 49%).

**R*f*** = 0.18 (10% DCM in cyclohexane).

**Melting Point:** 51-53 ˚C.

**$^1$H NMR** (599 MHz, $CDCl_3$): $\delta$ [ppm] = 7.38 – 7.28 (m, 2H, H-C7), 7.33 – 7.28 (m, 5H, H-C6, H-C8, H-C10), 7.09 – 7.05 (m, 2H, H-C11), 5.79 (ddd, $^2J_{HF}$ = 47.8 Hz, $^3J_{HH}$ = 9.3 Hz, $^3J_{HH}$ = 2.8 Hz, 1H, H-C1), 3.36 – 3.21 (m, 2H, H-C4), 2.54 (dtdd, $^3J_{HF}$ = 17.9 Hz, $^2J_{HH}$ = $^3J_{HF}$ = 15.6 Hz, $^3J_{HF}$ = 14.4 Hz, $^3J_{HH}$ = 9.3 Hz, 1H, H$^a$-C2), 2.20 (dtdd, $^3J_{HF}$ = 33.7 Hz, $^2J_{HH}$ = $^3J_{HF}$ = 15.6 Hz, $^3J_{HF}$ = 11.5 Hz, $^3J_{HH}$ = 2.9 Hz, 1H, H$^b$-C2).

**$^{19}$F NMR** (564 MHz, $CDCl_3$): $\delta$ [ppm] = -92.79 (ddtdd, $^2J_{FF}$ = 247.4 Hz, $^3J_{FH}$ = 18.7 Hz, $^3J_{FH}$ = 15.7 Hz $^3J_{FH}$ = 12.1 Hz, $^4J_{FF}$ = 4.3 Hz, 1F, F$^a$-C3), -95.96 (dttd, $^2J_{FF}$ = 247.4 Hz, $^3J_{FH}$ = 17.3 Hz, $^3J_{FH}$ = 14.2 Hz, $^4J_{FF}$ = 7.8 Hz, 1F, F$^b$-C3), -112.83 (ttd, $^3J_{FH}$ = 8.5 Hz, $^4J_{FH}$ = 5.2 Hz, $^6J_{FF}$ = 3.4 Hz, 1F, F-C12), -171.48 (ddddt, $^2J_{FH}$ = 48.4 Hz, $^3J_{FH}$ = 33.7 Hz, $^3J_{FH}$ = 15.4 Hz, $^4J_{FF}$ = 7.8 Hz, $^4J_{FF}$ = $^6J_{FF}$ = 3.6 Hz, 1F, F-C1).

**$^{19}$F{$^1$H} NMR** (564 MHz, $CDCl_3$): $\delta$ [ppm] = -92.79 (dd, $^2J_{FF}$ = 247.4 Hz, $^4J_{FF}$ = 4.3 Hz, 1F, F$^a$-C3), -95.96 (dd, $^2J_{FF}$ = 247.4 Hz, $^4J_{FF}$ = 7.9 Hz, 1F, F$^b$-C3), -112.83 (d, $^6J_{FF}$ = 3.4 Hz, 1F, F-C12), -171.48 (dt, $^4J_{FF}$ = 7.9 Hz, $^4J_{FF}$ = $^6J_{FF}$ = 3.9 Hz, 1F, F-C1).

**$^{13}$C{$^1$H} NMR** (151 MHz, $CDCl_3$): $\delta$ [ppm] = 163.0 (dd, $^1J_{CF}$ = 247.6 Hz, $^5J_{CF}$ = 2.4 Hz, C12), 135.2 (dd, $^2J_{CF}$ = 20.4 Hz, $^4J_{CF}$ = 3.3 Hz, C9), 133.0 (dd, $^3J_{CF}$ = 6.4 Hz, $^3J_{CF}$ = 3.1 Hz, C5), 130.7 (C6), 128.7 (C7), 127.6 (C8), 127.6 (dd, $^3J_{CF}$ = 8.4 Hz, $^3J_{CF}$ = 6.4 Hz, C10), 122.6 (t, $^1J_{CF}$ = 243.4 Hz, C3), 115.8 (d, $^2J_{CF}$ = 21.7 Hz, C11), 89.0 (ddd, $^1J_{CF}$ = 172.1 Hz, $^3J_{CF}$ = 7.7 Hz, $^3J_{CF}$ = 4.0 Hz, C1), 43.5 (ddd, $^2J_{CF}$ = 26.0 Hz, $^2J_{CF}$ = 24.7 Hz, $^4J_{CF}$ = 2.6 Hz, C4), 43.3 (q, $^2J_{CF}$ = 25.4 Hz, C2).

**GC-EI-MS:** Retention 8.36 min, (*m/z*) requires: [$(C_{16}H_{14}F_4)^+$] = 282.1026, (*m/z*) found: [$(C_{16}H_{14}F_4)^+$] = 282.1025.

**FT-IR** ($\tilde{\nu}$ = cm$^{-1}$)**:** 3066.4 (w), 3037.7 (w), 1604.2 (m), 1512.4 (m), 1495.2 (m), 1455.0 (w), 1429.2 (w), 1394.7 (m), 1361.7 (w), 1341.6 (w), 1323.0 (w), 1287.1 (w), 1268.5 (w), 1225.4 (m), 1211.1 (m), 1158.0 (m), 1136.5 (s), 1100.6 (m), 1081.9 (m), 1067.6 (m), 1024.5 (s), 1013.1 (m), 954.2 (m), 937.0 (w), 892.55 (m), 865.3 (m), 848.0 (m), 836.6 (s), 787.3 (m), 737.6 (s), 720.3 (m).

**1-Chloro-4-(1,3,3-trifluoro-4-phenylbutyl)benzene (2p)**

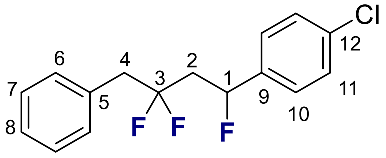


Compound **2p** was prepared according to general procedure **D** (amine:HF ratio = 1:5.0) using 3-(4-chlorophenyl)-1-phenyl-cyclobutan-1-ol **1p** (51.8 mg, 0.200 mmol). The crude product was purified by flash column chromatography (0-3% DCM in *n*-pentane) to yield the title compound as a colorless solid (40.0 mg, 0.134 mmol, 67%).

**R*f*** = 0.30 (5% DCM in in *n*-pentane).

**Melting Point:** 72-74 °C.

**$^1$H NMR** (599 MHz, $CDCl_3$): $\delta$ [ppm] = 7.37 – 7.33 (m, 4H, H-C7, H-C11), 7.33 – 7.29 (m, 3H, H-C6, H-C8), 7.27 – 7.23 (m, 2H, H-C10), 5.78 (ddd, $^2J_{HF}$ = 47.8 Hz, $^3J_{HH}$ = 9.3 Hz, $^3J_{HH}$ = 2.8 Hz, 1H, H-C1), 3.36 – 3.20 (m, 2H, H-C4), 2.51 (dqd, $^3J_{HF}$ = 17.6 Hz, $^2J_{HH}$ = $^3J_{HF}$ = 15.5 Hz, $^3J_{HH}$ = 9.3 Hz, 1H, $H^a$-C2), 2.20 (dtdd, $^3J_{HF}$ = 32.1 Hz, $^2J_{HH}$ = $^3J_{HF}$ = 14.6 Hz, $^3J_{HF}$ = 11.7 Hz, $^3J_{HH}$ = 2.4 Hz, 1H, $H^b$-C2).

**$^{19}$F NMR** (564 MHz, $CDCl_3$): $\delta$ [ppm] = -92.85 (ddtdd, $^2J_{FF}$ = 247.3 Hz, $^3J_{FH}$ = 19.1 Hz, $^3J_{FH}$ = 15.3 Hz, $^3J_{FH}$ = 11.6 Hz, $^4J_{FF}$ = 4.2 Hz, 1F, $F^a$-C3), -95.96 (ddtdd, $^2J_{FF}$ = 247.3 Hz, $^3J_{FH}$ = 17.5 Hz, $^3J_{FH}$ = 15.8 Hz $^3J_{FH}$ =14.3 Hz, $^4J_{FF}$ = 8.1 Hz, 1F, $F^b$-C3), -173.92 (ddddd, $^2J_{FH}$ = 48.3 Hz, $^3J_{FH}$ = 34.0 Hz, $^3J_{FH}$ = 15.7 Hz, $^4J_{FF}$ = 8.0 Hz, $^4J_{FF}$ = 4.2 Hz, 1F, F-C1).

**$^{19}$F{$^1$H} NMR** (564 MHz, $CDCl_3$): $\delta$ [ppm] = -92.85 (dd, $^2J_{FF}$ = 247.3 Hz, $^4J_{FF}$ = 4.2 Hz, 1F, $F^a$-C3), -95.96 (dd, $^2J_{FF}$ = 247.3 Hz, $^4J_{FF}$ = 8.1 Hz, $F^b$-C3), -173.92 (dd, $^4J_{FF}$ = 8.1 Hz, $^4J_{FF}$ = 4.3 Hz, 1F, F-C1).

**$^{13}$C{$^1$H} NMR** (151 MHz, $CDCl_3$): $\delta$ [ppm] = 137.8 (d, $^2J_{CF}$ = 20.3 Hz, C9), 134.8 (d, $^5J_{CF}$ = 2.4 Hz, C12), 132.9 (dd, $^3J_{CF}$ = 6.4 Hz, $^3J_{CF}$ = 3.1 Hz, C5), 130.7 (C6), 129.0 (C11), 128.7 (C7), 127.6 (C8), 127.0 (d, $^3J_{CF}$ = 6.7 Hz, C10), 122.62 (dd, $^1J_{CF}$ = 242.8 Hz, $^1J_{CF}$ = 242.0 Hz, C3), 88.9 (ddd, $^1J_{CF}$ = 172.9 Hz, $^3J_{CF}$ = 7.6 Hz, $^3J_{CF}$ = 3.8 Hz, C1), 43.7 – 42.9 (m, C2, C4).

**GC-EI-MS:** Retention 9.09 min, (*m/z*) requires: [$(C_{16}H_{14}ClF_3)^+$] = 298.0731, (*m/z*) found: [$(C_{16}H_{14}ClF_3)^+$] = 298.0721.

**FT-IR** ($\tilde{\nu}$ = $cm^{-1}$)**:** 1492.3 (m), 1455.0 (w), 1430.6 (w), 1386.1 (m), 1275.6 (s), 1267.0 (s), 1261.3 (s), 1212.5 (w), 1192.4 (m), 1119.2 (m), 1083.4 (s), 1051.8 (s), 1030.3 (m), 1013.1 (m), 971.4 (w), 882.7 (m), 871.0 (m), 850.9 (s), 828.0 (m), 809.3 (s), 764.8 (s), 751.9 (s), 718.9 (s).

**1-Bromo-4-(1,3,3-trifluoro-4-phenylbutyl)benzene (2q)**

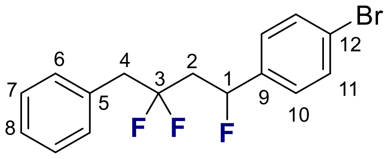


Compound **2q** was prepared according to general procedure **D** (amine:HF ratio = 1:5.0) using 3-(4-bromophenyl)-1-phenyl-cyclobutan-1-ol **1q** (60.6 mg, 0.200 mmol). The crude product was purified by flash column chromatography (5-10% DCM in cyclohexane) to yield the title compound as a colorless solid (42.4 mg, 0.124 mmol, 62%).

**R*f*** = 0.31 (10% DCM in cyclohexane).

**Melting Point:** 42-44 ˚C.

**$^{1}$H NMR** (500 MHz, $CDCl_3$): $\delta$ [ppm] = 7.54 – 7.49 (m, 2H, H-C11), 7.38 – 7.29 (m, 5H, H-C6, H-C7, H-C8), 7.21 – 7.17 (m, 2H, H-C10), 5.77 (ddd, $^{2}J_{HF}$ = 47.8 Hz, $^{3}J_{HH}$ = 9.3 Hz, $^{3}J_{HH}$ = 2.9 Hz, 1H, H-C1), 3.37 – 3.19 (m, 2H, H-C4), 2.51 (dqd, $^{3}J_{HF}$ = 17.5 Hz $^{2}J_{HH}$ = $^{3}J_{HF}$ = 15.6 Hz, $^{3}J_{HH}$ = 9.3 Hz, 1H, $H^{a}$-C2), 2.23 (dtdd, $^{3}J_{HF}$ = 33.4 Hz, $^{2}J_{HH}$ = $^{3}J_{HF}$ = 14.8 Hz, $^{3}J_{HF}$ = 11.4 Hz, $^{3}J_{HH}$ = 2.9 Hz, 1H, $H^{b}$-C2).

**$^{19}$F NMR** (470 MHz, $CDCl_3$): $\delta$ [ppm] = -92.84 (ddtdd, $^{2}J_{FF}$ = 247.5 Hz, $^{3}J_{FH}$ = 19.0 Hz, $^{3}J_{FH}$ = 15.3 Hz, $^{3}J_{FH}$ = 11.3 Hz, $^{4}J_{FF}$ = 4.3 Hz, 1F, $F^{a}$-C3), -95.94 (dttd, $^{2}J_{FF}$ = 247.1 Hz, $^{3}J_{FH}$ = 17.5 Hz, $^{3}J_{FH}$ = 14.3 Hz, $^{4}J_{FF}$ = 8.1 Hz, 1F, $F^{b}$-C3), -174.39 (ddddd, $^{2}J_{FH}$ = 47.8 Hz, $^{3}J_{FH}$ = 34.0 Hz, $^{3}J_{FH}$ = 15.9 Hz, $^{4}J_{FF}$ = 8.0 Hz, $^{4}J_{FF}$ = 4.3 Hz, 1F, F-C1).

**$^{19}$F{$^{1}$H} NMR** (470 MHz, $CDCl_3$): $\delta$ [ppm] = -92.84 (dd, $^{2}J_{FF}$ = 247.3 Hz, $^{4}J_{FF}$ = 4.3 Hz, 1F, $F^{a}$-C3), -95.94 (dd, $^{2}J_{FF}$ = 247.3 Hz, $^{4}J_{FF}$ = 8.1 Hz, 1F, $F^{b}$-C3), -174.39 (dd, $^{4}J_{FF}$ = 8.1 Hz, $^{4}J_{FF}$ = 4.3 Hz, 1F, F-C1).

**$^{13}$C{$^{1}$H} NMR** (126 MHz, $CDCl_3$): $\delta$ [ppm] = 138.4 (d, $^{2}J_{CF}$ = 20.2 Hz, C9), 132.9 (dd, $^{3}J_{CF}$ = 6.4 Hz, $^{3}J_{CF}$ = 3.2 Hz, C5), 132.0 (C11), 130.7 (C6), 128.7 (C7), 127.6 (C8), 127.3 (d, $^{3}J_{CF}$ = 6.7 Hz, C10), 122.9 (d, $^{5}J_{CF}$ = 2.5 Hz, C12), 122.2 (t, $^{1}J_{CF}$ = 243.5 Hz, C3), 88.9 (ddd, $^{1}J_{CF}$ = 173.1 Hz, $^{3}J_{CF}$ = 7.6 Hz, $^{3}J_{CF}$ = 3.9 Hz, C1), 43.5 (ddd, $^{2}J_{CF}$ = 25.9 Hz, $^{2}J_{CF}$ = 24.7 Hz, $^{4}J_{CF}$ = 2.9 Hz, C4), 42.8 (dt, $^{2}J_{CF}$ = 25.8 Hz, $^{2}J_{CF}$ = 24.3 Hz, C2).

**GC-EI-MS:** Retention 9.44 min, (*m/z*) requires: [$(C_{16}H_{14}BrF_3)^{+}$] = 342.0225, (*m/z*) found: [$(C_{16}H_{14}BrF_3)^{+}$] = 342.0226.

**FT-IR** ($\tilde{\nu}$ = $cm^{-1}$)**:** 3036.3 (w), 1595.6 (w), 1495.2 (w), 1488.0 (w), 1455.0 (w), 1433.5 (w), 1423.4 (w), 1406.2 (w), 1377.5 (m), 1343.1 (w), 1313.0 (w), 1294.3 (w),1282.8 (w), 1265.6 (w), 1224.0 (w), 1208.2 (w), 1153.7 (m), 1140.8 (m), 1104.9 (w), 1080.5 (m), 1061.8 (s), 1026.0 (m), 1010.2 (m), 975.7 (m), 968.6 (m), 894.0 (m), 866.7 (m), 845.2 (s), 830.8 (m), 817.9 (s), 803.6 (m), 743.3 (s), 714.6 (m).

**1-(1,3,3-Trifluoro-4-phenylbutyl)-4-(trifluoromethyl)benzene (2r)**

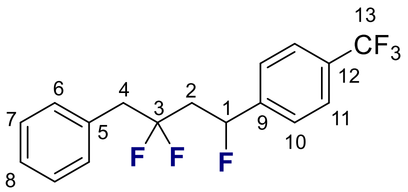


Compound **2r** was prepared according to general procedure **D** (amine:HF ratio = 1:6.5) using 1-phenyl-3-(4-(trifluoromethyl)-phenyl)cyclobutan-1-ol **1r** (58.5 mg, 0.200 mmol). The crude product was purified by flash column chromatography (5-10% DCM in cyclohexane) to yield the title compound as a colorless solid (43.8 mg, 0.132 mmol, 66%).

**$R_f$** = 0.23 (10% DCM in cyclohexane).

**Melting Point:** 52-54 ˚C.

**$^1$H NMR** (500 MHz, $CDCl_3$): $\delta$ [ppm] = 7.65 (d, $^3J_{HH}$ = 8.1 Hz, 2H, H-C11), 7.43 (d, $^3J_{HH}$ = 8.0 Hz, 2H, H-C10), 7.38 – 7.29 (m, 5H, H-C6, H-C7, H-C8), 5.87 (ddd, $^2J_{HF}$ = 48.1 Hz, $^3J_{HH}$ = 9.4 Hz, $^3J_{HH}$ = 2.8 Hz, 1H, H-C1), 3.39 – 3.21 (m, 2H, H-C4), 2.52 (pd, $^2J_{HH}$ = $^3J_{HF}$ = 16.0 Hz, $^3J_{HH}$ = 9.3 Hz, 1H, $H^a$-C2), 2.27 (ddddd, $^3J_{HF}$ = 33.5 Hz, $^3J_{HF}$ = 18.1 Hz, $^2J_{HH}$ = 15.5 Hz, $^3J_{HF}$ = 11.1 Hz, $^3J_{HH}$ = 2.8 Hz, 1H, $H^b$-C2).

**$^{19}$F NMR** (470 MHz, $CDCl_3$): $\delta$ [ppm] = -62.74 (s, 3F, F-C9), -92.92 (ddtdd, $^2J_{FF}$ = 247.1 Hz, $^3J_{FH}$ = 19.4 Hz, $^3J_{FH}$ = 15.6 Hz, $^3J_{FH}$ = 11.2 Hz, $^4J_{FF}$ = 4.2 Hz, 1F, $F^a$-C3), -95.71 (dttd, $^2J_{FF}$ = 247.1 Hz, $^3J_{FH}$ = 17.3 Hz, $^3J_{FH}$ = 14.4 Hz, $^4J_{FF}$ = 8.3 Hz, 1F, $F^b$-C3), -177.08 – -177.37 (m, 1F, F-C1).

**$^{19}$F{$^1$H} NMR** (470 MHz, $CDCl_3$): $\delta$ [ppm] = -62.74 (s, 3F, F-C13), -92.92 (dd, $^2J_{FF}$ = 247.3 Hz, $^4J_{FF}$ = 4.2 Hz, 1F, $F^a$-C3), -95.98 (dd, $^2J_{FF}$ = 247.3 Hz, $^4J_{FF}$ = 8.3 Hz, 1F, $F^b$-C3), -177.22 (dd, $^4J_{FF}$ = 8.7 Hz, $^4J_{FF}$ = 4.1 Hz, 1F, F-C1).

**$^{13}$C{$^1$H} NMR** (126 MHz, $CDCl_3$): $\delta$ [ppm] = 143.3 (dq, $^2J_{CF}$ = 20.2 Hz, $^5J_{CF}$ = 1.2 Hz, C9), 132.9 (dd, $^3J_{CF}$ = 6.4 Hz, $^3J_{CF}$ = 3.2 Hz, C5), 131.0 (q, $^2J_{CF}$ = 34.2 Hz, C12), 130.6 (C6), 128.7 (C8), 127.7 (C7), 125.9 (q, $^3J_{CF}$ = 3.8 Hz, C11), 125.8 (d, $^3J_{CF}$ = 7.1 Hz, C10), 124.0 (q, $^1J_{CF}$ = 272.1 Hz, C13), 122.0 (dd, $^1J_{CF}$ = 244.2 Hz, $^1J_{CF}$ = 243.2 Hz, C3), 88.8 (ddd, $^1J_{CF}$ = 174.3 Hz, $^3J_{CF}$ = 7.5 Hz, $^3J_{CF}$ = 3.8 Hz, C1), 43.6 (ddd, $^2J_{CF}$ = 26.0 Hz, $^2J_{CF}$ = 24.9 Hz, $^4J_{CF}$ = 2.3 Hz, C4), 43.4 (dt, $^2J_{CF}$ = 25.7 Hz, $^2J_{CF}$ = 24.4 Hz, C2).

**GC-EI-MS:** Retention 8.88 min, (*m/z*) requires: [$(C_{17}H_{14}F_6)^+$] = 332.0994, (*m/z*) found: [$(C_{17}H_{14}F_6)^+$] = 332.0994.

**FT-IR** ($\tilde{\nu}$ = $cm^{-1}$)**:** 1621.5 (w), 1495.2 (w), 1456.4 (w), 1447.8 (w), 1414.8 (w), 1389.0 (w), 1324.4 (s), 1268.5 (w), 1246.9 (w), 1215.4 (w), 1196.7 (m), 1189.5 (m), 1165.2 (m), 1153.7 (m), 1130.7 (m), 1112.1 (s), 1084.8 (s), 1054.7 (s), 1030.3 (w), 1015.9 (m), 1004.4 (w), 977.2 (w), 875.3 (m), 852.3 (m), 836.6 (m), 815.0 (w), 764.8 (w), 743.3 (s).

**4-(1,3,3-Trifluoro-4-phenylbutyl)phenyl trifluoromethanesulfonate (2s)**

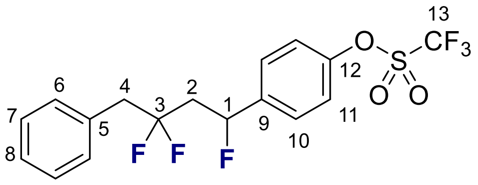


Compound **2s** was prepared according to general procedure **D** (amine:HF ratio = 1:5.5) using 4-(3-hydroxy-3-phenylcyclobutyl)phenyl trifluoromethanesulfonate **1s** (74.5 mg, 0.200 mmol). The crude product was purified by flash column chromatography (2.5-3% EtOAc in cyclohexane) to yield the title compound as a colorless solid (55.4 mg, 0.134 mmol, 67%).

**R*f*** = 0.30 (5% EtOAc in cyclohexane).

**Melting Point:** 53-55 ˚C.

**$^{1}$H NMR** (500 MHz, $CDCl_3$): $\delta$ [ppm] = 7.43 – 7.38 (m, 2H, H-C10), 7.38 – 7.33 (m, 3H, H-C7, H-C8), 7.33 – 7.28 (m, 4H, H-C6, H-C11), 5.84 (ddd, $^{2}J_{HF}$ = 47.7 Hz, $^{3}J_{HH}$ = 9.3 Hz, $^{3}J_{HH}$ = 2.8 Hz, 1H, H-C1), 3.39 – 3.20 (m, 2H, H-C4), 2.51 (pd, $^{2}J_{HH}$ = $^{3}J_{HF}$ = 16.0 Hz, $^{3}J_{HH}$ = 9.2 Hz, 1H, $H^{a}$-C2), 2.21 (ddddd, $^{3}J_{HF}$ = 33.5 Hz, $^{3}J_{HF}$ =18.1 Hz, $^{2}J_{HH}$ = 15.5 Hz, $^{3}J_{HF}$ = 11.0 Hz, $^{3}J_{HH}$ = 2.9 Hz, 1H, $H^{b}$-C2).

**$^{19}$F NMR** (470 MHz, $CDCl_3$): $\delta$ [ppm] = -72.84 (s, F-C13), -92.90 (dttd, $^{2}J_{FF}$ = 247.5 Hz, $^{3}J_{FH}$ = 16.4 Hz, $^{3}J_{FH}$ = 15.6 Hz, $^{4}J_{FF}$ = 4.1 Hz, 1F, $F^{a}$-C3), -95.98 (dttd, $^{2}J_{FF}$ = 247.5 Hz, $^{3}J_{FH}$ = 17.3 Hz, $^{3}J_{FH}$ = 14.4 Hz, $^{4}J_{FF}$ = 8.6 Hz, 1F, $F^{b}$-C3), -175.43 (ddddd, $^{2}J_{FH}$ = 47.2 Hz, $^{3}J_{FH}$ = 33.7 Hz, $^{3}J_{FH}$ = 16.4 Hz, $^{4}J_{FF}$ = 8.2 Hz, $^{4}J_{FF}$ = 4.1 Hz, 1F, F-C1).

**$^{19}$F{$^{1}$H} NMR** (376 MHz, $CDCl_3$): $\delta$ [ppm] = -72.80 (s, F-C13), -92.90 (dd, $^{2}J_{FF}$ = 247.5 Hz, $^{4}J_{FF}$ = 4.2 Hz, 1F, $F^{a}$-C3), -95.98 (dd, $^{2}J_{FF}$ = 247.5 Hz, $^{4}J_{FF}$ = 8.3 Hz, 1F, $F^{b}$-C3), -175.45 (dd, $^{4}J_{FF}$ = 8.4 Hz, $^{4}J_{FF}$ = 4.2 Hz, 1F, F-C1).

**$^{13}$C{$^{1}$H} NMR** (126 MHz, $CDCl_3$): $\delta$ [ppm] = 149.6 (d, $^{5}J_{CF}$ = 2.1 Hz, C12), 139.9 (d, $^{2}J_{CF}$ = 20.5 Hz, C9), 132.8 (dd, $^{3}J_{CF}$ = 6.3 Hz, $^{3}J_{CF}$ = 3.3 Hz, C5), 130.6 (C6), 128.7 (C7), 127.7 (C8), 127.5 (d, $^{3}J_{CF}$ = 7.0 Hz, C10), 122.5 (t, $^{1}J_{CF}$ = 243.2 Hz, C3), 121.9 (C11), 118.9 (q, $^{1}J_{CF}$ = 320.8 Hz, C13), 88.5 (ddd, $^{1}J_{CF}$ = 174.1 Hz, $^{3}J_{CF}$ = 7.3 Hz, $^{3}J_{CF}$ = 3.8 Hz, C1), 43.5 (ddd, $^{2}J_{CF}$ = 25.9 Hz, $^{2}J_{CF}$ = 24.8 Hz, $^{4}J_{CF}$ = 2.2 Hz, C4), 43.3 (dt, $^{2}J_{CF}$ = 26.0 Hz, $^{2}J_{CF}$ = 24.1 Hz, C2).

**ESI-MS:** (*m/z*) requires: [$(C_{17}H_{14}F_6O_3SNa)^+$] = 435.0460, (*m/z*) found: [$(C_{17}H_{14}F_6O_3SNa)^+$] = 435.0460.

**FT-IR** ($\tilde{\nu}$ = $cm^{-1}$)**:** 1605.7 (w), 1505.2 (w), 1457.9 (w), 1429.2 (m), 1404.8 (w), 1386.2 (w), 1350.3 (w), 1284.3 (w), 1252.7 (w), 1199.6 (s), 1179.5 (w), 1135.0 (s), 1114.9 (m), 1089.1 (m), 1067.6 (w), 1036.0 (w), 1018.8 (w), 1004.4 (w), 964.3 (w), 886.8 (m), 865.3 (m), 855.2 (m), 848.0 (s), 833.7 (w), 776.3 (w), 757.6 (w), 736.1 (w), 724.6 (w), 700.2 (m).

**1-Bromo-4-(2,2,4-trifluoro-4-(4-fluorophenyl)butyl)benzene (2t)**

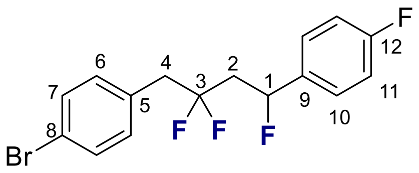


Compound **2t** was prepared according to general procedure **D** (amine:HF ratio = 1:5.0) using 1-(4-bromophenyl)-3-(4-fluorophenyl)cyclobutan-1-ol **1t** (64.2 mg, 0.200 mmol). The crude product was purified by flash column chromatography (5-10% DCM in cyclohexane) to yield the title compound as a colorless solid (49.0 mg, 0.136 mmol, 68%).

**R*f*** = 0.24 (10% DCM in cyclohexane).

**Melting Point:** 43-45 ˚C.

**$^1$H NMR** (599 MHz, $CDCl_3$): $\delta$[ppm] = 7.49 – 7.46 (m, 2H, H-C7), 7.30 (ddd, $^3J_{HH}$ = 8.7 Hz, $^4J_{HF}$ = 5.2 Hz, $J_{HH}$ = 1.3 Hz, 2H, H-C10), 7.18 (d, $^3J_{HH}$ = 8.1 Hz, 2H, H-C6), 7.10 – 7.05 (m, 2H, H-C11), 5.77 (ddd, $^2J_{HF}$ = 47.9 Hz, $^3J_{HH}$ = 9.6 Hz, $^3J_{HH}$ = 2.7 Hz, 1H, H-C1), 3.38 – 3.14 (m, 2H, H-C4), 2.51 (dtdd, $^3J_{HF}$ = 19.5 Hz, $^2J_{HH}$ = $^3J_{HF}$ = 15.5 Hz, $^3J_{HF}$ = 12.9 Hz, $^3J_{HH}$ = 9.5 Hz, 1H, H$^a$-C2), 2.20 (dtdd, $^3J_{HF}$ = 34.3 Hz, $^2J_{HH}$ = $^3J_{HF}$ = 15.5 Hz, $^3J_{HF}$ = 11.6 Hz, $^3J_{HH}$ = 2.7 Hz, 1H, H$^b$-C2).

**$^{19}$F NMR** (564 MHz, $CDCl_3$): $\delta$ [ppm] = -92.68 (ddtdd, $^2J_{FF}$ = 248.5 Hz, $^3J_{FH}$ = 19.3 Hz, $^3J_{FH}$ = 16.2 Hz, $^3J_{FH}$ = 12.1 Hz, $^4J_{FF}$ = 4.0 Hz, 1F, F$^a$-C3), -96.39 (ddtdd, $^2J_{FF}$ = 248.5 Hz, $^3J_{FH}$ = 19.6 Hz, $^3J_{FH}$ = 15.6 Hz, $^3J_{FH}$ = 12.3 Hz, $^4J_{FF}$ = 7.3 Hz, 1F, F$^b$-C3), -112.63 (ttd, $^3J_{FH}$ = 8.6 Hz, $^4J_{FH}$ = 5.2 Hz, $^6J_{FF}$ = 3.5 Hz, 1F, F-C12). -171.33 – -171.33 (m, 1F, F-C1).

**$^{19}$F{$^1$H} NMR** (564 MHz, $CDCl_3$): $\delta$ [ppm] = -92.68 (dd, $^2J_{FF}$ = 248.5 Hz, $^4J_{FF}$ = 4.0 Hz, 1F, F$^a$-C3), -96.39 (dd, $^2J_{FF}$ = 248.5 Hz, $^4J_{FF}$ = 7.3 Hz, 1F, F$^b$-C3), -112.63 (d, $^6J_{FF}$ = 3.5 Hz, 1F, F-C12) -171.45 (dt, $^4J_{FF}$ = 7.3 Hz, $^4J_{FF}$ = $^6J_{FF}$ = 3.7 Hz, 1F, F-C1).

**$^{13}$C{$^1$H} NMR** (151 MHz, $CDCl_3$): $\delta$ [ppm] = 163.0 (dd, $^1J_{CF}$ = 247.8 Hz, $^5J_{CF}$ = 2.4 Hz, C12), 135.0 (dd, $^2J_{CF}$ = 20.3 Hz, $^4J_{CF}$ = 3.3 Hz, C9), 132.4 (C6), 131.9 (dd, $^3J_{CF}$ = 6.6 Hz, $^3J_{CF}$ = 2.6 Hz, C5), 131.8 (C7), 127.6 (dd, $^3J_{CF}$ = 8.4 Hz, $^3J_{CF}$ = 6.5 Hz, C10), 122.2 (dd, $^1J_{CF}$ = 244.3 Hz, $^1J_{CF}$ = 242.9 Hz, C3), 121.8 (C8), 115.9 (dd, $^2J_{CF}$ = 21.7 Hz, C11), 89.0 (ddd, $^1J_{CF}$ = 172.0 Hz, $^3J_{CF}$ = 8.3 Hz, $^3J_{CF}$ = 3.7 Hz, C1), 43.3 (q, $^2J_{CF}$ = 24.7 Hz, C2), 42.8 (ddd, $^2J_{CF}$ = 26.3 Hz, $^2J_{CF}$ = 24.8 Hz, $^4J_{CF}$ = 2.8 Hz, C4).

**GC-EI-MS:** Retention 10.16 min, (*m/z*) requires: [$(C_{16}H_{13}BrF_4)^+$] = 360.0131, (*m/z*) found: [$(C_{16}H_{13}BrF_4)^+$] = 360.0130.

**FT-IR** ($\tilde{\nu}$ = cm$^{-1}$)**:** 1604.2 (w), 1512.4 (w), 1489.4 (w), 1423.4 (w), 1407.7 (w), 1381.8 (w), 1343.1(w), 1290.0 (w), 1264.2 (w), 1222.6 (m), 1155.1 (m), 1139.3 (m), 1107.8 (w), 1096.3 (w), 1063.3 (m), 1026.0 (w), 1013.1 (m), 971.4 (w), 895.4 (w), 873.9 (w), 838.0 (s), 786.3 (m), 777.7 (s), 728.9 (w).

**1-Bromo-4-(4-(4-chlorophenyl)-2,2,4-trifluorobutyl)benzene (2u)**

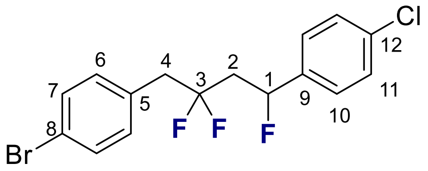


Compound **2u** was prepared according to general procedure **D** (amine:HF ratio = 1:5.5) using 1-(4-bromophenyl)-3-(4-chlorophenyl)cyclobutan-1-ol **1u** (67.5 mg, 0.200 mmol). The crude product was purified by flash column chromatography (5-10% DCM in cyclohexane) to yield the title compound as a colorless solid (58.0 mg, 0.154 mmol, 77%).

**$R_f$** = 0.27 (10% DCM in cyclohexane).

**Melting Point:** 84-86 ˚C.

**$^1$H NMR** (500 MHz, $CDCl_3$): $\delta$[ppm] = 7.49 – 7.45 (m, 2H, H-C7), 7.39 – 7.34 (m, 2H, H-C11), 7.27 – 7.23 (m, 2H, H-C10), 7.18 (d, $^3J_{HH}$ = 8.2 Hz, 2H, H-C6), 5.77 (ddd, $^2J_{HF}$ = 47.9 Hz, $^3J_{HH}$ = 9.4 Hz, $^3J_{HH}$ = 2.6 Hz, 1H, H-C1), 3.32 – 3.15 (m, 2H, H-C4), 2.49 (dtdd, $^3J_{HF}$ = 19.2 Hz, $^2J_{HH}$ = $^3J_{HF}$ = 15.6 Hz, $^3J_{HF}$ = 13.3 Hz, $^3J_{HH}$ = 9.5 Hz, 1H, H$^a$-C2), 2.19 (dtdd, $^3J_{HF}$ = 34.5 Hz, $^2J_{HH}$ = $^3J_{HF}$ = 15.7 Hz, $^3J_{HF}$ = 11.4 Hz, $^3J_{HH}$ = 2.8 Hz, 1H, H$^b$-C2).

**$^{19}$F NMR** (376 MHz, $CDCl_3$): $\delta$ [ppm] = -92.72 (ddtdd, $^2J_{FF}$ = 248.4 Hz, $^3J_{FH}$ = 19.3 Hz, $^3J_{FH}$ = 15.8 Hz, $^3J_{FH}$ = 12.4 Hz, $^4J_{FF}$ = 3.7 Hz, 1F, F$^a$-C3), -96.39 (ddtdd, $^2J_{FF}$ = 248.1 Hz, $^3J_{FH}$ = 19.7 Hz, $^3J_{FH}$ = 15.7 Hz, $^3J_{FH}$ = 12.6 Hz, $^4J_{FF}$ = 7.4 Hz, 1F, F$^b$-C3), -173.91 (ddddd, $^2J_{FH}$ = 47.3 Hz, $^3J_{FH}$ = 34.7 Hz, $^3J_{FH}$ = 15.4 Hz, $^4J_{FF}$ = 6.9 Hz, $^4J_{FF}$ = 3.7 Hz, 1F, F-C1).

**$^{19}$F{$^1$H} NMR** (376 MHz, $CDCl_3$): $\delta$[ppm] = -92.72 (dd, $^2J_{FF}$ = 248.4 Hz, $^4J_{FF}$ = 3.9 Hz, 1F, F$^a$-C3), -96.39 (dd, $^2J_{FF}$ = 248.1 Hz, $^4J_{FF}$ = 7.4 Hz, 1F, F$^b$-C3), -173.91 (dd, $^4J_{FF}$ = 7.4 Hz, $^4J_{FF}$ = 3.9 Hz, 1F, F-C1).

**$^{13}$C{$^1$H} NMR** (126 MHz, $CDCl_3$): $\delta$[ppm] = 137.6 (d, $^2J_{CF}$ = 20.3 Hz, C9), 134.9 (d, $^5J_{CF}$ = 2.5 Hz, C12), 132.4 (C6), 131.9 (dd, $^3J_{CF}$ = 6.6 Hz, $^3J_{CF}$ = 2.4 Hz, C5), 131.8 (C7), 129.1 (C11), 127.0 (d, $^3J_{CF}$ = 6.7 Hz, C10), 122.2 (dd, $^1J_{CF}$ = 244.4 Hz, $^1J_{CF}$ = 242.9 Hz, C3), 121.9 (C8), 89.9 (ddd, $^1J_{CF}$ = 172.8 Hz, $^3J_{CF}$ = 8.1 Hz, $^3J_{CF}$ = 3.7 Hz, C1), 43.3 (dt, $^2J_{CF}$ = 26.4 Hz, $^2J_{CF}$ = 24.4 Hz, C2), 42.8 (ddd, $^2J_{CF}$ = 26.2 Hz, $^2J_{CF}$ = 24.8 Hz, $^4J_{CF}$ = 2.5 Hz, C4).

**GC-EI-MS:** Retention 10.92 min, (*m/z*) requires: [$(C_{16}H_{13}BrClF_3)^+$] = 375.9836, (*m/z*) found: [$(C_{16}H_{13}BrClF_3)^+$] = 375.9835.

**FT-IR** ($\tilde{\nu}$ = $cm^{-1}$)**:** 1592.8 (w), 1489.4 (m), 1406.2 (w), 1379.0 (w), 1343.1 (w), 1315.8 (w), 1294.3 (w), 1261.3 (w), 1218.2 (w), 1155.1 (w), 1140.8 (m), 1103.5 (w), 1089.1 (m), 1064.7 (m), 1028.8 (m), 1013.1 (s), 975.7 (m), 896.8 (w), 879.6 (m), 853.8 (m), 836.6 (s), 823.6 (m), 810.7 (m), 780.6 (s), 728.9 (m), 714.6 (w).

**1-Bromo-4-(2,2,4-trifluoro-4-(4-(trifluoromethyl)phenyl)butyl)benzene (2v)**

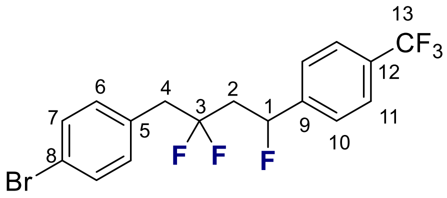


Compound **2v** was prepared according to general procedure **D** (amine:HF ratio = 1:6.5) using 1-(4-bromophenyl)-3-(4-(trifluoromethyl)phenyl)cyclobutan-1-ol **1v** (74.2 mg, 0.200 mmol). The crude product was purified by flash column chromatography (5-10% DCM in cyclohexane) to yield the title compound as a colorless solid (57.8 mg, 0.141 mmol, 71%).

**R*f*** = 0.22 (10% DCM in cyclohexane).

**Melting Point:** 74-76 ˚C.

**$^1$H NMR** (500 MHz, $CDCl_3$): $\delta$[ppm] = 7.68 – 7.63 (m, 2H, H-C11), 7.50 – 7.46 (m, 2H, H-C6), 7.46 – 7.40 (m, 2H, H-C10), 7.22 – 7.16 (m, 2H, H-C7), 5.86 (ddd, $^2J_{HF}$ = 48.0 Hz, $^3J_{HH}$ = 9.3 Hz, $^3J_{HH}$ = 2.2 Hz, 1H, H-C1), 3.35 – 3.15 (m, 2H, H-C4), 2.49 (dtdd, $^3J_{HF}$ = 18.4 Hz, $^2J_{HH}$ = $^3J_{HF}$ = 15.7 Hz, $^3J_{HF}$ = 14.0 Hz, $^3J_{HH}$ = 9.4 Hz, 1H, H$^a$-C2), 2.22 (dtdd, $^3J_{HF}$ = 34.8 Hz, $^2J_{HH}$ = $^3J_{HF}$ = 16.3 Hz, $^3J_{HF}$ = 11.0 Hz, $^3J_{HH}$ = 2.7 Hz, 1H, H$^b$-C2).

**$^{19}$F NMR** (470 MHz, $CDCl_3$): $\delta$ [ppm] = -62.76 (s, 3F, F-C13), -92.84 (ddtdd, $^2J_{FF}$ = 248.6 Hz, $^3J_{FH}$ = 18.6 Hz, $^3J_{FH}$ = 14.8 Hz, $^3J_{FH}$ = 11.6 Hz, $^4J_{FF}$ = 3.7 Hz, 1F, F$^a$-C3), -96.47 (ddtdd, $^2J_{FF}$ = 248.6 Hz, $^3J_{FH}$ = 20.2 Hz, $^3J_{FH}$ = 16.2 Hz, $^3J_{FH}$ = 12.7 Hz, $^4J_{FF}$ = 7.8 Hz, 1F, F$^b$-C3), -177.24 (ddddd, $^2J_{FH}$ = 47.8 Hz, $^3J_{FH}$ = 34.6 Hz, $^3J_{FH}$ = 15.8 Hz, $^4J_{FF}$ = 7.1 Hz, $^4J_{FF}$ = 3.1 Hz, 1F, F-C1).

**$^{19}$F{$^1$H} NMR** (470 MHz, $CDCl_3$): $\delta$ [ppm] = -62.76 (d, $^7J_{FF}$ = 1.1 Hz, 3F, F-C13), -92.84 (dd, $^2J_{FF}$ = 248.5 Hz, $^4J_{FF}$ = 3.9 Hz, 1F, F$^a$-C3), -96.47 (dd, $^2J_{FF}$ = 248.5 Hz, $^4J_{FF}$ = 7.7 Hz, 1F, F$^b$-C3), -177.24 (ddd, $^4J_{FF}$ = 7.8 Hz, $^4J_{FF}$ = 3.8 Hz, $^7J_{FF}$ = 1.2 Hz, 1F, F-C1).

**$^{13}$C{$^1$H} NMR** (126 MHz, $CDCl_3$): $\delta$ [ppm] = 143.1 (dq, $^2J_{CF}$ = 20.2 Hz, $^5J_{CF}$ = 1.4 Hz, C9), 132.4 (C7), 131.9 (C6), 131.8 (dd, $^3J_{CF}$ = 6.5 Hz, $^3J_{CF}$ = 2.9 Hz, C5), 131.1 (qd, $^2J_{CF}$ = 32.6 Hz, $^5J_{CF}$ = 1.6 Hz, C12), 125.9 (q, $^3J_{CF}$ = 3.8 Hz, C11), 125.8 (d, $^3J_{CF}$ = 7.3 Hz, C10), 124.0 (q, $^1J_{CF}$ = 272.3 Hz, C13), 122.1 (dd, $^1J_{CF}$ = 244.5 Hz, $^1J_{CF}$ = 243.1 Hz, C3), 121.9 (C8), 88.8 (ddd, $^1J_{CF}$ = 174.2 Hz, $^3J_{CF}$ = 7.9 Hz, $^3J_{CF}$ = 3.6 Hz, C1), 43.4 (dt, $^2J_{CF}$ = 26.3 Hz, $^2J_{CF}$ = 24.0 Hz, C2), 42.8 (ddd, $^2J_{CF}$ = 26.3 Hz, $^2J_{CF}$ = 24.7 Hz, $^4J_{CF}$ = 2.6 Hz, C4).

**GC-EI-MS:** Retention 10.05 min, (*m/z*) requires: [$(C_{17}H_{13}BrF_6)^+$] = 410.0099, (*m/z*) found: [$(C_{17}H_{13}BrF_6)^+$] = 410.0100.

**FT-IR** ($\tilde{\nu}$ = $cm^{-1}$)**:** 1621.5 (w), 1489.4 (w), 1417.7 (w), 1380.4 (w), 1344.5 (w), 1323.0 (s), 1262.7 (w), 1219.7 (w), 1165.1 (m), 1155.1 (m), 1125.0 (s), 1107.8 (s), 1034.6 (m), 1013.1 (m), 978.6 (m), 965.7 (w), 899.7 (w), 872.4 (w), 856.6 (w), 842.3 (m), 782.0 (s), 744.7 (w), 717.5 (w).

**4-(4-(4-Bromophenyl)-1,3,3-trifluorobutyl)phenyl trifluoromethanesulfonate (2w)**

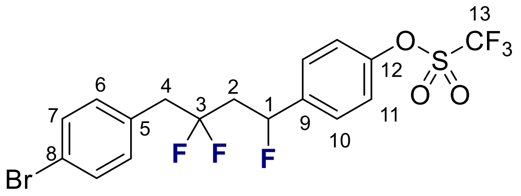


Compound **2w** was prepared according to general procedure **D** (amine:HF ratio = 1:6.5) using 4-(3-(4-bromophenyl)-3-hydroxycyclobutyl)phenyl trifluoromethane-sulfonate **1w** (90.3 mg, 0.200 mmol). The crude product was purified by flash column chromatography (5% EtOAc in cyclohexane) to yield the title compound as a colorless solid (80.7 mg, 0.164 mmol, 82%).

**R*f*** = 0.29 (5% EtOAc in cyclohexane).

**Melting Point:** 89-91 ˚C.

**$^{1}$H NMR** (500 MHz, $CDCl_3$): $\delta$ [ppm] = 7.50 – 7.45 (m, 2H, H-C7), 7.42 – 7.38 (m, 2H, H-C10), 7.32 – 7.28 (m, 2H, H-C11), 7.18 (d, $^{3}J_{HH}$ = 8.2 Hz, 2H, H-C6), 5.83 (ddd, $^{2}J_{HF}$ = 47.7 Hz, $^{3}J_{HH}$ = 9.4 Hz, $^{3}J_{HH}$ = 2.7 Hz, 1H, H-C1), 3.32 – 3.16 (m, 2H, H-C4), 2.58 – 2.40 (m, 1H, $H^{a}$-C2), 2.31 – 2.12 (m, 1H, $H^{b}$-C2).

**$^{19}$F NMR** (376 MHz, $CDCl_3$): $\delta$ [ppm] = -72.80 (s, 3F, F-C13), -92.85 (ddtdd, $^{2}J_{FF}$ = 248.6 Hz, $^{3}J_{FH}$ = 18.6 Hz, $^{3}J_{FH}$ = 15.0 Hz, $^{3}J_{FH}$ = 14.4 Hz, $^{4}J_{FF}$ = 3.8 Hz, 1F, $F^{a}$-C3), -96.46 (ddtdd, $^{2}J_{FF}$ = 248.7 Hz, $^{3}J_{FH}$ = 17.4 Hz, $^{3}J_{FH}$ = 15.8 Hz, $^{3}J_{FH}$ = 13.1 Hz, $^{4}J_{FF}$ = 7.9 Hz, 1F, $F^{b}$-C3), -175.42 (ddddd, $^{2}J_{FH}$ = 46.9 Hz, $^{3}J_{FH}$ = 34.3 Hz, $^{3}J_{FH}$ = 15.8 Hz, $^{4}J_{FF}$ = 7.7 Hz, $^{4}J_{FF}$ = 3.5 Hz, 1F, F-C1).

**$^{19}$F{$^{1}$H} NMR** (376 MHz, $CDCl_3$): $\delta$ [ppm] = -72.80 (s, 3F, F-C13), -92.85 (dd, $^{2}J_{FF}$ = 248.6 Hz, $^{4}J_{FF}$ = 3.9 Hz, 1F, $F^{a}$-C3), -96.46 (dd, $^{2}J_{FF}$ = 248.6 Hz, $^{4}J_{FF}$ = 7.9 Hz, 1F, $F^{b}$-C3), -175.42 (dd, $^{4}J_{FF}$ = 7.9 Hz, $^{4}J_{FF}$ = 3.9 Hz, 1F, F-C1).

**$^{13}$C{$^{1}$H} NMR** (126 MHz, $CDCl_3$): $\delta$ [ppm] = 149.6 (d, $^{5}J_{CF}$ = 2.1 Hz, C12), 139.7 (d, $^{2}J_{CF}$ = 20.5 Hz, C9), 132.3 (C6), 131.9 (C7), 131.8 (dd, $^{3}J_{CF}$ = 6.4 Hz, $^{3}J_{CF}$ = 2.8 Hz, C5), 127.5 (d, $^{3}J_{CF}$ = 7.1 Hz, C10), 122.2 (dd, $^{1}J_{CF}$ = 244.5 Hz, $^{1}J_{CF}$ = 243.1 Hz, C3), 122.0 – 121.8 (m, C8, C11), 118.9 (q, $^{1}J_{CF}$ = 320.9 Hz, C13), 88.5 (ddd, $^{1}J_{CF}$ = 174.1 Hz, $^{3}J_{CF}$ = 7.9 Hz, $^{3}J_{CF}$ = 3.6 Hz, C1), 43.4 (dt, $^{2}J_{CF}$ = 26.5 Hz, $^{2}J_{CF}$ = 24.2 Hz, C2), 42.9 (ddd, $^{2}J_{CF}$ = 26.2 Hz, $^{2}J_{CF}$ = 24.9 Hz, $^{4}J_{CF}$ = 2.7 Hz, C4).

**ESI-MS:** (*m/z*) requires: $[(C_{17}H_{13}BrF_6O_3SNa)^+]$ = 512.9565, (*m/z*) found: $[(C_{17}H_{13}BrF_6O_3SNa)^+]$ = 512.9565.

**FT-IR** ($\tilde{\nu}$ = $cm^{-1}$)**:** 1604.2 (w), 1506.7 (w), 1488.0 (w), 1419.1 (m), 1357.4 (w), 1340.2 (w), 1321.6 (w), 1294.3 (w), 1251.3 (w), 1201.0 (s), 1133.6 (s), 1106.3 (m), 1069.0 (m), 1030.3 (w), 1018.8 (w), 1011.61 (m), 964.3 (w), 892.5 (s), 871.0 (m), 836.6 (m), 815.0 (m), 780.6 (s), 743.3 (w), 730.4 (w), 714.6 (m).

**1-Bromo-4-(4-(4-chlorophenyl)-2,2,4-trifluoropentyl)benzene (2x)**

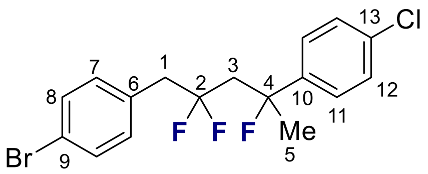


Compound **2x** was prepared according to modified general procedure **D** (amine:HF ratio = 1:5.5) using *cis*-1-(4-bromophenyl)-3-(4-chlorophenyl)-3-methylcyclobutan-1-ol *cis*-**1x** (70.3 mg, 0.200 mmol). The reaction time was elongated to 42 h. The crude product was purified by flash column chromatography (5-7.5% DCM in cyclohexane) to yield the title compound as a colorless solid (44.2 mg, 0.113 mmol, 57%).

**$R_f$** = 0.22 (10% DCM in cyclohexane).

**Melting Point:** 66-68 ˚C.

**$^1$H NMR** (599 MHz, $CDCl_3$): $\delta$[ppm] = 7.46 – 7.42 (m, 2H, H-C8), 7.36 – 7.32 (m, 2H, H-C12), 7.30 – 7.26 (m, 2H, H-C11), 7.15 – 7.09 (m, 2H, H-C7), 3.25 – 3.00 (m, 2H, H-C1), 2.57 – 2.36 (m, 2H, H-C3), 1.77 (dd, $^3J_{HF}$ = 22.9 Hz, 3H, H-C5).

**$^{19}$F NMR** (376 MHz, $CDCl_3$): $\delta$ [ppm] = -89.85 (ddqd, $^2J_{FF}$ = 250.8 Hz, $^3J_{HF}$ = 20.1 Hz, $^3J_{HF}$ = 15.2 Hz, $^4J_{FF}$ = 5.3 Hz, 1F, $F^a$-C2), -91.68 (dpd, $^2J_{FF}$ = 250.8 Hz, $^3J_{HF}$ = 16.0 Hz, $^4J_{FF}$ = 8.1 Hz, 1F, $F^b$-C2), -145.30 (hdd, $^2J_{FH}$ = 22.5 Hz, $^4J_{FF}$ = 8.2 Hz, $^4J_{FF}$ = 5.2 Hz, 1F, F-C4).

**$^{19}$F{$^1$H} NMR** (376 MHz, $CDCl_3$): $\delta$[ppm] = -89.85 (dd, $^2J_{FF}$ = 250.8 Hz, $^4J_{FF}$ = 5.2 Hz, 1F, $F^a$-C2), -91.68 (dd, $^2J_{FF}$ = 250.8 Hz, $^4J_{FF}$ = 8.2 Hz, 1F, $F^b$-C2), -145.30 (dd, $^4J_{FF}$ = 8.2 Hz, $^4J_{FF}$ = 5.2 Hz, 1F, F-C4).

**$^{13}$C{$^1$H} NMR** (151 MHz, $CDCl_3$): $\delta$[ppm] = 142.6 (d, $^2J_{CF}$ = 22.2 Hz, C10), 133.7 (d, $^5J_{CF}$ = 1.7 Hz, C13), 132.4 (C7), 132.0 (t, $^3J_{CF}$ = 4.3 Hz, C6), 131.7 (C8), 128.7 (d, $^4J_{CF}$ = 1.4 Hz, C12), 125.5 (d, $^3J_{CF}$ = 9.8 Hz, C11), 122.3 (t, $^1J_{CF}$ = 244.2 Hz, C2), 121.7 (C9), 95.1 (ddd, $^1J_{CF}$ = 175.7 Hz, $^3J_{CF}$ = 4.3 Hz, $^3J_{CF}$ = 2.9 Hz, C4), 46.8 (q, $^2J_{CF}$ = 24.4 Hz, C3), 43.2 (td, $^2J_{CF}$ = 25.3 Hz, $^4J_{CF}$ = 3.5 Hz, C1), 28.0 (dd, $^2J_{CF}$ = 24.8 Hz, $^4J_{CF}$ = 2.9 Hz, C5).

**GC-EI-MS:** Retention 10.27 min, (*m/z*) requires: [$(C_{17}H_{15}BrClF_3)^+$] = 389.9992, (*m/z*) found: [$(C_{17}H_{15}BrClF_3)^+$] = 389.9991.

**FT-IR** ($\tilde{\nu}$ = $cm^{-1}$)**:** 3003.3 (w), 1488.0 (m), 1457.9 (w), 1432.0 (w), 1399.0 (w), 1387.6 (w), 1366.0 (m), 1346.0 (w), 1308.6 (w), 1254.1 (w), 1211.1 (w), 1172.3 (m), 1147.9 (s), 1110.6 (m), 1090.5 (m), 1071.9 (m), 1059.0 (w), 1011.6 (s), 1004.4 (s), 939.9 (w), 888.2 (m), 861.0 (m), 835.1 (s), 777.7 (s), 760.5 (m), 727.5 (m), 714.6 (m).

**3-(4-(1,3,3-Trifluoro-4-phenylbutyl)phenyl)pyridine (2y)**

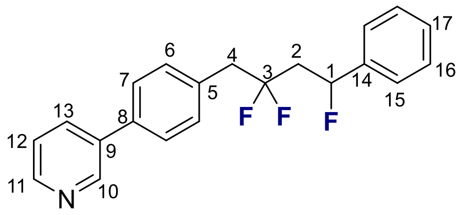


Compound **2y** was prepared according to general procedure **D** (amine:HF ratio = 1:5.5) using 3-phenyl-1-(4-(pyridin-3-yl)phenyl)cyclobutanol **1y** (60.2 mg, 0.200 mmol). The crude product was purified by flash column chromatography (0-5% EtOAc in DCM) to yield the title compound as a colorless solid (42.7 mg, 0.126 mmol, 63%).

**R*f*** = 0.32 (5% EtOAc in DCM).

**Melting Point:** 90-92 °C.

**$^1$H NMR** (500 MHz, CDCl$_3$): $\delta$ [ppm] = 8.86 (d, $^4J_{HH}$ = 2.5 Hz, 1H, H-C10), 8.60 (dd, $^3J_{HH}$ = 4.8 Hz, $^4J_{HH}$ = 1.6 Hz, 1H, H-C11), 7.87 (ddd, $^3J_{HH}$ = 7.9 Hz, $^4J_{HH}$ = 2.4 Hz, $^4J_{HH}$ = 1.6 Hz, 1H, H-C13), 7.56 (d, 2H, $^3J_{HH}$ = 8.3 Hz, H-C7), 7.43 (d, $^3J_{HH}$ = 8.1 Hz, 2H, H-C6), 7.40 – 7.33 (m, 6H, H-C12, H-C15, H-C16, H-C17), 5.83 (dd, $^2J_{HF}$ = 48.3 Hz, $^3J_{HH}$ = 9.6 Hz, $^4J_{FH}$ = 2.3 Hz, 1H, H-C1), 3.44 – 3.27 (m, 2H, H-C4), 2.59 (dtdd, $^3J_{HF}$ = 19.4 Hz, $^2J_{HH}$ = $^3J_{HF}$ = 15.6 Hz, $^3J_{HF}$ = 12.9 Hz, $^3J_{HH}$ = 9.6 Hz, 1H, H$^a$-C2), 2.28 (dtdd, $^3J_{HF}$ = 34.3 Hz, $^2J_{HH}$ = $^3J_{HF}$ = 15.3 Hz, $^3J_{HF}$ = 11.9 Hz, $^3J_{HH}$ = 2.6 Hz, 1H, H$^b$-C2).

**$^{19}$F NMR** (376 MHz, CDCl$_3$): $\delta$ [ppm] = -92.50 (ddtdd, $^2J_{FF}$ = 247.6 Hz, $^3J_{FH}$ = 18.0 Hz, $^3J_{FH}$ = 15.7 Hz, $^3J_{FH}$ = 12.4 Hz, $^4J_{FF}$ = 4.0 Hz, 1F, F$^a$-C3), -96.16 (ddtdd, $^2J_{FF}$ = 247.7 Hz, $^3J_{FH}$ =20.3 Hz, $^3J_{FH}$ =15.6 Hz, $^3J_{FH}$ =13.2 Hz, $^4J_{FF}$ = 7.6 Hz, 1F, F$^b$-C3), -173.66 (ddddd, $^2J_{FH}$ = 47.9 Hz, $^3J_{FH}$ = 35.7 Hz, $^3J_{FH}$ =15.8 Hz, $^4J_{FF}$ = 7.3 Hz, $^4J_{FF}$ = 3.9 Hz, 1F, F-C1).

**$^{19}$F{$^1$H} NMR** (376 MHz, CDCl$_3$): $\delta$ [ppm] = -92.50 (dd, $^2J_{FF}$ = 247.6 Hz, $^4J_{FF}$ = 4.1 Hz, 1F, F$^a$-C3), -96.16 (dd, $^2J_{FF}$ = 247.7 Hz, $^4J_{FF}$ = 7.7 Hz, 1F, F$^b$-C3), -173.64 (dd, $^4J_{FF}$ = 7.7 Hz, $^4J_{FF}$ = 4.0 Hz, 1F, F-C1).

**$^{13}$C{$^1$H} NMR** (126 MHz, CDCl$_3$): $\delta$ [ppm] = 148.7 (C11), 148.4 (C10), 139.2 (d, $^2J_{CF}$ = 19.8 Hz, C14), 137.1 (C8), 136.2 (C9), 134.4 (C13), 132.9 (dd, $^3J_{CF}$ = 6.4 Hz, $^3J_{CF}$ = 2.6 Hz, C5), 131.4 (C6), 128.9 (d, $^4J_{CF}$ = 1.8 Hz, C16), 128.8 (C17), 127.3 (C7), 125.6 (d, $^3J_{CF}$ = 6.6 Hz, C15), 123.7 (C12), 123.3 (dd, $^1J_{CF}$ = 273.8 Hz, $^1J_{CF}$ = 242.9 Hz, C3), 89.6 (ddd, $^3J_{CF}$ = 172.0 Hz, $^3J_{CF}$ = 8.1 Hz, $^3J_{CF}$ = 3.7 Hz, C1), 43.5 (q, $^2J_{CF}$ = 24.7 Hz, C2), 43.0 (td, $^2J_{CF}$ = 26.7 Hz, $^4J_{CF}$ = 2.4 Hz, C4).

**ESI-MS:** (*m/z*) requires: [($C_{21}H_{19}F_3N$)$^+$] = 342.1464, (*m/z*) found: [($C_{21}H_{19}F_3N$)$^+$] = 342.1463.

**FT-IR** ($\tilde{\nu}$ = cm$^{-1}$)**:** 3034.84 (broad), 1609.97 (w), 1495.18 (w), 1473.66 (m), 1457.87 (w), 1432.04 (w), 1361.73 (m), 1327.30 (w), 1285.39 (w), 1211.07 (m), 1156.54 (m), 1133.58 (m), 1116.36 (m), 1087.67 (m), 1060.40 (m),1043 (m), 1023.09 (m), 1000.14 (m), 957.09 (m), 919.78 (m), 886.78 (m), 856.64 (m), 839.41 (m), 806.42 (m), 782.02 (m),767.68 (s), 749.03 (m), 698.80 (s), 683.02 (m), 667.24 (m), 621.32 (m), 611.27 (m).

## Synthesis of 3,3-Difluoro-1-phenyl-1,2,3,4-tetrahydronaphthalene Derivatives

### General Procedure E (One Pot Procedure)

The 1,1,3-trifluorobutane derivative **2** was prepared according to general procedure **D** on a 0.200 mmol-scale with the indicated amine•HF mixture. Instead of quenching the reaction after 18 h, HFIP (2.0 mL) was added and the reaction was stirred for another 24 h at room temperature. The reaction mixture was diluted with DCM (2 mL) and poured in saturated aqueous $NaHCO_3$ (100 mL). The aqueous layer was extracted with DCM (3x 30 mL). The combined organic layers were dried over $Na_2SO_4$ and the solvent was removed under reduced pressure. The crude product was purified by flash column chromatography.

***Caution:*** *Olah's reagent is highly toxic and corrosive. Direct exposure should be avoided. In the case of skin exposure, immediate treatment of the affected skin area with calcium gluconate gel is necessary to prevent serious chemical burns.*

### General Procedure F (Step-wise Procedure)

Isolated 1,1,3-trifluorobutane derivative **2** (0.200 mmol, 1.0 eq.) was dissolved in $CHCl_3$ (1.0 mL). Olah´s reagent (1.0 mL) was added and the reaction was stirred at the indicated temperature for the indicated time. The reaction mixture was diluted with DCM (2 mL) and poured in saturated aqueous $NaHCO_3$ (200 mL). The aqueous layer was extracted with DCM (3x 30 mL). The combined organic layers were dried over $Na_2SO_4$ and the solvent was removed under reduced pressure. The crude product was purified by flash column chromatography.

***Caution:*** *Olah's reagent is highly toxic and corrosive. Direct exposure should be avoided. In the case of skin exposure, immediate treatment of the affected skin area with calcium gluconate gel is necessary to prevent serious chemical burns.*

**3,3-Difluoro-1-phenyl-1,2,3,4-tetrahydronaphthalene (3a)**

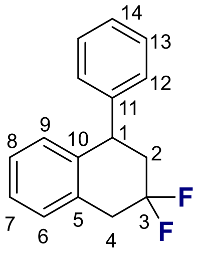


Compound **3a** was prepared according to general procedure **E** (amine:HF 1:4.5) using 1,3-diphenylcyclobutan-1-ol **1a** (44.9 mg, 0.200 mmol). The crude product was purified by flash column chromatography (4-7% DCM in cyclohexane) to yield the title compound as a colorless solid (27.5 mg, 0.113 mmol, 57%).

$\mathbf{R}_f$ = 0.42 (10% DCM in cyclohexane).

**Melting Point:** 53-55 °C.

**$^{1}$H NMR** (599 MHz, $CDCl_3$): $\delta$ [ppm] = 7.38 – 7.34 (m, 2H, H-C13), 7.32 – 7.28 (m, 1H, H-C14), 7.23 – 7.20 (m, 2H, H-C12), 7.20 – 7.18 (m, 1H, H-C8), 7.17 – 7.14 (m, 1H, H-C6), 7.12 – 7.07 (m, 1H, H-C7), 6.81 (dt, $^{3}J_{HH}$ = 8.0 Hz, $J_{HH}$= 1.1 Hz, 1H, H-C9), 4.35 (dd, $^{3}J_{HH}$ = 12.1 Hz, $^{3}J_{HH}$ = 5.9 Hz, 1H, H-C1), 3.49 – 3.33 (m, 2H, H-C4), 2.33 (dtdd, $^{2}J_{HH}$ = 14.0 Hz, $^{3}J_{HF}$ = 7.8 Hz, $^{3}J_{HH}$ = 5.8 Hz, $J_{HH}$ = 2.8 Hz, 1H, $H^{a}$-C2), 2.25 (dddd, $^{3}J_{HF}$ = 31.9 Hz, $^{2}J_{HH}$ = 14.0 Hz, $^{3}J_{HH}$ = 12.0 Hz, $^{3}J_{HF}$ = 2.8 Hz, 1H, $H^{b}$-C2).

**$^{19}$F NMR** (376 MHz, $CDCl_3$): $\delta$ [ppm] = -91.22 (ddddd, $^{2}J_{FF}$ = 237.6 Hz, $^{3}J_{FH}$ = 10.4 Hz, $^{3}J_{FH}$ = 8.5 Hz, $^{3}J_{FH}$ = 5.1 Hz, $^{3}J_{FH}$ = 3.1 Hz, 1F, $F^{a}$-C3), -98.73 (ddddd, $^{2}J_{FF}$ = 237.2 Hz, $^{3}J_{FH}$ = 32.3 Hz, $^{3}J_{FH}$ = 28.8 Hz, $^{3}J_{FH}$ = 16.9 Hz, $^{3}J_{FH}$ = 8.1 Hz, 1F, $F^{b}$-C3).

**$^{19}$F{$^{1}$H} NMR** (376 MHz, $CDCl_3$): $\delta$ [ppm] = -91.22 (d, $^{2}J_{FF}$ = 237.2 Hz, 1F, $F^{a}$-C3), -98.73 (dd, $^{2}J_{FF}$ = 237.2 Hz, $F^{b}$-C3).

**$^{13}$C{$^{1}$H} NMR** (151 MHz, $CDCl_3$): $\delta$ [ppm] = 144.0 (C11), 137.7 (d, $^{4}J_{CF}$ = 1.7 Hz, C10), 132.0 (d, $^{3}J_{CF}$ = 10.2 Hz, C5), 129.2 (d, $^{5}J_{CF}$ = 1.1 Hz, C9), 129.1 (d, $^{4}J_{CF}$ = 1.8 Hz, C6), 129.0 (C12, C13), 127.1 (C14), 127.0 (C7), 126.9 (C8), 122.5 (dd, $^{1}J_{CF}$ = 241.9 Hz, $^{1}J_{CF}$ = 239.1 Hz, C3), 44.4 (dd, $^{3}J_{CF}$ = 8.2 Hz, $^{3}J_{CF}$ = 2.2 Hz, C1), 40.4 (dd, $^{2}J_{CF}$ = 24.8 Hz, $^{2}J_{CF}$ = 22.0 Hz, C2), 38.5 (dd, $^{2}J_{CF}$ = 27.4 Hz, $^{2}J_{CF}$ = 26.0 Hz, C4).

**GC-EI-MS:** Retention 9.02 min, (*m/z*) requires: $[(C_{16}H_{14}F_2)^+]$ = 244.1058, (*m/z*) found: $[(C_{16}H_{14}F_2)^+]$ = 244.1059.

**FT-IR** ($\tilde{\nu}$ = cm$^{-1}$)**:** 3026.2 (w), 1598.5 (w), 1489.4 (m), 1455.0 (m), 1427.7 (w), 1366.0 (m), 1358.9 (m), 1318.7 (m), 1291.4 (w), 1277.1 (m), 1267.0 (m), 1241.2 (w), 1122.1 (m), 1092.0 (m), 1073.3 (m), 1059.0 (m), 1041.8 (s), 1031.7 (m), 1020.2 (m), 1000.1 (w), 957.1 (m), 916.9 (w), 876.7 (m), 861.0 (m), 835.1 (m), 800.7 (w), 783.5 (m), 754.8 (s), 737.6 (s), 700.2 (s).

**3,3,7-Trifluoro-1-(4-fluorophenyl)-1,2,3,4-tetrahydronaphthalene (3b)**

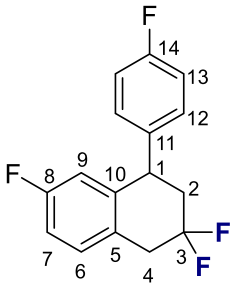


Compound **3b** was prepared according to general procedure **E** (amine:HF 1:4.5) using 1,3-bis(4-fluorophenyl)-cyclobutan-1-ol **1b** (52.0 mg, 0.200 mmol). The crude product was purified by flash column chromatography (2% DCM in *n*-pentane) to yield the title compound as a colorless oil (22.0 mg, 0.078 mmol, 39%).

$\mathbf{R}_f$ = 0.42 (5% DCM in *n*-pentane).

**$^1$H NMR** (500 MHz, $CDCl_3$): $\delta$[ppm] = 7.18 – 7.13 (m, 2H, H-C12), 7.14 – 7.07 (m, 1H, H-C6), 7.09 – 7.03 (m, 2H, H-C13), 6.91 (td, $^3J_{HH}$ = $^3J_{HF}$ = 8.7 Hz, $^4J_{HH}$ = 3.1 Hz, 1H, H-C7), 6.47 (dd, $^3J_{FH}$ = 9.6 Hz, $^4J_{HH}$ = 2.9 Hz, 1H, C9), 4.29 (dd, $^3J_{HH}$ = 12.1 Hz, $^3J_{HH}$ = 5.9 Hz, 1H, H-C1), 3.44 – 3.26 (m, 2H, H-C4), 2.55 (dtdd, $^2J_{HH}$ = 13.8 Hz, $^3J_{HF}$ = 8.2 Hz, $^3J_{HH}$ = 6.0 Hz, $J_{HH}$ = 2.1 Hz, 1H, H$^a$-C2), 2.25 (dddd, $^3J_{HF}$ = 32.6 Hz, $^2J_{HH}$ = 14.8 Hz, $^3J_{HH}$ = 12.0 Hz, $^3J_{HF}$ = 3.7 Hz, 1H, H$^b$-C2).

**$^{19}$F NMR** (376 MHz, $CDCl_3$): $\delta$ [ppm] = -91.80 (ddtdd, $^2J_{FF}$ = 238.2 Hz, $^3J_{FH}$ = 10.6 Hz, $^3J_{FH}$ = 8.2 Hz, $^3J_{FH}$ = 5.7 Hz, $J_{FH}$ = 2.7 Hz, 1F, F$^a$-C3), -98.94 (ddddd, $^2J_{FF}$ = 238.7 Hz, $^3J_{FH}$ = 37.2 Hz, $^3J_{FH}$ = 25.3 Hz, $^3J_{FH}$ = 14.4 Hz, $^3J_{FH}$ = 7.9 Hz, 1F, F$^b$-C3), -115.19 – -115.31 (m, 2F, F-C8, F-C14).

**$^{19}$F{$^1$H} NMR** (376 MHz, $CDCl_3$): $\delta$ [ppm] = -91.83 (d, $^2J_{FF}$ = 238.4 Hz, 1F, F$^a$-C3), -98.98 (d, $^2J_{FF}$ = 238.4 Hz, 1F, F$^b$-C3), -115.23 (s, 1F, F-C8 or F-C14), -115.28 (s, 1F, F-C8 or F-C14).

**$^{13}$C{$^1$H} NMR** (126 MHz, $CDCl_3$): $\delta$ [ppm] = 162.1 (d, $^1J_{CF}$ = 245.9 Hz, C8), 161.8 (d, $^1J_{CF}$ = 245.3 Hz, C14), 139.8 (d, $^3J_{CF}$ = 8.3 Hz, C10), 139.1 (d, $^4J_{CF}$ = 3.3 Hz, C11), 130.7 (dd, $^3J_{CF}$ = 8.1 Hz, $^4J_{CF}$ = 2.0 Hz, C6), 130.3 (d, $^3J_{CF}$ = 8.0 Hz, C12), 127.7 (dd, $^3J_{CF}$ = 10.5 Hz, $^4J_{CF}$ = 2.7 Hz, C5), 122.4 (dd, $^1J_{CF}$ = 242.2 Hz, $^1J_{CF}$ = 239.1 Hz, C3), 116.0 (d, $^2J_{CF}$ = 21.4 Hz, C13), 115.7 (dd, $^2J_{CF}$ = 22.1 Hz, $^5J_{CF}$ = 1.2 Hz, C9), 114.5 (dd, $^2J_{CF}$ = 21.8 Hz, $^5J_{CF}$ = 1.0 Hz, C7), 43.9 (d, $^3J_{CF}$ = 8.4 Hz, C1), 40.3 (dd, $^2J_{CF}$ = 25.0 Hz, $^2J_{CF}$ = 24.5 Hz, C2), 38.0 (dd, $^2J_{CF}$ = 27.4 Hz, $^2J_{CF}$ = 26.3 Hz, C4).

**GC-EI-MS:** Retention 9.00 min, (*m/z*) requires: $[(C_{16}H_{12}F_4)^+]$ = 280.0869, (*m/z*) found: $[(C_{16}H_{12}F_4)^+]$ = 280.0867.

**FT-IR** ($\tilde{\nu}$ = cm$^{-1}$)**:** 1492.3 (m), 1430.6 (m), 1386.1 (m), 1275.6 (m), 1267.6 (m), 1213.9 (m), 1192.4 (s), 1119.2 (m), 1083.4 (s), 1051.8 (s), 1030.3 (m), 1013.1 (m), 882.5 (m), 871.0 (m), 850.9 (s), 809.3 (s), 764.8 (s), 753.3 (s), 717.5 (s), 698.8 (s), 651.5 (s).

**7-Chloro-1-(4-chlorophenyl)-3,3-difluoro-1,2,3,4-tetrahydronaphthalene (3c)**

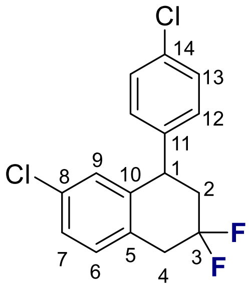

Compound **3c** was prepared according to general procedure **E** (amine:HF 1:5.5) using 1,3-bis(4-chlorophenyl)-cyclobutan-1-ol ***major*-1c** (58.6 mg, 0.200 mmol). The crude product was purified by flash column chromatography (4-7% DCM in cyclohexane) to yield the title compound as a yellow oil (43.7 mg, 0.139 mmol, 70%).

$\mathbf{R}_f$ = 0.32 (10% DCM in cyclohexane).

**$^1$H NMR** (500 MHz, $CDCl_3$): $\delta$ [ppm] = 7.36 – 7.32 (m, 2H, H-C13), 7.18 (ddd, $^3J_{HH}$ = 8.3 Hz, $^4J_{HH}$ = 2.2 Hz, $J_{HH}$ = 0.9 Hz, 1H, H-C7), 7.14 – 7.10 (m, 2H, H-C12), 7.08 (d, $^3J_{HH}$ = 8.2 Hz, 1H, H-C6), 6.75 (dd, $^4J_{HH}$ = 2.3 Hz, $J_{HH}$ = 1.0 Hz, 1H, H-C9), 4.28 (dd, $^3J_{HH}$ = 12.0 Hz, $^3J_{HH}$ = 6.0 Hz, 1H, H-C1), 3.41 – 3.25 (m, 2H, H-C4), 2.54 (dtdd, $^2J_{HH}$ = 14.0 Hz, $^3J_{HF}$ = 8.2 Hz, $^3J_{HH}$ = 6.6 Hz, $J_{HH}$ = 1.7 Hz, 1H, $H^a$-C2), 2.25 (dddd, $^3J_{HF}$ = 31.6 Hz, $^2J_{HH}$ = 13.7 Hz, $^3J_{HH}$ = 11.8 Hz, $^3J_{HF}$ = 2.8 Hz, 1H, $H^b$-C2).

**$^{19}$F NMR** (376 MHz, $CDCl_3$): $\delta$ [ppm] = -91.92 (dddddd, $^2J_{FF}$ = 239.1 Hz, $^3J_{FH}$ = 10.2 Hz, $^3J_{FH}$ = 8.2 Hz, $^3J_{FH}$ = 5.9 Hz, $^3J_{FH}$ = 2.3 Hz, $J_{FH}$ = 1.5 Hz, 1F, $F^a$-C3), -98.80 (ddddd, $^2J_{FF}$ = 239.1 Hz, $^3J_{FH}$ = 31.5 Hz, $^3J_{FH}$ = 26.7 Hz, $^3J_{FH}$ = 18.8 Hz, $^3J_{FH}$ = 7.8 Hz, 1F, $F^b$-C3).

**$^{19}$F{$^1$H} NMR** (376 MHz, $CDCl_3$): $\delta$ [ppm] = -91.92 (d, $^2J_{FF}$ = 239.1 Hz, 1F, $F^a$-C3), -98.80 (d, $^2J_{FF}$ = 239.1 Hz, $F^b$-C3).

**$^{13}$C{$^1$H} NMR** (126 MHz, $CDCl_3$): $\delta$ [ppm] = 141.8 (C11), 139.1 (d, $^4J_{CF}$ = 1.7 Hz, C10), 133.3 (C14), 133.0 (C8), 130.7 – 130.5 (m, C5), 130.6 (d, $^4J_{CF}$ = 1.7 Hz, C6), 130.2 (C12), 129.4 (C13), 129.1 (d, $^5J_{CF}$ = 1.1 Hz, C9), 127.5 (C7), 122.1 (dd, $^1J_{CF}$ = 242.4 Hz, $^1J_{CF}$ = 239.3 Hz, C3), 43.9 (dd, $^3J_{CF}$ = 8.3 Hz, $^3J_{CF}$ = 2.4 Hz, C1), 40.2 (dd, $^2J_{CF}$ = 24.8 Hz, $^2J_{CF}$ = 22.5 Hz, C2), 40.2 (t, $^2J_{CF}$ = 27.4 Hz, C4).

**GC-EI-MS:** Retention 10.52 min, (*m/z*) requires: [$(C_{16}H_{12}Cl_2F_2)^+$] = 312.0279, (*m/z*) found: [$(C_{16}H_{12}Cl_2F_2)^+$] = 312.0279.

**FT-IR** ($\tilde{\nu}$ = $cm^{-1}$)**:** 1597.1 (w), 1488.0 (m), 1446.4 (w), 1424.9 (w), 1406.2 (w), 1360.3 (w), 1310.1 (w), 1265.6 (m), 1236.9 (w), 1202.5 (w), 1178.1 (w), 1129.3 (m), 1084.8 (s), 1066.1 (s), 1031.7 (s), 1014.5 (m), 958.5 (m), 882.5 (m), 848.0 (w), 829.4 (m), 802.1 (s), 763.4 (w), 740.4 (w), 721.8 (w), 711.7 (w), 703.1 (m).

**7-Bromo-1-(4-bromophenyl)-3,3-difluoro-1,2,3,4-tetrahydronaphthalene (3d)**

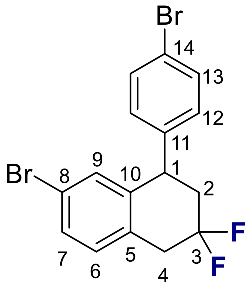


Compound **3d** was prepared according to general procedure **E** (amine:HF 1:5.5) using 1,3-bis(4-bromophenyl)cyclobutan-1-ol **1d** (76.4 mg, 0.200 mmol). The crude product was purified by flash column chromatography (4-7% DCM in cyclohexane) to yield the title compound as a colorless oil (60.9 mg, 0.151 mmol, 76%).

**$R_f$** = 0.40 (10% DCM in cyclohexane).

**$^{1}$H NMR** (500 MHz, $CDCl_3$): $\delta$ [ppm] = 7.52 – 7.48 (m, 2H, H-C13), 7.33 (ddd, $^{3}J_{HH}$ = 8.2 Hz, $^{4}J_{HH}$ = 2.1 Hz, $J_{HH}$ = 0.9 Hz, 1H, H-C7), 7.09 – 7.04 (m, 2H, H-C12), 7.02 (dd, $^{3}J_{HH}$ = 8.2 Hz, $J_{HH}$ = 1.0 Hz, 1H, H-C6), 6.91 (dd, $^{4}J_{HH}$ = 2.1 Hz, $J_{HH}$ = 1.1 Hz, 1H, H-C9), 4.27 (dd, $^{3}J_{HH}$ = 11.9 Hz, $^{3}J_{HH}$ = 5.9 Hz, 1H, H-C1), 3.39 – 3.26 (m, 2H, H-C4), 2.54 (dtdd, $^{2}J_{HH}$ = 13.9 Hz, $^{3}J_{HF}$ = 8.4 Hz, $^{3}J_{HH}$ = 6.0 Hz, $J_{HH}$ = 2.3 Hz, 1H, $H^{a}$-C2), 2.21 (dddd, $^{3}J_{HF}$ = 31.6 Hz, $^{2}J_{HH}$ = 13.7 Hz, $^{3}J_{HH}$ = 11.8 Hz, $^{3}J_{HF}$ = 2.8 Hz, 1H, $H^{b}$-C2).

**$^{19}$F NMR** (376 MHz, $CDCl_3$): $\delta$ [ppm] = -91.96 (dddddd, $^{2}J_{FF}$ = 239.2 Hz, $^{3}J_{FH}$ = 10.6 Hz, $^{3}J_{FH}$ = 8.2 Hz, $^{3}J_{FH}$ = 6.0 Hz, $^{3}J_{FH}$ = 2.7 Hz, $J_{FH}$ = 1.6 Hz, 1F, $F^{a}$-C3), -98.78 (ddddd, $^{2}J_{FF}$ = 239.3 Hz, $^{3}J_{FH}$ = 31.5 Hz, $^{3}J_{FH}$ = 25.7 Hz, $^{3}J_{FH}$ = 19.2 Hz, $^{3}J_{FH}$ = 7.8 Hz, 1F, $F^{b}$-C3).

**$^{19}$F{$^{1}$H} NMR** (376 MHz, $CDCl_3$): $\delta$ [ppm] = -91.96 (d, $^{2}J_{FF}$ = 239.2 Hz, 1F, $F^{a}$-C3), -98.78 (d, $^{2}J_{FF}$ = 239.3 Hz, $F^{b}$-C3).

**$^{13}$C{$^{1}$H} NMR** (126 MHz, $CDCl_3$): $\delta$ [ppm] = 142.3 (C11), 139.4 (d, $^{4}J_{CF}$ = 1.6 Hz, C10), 132.3 (C13), 132.0 (d, $^{5}J_{CF}$ = 1.1 Hz, C9), 131.2 (d, $^{3}J_{CF}$ = 10.4 Hz, C5), 130.9 (d, $^{4}J_{CF}$ = 1.8 Hz, C6), 130.5 (C12), 130.4 (C7), 122.0 (dd, $^{1}J_{CF}$ = 242.5 Hz, $^{1}J_{CF}$ = 239.2 Hz, C3), 121.4 (C8), 121.0 (C14), 43.9 (dd, $^{3}J_{CF}$ = 8.2 Hz, $^{3}J_{CF}$ = 2.4 Hz, C1), 40.2 (dd, $^{2}J_{CF}$ = 24.8 Hz, $^{2}J_{CF}$ = 22.5 Hz, C2), 38.2 (dd, $^{2}J_{CF}$ = 27.6 Hz, $^{2}J_{CF}$ = 26.5 Hz, C4).

**GC-EI-MS:** Retention 11.25 min, (*m/z*) requires: [$(C_{16}H_{12}Br_2F_2)^+$] = 401.9249, (*m/z*) found: [$(C_{16}H_{12}Br_2F_2)^+$] = 401.9248.

**FT-IR** ($\tilde{\nu}$ = $cm^{-1}$)**:** 1591.3 (w), 1485.1 (m), 1445.0 (w), 1423.4 (w), 1401.9 (w), 1360.3 (w), 1308.6 (w), 1267.0 (m), 1236.9 (w), 1202.5 (w), 1175.2 (w), 1129.3 (m), 1093.4 (m), 1080.5 (m), 1066.1 (s), 1028.8 (m), 1010.2 (s), 957.1 (m), 875.3 (m), 845.2 (w), 823.6 (m), 807.9 (m), 797.8 (s), 780.6 (m), 754.8 (w), 716.0 (w), 708.9 (w).

**3,3-Difluoro-7-(trifluoromethyl)-1-(4-(trifluoromethyl)phenyl)-1,2,3,4-tetrahydronaphthalene (3e)**

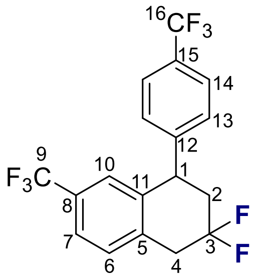

Compound **3e** was prepared according to modified general procedure **F** using 4,4'-(1,3,3-trifluorobutane-1,4-diyl)bis((trifluoromethyl)benzene) **2e** (80.0 mg, 0.200 mmol). The reaction was stirred for 72 h at 40 ˚C. The crude product was purified by flash column chromatography (100% *n*-pentane) to yield the title compound as a colorless solid (60.7 mg, 0.160 mmol, 80%).

**$R_f$** = 0.33 (100% *n*-pentane).

**Melting Point:** 72-74 °C.

**$^1$H NMR** (500 MHz, $CDCl_3$): $\delta$ [ppm] = 7.64 (d, $^3J_{HH}$ = 8.1 Hz, 2H, H-C14), 7.48 (d, $^3J_{HH}$ = 7.9 Hz, 1H, H-C7), 7.32 – 7.29 (m, 3H, H-C6, H-C13), 7.04 – 7.03 (m, 1H, H-C10), 4.45 (dd, $^3J_{HH}$ = 11.7 Hz, $^3J_{HH}$ = 6.1 Hz, 1H, H-C1), 3.48 – 3.41 (m, 2H, H-C4), 2.62 (dtdd, $^2J_{HH}$ = 13.5 Hz, $^3J_{HF}$ = 8.1 Hz, $^3J_{HH}$ = 6.1 Hz, $J_{HH}$ = 2.1 Hz, 1H, $H^a$-C2), 2.29 (dddd, $^3J_{FH}$ = 31.0 Hz, $^2J_{HH}$ = 14.2 Hz, $^3J_{HH}$ = 11.6 Hz, $^3J_{FH}$ = 2.8 Hz, 1H, $H^b$-C2).

**$^{19}$F NMR** (470 MHz, $CDCl_3$): $\delta$ [ppm] = -62.58 (s, 3F, F-C9 or F-C16), -62.67 (s, 3F, F-C9 or F-C16), -92.37 (ddddd, $^2J_{FF}$ = 241.0 Hz, $^3J_{FH}$ = 11.2 Hz, $^3J_{FH}$ = 9.3 Hz, $^3J_{FH}$ = 5.4 Hz, $^3J_{FH}$ = 2.6 Hz, 1F, $F^a$-C3), -98.53 (ddddd, $^2J_{FF}$ = 240.9 Hz, $^3J_{FH}$ = 30.9 Hz, $^3J_{FH}$ = 24.8 Hz, $^3J_{FH}$ = 17.2 Hz, $^3J_{FH}$ = 7.7 Hz, 1F, $F^b$-C3).

**$^{19}$F{$^1$H} NMR** (470 MHz, $CDCl_3$): $\delta$ [ppm] = -62.58 (s, 3F, F-C9 or F-C16), -62.67 (s, 3F, F-C9 or F-C16), -92.34 (d, $^2J_{FF}$ = 240.5 Hz, 1F, $F^a$-C3), -98.54 (d, $^2J_{FF}$ = 240.4 Hz, 1F, $F^b$-C3).

**$^{13}$C{$^1$H} NMR** (126 MHz, $CDCl_3$): $\delta$ [ppm] = 147.2 (C12), 137.5 (d, $^4J_{CF}$ = 1.5 Hz, C11), 136.3 (d, $^3J_{CF}$ = 10.1 Hz, C5), 130.0 (d, $^4J_{CF}$ = 1.7 Hz, C6), 129.9 (q, $^2J_{CF}$ = 32.0 Hz, C8), 129.7 (q, $^2J_{CF}$ = 32.7 Hz, C15), 129.2 (C13), 126.3 (q, $^3J_{CF}$ = 3.7 Hz, H-C14), 126.1 (q, $^3J_{CF}$ = 3.6 Hz, C10), 124.2 (q, $^1J_{CF}$ = 272.1 Hz, C9), 124.1 (q, $^3J_{CF}$ = 3.8 Hz, C7), 123.0 (q, $^1J_{CF}$ = 272.1 Hz, C16), 121.7 (dd, $^1J_{CF}$ = 242.8 Hz, $^1J_{CF}$ = 239.3 Hz, C3), 44.4 (dd, $^3J_{CF}$ = 8.1 Hz, $^3J_{CF}$ = 2.6 Hz, C1), 40.3 (t, $^2J_{CF}$ = 22.5 Hz, C2), 38.6 (t, $^2J_{CF}$ = 27.3 Hz, C4).

**GC-EI-MS:** Retention 8.08 min, (*m/z*) requires: $[(C_{18}H_{12}F_8)^+]$ = 380.0806, (*m/z*) found: $[(C_{18}H_{12}F_8)^+]$ = 380.0805.

**FT-IR** ($\tilde{\nu}$ = cm$^{-1}$)**:** 1620.0 (m), 1423.4 (w), 1367.5 (m), 1324.4 (s), 1268.5 (w), 1244.1 (w), 1156.5 (m), 1109.2 (s), 1096.3 (m), 1066.1 (w), 1034.6 (w), 1018.8 (w), 960.0 (m), 848.0 (m), 838.0 (m), 819.3 (m), 812.2 (m), 793.5 (m), 718.9 (m), 734.7 (w), 668.7 (m), 608.4 (m).

**3,3,7-Trifluoro-1-phenyl-1,2,3,4-tetrahydronaphthalene (3f)**

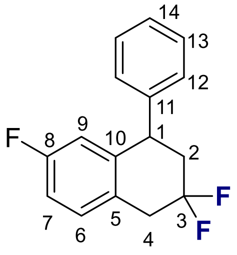


Compound **3f** was prepared according to general procedure **E** (amine:HF 1:4.5) using 1-(4-fluorophenyl)-3-phenylcyclobutan-1-ol **1f** (48.5 mg, 0.200 mmol). The crude product was purified by flash column chromatography (4-7% DCM in cyclohexane) to yield the title compound as a yellow oil (26.5 mg, 0.101 mmol, 51%).

**R*f*** = 0.38 (10% DCM in cyclohexane).

**$^{1}$H NMR** (599 MHz, $CDCl_3$): $\delta$ [ppm] = 7.40 – 7.35 (m, 2H, H-C13), 7.33 – 7.29 (m, 1H, H-C14), 7.22 – 7.18 (m, 2H, H-C12), 7.11 (dd, $^{3}J_{HH}$ = 8.7 Hz, $^{4}J_{HF}$ = 5.7 Hz, 1H, H-C6), 6.90 (td, $^{3}J_{HH}$ = $^{3}J_{HF}$ = 8.3 Hz, $^{4}J_{HH}$ = 2.7 Hz, 1H, H-C7), 6.78 (ddd, $^{3}J_{HF}$ = 10.1 Hz, $^{4}J_{HH}$ = 2.8 Hz, $J_{HH}$ = 1.0 Hz, 1H, H-C9), 4.29 (dd, $^{3}J_{HH}$ = 12.2 Hz, $^{3}J_{HH}$ = 5.9 Hz, 1H, H-C1), 3.42 – 3.31 (m, 2H, H-C4), 2.58 (dtdd, $^{2}J_{HH}$ = 13.8 Hz, $^{3}J_{HF}$ = 8.3 Hz, $^{3}J_{HH}$ = 5.9 Hz, $J_{HH}$ = 2.3 Hz, 1H, $H^{a}$-C2), 2.28 (dddd, $^{3}J_{HF}$ = 32.4 Hz, $^{2}J_{HH}$ = 14.5 Hz, $^{3}J_{HH}$ = 12.3 Hz, $^{3}J_{HF}$ = 2.9 Hz, 1H, $H^{b}$-C2).

**$^{19}$F NMR** (376 MHz, $CDCl_3$): $\delta$ [ppm] = -91.65 (dddddd, $^{2}J_{FF}$ = 237.9 Hz, $^{3}J_{FH}$ = 10.2 Hz, $^{3}J_{FH}$ = 8.3 Hz, $^{3}J_{FH}$ = 5.7 Hz, $^{3}J_{FH}$ = 2.7 Hz, $J_{FH}$ = 1.9 Hz, 1F, $F^{a}$-C3), -99.02 (ddddd, $^{2}J_{FF}$ = 237.8 Hz, $^{3}J_{FH}$ = 32.3 Hz, $^{3}J_{FH}$ = 27.1 Hz, $^{3}J_{FH}$ = 18.7 Hz, $^{3}J_{FH}$ = 8.1 Hz, 1F, $F^{b}$-C3), -115.41 (dddd, $^{3}J_{FH}$ = 9.8 Hz, $^{3}J_{FH}$ = 8.3 Hz, $^{4}J_{FH}$ = 6.9 Hz, $J_{FH}$ = 1.3 Hz, 1F, F-C8).

**$^{19}$F{$^{1}$H} NMR** (376 MHz, $CDCl_3$): $\delta$ [ppm] = -91.65 (d, $^{2}J_{FF}$ = 237.9 Hz, 1F, $F^{a}$-C3), -99.02 (d, $^{2}J_{FF}$ = 237.8 Hz, $F^{b}$-C3), -115.41 (s, 1F, F-C8).

**$^{13}$C{$^{1}$H} NMR** (151 MHz, $CDCl_3$): $\delta$ [ppm] = 161.8 (d, $^{1}J_{CF}$ = 244.9 Hz, C8), 143.3 (C11), 140.1 (dd, $^{3}J_{CF}$ = 7.0 Hz, $^{4}J_{CF}$ = 1.8 Hz, C10), 130.5 (dd, $^{3}J_{CF}$ = 8.1 Hz, $^{4}J_{CF}$ = 2.0 Hz, C6), 129.1 (C13), 128.9 (C12), 127.7 (dd, $^{3}J_{CF}$ = 10.7 Hz, $^{4}J_{CF}$ = 3.0 Hz, C5), 127.4 (C14), 122.5 (dd, $^{1}J_{CF}$ = 242.1 Hz, $^{1}J_{CF}$ = 239.2 Hz, C3), 115.8 (dd, $^{3}J_{CF}$ = 22.0 Hz, $^{5}J_{CF}$ = 1.2 Hz, C9), 114.3 (d, $^{3}J_{CF}$ = 21.7 Hz, C7), 44.6 (dt, $^{3}J_{CF}$ = 8.4 Hz, $^{3}J_{CF}$ = $^{4}J_{CF}$ = 1.8 Hz, C1), 40.2 (dd, $^{2}J_{CF}$ = 24.8 Hz, $^{2}J_{CF}$ = 22.3 Hz, C2), 38.0 (dd, $^{2}J_{CF}$ = 27.4 Hz, $^{2}J_{CF}$ = 26.3 Hz, C4).

**GC-EI-MS:** Retention 8.36 min, (*m/z*) requires: [$(C_{16}H_{13}F_3)^{+}$] = 262.0964, (*m/z*) found: [$(C_{16}H_{13}F_3)^{+}$] = 262.0959.

**FT-IR** ($\tilde{\nu}$ = $cm^{-1}$)**:** 3030.5 (w), 1615.7 (w), 1589.9 (w), 1493.8 (m), 1455.0 (w), 1423.4 (w), 1364.6 (w), 1334.5 (w), 1313.0 (w), 1265.6 (m), 1245.5 (m), 1225.4 (m), 1196.7 (w), 1149.4 (w), 1119.2 (m), 1089.1 (m), 1074.8 (s), 1060.4 (s), 1037.4 (s), 1001.6 (w), 965.7 (m), 919.8 (m), 873.9 (m), 856.6 (w), 810.7 (m), 793.5 (m), 764.8 (m), 731.8 (m).

**7-Chloro-3,3-difluoro-1-phenyl-1,2,3,4-tetrahydronaphthalene (3g)**

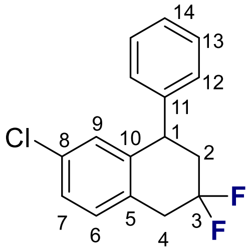


Compound **3g** was prepared according to general procedure **E** (amine:HF 1:5.5) using 3-phenyl-1-(4-chlorophenyl)cyclobutan-1-ol **1g** (51.8 mg, 0.200 mmol). The crude product was purified by flash column chromatography (4-7% DCM in cyclohexane) to yield the title compound as a yellow oil (28.2 mg, 0.101 mmol, 51%).

**R*f*** = 0.38 (10% DCM in cyclohexane).

**$^1$H NMR** (500 MHz, $CDCl_3$): $\delta$ [ppm] = 7.40 – 7.35 (m, 2H, H-C13), 7.31 (tt, $^3J_{HH}$ = 7.3 Hz, $^4J_{HH}$ = 1.4 Hz, 1H, H-C14), 7.21 – 7.15 (m, 3H, H-C7, H-C12), 7.08 (dd, $^3J_{HH}$ = 8.3 Hz, $J_{HH}$ = 1.2 Hz, 1H, H-C6), 6.78 (ddd, $^4J_{HH}$ = 2.2 Hz, $J_{HH}$ = 1.2 Hz, 1H, H-C9), 4.28 (dd, $^3J_{HH}$ = 12.1 Hz, $^3J_{HH}$ = 5.9 Hz, 1H, H-C1), 3.43 – 3.28 (m, 2H, H-C4), 2.57 (ddddd, $^2J_{HH}$ = 13.8 Hz, $^3J_{HF}$ = 10.1 Hz, $^3J_{HF}$ = 8.2 Hz, $^3J_{HH}$ = 5.8 Hz, $J_{HH}$ = 1.8 Hz, 1H, $H^a$-C2), 2.27 (dddd, $^3J_{HF}$ = 32.2 Hz, $^2J_{HH}$ = 13.7 Hz, $^3J_{HH}$ = 12.1 Hz, $^3J_{HF}$ = 3.0 Hz, 1H, $H^b$-C2).

**$^{19}$F NMR** (376 MHz, $CDCl_3$): $\delta$ [ppm] = -91.73 (dddddd, $^2J_{FF}$ = 238.5 Hz, $^3J_{FH}$ = 10.2 Hz, $^3J_{FH}$ = 8.3 Hz, $^3J_{FH}$ = 5.7 Hz, $^3J_{FH}$ = 2.9 Hz, $J_{FH}$ = 1.7 Hz, 1F, $F^a$-C3), -98.89 (ddddd, $^2J_{FF}$ = 238.6 Hz, $^3J_{FH}$ = 32.3 Hz, $^3J_{FH}$ = 27.6 Hz, $^3J_{FH}$ = 18.0 Hz, $^3J_{FH}$ = 8.1 Hz, 1F, $F^b$-C3).

**$^{19}$F{$^1$H} NMR** (376 MHz, $CDCl_3$): $\delta$ [ppm] = -91.73 (d, $^2J_{FF}$ = 238.4 Hz, 1F, $F^a$-C3), -98.89 (dd, $^2J_{FF}$ = 238.4 Hz, $F^b$-C3).

**$^{13}$C{$^1$H} NMR** (126 MHz, $CDCl_3$): $\delta$ [ppm] = 143.2 (C11), 139.8 (d, $^4J_{CF}$ = 1.8 Hz, C10), 132.8 (C8), 130.6 (d, $^3J_{CF}$ = 10.5 Hz, C5), 130.4 (d, $^4J_{CF}$ = 1.9 Hz, C6), 129.3 – 129.1 (m, C9, C13), 128.8 (C12), 127.5 (C14), 127.3 (C7), 122.5 (dd, $^1J_{CF}$ = 242.3 Hz, $^1J_{CF}$ = 239.1 Hz, C3), 44.5 (dd, $^3J_{CF}$ = 8.4 Hz, $^3J_{CF}$ = 2.2 Hz, C1), 40.4 (dd, $^2J_{CF}$ = 24.8 Hz, $^2J_{CF}$ = 22.2 Hz, C2), 38.2 (dd, $^2J_{CF}$ = 27.5 Hz, $^2J_{CF}$ = 26.4 Hz, C4).

**GC-EI-MS:** Retention 9.75 min, (*m/z*) requires: [$(C_{16}H_{13}ClF_2)^+$] = 278.0668, (*m/z*) found: [$(C_{16}H_{13}ClF_2)^+$] = 278.0670.

**FT-IR** ($\tilde{\nu}$ = $cm^{-1}$)**:** 1597.1 (w), 1495.2 (w), 1482.3 (m), 1455.0 (w), 1424.9 (w), 1406.2 (w), 1353.1 (w), 1333.0 (w), 1311.5 (w), 1267.0 (m), 1238.3 (w), 1195.3 (w), 1178.1 (w), 1129.3 (m), 1100.6 (m), 1077.6 (s), 1061.8 (s), 1036.0 (m), 1027.4 (m), 1001.6 (w), 957.1 (m), 882.5 (s), 845.2 (w), 802.1 (s), 754.8 (s), 713.2 (m).

**7-Bromo-3,3-difluoro-1-phenyl-1,2,3,4-tetrahydronaphthalene (3h)**

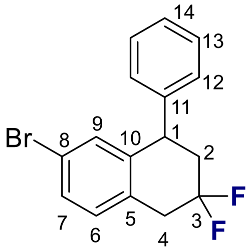

Compound **3h** was prepared according to general procedure **E** (amine:HF 1:5.5) using 3-phenyl-1-(4-bromophenyl)cyclobutan-1-ol **1h** (60.6 mg, 0.200 mmol). The crude product was purified by flash column chromatography (4-7% DCM in cyclohexane) to yield the title compound as a yellow oil (34.4 mg, 0.101 mmol, 53%).

**R*f*** = 0.39 (10% DCM in cyclohexane).

**$^{1}$H NMR** (500 MHz, $CDCl_3$): $\delta$ [ppm] = 7.40 – 7.34 (m, 2H, H-C13), 7.34 – 7.29 (m, 2H, H-C7, H-C14), 7.20 – 7.16 (m, 2H, H-C12), 7.02 (d, $^{3}J_{HH}$ = 8.2 Hz, 1H, H-C6), 6.94 (dd, $^{4}J_{HH}$ = 2.0 Hz, $J_{HH}$= 1.2 Hz, 1H, H-C9), 4.29 (dd, $^{3}J_{HH}$ = 12.0 Hz, $^{3}J_{HH}$ = 5.9 Hz, 1H, H-C1), 3.40 – 3.28 (m, 2H, H-C4), 2.57 (dtdd, $^{2}J_{HH}$ = 13.9 Hz, $^{3}J_{HF}$ = 8.2 Hz, $^{3}J_{HH}$ = 5.8 Hz, $J_{HF}$ = 2.4 Hz, 1H, $H^{a}$-C2), 2.26 (dddd, $^{3}J_{HF}$ = 32.1 Hz, $^{2}J_{HH}$ = 14.6 Hz, $^{3}J_{HH}$ = 12.2 Hz, $^{3}J_{HF}$ = 2.8 Hz, 1H, $H^{b}$-C2).

**$^{19}$F NMR** (470 MHz, $CDCl_3$): $\delta$ [ppm] = -91.79 (dddddd, $^{2}J_{FF}$ = 238.5 Hz, $^{3}J_{FH}$ = 10.5 Hz, $^{3}J_{FH}$ = 8.4 Hz, $^{3}J_{FH}$ = 4.8 Hz, $^{3}J_{FH}$ = 2.3 Hz, $J_{FH}$ = 1.6 Hz, 1F, $F^{a}$-C3), -98.89 (ddddd, $^{2}J_{FF}$ = 238.5 Hz, $^{3}J_{FH}$ = 32.2 Hz, $^{3}J_{FH}$ = 27.7 Hz, $^{3}J_{FH}$ = 17.7 Hz, $^{3}J_{FH}$ = 8.1 Hz, 1F, $F^{b}$-C3).

**$^{19}$F{$^{1}$H} NMR** (470 MHz, $CDCl_3$): $\delta$ [ppm] = -91.79 (d, $^{2}J_{FF}$ = 238.4 Hz, 1F, $F^{a}$-C3), -98.89 (d, $^{2}J_{FF}$ = 238.5 Hz, $F^{b}$-C3).

**$^{13}$C{$^{1}$H} NMR** (126 MHz, $CDCl_3$): $\delta$ [ppm] = 143.2 (C11), 140.1 (d, $^{4}J_{CF}$ = 1.7 Hz, C10), 132.1 (d, $^{5}J_{CF}$ = 1.1 Hz, C9), 131.1 (d, $^{3}J_{CF}$ = 9.9 Hz, C5), 130.7 (d, $^{4}J_{CF}$ = 1.9 Hz, C6), 130.2 (C7), 129.2 (C13), 128.8 (C12), 127.5 (C14), 122.2 (dd, $^{1}J_{CF}$ = 242.3 Hz, $^{1}J_{CF}$ = 239.1 Hz, C3), 120.9 (C8), 44.4 (dd, $^{3}J_{CF}$ = 8.3 Hz, $^{3}J_{CF}$ = 2.2 Hz, C1), 40.3 (dd, $^{2}J_{CF}$ = 24.7 Hz, $^{2}J_{CF}$ = 22.2 Hz, C2), 38.3 (t, $^{2}J_{CF}$ = 26.5 Hz, C4).

**GC-EI-MS:** Retention 10.10 min, (*m/z*) requires: [$(C_{16}H_{13}BrF_2)^+$] = 322.0163, (*m/z*) found: [$(C_{16}H_{13}BrF_2)^+$] = 322.0163.

**FT-IR** ($\tilde{\nu}$ = $cm^{-1}$)**:** 1592.8 (w), 1495.2 (w), 1480.8 (m), 1453.6 (w), 1424.9 (w), 1403.4 (w), 1361.7 (w), 1333.0 (w), 1311.5 (w), 1267.0 (m), 1238.3 (w), 1195.3 (w), 1175.2 (w), 1127.8 (m), 1094.8 (m), 1077.6 (s), 1060.4 (s), 1034.6 (m), 1026.0 (m), 1001.6 (w), 955.7 (m), 875.3 (s), 842.3 (m), 797.8 (s), 751.9 (m).

**3,3-Difluoro-1-phenyl-7-(trifluoromethoxy)-1,2,3,4-tetrahydronaphthalene (3i)**

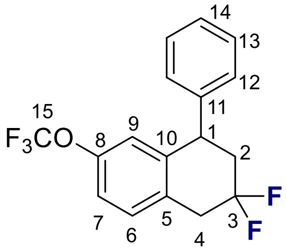


Compound **3i** was prepared according to general procedure **E** (amine:HF 1:5.5) using 3-phenyl-1-(4-(trifluoromethoxy)phenyl)cyclobutan-1-ol **1i** (61.7 mg, 0.200 mmol). The crude product was purified by flash column chromatography (4-7% DCM in cyclohexane) to yield the title compound as a yellow oil (26.9 mg, 0.082 mmol, 41%).

$\mathbf{R}_f$ = 0.33 (10% DCM in cyclohexane).

**Melting Point:** 51-53 ˚C.

**$^1$H NMR** (599 MHz, $CDCl_3$): $\delta$ [ppm] = 7.39 – 7.35 (m, 2H, H-C13), 7.33 – 7.30 (m, 1H, H-C14), 7.21 – 7.18 (m, 2H, H-C12), 7.17 (d, $^3J_{HH}$ = 8.5 Hz, 1H, H-C6), 7.09 – 7.05 (m, 1H, H-C7), 6.65 (s, 1H, H-C9), 4.32 (dd, $^3J_{HH}$ = 12.2 Hz, $^3J_{HH}$ = 5.9 Hz, 1H, H-C1), 3.46 – 3.32 (m, 2H, H-C4), 2.59 (dtdd, $^2J_{HH}$ = 14.0 Hz, $^3J_{HF}$ = 8.2 Hz, $^3J_{HH}$ = 5.9 Hz, $J_{HH}$ = 2.3 Hz, 1H, $H^a$-C2), 2.29 (dddd, $^3J_{HF}$ = 32.3 Hz, $^2J_{HH}$ = 14.3 Hz, $^3J_{HH}$ = 12.1 Hz, $^3J_{HF}$ = 2.6 Hz, 1H, $H^b$-C2).

**$^{19}$F NMR** (564 MHz, $CDCl_3$): $\delta$ [ppm] = -58.04 (s, 3F, F-C15), -91.86 (ddddd, $^2J_{FF}$ = 238.7 Hz, $^3J_{FH}$ = 10.1 Hz, $^3J_{FH}$ = 8.4 Hz, $^3J_{FH}$ = 5.3 Hz, $^3J_{FH}$ = 2.5 Hz, 1F, $F^a$-C3), -98.86 (ddddd, $^2J_{FF}$ = 238.7 Hz, $^3J_{FH}$ = 32.3 Hz, $^3J_{FH}$ = 27.0 Hz, $^3J_{FH}$ = 18.4 Hz, $^3J_{FH}$ = 8.0 Hz, 1F, $F^b$-C3).

**$^{19}$F{$^1$H} NMR** (564 MHz, $CDCl_3$): $\delta$ [ppm] = -58.04 (s, 3F, F-C15), -91.86 (d, $^2J_{FF}$ = 238.7 Hz, 1F, $F^a$-C3), -98.86 (d, $^2J_{FF}$ = 238.7 Hz, $F^b$-C3).

**$^{13}$C{$^1$H} NMR** (151 MHz, $CDCl_3$): $\delta$ [ppm] = 148.2 (q, $^3J_{CF}$ = 1.8 Hz, C8), 143.1 (C11), 140.0 (d, $^4J_{CF}$ = 1.7 Hz, C10), 130.8 (d, $^3J_{CF}$ = 10.6 Hz, C5), 130.4 (d, $^4J_{CF}$ = 2.0 Hz, C6), 129.2 (C13), 128.8 (C12), 127.5 (C14), 122.3 (dd, $^1J_{CF}$ = 242.3 Hz, $^1J_{CF}$ = 239.1 Hz, C3), 121.9 (C9), 120.5 (q, $^1J_{CF}$ = 257.1 Hz, C15), 119.6 (C7), 44.5 (dd, $^3J_{CF}$ = 8.5 Hz, $^3J_{CF}$ = 2.1 Hz, C1), 40.2 (dd, $^2J_{CF}$ = 24.7 Hz, $^2J_{CF}$ = 22.3 Hz, C2), 38.2 (t, $^2J_{CF}$ = 26.6 Hz, C4).

**GC-EI-MS:** Retention 8.69 min, (*m/z*) requires: [$(C_{17}H_{13}F_5O)^+$] = 328.0881, (*m/z*) found: [$(C_{17}H_{13}F_5O)^+$] = 328.0880.

**FT-IR** ($\tilde{\nu}$ = $cm^{-1}$)**:** 1495.2 (m), 1455.0 (w), 1367.5 (w), 1356.0 (w), 1334.5 (w), 1315.8 (w), 1262.7 (m), 1242.6 (m), 1208.2 (s), 1163.7 (s), 1153.7 (s), 1122.1 (m), 1093.4 (m), 1080.5 (m), 1061.8 (m), 1040.3 (m), 1027.4 (m), 1003.0 (w), 970.0 (m), 941.3 (m), 914.0 (w), 892.5 (w), 876.7 (m), 853.8 (w), 8278.0 (m), 794.9 (w), 772.0 (m), 743.3 (m), 704.5 (m).

**3,3-Difluoro-1-phenyl-7-(trifluoromethyl)-1,2,3,4-tetrahydronaphthalene (3j)**

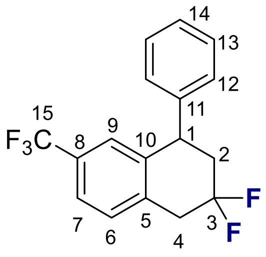


Compound **3j** was prepared according to general procedure **F** using 1-(2,2,4-trifluoro-4-phenylbutyl)-4-(trifluoromethyl)benzene **2j** (66.5 mg, 0.200 mmol). The reaction was stirred for 24 h at room temperature. The crude product was purified by flash column chromatography (4-7% DCM in cyclohexane) to yield the title compound as a colorless solid (53.0 mg, 0.170 mmol, 85%).

**R*f*** = 0.33 (10% DCM in cyclohexane).

**Melting Point:** 53-55 ˚C.

**$^{1}$H NMR** (599 MHz, $CDCl_3$): $\delta$[ppm] = 7.47 – 7.44 (m, 1H, H-C7), 7.40 – 7.36 (m, 2H, H-C13), 7.34 – 7.30 (m, 1H, H-C14), 7.27 (d, $^{3}J_{HH}$ = 8.0 Hz, 1H, H-C6), 7.21 – 7.17 (m, 2H, H-C12), 7.08 (s, 1H, H-C9), 4.36 (dd, $^{3}J_{HH}$ = 12.1 Hz, $^{3}J_{HH}$ = 5.9 Hz, 1H, H-C1), 3.52 – 3.37 (m, 2H, H-C4), 2.63 (dtdd, $^{2}J_{HH}$ = 13.8 Hz, $^{3}J_{HF}$ = 8.3 Hz, $^{3}J_{HH}$ = 5.9 Hz, $J_{HH}$ = 2.4 Hz, 1H, H$^{a}$-C2), 2.31 (dddd, $^{3}J_{HF}$ = 32.2 Hz, $^{2}J_{HH}$ = 13.9 Hz, $^{3}J_{HH}$ = 12.0 Hz, $^{3}J_{HF}$ = 2.8 Hz, 1H, H$^{b}$-C2).

**$^{19}$F NMR** (376 MHz, $CDCl_3$): $\delta$ [ppm] = -62.59 (s, 3F, F-C15), -91.84 (dddddd, $^{2}J_{FF}$ = 239.3 Hz, $^{3}J_{FH}$ = 9.9 Hz, $^{3}J_{FH}$ = 8.1 Hz, $^{3}J_{FH}$ = 5.9 Hz, $^{3}J_{FH}$ = 2.6 Hz, $J_{FH}$ = 1.9 Hz, 1F, F$^{a}$-C3), -98.62 (ddddd, $^{2}J_{FF}$ = 239.3 Hz, $^{3}J_{FH}$ = 32.0 Hz, $^{3}J_{FH}$ = 26.7 Hz, $^{3}J_{FH}$ = 18.2 Hz, $^{3}J_{FH}$ = 8.0 Hz, 1F, F$^{b}$-C3).

**$^{19}$F{$^{1}$H} NMR** (376 MHz, $CDCl_3$): $\delta$[ppm] = -62.60 (s, 3F, F-C15), -91.84 (d, $^{2}J_{FF}$ = 239.3 Hz, 1F, F$^{a}$-C3), -98.62 (d, $^{2}J_{FF}$ = 239.3 Hz, F$^{b}$-C3).

**$^{13}$C{$^{1}$H} NMR** (151 MHz, $CDCl_3$): $\delta$ [ppm] = 143.0 (C11), 138.8 (d, $^{4}J_{CF}$ = 1.7 Hz, C10), 136.2 (d, $^{3}J_{CF}$ = 10.4 Hz, C5), 129.7 (d, $^{4}J_{CF}$ = 1.8 Hz, C6), 129.5 (q, $^{2}J_{CF}$ = 32.4 Hz, C8), 129.3 (C13), 128.8 (C12), 127.6 (C14), 126.2 (qd, $^{3}J_{CF}$ = 4.0 Hz, $^{5}J_{CF}$ = 1.0 Hz, C9), 124.1 (q, $^{1}J_{CF}$ = 272.3 Hz, C15), 123.7 (q, $^{3}J_{CF}$ = 3.7 Hz, C7), 122.0 (dd, $^{1}J_{CF}$ = 242.5 Hz, $^{1}J_{CF}$ = 239.0 Hz, C3), 44.5 (dd, $^{3}J_{CF}$ = 8.2 Hz, $^{3}J_{CF}$ = 2.3 Hz, C1), 40.4 (dd, $^{2}J_{CF}$ = 24.7 Hz, $^{2}J_{CF}$ = 22.2 Hz, C2), 38.7 (t, $^{2}J_{CF}$ = 27.2 Hz, C4).

**GC-EI-MS:** Retention 8.14 min, (*m/z*) requires: [$(C_{17}H_{13}F_5)^+$] = 312.0932, (*m/z*) found: [$(C_{17}H_{13}F_5)^+$] = 312.0931.

**FT-IR** ($\tilde{\nu}$ = $cm^{-1}$)**:** 1618.6 (w), 1496.6 (w), 1455.0 (w), 1449.2 (w), 1423.4 (w), 1368.9 (m), 1331.6 (m), 1313.0 (m), 1268.5 (m), 1239.8 (m), 1179.5 (w), 1153.7 (m), 1123.5 (s), 1073.3 (s), 1061.8 (m), 1037.4 (s), 1026.0 (m), 1001.6 (w), 958.5 (m), 914.0 (w), 888.2 (m), 829.4 (m), 807.9 (m), 793.5 (w), 763.4 (m), 737.6 (m), 718.9 (w), 700.2 (s).

**3,3-Difluoro-1-phenyl-7-(4-(trifluoromethyl)phenyl)-1,2,3,4-tetrahydronaphthalene (3l)**

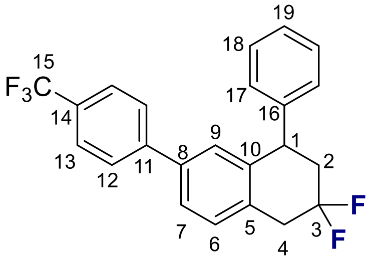


Compound **3l** was prepared according to general procedure **E** (amine:HF 1:5.5) using 3-phenyl-1-(4'-(trifluoromethyl)-[1,1'-biphenyl]-4-yl)-cyclobutanol **1l** (73.7 mg, 0.200 mmol). The crude product was purified by flash column chromatography (0-2% DCM in *n*-pentane) to yield the title compound as a colorless oil (45.2 mg, 0.116 mmol, 58%).

**R*f*** = 0.27 (5% DCM in *n*-pentane).

**$^{1}$H NMR** (599 MHz, $CDCl_3$): $\delta$ [ppm] = 7.61 (d, $^{3}J_{HH}$ = 8.2 Hz, 2H, H-C13), 7.49 (d, $^{3}J_{HH}$ = 8.2 Hz, 2H, H-C12), 7.45 – 7.43 (m, 1H, H-C7), 7.39 (t, $^{3}J_{HH}$ = 7.5 Hz, 2H, H-C18), 7.34 – 7.30 (m, 1H, H-C19), 7.29 – 7.25 (m, 3H, H-C6, H-C17), 7.06 – 7.05 (m, 1H, H-C9), 4.42 (dd, $^{3}J_{HH}$ = 11.8 Hz, $^{3}J_{HH}$ = 5.8 Hz, 1H, H-C1), 3.54 – 3.40 (m, 2H, H-C4), 2.65 (dtdd, $^{2}J_{HH}$ = 13.7 Hz, $^{3}J_{FH}$ = 8.2 Hz, $^{3}J_{HH}$ = 5.9 Hz, $J_{HH}$ = 2.3 Hz, 1H, $H^{a}$-C2), 2.35 (dddd, $^{3}J_{HF}$ = 32.3 Hz, $^{2}J_{HH}$ = 14.3 Hz, $^{3}J_{HH}$ = 12.1 Hz, $^{3}J_{HF}$ = 2.6 Hz, 1H, $H^{b}$-C2).

**$^{19}$F NMR** (564 MHz, $CDCl_3$): $\delta$ [ppm] = -62.47 (s, 3F, F-C19), -91.53 (ddddd, $^{2}J_{FF}$ = 238.0 Hz, $^{3}J_{FH}$ = 10.4 Hz, $^{3}J_{FH}$ = 8.0 Hz, $^{3}J_{FH}$ = 5.8 Hz, $^{3}J_{FH}$ = 2.8 Hz, 1F, $F^{a}$-C3), -98.75 (ddddd, $^{2}J_{FF}$ = 238.0 Hz, $^{3}J_{FH}$ = 32.3 Hz, $^{3}J_{FH}$ = 28.7 Hz, $^{3}J_{FH}$ = 16.4 Hz, $^{3}J_{FH}$ = 7.9 Hz, 1F, $F^{b}$-C3).

**$^{19}$F{$^{1}$H} NMR** (564 MHz, $CDCl_3$): $\delta$ [ppm] = -62.47 (s, 3F, F-C19), -91.54 (d, $^{2}J_{FF}$ = 238.0 Hz, 1F, $F^{a}$-C3), -98.75 (d, $^{2}J_{FF}$ = 238.0 Hz, 1F, $F^{b}$-C3).

**$^{13}$C{$^{1}$H} NMR** (151 MHz, $CDCl_3$): $\delta$ [ppm] = 144.3 (q, $^{5}J_{CF}$ = 1.4 Hz, C11), 143.8 (C16), 138.6 (C8), 132.3 (d, $^{3}J_{CF}$ = 10.3 Hz, C5), 129.9 (d, $^{4}J_{CF}$ = 1.7 Hz, C6), 129.6 (q, $^{2}J_{CF}$ = 32.6 Hz, C14), 129.1 (C18), 128.9 (C17), 128.2 (C9), 127.4 (C12), 127.5 (C10), 127.3 (C19), 125.9 (C7), 125.8 (q, $^{3}J_{CF}$ = 3.8 Hz, C13), 124.4 (q, $^{1}J_{CF}$ = 271.9 Hz, C15),122.5 (dd, $^{1}J_{CF}$ = 242.2 Hz, $^{1}J_{CF}$ = 239.1 Hz, C3), 44.7 (dd, $^{3}J_{CF}$ = 8.3 Hz, $^{3}J_{CF}$ = 2.1 Hz, C1), 40.6 (dd, $^{2}J_{CF}$ = 24.7 Hz, $^{2}J_{CF}$ = 22.1 Hz, C2), 38.3 (dd, $^{2}J_{CF}$ = 27.5 Hz, $^{2}J_{CF}$ = 26.1 Hz, C4).

**GC-EI-MS:** Retention 10.51 min, (*m/z*) requires: [$(C_{23}H_{17}F_5)^+$] = 388.1245, (*m/z*) found: [$(C_{23}H_{17}F_5)^+$] = 388.1244.

**FT-IR** ($\tilde{\nu}$ = $cm^{-1}$)**:** 2360.4 (broad), 1615.7 (m), 1495.2 (m), 1455.0 (w), 1424.9 (w), 1394.7 (w), 1364.6 (w), 1298.6 (m), 1271.3 (s), 1241.2 (w), 1163.7 (s), 1122.1 (s), 1110.6 (s), 1096.3 (s), 1043.2 (s), 1014.5 (s), 957.1 (s), 914.0 (w), 885.3 (m), 846.6 (s), 812.2 (s), 793.5 (m), 776.3 (m), 756.2 (s), 743.3 (s), 723.2 (m), 700.2 (s), 652.9 (m), 609.8 (w).

**6-Bromo-3,3-difluoro-1-phenyl-1,2,3,4-tetrahydronaphthalene (3m)**

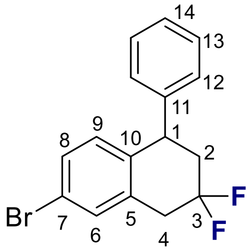


Compound **3m** was prepared according to general procedure **E** (amine:HF 1:5.5) using 1-(3-bromophenyl)-3-phenylcyclobutan-1-ol **1m** (60.6 mg, 0.200 mmol). The crude product was purified by flash column chromatography (2% DCM in *n*-pentane) to yield the title compound as a colorless oil (33.1 mg, 0.102 mmol, 51%).

**R*f*** = 0.33 (5% DCM in *n*-pentane).

**$^{1}$H NMR** (500 MHz, $CDCl_3$): $\delta$ [ppm] = 7.39 – 7.33 (m, 2H, H-C13), 7.32 – 7.28 (m, 2H, H-C6, H-C14), 7.23 – 7.16 (m, 3H, H-C8, H-C12), 6.68 (d, $^{3}J_{HH}$ = 8.4 Hz, 1H, H-C9), 4.26 (dd, $^{3}J_{HH}$ = 12.0 Hz, $^{3}J_{HH}$ = 5.9 Hz, 1H, H-C1), 3.45 – 3.32 (m, 2H, H-C4), 2.58 (dtdd, $^{2}J_{HH}$ = 13.6 Hz, $^{3}J_{FH}$ = 8.3 Hz, $^{3}J_{HH}$ = 6.0 Hz, $J_{HH}$ = 2.6 Hz, 1H, $H^{a}$-C2), 2.27 (dddd, $^{3}J_{HF}$ = 32.5 Hz, $^{2}J_{HH}$ = 14.0 Hz, $^{3}J_{HH}$ = 12.1 Hz, $^{3}J_{HF}$ = 2.8 Hz, 1H, $H^{b}$-C2).

**$^{19}$F NMR** (376 MHz, $CDCl_3$): $\delta$ [ppm] = -91.79 (ddddd, $^{2}J_{FF}$ = 240.5 Hz, $^{3}J_{FH}$ = 10.4 Hz, $^{3}J_{FH}$ = 8.1 Hz, $^{3}J_{FH}$ = 6.4 Hz, $^{3}J_{FH}$ = 3.0 Hz, 1F, $F^{a}$-C3), -98.89 (ddddd, $^{2}J_{FF}$ = 238.7 Hz, $^{3}J_{FH}$ = 32.1 Hz, $^{3}J_{FH}$ = 27.9 Hz, $^{3}J_{FH}$ = 16.7 Hz, $^{3}J_{FH}$ = 8.0 Hz, 1F, $F^{b}$-C3).

**$^{19}$F{$^{1}$H} NMR** (376 MHz, $CDCl_3$): $\delta$ [ppm] = -91.79 (d, $^{2}J_{FF}$ = 238.7 Hz, $F^{a}$-C3), -98.89 (d, $^{2}J_{FF}$ = 238.7 Hz, $F^{b}$-C3).

**$^{13}$C{$^{1}$H} NMR** (126 MHz, $CDCl_3$): $\delta$ [ppm] = 143.6 (C11), 136.9 (d, $^{4}J_{CF}$ = 1.7 Hz, C10), 134.4 (d, $^{3}J_{CF}$ = 10.5 Hz, C5), 131.8 (d, $^{4}J_{CF}$ = 1.9 Hz, C6), 131.1 (d, $^{5}J_{CF}$ = 1.2 Hz, C9), 130.2 (C8), 129.1 (C13), 128.8 (C12), 127.3 (C14), 122.1 (dd, $^{1}J_{CF}$ = 242.4 Hz, $^{1}J_{CF}$ = 239.2 Hz, C3), 120.7 (d, $^{5}J_{CF}$ = 1.2 Hz, C7), 44.2 (dd, $^{3}J_{CF}$ = 8.4 Hz, $^{3}J_{CF}$ = 2.2 Hz, C1), 40.3 (dd, $^{2}J_{CF}$ = 24.7 Hz, $^{2}J_{CF}$ = 22.1 Hz, C2), 38.4 (dd, $^{2}J_{CF}$ = 27.7 Hz, $^{2}J_{CF}$ = 26.7 Hz, C4).

**GC-EI-MS:** Retention 9.96 min, (*m/z*) requires: [($C_{16}H_{13}BrF_2$)$^{+}$] = 322.0162, (*m/z*) found: [($C_{16}H_{13}BrF_2$)$^{+}$] = 322.0163.

**FT-IR** ($\tilde{\nu}$ = $cm^{-1}$)**:** 1492.3 (m), 1275.6 (w), 1267.0 (m), 1192.4 (m), 1119.2 (w), 1083.4 (m), 1059.0 (m), 1030.3 (w), 1013.1 (w), 883.9 (w), 871.0 (w), 850.9 (w), 850.9 (m), 809.3 (m), 764.8 (s), 751.9 (s), 718.9 (m), 700.2 (s), 651.5 (w).

**5-Bromo-3,3-difluoro-1-phenyl-1,2,3,4-tetrahydronaphthalene (3n)**

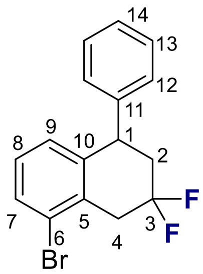


Compound **3n** was prepared according to modified general procedure **E** (amine:HF 1:5.5) using 1-(2-bromophenyl)-3-phenylcyclobutan-1-ol **1n** (60.6 mg, 0.200 mmol). The reaction time for the formation of intermediate **2n** was elongated to 42 h. The crude product was purified by flash column chromatography (100% *n*-pentane) to yield the title compound as a colorless oil (27.1 mg, 0.086 mmol, 43%).

**R*f*** = 0.38 (100% *n*-pentane).

**$^1$H NMR** (599 MHz, $CDCl_3$): $\delta$ [ppm] = 7.46 (d, $^3J_{HH}$ = 7.9 Hz, 1H, H-C7), 7.36 (t, $^3J_{HH}$ = 7.3 Hz, 2H, H-C12), 7.32 – 7.29 (m, 1H, H-C14), 7.20 – 7.16 (m, 2H, H-C13), 6.96 (t, $^3J_{HH}$ = 7.9 Hz, 1H, H-C8), 6.76 (d, $^3J_{HH}$ = 7.8 Hz, 1H, H-C9), 4.36 (dd, $^3J_{HH}$ = 12.5 Hz, $^3J_{HH}$ = 5.9 Hz, 1H, H-C1), 3.50 (tt, $^2J_{HH}$ = $^3J_{HF}$ = 17.0 Hz, $J_{HH}$ = $^3J_{HF}$ = 2.3 Hz, 1H, $H^a$-C4), 3.28 (ddd, $^3J_{FH}$ = 29.6 Hz, $^2J_{HH}$ = 17.8 Hz, $^3J_{FH}$ = 9.7 Hz, $H^b$-C4), 2.55 (dtdd, $^2J_{HH}$ = 13.2 Hz, $^3J_{HF}$ = 8.0 Hz, $^3J_{HH}$ = 5.5 Hz, $J_{HH}$ = 2.6 Hz, $H^a$-C2), 2.28 (dtd, $^3J_{FH}$ = 32.9 Hz, $^2J_{HH}$ = $^3J_{HH}$ = 14.0 Hz, $^3J_{FH}$ = 1.9 Hz, $H^b$-C2).

**$^{19}$F NMR** (564 MHz, $CDCl_3$): $\delta$ [ppm] = -91.80 (dtdt, $^2J_{FF}$ = 240.1 Hz, $^3J_{FH}$ = 9.6 Hz, $^3J_{FH}$ = 4.0 Hz, $^3J_{FH}$ = $J_{FH}$ = 2.1 Hz, 1F, $F^a$-C3), -97.00 (ddddd, $^2J_{FF}$ = 239.9 Hz, $^3J_{FH}$ = 32.9 Hz, $^3J_{FH}$ = 30.3 Hz, $^3J_{FH}$ = 16.3 Hz, $^3J_{FH}$ = 7.4 Hz, 1F, $F^b$-C3).

**$^{19}$F{$^1$H} NMR** (564 MHz, $CDCl_3$): $\delta$ [ppm] = -91.78 (d, $^2J_{FF}$ = 240.0 Hz, 1F, $F^a$-C3), -97.00 (d, $^2J_{FF}$ = 240.0 Hz, 1F, $F^b$-C3).

**$^{13}$C{$^1$H} NMR** (151 MHz, $CDCl_3$): $\delta$ [ppm] = 143.7 (C11), 140.5 (d, $^4J_{CF}$ = 1.7 Hz, C10), 132.1 (d, $^3J_{CF}$ = 11.3 Hz, C5) 131.0 (C7), 129.1 (C12), 128.9 (C13), 128.6 (C9), 128.0 (C8), 127.3 (C14), 125.2 (d, $^4J_{CF}$ = 1.7 Hz, C6), 122.3 (dd, $^1J_{CF}$ = 241.5 Hz, $^1J_{CF}$ = 238.3 Hz, C3), 44.9 (dd, $^3J_{CF}$ = 8.4 Hz, $^3J_{CF}$ = 2.3 Hz, C1), 40.2 (dd, $^2J_{CF}$ = 24.6 Hz , $^2J_{CF}$ = 22.1 Hz, C2), 39.9 (t, $^2J_{CF}$ = 28.3 Hz, C4).

**GC-EI-MS:** Retention 9.40 min, (*m/z*) requires: [$(C_{16}H_{13}F_2Br)^+$] = 322.0163, (*m/z*) found: [$(C_{16}H_{13}F_2Br)^+$] = 322.0163.

**FT-IR** ($\tilde{\nu}$ = $cm^{-1}$)**:** 2935.8 (broad), 1495.2 (w), 1453.6 (m),1437.8 (m), 1417.7 (w), 1361.7 (m), 1331.6 (w), 1313.0 (w), 1274.2 (m), 1261.3 (w), 1225.4 (w), 1192.4 (w), 1133.6 (m), 1102.0 (s), 1079.1 (s), 1059.0 (s), 1034.6 (m), 1026.0 (m), 961.4 (m), 875.3 (m), 839.4 (m), 796.4 (m), 770.6 (s), 757.6 (s), 713.2 (s).

**3,3-Difluoro-1-(4-fluorophenyl)-1,2,3,4-tetrahydronaphthalene (3o)**

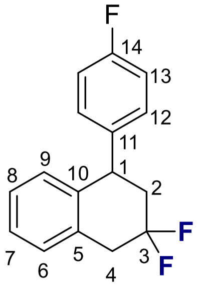


Compound **3o** was prepared according to general procedure **E** (amine:HF 1:4.5) using 3-(4-fluorophenyl)-1-phenylcyclobutan-1-ol **1o** (48.5 mg, 0.200 mmol). The crude product was purified by flash column chromatography (4-7% DCM in cyclohexane) to yield the title compound as a colorless oil (29.8 mg, 0.114 mmol, 57%).

**R*f*** = 0.30 (10% DCM in cyclohexane).

**$^{1}$H NMR** (599 MHz, $CDCl_3$): $\delta$[ppm] = 7.23 – 7.19 (m, 1H, H-C7), 7.19 – 7.14 (m, 3H, H-C6, H-C12), 7.11 (t, $^{3}J_{HH}$ = 7.4 Hz, 1H, H-C8), 7.07 – 7.02 (m, 2H, H-C13), 6.81 (dt, $^{3}J_{HH}$ = 7.8 Hz, $J_{HH}$= 1.2 Hz, 1H, H-C9), 4.34 (dd, $^{3}J_{HH}$ = 11.9 Hz, $^{3}J_{HH}$ = 5.9 Hz, 1H, H-C1), 3.49 – 3.30 (m, 2H, H-C4), 2.57 (dtdd, $^{2}J_{HH}$ = 13.7 Hz, $^{3}J_{HF}$ = 8.2 Hz, $^{3}J_{HH}$ = 5.9 Hz, $J_{HH}$ = 2.8 Hz, 1H, H$^{a}$-C2), 2.26 (dddd, $^{3}J_{HF}$ = 31.8 Hz, $^{2}J_{HH}$ = 14.2 Hz, $^{3}J_{HH}$ = 11.7 Hz, $^{3}J_{HF}$ = 2.9 Hz, 1H, H$^{b}$-C2).

**$^{19}$F NMR** (564 MHz, $CDCl_3$): $\delta$ [ppm] = -91.38 (ddddd, $^{2}J_{FF}$ = 237.8 Hz, $^{3}J_{FH}$ = 11.4 Hz, $^{3}J_{FH}$ = 8.6 Hz, $^{3}J_{FH}$ = 6.1 Hz, $^{3}J_{FH}$ = 2.8 Hz, 1F, F$^{a}$-C3), -98.65 (ddddd, $^{2}J_{FF}$ = 237.8 Hz, $^{3}J_{FH}$ = 31.8 Hz, $^{3}J_{FH}$ = 28.7 Hz, $^{3}J_{FH}$ = 16.3 Hz, $^{3}J_{FH}$ = 7.8 Hz, 1F, F$^{b}$-C3), -115.84 (tt, $^{3}J_{FH}$ = 8.6 Hz, $^{4}J_{FH}$ = 5.3 Hz, 1F, F-C14).

**$^{19}$F{$^{1}$H} NMR** (564 MHz, $CDCl_3$): $\delta$ [ppm] = -91.38 (d, $^{2}J_{FF}$ = 237.8 Hz, 1F, F$^{a}$-C3), -98.65 (d, $^{2}J_{FF}$ = 237.8 Hz, F$^{b}$-C3), -115.84 (s, 1F, F-C14).

**$^{13}$C{$^{1}$H} NMR** (151 MHz, $CDCl_3$): $\delta$[ppm] = 161.1 (d, $^{1}J_{CF}$ = 245.4 Hz, C14), 139.9 (d, $^{4}J_{CF}$ = 3.2 Hz, C11), 137.5 (d, $^{4}J_{CF}$ = 1.7 Hz, C10), 132.1 (d, $^{3}J_{CF}$ = 10.2 Hz, C5), 130.4 (d, $^{3}J_{CF}$ = 8.2 Hz, C12) 129.24 (d, $^{5}J_{CF}$ = 1.8 Hz, C9), 129.22 (d, $^{4}J_{CF}$ = 1.0 Hz, C6), 127.1 (C7), 127.0 (C8), 122.5 (dd, $^{1}J_{CF}$ = 242.0 Hz, $^{1}J_{CF}$ = 239.1 Hz, C3), 115.8 (d, $^{2}J_{CF}$ = 21.4 Hz, C13), 43.8 (dd, $^{3}J_{CF}$ = 8.2 Hz, $^{3}J_{CF}$ = 2.4 Hz, C1), 40.7 (dd, $^{2}J_{CF}$ = 24.9 Hz, $^{2}J_{CF}$ = 22.0 Hz, C2), 38.6 (dd, $^{2}J_{CF}$ = 27.3 Hz, $^{2}J_{CF}$ = 25.9 Hz, C4).

**GC-EI-MS:** Retention 10.26 min, (*m/z*) requires: [$(C_{16}H_{13}F_3)^+$] = 262.0964, (*m/z*) found: [$(C_{16}H_{13}F_3)^+$] = 262.0964.

**FT-IR** ($\tilde{\nu}$ = $cm^{-1}$)**:** 1604.2 (w), 1509.5 (s), 1453.6 (w), 1424.9 (w), 1367.5 (m), 1315.8 (w), 1278.5 (w), 1267.0 (w), 1224.0 (s), 1158.0 (m), 1123.5 (m), 1084.8 (s), 1067.6 (s), 1044.6 (s), 1024.5 (m), 955.7 (w), 879.4 (m), 862.4 (w), 829.4 (m), 819.3 (m), 774.9 (m), 741.9 (s), 718.9 (w), 707.4 (w).

**1-(4-Chlorophenyl)-3,3-difluoro-1,2,3,4-tetrahydronaphthalene (3p)**

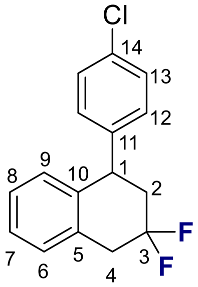


Compound **3p** was prepared according to general procedure **E** (amine:HF 1:5.0) using 3-(4-chlorophenyl)-1-phenylcyclobutan-1-ol **1p** (51.8 mg, 0.200 mmol). The crude product was purified by flash column chromatography (0-2% DCM in *n*-pentane) to yield the title compound as a colorless solid (31.8 mg, 0.114 mmol, 57%).

$\mathbf{R}_f$ = 0.39 (5% DCM in *n*-pentane).

**Melting Point:** 70-72 °C.

**$^1$H NMR** (500 MHz, $CDCl_3$): $\delta$ [ppm] = 7.33 (d, $^3J_{HH}$ = 8.5 Hz, 2H, H-C13), 7.21 (t, $^3J_{HH}$ = 7.4 Hz, 1H, H-C7), 7.17 – 7.08 (m, 4H, H-C6, H-C8, H-C12), 6.78 (d, $^3J_{HH}$ = 7.8 Hz, 1H, C9), 4.33 (dd, $^3J_{HH}$ = 11.7 Hz, $^3J_{HH}$ = 5.9 Hz, 1H, H-C1), 3.47 – 3.33 (m, 2H, H-C4), 2.56 (dtdd, $^2J_{HH}$ = 13.5 Hz, $^3J_{FH}$ = 8.2 Hz, $^3J_{HH}$ = 5.9 Hz, $J_{FH}$ = 2.4 Hz, 1H, H$^a$-C2), 2.25 (dddd, $^3J_{HF}$ = 31.5 Hz, $^2J_{HH}$ = 13.8 Hz, $^3J_{HH}$ = 11.7 Hz, $^3J_{HF}$ = 2.8 Hz, 1H, H$^b$-C2).

**$^{19}$F NMR** (376 MHz, $CDCl_3$): $\delta$ [ppm] = -91.48 (ddddd, $^2J_{FF}$ = 237.7 Hz, $^3J_{FH}$ = 10.4 Hz, $^3J_{FH}$ = 8.5 Hz, $^3J_{FH}$ = 5.0 Hz, $^3J_{FH}$ = 2.5 Hz, 1F, F$^a$-C3), -98.62 (ddddd, $^2J_{FF}$ = 238.4 Hz, $^3J_{FH}$ = 31.6 Hz, $^3J_{FH}$ = 27.2 Hz, $^3J_{FH}$ = 17.5 Hz, $^3J_{FH}$ = 7.6 Hz, 1F, F$^b$-C3).

**$^{19}$F{$^1$H} NMR** (376 MHz, $CDCl_3$): $\delta$ [ppm] = -91.45 (d, $^2J_{FF}$ = 237.9 Hz, 1F, F$^a$-C3), -98.64 (d, $^2J_{FF}$ = 237.9 Hz, 1F, F$^b$-C3).

**$^{13}$C{$^1$H} NMR** (126 MHz, $CDCl_3$): $\delta$ [ppm] = 142.7 (C11), 137.2 (d, $^4J_{CF}$ = 1.6 Hz, C10), 132.9 (C14), 132.1 (d, $^3J_{CF}$ = 9.2 Hz, C5), 130.3 (C12), 129.3 (d, $^5J_{CF}$ = 1.7 Hz, C9), 129.2 (C8), 129.1 (C13), 127.2 (C7), 127.1 (C6), 122.4 (dd, $^1J_{CF}$ = 242.1 Hz, $^1J_{CF}$ = 239.2 Hz, C3), 44.0 (dd, $^3J_{CF}$ = 8.1 Hz, $^3J_{CF}$ = 2.4 Hz, C1), 40.5 (dd, $^2J_{CF}$ = 24.8 Hz, $^2J_{CF}$ = 22.2 Hz, C2), 38.6 (dd, $^2J_{CF}$ = 27.3 Hz, $^2J_{CF}$ = 26.0 Hz, C4).

**GC-EI-MS:** Retention 9.14 min, (*m/z*) requires: [$(C_{16}H_{13}F_2Cl)^+$] = 278.0668, (*m/z*) found: [$(C_{16}H_{13}F_2Cl)^+$] = 278.0670.

**FT-IR** ($\tilde{\nu}$ = cm$^{-1}$)**:** 3006.2 (broad), 1489.4 (m), 1453.6 (w), 1424.9 (w), 1410.5 (w), 1366.0 (m), 1317.3 (m), 1275.6 (s), 1267.0 (s), 1261.1 (s), 1125.0 (m), 1086.2 (m), 1067.6 (m), 1043.2 (m), 954.2 (m), 876.7 (w), 822.2 (m), 800.7 (m), 641.4 (w), 609.8 (m), 749.0 (m).

**1-(4-Bromophenyl)-3,3-difluoro-1,2,3,4-tetrahydronaphthalene (3q)**

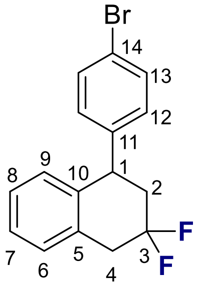


Compound **3q** was prepared according to general procedure **E** (amine:HF 1:5.0) using 3-(4-bromophenyl)-1-phenylcyclobutan-1-ol **1q** (60.6 mg, 0.200 mmol). The crude product was purified by flash column chromatography (4-7% DCM in cyclohexane) to yield the title compound as a colorless solid (40.2 mg, 0.124 mmol, 62%).

**$R_f$** = 0.44 (10% DCM in cyclohexane).

**Melting Point:** 88-90 ˚C.

**$^1$H NMR** (500 MHz, $CDCl_3$): $\delta$ [ppm] = 7.50 – 7.46 (m, 2H, H-C13), 7.24 – 7.19 (m, 1H, H-C7), 7.15 (d, $^3J_{HH}$ = 7.6 Hz, 1H, H-C6), 7.13 – 7.10 (m, 1H, H-C8), 7.10 – 7.06 (m, 2H, H-C12), 6.79 (d, $^3J_{HH}$ = 7.8 Hz, 1H, H-C9), 4.32 (dd, $^3J_{HH}$ = 11.9 Hz, $^3J_{HH}$ = 5.9 Hz, 1H, H-C1), 3.49 – 3.31 (m, 2H, H-C4), 2.56 (dtdd, $^2J_{HH}$ = 13.7 Hz, $^3J_{HF}$ = 8.3 Hz, $^3J_{HH}$ = 5.9 Hz, $J_{HH}$ = 2.4 Hz, 1H, $H^a$-C2), 2.24 (dddd, $^3J_{HF}$ = 31.5 Hz, $^2J_{HH}$ = 13.9 Hz, $^3J_{HH}$ = 11.7 Hz, $^3J_{HF}$ = 2.8 Hz, 1H, $H^b$-C2).

**$^{19}$F NMR** (376 MHz, $CDCl_3$): $\delta$ [ppm] = -91.51 (ddddd, $^2J_{FF}$ = 237.8 Hz, $^3J_{FH}$ = 10.5 Hz, $^3J_{FH}$ = 8.5 Hz, $^3J_{FH}$ = 5.3 Hz, $^3J_{FH}$ = 2.5 Hz, 1F, $F^a$-C3), -98.74 (ddddd, $^2J_{FF}$ = 237.9 Hz, $^3J_{FH}$ = 31.7 Hz, $^3J_{FH}$ = 28.1 Hz, $^3J_{FH}$ = 17.3 Hz, $^3J_{FH}$ = 7.7 Hz, 1F, $F^b$-C3).

**$^{19}$F{$^1$H} NMR** (376 MHz, $CDCl_3$): $\delta$ [ppm] = -91.51 (d, $^2J_{FF}$ = 237.8 Hz, 1F, $F^a$-C3), -98.74 (dd, $^2J_{FF}$ = 237.9 Hz, $F^b$-C3).

**$^{13}$C{$^1$H} NMR** (126 MHz, $CDCl_3$): $\delta$ [ppm] = 143.3 (C11), 137.1 (d, $^4J_{CF}$ = 1.7 Hz, C10), 132.2 (dd, $^3J_{CF}$ = 10.2 Hz, $^3J_{CF}$ = 1.1 Hz, C5), 132.1 (C13), 130.7 (C12), 129.3 (d, $^4J_{CF}$ = 1.8 Hz, C6), 129.2 (d, $^5J_{CF}$ = 1.0 Hz, C9), 127.2 (C7), 127.1 (C8), 122.4 (dd, $^1J_{CF}$ = 242.0 Hz, $^1J_{CF}$ = 239.2 Hz, C3), 121.0 (C14), 44.0 (dd, $^3J_{CF}$ = 8.1 Hz, $^3J_{CF}$ = 2.4 Hz, C1), 40.4 (dd, $^2J_{CF}$ = 24.8 Hz, $^2J_{CF}$ = 22.3 Hz, C2), 38.6 (dd, $^2J_{CF}$ = 27.3 Hz, $^2J_{CF}$ = 25.9 Hz, C4).

**GC-EI-MS:** Retention 10.26 min, (*m/z*) requires: $[(C_{16}H_{13}BrF_2)^+]$ = 322.0163, (*m/z*) found: $[(C_{16}H_{13}BrF_2)^+]$ = 322.0163.

**FT-IR** ($\tilde{\nu}$ = cm$^{-1}$)**:** 1486.6 (w), 1452.1 (w), 1424.9 (w), 1407.7 (w), 1364.6 (w), 1317.3 (w), 1278.5 (w), 1265.6 (w), 1236.9 (w), 1125.0 (m), 1103.5 (w), 1086.2 (m), 1066.1 (s), 1043.2 (m), 1024.5 (m), 1010.2 (m), 954.2 (m), 876.7 (w), 865.3 (w), 840.9 (w), 830.8 (w), 820.8 (m), 797.8 (w), 770.6 (m), 744.7 (s), 739.0 (s), 710.4 (w).

**3,3-Difluoro-1-(4-(trifluoromethyl)phenyl)-1,2,3,4-tetrahydronaphthalene (3r)**

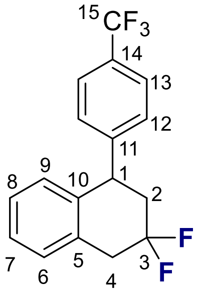


Compound **3r** was prepared according to general procedure **F** using 1-(1,3,3 trifluoro-4-phenylbutyl)-4-(trifluoromethyl)benzene **2r** (66.5 mg, 0.200 mmol). The reaction was stirred for 24 h at 40 ˚C. The crude product was purified by flash column chromatography (4-7% DCM in cyclohexane) to yield the title compound as a yellow solid (48.6 mg, 0.156 mmol, 78%).

**R*f*** = 0.41 (10% DCM in cyclohexane).

**Melting Point:** 52-54 ˚C.

**$^{1}$H NMR** (599 MHz, $CDCl_3$): $\delta$ [ppm] = 7.62 (d, $^{3}J_{HH}$ = 8.0 Hz, 2H, H-C13), 7.34 (d, $^{3}J_{HH}$ = 8.2 Hz, 2H, H-C12), 7.23 (t, $^{3}J_{HH}$ = 7.3 Hz, 1H, H-C7), 7.18 (dd, $^{3}J_{HH}$ = 7.9 Hz, $J_{HH}$ = 1.4 Hz, 1H, H-C6), 7.13 (td, $^{3}J_{HH}$ = 7.5 Hz, $J_{HH}$ = 1.5 Hz, 1H, H-C8), 6.77 (dt, $^{3}J_{HH}$ = 7.8 Hz, $J_{HH}$ = 1.3 Hz, 1H, H-C9), 4.44 (dd, $^{3}J_{HH}$ = 11.7 Hz, $^{3}J_{HH}$ = 6.0 Hz, 1H, H-C1), 3.50 – 3.34 (m, 2H, H-C4), 2.59 (ddddd, $^{2}J_{HH}$ = 13.5 Hz, $^{3}J_{HF}$ = 11.2 Hz, $^{3}J_{HF}$ = 7.7 Hz, $^{3}J_{HH}$ = 6.0 Hz, $J_{HH}$ = 2.2 Hz, 1H, $H^{a}$-C2), 2.29 (dddd, $^{3}J_{HF}$ = 31.0 Hz, $^{2}J_{HH}$ = 14.1 Hz, $^{3}J_{HH}$ = 11.7 Hz, $^{3}J_{HF}$ = 2.7 Hz, 1H, $H^{b}$-C2).

**$^{19}$F NMR** (564 MHz, $CDCl_3$): $\delta$ [ppm] = -62.46 (s, 3F, F-C15), -91.69 (ddddd, $^{2}J_{FF}$ = 238.4 Hz, $^{3}J_{FH}$ = 11.5 Hz, $^{3}J_{FH}$ = 8.9 Hz, $^{3}J_{FH}$ = 6.2 Hz, $^{3}J_{FH}$ = 2.7 Hz, 1F, $F^{a}$-C3), -98.62 (ddddd, $^{2}J_{FF}$ = 238.4 Hz, $^{3}J_{FH}$ = 31.1 Hz, $^{3}J_{FH}$ = 28.4 Hz, $^{3}J_{FH}$ = 16.2 Hz, $^{3}J_{FH}$ = 7.6 Hz, 1F, $F^{b}$-C3).

**$^{19}$F{$^{1}$H} NMR** (564 MHz, $CDCl_3$): $\delta$ [ppm] = -62.46 (s, 3F, F-C15), -91.69 (d, $^{2}J_{FF}$ = 238.4 Hz, 1F, $F^{a}$-C3), -98.61 (d, $^{2}J_{FF}$ = 238.4 Hz, $F^{b}$-C3).

**$^{13}$C{$^{1}$H} NMR** (151 MHz, $CDCl_3$): $\delta$ [ppm] = 148.4 (C11), 136.7 (d, $^{4}J_{CF}$ = 1.6 Hz, C10), 132.3 (dd, $^{3}J_{CF}$ = 9.9 Hz, $^{3}J_{CF}$ = 1.1 Hz, C5), 129.5 (q, $^{2}J_{CF}$ = 32.4 Hz, C14), 129.4 (d, $^{4}J_{CF}$ = 1.7 Hz, C6), 129.3 (C12), 129.2 (d, $^{5}J_{CF}$ = 1.0 Hz, C9), 127.3 (C7), 127.2 (C8), 125.9 (q, $^{3}J_{CF}$ = 3.8 Hz, C13), 124.3 (q, $^{1}J_{CF}$ = 272.0 Hz, C15), 122.3 (dd, $^{1}J_{CF}$ = 242.1 Hz, $^{1}J_{CF}$ = 239.2 Hz, C3), 44.4 (dd, $^{3}J_{CF}$ = 8.0 Hz, $^{3}J_{CF}$ = 2.5 Hz, C1), 40.3 (dd, $^{2}J_{CF}$ = 24.8 Hz, $^{2}J_{CF}$ = 22.6 Hz, C2), 38.6 (dd, $^{2}J_{CF}$ = 27.3 Hz, $^{2}J_{CF}$ = 25.9 Hz, C4).

**GC-EI-MS:** Retention 8.33 min, (*m/z*) requires: [$(C_{17}H_{13}F_5)^+$] = 312.0932, (*m/z*) found: [$(C_{17}H_{13}F_5)^+$] = 312.0932.

**FT-IR** ($\tilde{\nu}$ = $cm^{-1}$)**:** 1618.6 (w), 1493.8 (w), 1453.6 (w), 1445.0 (w), 1420.6 (w), 1368.9 (w), 1282.8 (w), 1268.5 (w), 1241.2 (w), 1208.2 (w), 1169.5 (w), 1156.5 (w), 1125.0 (w), 1109.2 (m), 1084.8 (s), 1064.7 (m), 1043.2 (s), 1018.8 (m), 980.1 (m), 949.9 (w), 876.7 (w), 848.0 (m), 829.4 (m), 809.3 (w), 777.7 (w), 747.6 (m), 737.6 (m), 708.9 (w).

**4-(3,3-Difluoro-1,2,3,4-tetrahydronaphthalen-1-yl)phenyl trifluoromethane-sulfonate (3s)**

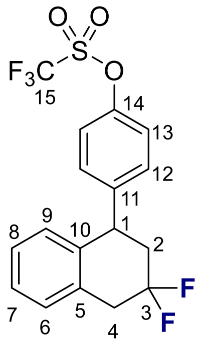

Compound **3s** was prepared according to modified general procedure **E** (amine:HF 1:5.5) using 4-(3-hydroxy-3-phenylcyclobutyl)phenyl trifluoromethanesulfonate **1s** (74.5 mg, 0.200 mmol). The reaction was stirred for 72 h after the addition of HFIP. The crude product was purified by flash column chromatography (5-15% DCM in cyclohexane) to yield the title compound as a colorless solid (43.5 mg, 0.111 mmol, 56%).

**R*f*** = 0.26 (20% DCM in cyclohexane).

**Melting Point:** 67-69 ˚C.

**$^{1}$H NMR** (599 MHz, $CDCl_3$): $\delta$ [ppm] = 7.30 – 7.24 (m, 4H, H-C12, H-C13), 7.23 (t, $^{3}J_{HH}$ = 7.4 Hz, 1H, H-C7), 7.17 (dd, $^{3}J_{HH}$ = 7.6 Hz, $J_{HH}$ = 1.3 Hz, 1H, H-C6), 7.13 (t, $^{3}J_{HH}$ = 7.5 Hz, 1H, H-C8), 6.76 (dt, $^{3}J_{HH}$ = 7.9 Hz, $J_{HH}$ = 1.3 Hz, 1H, H-C9), 4.41 (dd, $^{3}J_{HH}$ = 11.6 Hz, $^{3}J_{HH}$ = 6.0 Hz, 1H, H-C1), 3.47 – 3.33 (m, 2H, H-C4), 2.58 (ddddd, $^{2}J_{HH}$ = 13.5 Hz, $^{3}J_{HF}$ = 11.4 Hz, $^{3}J_{HF}$ = 7.6 Hz, $^{3}J_{HH}$ = 6.0 Hz, $J_{HH}$ = 2.2 Hz, 1H, $H^{a}$-C2), 2.25 (dddd, $^{3}J_{HF}$ = 30.8 Hz, $^{2}J_{HH}$ = 13.9 Hz, $^{3}J_{HH}$ = 11.6 Hz, $^{3}J_{HF}$ = 2.9 Hz, 1H, $H^{b}$-C2).

**$^{19}$F NMR** (564 MHz, $CDCl_3$): $\delta$ [ppm] = -72.89 (s, 3F, F-C15), -91.75 (dddddd, $^{2}J_{FF}$ = 238.6 Hz, $^{3}J_{FH}$ = 10.9 Hz, $^{3}J_{FH}$ = 8.9 Hz, $^{3}J_{FH}$ = 6.0 Hz, $^{3}J_{FH}$ = 2.4 Hz, $J_{FH}$ = 1.5 Hz, 1F, $F^{a}$-C3), -98.54 (ddddd, $^{2}J_{FF}$ = 238.5 Hz, $^{3}J_{FH}$ = 30.9 Hz, $^{3}J_{FH}$ = 27.7 Hz, $^{3}J_{FH}$ = 16.5 Hz, $^{3}J_{FH}$ = 7.5 Hz, 1F, $F^{b}$-C3).

**$^{19}$F{$^{1}$H} NMR** (564 MHz, $CDCl_3$): $\delta$ [ppm] = -72.89 (s, 3F, F-C15), -91.75 (d, $^{2}J_{FF}$ = 238.6 Hz, 1F, $F^{a}$-C3), -98.54 (d, $^{2}J_{FF}$ = 238.5 Hz, $F^{b}$-C3).

**$^{13}$C{$^{1}$H} NMR** (151 MHz, $CDCl_3$): $\delta$ [ppm] = 148.6 (C14), 144.9 (C11), 136.6 (C10), 132.2 (d, $^{3}J_{CF}$ = 9.8 Hz, C5), 130.7 (C12), 129.4 (C6), 129.2 (C9), 127.4 (C7), 127.2 (C8), 122.3 (dd, $^{1}J_{CF}$ = 242.0 Hz, $^{1}J_{CF}$ = 239.2 Hz, C3), 121.9 (C13), 118.9 (q, $^{1}J_{CF}$ = 320.7 Hz, C15), 44.0 (dd, $^{3}J_{CF}$ = 7.9 Hz, $^{3}J_{CF}$ = 2.7 Hz, C1), 40.5 (dd, $^{2}J_{CF}$ = 24.8 Hz, $^{2}J_{CF}$ = 22.6 Hz, C2), 38.6 (t, $^{2}J_{CF}$ = 26.6 Hz, C4).

**GC-EI-MS:** Retention 9.36 min, (*m/z*) requires: $[(C_{17}H_{13}F_5O_3S)^{+}]$ = 392.0500, (*m/z*) found: $[(C_{17}H_{13}F_5O_3S)^{+}]$ = 392.0498.

**FT-IR** ($\tilde{\nu}$ = cm$^{-1}$)**:** 1498.1 (m), 1417.7 (s), 1368.9 (w), 1315.8 (w), 1279.9 (w), 1268.5 (w), 1249.8 (m), 1205.3 (s), 1180.9 (m), 1135.0 (s), 1125.0 (s), 1087.7 (m), 1069.0 (s), 1044.6 (m), 1024.5 (w), 1017.4 (m), 955.7 (w), 945.6 (w), 883.9 (s), 878.2 (s), 846.6 (m), 832.3 (m), 807.9 (w), 776.3 (m), 747.6 (m), 733.2 (m), 720.3 (m), 704.5 (w).

**7-Bromo-1-(4-fluorophenyl)-3,3-difluoro-1,2,3,4-tetrahydronaphthalene (3t)**

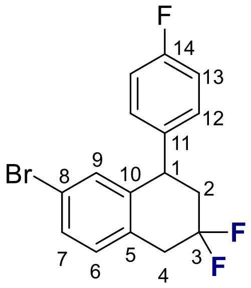


Compound **3t** was prepared according to general procedure **E** (Amine:HF 1:5.0) using 1-(4-bromophenyl)-3-(4-fluorophenyl)cyclobutan-1-ol **1t** (64.2 mg, 0.200 mmol). The crude product was purified by flash column chromatography (4-7% DCM in cyclohexane) to yield the title compound as a colorless oil (39.0 mg, 0.114 mmol, 57%).

**$R_f$** = 0.36 (10% DCM in cyclohexane).

**$^1$H NMR** (599 MHz, $CDCl_3$): $\delta$ [ppm] = 7.32 (ddd, $^3J_{HH}$ = 8.2 Hz, $^4J_{HH}$ = 2.1 Hz, $J_{HH}$ = 0.9 Hz, 1H, H-C7), 7.14 (ddd, $^3J_{HH}$ = 8.3 Hz, $^4J_{HF}$ = 5.3 Hz, $^4J_{HH}$ = 2.6 Hz, 1H, H-C12), 7.09 – 7.04 (m, 2H, H-C13), 7.02 (dd, $^3J_{HH}$ = 8.2 Hz, $J_{HH}$ = 1.0 Hz, 1H, H-C6), 6.91 (dd, $^4J_{HH}$ = 2.0 Hz, $J_{HH}$= 1.0 Hz, 1H, H-C9), 4.29 (dd, $^3J_{HH}$ = 12.0 Hz, $^3J_{HH}$ = 5.9 Hz, 1H, H-C1), 3.40 – 3.27 (m, 2H, H-C4), 2.54 (dtdd, $^2J_{HH}$ = 13.8 Hz, $^3J_{HF}$ = 8.4 Hz, $^3J_{HH}$ = 6.0 Hz, $J_{HF}$ = 2.9 Hz, 1H, $H^a$-C2), 2.22 (dddd, $^3J_{HF}$ = 31.5 Hz, $^2J_{HH}$ = 14.4 Hz, $^3J_{HH}$ = 12.0 Hz, $^3J_{HF}$ = 2.9 Hz, 1H, $H^b$-C2).

**$^{19}$F NMR** (376 MHz, $CDCl_3$): $\delta$ [ppm] = -91.84 (dddddd, $^2J_{FF}$ = 238.9 Hz, $^3J_{FH}$ = 10.4 Hz, $^3J_{FH}$ = 8.3 Hz, $^3J_{FH}$ = 5.8 Hz, $^3J_{FH}$ = 2.7 Hz, $J_{FH}$ = 1.8 Hz, 1F, $F^a$-C3), -98.75 (ddddd, $^2J_{FF}$ = 238.9 Hz, $^3J_{FH}$ = 31.7 Hz, $^3J_{FH}$ = 25.9 Hz, $^3J_{FH}$ = 19.1 Hz, $^3J_{FH}$ = 7.9 Hz, 1F, $F^b$-C3), -115.15 (tt, $^3J_{FH}$ = 8.6 Hz, $^4J_{FH}$ = 5.3 Hz, 1F, $F_b$-C14).

**$^{19}$F{$^1$H} NMR** (376 MHz, $CDCl_3$): $\delta$ [ppm] = -91.84 (d, $^2J_{FF}$ = 238.9 Hz, 1F, $F^a$-C3), -98.75 (d, $^2J_{FF}$ = 237.2 Hz, $F^b$-C3), -115.15 (s, F-C14).

**$^{13}$C{$^1$H} NMR** (151 MHz, $CDCl_3$): $\delta$ [ppm] = 162.1 (d, $^1J_{CF}$ = 246.1 Hz, C14), 139.8 (d, $^4J_{CF}$ = 1.6 Hz, C10), 138.9 (d, $^4J_{CF}$ = 3.2 Hz, C11), 132.0 (d, $^5J_{CF}$ = 1.1 Hz, C9), 131.1 (d, $^3J_{CF}$ = 10.2 Hz, C5), 130.8 (d, $^4J_{CF}$ = 1.9 Hz, C6), 130.4 (C7), 130.3 (d, $^3J_{CF}$ = 3.9 Hz, C12), 122.1 (dd, $^1J_{CF}$ = 242.4 Hz, $^1J_{CF}$ = 239.3 Hz, C3), 121.0 (C8), 116.1 (d, $^2J_{CF}$ = 21.4 Hz, C13), 43.7 (dd, $^3J_{CF}$ = 8.3 Hz, $^3J_{CF}$ = 2.3 Hz, C1), 40.4 (dd, $^2J_{CF}$ = 25.3 Hz, $^2J_{CF}$ = 21.8 Hz, C2), 38.2 (dd, $^2J_{CF}$ = 27.5 Hz, $^2J_{CF}$ = 26.5 Hz, C4).

**GC-EI-MS:** Retention 9.38 min, (*m/z*) requires: [$(C_{16}H_{12}BrF_3)^+$] = 340.0069, (*m/z*) found: [$(C_{16}H_{12}BrF_3)^+$] = 340.0066.

**FT-IR** ($\tilde{\nu}$ = $cm^{-1}$)**:** 2938.7 (w), 1605.7 (w), 1592.8 (w), 1509.5 (s), 1480.8 (m), 1446.3 (w), 1424.9 (w), 1403.4 (w), 1361.7 (m), 1310.1 (w), 1269.9 (m), 1224.0 (s), 1202.5 (w), 1176.6 (w), 1159.4 (m), 1127.8 (m), 1092.0 (m), 1080.5 (m), 1066.1 (s), 1030.3 (s), 1015.9 (m), 957.1 (m), 878.2 (s), 839.4 (s), 820.8 (m), 805.0 (s), 774.9 (m), 744.7 (w), 721.8 (w), 707.4 (w).

**7-Bromo-1-(4-chlorophenyl)-3,3-difluoro-1,2,3,4-tetrahydronaphthalene (3u)**

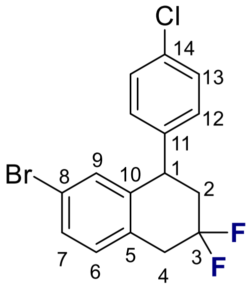


Compound **3u** was prepared according to general procedure **E** (amine:HF 1:5.5) using 1-(4-bromophenyl)-3-(4-chlorophenyl)cyclobutan-1-ol **1u** (67.5 mg, 0.200 mmol). The crude product was purified by flash column chromatography (4-7% DCM in cyclohexane) to yield the title compound as a yellow oil (50.3 mg, 0.141 mmol, 71%).

**$R_f$** = 0.44 (10% DCM in cyclohexane).

**$^1$H NMR** (500 MHz, $CDCl_3$): $\delta$ [ppm] = 7.36 – 7.31 (m, 3H, H-C7, H-C13), 7.15 – 7.10 (m, 2H, H-C12), 7.03 (d, $^3J_{HH}$ = 8.2 Hz, 1H, H-C6), 6.91 (dd, $^4J_{HH}$ = 2.0 Hz, $J_{HH}$= 1.1 Hz, 1H, H-C9), 4.28 (dd, $^3J_{HH}$ = 12.0 Hz, $^3J_{HH}$ = 5.9 Hz, 1H, H-C1), 3.39 – 3.26 (m, 2H, H-C4), 2.54 (dtdd, $^2J_{HH}$ = 13.6 Hz, $^3J_{HF}$ = 8.0 Hz, $^3J_{HH}$ = 5.9 Hz, $J_{HF}$ = 2.1 Hz, 1H, $H^a$-C2), 2.21 (dddd, $^3J_{HF}$ = 31.5 Hz, $^2J_{HH}$ = 14.3 Hz, $^3J_{HH}$ = 11.9 Hz, $^3J_{HF}$ = 2.8 Hz, 1H, $H^b$-C2).

**$^{19}$F NMR** (376 MHz, $CDCl_3$): $\delta$ [ppm] = -91.93 (dddddd, $^2J_{FF}$ = 239.1 Hz, $^3J_{FH}$ = 10.5 Hz, $^3J_{FH}$ = 8.2 Hz, $^3J_{FH}$ = 6.0 Hz, $^3J_{FH}$ = 2.6 Hz, $J_{FH}$ = 1.6 Hz, 1F, $F^a$-C3), -98.77 (ddddd, $^2J_{FF}$ = 239.1 Hz, $^3J_{FH}$ = 31.6 Hz, $^3J_{FH}$ = 25.7 Hz, $^3J_{FH}$ = 19.2 Hz, $^3J_{FH}$ = 7.8 Hz, 1F, $F^b$-C3).

**$^{19}$F{$^1$H} NMR** (376 MHz, $CDCl_3$): $\delta$ [ppm] = -91.93 (d, $^2J_{FF}$ = 239.1 Hz, 1F, $F^a$-C3), -98.77 (d, $^2J_{FF}$ = 239.1 Hz, $F^b$-C3).

**$^{13}$C{$^1$H} NMR** (126 MHz, $CDCl_3$): $\delta$ [ppm] = 141.8 (C11), 139.5 (d, $^4J_{CF}$ = 1.4 Hz, C10), 133.3 (C14), 132.0 (d, $^5J_{CF}$ = 1.1 Hz, C9), 131.2 (d, $^3J_{CF}$ = 10.5 Hz, C5), 130.8 (d, $^4J_{CF}$ = 1.8 Hz, C6), 130.4 (C7), 130.2 (C12), 129.4 (C13), 122.0 (dd, $^1J_{CF}$ = 242.4 Hz, $^1J_{CF}$ = 239.3 Hz, C3), 121.0 (C8), 43.8 (dd, $^3J_{CF}$ = 8.3 Hz, $^3J_{CF}$ = 2.2 Hz, C1), 40.2 (dd, $^2J_{CF}$ = 24.8 Hz, $^2J_{CF}$ = 22.6 Hz, C2), 38.2 (t, $^2J_{CF}$ = 26.7 Hz, C4).

**GC-EI-MS:** Retention 10.87 min, (*m/z*) requires: [$(C_{16}H_{12}BrClF_2)^+$] = 355.9773, (*m/z*) found: [$(C_{16}H_{12}BrClF_2)^+$] = 355.9772.

**FT-IR** ($\tilde{\nu}$ = cm$^{-1}$)**:** 1592.8 (w), 1489.4 (m), 1446.4 (w), 1424.9 (w), 1401.9 (w), 1360.3 (w), 1310.1 (w), 1267.0 (m), 1236.9 (w), 1201.0 (w), 1176.6 (w), 1127.8 (m), 1090.5 (s), 1081.9 (m), 1064.7 (s), 1030.3 (m), 1014.5 (m), 957.1 (m), 875.3 (s), 828.0 (m), 799.3 (m), 782.0 (w), 759.1 (w), 718.9 (w).

**7-Bromo-3,3-difluoro-1-(4-(trifluoromethyl)phenyl)-1,2,3,4-tetrahydronaphthalene (3v)**

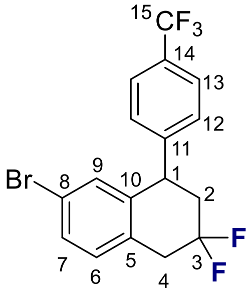


Compound **3v** was prepared according to general procedure **F** using 1-bromo-4-(2,2,4-trifluoro-4-(4-(trifluoromethyl)phenyl)butyl)benzene **2v** (88.2 mg, 0.200 mmol). The reaction was stirred for 72 h at 40 ˚C The crude product was purified by flash column chromatography (4-7% DCM in cyclohexane) to yield the title compound as a yellow solid (76.6 mg, 0.196 mmol, 98%).

**$R_f$** = 0.40 (10% DCM in cyclohexane).

**Melting Point:** 86-88 ˚C.

**$^{1}$H NMR** (500 MHz, $CDCl_3$): $\delta$ [ppm] = 7.64 (d, $^3J_{HH}$ = 8.1 Hz, 2H, H-C13), 7.35 (dd, $^3J_{HH}$ = 8.4 Hz, $^4J_{HH}$ = 2.1 Hz, 1H, H-C7), 7.31 (d, $^3J_{HH}$ = 8.0 Hz, 2H, H-C12), 7.05 (d, $^3J_{HH}$ = 8.2 Hz, 1H, H-C6), 6.90 (dd, $^4J_{HH}$ = 2.1 Hz, $J_{HH}$ = 1.1 Hz, 1H, H-C9), 4.39 (dd, $^3J_{HH}$ = 11.7 Hz, $^3J_{HH}$ = 6.0 Hz, 1H, H-C1), 3.44 – 3.25 (m, 2H, H-C4), 2.56 (dtdd, $^2J_{HH}$ = 13.6 Hz, $^3J_{HF}$ = 8.2 Hz, $^3J_{HH}$ = 6.2 Hz, $J_{HH}$ = 2.1 Hz, 1H, $H^a$-C2), 2.25 (dddd, $^3J_{HF}$ = 31.2 Hz, $^2J_{HH}$ = 13.8 Hz, $^3J_{HH}$ = 12.8 Hz, $^3J_{HF}$ = 2.6 Hz, 1H, $H^b$-C2).

**$^{19}$F NMR** (470 MHz, $CDCl_3$): $\delta$ [ppm] = -62.51 (s, 3F, F-C15), -92.20 (dddddd, $^2J_{FF}$ = 239.5 Hz, $^3J_{FH}$ = 10.6 Hz, $^3J_{FH}$ = 8.6 Hz, $^3J_{FH}$ = 6.1 Hz, $^3J_{FH}$ = 2.6 Hz, $J_{FH}$ = 1.9 Hz, 1F, $F^a$-C3), -98.76 (ddddd, $^2J_{FF}$ = 239.6 Hz, $^3J_{FH}$ = 31.1 Hz, $^3J_{FH}$ = 26.1 Hz, $^3J_{FH}$ = 18.6 Hz, $^3J_{FH}$ = 7.5 Hz, 1F, $F^b$-C3).

**$^{19}$F{$^1$H} NMR** (470 MHz, $CDCl_3$): $\delta$ [ppm] = -62.51 (s, 3F, F-C15), -92.20 (d, $^2J_{FF}$ = 239.5 Hz, 1F, $F^a$-C3), -98.76 (d, $^2J_{FF}$ = 239.6 Hz, $F^b$-C3).

**$^{13}$C{$^1$H} NMR** (126 MHz, $CDCl_3$): $\delta$ [ppm] = 147.4 (C11), 138.9 (C10), 132.0 (C9), 131.3 (d, $^3J_{CF}$ = 10.3 Hz, C5), 131.0 (d, $^4J_{CF}$ = 1.8 Hz, C6), 130.6 (C7), 129.9 (q, $^2J_{CF}$ = 32.7 Hz, C14), 129.2 (C12), 126.2 (q, $^3J_{CF}$ = 3.8 Hz, C13), 124.2 (q, $^1J_{CF}$ = 272.1 Hz, C15), 121.9 (dd, $^1J_{CF}$ = 242.4 Hz, $^1J_{CF}$ = 239.3 Hz, C3), 121.1 (C8), 44.3 (dd, $^3J_{CF}$ = 8.1 Hz, $^3J_{CF}$ = 2.5 Hz, C1), 40.1 (dd, $^2J_{CF}$ = 24.7 Hz, $^2J_{CF}$ = 22.8 Hz, C2), 38.2 (t, $^2J_{CF}$ = 27.0 Hz, C4).

**GC-EI-MS:** Retention 9.26 min, (*m/z*) requires: [$(C_{17}H_{12}BrF_5)^+$] = 390.0037, (*m/z*) found: [$(C_{17}H_{12}BrF_5)^+$] = 390.0037.

**FT-IR** ($\tilde{\nu}$ = $cm^{-1}$)**:** 1618.6 (w), 1592.8 (w), 1480.8 (w), 1446.4 (w), 1419.1 (w), 1401.9 (w), 1356.0 (w), 1324.4 (m), 1314.4 (m), 1271.3 (w), 1239.8 (w), 1191.0 (w), 1163.7 (s), 1116.4 (s), 1097.7 (m), 1080.5 (m), 1066.1 (s), 1031.7 (s), 1017.4 (m), 958.5 (w), 949.9 (w), 888.2 (w), 878.2 (m), 840.9 (m), 835.1 (m), 800.7 (s), 784.9 (w), 760.5 (w), 710.3 (w).

**4-(7-Bromo-3,3-difluoro-1,2,3,4-tetrahydronaphthalen-1-yl)phenyl trifluoromethanesulfonate (3w)**

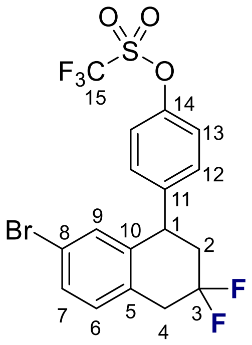


Compound **3w** was prepared according to modified general procedure **F** using (4-(4-bromophenyl)-1,3,3-trifluorobutyl)phenyl trifluoromethanesulfonate **2w** (98.2 mg, 0.200 mmol). The reaction was stirred for 72 h at room temperature. The crude product was purified by flash column chromatography (10% DCM in cyclohexane) to yield the title compound as a yellow solid (70.8 mg, 0.150 mmol, 75%).

**R*f*** = 0.18 (15% DCM in cyclohexane).

**Melting Point:** 67-69 ˚C.

**$^{1}$H NMR** (500 MHz, $CDCl_3$): $\delta$ [ppm] = 7.35 (ddd, $^{3}J_{HH}$ = 8.3 Hz, $^{4}J_{HH}$ = 2.1 Hz, $J_{HH}$ = 0.9 Hz, 1H, H-C7), 7.31 – 7.25 (m, 4H, H-C12, H-C13), 7.05 (dd, $^{3}J_{HH}$ = 8.0 Hz, $J_{HH}$ = 1.0 Hz, 1H, H-C6), 6.90 (dd, $^{4}J_{HH}$ = 2.1 Hz, $J_{HH}$ = 1.1 Hz, 1H, H-C9), 4.37 (dd, $^{3}J_{HH}$ = 11.6 Hz, $^{3}J_{HH}$ = 6.1 Hz, 1H, H-C1), 3.40 – 3.28 (m, 2H, H-C4), 2.57 (dtdd, $^{2}J_{HH}$ = 13.8 Hz, $^{3}J_{HF}$ = 8.4 Hz, $^{3}J_{HH}$ = 6.2 Hz, $J_{HH}$ = 2.0 Hz, 1H, $H^{a}$-C2), 2.25 (dddd, $^{3}J_{HF}$ = 30.7 Hz, $^{2}J_{HH}$ = 13.8 Hz, $^{3}J_{HH}$ = 11.7 Hz, $^{3}J_{HF}$ = 2.7 Hz, 1H, $H^{b}$-C2).

**$^{19}$F NMR** (470 MHz, $CDCl_3$): $\delta$ [ppm] = -72.87 (s, 3F, F-C15), -92.23 (dddddd, $^{2}J_{FF}$ = 239.8 Hz, $^{3}J_{FH}$ = 11.0 Hz, $^{3}J_{FH}$ = 8.4 Hz, $^{3}J_{FH}$ = 6.2 Hz, $^{3}J_{FH}$ = 2.7 Hz, $J_{FH}$ = 1.8 Hz, 1F, $F^{a}$-C3), -98.65 (ddddd, $^{2}J_{FF}$ = 239.7 Hz, $^{3}J_{FH}$ = 30.6 Hz, $^{3}J_{FH}$ = 25.9 Hz, $^{3}J_{FH}$ = 18.2 Hz, $^{3}J_{FH}$ = 7.5 Hz, 1F, $F^{b}$-C3).

**$^{19}$F{$^{1}$H} NMR** (470 MHz, $CDCl_3$): $\delta$ [ppm] = -72.87 (s, 3F, F-C15), -92.23 (d, $^{2}J_{FF}$ = 239.8 Hz, 1F, $F^{a}$-C3), -98.65 (d, $^{2}J_{FF}$ = 239.7 Hz, $F^{b}$-C3).

**$^{13}$C{$^{1}$H} NMR** (126 MHz, $CDCl_3$): $\delta$ [ppm] = 148.8 (C14), 143.9 (C11), 138.8 (C10), 132.0 (C9), 131.2 (d, $^{3}J_{CF}$ = 10.3 Hz, C5), 131.0 (d, $^{4}J_{CF}$ = 1.8 Hz, C6), 130.6 (C7 and C12), 122.1 (C13), 121.8 (dd, $^{1}J_{CF}$ = 242.5 Hz, $^{1}J_{CF}$ = 239.4 Hz, C3), 121.8 (C8), 118.9 (q, $^{1}J_{CF}$ = 320.8 Hz, C15), 43.8 (dd, $^{3}J_{CF}$ = 7.9 Hz, $^{3}J_{CF}$ = 2.7 Hz, C1), 40.2 (dd, $^{2}J_{CF}$ = 24.8 Hz, $^{2}J_{CF}$ = 22.8 Hz, C2), 38.1 (t, $^{2}J_{CF}$ = 27.0 Hz, C4).

**ESI-MS:** (*m/z*) requires: [$(C_{17}H_{12}BrF_5O_3SNa)^+$] = 492.9503, (*m/z*) found: [$(C_{17}H_{12}BrF_5O_3SNa)^+$] = 492.9506.

**FT-IR** ($\tilde{\nu}$ = $cm^{-1}$)**:** 1591.3 (w), 1499.5 (m), 1480.8 (w), 1446.4 (w), 1416.3 (s), 1357.4 (w), 1317.3 (w), 1271.3 (w), 1249.8 (m), 1206.8 (s), 1139.3 (s), 1094.8 (m), 1080.5 (m), 1066.1 (m), 1031.7 (m), 1017.4 (m), 958.5 (m), 941.3 (w), 896.8 (s), 878.2 (s), 850.9 (m), 835.1 (m), 803.6 (s), 783.5 (w), 774.9 (w), 739.0 (m), 721.8 (m), 706.0 (w).

**3-(6,6-Difluoro-8-phenyl-5,6,7,8-tetrahydronaphthalen-2-yl)pyridine (3y)**

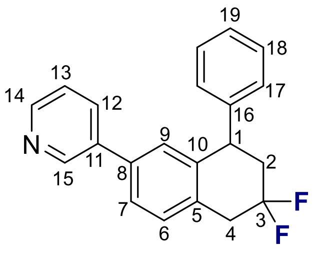


Compound **3y** was prepared according to modified general procedure **F** using 3-(4-(1,3,3-trifluoro-4-phenylbutyl)phenyl)pyridine **2y** (68.2 mg, 0.200 mmol). The reaction was stirred for 24 h at 40 ˚C. The crude product was purified by flash column chromatography (0-10% EtOAc in DCM) to yield the title compound as a colorless solid (43.7 mg, 0.136 mmol, 68%).

**R*f*** = 0.41 (10% EtOAc in DCM).

**Melting Point:** 64-66 °C.

**$^1$H NMR** (500 MHz, $CDCl_3$): $\delta$ [ppm] = 8.64 (dd, $^4J_{HH}$ = 2.4 Hz, $J_{HH}$ = 0.9 Hz, 1H, H-C15), 8.51 (dd, $^3J_{HH}$ = 4.8 Hz, $J_{HH}$ = 1.6 Hz, 1H, H-C14), 7.66 (ddd, $^3J_{HH}$ = 7.9 Hz, $^4J_{HH}$ = 2.4 Hz, $J_{HH}$ = 1.7 Hz, 1H, H-C12), 7.42 (dd, $^3J_{HH}$ = 7.9 Hz, $^4J_{HH}$ = 2.1 Hz, 1H, H-C7), 7.37 (t, $^3J_{HH}$ = 7.3 Hz, 2H, H-C18), 7.32 – 7.22 (m, 5H, H-C6, H-C13, H-C17, H-C19), 7.02 – 7.01 (m, 1H, H-C9), 4.40 (dd, $^3J_{HH}$ = 12.0 Hz, $^3J_{HH}$ = 6.1 Hz, 1H, H-C1), 3.52 – 3.40 (m, 2H, H-C4), 2.62 (dtdd, $^2J_{HH}$ = 13.8 Hz, $^3J_{HF}$ = 8.2 Hz, $^3J_{HH}$ = 6.0 Hz, $J_{HH}$ = 2.5 Hz, 1H, $H^a$-C2), 2.33 (dddd, $^3J_{FH}$ = 32.4 Hz, $^2J_{HH}$ = 13.7 Hz, $^3J_{HH}$ = 11.9 Hz, $^3J_{FH}$ = 2.8 Hz, 1H, $H^b$-C2).

**$^{19}$F NMR** (470 MHz, $CDCl_3$): $\delta$ [ppm] = -91.54 (dddddd, $^2J_{FF}$ = 237.8 Hz, $^3J_{FH}$ = 10.0 Hz, $^3J_{FH}$ = 8.1 Hz, $^3J_{FH}$ =4.7 Hz, $^3J_{FH}$ = 2.7 Hz, $J_{FH}$ = 1.8 Hz, 1F, $F^a$-C3), -98.76 (ddddd, $^2J_{FF}$ = 237.9 Hz, $^3J_{FH}$ = 32.3 Hz, $^3J_{FH}$ = 28.4 Hz, $^3J_{FH}$ = 17.0 Hz, $^3J_{FH}$ =8.0 Hz, 1F, $F^b$-C3).

**$^{19}$F{$^1$H} NMR** (470 MHz, $CDCl_3$): $\delta$ [ppm] = -91.54 (d, $^2J_{FF}$ = 237.9 Hz, 1F, $F^a$-C3), -98.78 (d, $^2J_{FF}$ = 237.9 Hz, 1F, $F^b$-C3).

**$^{13}$C{$^1$H} NMR** (126 MHz, $CDCl_3$): $\delta$ [ppm] = 148.6 (C15), 148.4 (C14), 143.7 (C16), 138.7 (C10), 136.6 (C8), 136.2 (C11), 134.3 (C12), 132.2 (d, $^3J_{CF}$ = 10.3 Hz, C5), 129.9 (C6), 129.1 (C18), 128.9 (C17), 128.0 (C9), 127.3 (C19), 125.7 (C7), 123.6 (C13), 122.5 (dd, $^1J_{CF}$ = 242.4 Hz, $^1J_{CF}$ = 239.0 Hz, C3), 44.6 (dd, $^3J_{CF}$ = 8.6 Hz, $^3J_{CF}$ = 2.0 Hz, C1), 40.6 (dd, $^2J_{CF}$ = 22.8 Hz, $^2J_{CF}$ = 22.2 Hz, C2), 38.5 (t, $^2J_{CF}$ = 26.9 Hz, C4)

**ESI-MS:** (*m/z*) requires: [$(C_{21}H_{17}NF_2Na)^+$] = 344.1221, (*m/z*) found: [$(C_{21}H_{17}NF_2Na)^+$] = 344.1220.

**FT-IR** ($\tilde{\nu}$ = $cm^{-1}$)**:** 2325.8 (broad), 1575.5 (w), 1495.2 (w), 1472.2 (m), 1455.0 (w), 1427.7 (w), 1390.4 (w), 1367.5 (m), 1335.9 (m), 1320.1 (m), 1298.6 (m), 1265.6 (m), 1244.1 (w), 1176.6 (w), 1130.7 (m), 1094.8 (m), 1079.1 (m), 1064.7 (m), 1037.4 (m), 1015.9 (m), 1001.6 (m), 958.5 (m), 914.0 (w), 892.5 (w), 883.9 (m), 845.2 (w), 833.7 (m), 799.3 (s), 790.6 (s), 761.9 (s), 728.9 (m), 711.7 (s), 701.7 (s).

## Stepwise Synthesis of 3c from *major*-1c

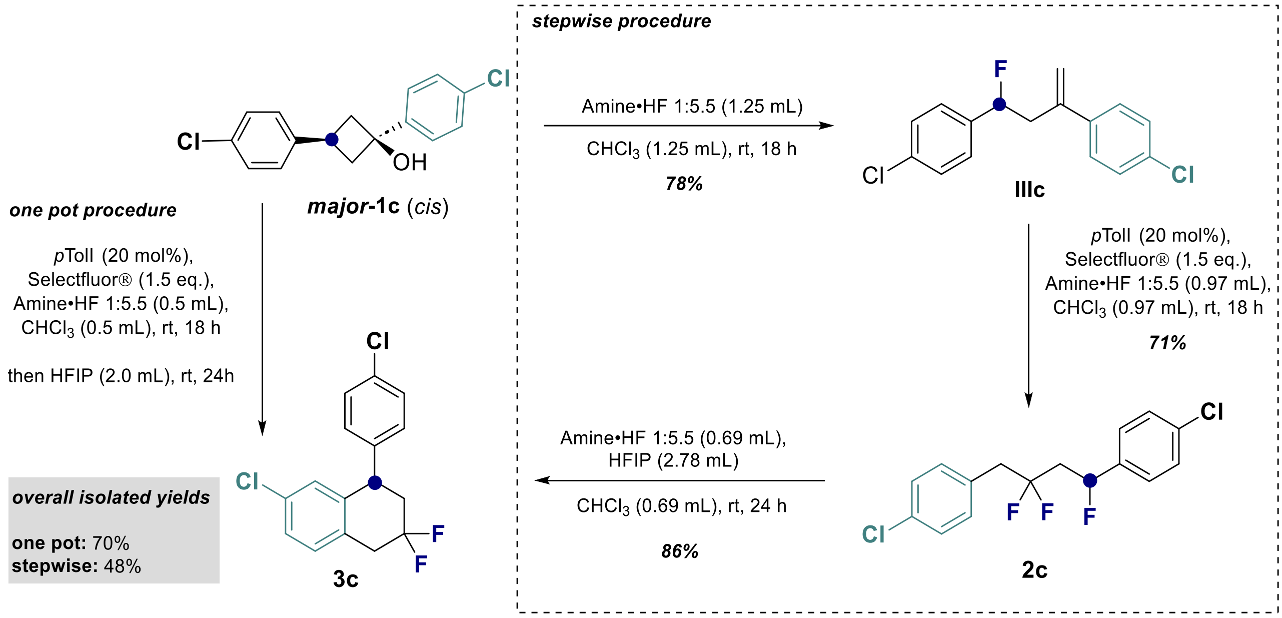


**Supplementary Figure 1.** One pot versus stepwise synthesis of tetralin **3c**.

### 4,4'-(1-Fluorobut-3-ene-1,3-diyl)bis(chlorobenzene) (IIIc)

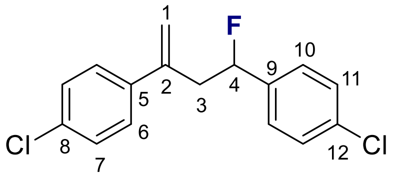

Compound **IIIc** was prepared according to modified general procedure **D** using 1,3-bis(4-chlorophenyl)cyclobutan-1-ol ***major*-1c** (146.6 mg, 0.500 mmol). No Selectfluor® and no *p*Toll was added to the reaction mixture and the amount of solvent and HF source was adapted on the scale. The crude product was purified by flash column chromatography (4-7% DCM in cyclohexane) to yield the title compound as a colorless solid (114.9 mg, 0.389 mmol, 78%).

***Caution:*** *Olah's reagent is highly toxic and corrosive. Direct exposure should be avoided. In the case of skin exposure, immediate treatment of the affected skin area with calcium gluconate gel is necessary to prevent serious chemical burns.*

**$R_f$** = 0.41 (10% DCM in cyclohexane).

**Melting Point:** 52-54 ˚C.

**$^1$H NMR** (500 MHz, $CDCl_3$): $\delta$ [ppm] = 7.36 – 7.29 (m, 6H, H-C6, H-C7, H-C11), 7.24 – 7.19 (m, 2H, H-C10), 5.45 (ddd, $^2J_{HF}$ = 47.1 Hz, $^3J_{HH}$ = 8.2 Hz, $^3J_{HH}$ = 5.2 Hz, 1H, H-C4), 5.36 (s, $H^a$-C1), 5.14 (s, $H^b$-C1), 3.15 (tdd, $^2J_{HH}$ = $^3J_{HF}$ = 14.9 Hz, $^3J_{HH}$ = 8.1 Hz, $J_{HH}$ = 1.1 Hz, 1H, $H^a$-C3), 3.15 (dddd, $^3J_{HF}$ = 27.2 Hz, $^2J_{HH}$ = 15.0 Hz, $^3J_{HH}$ = 5.2 Hz, $J_{HH}$ = 1.0 Hz, 1H, $H^b$-C3).

**$^{19}$F NMR** (376 MHz, $CDCl_3$): $\delta$ [ppm] = -173.42 (ddd, $^2J_{FH}$ = 47.1 Hz, $^3J_{FH}$ = 27.1 Hz, $^3J_{FH}$ = 14.8 Hz, 1F, F-C4).

**$^{19}$F{$^{1}$H} NMR** (376 MHz, $CDCl_3$): $\delta$ [ppm] = -173.41 (s, 3F, F-C4).

**$^{13}$C{$^{1}$H} NMR** (126 MHz, $CDCl_3$): $\delta$ [ppm] = 142.2 (d, $^{4}J_{CF}$ = 4.8 Hz, C5), 139.0 (C2), 138.3 (d, $^{2}J_{CF}$ = 20.3 Hz, C9), 134.4 (d, $^{5}J_{CF}$ = 3.2 Hz, C12), 133.8 (C8), 128.82 (C6, C7 or C11), 128.80 (C6, C7 or C11 ), 127.7 (C6, C7 or C11), 127.1 (d, $^{3}J_{CF}$ = 6.6 Hz, C10), 116.9 (C1), 92.1 (d, $^{1}J_{CF}$ = 174.4 Hz, C4), 92.1 (d, $^{2}J_{CF}$ = 25.4 Hz, C3).

**GC-EI-MS:** Retention 8.93 min, (*m/z*) requires: $[(C_{16}H_{13}Cl_2F)^{+}]$ = 294.0373, (*m/z*) found: $[(C_{16}H_{13}Cl_2F)^{+}]$ = 294.0373.

**FT-IR** ($\tilde{\nu}$ = cm$^{-1}$)**:** 2927.2 (w), 1628.6 (w), 1597.1 (w), 1490.9 (m), 1414.8 (w), 1394.7 (w), 1364.6 (w), 1317.3 (w), 1298.6 (w), 1261.3 (w), 1224.0 (w), 1192.4 (w), 1086.2 (m), 1048.9 (w), 1010.2 (m), 994.4 (m), 962.8 (w), 915.5 (w), 909.7 (m), 863.8 (m), 836.6 (s), 816.5 (s), 766.2 (m), 740.4 (m), 726.1 (w), 707.4 (w).

**4,4'-(1,3,3-Trifluorobutane-1,4-diyl)bis(chlorobenzene) (2c)**

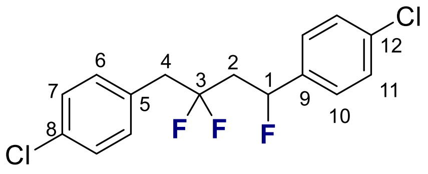


Compound **2c** was prepared according to general procedure **D** (amine:HF = 1:5.5) using 4,4'-(1-fluorobut-3-ene-1,3-diyl)bis-(chlorobenzene) **IIIc** (114.9 mg, 0.389 mmol). The crude product was purified by flash column chromatography (5-10% DCM in cyclohexane) to yield the title compound as a colorless solid (92.0 mg, 0.276 mmol, 71%).

**7-Chloro-1-(4-chlorophenyl)-3,3-difluoro-1,2,3,4-tetrahydronaphthalene (3c)**

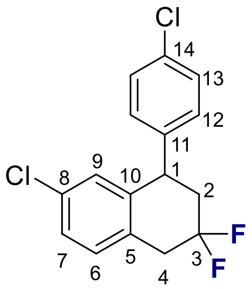


4,4'-(1,3,3-Trifluorobutane-1,4-diyl)bis(chlorobenzene) **2c** (92.0 mg, 0.276 mmol) was dissolved in $CHCl_3$ (0.69 mL). $NEt_3$•3HF (0.34 mL) and Olah's reagent (0.34 mL) were added subsequently, followed by HFIP (2.76 mL). The reaction was stirred at room temperature for 24 h. The work-up was performed according to general procedure **E**. The crude product was purified by flash column chromatography (4-7% DCM in cyclohexane) to yield the title compound as a yellow oil (74.4 mg, 0.238 mmol, 86%).

***Caution:*** *Olah's reagent is highly toxic and corrosive. Direct exposure should be avoided. In the case of skin exposure, immediate treatment of the affected skin area with calcium gluconate gel is necessary to prevent serious chemical burns.*

## Synthesis of 3c via Deoxofluorination of Tetralone S17

DAST (Diethylaminosulfur trifluoride) has been used in the deoxofluorination of tetralone **S15** and this enables the formation of the difluorinated tetralin derivative **S16** in a moderate 38% yield.[13] However, the synthesis of difluorinated aryl tetralin **3a** from aryl tetralin **S17** has not been described yet. Using common deoxofluorinating conditions,[14] we have demonstrated that the desired product **3a** can be obtained in a yield of 18% $^{19}$F NMR (see Supplementary Figure 2). Full conversion, and substantial degradation, of the starting material **S17** was observed by TLC and $^{1}$H NMR. In comparison, this approach allows for the synthesis of this complex motif **3a** in 57% isolated yield starting from the corresponding cyclobutanol ***major*-1a** (*cis*).

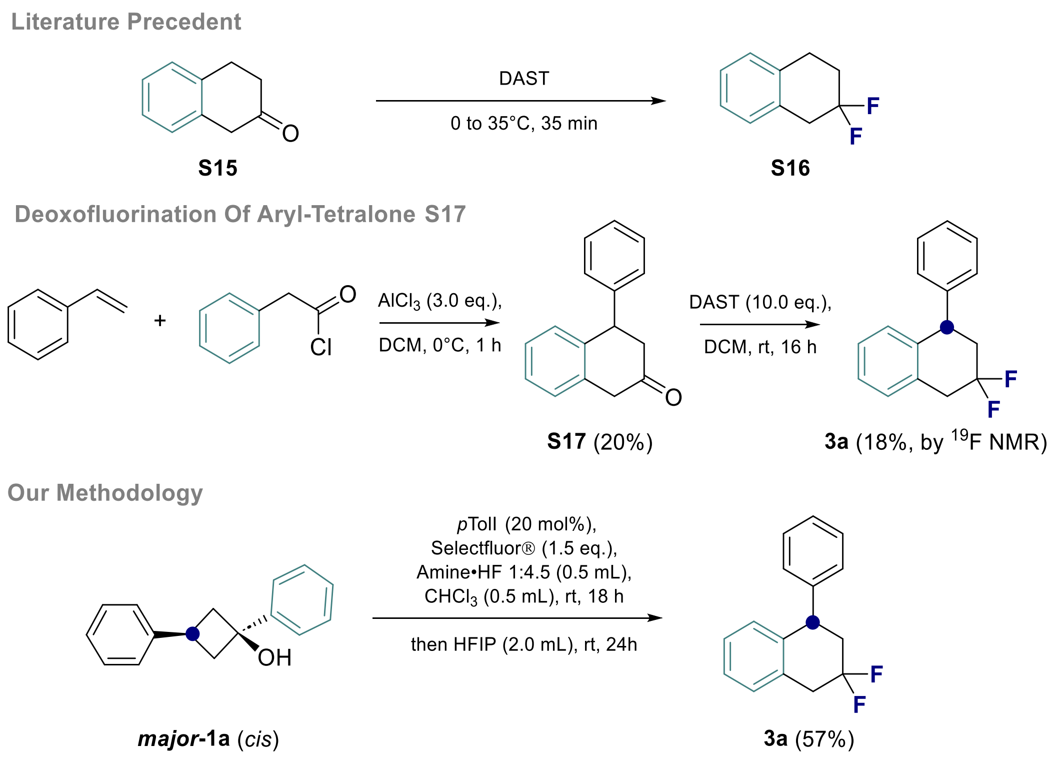


**Supplementary Figure 2.** Comparison of different methodologies for the synthesis of difluorinated tetralin derivatives.

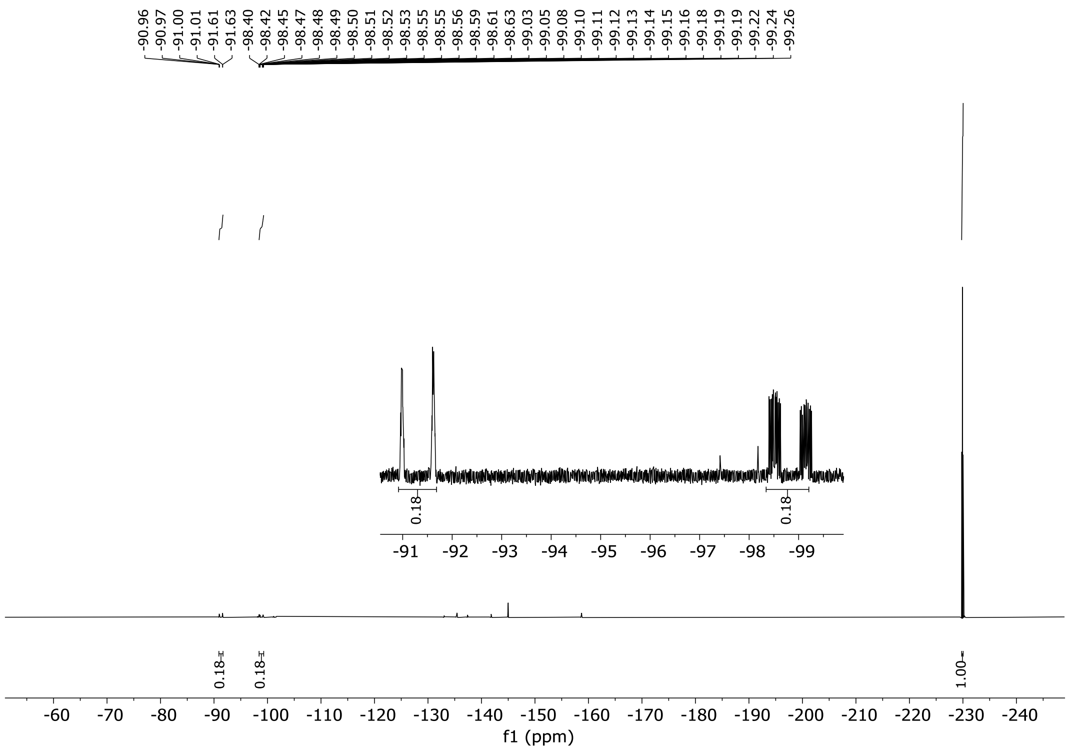


**Supplementary Figure 3.** $^{19}F$ crude NMR of the deoxofluorination of **S17**. Ethyl 2-fluoroacetate was used as the internal standard.

### 4-Phenyl-3,4-dihydronaphthalen-2(1H)-one (S17)

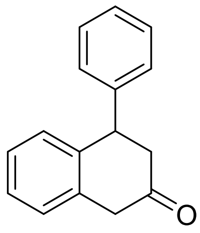


Compound **S17** was prepared following a literature procedure.[15]

Anhydrous $AlCl_3$ (975 mg, 7.2 mmol, 3.0 eq.) and phenylacetyl chloride (320 µL, 2.4 mmol, 1.0 eq.) were dissolved in dry DCM (30 mL). A solution of styrene (276 µL, 2.4 mmol, 1.0 eq.) in dry DCM (30 mL) was added drop wise over 15 min at 0 °C. The reaction mixture was stirred at 0 °C for 1 h. The reaction was quenched by adding a saturated solution of Seignette salt (50 mL). The layers were separated and the organic layer was washed with a saturated aqueous $NaHCO_3$ (3x). The organic layer was dried over $Na_2SO_4$ and the solvent was removed under reduced pressure. The crude product was purified by flash column chromatography (0-5% EtOAc in *n*-pentane) to yield the product as a colorless oil (108 mg, 0.48 mmol, 20%).

$\mathbf{R_f}$ = 0.28 (5% EtOAc in *n*-pentane).

**$^1$H NMR** (599 MHz, $CDCl_3$): $\delta$[ppm] = 7.35 (t, $J$ = 7.0 Hz, 2H), 7.28 (tt, $J$ = 7.1 Hz, 0.8 Hz, 2H), 7.25 – 7.19 (m, 2H), 7.16 (ddd, $J$ = 7.5 Hz, $J$ = 1.4 Hz, $J$ = 0.6 Hz, 2H), 7.03 (d, $J$ = 6.9 Hz, 1H), 4.48 (t, $J$ = 6.6 Hz, 1H), 3.75 – 3.57 (m, 2H), 3.02 – 2.87 (m, 2H).

**GC-EI-MS:** Retention 8.86 min**,** (*m/z*) requires: [$(C_{16}H_{14}O)^+$] = 222.10, (*m/z*) found: [$(C_{16}H_{14}O)^+$] = 222.11.

The analytic data is in good agreement with literature values.[15]

**3,3-Difluoro-1-phenyl-1,2,3,4-tetrahydronaphthalene (3a)**

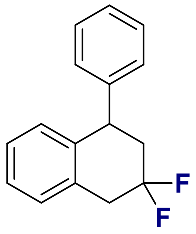


Compound **3a** was prepared following a literature procedure.[14]

In a Schlenk flask, 4-phenyl-3,4-dihydronaphthalen-2(1H)-one **S17** (44.4 mg, 0.2 mmol, 1.0 eq.) was dissolved in dry DCM (2 mL). DAST (0.27 mL, 2.0 mmol, 10.0 eq.) was added drop wise at 0 °C. The reaction mixture was stirred at room temperature for 16 h. The reaction was carefully quenched by adding saturated aqueous $NaHCO_3$. The aqueous layers were extracted with DCM (3x). The combined organic layers were dried over $Na_2SO_4$ and the solvent was removed under reduced pressure. An $^{19}F$ NMR yield of 18% was measured using ethyl fluoroacetate as the internal standard. (See Supplementary Figure 3)

## Additional Modifications of the Products 2c, 3q and 3s

### 1,4-Bis(4-chlorophenyl)-3,3-difluorobutyl acetate (4)

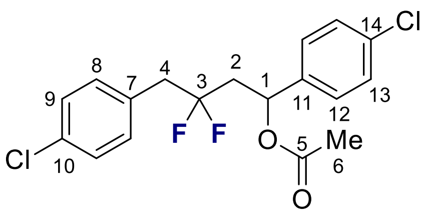


Compound **4** was prepared according to modified general procedure **E** (amine:HF 1:5.5) using 1,3-bis(4-chlorophenyl)cyclobutan-1-ol ***major*-1c** (58.6 mg, 0.200 mmol). Instead of HFIP, AcOH (1.0 mL) followed by Olah's reagent (1.0 mL) were added after 18 h. The crude product was purified by flash column chromatography (5-7.5% EtOAc in cyclohexane) to yield the title compound as a colorless solid (53.2 mg, 0.143 mmol, 72%).

**$R_f$** = 0.27 (10% EtOAc in cyclohexane).

**Melting Point:** 67-69 ˚C.

**$^1$H NMR** (500 MHz, $CDCl_3$): $\delta$ [ppm] = 7.33 – 7.28 (m, 4H, H-C9, H-C13), 7.26 – 7.22 (m, 2H, H-C12), 7.21 – 7.16 (m, 2H, H-C8), 6.10 (dd, $^3J_{HH}$ = 8.7 Hz, $^3J_{HH}$ = 4.1 Hz, 1H, H-C1), 3.31 – 3.06 (m, 2H, H-C4), 2.47 (dddd, $^3J_{HF}$ = 19.3 Hz, $^2J_{HH}$ = 15.3 Hz, $^3J_{HF}$ = 11.7 Hz, $^3J_{HH}$ = 9.0 Hz, 1H, $H^a$-C2), 2.21 (dddd, $^3J_{HF}$ = 32.1 Hz, $^2J_{HH}$ = 15.3 Hz, $^3J_{HF}$ = 12.7 Hz, $^3J_{HH}$ = 3.7 Hz, 1H, $H^b$-C2), 2.07 (s, 3H, H-C6).

**$^{19}$F NMR** (376 MHz, $CDCl_3$): $\delta$ [ppm] = -94.73 (ddtd, $^2J_{FF}$ = 247.5 Hz, $^3J_{FH}$ = 19.1 Hz, $^3J_{FH}$ = 16.2 Hz, $^3J_{FH}$ = 12.9 Hz, 1F, $F^a$-C3), -95.86 (ddtd, $^2J_{FF}$ = 247.5 Hz, $^3J_{FH}$ = 31.4 Hz, $^3J_{FH}$ = 16.5 Hz, $^3J_{FH}$ = 12.0 Hz, 1F, $F^b$-C3).

**$^{19}$F{$^1$H} NMR** (376 MHz, $CDCl_3$): $\delta$ [ppm] = -94.73 (d, $^2J_{FF}$ = 247.5 Hz, 1F, $F^a$-C3), -95.86 (dd, $^2J_{FF}$ = 247.5 Hz, 1F, $F^b$-C3).

**$^{13}$C{$^1$H} NMR** (126 MHz, $CDCl_3$): $\delta$ [ppm] = 169.8 (C5), 138.5 (C11), 134.4 (C14), 133.8 (C10), 131.8 (d, $^4J_{CF}$ = 1.2 Hz, C8), 131.2 (t, $^3J_{CF}$ = 4.5 Hz, C7), 129.0 (C9), 128.9 (C13), 128.0 (C12), 122.2 (t, $^1J_{CF}$ = 244.1 Hz, C3), 69.7 (t, $^3J_{CF}$ = 4.5 Hz, C1), 42.9 (t, $^2J_{CF}$ = 25.7 Hz, C4), 42.2 (t, $^2J_{CF}$ = 24.5 Hz, C2), 21.2 (C6).

**ESI-MS:** (*m/z*) requires: $[(C_{18}H_{16}Cl_2F_2O_2Na)^+]$ = 395.0388, (*m/z*) found: $[(C_{18}H_{16}Cl_2F_2O_2Na)^+]$ = 395.0387.

**FT-IR** ($\tilde{\nu}$ = $cm^{-1}$)**:** 1740.6 (s), 1492.3 (m), 1407.7 (w), 1371.8 (m), 1315.8 (w), 1257.0 (w), 1231.2 (s), 1212.5 (s), 1147.9 (m), 1103.5 (m), 1076.2 (m), 1011.6 (s), 942.7 (m), 888.2 (w), 856.6 (m), 829.4 (m), 780.6 (s), 733.2 (m), 714.6 (w).

**4,4'-(3,3-Difluoro-1-methoxybutane-1,4-diyl)bis(chlorobenzene) (5)**

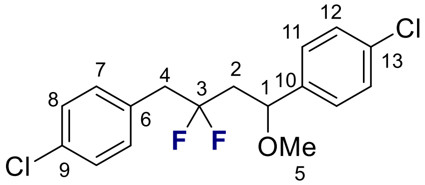


Compound **5** was prepared according to modified general procedure **E** (amine:HF 1:5.5) using 1,3-bis(4-chlorophenyl)cyclobutan-1-ol ***major*-1c** (58.6 mg, 0.200 mmol). Instead of HFIP, MeOH (1.0 mL) followed by Olah's reagent (2.0 mL) were added after 18 h. The reaction mixture was stirred for additional 48 h. The crude product was purified by flash column chromatography (10-20% DCM in cyclohexane) to yield the title compound as a colorless solid (47.7 mg, 0.138 mmol, 69%).

**R*f*** = 0.41 (5% EtOAc in cyclohexane).

**Melting Point:** 87-89 ˚C.

**$^{1}$H NMR** (500 MHz, $CDCl_3$): $\delta$[ppm] = 7.35 – 7.31 (m, 2H, H-C12), 7.31 – 7.28 (m, 2H, H-C8), 7.25 – 7.20 (m, 4H, H-C7, H-C11), 4.45 (dd, $^{3}J_{HH}$ = 9.0 Hz, $^{3}J_{HH}$ = 3.5 Hz, 1H, H-C1), 3.27 – 3.16 (m, 2H, H-C4), 3.22 (s, 3H, H-C5), 2.29 (dddd, $^{3}J_{HF}$ = 21.7 Hz, $^{2}J_{HH}$ = 15.0 Hz, $^{3}J_{HF}$ = 12.8 Hz, $^{3}J_{HH}$ = 9.0 Hz, 1H, $H^{a}$-C2), 2.00 (dddd, $^{2}J_{HH}$ = $^{3}J_{HF}$ = 15.3 Hz, $^{3}J_{HF}$ = 12.5 Hz, $^{3}J_{HH}$ = 3.5 Hz, 1H, $H^{b}$-C2).

**$^{19}$F NMR** (376 MHz, $CDCl_3$): $\delta$ [ppm] = -91.47 (dtt, $^{2}J_{FF}$ = 246.2 Hz, $^{3}J_{FH}$ = 19.4 Hz, $^{3}J_{FH}$ = 12.8 Hz, 1F, $F^{a}$-C3), -96.29 (ddtd, $^{2}J_{FF}$ = 246.2 Hz, $^{3}J_{FH}$ = 20.6 Hz, $^{3}J_{FH}$ = 15.2 Hz, $^{3}J_{FH}$ = 12.8 Hz, 1F, $F^{b}$-C3).

**$^{19}$F{$^{1}$H} NMR** (376 MHz, $CDCl_3$): $\delta$ [ppm] = -91.47 (d, $^{2}J_{FF}$ = 246.2 Hz, 1F, $F^{a}$-C3), -96.29 (d, $^{2}J_{FF}$ = 246.2 Hz, 1F, $F^{b}$-C3).

**$^{13}$C{$^{1}$H} NMR** (126 MHz, $CDCl_3$): $\delta$[ppm] = 139.9 (C10), 133.9 (C13), 133.5 (C9), 132.1 (C7), 131.9 (dd, $^{3}J_{CF}$ = 6.6 Hz, $^{3}J_{CF}$ = 2.7 Hz, C6), 129.0 (C12), 128.7 (C8), 128.0 (C11), 122.8 (dd, $^{1}J_{CF}$ = 244.0 Hz, $^{1}J_{CF}$ = 242.4 Hz, C3), 78.1 (dd, $^{3}J_{CF}$ = 7.9 Hz, $^{3}J_{CF}$ = 3.5 Hz, C1), 59.7 (C5), 44.1 (t, $^{2}J_{CF}$ = 24.6 Hz, C2), 42.9 (dd, $^{2}J_{CF}$ = 26.7 Hz, $^{2}J_{CF}$ = 24.8 Hz, C4).

Comment: HRMS-analysis was inconclusive, therefore [M]-$CH_3OH$ was reported as an indicative fragment.

**GC-EI-MS:** Retention: 9.98 min, (*m/z*) requires: [($C_{17}H_{16}Cl_2F_2O$ – $HOCH_3$)$^{+}$] = 312.0279; (*m/z*) found: [($C_{17}H_{16}Cl_2F_2O$ – $HOCH_3$)$^{+}$] = 312.0277.

**FT-IR** ($\tilde{\nu}$ = $cm^{-1}$)**:** 2988.9 (w), 2822.5 (w), 1594.2 (w), 1492.3 (m), 1430.6 (w), 1407.7 (w), 1373.2 (w), 1347.4 (w), 1318.7 (w), 1255.6 (w), 1215.4 (w), 1178.1 (w), 1153.7 (m), 1133.6 (w), 1099.1 (s), 1054.7 (w), 1011.6 (s), 977.2 (w), 949.9 (w), 891.1 (w), 863.8 (m), 849.5 (w), 838.0 (m), 828.0 (w), 807.9 (m), 782.0 (s), 733.2 (m), 717.5 (w), 700.2 (w).

**4,4'-(1-(2,5-Dimethylphenyl)-3,3-difluorobutane-1,4-diyl)bis(chlorobenzene) (6)**

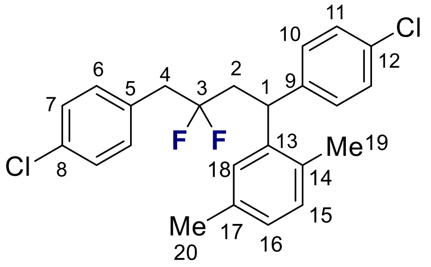


Compound **6** was prepared according to modified general procedure **E** (amine:HF 1:5.5) using 1,3-bis(4-chlorophenyl)cyclobutan-1-ol ***major*-1c** (58.6 mg, 0.200 mmol). Instead of HFIP, 1,4-dimethylbenzene (1.0 mL) followed by Olah's reagent (1.0 mL) were added after 18 h. The reaction mixture was stirred for 48 h after the addition. The crude product was purified by flash column chromatography (4-5% DCM in cyclohexane) to yield the title compound as a colorless oil (41.0 mg, 0.098 mmol, 49%).

**R*f*** = 0.21 (10% DCM in cyclohexane).

**$^1$H NMR** (500 MHz, $CDCl_3$): $\delta$ [ppm] = 7.29 – 7.26 (m, 2H, H-C7), 7.25 – 7.22 (m, 2H, H-C11), 7.16 – 7.12 (m, 2H, H-C10), 7.07 (d, $^3J_{HH}$ = 8.2 Hz, 2H, H-C6), 7.03 (d, $^3J_{HH}$ = 8.2 Hz, 1H, H-C15), 6.96 – 6.92 (m, 2H, H-C16, H-C18), 4.56 (t, $^3J_{HH}$ = 6.8 Hz, 1H, H-C1), 2.98 (t, $^3J_{HF}$ = 16.2 Hz, 2H, H-C4). 2.63 – 2.51 (m, 2H, H-C2), 2.30 (s, 3H, H-C20), 2.28 (s, 3H, H-C19).

**$^{19}$F NMR** (376 MHz, $CDCl_3$): $\delta$ [ppm] = -93.79 (dp, $^2J_{FF}$ = 244.0 Hz, $^3J_{FH}$ = 16.5 Hz, 1F, $F^a$-C3), -95.05 (dp, $^2J_{FF}$ = 243.9 Hz, $^3J_{FH}$ = 16.3 Hz, 1F, $F^b$-C3).

**$^{19}$F{$^1$H} NMR** (376 MHz, $CDCl_3$): $\delta$ [ppm] = -93.79 (d, $^2J_{FF}$ = 244.0 Hz, 1F, $F^a$-C3), -95.05 (d, $^2J_{FF}$ = 243.9 Hz, 1F, $F^b$-C3).

**$^{13}$C{$^1$H} NMR** (126 MHz, $CDCl_3$): $\delta$ [ppm] = 142.4 (C9), 141.6 (C13), 135.8 (C17), 133.5 (C8), 132.6 (C14), 132.2 (C12), 131.8 (C6), 131.6 (t, $^3J_{CF}$ = 4.2 Hz, C5), 130.9 (C15), 129.5 (C10), 128.7 (C7 and C11), 127.49 (C16 or C18), 127.46 (C16 or C18), 123.3 (t, $^1J_{CF}$ = 243.9 Hz, C3), 43.1 (t, $^2J_{CF}$ = 25.9 Hz, C4), 42.0 (t, $^2J_{CF}$ = 24.2 Hz, C2), 39.8 (t, $^3J_{CF}$ = 3.9 Hz, C1), 21.4 (C20), 19.5 (C19).

**GC-EI-MS:** Retention: 11.79 min, (*m/z*) requires: [$(C_{24}H_{22}Cl_2F_2)^+$] = 418.1061, (*m/z*) found: [$(C_{24}H_{22}Cl_2F_2)^+$] = 418.1061.

**FT-IR** ($\tilde{\nu}$ = $cm^{-1}$)**:** 3043.5 (w), 2924.4 (w), 1489.4 (s), 1437.8 (w), 1409.1 (w), 1370.3 (w), 1346.0 (w), 1288.6 (w), 1258.4 (w), 1122.1 (w), 1089.1 (s), 1047.5 (m), 1013.1 (s), 991.5 (w), 942.7 (w), 885.3 (w), 840.9 (m), 807.9 (s), 783.5 (m).

**2-((1,4-Bis(4-chlorophenyl)-3,3-difluorobutyl)thio)benzo[d]thiazole (7)**

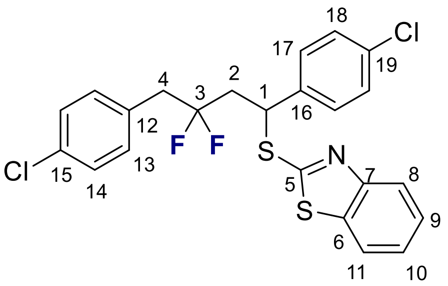


4,4'-(1,3,3-Trifluorobutane-1,4-diyl)bis(chloro-benzene) **2c** (66.6 mg, 0.200 mmol, 1.0 eq.) was dissolved in a mixture of $CHCl_3$ (0.4 mL) and HFIP (1.2 mL). 2-Mercaptobenzothiazole (66.9 mg, 0.400 mmol, 2.0 eq.) was added in one portion and the reaction mixture was stirred at 40 ˚C for 66 h. The reaction mixture was poured into saturated aqueous $NaHCO_3$ (25 mL). The aqueous layer was extracted with DCM (3 x 25 mL). The combined organic layers were dried over $Na_2SO_4$ and the solvent was removed under reduced pressure. The crude product was purified by flash column chromatography (4% EtOAc in cyclohexane) to yield the title compound as a pale-yellow solid (82.2 mg, 0.171 mmol, 86%).

$\mathbf{R}_f$ = 0.12 (4% EtOAc in cyclohexane).

**Melting Point:** 114-116 ˚C.

**$^1$H NMR** (500 MHz, $CDCl_3$): $\delta$ [ppm] = 7.88 (dd, $^3J_{HH}$ = 8.2 Hz, $^4J_{HH}$ = 1.2 Hz, 1H, H-C8), 7.75 (dd, $^3J_{HH}$ = 8.0 Hz, $^4J_{HH}$ = 1.3 Hz, 1H, H-C11), 7.45 (ddd, $^3J_{HH}$ = 8.3 Hz, $^3J_{HH}$ = 7.3 Hz, $^4J_{HH}$ = 1.2 Hz, 1H, H-C9), 7.37 – 7.34 (m, 2H, H-C17), 7.33 (ddd, $^3J_{HH}$ = 8.0 Hz, $^3J_{HH}$ = 7.3 Hz, $^4J_{HH}$ = 1.1 Hz, 1H, H-C10), 7.31 – 7.27 (m, 2H, H-C18), 7.27 – 7.24 (m, 2H, H-C14), 7.15 – 7.10 (m, 2H, H-C13), 5.32 (dd, $^3J_{HH}$ = 9.8 Hz, $^3J_{HH}$ = 4.5 Hz, 1H, H-C1), 3.10 (t, $^3J_{HF}$ = 16.2 Hz, 2H, C4), 2.84 (dddd, $^3J_{HF}$ = 21.7 Hz, $^2J_{HH}$ = 15.3 Hz, $^3J_{HF}$ = 11.0 Hz, $^3J_{HH}$ = 4.5 Hz, 1H, H$^a$-C2), 2.63 (dddd, $^3J_{HF}$ = 20.1 Hz, $^2J_{HH}$ = 14.9 Hz, $^3J_{HF}$ = $^3J_{HH}$ = 10.1 Hz, 1H, H$^b$-C2).

**$^{19}$F NMR** (376 MHz, $CDCl_3$): $\delta$ [ppm] = -93.16 (ddtd, $^2J_{FF}$ = 245.4 Hz, $^3J_{FH}$ = 21.6 Hz, $^3J_{FH}$ = 16.0 Hz, $^3J_{FH}$ = 10.4 Hz, 1F, F$^a$-C3), -95.44 (ddtd, $^2J_{FF}$ = 243.5 Hz, $^3J_{FH}$ = 20.0 Hz, $^3J_{FH}$ = 16.6 Hz, $^3J_{FH}$ = 11.1 Hz, 1F, F$^b$-C3).

**$^{19}$F{$^1$H} NMR** (376 MHz, $CDCl_3$): $\delta$ [ppm] = -93.16 (d, $^2J_{FF}$ = 245.4 Hz, 1F, F$^a$-C3), -95.44 (d, $^2J_{FF}$ = 245.4 Hz, 1F, F$^b$-C3).

**$^{13}$C{$^1$H} NMR** (126 MHz, $CDCl_3$): $\delta$ [ppm] = 164.0 (C5), 153.2 (C7), 138.4 (C16), 135.6 (C6), 134.0 (C19), 133.7 (C15), 131.8 (C13), 131.1 (t, $^3J_{CF}$ = 4.2 Hz, C12), 129.4 (C17), 129.0 (C18), 128.8 (C14), 126.3 (C9), 124.9 (C10), 122.4 (t, $^1J_{CF}$ = 245.0 Hz, C3), 122.1 (C8), 121.2 (C11), 46.0 (t, $^3J_{CF}$ = 3.5 Hz, C1), 43.0 (t, $^2J_{CF}$ = 25.6 Hz, C4), 42.2 (t, $^2J_{CF}$ = 24.3 Hz, C2).

**ESI-MS:** (*m/z*) requires: $[(C_{23}H_{17}Cl_2F_2NS_2Na)^+]$ = 502.0040, (*m/z*) found: $[(C_{23}H_{17}Cl_2F_2NS_2Na)^+]$ = 502.0038.

**FT-IR** ($\tilde{\nu}$ = cm$^{-1}$)**:** 1490.9 (w),1456.4 (w), 1427.7 (m), 1409.1 (w), 1357.4 (w), 1341.6 (w), 1311.5 (w), 1255.6 (w), 1142.2 (w), 1116.4 (m), 1103.5 (w), 1086.2 (m), 1050.4 (m), 1014.5 (m), 991.5 (m), 919.8 (w), 883.9 (w), 863.8 (w), 840.9 (m), 803.6 (s), 757.6 (s), 731.8 (m), 720.3 (m), 706.0 (w).

**2-((1,4-Bis(4-chlorophenyl)-3,3-difluorobutyl)sulfonyl)benzo[d]thiazole (8)**

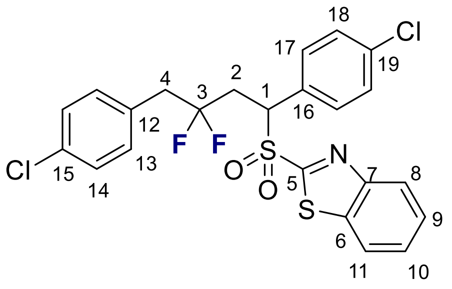


The reaction was performed following a literature procedure.[16]

2-((1,4-Bis(4-chlorophenyl)-3,3-difluorobutyl)thio)-benzo[d]thiazole **7** (96.1 mg, 0.200 mmol, 1.0 eq.) was dissolved in $CHCl_3$ (1.0 mL) and a solution of *m*-CPBA (108.4 mg, 70 wt%, 0.440 mmol, 2.2 eq.) in $CHCl_3$ (2.0 mL) was added at -10 ˚C. The reaction was warmed to room temperature after 15 min and was stirred for additional 15 h. The reaction mixture was poured into saturated aqueous $NaHCO_3$ (25 mL). The aqueous layer was extracted with DCM (3 x 25 mL). The combined organic layers were dried over $Na_2SO_4$ and the solvent was removed under reduced pressure. The crude product was purified by flash column chromatography (5-10% EtOAc in cyclohexane) to yield the title compound as a colorless solid (87.4 mg, 0.171 mmol, 86%).

**R*f*** = 0.14 (10% EtOAc in cyclohexane).

**Melting Point:** 136-138 ˚C.

**$^1$H NMR** (500 MHz, $CDCl_3$): $\delta$ [ppm] = 8.21 (dt, $^3J_{HH}$ = 8.3 Hz, $^4J_{HH}$ = 1.0 Hz, 1H, H-C8), 7.92 (dt, $^3J_{HH}$ = 8.0 Hz, $^4J_{HH}$ = 1.0 Hz, 1H, H-C11), 7.65 (ddd, $^3J_{HH}$ = 8.4 Hz, $^3J_{HH}$ = 7.1 Hz, $^4J_{HH}$ = 1.0 Hz, 1H, H-C9), 7.59 (ddd, $^3J_{HH}$ = 8.3 Hz, $^3J_{HH}$ = 7.2 Hz, $^4J_{HH}$ = 1.2 Hz, 1H, H-C10), 7.24 – 7.18 (m, 6H, H-C14, H-C17, H-C18), 7.10 – 7.03 (m, 2H, H-C13), 5.00 (dd, $^3J_{HH}$ = 11.1 Hz, $^3J_{HH}$ = 2.2 Hz, 1H, H-C1), 3.12 – 2.97 (m, 3H, H$^a$-C2, H-C4), 2.21 (dddd, $^3J_{HF}$ = 22.8 Hz, $^2J_{HH}$ = 15.1 Hz, $^3J_{HH}$ = 11.1 Hz, $^3J_{HF}$ = 7.4 Hz, 1H, H$^b$-C2).

**$^{19}$F NMR** (376 MHz, $CDCl_3$): $\delta$ [ppm] = -93.99 (ddtd, $^2J_{FF}$ = 243.6 Hz, $^3J_{FH}$ = 23.2 Hz, $^3J_{FH}$ = 15.8 Hz, $^3J_{FH}$ = 7.4 Hz, 1F, F$^a$-C3), -96.44 (ddtd, $^2J_{FF}$ = 243.5 Hz, $^3J_{FH}$ = 23.9 Hz, $^3J_{FH}$ = 16.4 Hz, $^3J_{FH}$ = 7.7 Hz, 1F, F$^b$-C3).

**$^{19}$F{$^1$H} NMR** (376 MHz, $CDCl_3$): $\delta$ [ppm] = -93.99 (d, $^2J_{FF}$ = 243.6 Hz, 1F, F$^a$-C3), -96.82 (d, $^2J_{FF}$ = 243.5 Hz, 1F, F$^b$-C3).

**$^{13}$C{$^1$H} NMR** (126 MHz, $CDCl_3$): $\delta$ [ppm] = 163.8 (C5), 152.6 (C7), 137.3 (C6), 135.8 (C19), 133.9 (C15), 131.6 (C13), 131.3 (C17), 130.5 (t, $^3J_{CF}$ = 4.4 Hz, C12), 129.3 (C16), 129.2 (C18), 128.9 (C14), 128.4 (C10), 127.9 (C9), 125.7 (C8), 122.4 (C11), 122.1 (t, $^1J_{CF}$ = 245.7 Hz, C3), 64.6 (dd, $^3J_{CF}$ = 3.8 Hz, $^3J_{CF}$ = 1.5 Hz, C1), 43.2 (t, $^2J_{CF}$ = 25.4 Hz, C4), 34.6 (t, $^2J_{CF}$ = 24.7 Hz, C2).

**ESI-MS:** (*m/z*) requires: $[(C_{23}H_{17}Cl_2F_2NO_2S_2Na)^+]$ = 533.9938, (*m/z*) found: $[(C_{23}H_{17}Cl_2F_2NO_2S_2Na)^+]$ = 533.9939.

**FT-IR** ($\tilde{\nu}$ = cm$^{-1}$)**:** 1492.3 (w), 1463.6 (w), 1412.0 (w), 1383.3 (w), 1331.6 (m), 1257.0 (w), 1150.8 (s), 1120.7 (m), 1089.1 (m), 1057.5 (m), 1031.7 (w), 919.8 (w), 883.9 (m), 842.3 (m), 803.6 (m), 760.5 (s), 728.9 (m), 711.7 (m).

**(1,4-Bis(4-chlorophenyl)-3,3-difluorobutyl)(4-nitrophenyl)sulfane (9)**

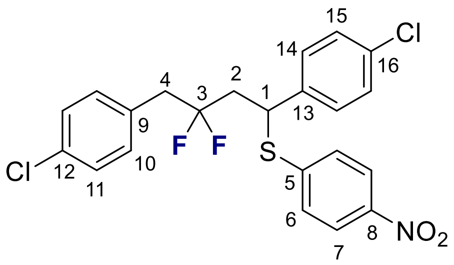


4,4'-(1,3,3-Trifluorobutane-1,4-diyl)bis(chloro-benzene) **2c** (66.6 mg, 0.200 mmol, 1.0 eq.) was dissolved in a mixture of $CHCl_3$ (0.4 mL) and HFIP (1.2 mL). 4-Nitrobenzenethiol (195 mg, 80 wt%, 1.00 mmol, 5.0 eq.) was added in one portion and the reaction mixture was stirred at 40 ˚C for 24 h. The reaction mixture was poured into saturated aqueous $NaHCO_3$ (25 mL). The aqueous layer was extracted with DCM (3 x 25 mL) and the combined organic layers were dried over $Na_2SO_4$, before the solvent was removed under reduced pressure. The crude product was purified by flash column chromatography (20-30% DCM in *n*-pentane) to yield the title compound as a pale-yellow solid (86.6 mg, 0.185 mmol, 93%).

**R*f*** = 0.25 (10% EtOAc in cyclohexane).

**Melting Point:** 96-98 ˚C.

**$^1$H NMR** (500 MHz, $CDCl_3$): $\delta$ [ppm] = 8.07 – 8.03 (m, 2H, H-C7), 7.30 – 7.23 (m, 8H, H-C6, H-C11, H-C14, H-C15), 7.12 – 7.06 (m, 2H, H-C10), 4.69 (dd, $^3J_{HH}$ = 8.6 Hz, $^3J_{HH}$ = 4.9 Hz, 1H, H-C1), 3.14 – 2.92 (m, 2H, H-C4), 2.57 – 2.37 (m, 2H, H-C2).

**$^{19}$F NMR** (376 MHz, $CDCl_3$): $\delta$ [ppm] = -94.81 (dp, $^2J_{FF}$ = 244.8 Hz, $^3J_{FH}$ = 16.8 Hz, 1F, F$^a$-C3), -95.48 (dp, $^2J_{FF}$ = 244.8 Hz, $^3J_{FH}$ = 16.5 Hz, 1F, F$^b$-C3).

**$^{19}$F{$^1$H} NMR** (376 MHz, $CDCl_3$): $\delta$ [ppm] = -94.81 (d, $^2J_{FF}$ = 244.8 Hz, 1F, F$^a$-C3), -95.48 (d, $^2J_{FF}$ = 244.8 Hz, 1F, F$^b$-C3).

**$^{13}$C{$^1$H} NMR** (126 MHz, $CDCl_3$): $\delta$ [ppm] = 146.3 (C5), 144.2 (C8), 138.6 (C13), 134.0 (C16), 133.9 (C12), 131.7 (C10), 130.9 (t, $^3J_{CF}$ = 4.4 Hz, C9), 129.5 (C6), 129.2 (C14 and C15), 128.9 (C11), 124.1 (C7), 122.4 (t, $^1J_{CF}$ = 244.7 Hz, C3), 45.0 (t, $^3J_{CF}$ = 3.2 Hz, C1), 43.1 (t, $^2J_{CF}$ = 25.6 Hz, C4), 42.4 (t, $^2J_{CF}$ = 24.4 Hz, C2).

**ESI-MS:** (*m/z*) requires: $[(C_{22}H_{17}Cl_2F_2NO_2SNa)^+]$ = 490.0217, (*m/z*) found: $[(C_{22}H_{17}Cl_2F_2NO_2SNa)^+]$ = 490.0217.

**FT-IR** ($\tilde{\nu}$ = cm$^{-1}$)**:** 1592.8 (w), 1577.0 (m), 1508.1 (m), 1492.3 (m), 1479.4 (m), 1440.7 (w), 1413.4 (w), 1338.8 (s), 1249.8 (w), 1211.1 (m), 1189.5 (m), 1090.5 (s), 1047.5 (m), 1020.2 (m), 1013.1 (m), 876.7 (m), 839.4 (s), 800.7 (m), 784.9 (m), 769.1 (m), 739.0 (s), 726.1 (s).

***N*-(1,4-Bis(4-chlorophenyl)-3,3-difluorobutyl)acetamide (10)**

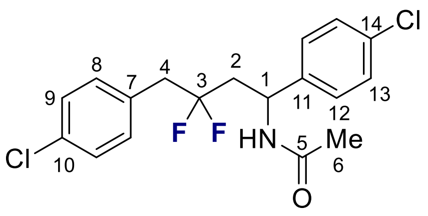


4,4'-(1,3,3-Trifluorobutane-1,4-diyl)bis(chloro-benzene) **2c** (66.6 mg, 0.200 mmol, 1.0 eq.) was dissolved in MeCN (1.0 mL) in a Teflon vial. Olah's reagent was added carefully, the vial was sealed and stirred at 40 ˚C for 24 h. The reaction mixture was poured into saturated aqueous $NaHCO_3$ (150 mL). The aqueous layer was extracted with DCM (3 x 30 mL). The combined organic layers were dried over $Na_2SO_4$ and the solvent was removed under reduced pressure. The crude product was purified by flash column chromatography (10-50% EtOAc in cyclohexane) to yield the title compound as a colorless solid (58.1 mg, 0.156 mmol, 78%).

***Caution:*** *Olah's reagent is highly toxic and corrosive. Direct exposure should be avoided. In the case of skin exposure, immediate treatment of the affected skin area with calcium gluconate gel is necessary to prevent serious chemical burns.*

**R*f*** = 0.33 (50% EtOAc in cyclohexane).

**Melting Point:** 184-186 ˚C.

**$^{1}$H NMR** (500 MHz, DMSO-*d*$_6$): $\delta$ [ppm] = 8.43 (d, $^3J_{HH}$ = 8.6 Hz, 1H, N-H), 7.41 – 7.36 (m, 4H, H-C9, H-C13), 7.34 – 7.31 (m, 2H, H-C12), 7.30 – 7.25 (m, 2H, H-C8), 5.20 (td, $^3J_{HH}$ = 8.6 Hz, $^3J_{HH}$ = 5.3 Hz, 1H, H-C1), 3.34 (t, $^3J_{HF}$ = 17.3 Hz, 2H, H-C4), 2.40 – 2.19 (m, 2H, H-C2), 1.82 (s, 3H, H-C6).

**$^{19}$F NMR** (376 MHz, DMSO-*d*$_6$): $\delta$ [ppm] = -94.15 (dp, $^2J_{FF}$ = 240.9 Hz, $^3J_{FH}$ = 16.9 Hz, 1F, F$^a$-C3), -94.93 (dqd, $^2J_{FF}$ = 240.8 Hz, $^3J_{FH}$ = 18.4 Hz, $^3J_{FH}$ = 12.9 Hz, 1F, F$^b$-C3).

**$^{19}$F{$^1$H} NMR** (376 MHz, DMSO-*d*$_6$): $\delta$ [ppm] = -94.15 (dd, $^2J_{FF}$ = 240.9 Hz, 1F, F$^a$-C3), -94.93 (d, $^2J_{FF}$ = 240.8 Hz, 1F, F$^b$-C3).

**$^{13}$C{$^1$H} NMR** (126 MHz, DMSO-*d*$_6$): $\delta$ [ppm] = 168.2 (C5), 142.0 (C11), 132.2 (C8), 132.1 (t, $^3J_{CF}$ = 3.3 Hz, C7), 132.0 (C10 or C14), 131.5 (C10 or C14), 128.4 (C12), 128.3 (C9 or C13), 128.2 (C9 or C13), 123.1 (t, $^1J_{CF}$ = 243.1 Hz, C3), 46.6 (t, $^3J_{CF}$ = 4.2 Hz, C1), 41.6 (t, $^2J_{CF}$ = 23.3 Hz, C2), 41.3 (t, $^2J_{CF}$ = 25.0 Hz, C4), 22.6 (C6).

**ESI-MS:** (*m/z*) requires: [($C_{18}H_{17}Cl_2F_2NONa$)$^+$] = 394.0547, (*m/z*) found: [($C_{18}H_{17}Cl_2F_2NONa$)$^+$] = 394.0546.

**FT-IR** ($\tilde{\nu}$ = cm$^{-1}$)**:** 3314.7 (w), 1653.0 (s), 1541.1 (m), 1493.8 (m), 1432.0 (w), 1407.7 (w), 1368.9 (m), 1290.0 (w), 1268.5 (w), 1248.4 (w), 1216.8 (m), 1150.8 (m), 1106.3 (w), 1089.1 (m), 1048.9 (w), 1011.6 (s), 964.3 (w), 889.7 (w), 866.7 (w), 855.2 (s), 845.2 (w), 829.4 (w), 782.0 (s), 734.7 (s), 710.3 (w).

**4-(3,3-Difluoro-1,2,3,4-tetrahydronaphthalen-1-yl)phenol (11)**

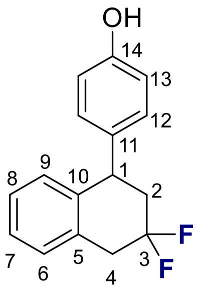


Compound **11** was prepared according to a literature procedure.[17]

4-(3,3-Difluoro-1,2,3,4-tetrahydronaphthalen-1-yl)phenyl trifluoromethanesulfonate **3s** (228.7 mg, 0.583 mmol, 1.0 eq.) was dissolved in 1,4-dioxane (1.75 mL) and $NEt_4OH$ (172.3 mg, 1.17 mmol, 2.0 eq. as a solution in water, 10 wt%) was added drop wise. The reaction was stirred for 90 min at room temperature. The reaction mixture was diluted with DCM (20 mL) and 1 M aqueous HCl (10 mL). The layers were separated and the organic layer was washed with brine and dried over $Na_2SO_4$. The solvent was removed under reduced pressure and the crude product was purified by flash column chromatography (10% EtOAc in cyclohexane) to yield the title compound as a colorless solid (104.2 mg, 0.400 mmol, 69%).

**R*f*** = 0.26 (15% EtOAc in cyclohexane).

**Melting Point:** 133-135 ˚C.

**$^{1}$H NMR** (599 MHz, $CDCl_3$): $\delta$ [ppm] = 7.19 (t, $^{3}J_{HH}$ = 7.3 Hz, 1H, H-C7), 7.14 (dd, $^{3}J_{HH}$ = 7.7 Hz, $J_{HH}$ = 1.4 Hz, 1H, H-C6), 7.10 (td, $^{3}J_{HH}$ = 7.6 Hz, $J_{HH}$ = 1.5 Hz, 1H, H-C8), 7.08 – 7.06 (m, 2H, H-C12), 6.84 – 6.79 (m, 3H, H-C9, H-C13), 4.78 (s, 1H, H-O), 4.28 (dd, $^{3}J_{HH}$ = 12.0 Hz, $^{3}J_{HH}$ = 5.9 Hz, 1H, H-C1), 3.46 – 3.30 (m, 2H, H-C4), 2.55 (tddd, $^{2}J_{HH}$ = $^{3}J_{HF}$ = 13.7 Hz, $^{3}J_{HF}$ = 8.0 Hz, $^{3}J_{HH}$ = 5.8 Hz, $J_{HH}$ = 2.7 Hz, 1H, H$^{a}$-C2), 2.25 (dddd, $^{3}J_{HF}$ = 32.3 Hz, $^{2}J_{HH}$ = 14.1 Hz, $^{3}J_{HH}$ = 12.0 Hz, $^{3}J_{HF}$ = 2.9 Hz, 1H, H$^{b}$-C2).

**$^{19}$F NMR** (376 MHz, $CDCl_3$): $\delta$ [ppm] = -91.04 (ddddd, $^{2}J_{FF}$ = 237.1 Hz, $^{3}J_{FH}$ = 10.5 Hz, $^{3}J_{FH}$ = 8.7 Hz, $^{3}J_{FH}$ = 5.2 Hz, $^{3}J_{FH}$ = 3.1 Hz, 1F, F$^{a}$-C3), -98.58 (ddddd, $^{2}J_{FF}$ = 237.0 Hz, $^{3}J_{FH}$ = 32.2 Hz, $^{3}J_{FH}$ = 28.3 Hz, $^{3}J_{FH}$ = 17.2 Hz, $^{3}J_{FH}$ = 8.2 Hz, 1F, F$^{b}$-C3).

**$^{19}$F{$^{1}$H} NMR** (376 MHz, $CDCl_3$): $\delta$ [ppm] = -91.04 (d, $^{2}J_{FF}$ = 237.1 Hz, 1F, F$^{a}$-C3), -98.58 (d, $^{2}J_{FF}$ = 237.0 Hz, F$^{b}$-C3).

**$^{13}$C{$^{1}$H} NMR** (151 MHz, $CDCl_3$): $\delta$ [ppm] = 154.5 (C14), 138.1 (d, $^{4}J_{CF}$ = 1.6 Hz, C10), 136.5 (C11), 132.0 (d, $^{3}J_{CF}$ = 10.1 Hz, C5), 130.1 (C12), 129.3 (d, $^{5}J_{CF}$ = 1.0 Hz, C9), 129.1 (d, $^{4}J_{CF}$ = 1.7 Hz, C6), 126.93 (C8), 126.89 (C7), 122.7 (dd, $^{1}J_{CF}$ = 241.9 Hz, $^{1}J_{CF}$ = 239.0 Hz, C3), 115.8 (C13), 43.7 (dd, $^{3}J_{CF}$ = 8.2 Hz, $^{3}J_{CF}$ = 2.3 Hz, C1), 40.7 (dd, $^{2}J_{CF}$ = 24.6 Hz, $^{2}J_{CF}$ = 21.7 Hz, C2), 38.7 (dd, $^{2}J_{CF}$ = 27.4 Hz, $^{2}J_{CF}$ = 25.9 Hz, C4).

**ESI-MS:** (*m/z*) requires: [$(C_{16}H_{13}F_2O)^-$] = 259.0940, (*m/z*) found: [$(C_{16}H_{13}F_2O)^-$] = 259.0941.

**FT-IR** ($\tilde{\nu}$ = cm$^{-1}$)**:** 3224.3 (broad), 2958.8 (w), 1615.7 (w), 1601.4 (w), 1513.8 (m), 1490.9 (w), 1450.7 (m), 1423.4 (w), 1367.5 (m), 1318.7 (m), 1278.5 (m), 1265.6 (m), 1244.1 (s), 1178.1 (w), 1156.5 (w), 1123.5 (m), 1084.8 (m), 1069.0 (s), 1041.8 (m), 1026.0 (m), 954.2 (m), 937.0 (w), 906.9 (w), 894.0 (w), 878.2 (w), 865.3 (w), 838.0 (m), 820.8 (s), 784.9 (m), 774.9 (m), 737.6 (m).

**2-(4-(3,3-Difluoro-1,2,3,4-tetrahydronaphthalen-1-yl)phenoxy)-2-methyl-propanoic acid (12)**

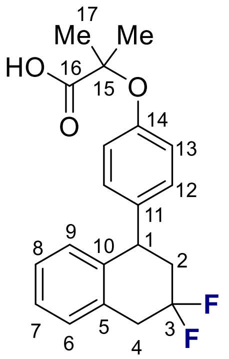


Compound **12** was prepared according to a literature procedure.[18]

4-(3,3-Difluoro-1,2,3,4-tetrahydronaphthalen-1-yl)phenol **14** (78.1 mg, 0.300 mmol, 1.0 eq.), $MgSO_4$ (36.1 mg, 0.300 mmol, 1.0 eq.) and $K_2CO_3$ (165.8 mg, 1.20 mmol, 4.0 eq.) were suspended in DMF (2.0 mL). Subsequently, *tert*-butyl 2-bromo-2-methylpropanoate (335 mg, 1.50 mmol, 5.0 eq.) was added in one portion. The mixture was stirred at 100 ˚C for 24 h. The reaction mixture was cooled to room temperature and diluted with water (20 mL) and EtOAc (20 mL). The layers were separated and the aqueous layer was extracted with additional EtOAc (2x). The combined organic layers were dried over $MgSO_4$. The solvent was removed under reduced pressure and the crude was purified by flash column chromatography (2% EtOAc in cyclohexane) to yield the intermediate *tert*-butyl ester (**R*f*** = 0.28 in 5% EtOAc in cyclohexane). The obtained intermediate was dissolved in DCM (3 mL) and the mixture was cooled to 0 ˚C. Trifluoroacetic acid (684 mg, 6.00 mmol, 20 eq.) was added drop wise and the reaction was stirred at room temperature for 1 h. The volatiles were removed under reduced pressure and the residual oil was coevaporated with toluene (2x 1 mL). The title compound was obtained as a pale-yellow solid (60.1 mg, 0.173 mmol, 58%).

**R*f*** = 0.32 (75% EtOAc in cyclohexane).

**Melting Point:** 138-140 ˚C.

**$^1$H NMR** (599 MHz, $CDCl_3$): $\delta$[ppm] = 7.18 (t, $^3J_{HH}$ = 7.4 Hz, 1H, H-C7), 7.13 (d, $^3J_{HH}$ = 7.5 Hz, 1H, H-C6), 7.11 – 7.07 (m, 3H, H-C8, H-C12), 6.94 – 6.89 (m, 2H, H-C13), 6.80 (d, $^3J_{HH}$ = 7.9 Hz, 1H, H-C9), 4.29 (dd, $^3J_{HH}$ = 12.0 Hz, $^3J_{HH}$ = 5.9 Hz, 1H, H-C1), 3.46 – 3.30 (m, 2H, H-C4), 2.55 (dtdd, $^2J_{HH}$ = 13.9 Hz, $^3J_{HF}$ = 8.3 Hz, $^3J_{HH}$ = 5.9 Hz, $J_{HH}$ = 2.2 Hz, 1H, $H^a$-C2), 2.25 (dddd, $^3J_{HF}$ = 32.1 Hz, $^2J_{HH}$ = 14.0 Hz, $^3J_{HH}$ = 12.1 Hz, $^3J_{HF}$ = 2.9 Hz, 1H, $H^b$-C2), 1.63 (s, 6H, H-C17).

*Comment: Signal for O-H was not observed.*

**$^{19}$F NMR** (564 MHz, $CDCl_3$): $\delta$ [ppm] = -91.18 (ddddd, $^2J_{FF}$ = 237.3 Hz, $^3J_{FH}$ = 10.4 Hz, $^3J_{FH}$ = 8.6 Hz, $^3J_{FH}$ = 5.0 Hz, $^3J_{FH}$ = 2.1 Hz, 1F, $F^a$-C3), -98.59 (ddddd, $^2J_{FF}$ = 237.5 Hz, $^3J_{FH}$ = 32.1 Hz, $^3J_{FH}$ = 28.3 Hz, $^3J_{FH}$ = 17.3 Hz, $^3J_{FH}$ = 8.0 Hz, 1F, $F^b$-C3).

**$^{19}$F{$^1$H} NMR** (564 MHz, $CDCl_3$): $\delta$ [ppm] = -91.18 (d, $^2J_{FF}$ = 237.3 Hz, 1F, F$^a$-C3), -98.59 (d, $^2J_{FF}$ = 237.5 Hz, F$^b$-C3).

**$^{13}$C{$^1$H} NMR** (151 MHz, $CDCl_3$): $\delta$ [ppm] = 178.9 (C16), 153.5 (C14), 138.9 (C11), 137.8 (d, $^4J_{CF}$ = 1.5 Hz, C10), 132.0 (d, $^3J_{CF}$ = 9.9 Hz, C5), 129.7 (C12), 129.3 (C9), 129.2 (d, $^4J_{CF}$ = 1.6 Hz, C6), 126.9 (C7 and C8), 122.6 (dd, $^1J_{CF}$ = 241.9 Hz, $^1J_{CF}$ = 239.0 Hz, C3), 120.9 (C13), 79.7 (C15), 43.8 (dd, $^3J_{CF}$ = 8.1 Hz, $^3J_{CF}$ = 2.2 Hz, C1), 40.6 (dd, $^2J_{CF}$ = 24.8 Hz, $^2J_{CF}$ = 21.9 Hz, C2), 38.6 (dd, $^2J_{CF}$ = 27.4 Hz, $^2J_{CF}$ = 26.0 Hz, C4), 25.29 (C$^a$17), 25.27 (C$^b$17).

**ESI-MS:** (*m/z*) requires: $[(C_{20}H_{19}F_2O_3)^-]$ = 345.1308, (*m/z*) found: $[(C_{20}H_{19}F_2O_3)^-]$ = 345.1298.

**FT-IR** ($\tilde{\nu}$ = cm$^{-1}$)**:** 3003.3 (w), 2914.3 (w), 1698.9 (m), 1607.1 (w), 1509.5 (m), 1469.4 (w), 1361.7 (w), 1320.1 (w), 1301.5 (w), 1264.2 (m), 1244.1 (s), 1201.0 (w), 1159.4 (s), 1125.0 (m), 1112.1 (w), 1086.2 (m), 1067.6 (s), 1044.6 (m), 1024.5 (w), 975.7 (w), 957.1 (m), 911.2 (m), 881.0 (m), 840.9 (m), 828.0 (m), 793.5 (w), 779.2 (w), 746.2 (s), 723.2 (m).

**((4-(3,3-Difluoro-1,2,3,4-tetrahydronaphthalen-1-yl)phenyl)ethynyl)triisopropylsilane (13)**

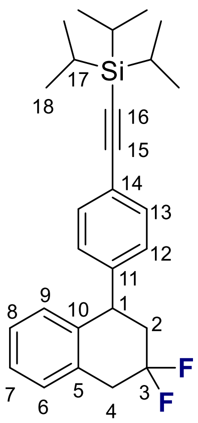


Compound **13** was prepared according to a modified literature procedure.[7]

1-(4-Bromophenyl)-3,3-difluoro-1,2,3,4-tetrahydronaphthalene **3q** (64.4 mg, 0.200 mmol, 1.0 eq.), $Pd(PPh_3)_2Cl_2$ (14.0 mg, 0.020 mmol, 0.1 eq.) and CuI (7.6 mg, 0.040 mmol, 1.0 eq.) were suspended in NH(*i*-Pr)$_2$ (0.5 mL). Ethynyltriisopropylsilane (54.7 mg, 0.300 mmol, 1.5 eq.) was added and the reaction mixture was stirred at 70˚C for 14 h. The reaction mixture was cooled to room temperature and was diluted with EtOAc (10 mL) and $H_2O$ (10 mL). The layers were separated and the aqueous layer was extracted with EtOAc (2x). The combined organic layers were washed with brine and dried over $Na_2SO_4$. The solvent was removed under reduced pressure and the crude product was purified by flash column chromatography (4% DCM in cyclohexane) to yield compound **13** as a colorless oil (79.1 mg, 0.186 mmol, 93%).

**$R_f$** = 0.32 (10% DCM in cyclohexane).

**$^1$H NMR** (500 MHz, $CDCl_3$): $\delta$ [ppm] = 7.50 – 7.44 (m, 2H, H-C13), 7.20 (t, $^3J_{HH}$ = 7.4 Hz, 1H, H-C7), 7.17 – 7.12 (m, 3H, H-C6, H-C12), 7.09 (ddd, $^3J_{HH}$ = 8.6 Hz, $^3J_{HH}$ = 7.3 Hz, $J_{HH}$ = 1.5 Hz, 1H, H-C8), 6.77 (d, $^3J_{HH}$ = 7.8 Hz, 1H, H-C9), 4.33 (dd, $^3J_{HH}$ = 12.0 Hz, $^3J_{HH}$ = 5.9 Hz, 1H, H-C1), 3.49 – 3.29 (m, 2H, H-C4), 2.55 (dtdd, $^2J_{HH}$ = 13.6 Hz, $^3J_{HF}$ = 8.0 Hz, $^3J_{HH}$ = 6.0 Hz, $J_{HH}$ = 2.5 Hz, 1H, H$^a$-C2), 2.24 (dddd, $^3J_{HF}$ = 32.0 Hz, $^2J_{HH}$ = 14.1 Hz, $^3J_{HH}$ = 12.0 Hz, $^3J_{HF}$ = 2.8 Hz, 1H, H$^b$-C2), 1.14 (s, 21H, H-C17, H-C18)

**$^{19}$F NMR** (470 MHz, $CDCl_3$): $\delta$ [ppm] = -91.38 (dtdd, $^{2}J_{FF}$ = 237.7 Hz, $^{3}J_{FH}$ = 10.7 Hz, $^{3}J_{FH}$ = 5.2 Hz, $^{3}J_{FH}$ = 2.3 Hz, 1F, $F^{a}$-C3), -98.72 (ddddd, $^{2}J_{FF}$ = 237.8 Hz, $^{3}J_{FH}$ = 32.0 Hz, $^{3}J_{FH}$ = 29.2 Hz, $^{3}J_{FH}$ = 16.3 Hz, $^{3}J_{FH}$ = 7.9 Hz, 1F, $F^{b}$-C3).

**$^{19}$F{$^{1}$H} NMR** (470 MHz, $CDCl_3$): $\delta$ [ppm] = -91.38 (d, $^{2}J_{FF}$ = 237.7 Hz, 1F, $F^{a}$-C3), -98.72 (d, $^{2}J_{FF}$ = 237.8 Hz, $F^{b}$-C3).

**$^{13}$C{$^{1}$H} NMR** (126 MHz, $CDCl_3$): $\delta$ [ppm] = 144.5 (C11), 137.3 (C10), 132.7 (C13), 132.1 (d, $^{3}J_{CF}$ = 10.3 Hz, C5), 129.3 (C9), 129.2 (d, $^{4}J_{CF}$ = 1.7 Hz, C6), 128.8 (C12), 127.1 (C7), 127.0 (C8), 122.5 (dd, $^{1}J_{CF}$ = 242.1 Hz, $^{1}J_{CF}$ = 239.2 Hz, C3), 122.4 (C14), 106.9 (C15), 90.8 (C16), 44.5 (dd, $^{3}J_{CF}$ = 8.2 Hz, $^{3}J_{CF}$ = 2.3 Hz, C1), 40.3 (dd, $^{2}J_{CF}$ = 24.7 Hz, $^{2}J_{CF}$ = 22.2 Hz, C2), 38.6 (dd, $^{2}J_{CF}$ = 27.2 Hz, $^{2}J_{CF}$ = 26.1 Hz, C4), 18.8 (C18), 11.5 (C17).

**GC-EI-MS:** Retention 12.06 min, (*m/z*) requires: [($C_{27}H_{34}F_2Si)^{+}$] = 424.2392, (*m/z*) found: [($C_{27}H_{34}F_2Si)^{+}$] = 424.2394.

**FT-IR** ($\tilde{\nu}$ = $cm^{-1}$)**:** 2943.0 (m), 2891.4 (w), 2864.1 (m), 2155.2 (w), 1503.8 (w), 1462.2 (w), 1424.9 (w), 1366.0 (w), 1315.8 (w), 1278.5 (w), 1267.0 (w), 1242.6 (w), 1221.1 (w), 1125.0 (m), 1086.2 (m), 1069.0 (s), 1046.1 (m), 1018.8 (m), 995.8 (w), 955.7 (w), 919.8 (w), 879.6 (m), 836.6 (s), 825.1 (s), 790.6 (w), 774.9 (w), 774.9 (w), 743.3 (s), 708.9 (w).

**5-(4-(3,3-Difluoro-1,2,3,4-tetrahydronaphthalen-1-yl)phenyl)benzo[d][1,3]dioxole (14)**

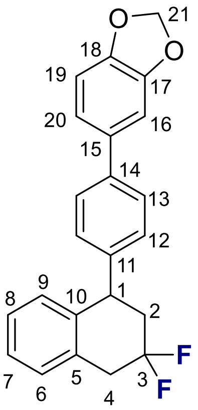


Compound **14** was prepared according to a literature procedure.[19]

1-(4-Bromophenyl)-3,3-difluoro-1,2,3,4-tetrahydronaphthalene **3q** (64.4 mg, 0.200 mmol, 1.0 eq.), benzo[d][1,3]dioxol-5-ylboronic acid (36.5 mg, 0.220 mmol, 1.1 eq.) and $Pd(PPh_3)_4$ (5.8 mg, 0.005 mmol, 0.025 eq.) were suspended in EtOH (0.6 mL). An aqueous solution of $Na_2CO_3$ (2 M, 0.12 mL, 0.24 mmol, 1.2 eq.) was added and the reaction mixture was stirred at 80˚C for 14 h. The reaction mixture was cooled to room temperature and was diluted with EtOAc (10 mL) and $H_2O$ (10 mL). The layers were separated and the aqueous layer was extracted with EtOAc (2x). The combined organic layers were dried over $Na_2SO_4$. The solvent was removed under reduced pressure and the crude product was purified by flash column chromatography (20-25% DCM in cyclohexane) to yield compound **14** as a colorless solid (60.5 mg, 0.166 mmol, 83%).

**$R_f$** = 0.32 (10% DCM in cyclohexane).

**Melting Point:** 155-157 ˚C.

**$^1$H NMR** (599 MHz, $CDCl_3$): $\delta$ [ppm] = 7.53 – 7.49 (m, 2H, H-C13), 7.26 – 7.23 (m, 2H, H-C12), 7.22 (t, $^3J_{HH}$ = 7.1 Hz, 1H, H-C7), 7.17 (dd, $^3J_{HH}$ = 7.8 Hz, $^4J_{HH}$ = 1.5 Hz, 1H, H-C6), 7.15 – 7.11 (m, 1H, H-C8), 7.10 (d, $^4J_{HH}$ = 1.8 Hz, 1H, H-C16), 7.09 (dd, $^3J_{HH}$ = 7.9 Hz, $^4J_{HH}$ = 1.8 Hz, 1H, H-C20), 6.90 (d, $^3J_{HH}$ = 7.9 Hz, 1H, H-C19), 6.88 (d, $^3J_{HH}$ = 8.1 Hz, 1H, H-C9), 6.01 (s, 2H, H-C21), 4.39 (dd, $^3J_{HH}$ = 12.0 Hz, $^3J_{HH}$ = 5.9 Hz, 1H, H-C1), 3.51 – 3.34 (m, 2H, H-C4), 2.62 (ddddd, $^2J_{HH}$ = 13.4 Hz, $^3J_{HF}$ = 11.1 Hz, $^3J_{HF}$ = 8.2 Hz, $^3J_{HH}$ = 6.0 Hz, $J_{HH}$ = 2.5 Hz, 1H, $H^a$-C2), 2.33 (dddd, $^3J_{HF}$ = 32.2 Hz, $^3J_{HH}$ = 13.9 Hz, $^2J_{HH}$ = 11.9 Hz, $^3J_{HF}$ = 2.8 Hz, 1H, $H^b$-C2).

**$^{19}$F NMR** (564 MHz, $CDCl_3$): $\delta$ [ppm] = -91.20 (ddddd, $^2J_{FF}$ = 237.3 Hz, $^3J_{FH}$ = 10.6 Hz, $^3J_{FH}$ = 8.5 Hz, $^3J_{FH}$ = 5.3 Hz, $^3J_{FH}$ = 2.4 Hz, 1F, $F^a$-C3), -98.65 (ddddd, $^2J_{FF}$ = 237.4 Hz, $^3J_{FH}$ = 32.2 Hz, $^3J_{FH}$ = 29.2 Hz, $^3J_{FH}$ = 16.3 Hz, $^3J_{FH}$ = 8.0 Hz, 1F, $F^b$-C3).

**$^{19}$F{$^1$H} NMR** (564 MHz, $CDCl_3$): $\delta$ [ppm] = -91.20 (d, $^2J_{FF}$ = 237.3 Hz, 1F, $F^a$-C3), -98.65 (d, $^2J_{FF}$ = 237.4 Hz, $F^b$-C3).

**$^{13}$C{$^1$H} NMR** (151 MHz, $CDCl_3$): $\delta$ [ppm] = 148.3 (C17), 147.2 (C18), 142.9 (C11), 139.7 (C14), 137.7 (d, $^4J_{CF}$ = 1.5 Hz, C10), 135.2 (C15), 132.1 (d, $^3J_{CF}$ = 10.1 Hz, C5), 129.4 (C9), 129.3 (C12), 129.2 (d, $^4J_{CF}$ = 1.8 Hz, C6), 127.4 (C13), 126.98 (C8), 126.97 (C7), 122.6 (dd, $^1J_{CF}$ = 241.9 Hz, $^1J_{CF}$ = 239.1 Hz, C3), 120.7 (C20), 108.7 (C19), 107.7 (C16), 101.3 (C21), 44.2 (dd, $^3J_{CF}$ = 8.2 Hz, $^3J_{CF}$ = 2.2 Hz, C1), 40.5 (dd, $^2J_{CF}$ = 24.7 Hz, $^2J_{CF}$ = 22.0 Hz, C2), 38.7 (dd, $^2J_{CF}$ = 27.3 Hz, $^2J_{CF}$ = 25.9 Hz, C4).

**GC-EI-MS:** Retention 12.49 min, (*m/z*) requires: [$(C_{23}H_{18}F_2O_2)^+$] = 364.1269, (*m/z*) found: [$(C_{23}H_{18}F_2O_2)^+$] = 364.1269.

**FT-IR** ($\tilde{\nu}$ = $cm^{-1}$)**:** 2960.2 (w), 2887.1 (w), 1502.4 (m), 1490.9 (m), 1452.1 (w), 1442.1 (w), 1416.3 (w), 1370.3 (w), 1340.2 (w), 1318.7 (w), 1279.9 (w), 1234.0 (m), 1208.2 (w), 1126.4 (m), 1110.6 (m), 1086.2 (m), 1064.7 (m), 1041.8 (s), 958.5 (m), 938.4 (s), 889.7 (m), 879.6 (w), 863.8 (w), 845.2 (m), 830.8 (m), 802.1 (s), 773.4 (m), 749.0 (s), 731.8 (m), 714.6 (w), 701.7 (w).

**1-(4-(3,3-Difluoro-1,2,3,4-tetrahydronaphthalen-1-yl)phenyl)-1H-indole (15)**

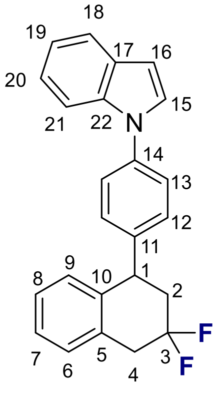


Compound **15** was prepared according to a literature procedure.[20]

$K_3PO_4$ (89.2 mg, 0.420 mmol, 2.1 eq.) was dried at 500 ˚C for 5 minutes in a pressure tube in vacuo. After cooling to room temperature, 1-(4-bromophenyl)-3,3-difluoro-1,2,3,4-tetrahydronaphthalene **3q** (64.4 mg, 0.200 mmol, 1.0 eq.), indole (23.4 mg, 0.200 mmol, 1.0 eq.) and CuI (3.8 mg, 0.020 mmol, 0.1 eq.) were added. The reaction vessel was flushed with Argon for three times before toluene (0.5 mL) and *N$^1$,N$^2$*-dimethylethane-1,2-diamine (7.0 mg, 0.080 mmol, 0.4 eq.) were added. The reaction mixture was sealed and heated to 110 ˚C for 20 h. The reaction mixture was

cooled to room temperature and was diluted with EtOAc (10 mL) and $H_2O$ (10 mL). The layers were separated and the aqueous layer was extracted with EtOAc (2x). The combined organic layers were dried over $Na_2SO_4$. The solvent was removed under reduced pressure and the crude product was purified by flash column chromatography (10-15% DCM in cyclohexane) to yield compound **15** as an orange solid (69.8 mg, 0.194 mmol, 97%).

**$R_f$** = 0.12 (10% DCM in cyclohexane).

**Melting Point:** 47-49 ˚C.

**$^1$H NMR** (500 MHz, $CDCl_3$): $\delta$ [ppm] = 7.71 (dt, $^3J_{HH}$ = 7.8 Hz, $J_{HH}$ = 1.1 Hz, 1H, H-C18), 7.61 (dq, $^3J_{HH}$ = 8.3 Hz, $J_{HH}$ = 0.9 Hz, 1H, H-C21), 7.52 – 7.48 (m, 2H, H-C13), 7.38 – 7.33 (m, 3H, H-C12, H-C15), 7.27 – 7.22 (m, 2H, H-C7, H-C19), 7.21 – 7.14 (m, 3H, H-C6, H-C8, H-C20), 6.91 (dt, $^3J_{HH}$ = 7.8 Hz, $J_{HH}$ = 1.3 Hz, 1H, H-C9), 6.70 (dd, $^3J_{HH}$ = 3.3 Hz, $J_{HH}$ = 0.8 Hz, 1H, H-C16), 4.45 (dd, $^3J_{HH}$ = 11.8 Hz, $^3J_{HH}$ = 6.0 Hz, 1H, H-C1), 3.55 – 3.34 (m, 2H, H-C4), 2.66 (ddddd, $^2J_{HH}$ = 13.3 Hz, $^3J_{HF}$ = 11.0 Hz, $^3J_{HF}$ = 8.0 Hz $^3J_{HH}$ = 6.1 Hz, $J_{HH}$ = 2.3 Hz, 1H, $H^a$-C2), 2.35 (dddd, $^3J_{HF}$ = 31.6 Hz, $^2J_{HH}$ = 14.5 Hz, $^3J_{HH}$ = 11.7 Hz, $^3J_{HF}$ = 2.8 Hz, 1H, $H^b$-C2).

**$^{19}$F NMR** (376 MHz, $CDCl_3$): $\delta$ [ppm] = -91.34 (ddddd, $^2J_{FF}$ = 237.8 Hz, $^3J_{FH}$ = 10.5 Hz, $^3J_{FH}$ = 8.2 Hz, $^3J_{FH}$ = 5.3 Hz, $^3J_{FH}$ = 2.5 Hz, 1F, $F^a$-C3), -98.53 (ddddd, $^2J_{FF}$ = 237.7 Hz, $^3J_{FH}$ = 31.6 Hz, $^3J_{FH}$ = 27.7 Hz, $^3J_{FH}$ = 17.3 Hz, $^3J_{FH}$ = 7.9 Hz, 1F, $F^b$-C3).

**$^{19}$F{$^1$H} NMR** (376 MHz, $CDCl_3$): $\delta$ [ppm] = -91.34 (d, $^2J_{FF}$ = 237.8 Hz, 1F, $F^a$-C3), -98.53 (d, $^2J_{FF}$ = 237.7 Hz, $F^b$-C3).

**$^{13}$C{$^1$H} NMR** (126 MHz, $CDCl_3$): $\delta$ [ppm] = 142.5 (C11), 138.8 (C14), 137.6 (d, $^4J_{CF}$ = 1.5 Hz, C10), 136.0 (C22), 132.2 (d, $^3J_{CF}$ = 10.8 Hz, C5), 130.1 (C12), 129.5 (C17), 129.4 (d, $^5J_{CF}$ = 0.9 Hz, C9), 129.3 (d, $^4J_{CF}$ = 1.7 Hz, C6), 128.0 (C15), 127.2 (C7), 127.1 (C8), 124.8 (C13), 122.53 (dd, $^1J_{CF}$ = 241.9 Hz, $^1J_{CF}$ = 239.6 Hz, C3), 122.51 (C19), 121.3 (C18), 120.5 (C20), 110.7 (C21), 103.8 (C16), 44.2 (dd, $^3J_{CF}$ = 8.1 Hz, $^3J_{CF}$ = 2.4 Hz, C1), 40.6 (dd, $^2J_{CF}$ = 24.7 Hz, $^2J_{CF}$ = 22.2 Hz, C2), 38.7 (dd, $^2J_{CF}$ = 27.3 Hz, $^2J_{CF}$ = 25.9 Hz, C4).

**ESI-MS:** (*m/z*) requires: $[(C_{24}H_{19}F_2NH)^+]$ = 360.1558, (*m/z*) found: $[(C_{24}H_{19}F_2NH)^+]$ = 360.1558.

**FT-IR** ($\tilde{\nu}$ = cm$^{-1}$)**:** 1607.1 (w), 1516.7 (s), 1493.8 (w), 1475.1 (w), 1456.4 (s), 1423.4 (w), 1367.5 (m), 1348.8 (m), 1333.0 (m), 1315.9 (m), 1278.5 (m), 1265.6 (m), 1232.6 (m), 1212.5 (m), 1135.0 (m), 1123.5 (m), 1086.2 (m), 1066.1 (s), 1044.6 (m), 955.7 (m), 908.3 (w), 878.2 (m), 846.6 (m), 829.4 (m), 803.6 (w), 776.3 (m), 761.9 (m), 737.6 (s).

## X-Ray Crystallographic Data

Data sets for compounds ***minor*-1c, 2e** and **12** were collected with a Bruker D8 Venture Photon III Diffractometer. Programs used: data collection: *APEX4* Version 2021.4-0 [21] (Bruker AXS Inc., **2021**); cell refinement: *SAINT* Version 8.40B (Bruker AXS Inc., **2021**); data reduction: *SAINT* Version 8.40B (Bruker AXS Inc., **2021**); absorption correction, *SADABS* Version 2016/2 (Bruker AXS Inc., **2021**); structure solution *SHELXT*-Version 2018-3 [22]; structure refinement *SHELXL*-Version 2018-3 [23] and graphics, *XP* [24]. *R*-values are given for observed reflections, and $wR^2$ values are given for all reflections.

**Exceptions and Special Features:**

For compound **2e**, parts of the trifluorobutane chain and the $CF_3$ group (in ratio 80:20) and for compound **12** parts of the 1-aryltetrahydronaphthalene-backbone (in ratio 84:16) were found disordered over two positions in the asymmetric unit. Several restraints (SADI, SAME, ISOR and SIMU) were used in order to improve refinement stability.

For compound ***minor*-1c** the hydrogens at O1A and O1B atoms and for compound **12** the hydrogen at O3 atom were refined freely.

**X-Ray Crystal Structure Analysis of *minor*-1c:**

A colourless, prism-like specimen of $C_{16}H_{14}Cl_2O$, approximate dimensions 0.128 mm x 0.150 mm x 0.169 mm, was used for the X-ray crystallographic analysis. The X-ray intensity data were measured on a single crystal diffractometer Bruker D8 Venture Photon III system equipped with a micro focus tube Cu ImS (CuKα, λ = 1.54178 Å) and a MX mirror monochromator. A total of 1778 frames were collected. The total exposure time was 21.81 hours. The frames were integrated with the Bruker SAINT software package using a wide-frame algorithm. The integration of the data using a monoclinic unit cell yielded a total of 53010 reflections to a maximum θ angle of 68.26° (0.83 Å resolution), of which 5186 were independent (average redundancy 10.222, completeness = 99.2%, $R_{int}$ = 4.86%, $R_{sig}$ = 2.29%) and 4901 (94.50%) were greater than 2σ($F^2$). The final cell constants of a = 23.0035(5) Å, b = 13.2204(3) Å, c = 19.4633(4) Å, β = 105.1850(10)°, volume = 5712.4(2) $Å^3$, are based upon the refinement of the XYZ-centroids of 9926 reflections above 20 σ(I) with 9.624° < 2θ < 136.4°. Data were corrected for absorption effects using the Multi-Scan method (SADABS). The ratio of minimum to maximum apparent transmission was 0.882. The calculated minimum and maximum transmission coefficients (based on crystal size) are 0.5520 and 0.6290. The structure was solved and refined using the Bruker SHELXTL Software Package, using the space group *C*2/*c*, with Z = 16 for the formula unit, $C_{16}H_{14}Cl_2O$. The final anisotropic full-matrix least-squares refinement on $F^2$ with 351 variables converged at R1 = 3.41%, for the observed data and wR2 = 8.92% for all data. The goodness-of-fit was 1.042. The largest peak in the final difference electron density synthesis was 0.285 $e^-/Å^3$ and the largest hole was -0.563 $e^-/Å^3$ with an RMS deviation of 0.045 $e^-/Å^3$. On the basis of the final model, the calculated density was 1.364 g/cm$^3$ and F(000), 2432 $e^-$. The hydrogens at O1A and O1B atoms were refined freely. CCDC Nr.: 2239011.

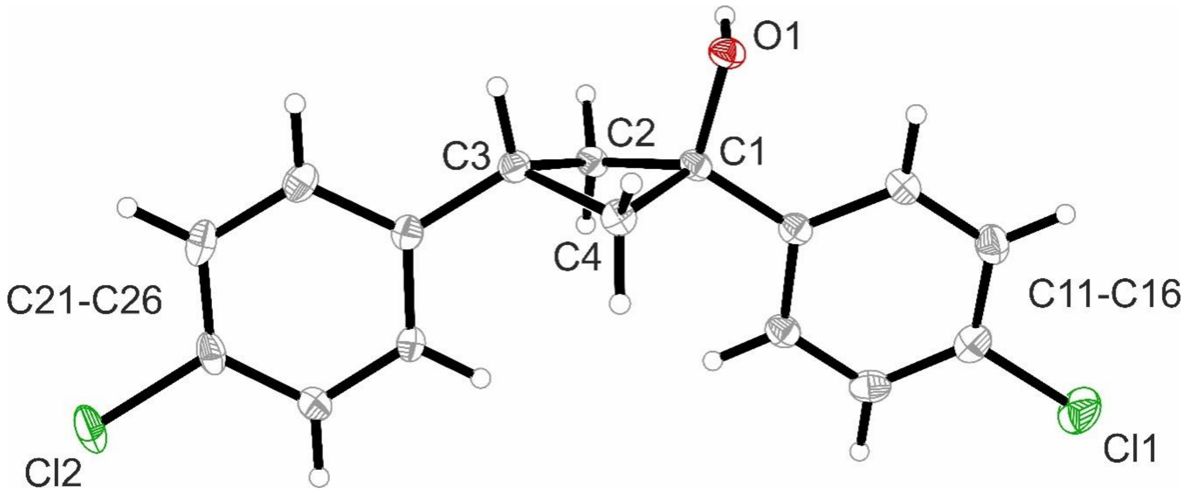


**Supplementary Figure 4:** Crystal structure of compound ***minor*-1c**. Thermal ellipsoids are shown at 50% probability. Only one molecule (molecule “A”) of two found in the asymmetric unit is shown.

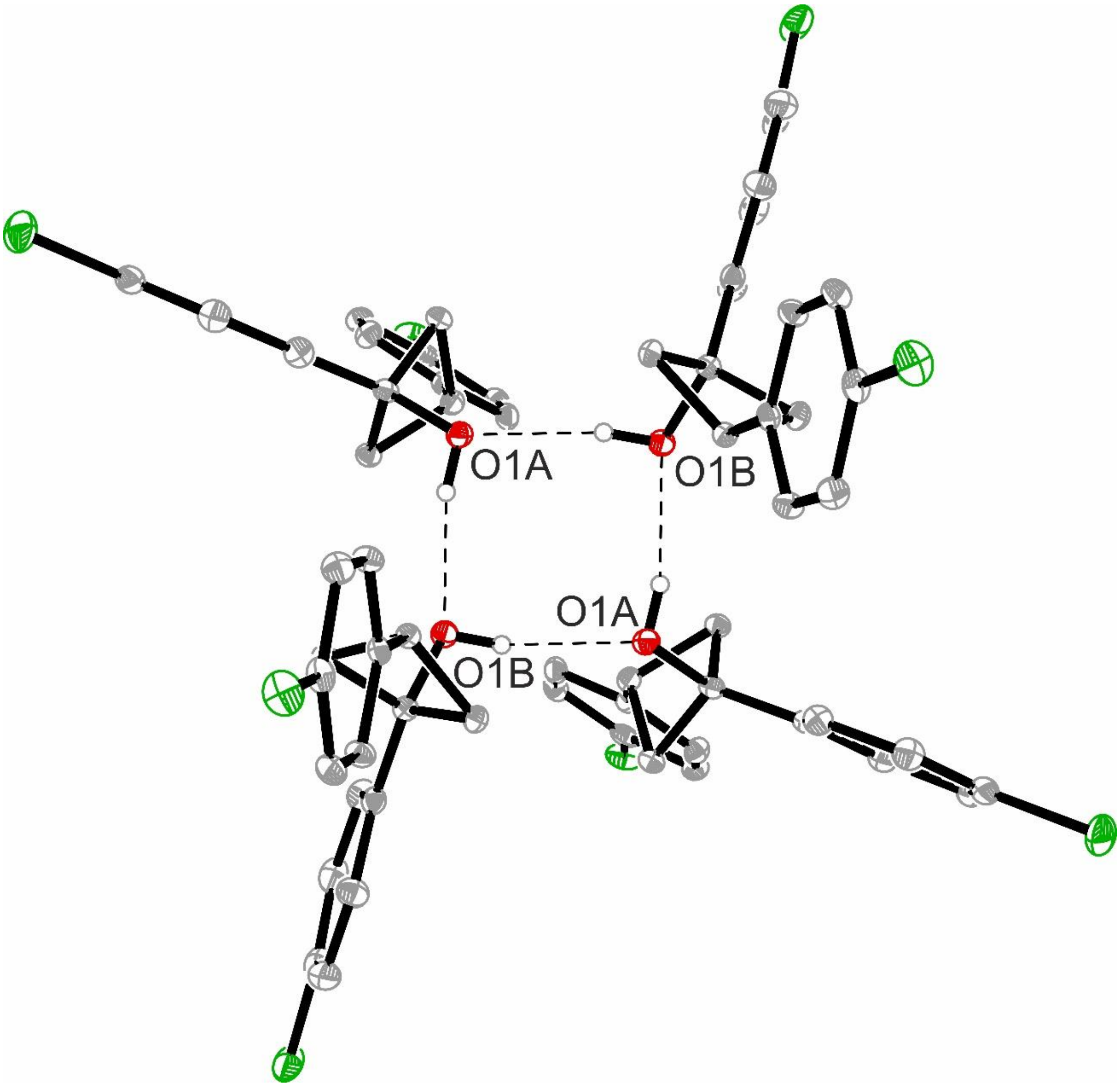


**Supplementary Figure 5.** View representing the tetramer formation involving the O-H···O hydrogen bond interactions between the molecules of compound ***minor*-1c**.

**Supplementary Table 1.** Non-covalent intermolecular interactions in compound ***minor*-1c** (Å and deg).

| *D*-H···*A* | *d*(*D*-H) | *d*(H···*A*) | *d*(*D*···*A*) | ∠(*DHA*) |
|---|---|---|---|---|
| O1A-H1A···O1B | 0.80(2) | 1.91(2) | 2.688(2) | 167(2) |
| O1B-H1B···O1A[#1] | 0.80(3) | 1.95(3) | 2.725(2) | 164(2) |

Symmetry transformations used to generate equivalent atoms: [#1] -x+1, y, -z+3/2.

**X-Ray Crystal Structure Analysis of 2e:**

A colourless, plate-like specimen of $C_{18}H_{13}F_9$, approximate dimensions 0.040 mm x 0.110 mm x 0.223 mm, was used for the X-ray crystallographic analysis. The X-ray intensity data were measured on a single crystal diffractometer Bruker D8 Venture Photon III system equipped with a micro focus tube Cu ImS (CuKα, λ = 1.54178 Å) and a MX mirror monochromator. A total of 1691 frames were collected. The total exposure time was 22.24 hours. The frames were integrated with the Bruker SAINT software package using a wide-frame algorithm. The integration of the data using a triclinic unit cell yielded a total of 15096 reflections to a maximum θ angle of 66.66° (0.84 Å resolution), of which 2854 were independent (average redundancy 5.289, completeness = 98.5%, $R_{int}$ = 4.20%, $R_{sig}$ = 3.01%) and 2614 (91.59%) were greater than 2σ($F^2$). The final cell constants of a = 9.9491(2) Å, b = 9.9690(2) Å, c = 10.0269(2) Å, α = 67.9530(10)°, β = 67.5970(10)°, γ = 68.2140 (10)°, volume = 821.46(3) Å$^3$, are based upon the refinement of the XYZ-centroids of 9716 reflections above 20 σ(I) with 9.916° < 2θ < 133.3°. Data were corrected for absorption effects using the Multi-Scan method (SADABS). The ratio of minimum to maximum apparent transmission was 0.878. The calculated minimum and maximum transmission coefficients (based on crystal size) are 0.7330 and 0.9430. The structure was solved and refined using the Bruker SHELXTL Software Package, using the space group *P*-1, with Z = 2 for the formula unit, $C_{18}H_{13}F_9$. The final anisotropic full-matrix least-squares refinement on $F^2$ with 405 variables converged at R1 = 4.86%, for the observed data and wR2 = 12.81% for all data. The goodness-of-fit was 1.054. The largest peak in the final difference electron density synthesis was 0.420 e$^-$/Å$^3$ and the largest hole was -0.225 e$^-$/Å$^3$ with an RMS deviation of 0.044 e$^-$/Å$^3$. On the basis of the final model, the calculated density was 1.618 g/cm$^3$ and F(000), 404 e$^-$. CCDC Nr.: 2239010.

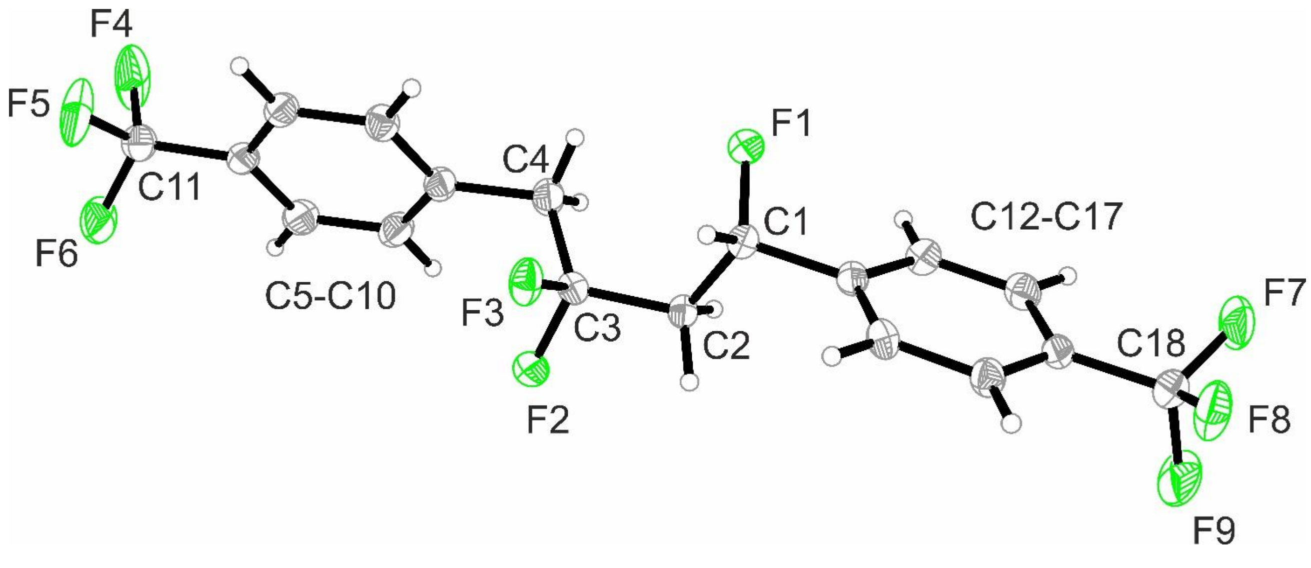


**Supplementary Figure 6:** Crystal structure of compound **2e** representing the main conformation found in the asymmetric unit (80%). Thermal ellipsoids are shown at 50% probability.

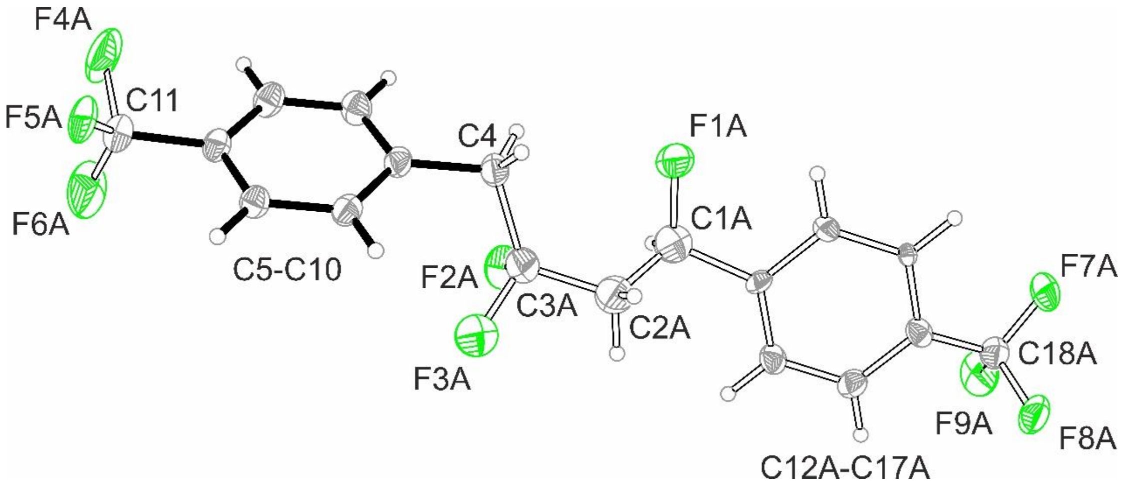


**Supplementary Figure 7.** Crystal structure of compound **2e** representing the second conformation found in the asymmetric unit (20%). Thermal ellipsoids are shown at 50% probability.

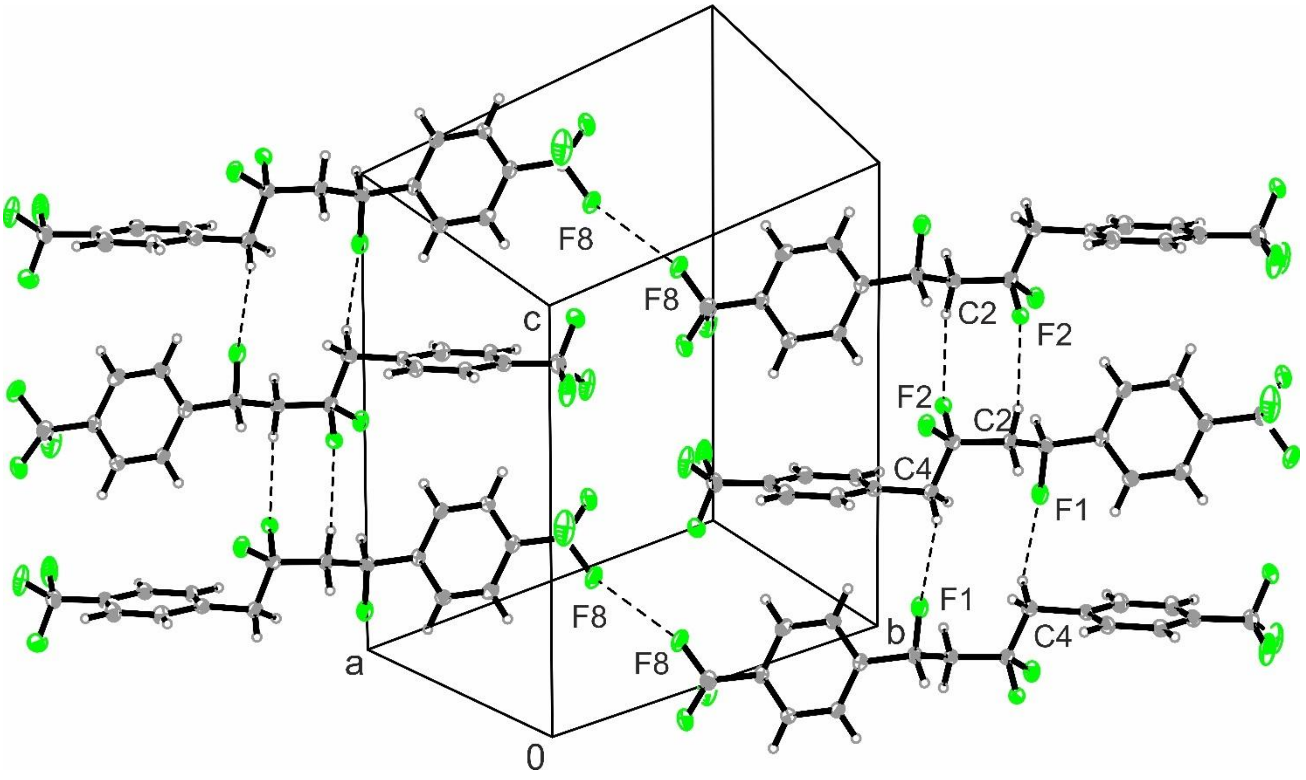


**Supplementary Figure 8.** Excerpt of the packing diagram of compound **2e** presenting the C-H···F and F···F interactions perpendicular on the *ab*-diagonal.

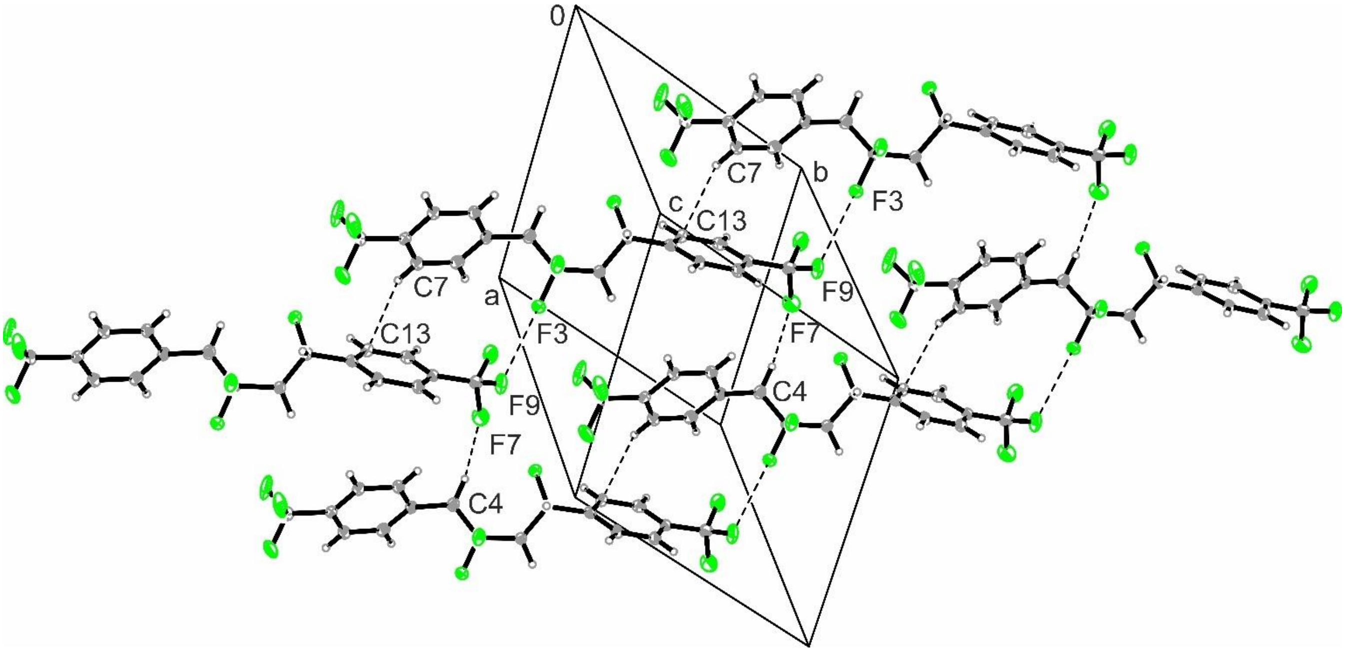


**Supplementary Figure 9.** Excerpt of the packing diagram of compound **2e** presenting the C-H···F, F···F and C-H···π interactions along the *ab*-diagonal.

**Supplementary Table 2.** Non-covalent intermolecular interactions in compound **2e** (Å and deg).

| *D*-H···*A* | *d*(*D*-H) | *d*(H···*A*) | *d*(*D*···*A*) | ∠(*D*H*A*) |
|---|---|---|---|---|
| C4-H4A···F1#1 | 0.99 | 2.43 | 3.026(3) | 118.3 |
| C2-H2B···F2#2 | 0.99 | 2.42 | 3.280(3) | 145.3 |
| F8···F8#3 | | | 2.862(2) | |
| F3···F9#4 | | | 2.873(3) | |
| C4-H4B···F7#5 | 0.99 | 2.52 | 3.480(3) | 162.7 |
| C7-H7···*Cg*1#4 | 0.95 | 2.85 | 3.746(4) | 158.2 |

Symmetry transformations used to generate equivalent atoms: #1 -x+1, -y+1, -z; #2 -x+1, -y+1, -z+1; #3 -x, y+3, -z-1; #4 x+1, y-1, z; #5 x, y+1, z. *Cg*1 represent the shortest distance between two aromatic phenyl rings C7-H7···C13 3.746 Å.

**X-Ray Crystal Structure Analysis of 12:**

A colourless, prism-like specimen of $C_{20}H_{20}F_2O_3$, approximate dimensions 0.074 mm x 0.096 mm x 0.139 mm, was used for the X-ray crystallographic analysis. The X-ray intensity data were measured on a single crystal diffractometer Bruker D8 Venture Photon III system equipped with a micro focus tube Cu ImS (CuKα, λ = 1.54178 Å) and a MX mirror monochromator. A total of 1616 frames were collected. The total exposure time was 21.28 hours. The frames were integrated with the Bruker SAINT software package using a wide-frame algorithm. The integration of the data using a monoclinic unit cell yielded a total of 28111 reflections to a maximum θ angle of 67.27° (0.84 Å resolution), of which 2954 were independent (average redundancy 9.516, completeness = 98.4%, $R_{int}$ = 11.15%, $R_{sig}$ = 5.40%) and 2595 (87.85%) were greater than 2σ($F^2$). The final cell constants of a = 12.7414(4) Å, b = 13.0405(4) Å, c = 10.1357(3) Å, β = 96.769(2)°, volume = 1672.35(9) $Å^3$, are based upon the refinement of the XYZ-centroids of 9990 reflections above 20 σ(I) with 6.778° < 2θ < 133.8°. Data were corrected for absorption effects using the Multi-Scan method (SADABS). The ratio of minimum to maximum apparent transmission was 0.877. The calculated minimum and maximum transmission coefficients (based on crystal size) are 0.8860 and 0.9370. The structure was solved and refined using the Bruker SHELXTL Software Package, using the space group $P2_1/c$, with Z = 4 for the formula unit, $C_{20}H_{20}F_2O_3$. The final anisotropic full-matrix least-squares refinement on $F^2$ with 386 variables converged at R1 = 9.98%, for the observed data and wR2 = 22.99% for all data. The goodness-of-fit was 1.152. The largest peak in the final difference electron density synthesis was 0.348 $e^-/Å^3$ and the largest hole was -0.374 $e^-/Å^3$ with an RMS deviation of 0.085 $e^-/Å^3$. On the basis of the final model, the calculated density was 1.376 g/cm$^3$ and F(000), 728 $e^-$. The hydrogen at O3 atom was refined freely. CCDC Nr.: 2239012.

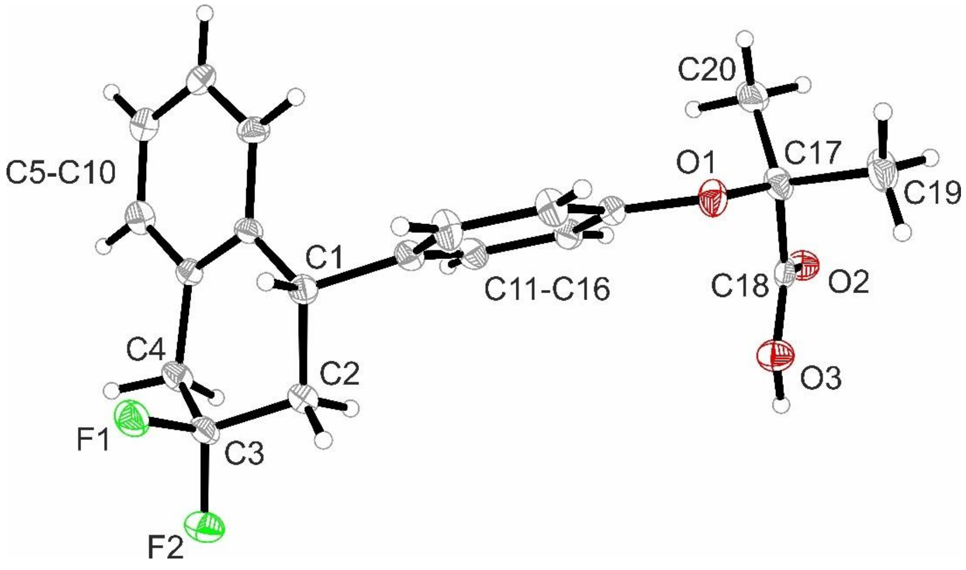


**Supplementary Figure 10.** Crystal structure of compound **12** representing the main conformation found in the asymmetric unit (84%). Thermal ellipsoids are shown at 50% probability.

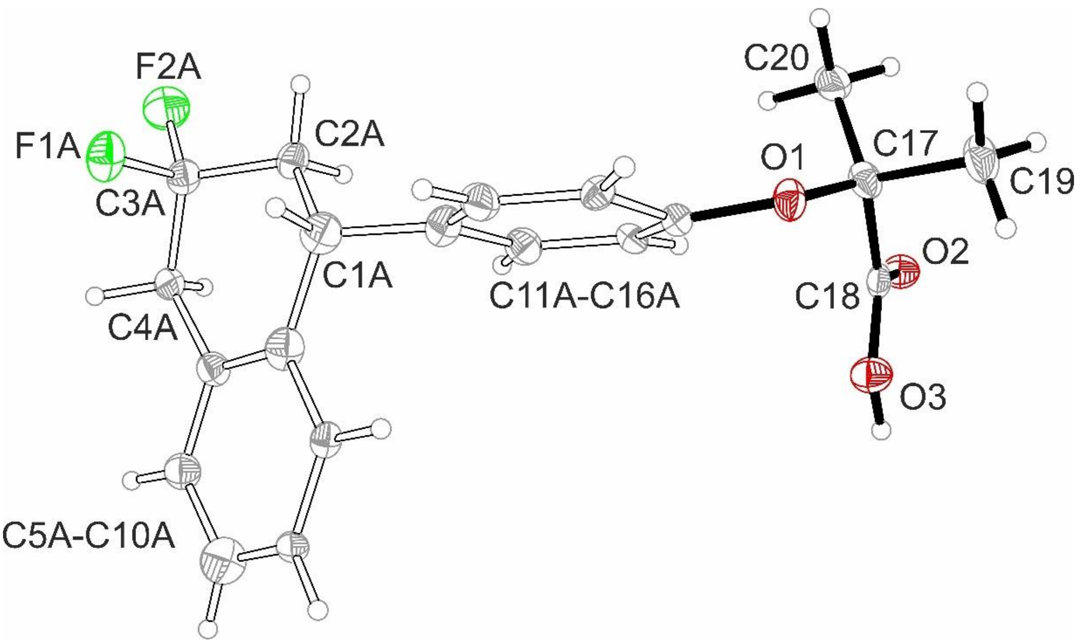


**Supplementary Figure 11.** Crystal structure of compound **12** representing the second conformation found in the asymmetric unit (16%). Thermal ellipsoids are shown at 50% probability.

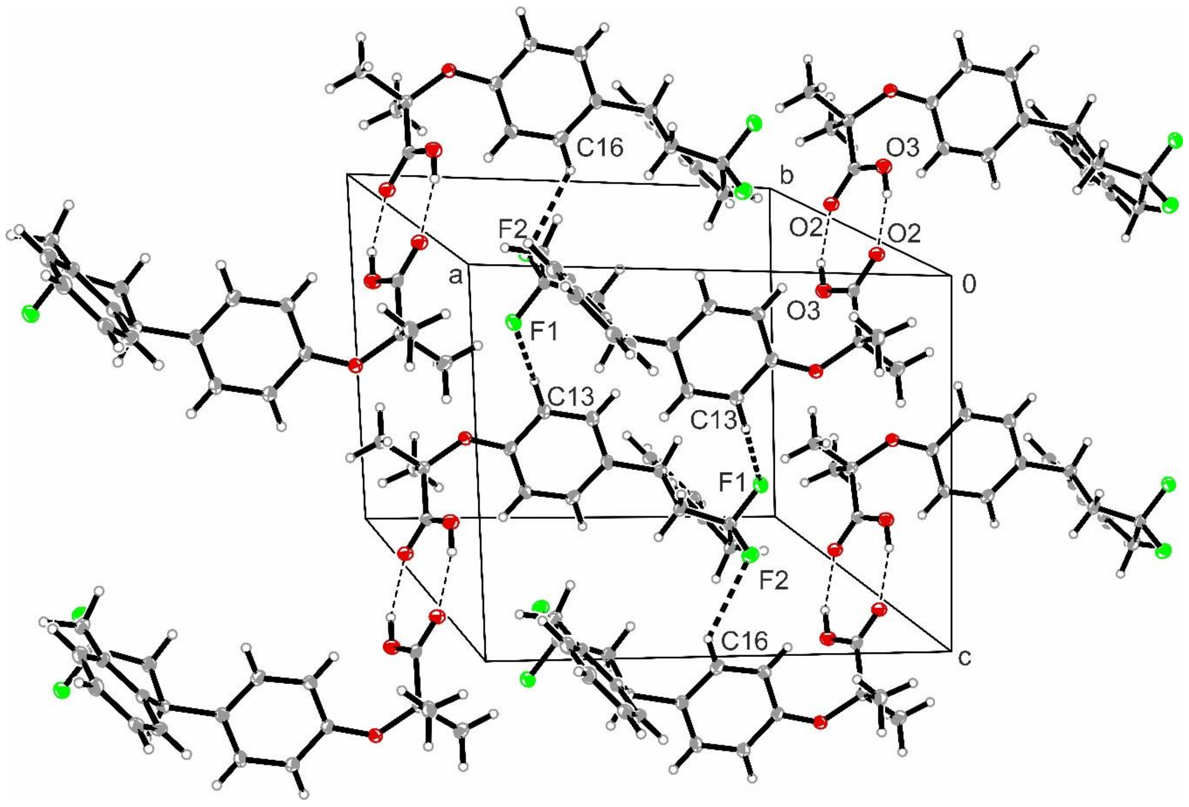


**Supplementary Figure 12.** View perpendicular to ac-plane presenting the C-H···F and O-H···O interactions between the dimeric units of compound **12**.

**Supplementary Table 3.** Non-covalent intermolecular interactions in compound **12** (Å and deg).

| *D*-H···*A* | *d*(*D*-H) | *d*(H···*A*) | *d*(*D*···*A*) | ∠(*D*H*A*) |
|---|---|---|---|---|
| O3-H3···O2[#1] | 0.87(8) | 1.88(8) | 2.729(5) | 165(7) |
| C13-H13···F1[#2] | 0.95 | 2.64 | 3.506(8) | 152.3 |
| C16-H16···F2[#3] | 0.95 | 2.58 | 3.216(8) | 125.0 |
| C8-H8···*Cg*1[#4] | 0.95 | 2.68 | 3.621(9) | 169.8 |

Symmetry transformations used to generate equivalent atoms: [#1] -x, -y+1, -z+1; [#2] -x+1, -y+1, -z+1; [#3] -x+1, -y+1, -z+1; [#4] -x+1, y-0.5, -z+1.5 . *Cg*1 represent the centroid of the phenyl ring involving the C11/C16/C15 atoms.

# NMR Spectra

## 4-(3-Oxocyclobutyl)phenyl trifluoromethanesulfonate (S11)

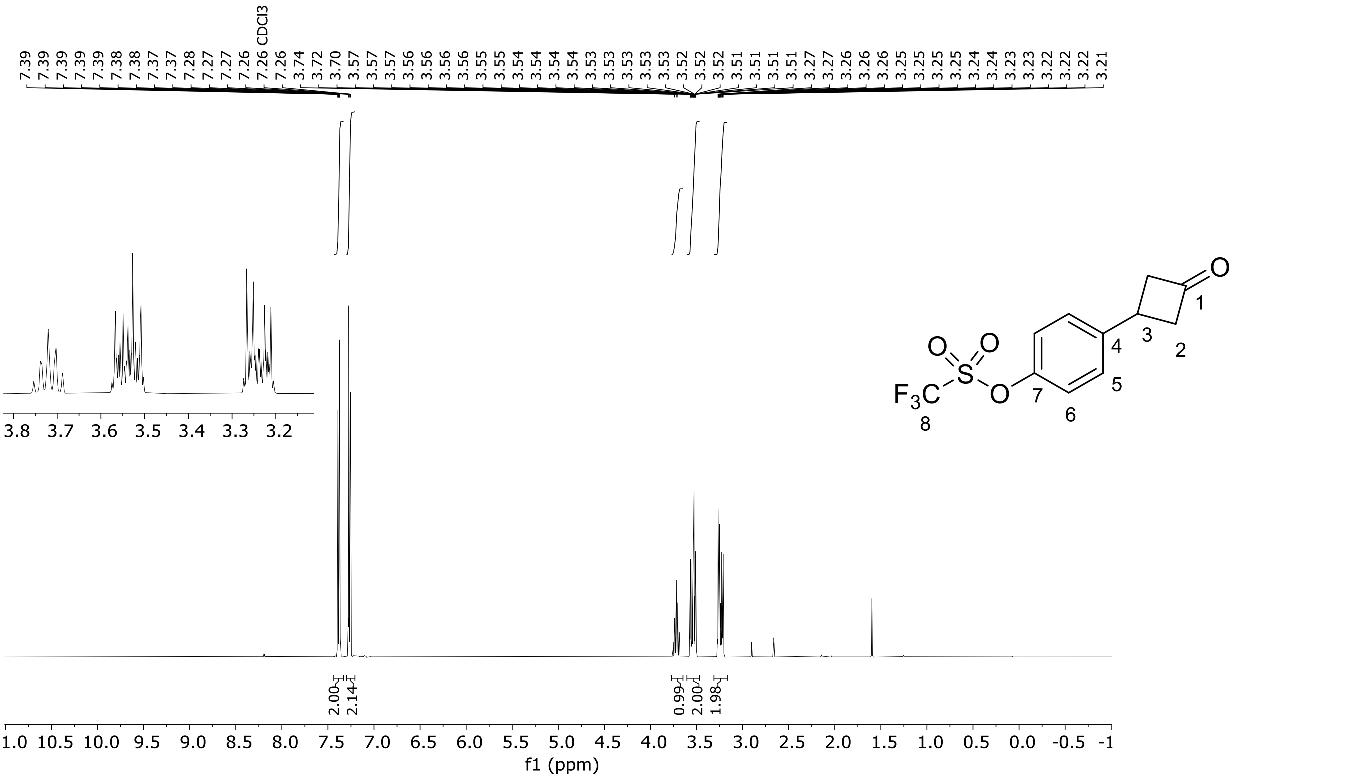

**Supplementary Figure 13.** $^{1}H$ NMR of **S11** (500 MHz, 299 K, $CDCl_3$).

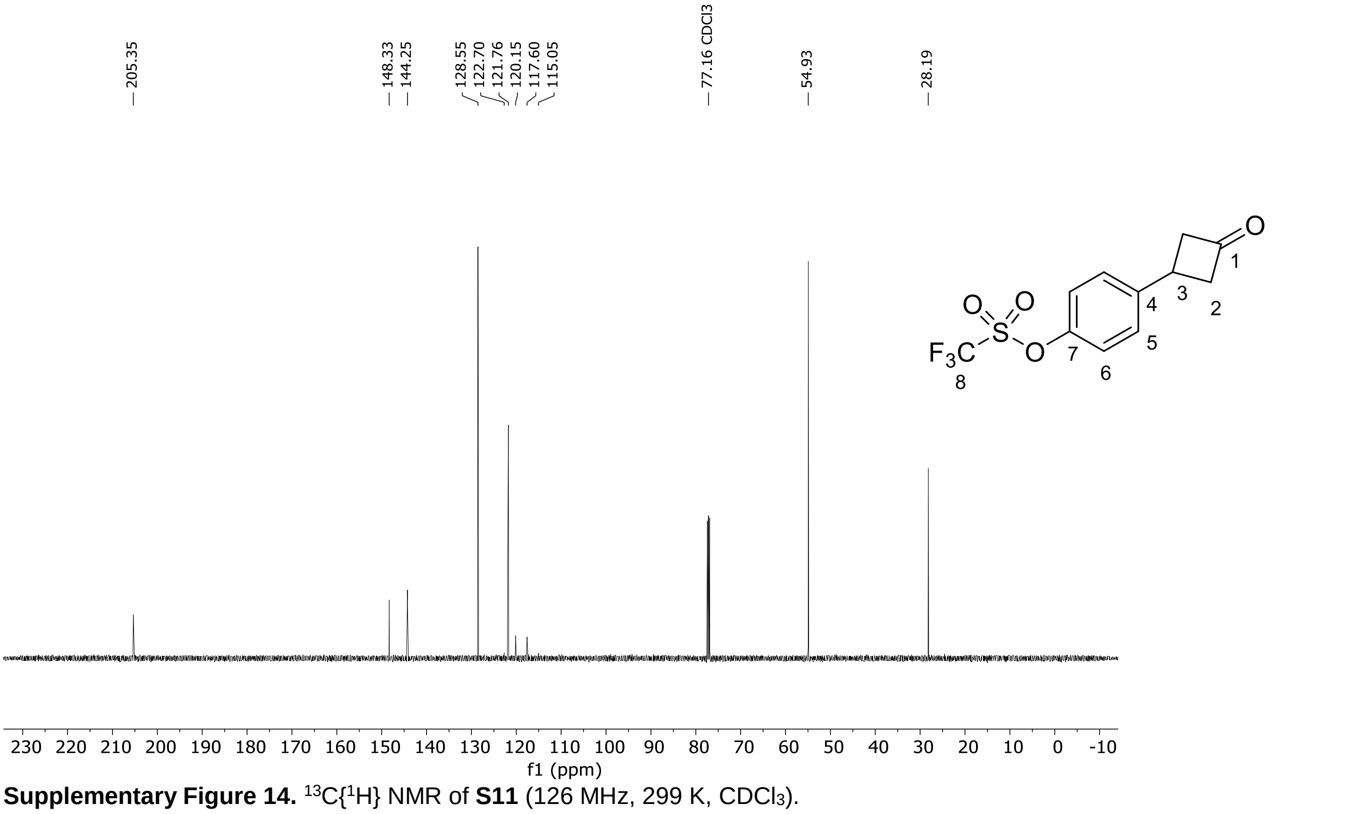

**Supplementary Figure 14.** $^{13}C\{^{1}H\}$ NMR of **S11** (126 MHz, 299 K, $CDCl_3$).

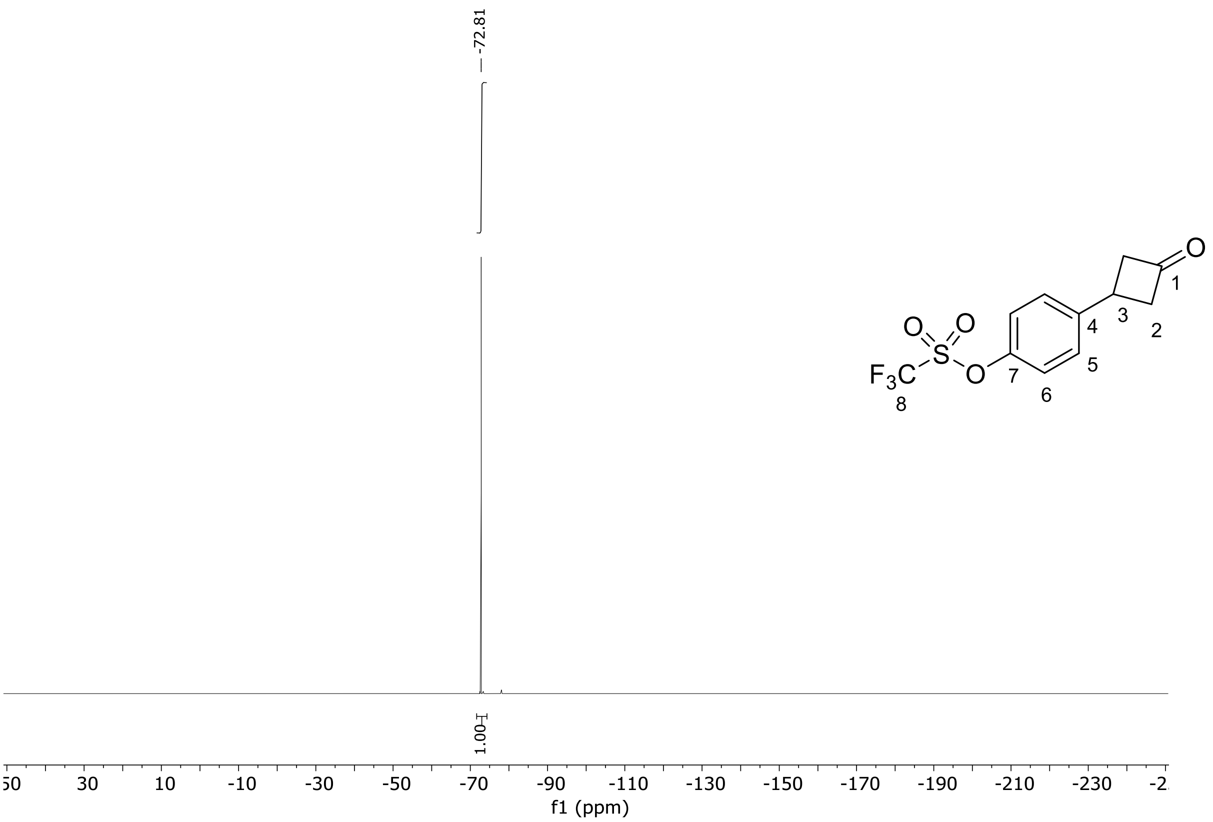


**Supplementary Figure 15.** $^{19}$F NMR of **S11** (376 MHz, 299 K, $CDCl_3$).

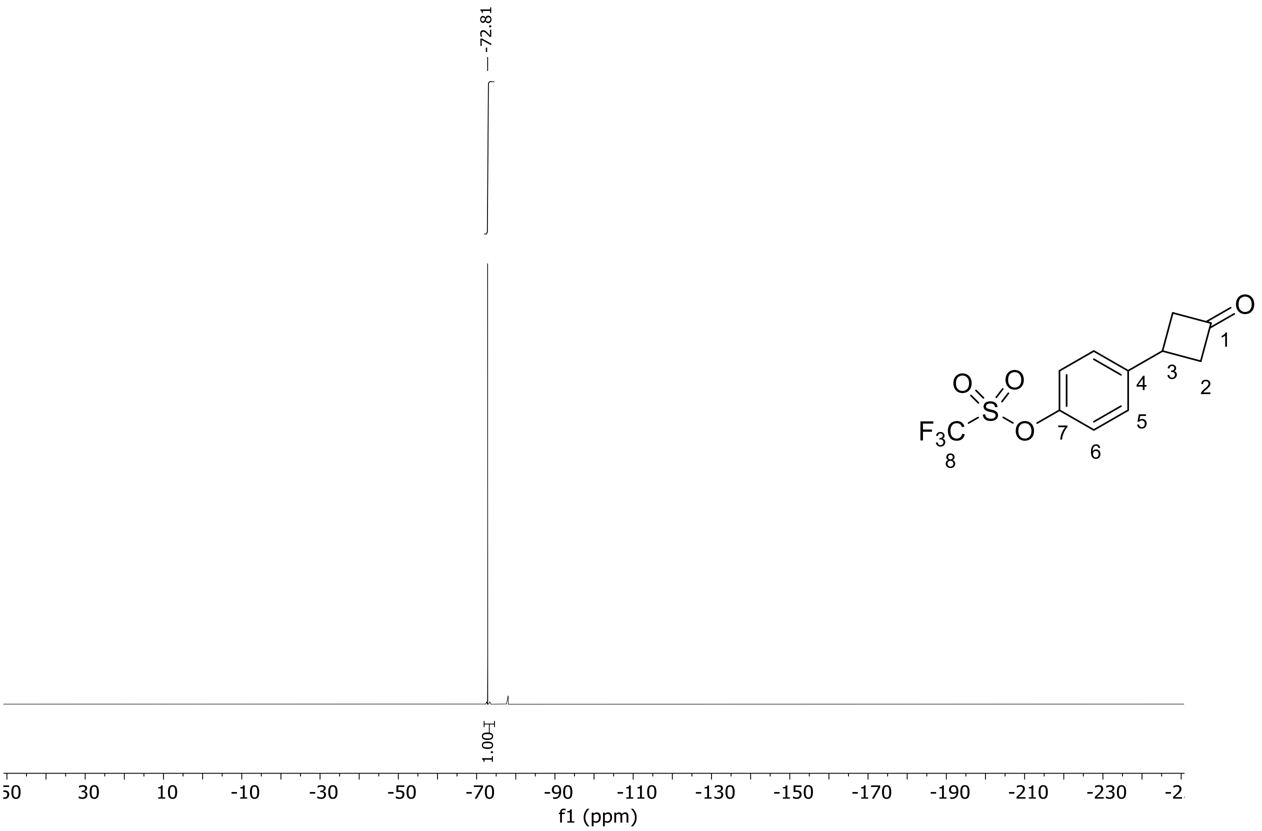


**Supplementary Figure 16.** $^{19}$F{$^1$H} NMR of **S11** (376 MHz, 299 K, $CDCl_3$).

## 1,3-Bis(4-fluorophenyl)cyclobutan-1-ol (1b)

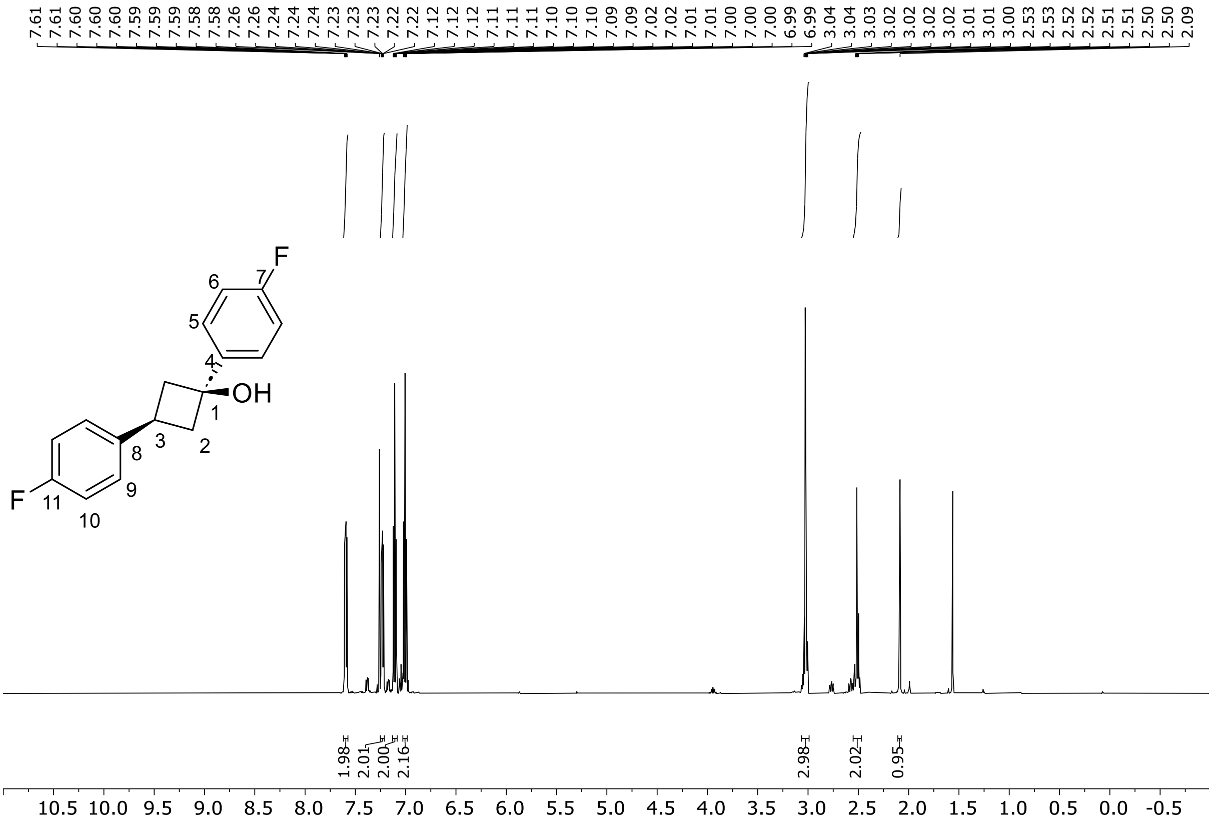


**Supplementary Figure 17.** $^{1}H$ NMR of **1b** (599 MHz, 299 K, $CDCl_3$).

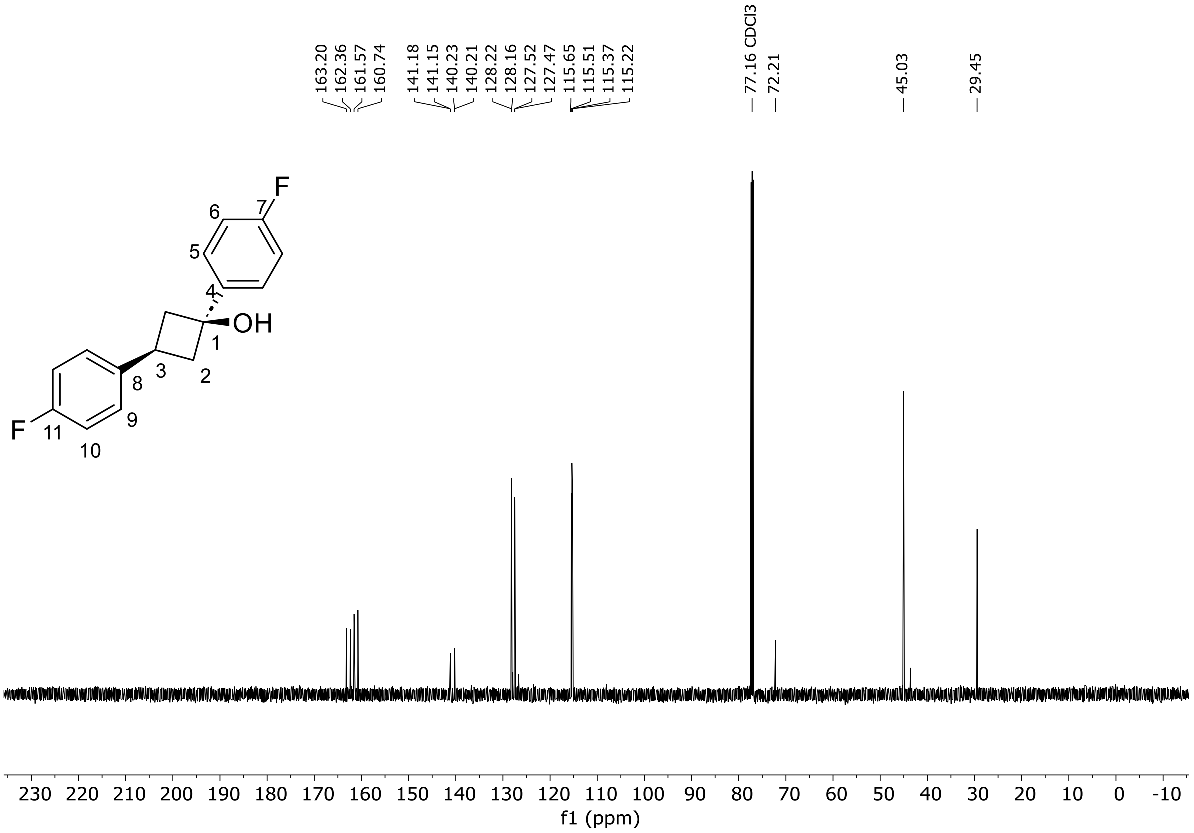


**Supplementary Figure 18.** $^{13}C\{^{1}H\}$ NMR of **1b** (126 MHz, 299 K, $CDCl_3$).

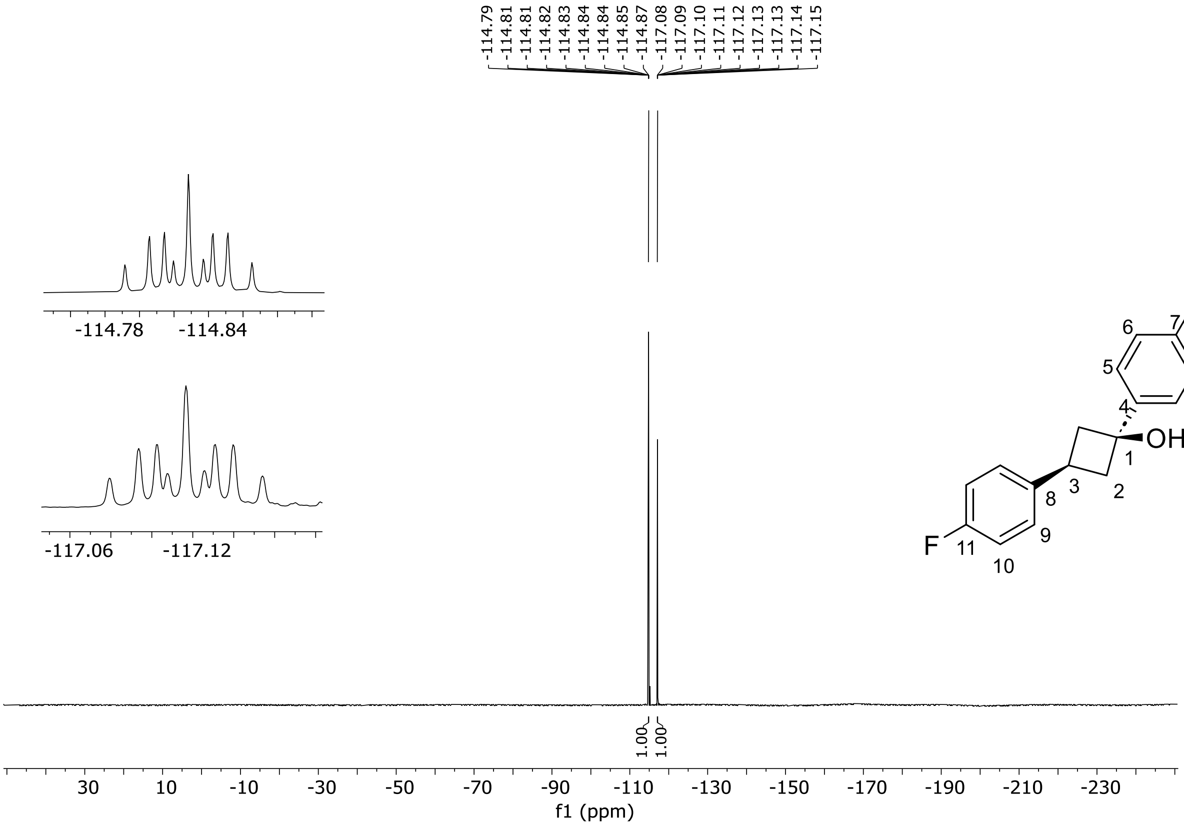


**Supplementary Figure 19.** $^{19}F$ NMR of **1b** (376 MHz, 299 K, $CDCl_3$).

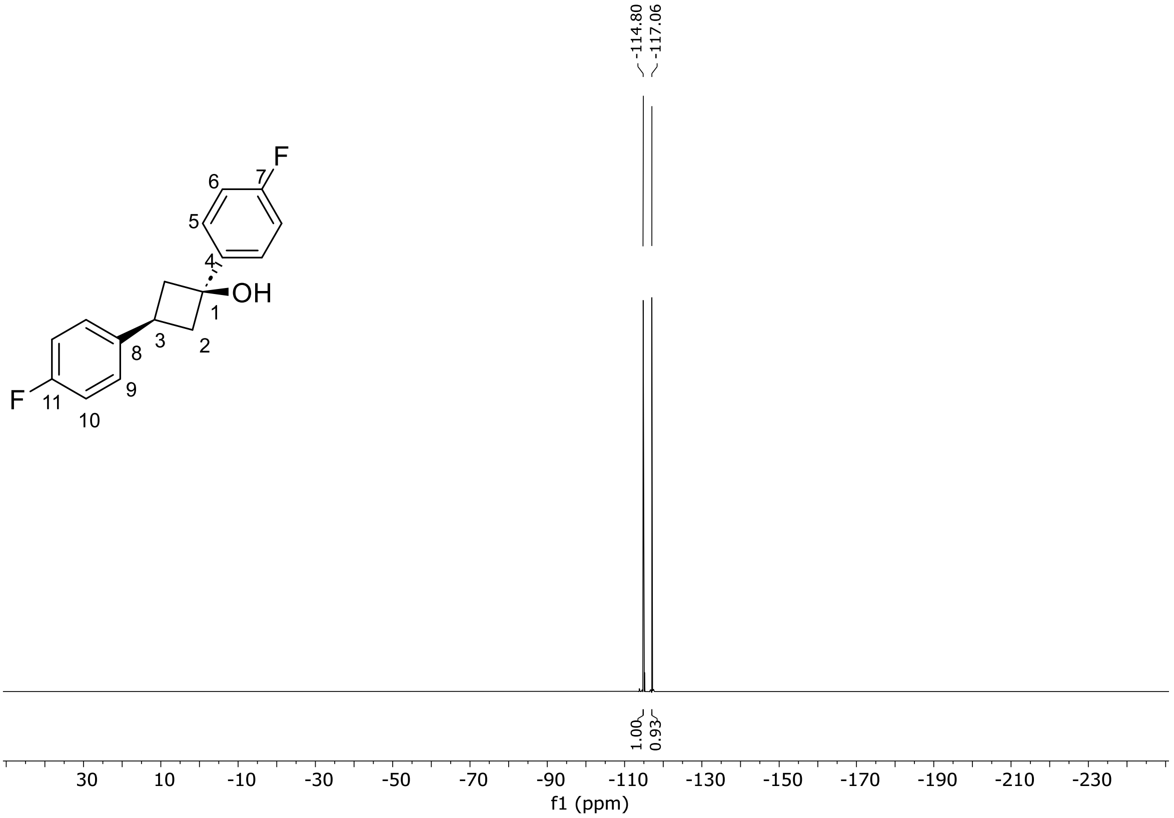


**Supplementary Figure 20.** $^{19}F\{^1H\}$ NMR of **1b** (376 MHz, 299 K, $CDCl_3$).

## 1,3-Bis(4-chlorophenyl)cyclobutan-1-ol (*major*-1c)

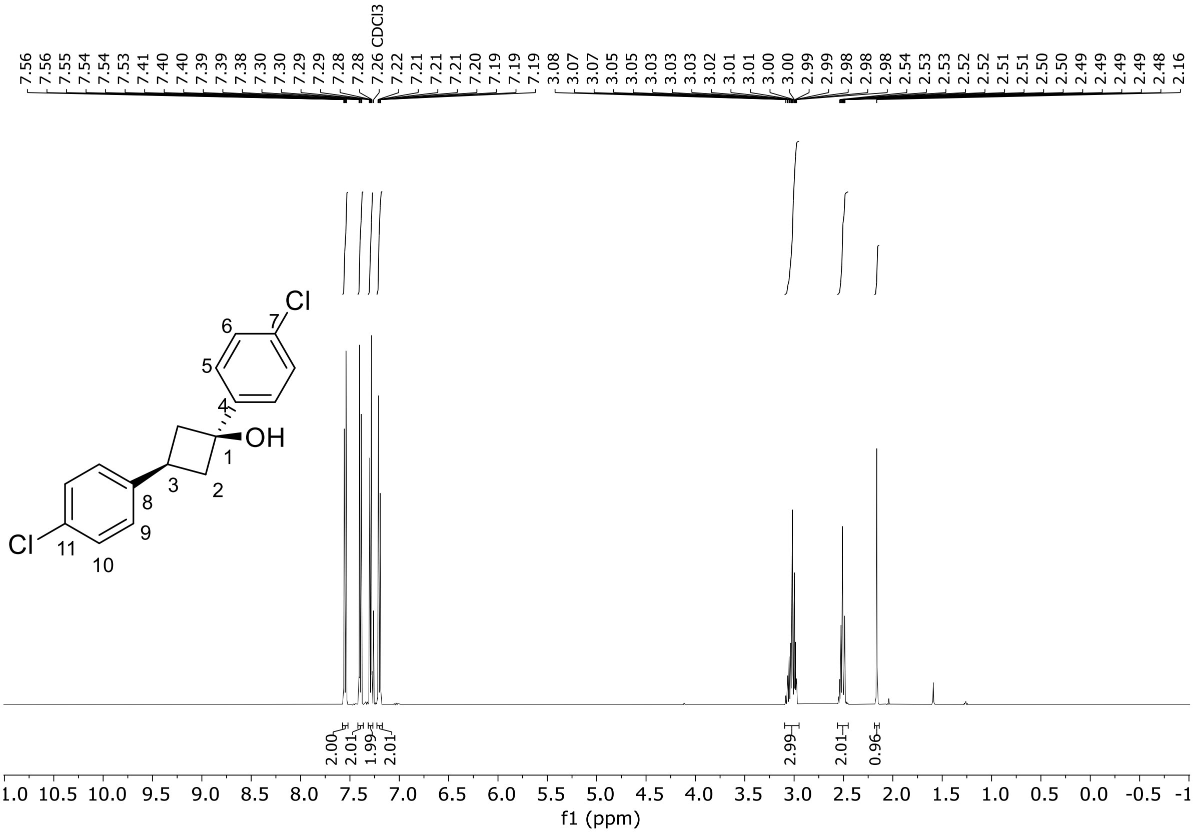


**Supplementary Figure 21.** $^{1}$H NMR of ***major*-1c** (500 MHz, 299 K, $CDCl_3$).

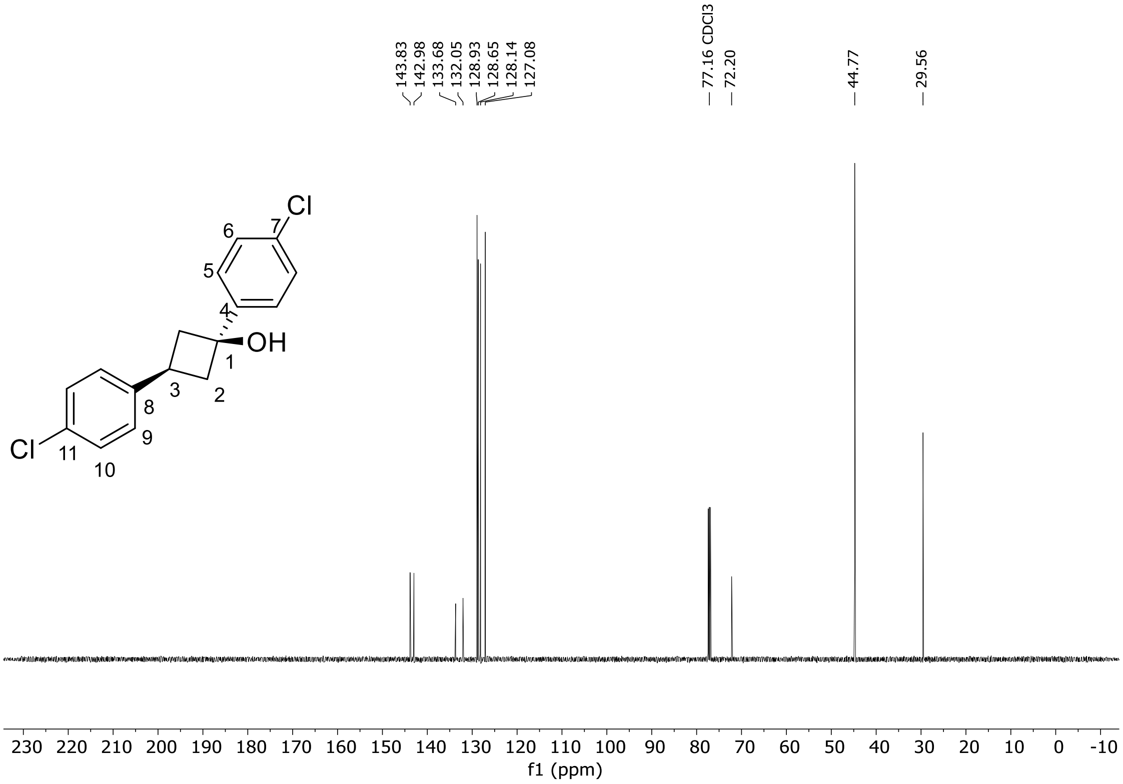


**Supplementary Figure 22.** $^{13}C\{^{1}H\}$ NMR of ***major*-1c** (126 MHz, 299 K, $CDCl_3$).

## 1,3-Bis(4-chlorophenyl)cyclobutan-1-ol (*minor*-1c)

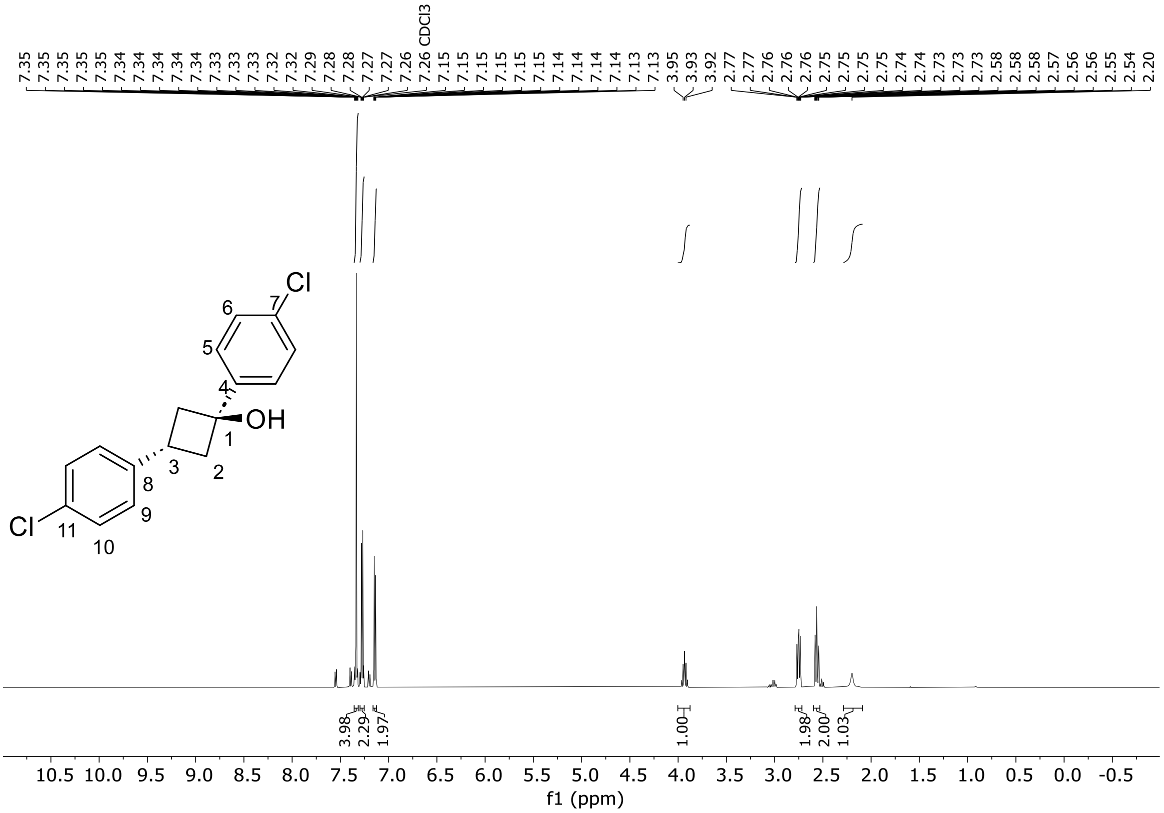


**Supplementary Figure 23.** $^{1}$H NMR of ***minor*-1c** (599 MHz, 299 K, $CDCl_3$).

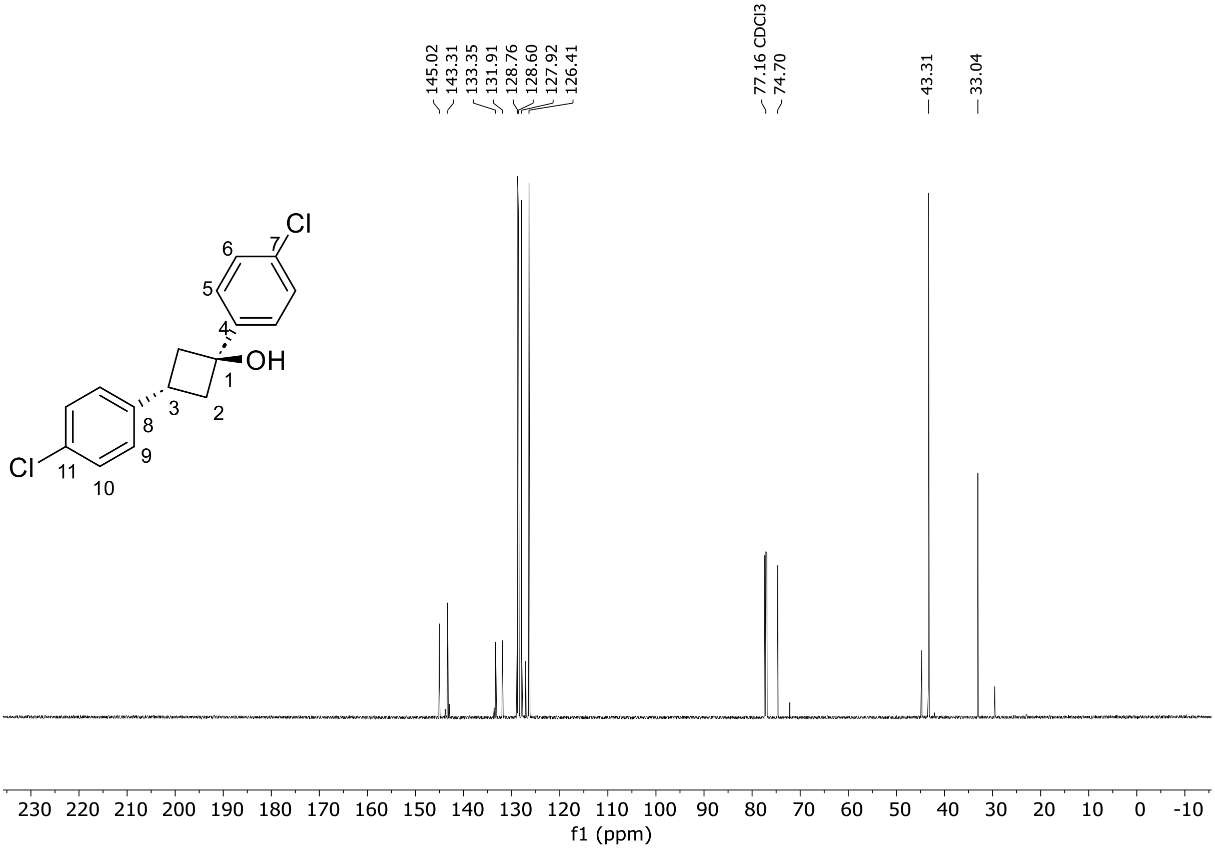


**Supplementary Figure 24.** $^{13}C\{^{1}H\}$ NMR of ***minor*-1c** (151 MHz, 299 K, $CDCl_3$).

**1,3-Bis(4-bromophenyl)cyclobutan-1-ol (1d)**

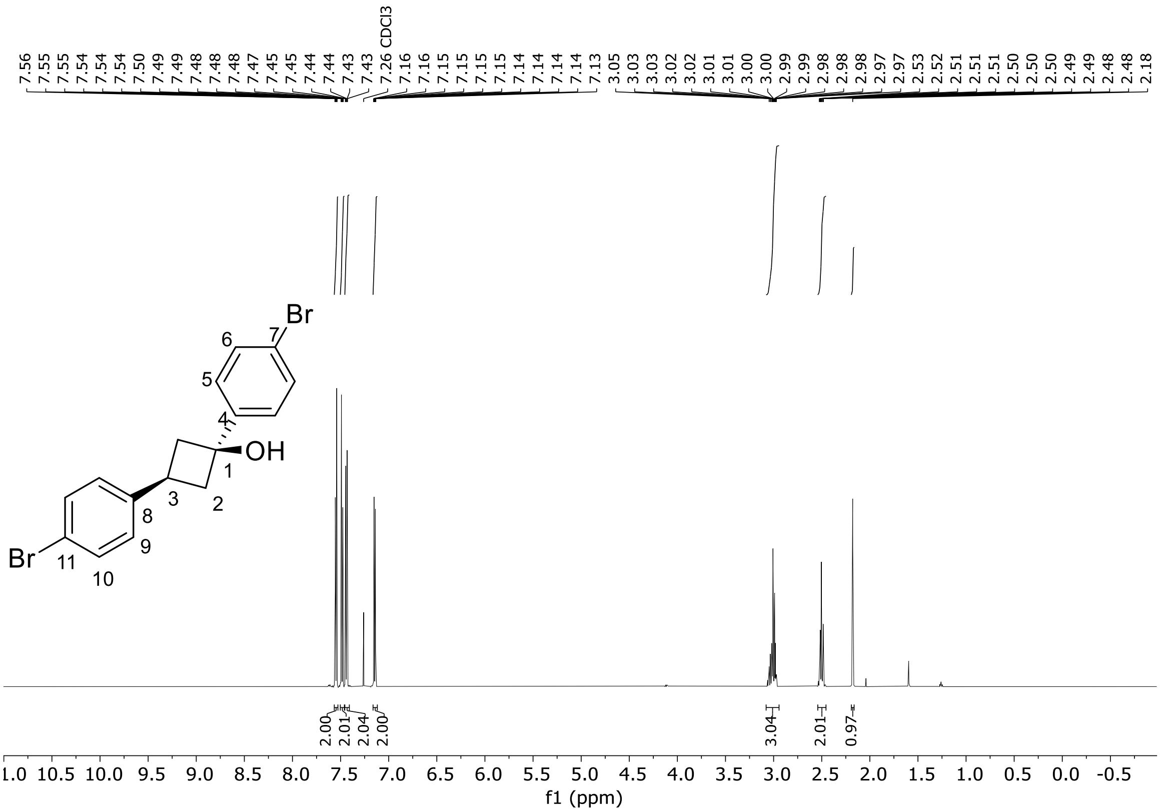

**Supplementary Figure 25.** $^{1}$H NMR of **1d** (599 MHz, 299 K, $CDCl_3$).

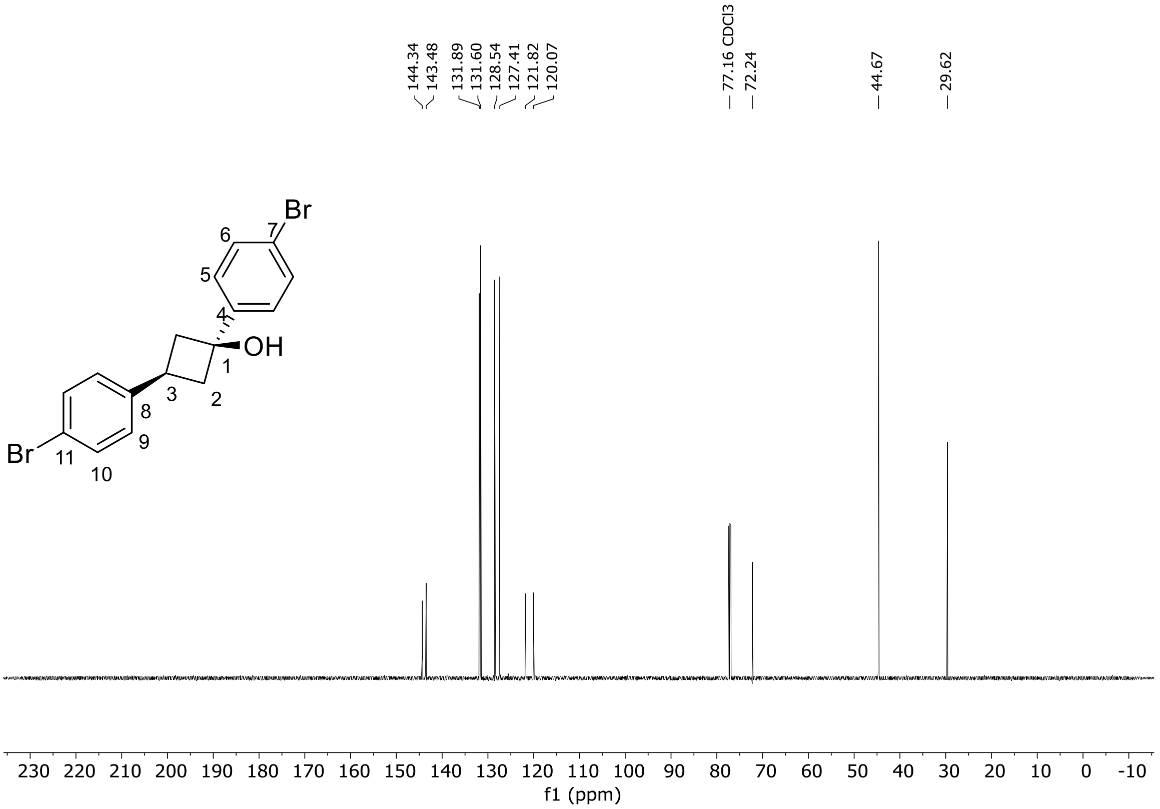

**Supplementary Figure 26.** $^{13}C\{^{1}H\}$ NMR of **1d** (151 MHz, 299 K, $CDCl_3$).

## 1,3-Bis(4-(trifluoromethyl)phenyl)cyclobutan-1-ol (1e)

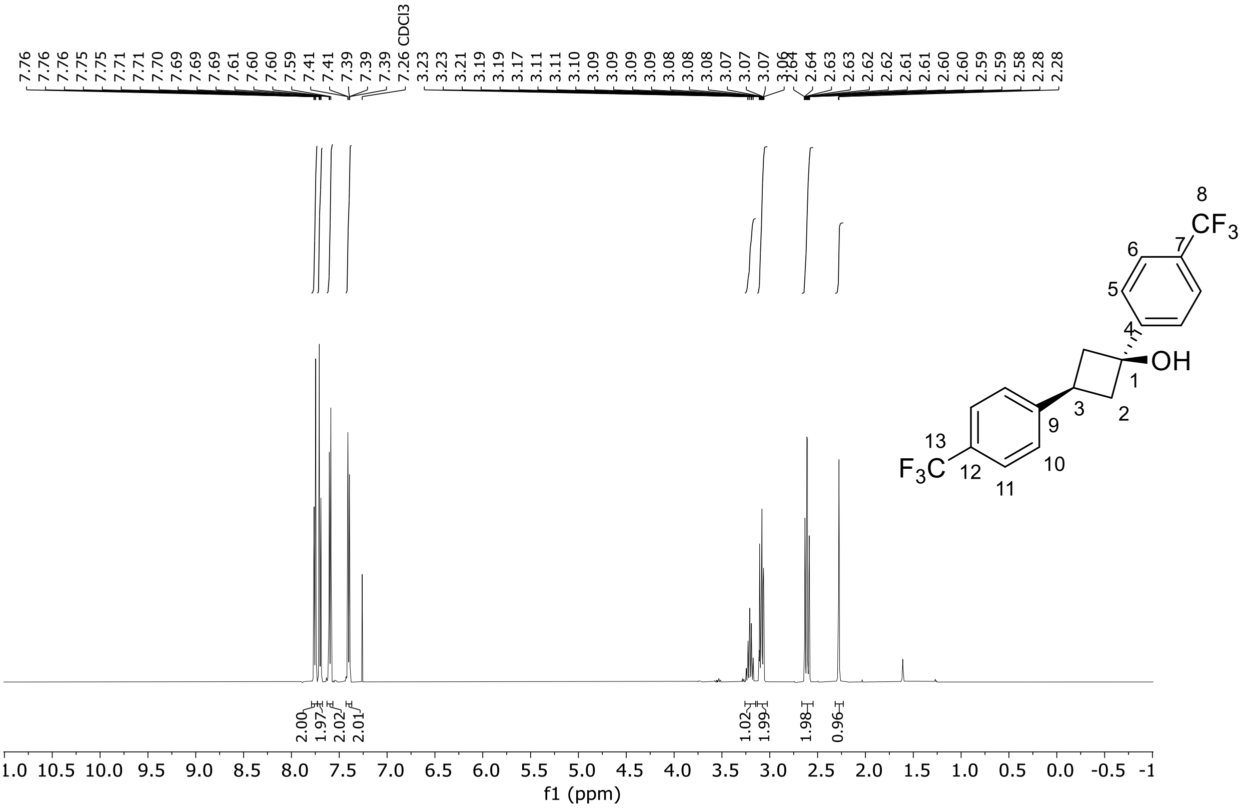

**Supplementary Figure 27.** $^{1}$H NMR of **1e** (500 MHz, 299 K, $CDCl_3$).

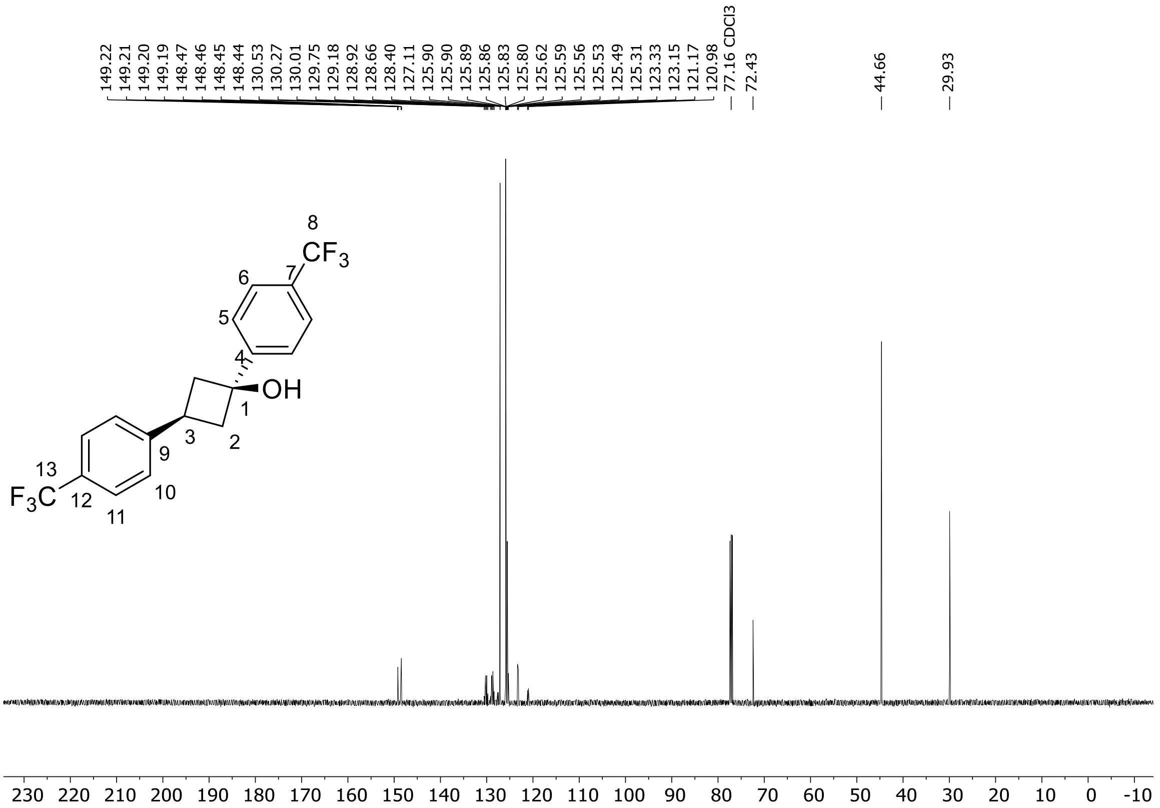

**Supplementary Figure 28.** $^{13}C\{^{1}H\}$ NMR of **1e** (126 MHz, 299 K, $CDCl_3$).

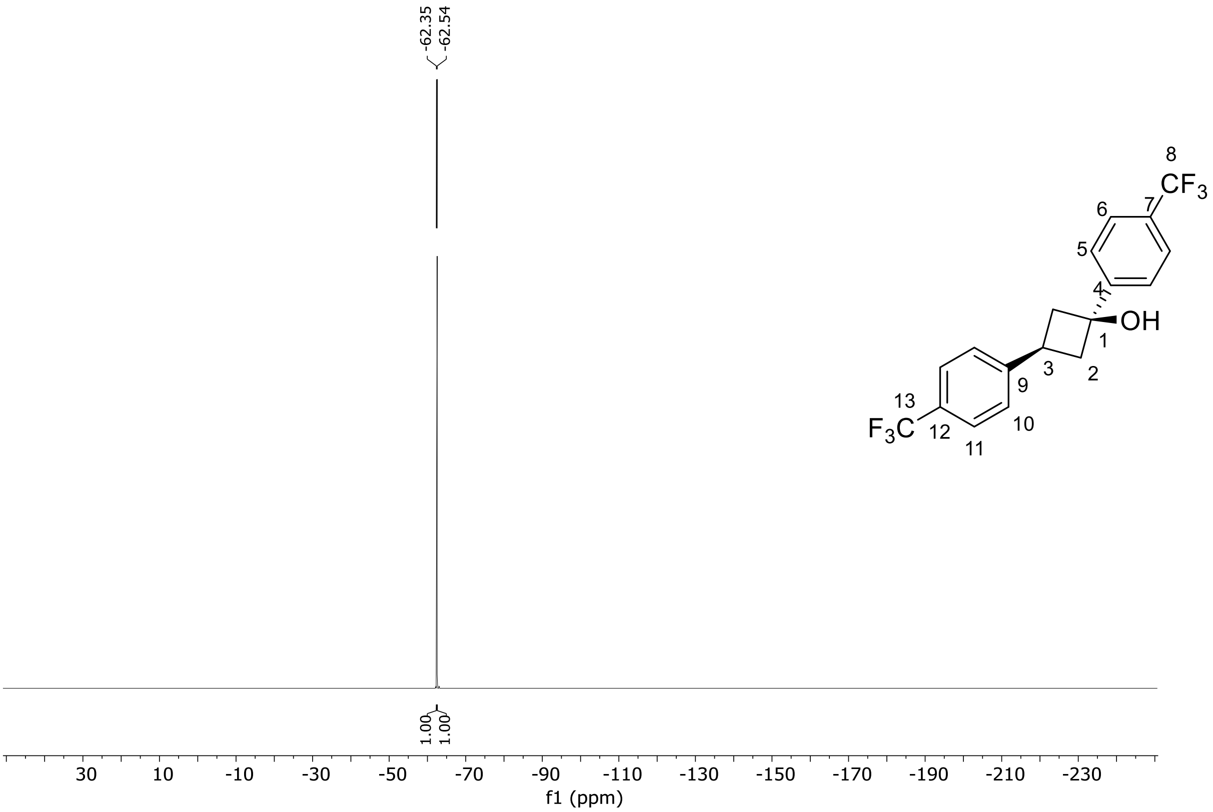


**Supplementary Figure 29.** $^{19}$F NMR of **1e** (376 MHz, 299 K, $CDCl_3$).

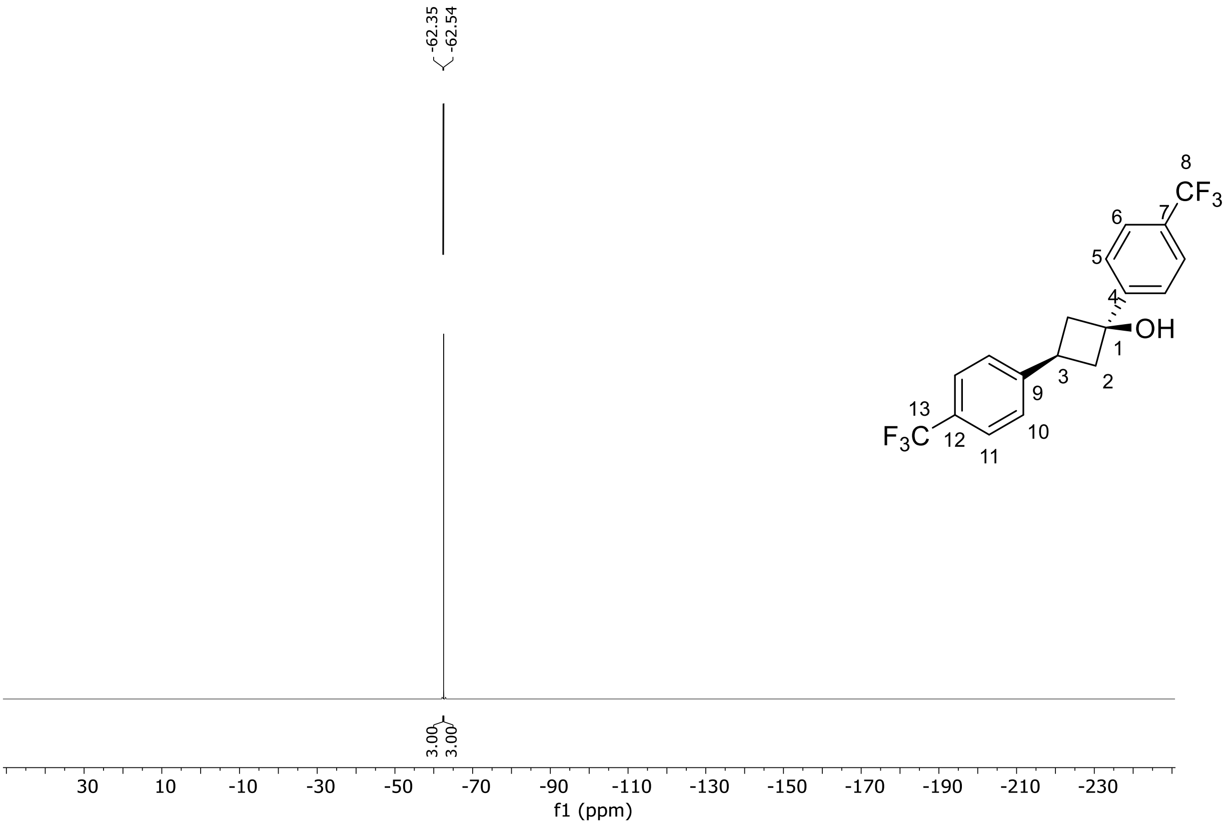


**Supplementary Figure 30.** $^{19}$F{$^{1}$H} NMR of **1e** (376 MHz, 299 K, $CDCl_3$).

## 1-(4-Fluorophenyl)-3-phenylcyclobutan-1-ol (1f)

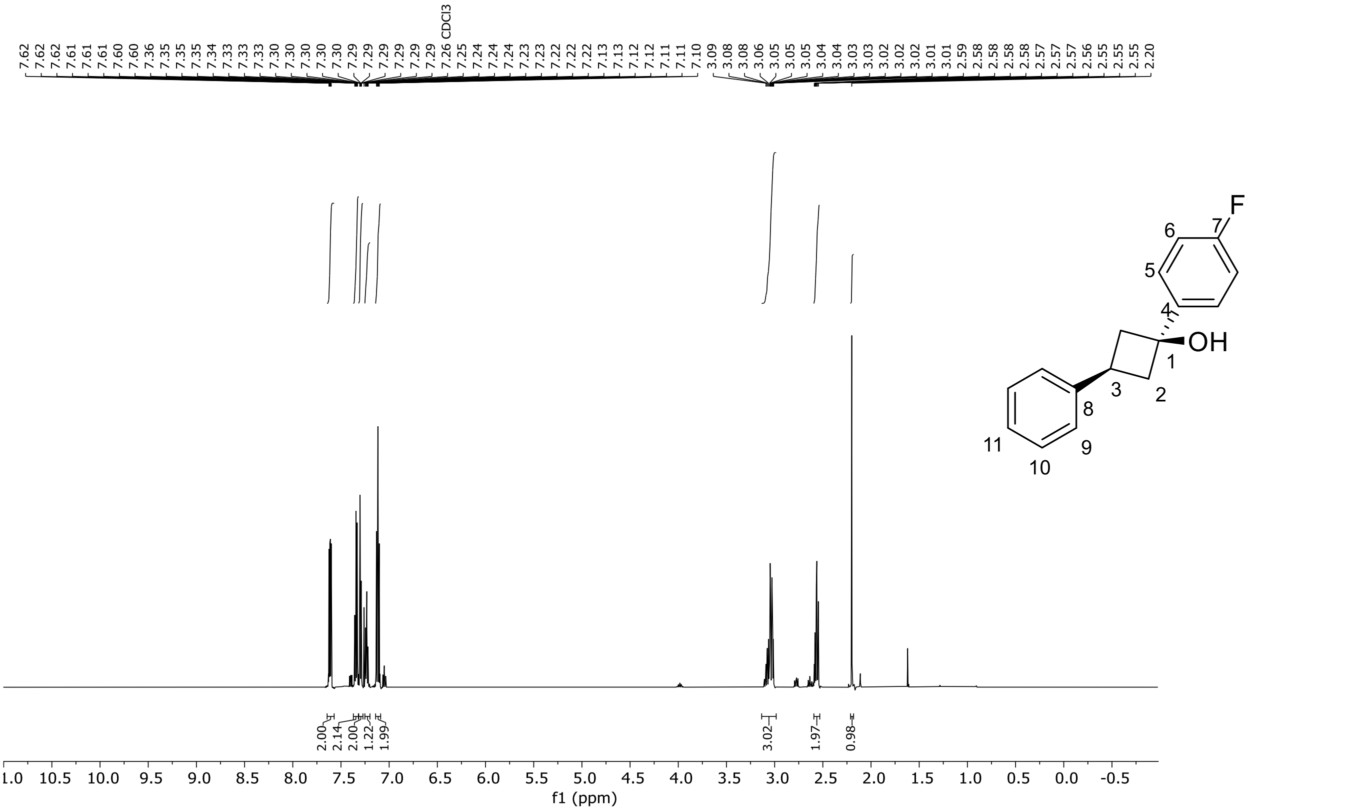

**Supplementary Figure 31.** $^{1}H$ NMR of **1f** (599 MHz, 299 K, $CDCl_3$).

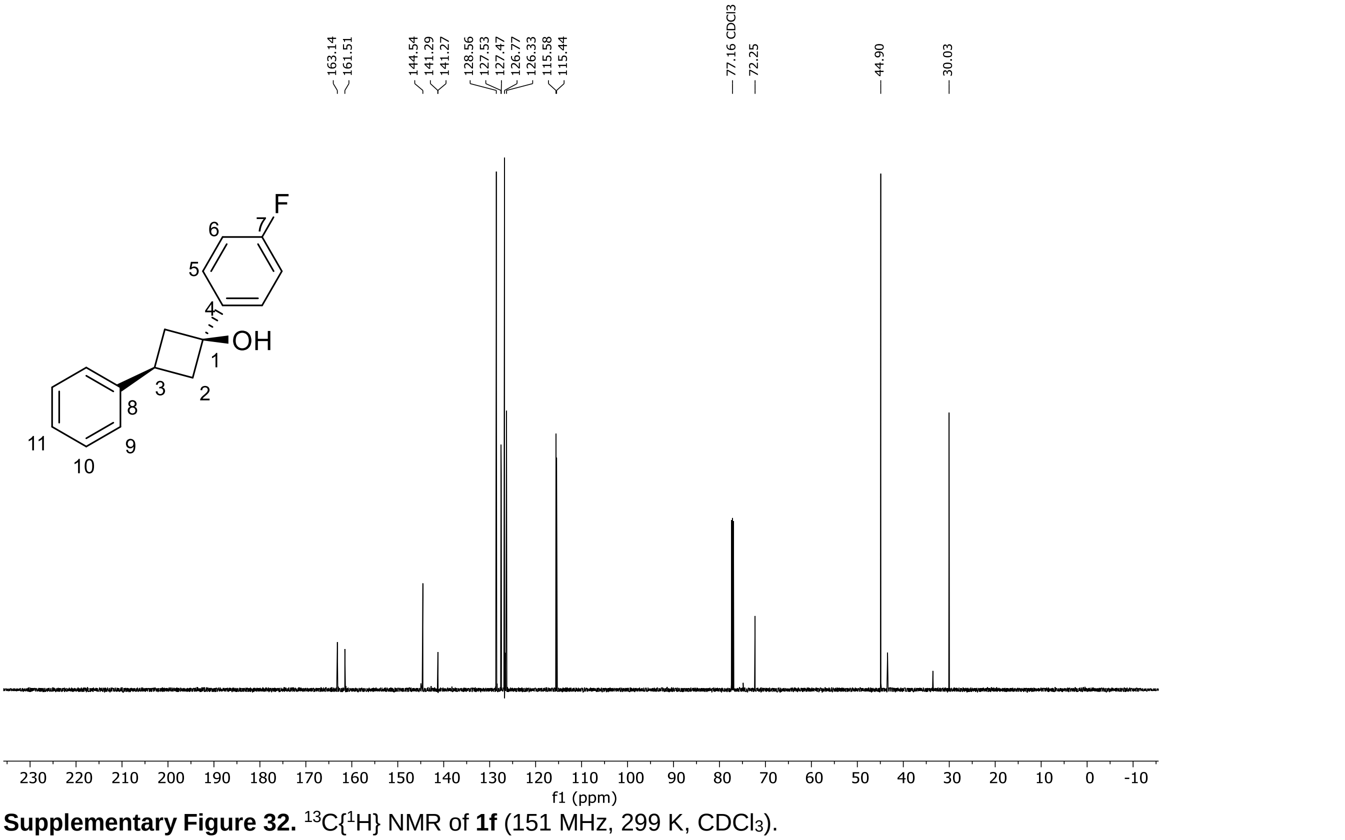

**Supplementary Figure 32.** $^{13}C\{^{1}H\}$ NMR of **1f** (151 MHz, 299 K, $CDCl_3$).

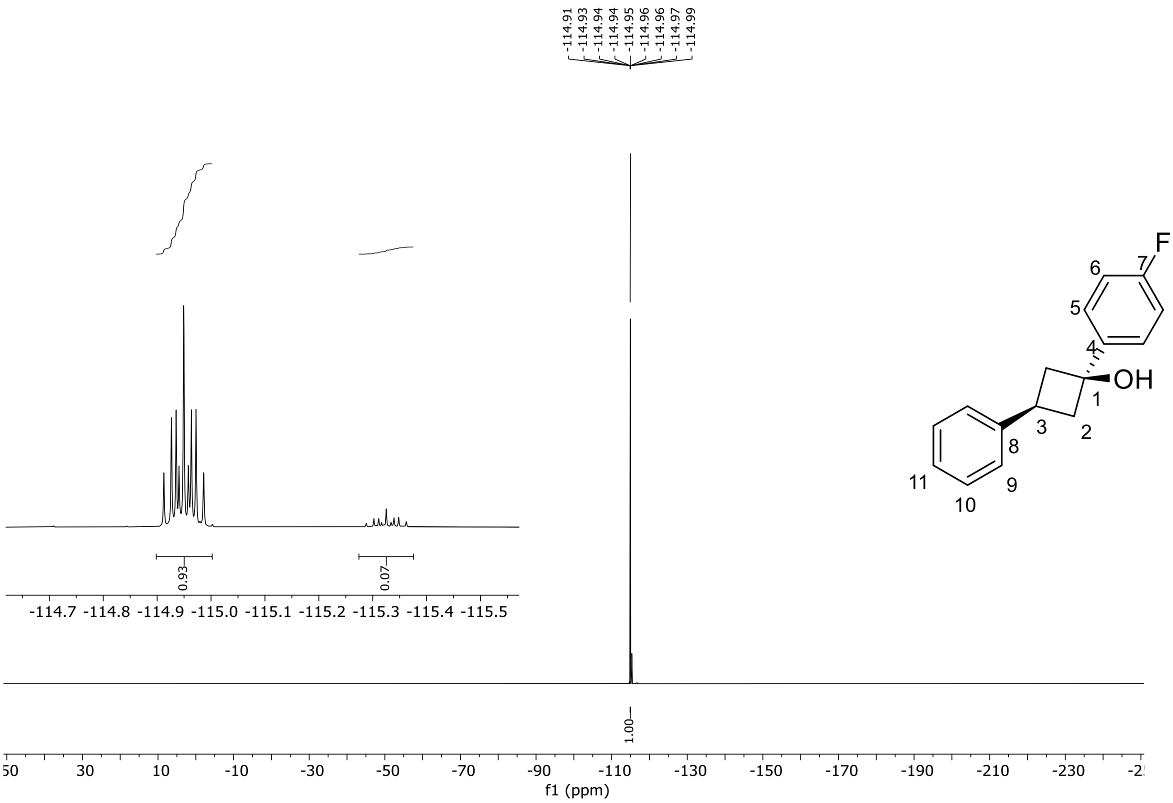


**Supplementary Figure 33.** $^{19}$F NMR of **1f** (376 MHz, 299 K, $CDCl_3$).

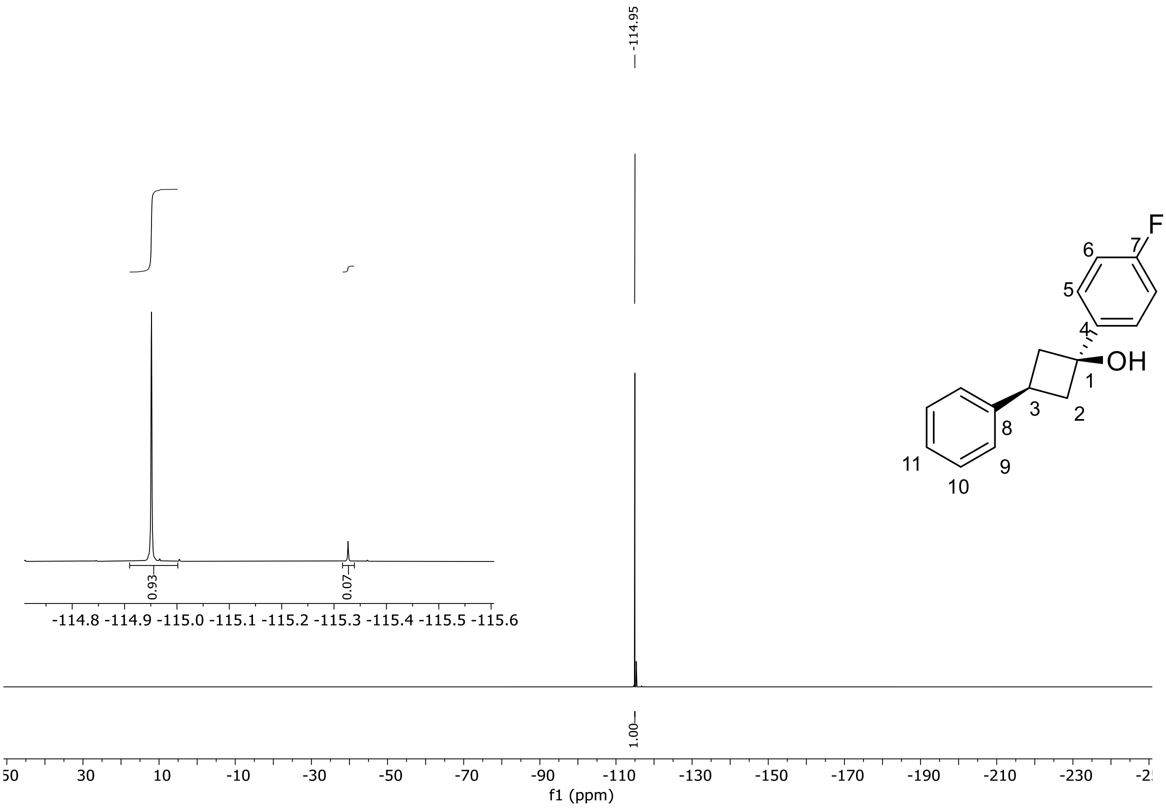


**Supplementary Figure 34.** $^{19}$F{$^1$H} NMR of **1f** (376 MHz, 299 K, $CDCl_3$).

**3-Phenyl-1-(4-chlorophenyl)cyclobutan-1-ol (1g)**

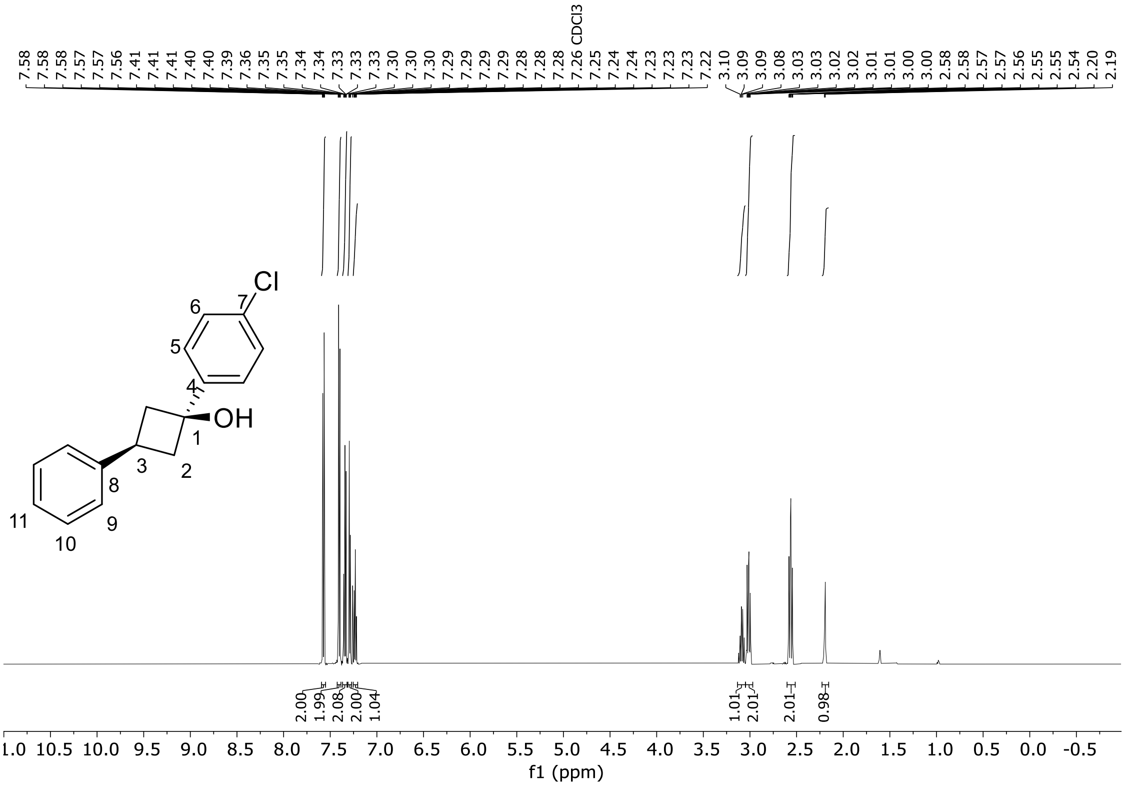


**Supplementary Figure 35.** $^{1}H$ NMR of **1g** (599 MHz, 299 K, $CDCl_3$).

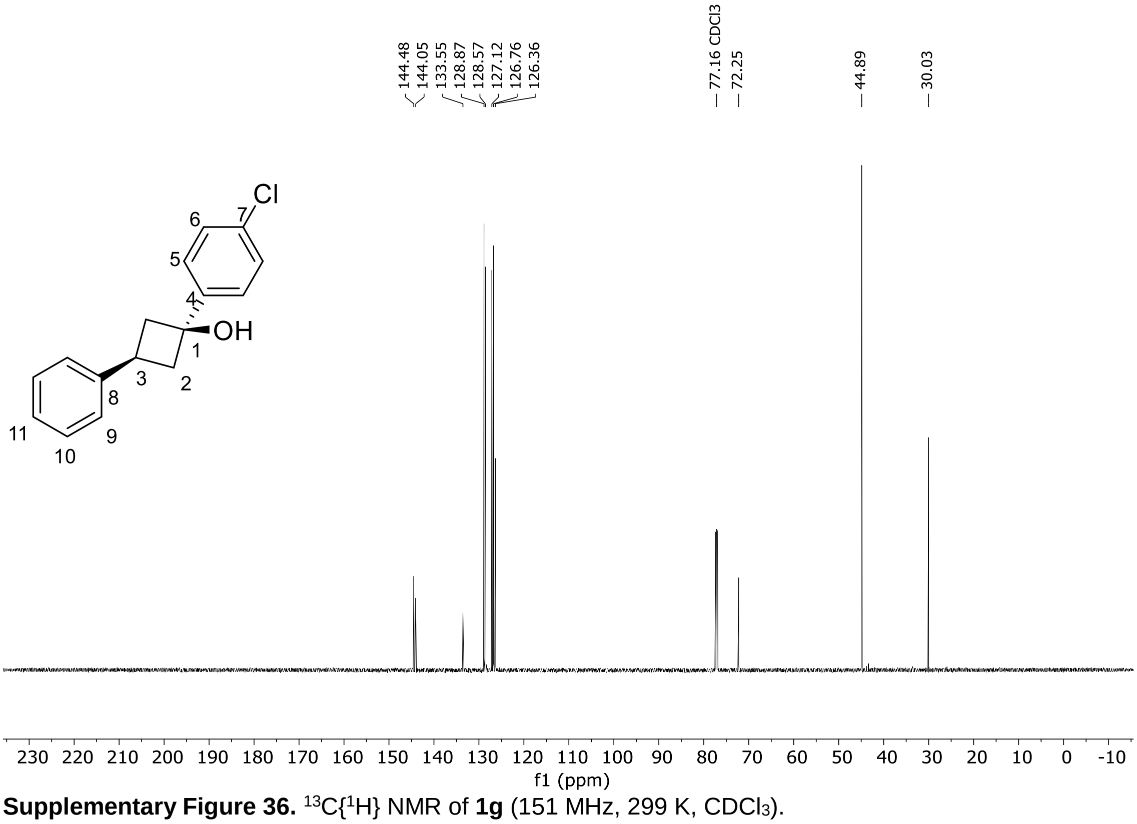


**Supplementary Figure 36.** $^{13}C\{^{1}H\}$ NMR of **1g** (151 MHz, 299 K, $CDCl_3$).

**3-Phenyl-1-(4-bromophenyl)cyclobutan-1-ol (1h)**

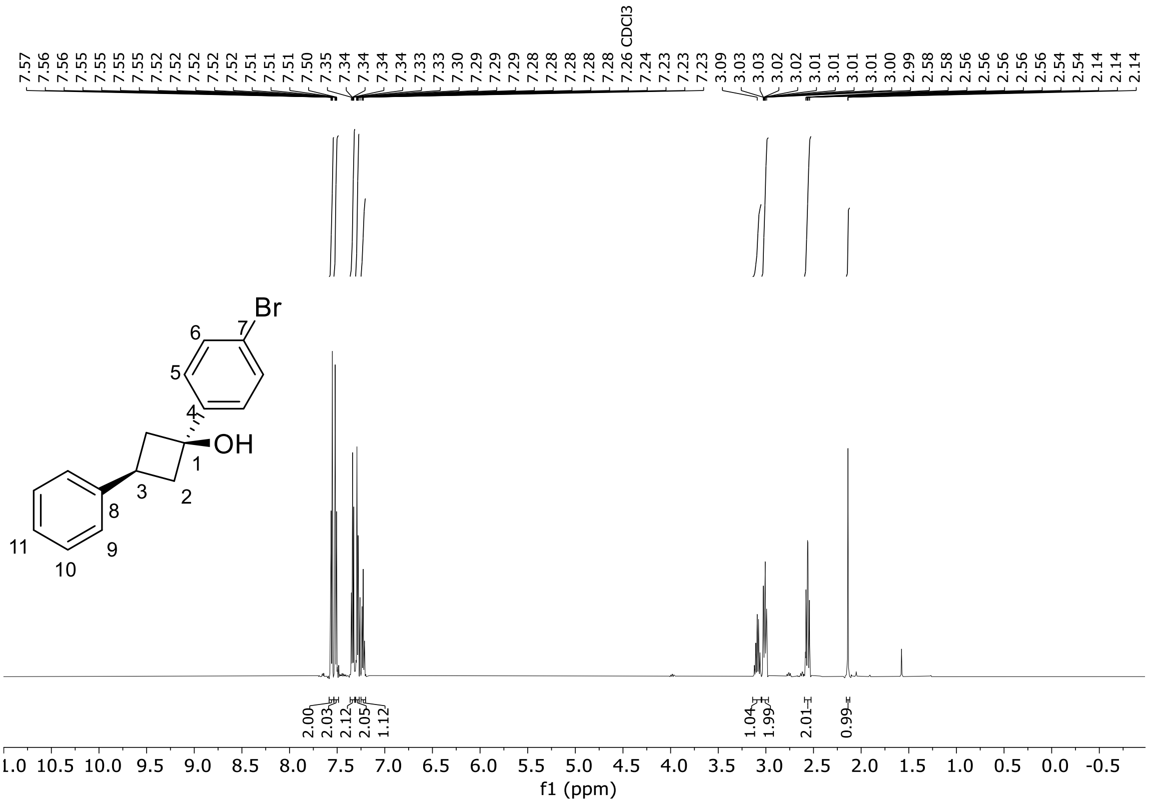

**Supplementary Figure 37.** $^{1}H$ NMR of **1h** (599 MHz, 299 K, $CDCl_3$).

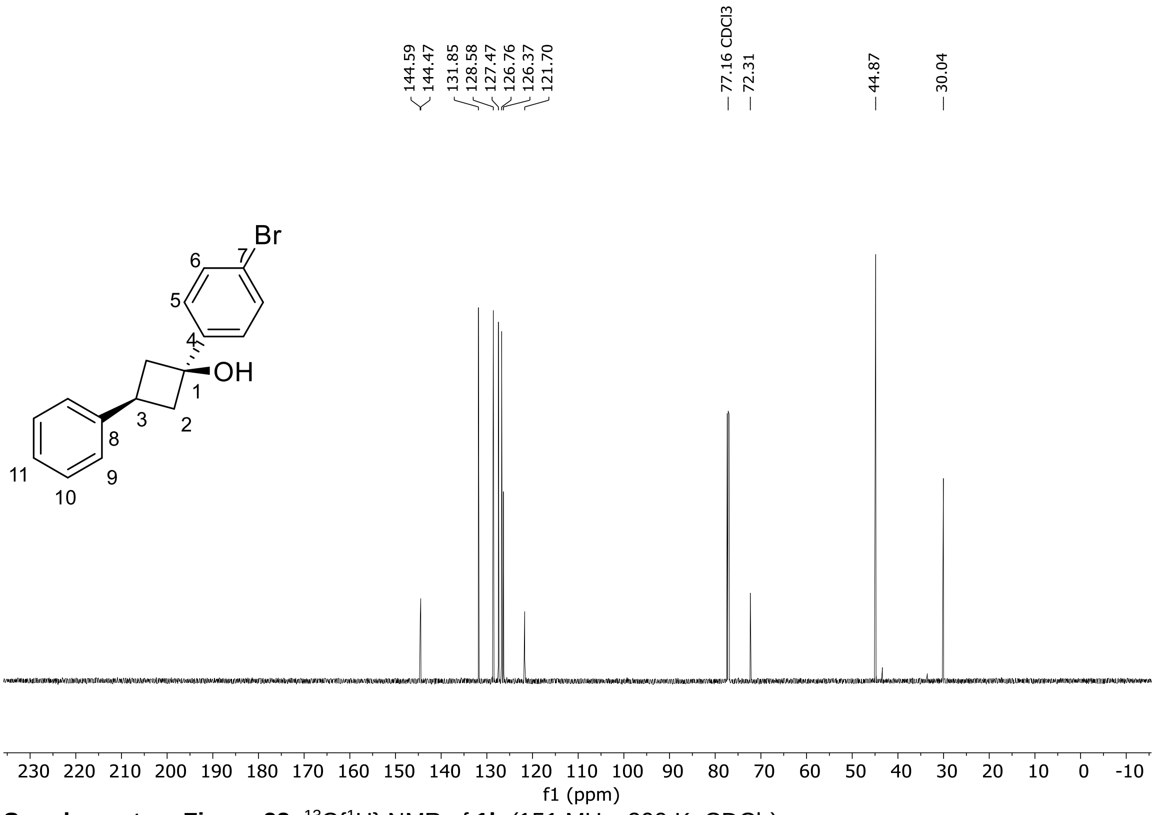

**Supplementary Figure 38.** $^{13}C\{^{1}H\}$ NMR of **1h** (151 MHz, 299 K, $CDCl_3$).

**3-Phenyl-1-(4-(trifluoromethoxy)phenyl)cyclobutan-1-ol (1i)**

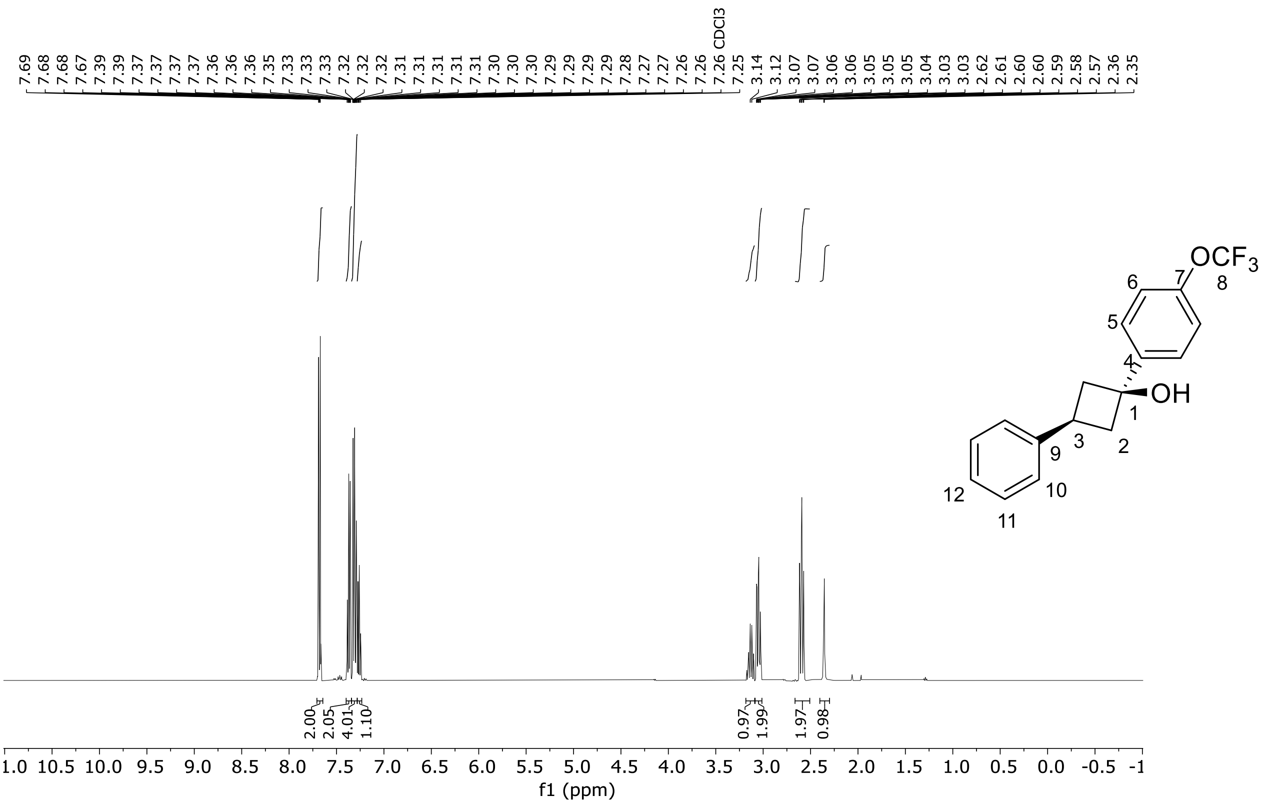

**Supplementary Figure 39.** $^{1}H$ NMR of **1i** (500 MHz, 299 K, $CDCl_3$).

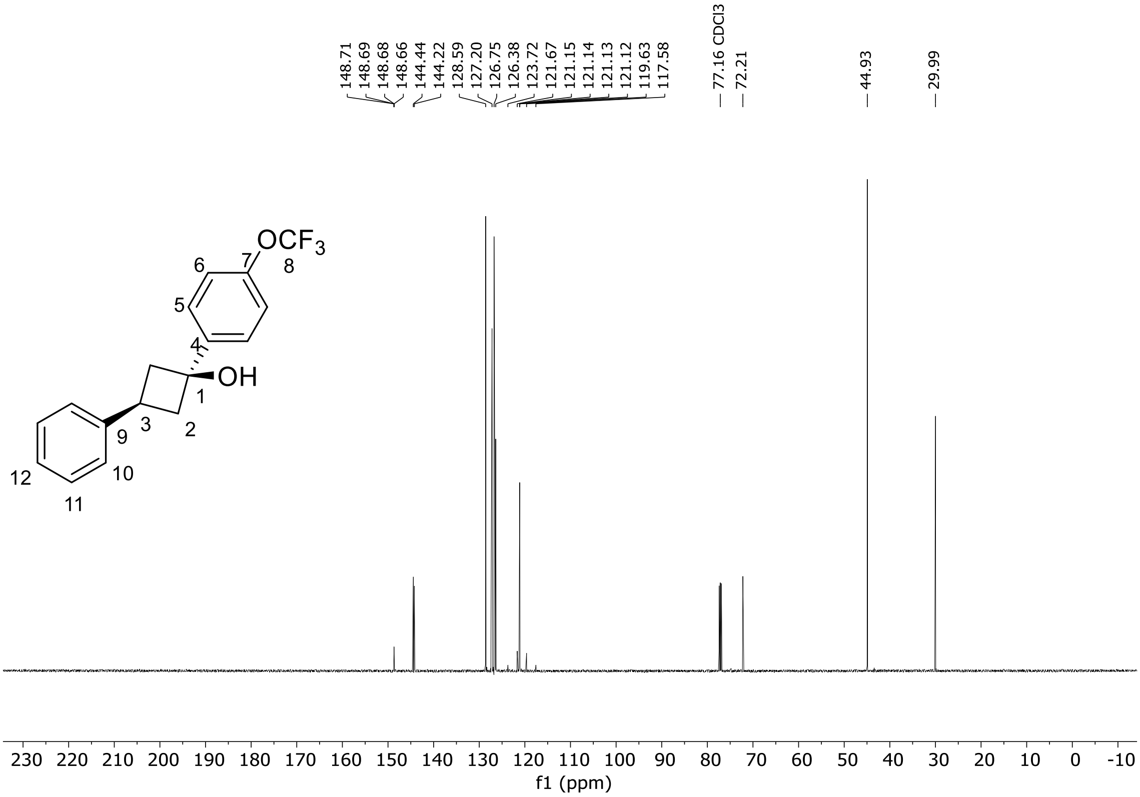

**Supplementary Figure 40.** $^{13}C\{^{1}H\}$ NMR of **1i** (126 MHz, 299 K, $CDCl_3$).

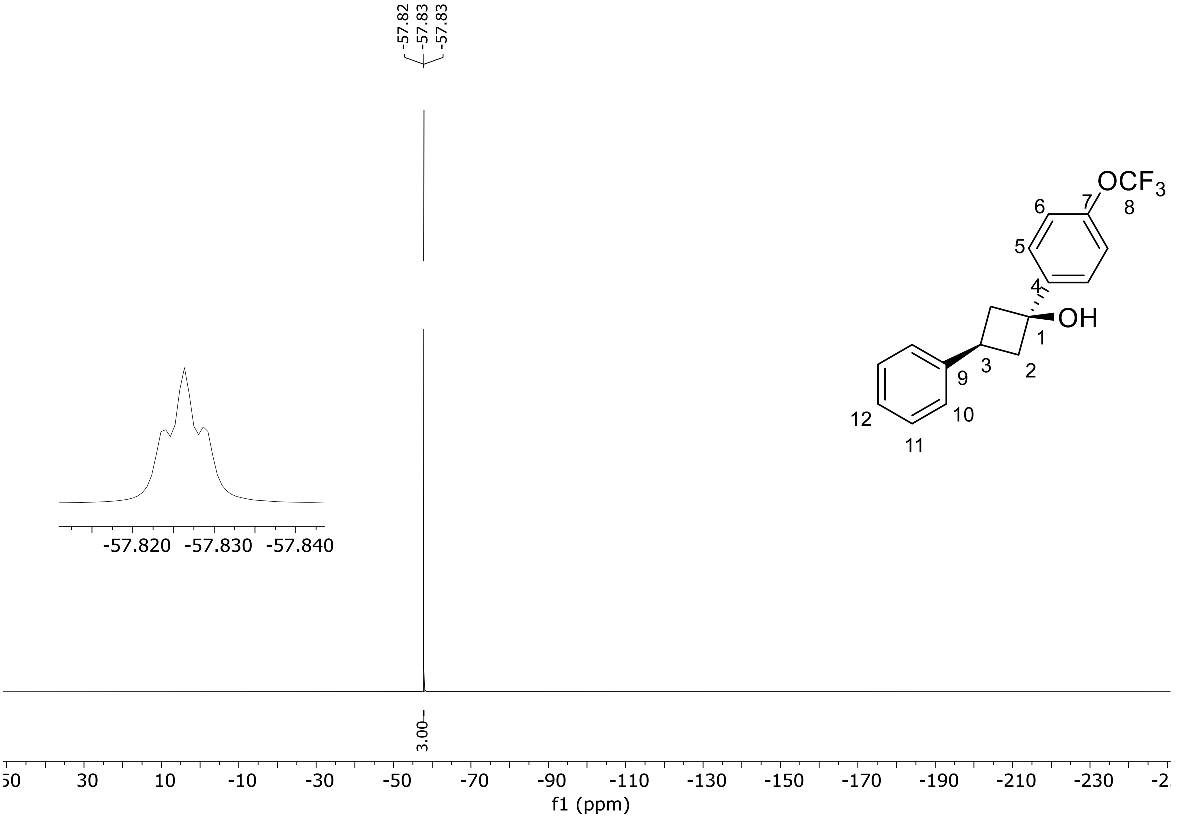


**Supplementary Figure 41.** $^{19}$F NMR of **1i** (376 MHz, 299 K, $CDCl_3$).

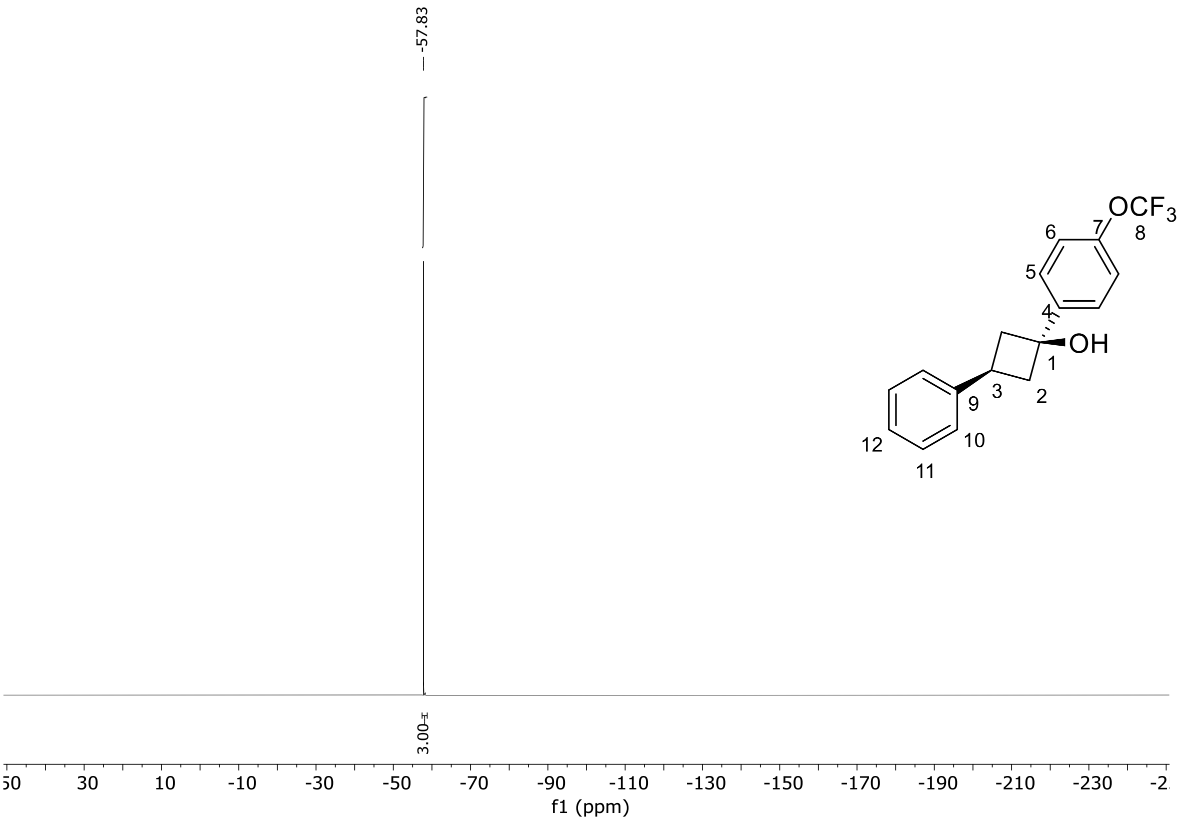


**Supplementary Figure 42.** $^{19}$F{$^1$H} NMR of **1i** (376 MHz, 299 K, $CDCl_3$).

**3-Phenyl-1-(4-(trifluoromethyl)phenyl)cyclobutan-1-ol (1j)**

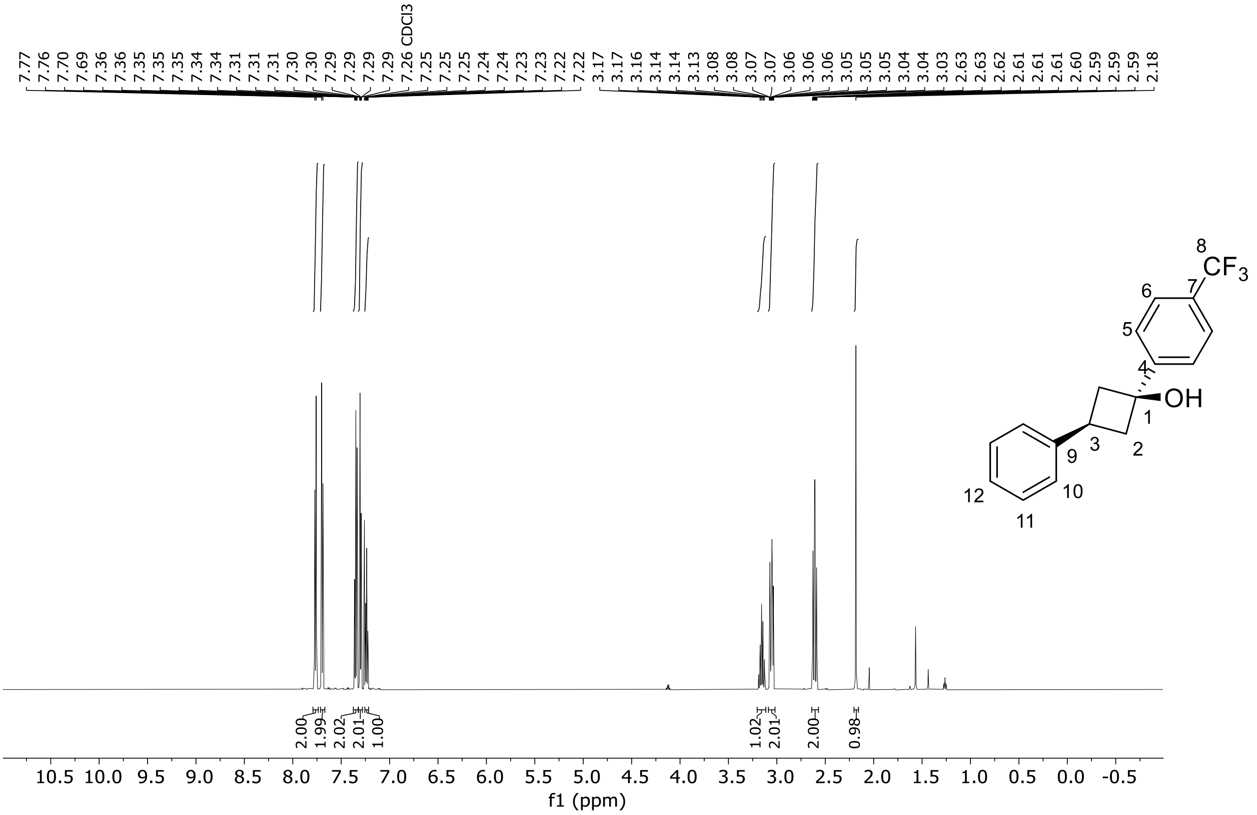


**Supplementary Figure 43.** $^{1}H$ NMR of **1j** (599 MHz, 299 K, $CDCl_3$).

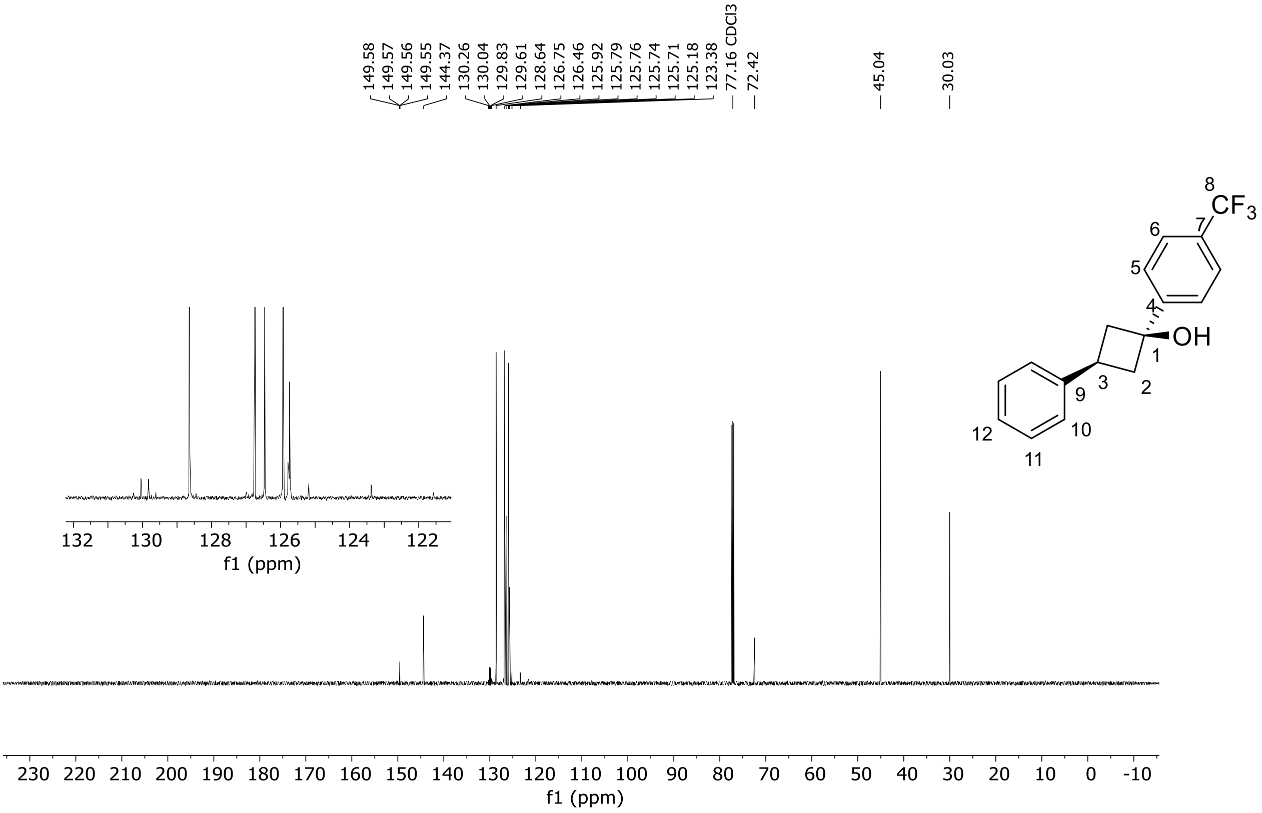


**Supplementary Figure 44.** $^{13}C\{^{1}H\}$ NMR of **1j** (151 MHz, 299 K, $CDCl_3$).

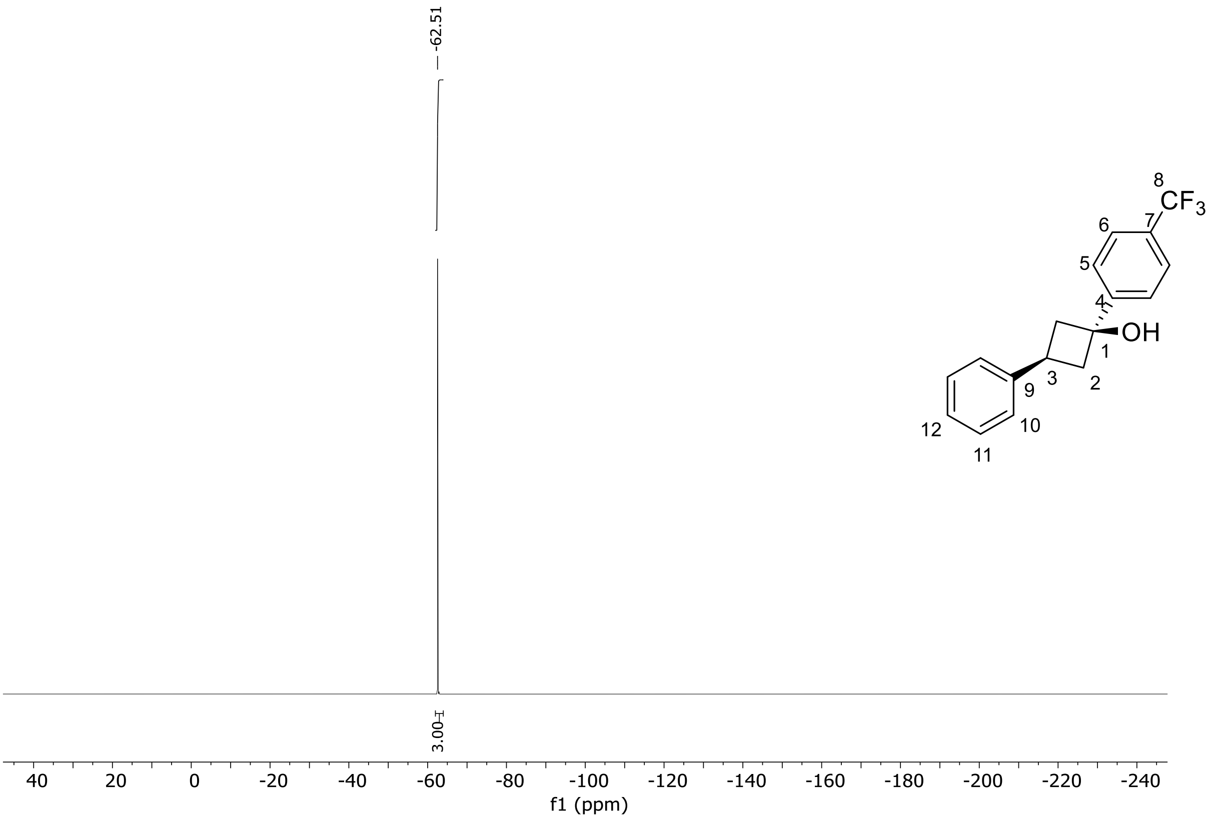


**Supplementary Figure 45.** $^{19}F$ NMR of **1j** (564 MHz, 299 K, $CDCl_3$).

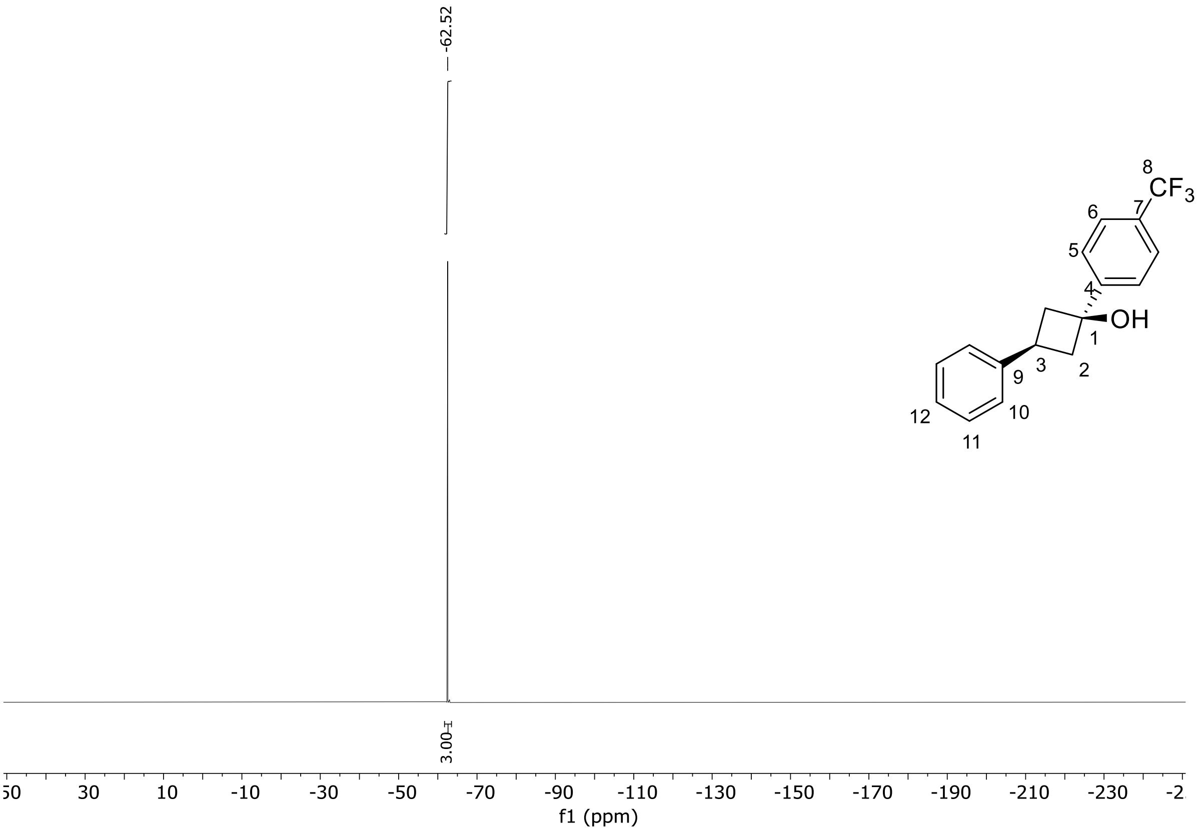


**Supplementary Figure 46.** $^{19}F\{^1H\}$ NMR of **1j** (376 MHz, 299 K, $CDCl_3$).

**4-(1-Hydroxy-3-phenylcyclobutyl)benzonitrile (1k)**

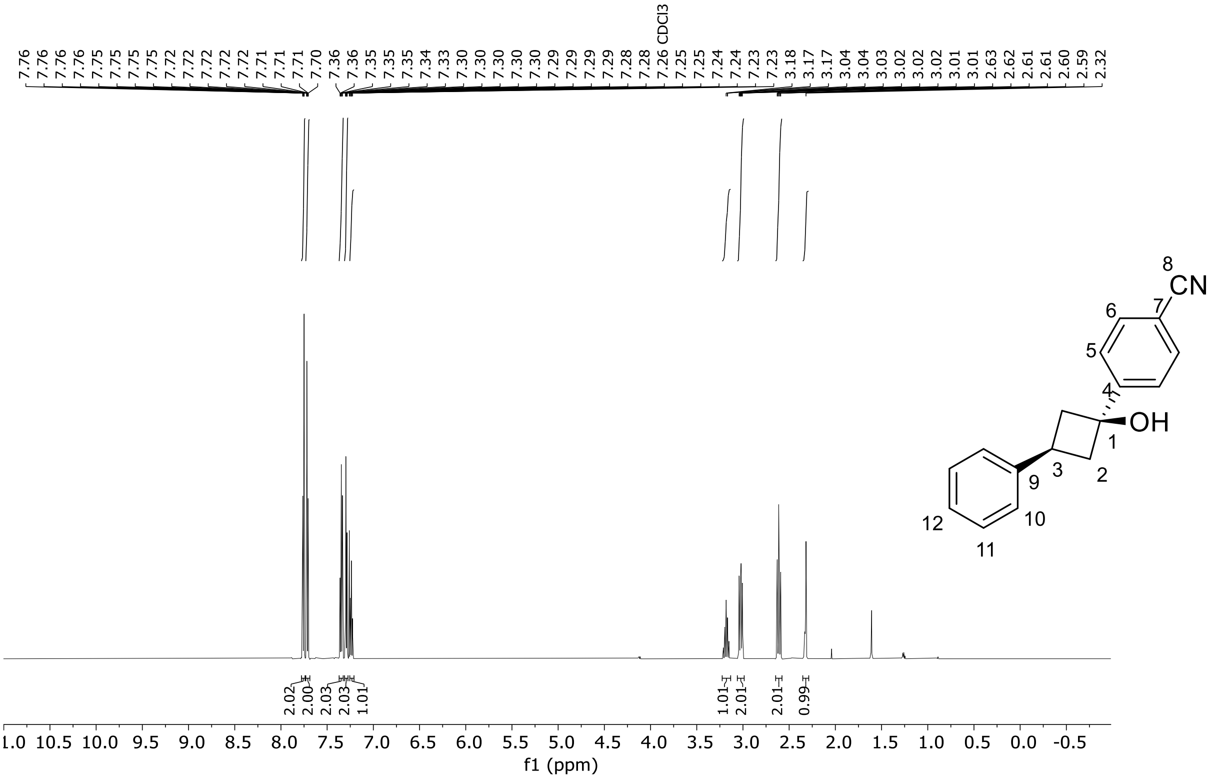


**Supplementary Figure 47.** $^{1}H$ NMR of **1k** (599 MHz, 299 K, $CDCl_3$).

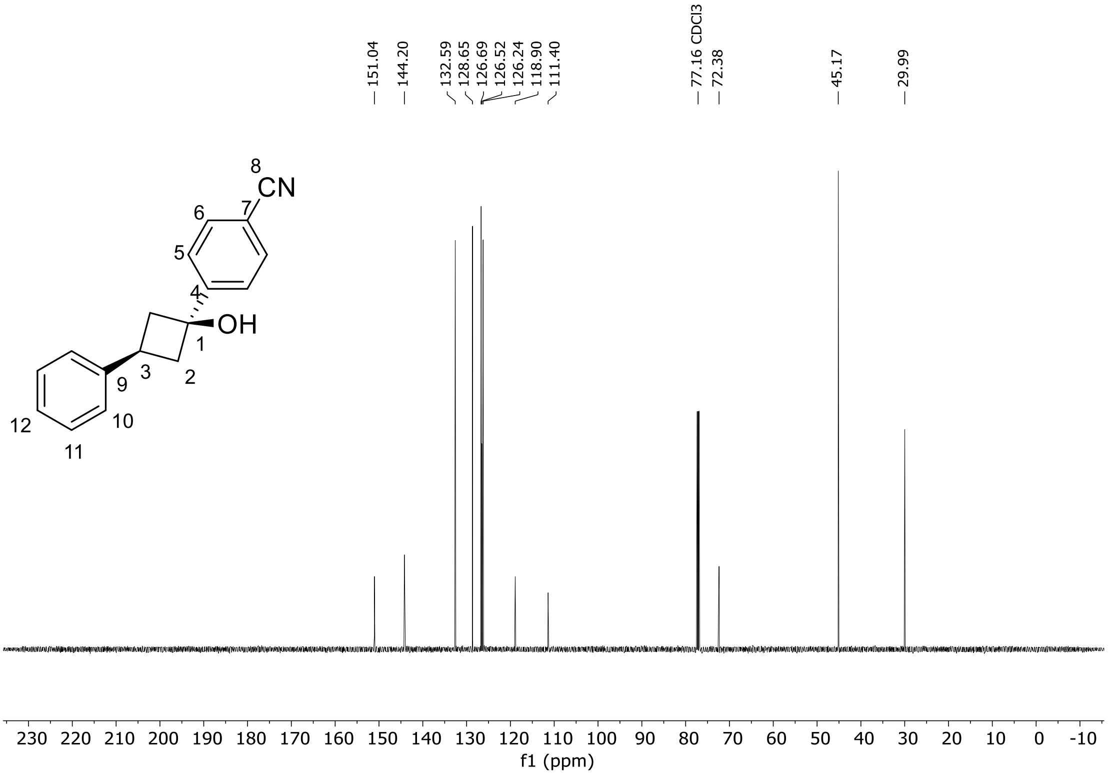


**Supplementary Figure 48.** $^{13}C\{^{1}H\}$ NMR of **1k** (151 MHz, 299 K, $CDCl_3$).

## 3-Phenyl-1-(4'-(trifluoromethyl)-[1,1'-biphenyl]-4-yl)cyclobutan-1-ol (1l)

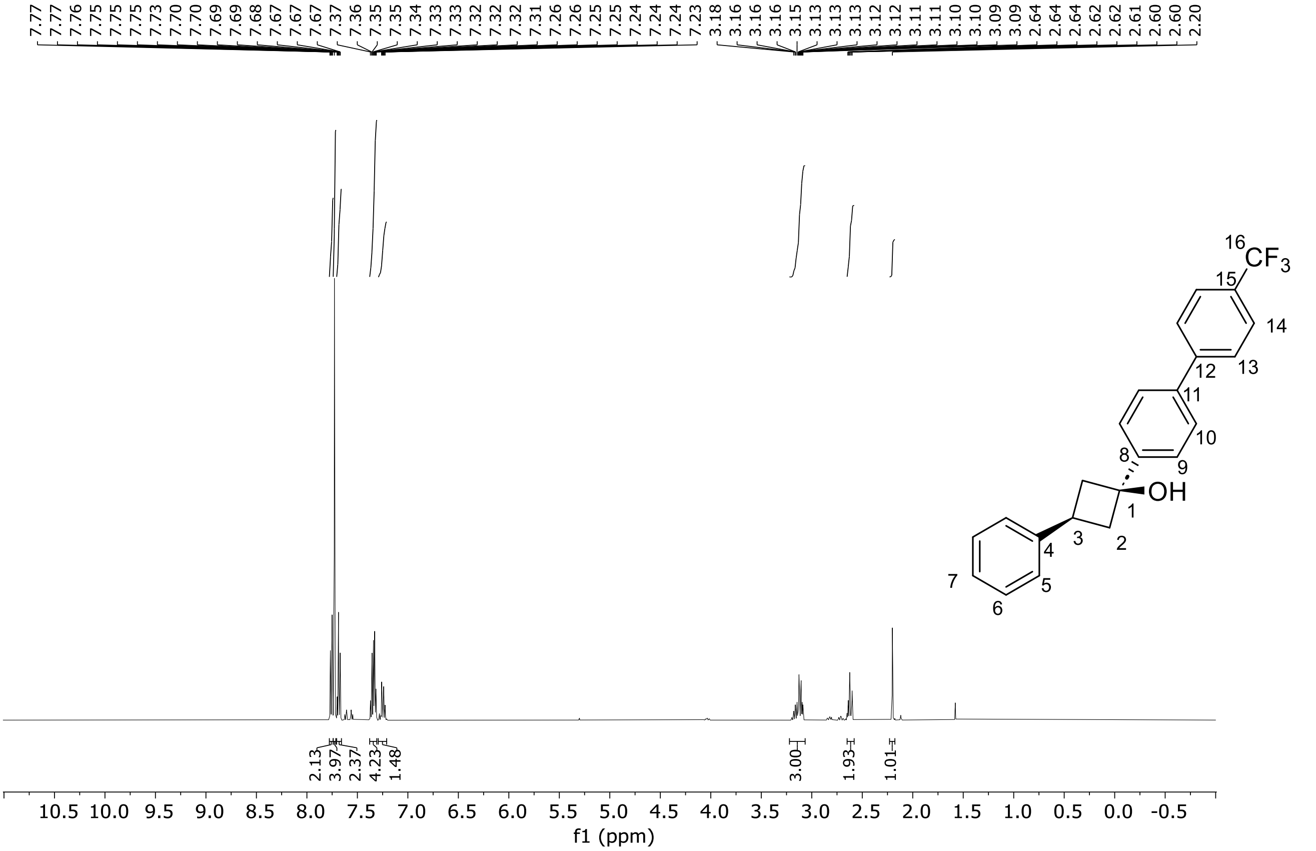


**Supplementary Figure 49.** $^1$H NMR of **1l** (500 MHz, 299 K, $CDCl_3$).

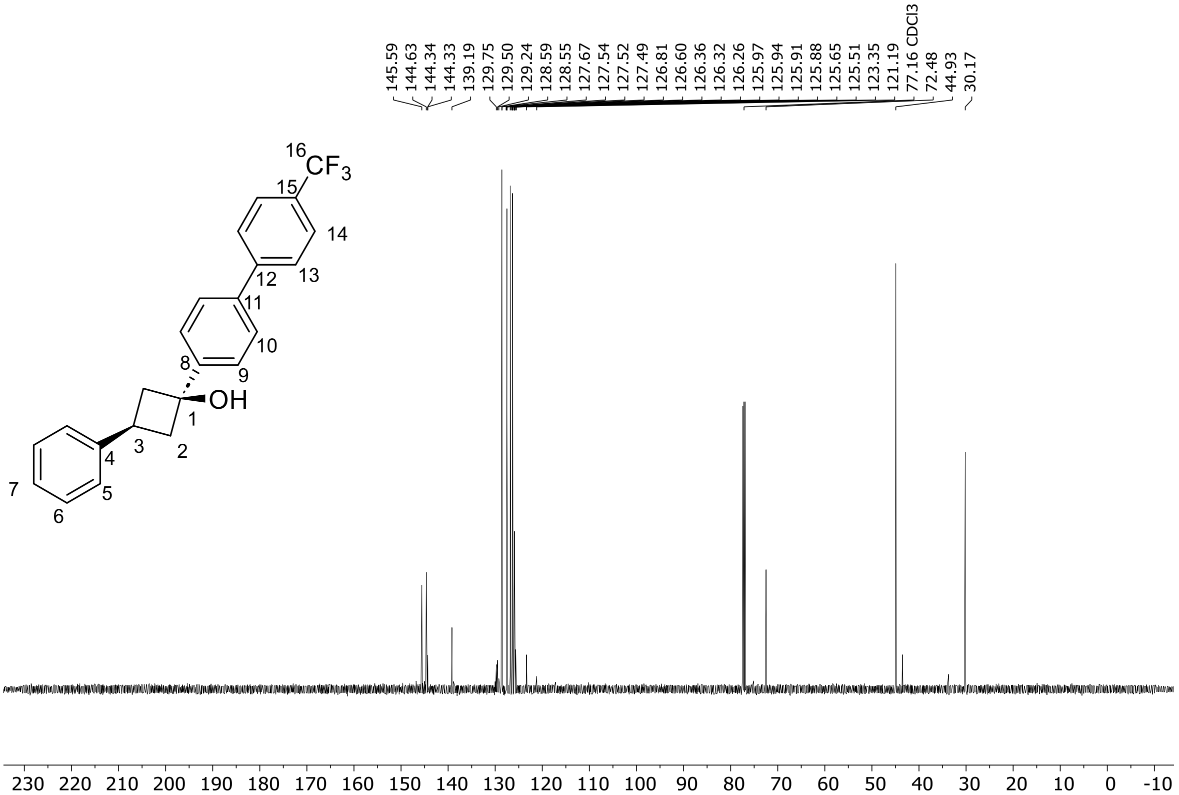


**Supplementary Figure 50.** $^{13}$C{$^1$H} NMR of **1l** (126 MHz, 299 K, $CDCl_3$).

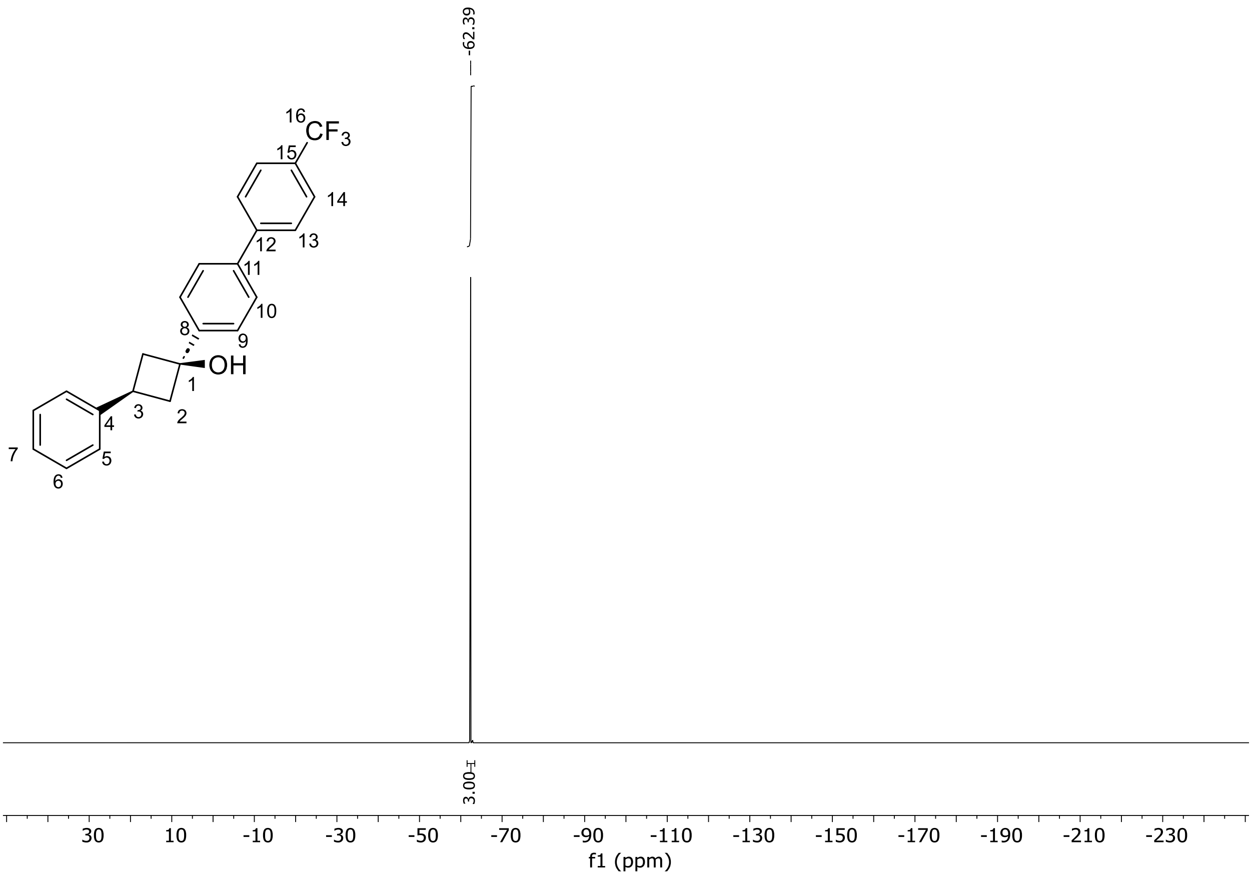


**Supplementary Figure 51.** $^{19}F$ NMR of **1l** (376 MHz, 299 K, $CDCl_3$).

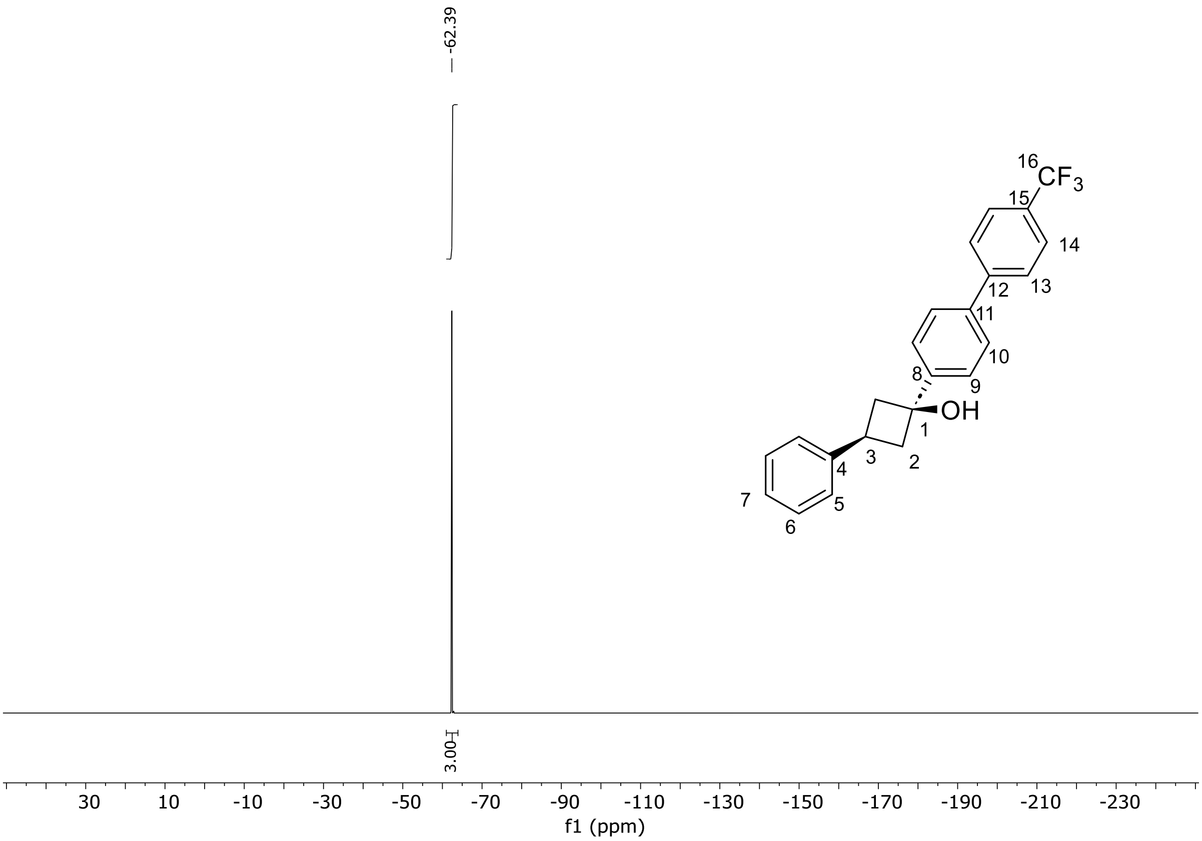


**Supplementary Figure 52.** $^{19}F\{^1H\}$ NMR of **1l** (376 MHz, 299 K, $CDCl_3$).

## 1-(3-Bromophenyl)-3-phenylcyclobutan-1-ol (1m)

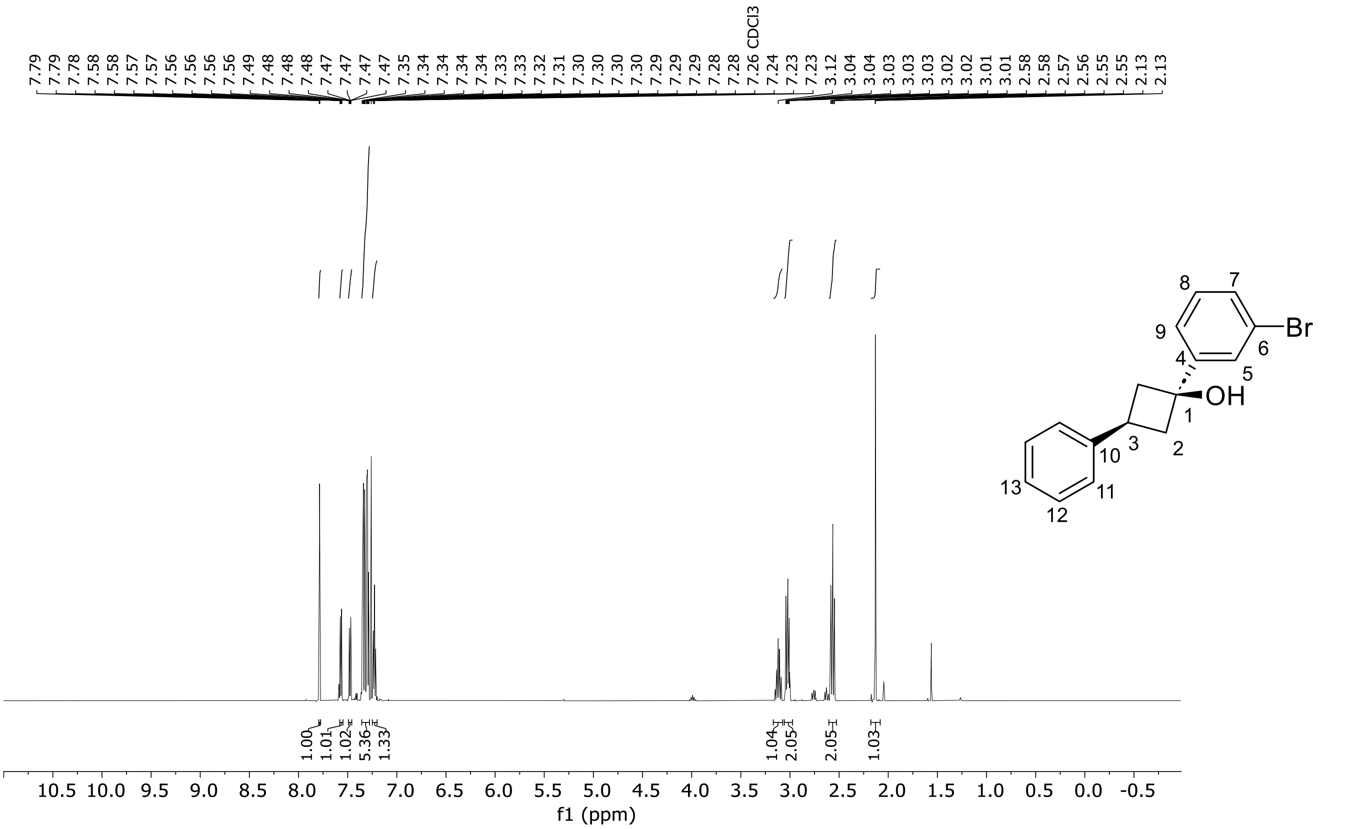


**Supplementary Figure 53.** $^1H$ NMR of **1m** (599 MHz, 299 K, $CDCl_3$).

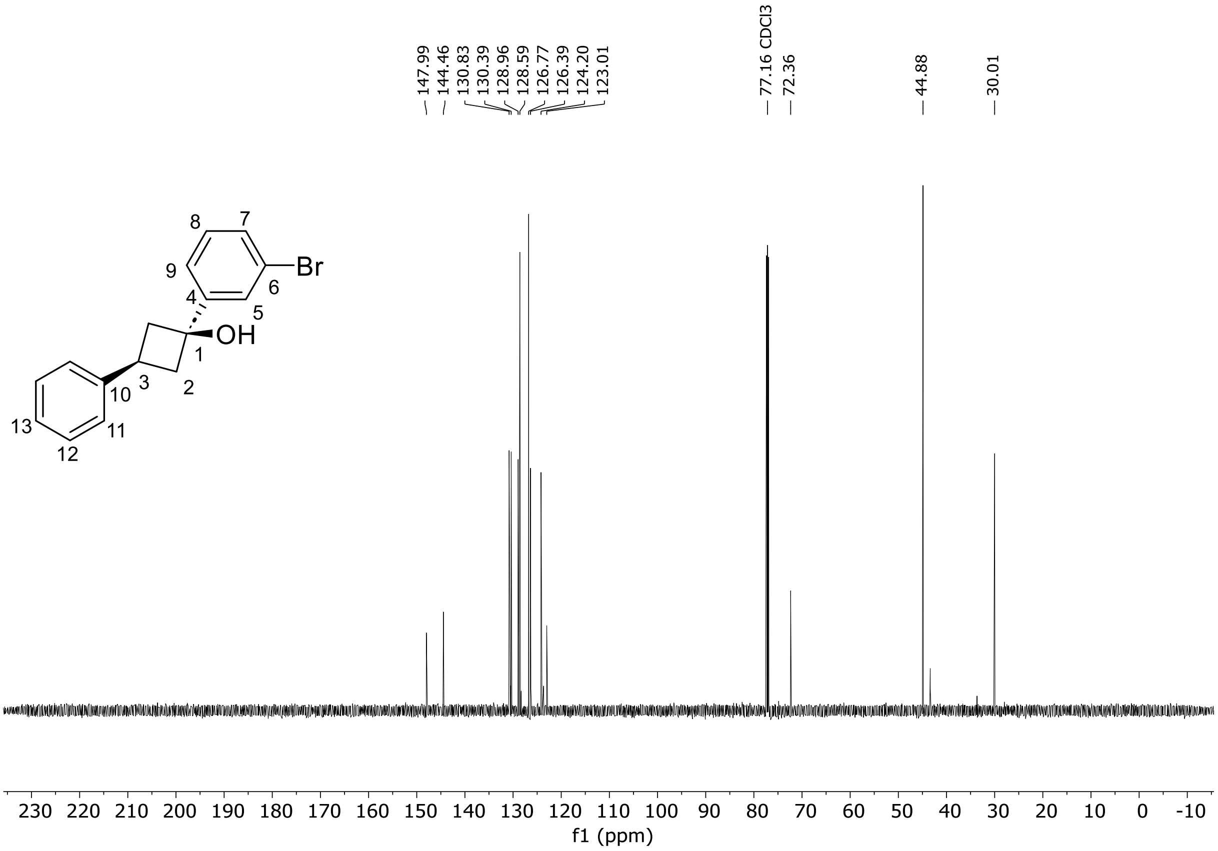


**Supplementary Figure 54.** $^{13}C\{^1H\}$ NMR of **1m** (151 MHz, 299 K, $CDCl_3$).

**1-(2-Bromophenyl)-3-phenylcyclobutan-1-ol (1n)**

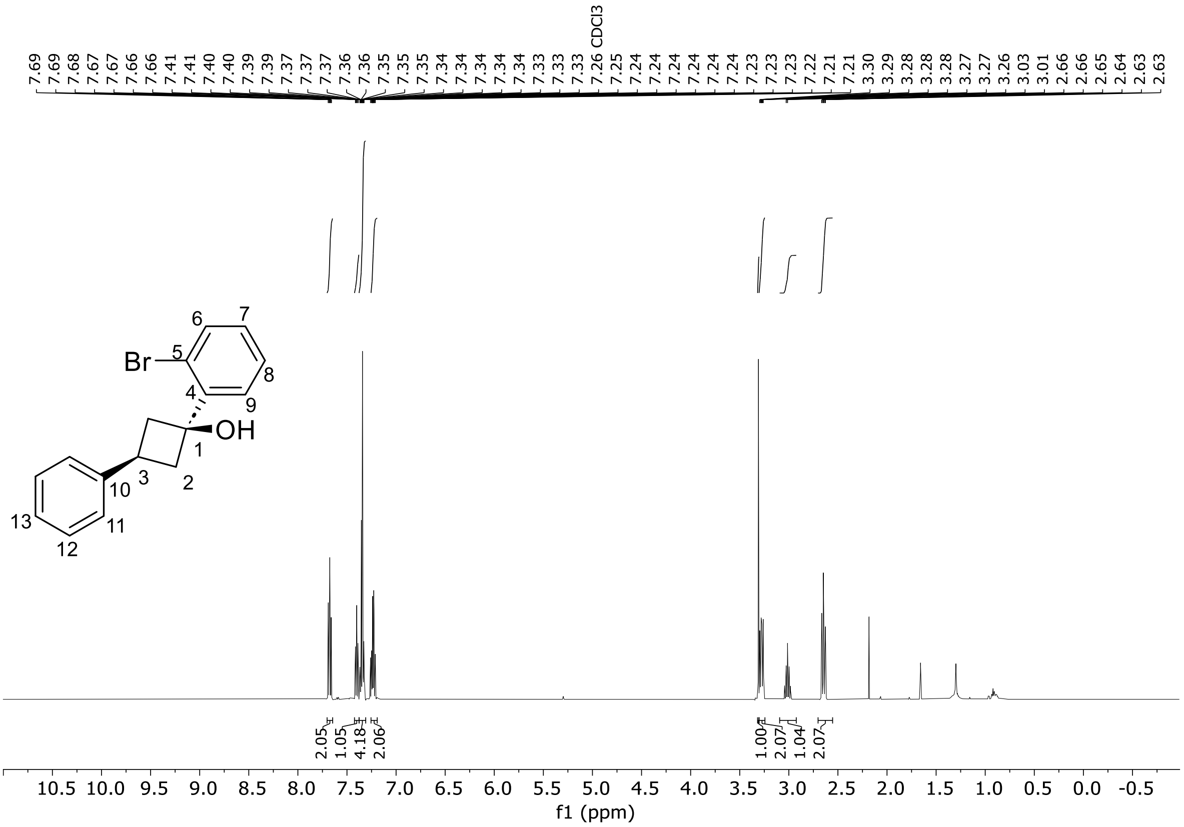


**Supplementary Figure 55.** $^{1}H$ NMR of **1n** (599 MHz, 299 K, $CDCl_3$).

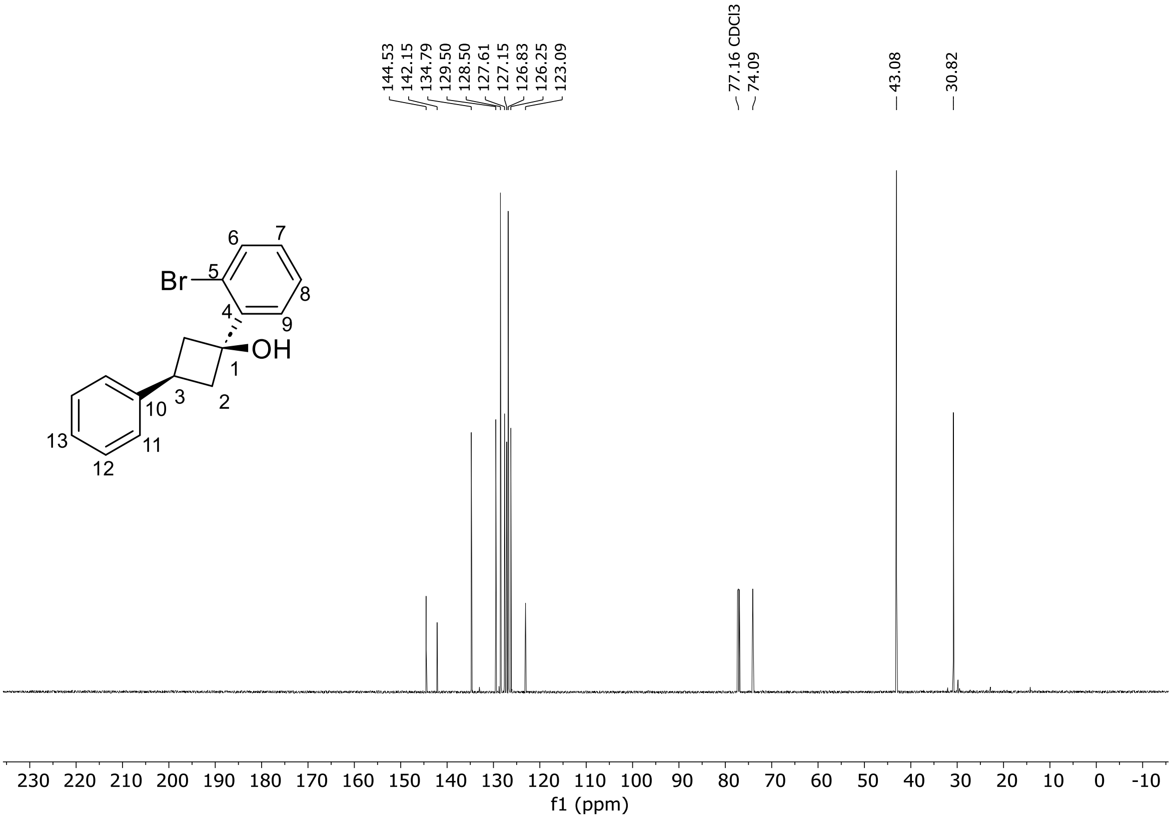


**Supplementary Figure 56.** $^{13}C\{^{1}H\}$ NMR of **1n** (151 MHz, 299 K, $CDCl_3$).

## 3-(4-Fluorophenyl)-1-phenylcyclobutan-1-ol (1o)

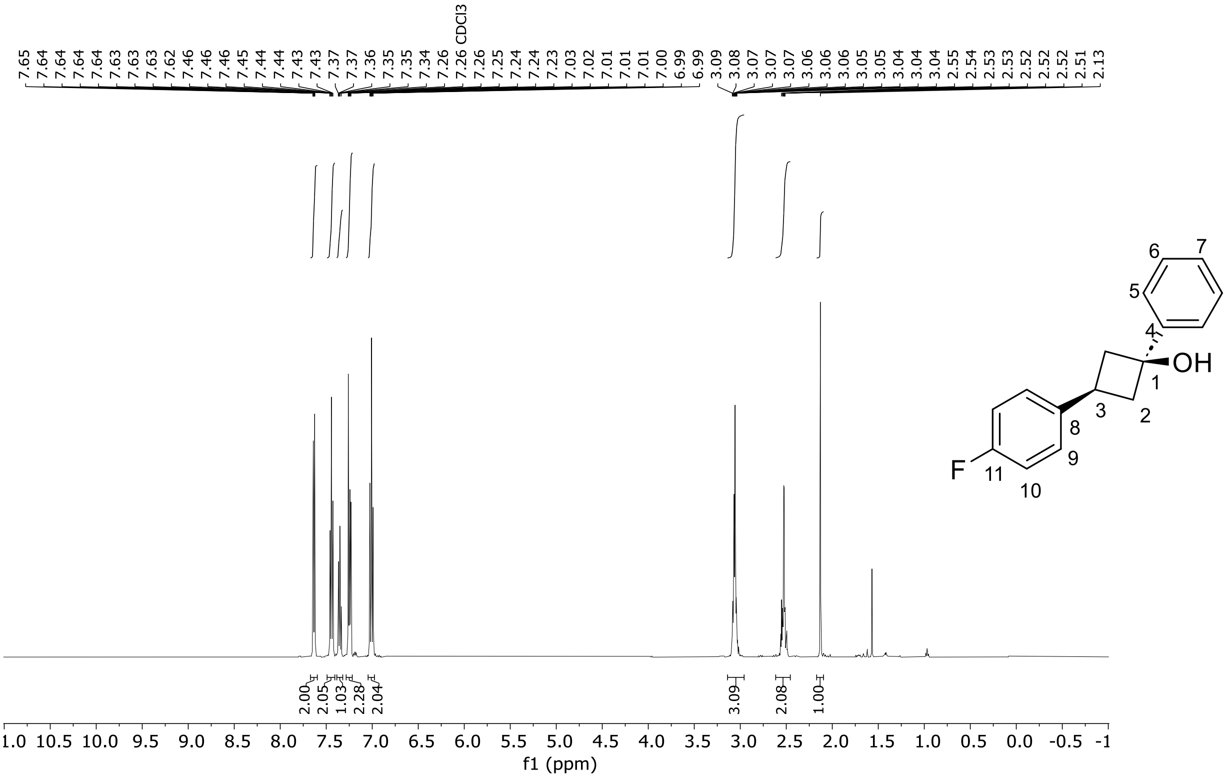


**Supplementary Figure 57.** $^{1}$H NMR of **1o** (500 MHz, 299 K, $CDCl_3$).

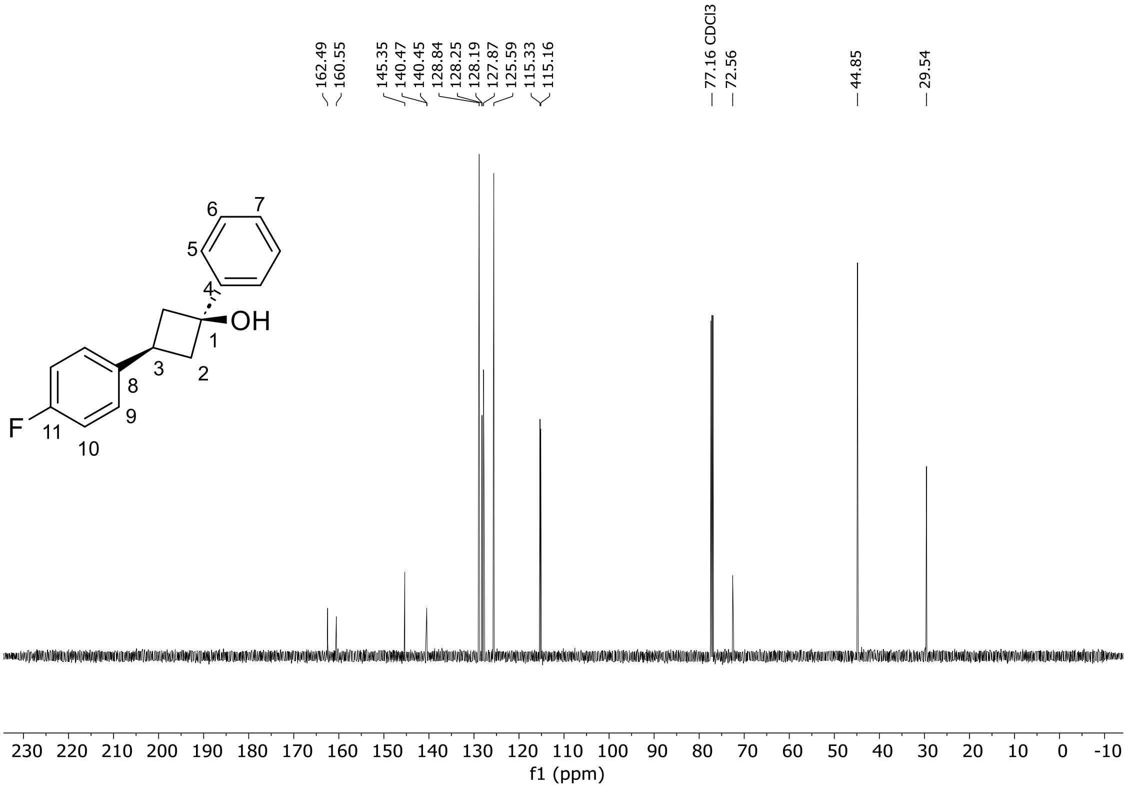


**Supplementary Figure 58.** $^{13}C\{^{1}H\}$ NMR of **1o** (126 MHz, 299 K, $CDCl_3$).

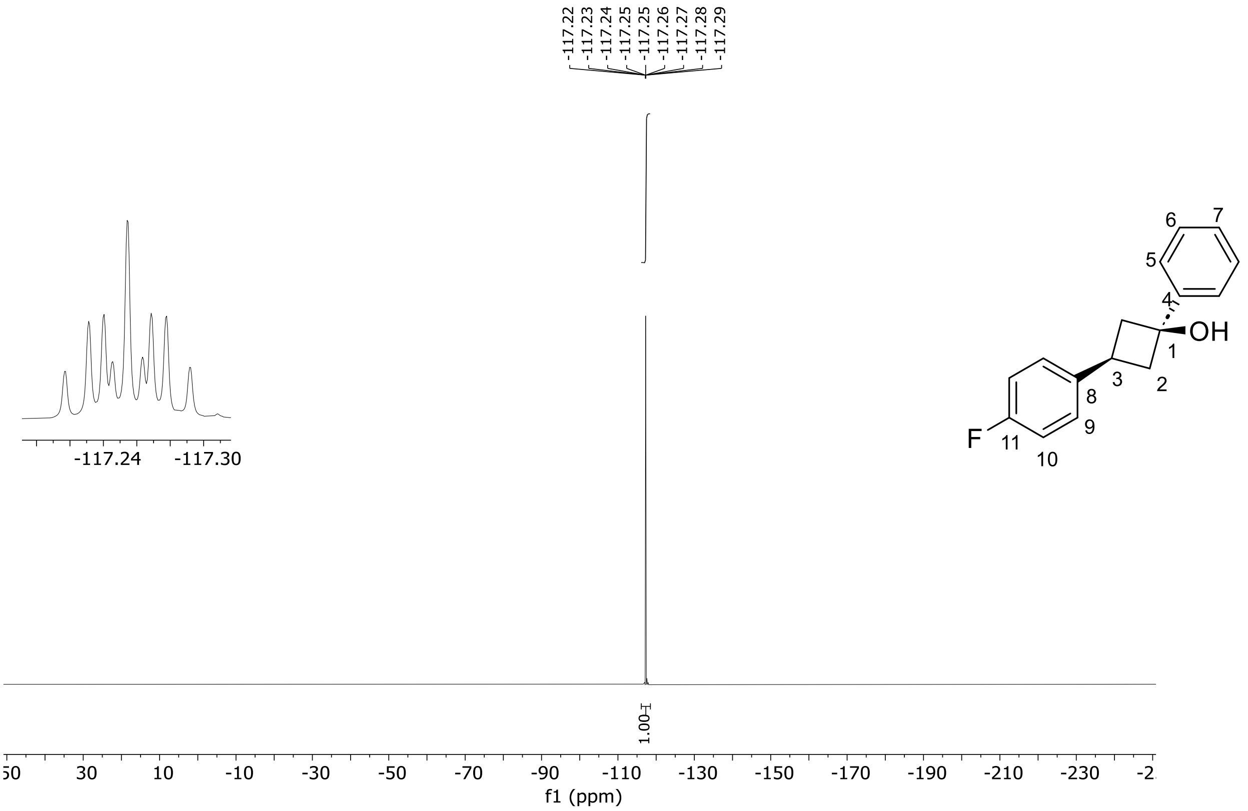


**Supplementary Figure 59.** $^{19}$F NMR of **1o** (376 MHz, 299 K, CDCl$_3$).

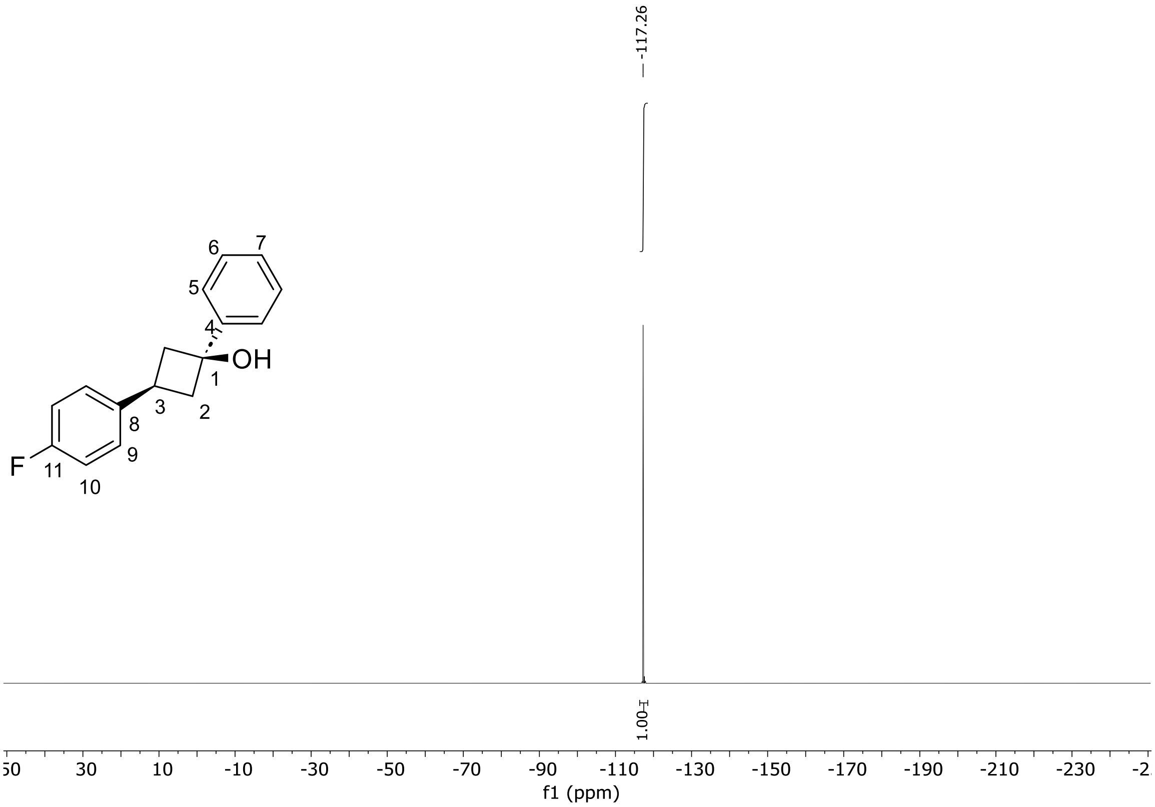


**Supplementary Figure 60.** $^{19}$F{$^{1}$H} NMR of **1o** (376 MHz, 299 K, CDCl$_3$).

**3-(4-Chlorophenyl)-1-phenylcyclobutan-1-ol (1p)**

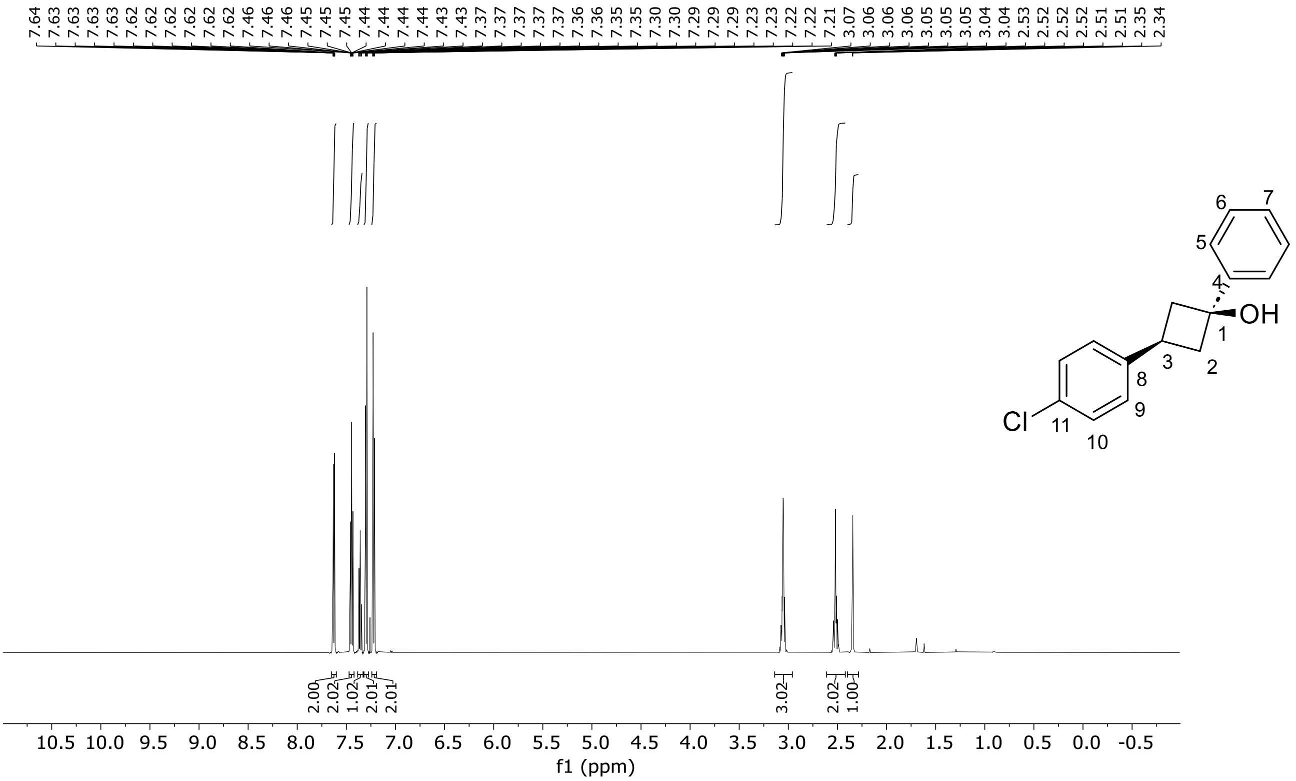


**Supplementary Figure 61.** $^{1}H$ NMR of **1p** (599 MHz, 299 K, $CDCl_3$).

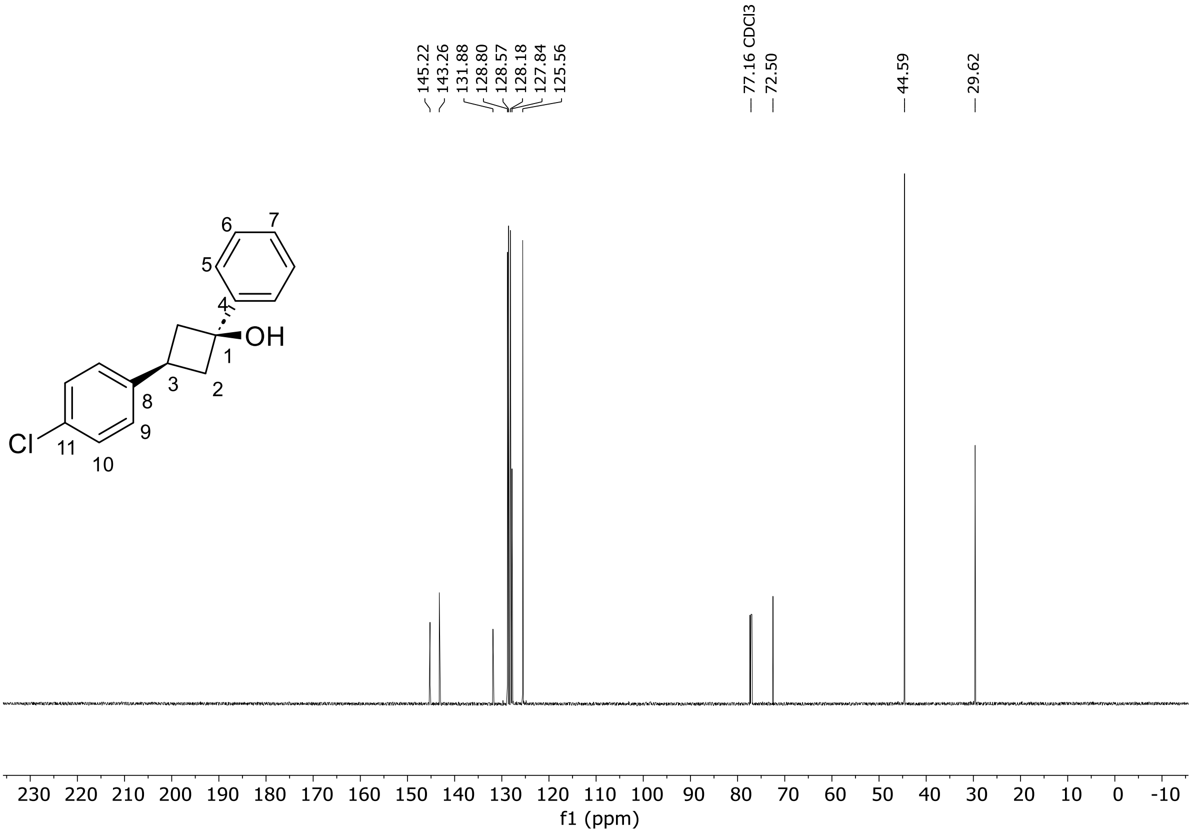


**Supplementary Figure 62.** $^{13}C\{^{1}H\}$ NMR of **1p** (151 MHz, 299 K, $CDCl_3$).

**3-(4-Bromophenyl)-1-phenylcyclobutan-1-ol (1q)**

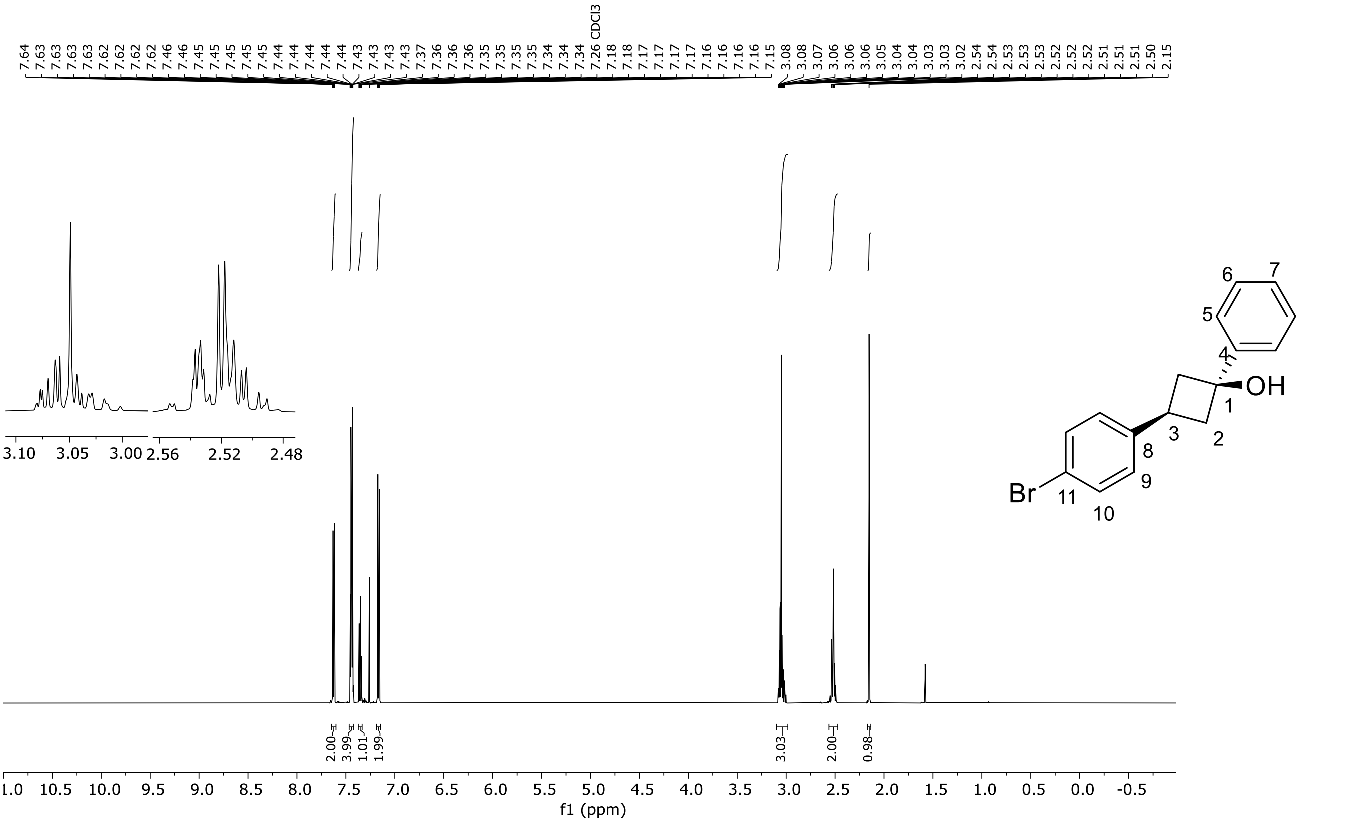


**Supplementary Figure 63.** $^{1}$H NMR of **1q** (599 MHz, 299 K, $CDCl_3$).

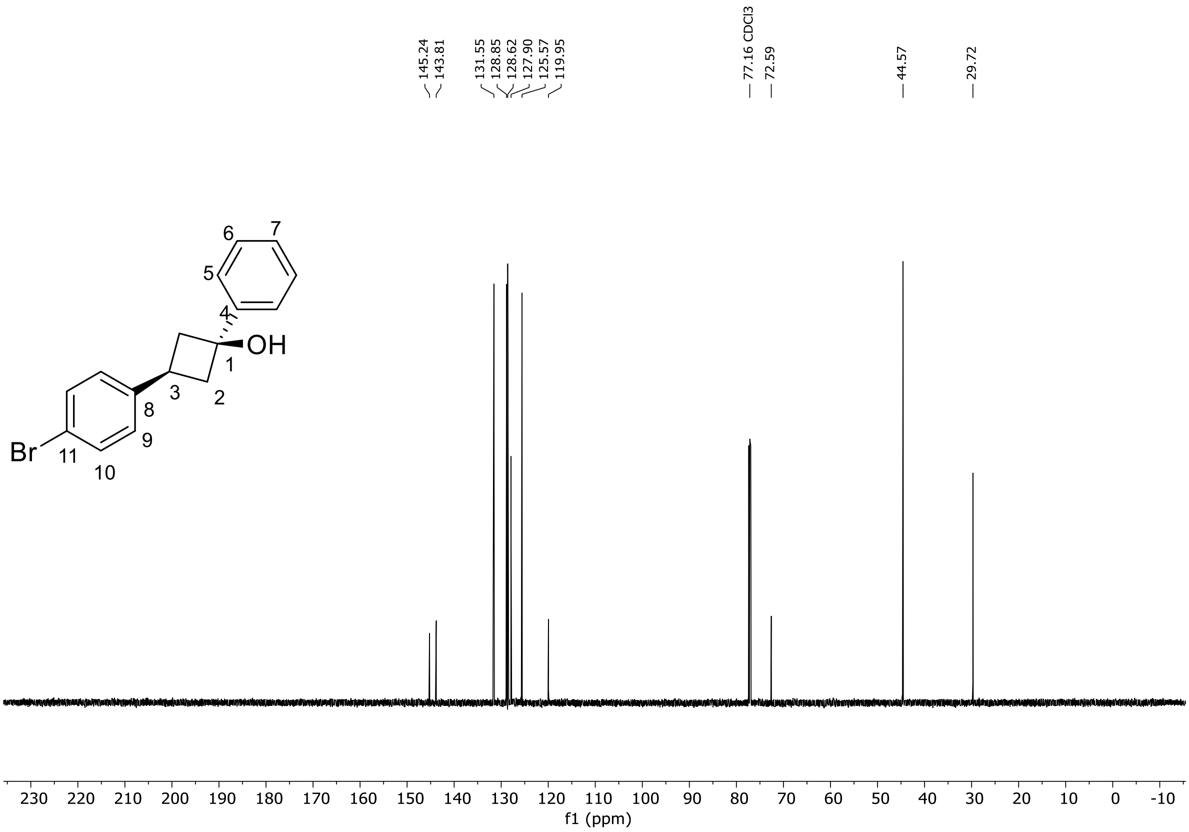


**Supplementary Figure 64.** $^{13}C\{^{1}H\}$ NMR of **1q** (151 MHz, 299 K, $CDCl_3$).

**1-Phenyl-3-(4-(trifluoromethyl)phenyl)cyclobutan-1-ol (1r)**

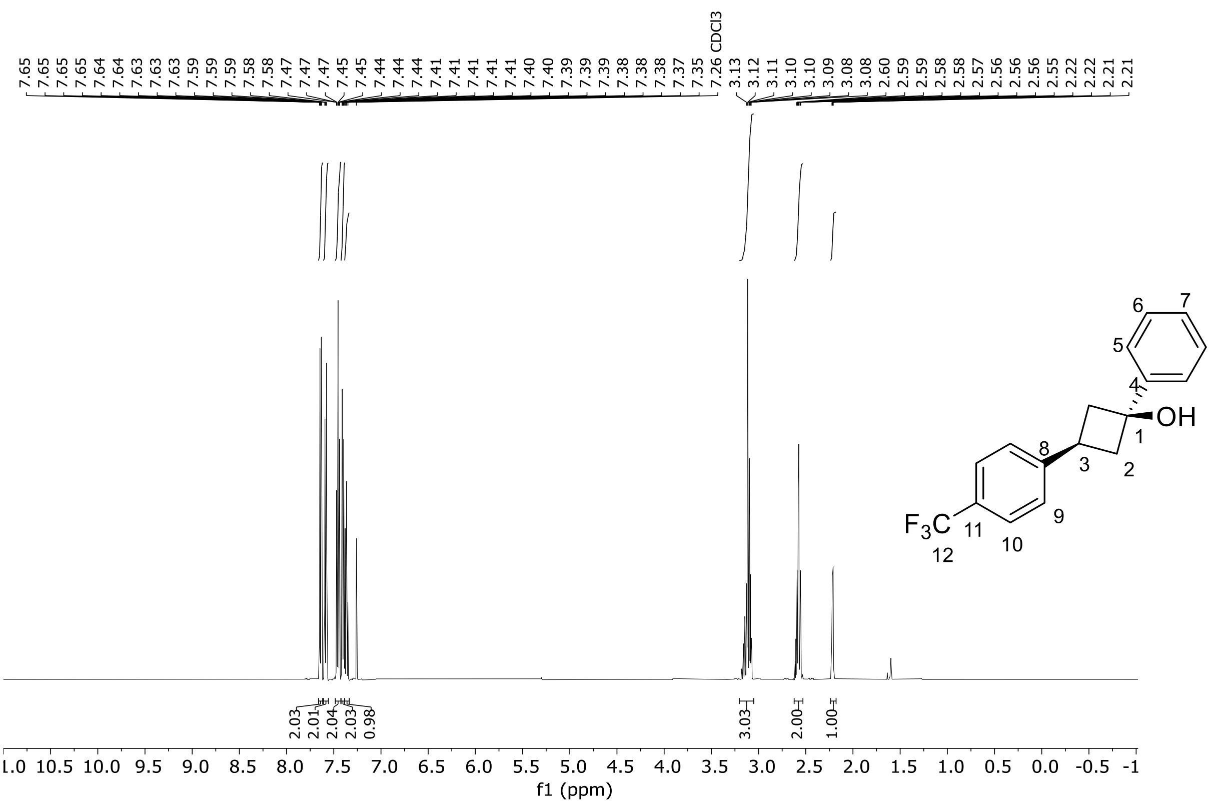


**Supplementary Figure 65.** $^{1}$H NMR of **1r** (500 MHz, 299 K, $CDCl_3$).

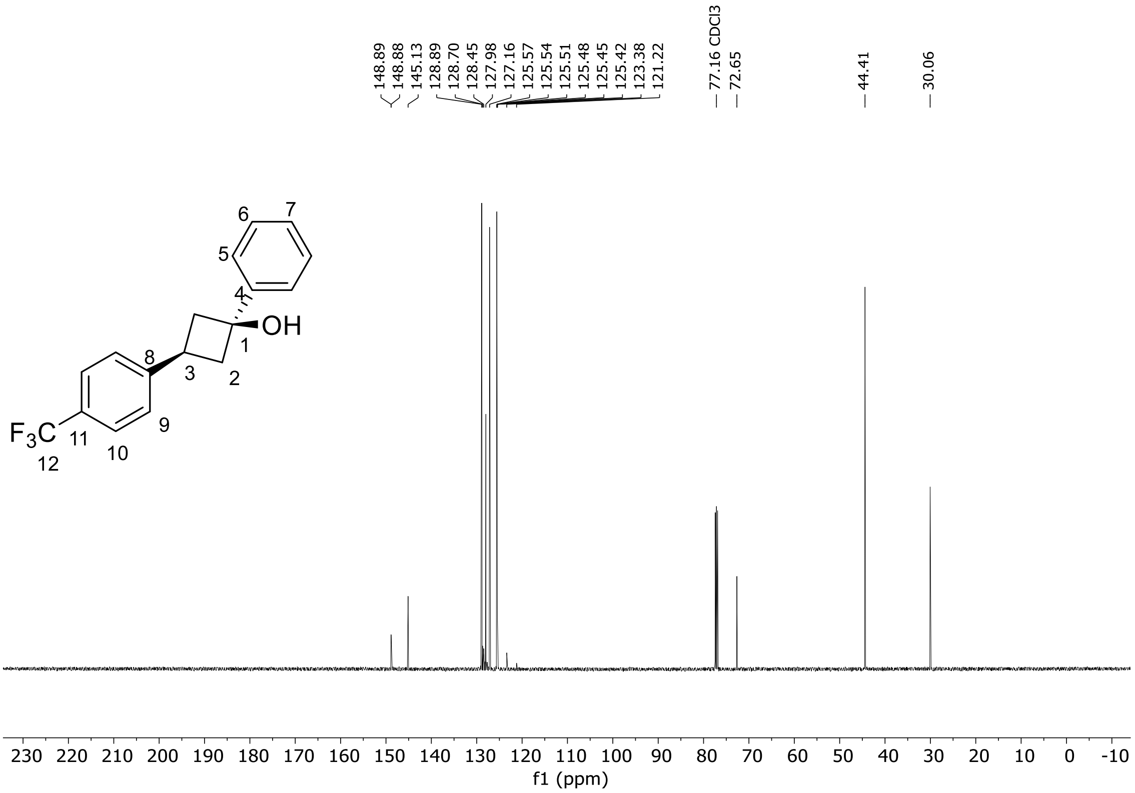


**Supplementary Figure 66.** $^{13}C\{^{1}H\}$ NMR of **1r** (126 MHz, 299 K, $CDCl_3$).

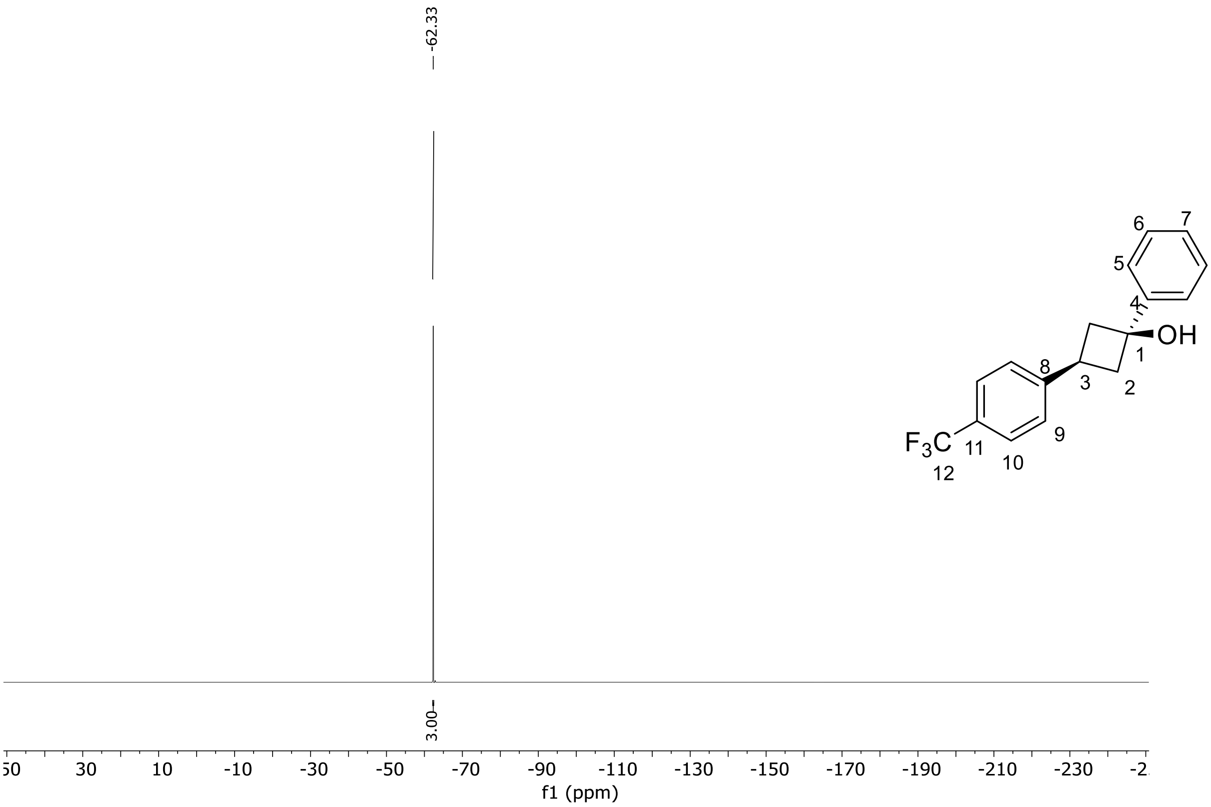


**Supplementary Figure 67.** $^{19}F$ NMR of **1r** (376 MHz, 299 K, $CDCl_3$).

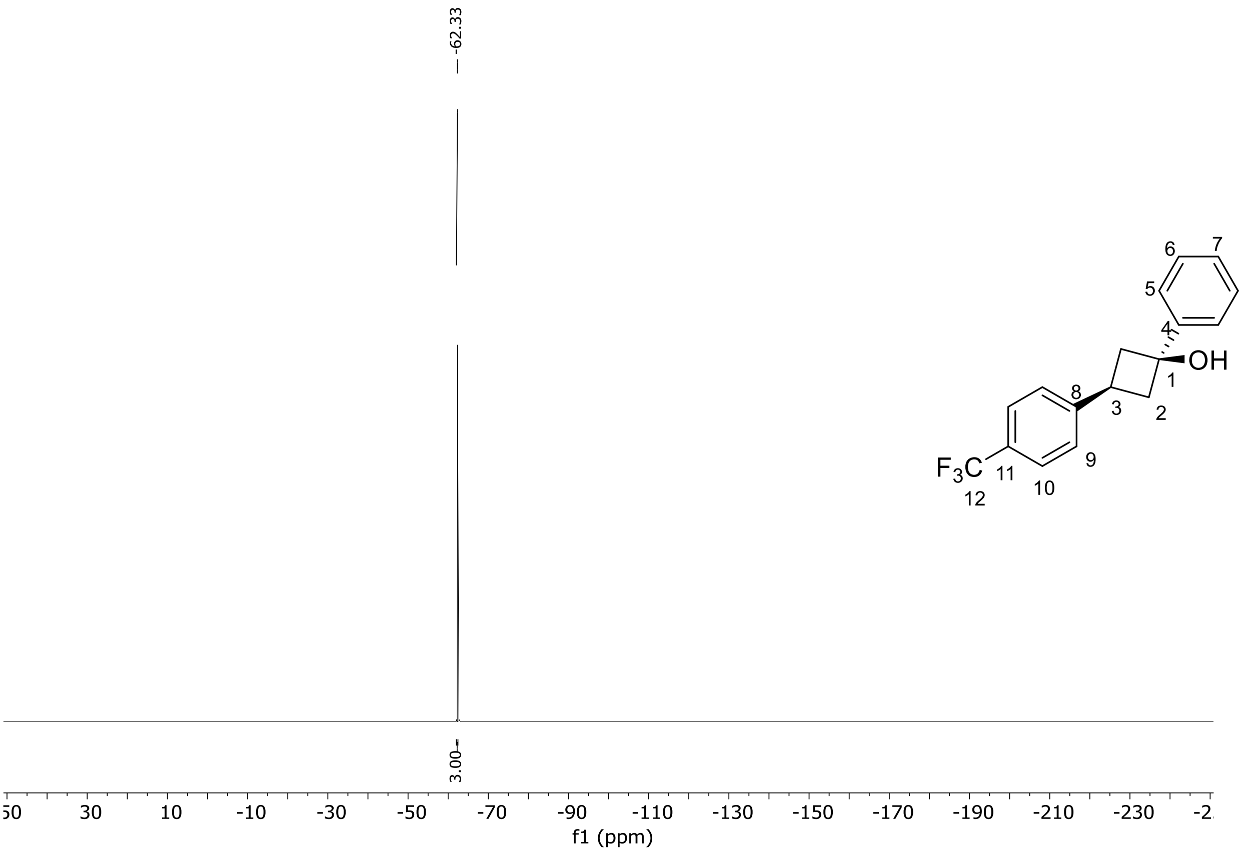


**Supplementary Figure 68.** $^{19}F\{^1H\}$ NMR of **1r** (376 MHz, 299 K, $CDCl_3$).

## 4-(3-Hydroxy-3-phenylcyclobutyl)phenyl trifluoromethanesulfonate (1s)

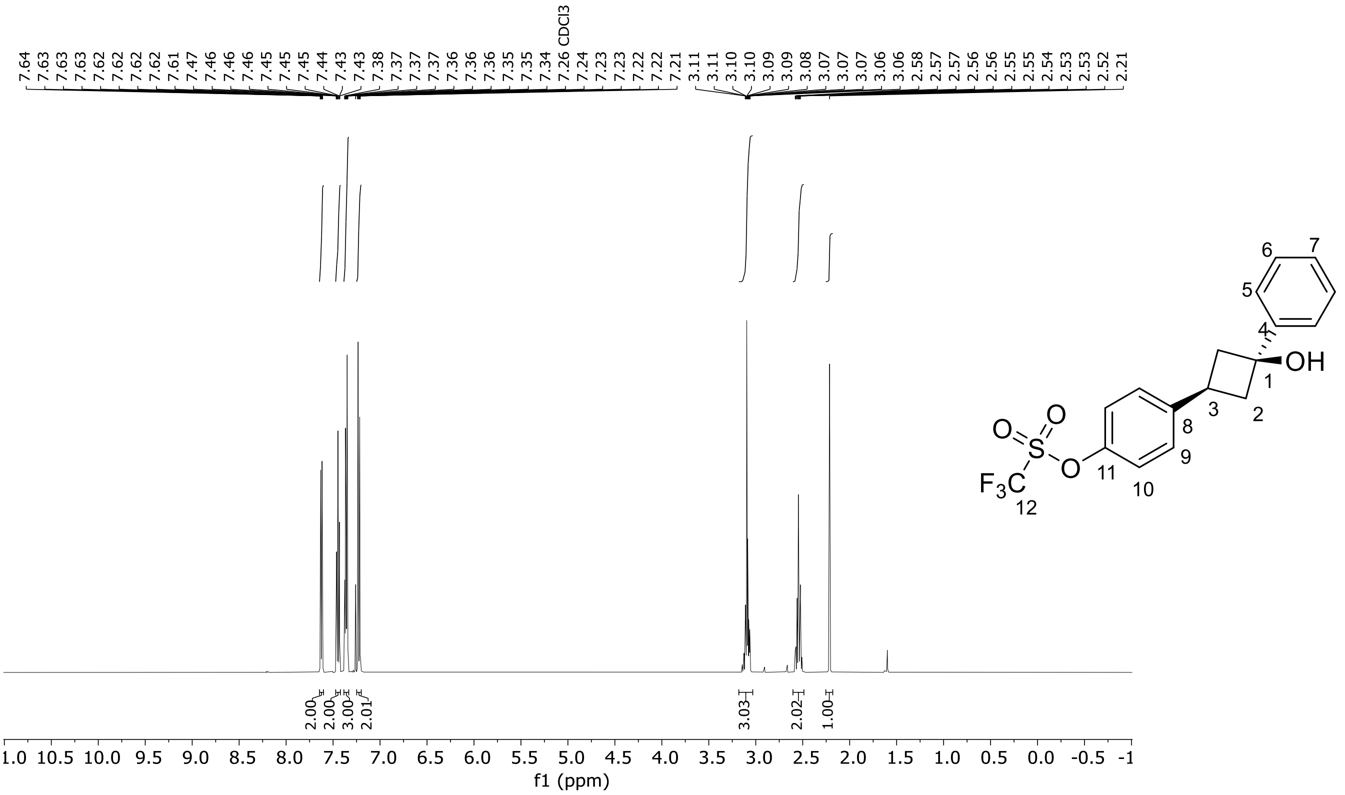


**Supplementary Figure 69.** $^{1}$H NMR of **1s** (500 MHz, 299 K, $CDCl_3$).

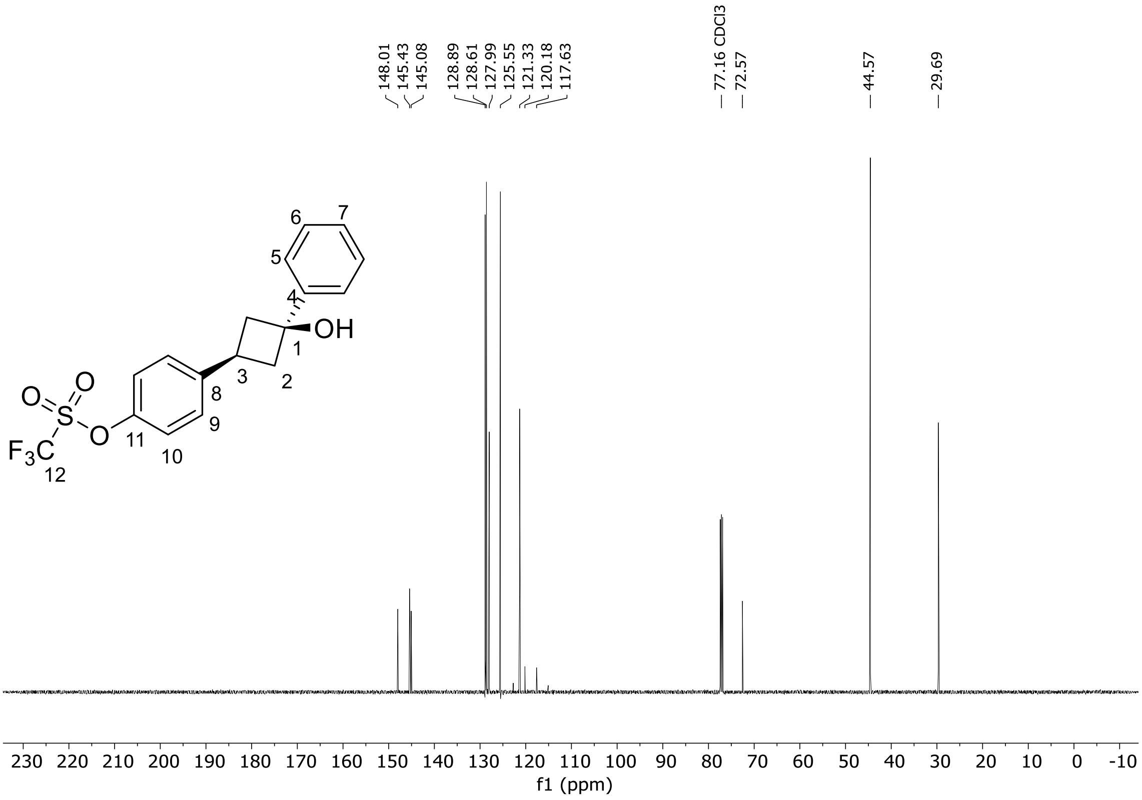


**Supplementary Figure 70.** $^{13}$C{$^{1}$H} NMR of **1s** (126 MHz, 299 K, $CDCl_3$).

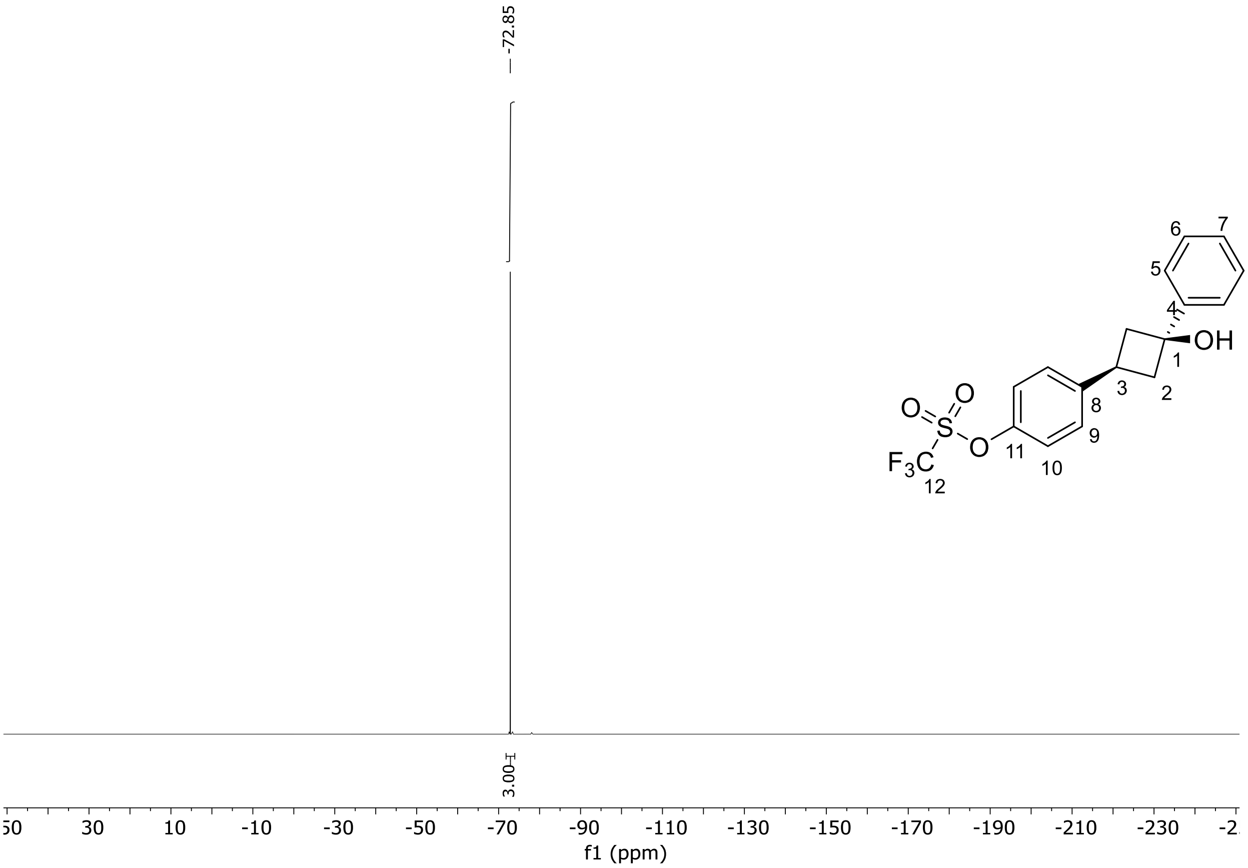


**Supplementary Figure 71.** $^{19}F$ NMR of **1s** (376 MHz, 299 K, $CDCl_3$).

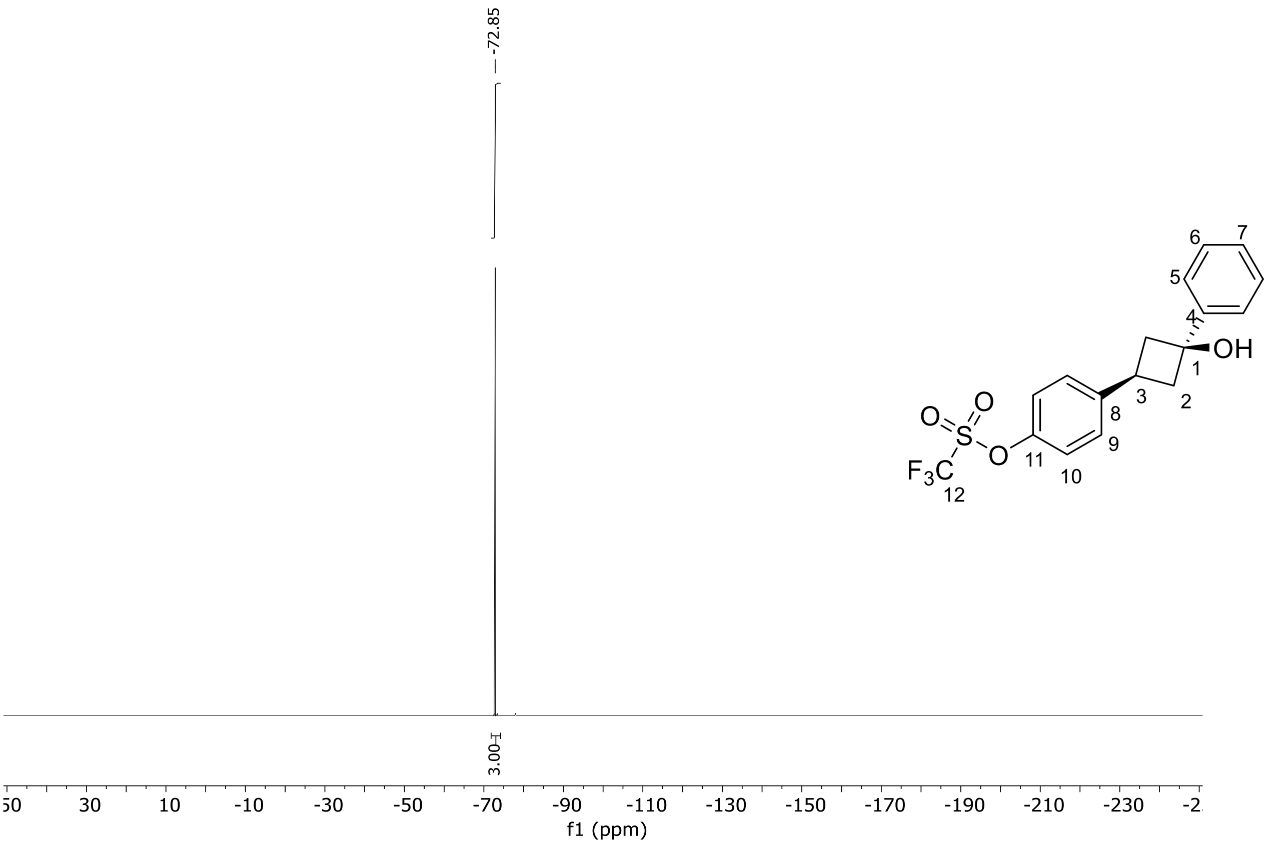


**Supplementary Figure 72.** $^{19}F\{^1H\}$ NMR of **1s** (376 MHz, 299 K, $CDCl_3$).

## 1-(4-Bromophenyl)-3-(4-fluorophenyl)cyclobutan-1-ol (1t)

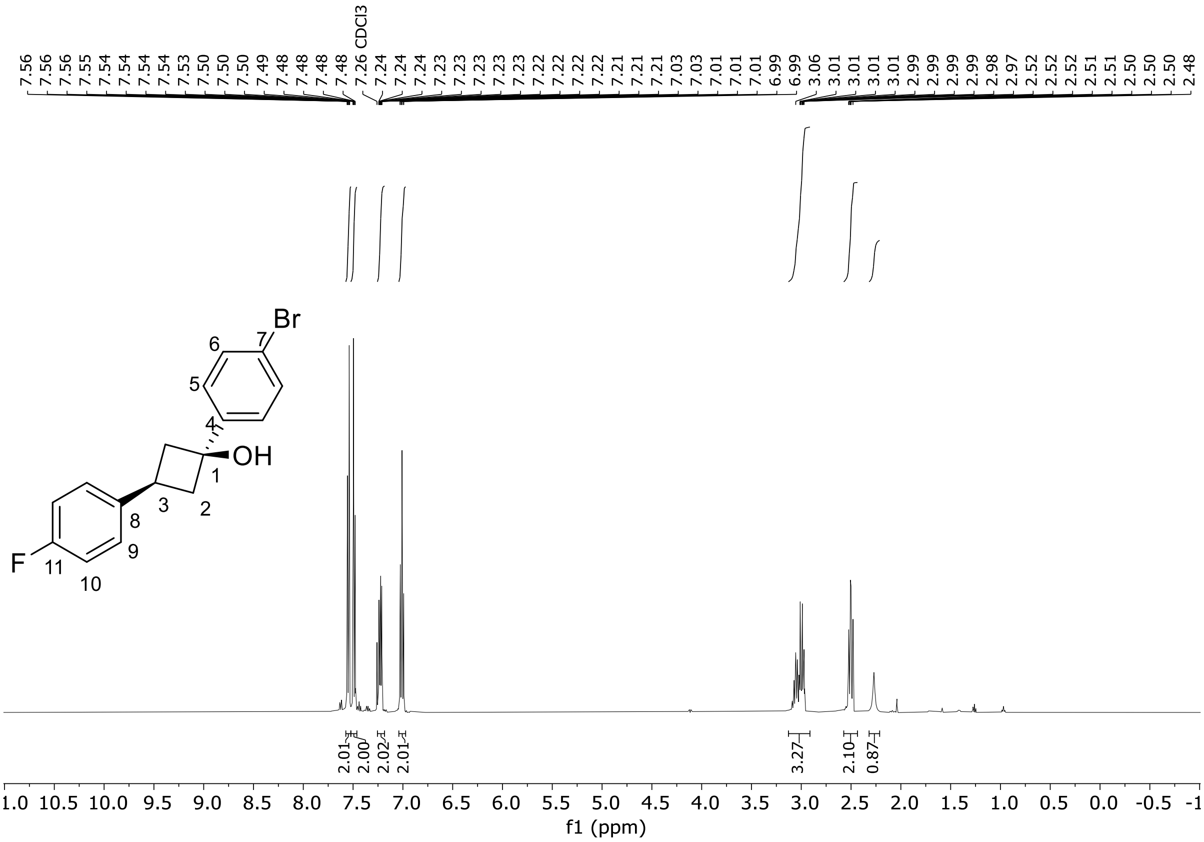


**Supplementary Figure 73.** $^{1}$H NMR of **1t** (500 MHz, 299 K, $CDCl_3$).

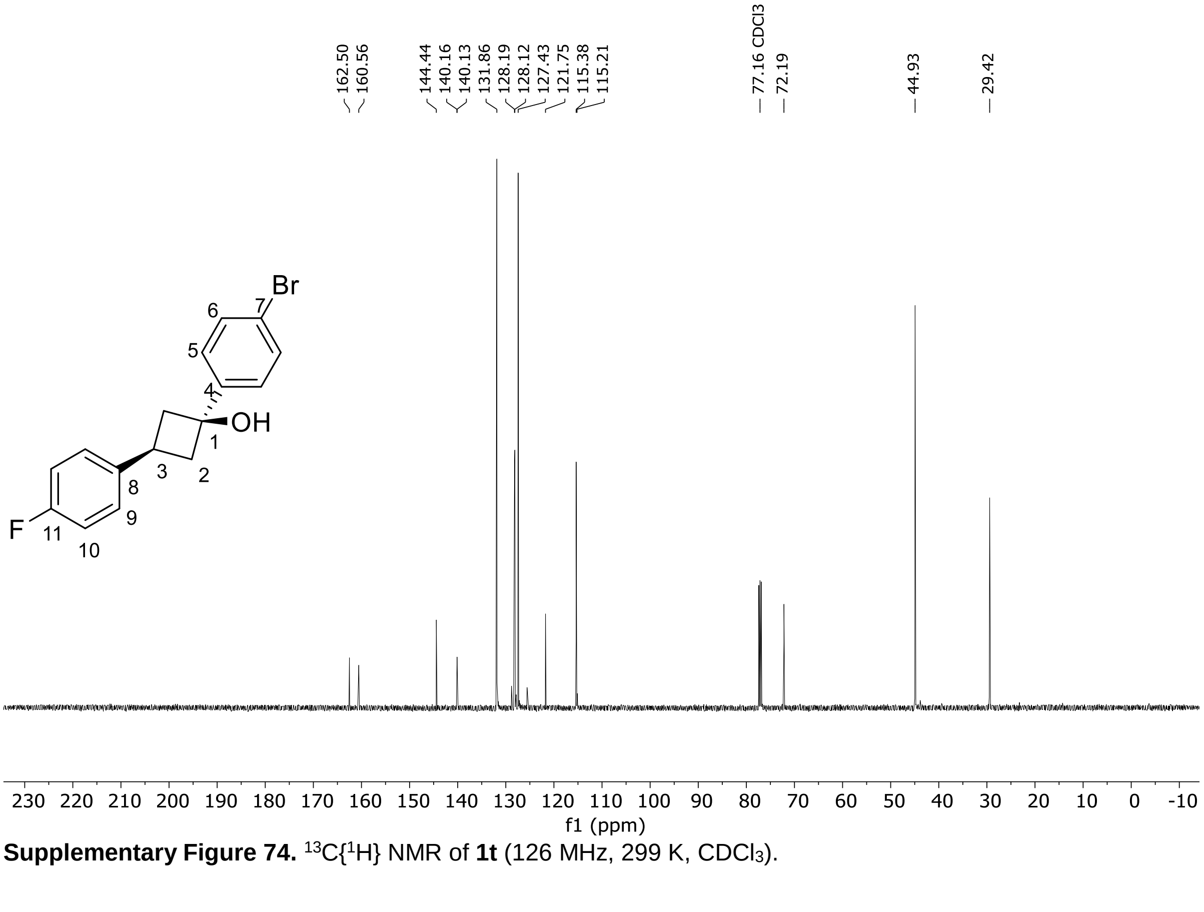


**Supplementary Figure 74.** $^{13}$C{$^{1}$H} NMR of **1t** (126 MHz, 299 K, $CDCl_3$).

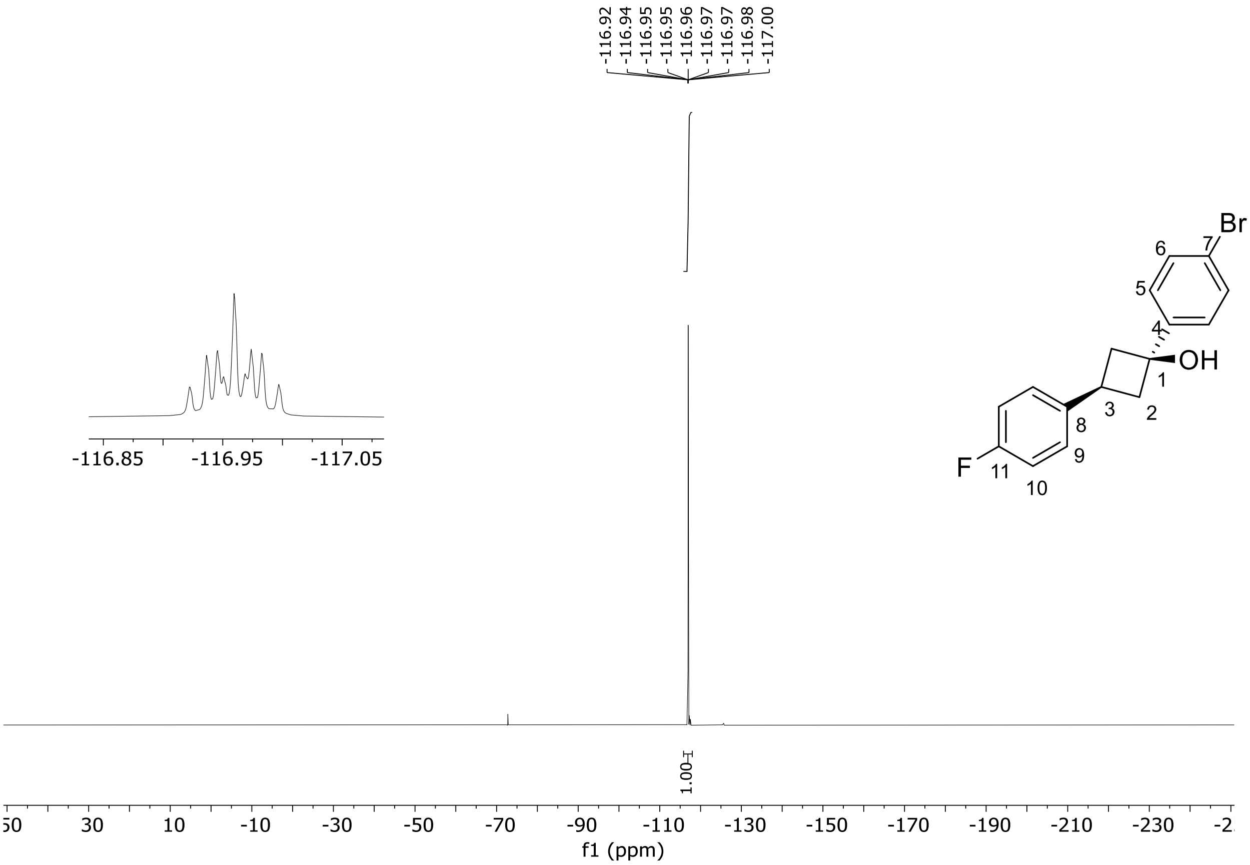


**Supplementary Figure 75.** $^{19}$F NMR of **1t** (376 MHz, 299 K, $CDCl_3$).

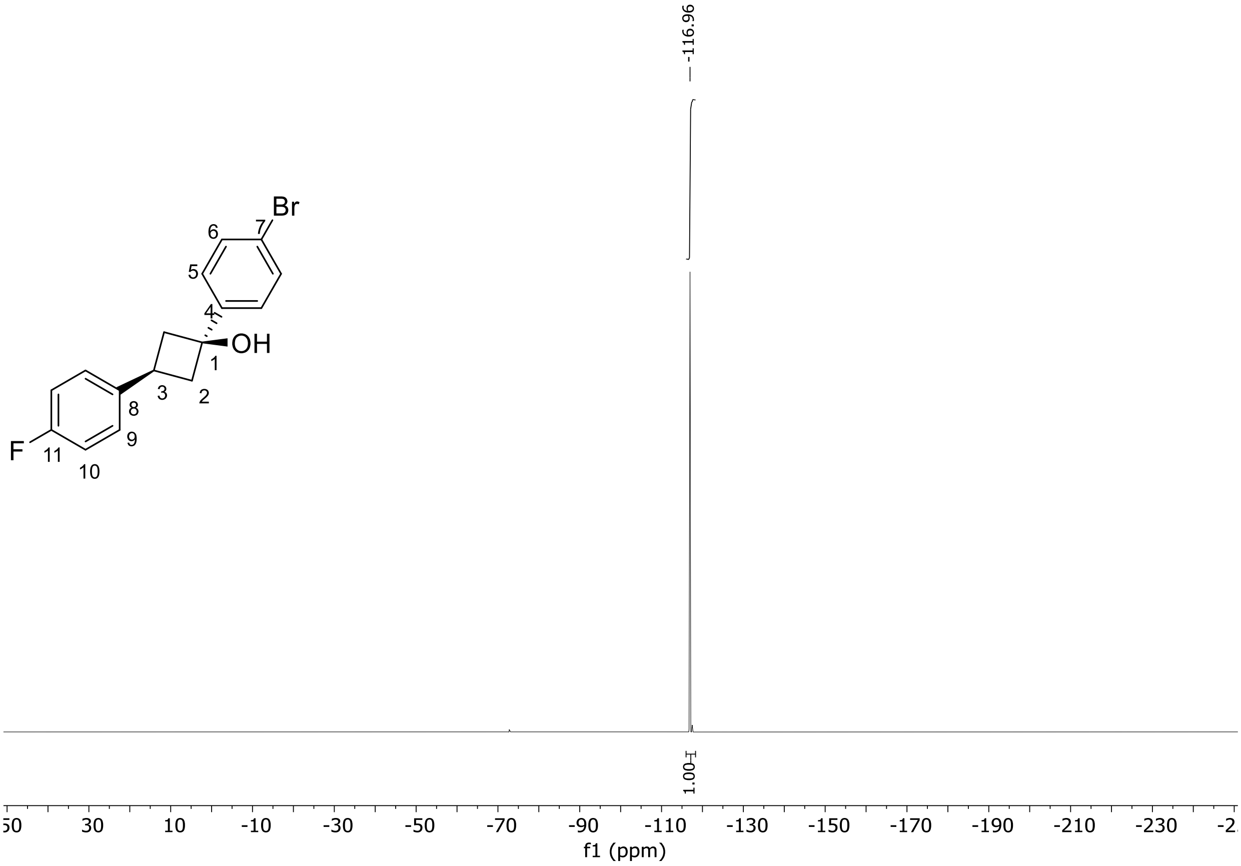


**Supplementary Figure 76.** $^{19}$F{$^1$H} NMR of **1t** (376 MHz, 299 K, $CDCl_3$).

## 1-(4-Bromophenyl)-3-(4-chlorophenyl)cyclobutan-1-ol (1u)

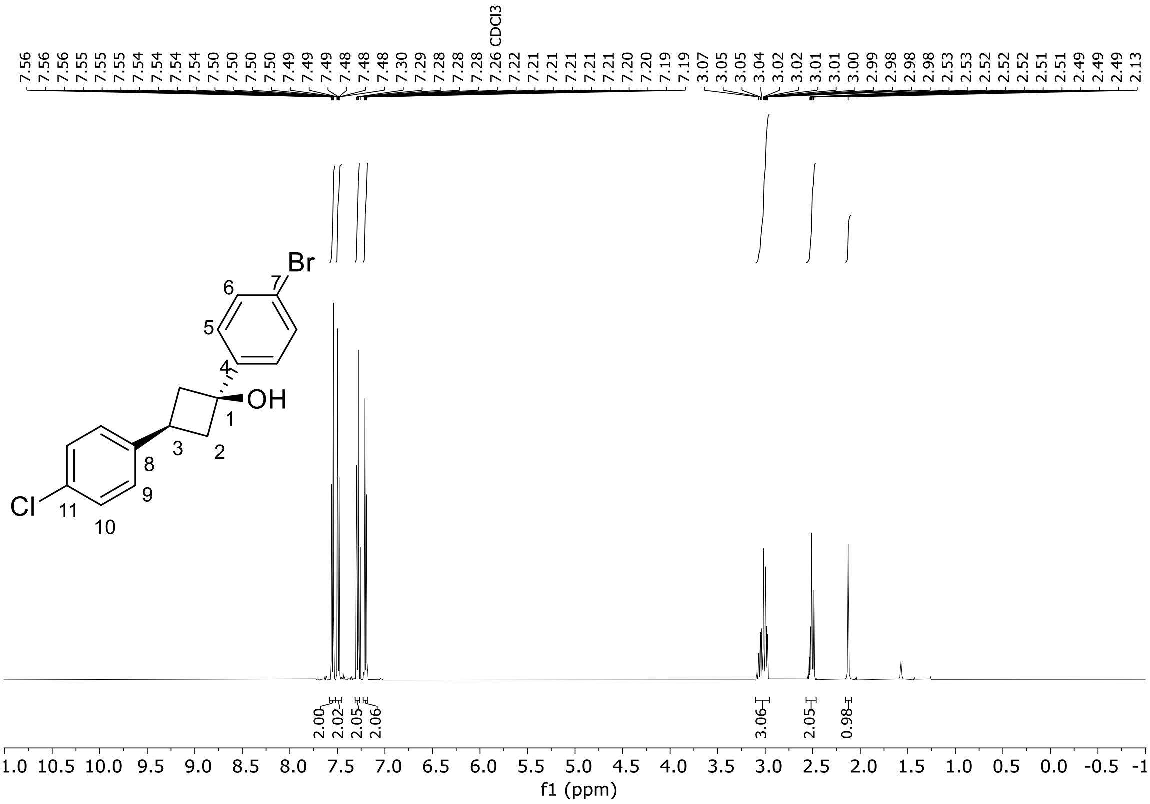

**Supplementary Figure 77.** $^{1}H$ NMR of **1u** (500 MHz, 299 K, $CDCl_3$).

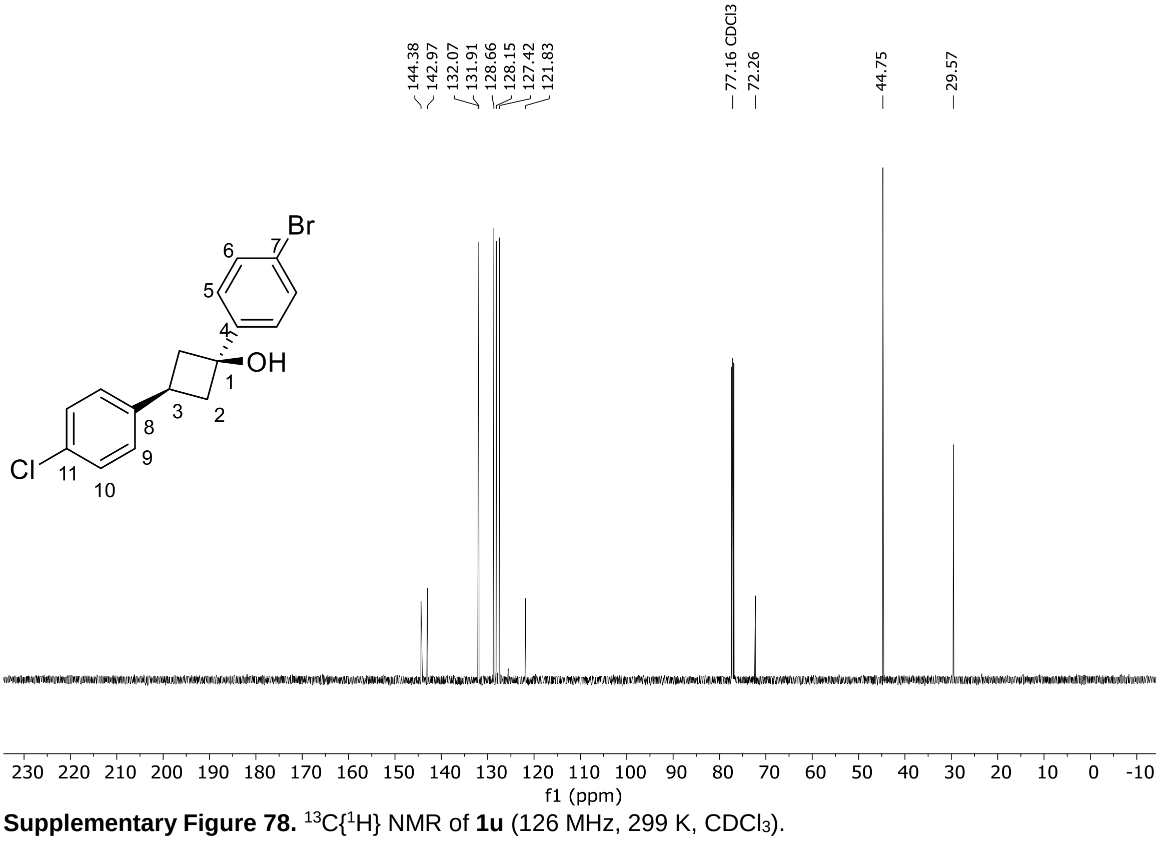

**Supplementary Figure 78.** $^{13}C\{^{1}H\}$ NMR of **1u** (126 MHz, 299 K, $CDCl_3$).

**1-(4-Bromophenyl)-3-(4-(trifluoromethyl)phenyl)cyclobutan-1-ol (1v)**

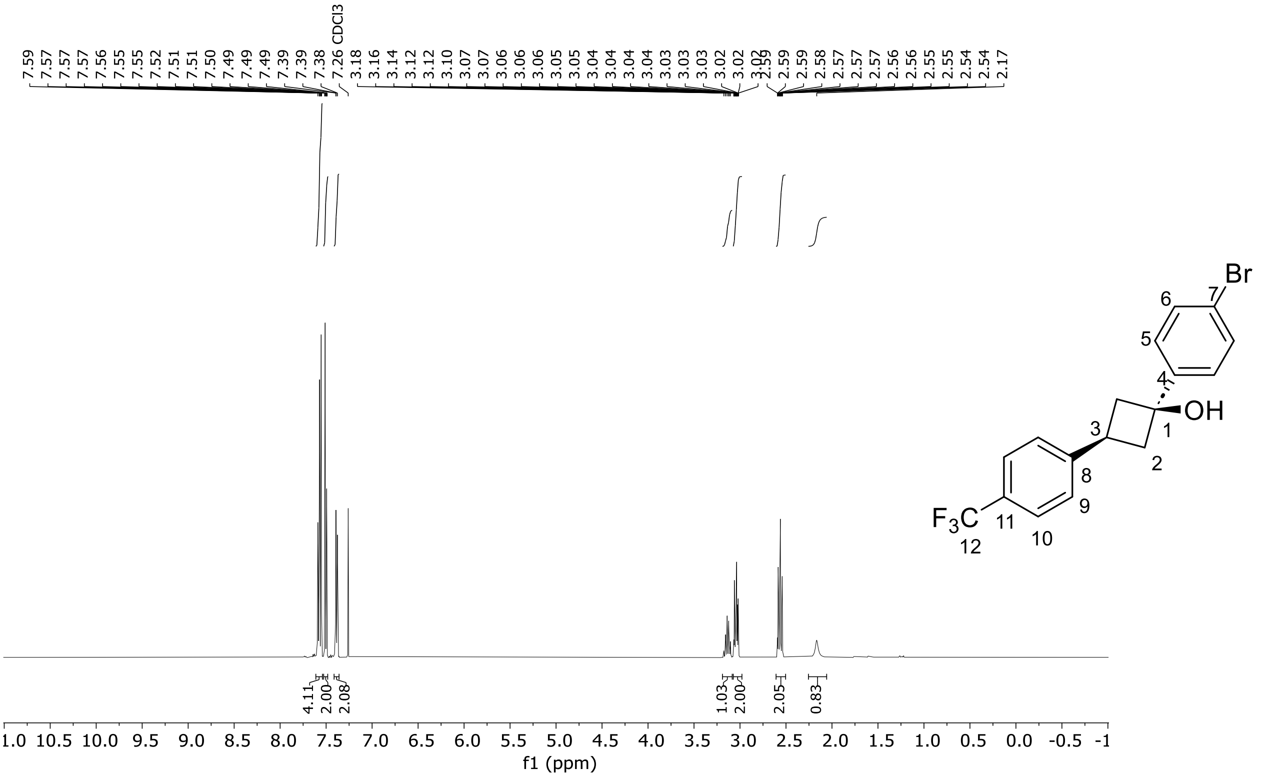


**Supplementary Figure 79.** $^{1}$H NMR of **1v** (500 MHz, 299 K, $CDCl_3$).

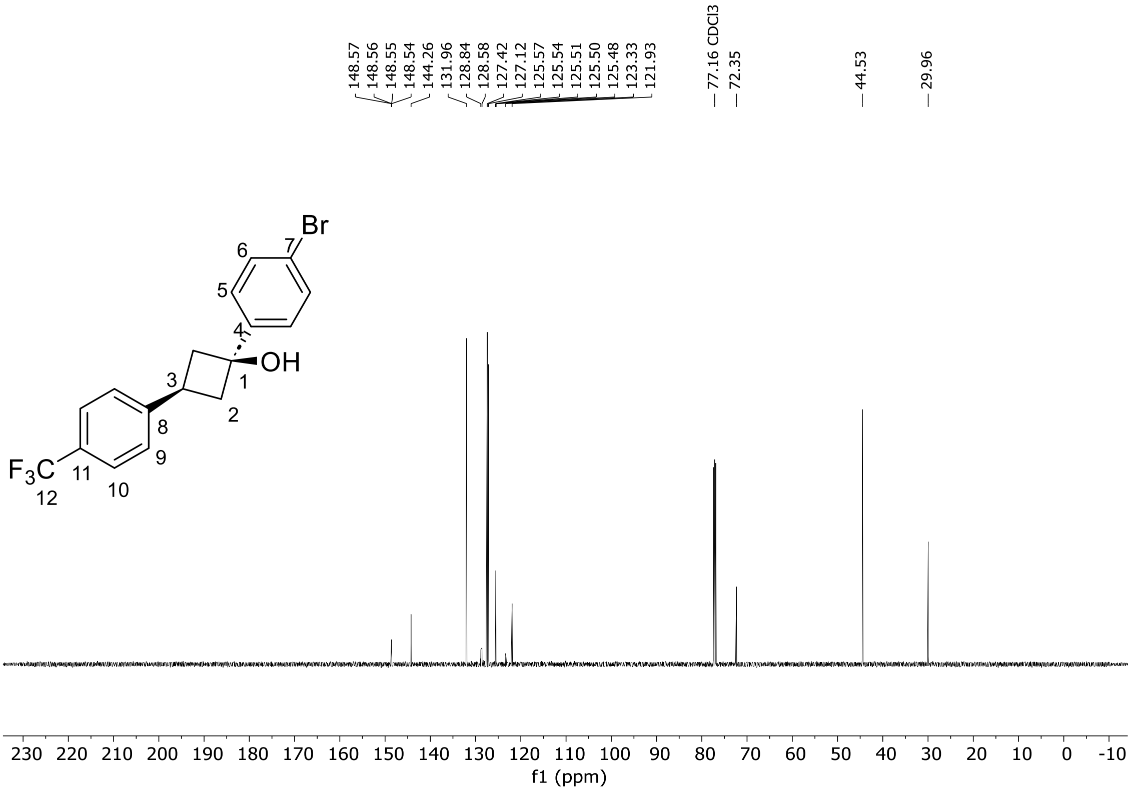


**Supplementary Figure 80.** $^{13}C\{^{1}H\}$ NMR of **1v** (126 MHz, 299 K, $CDCl_3$).

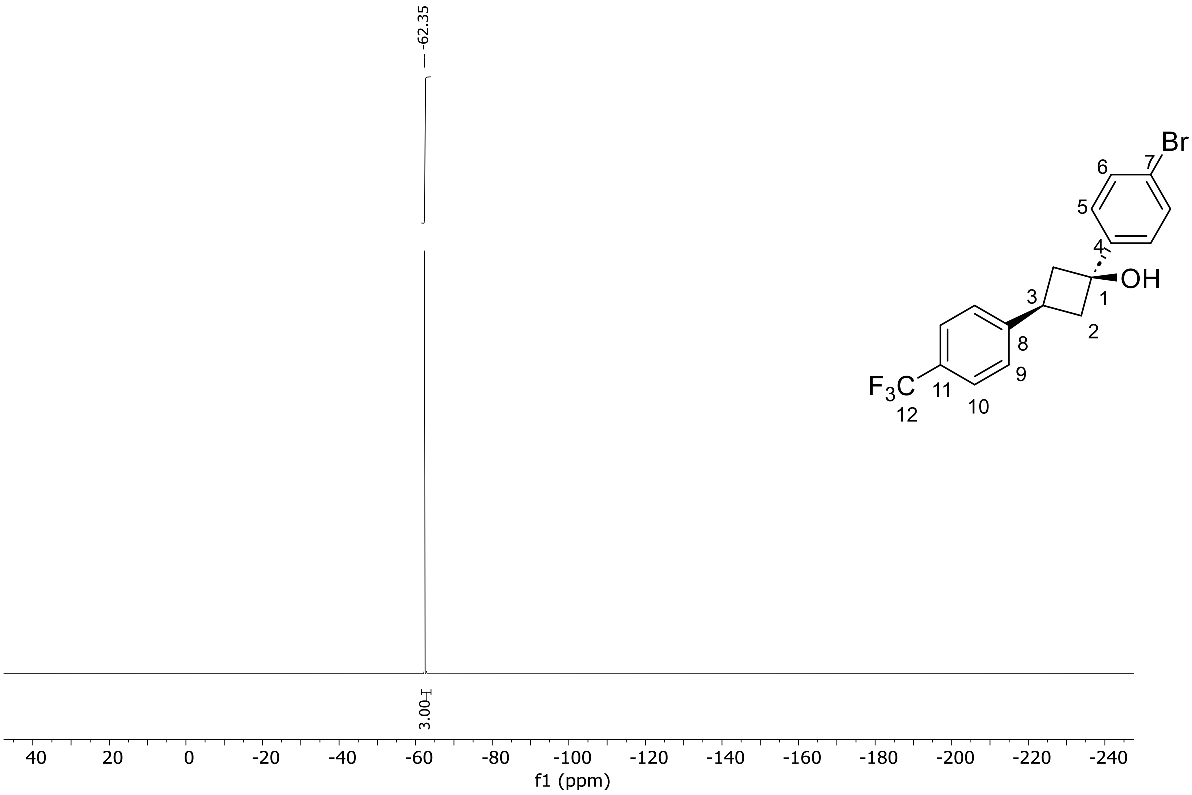


**Supplementary Figure 81.** $^{19}$F NMR of **1v** (470 MHz, 299 K, $CDCl_3$).

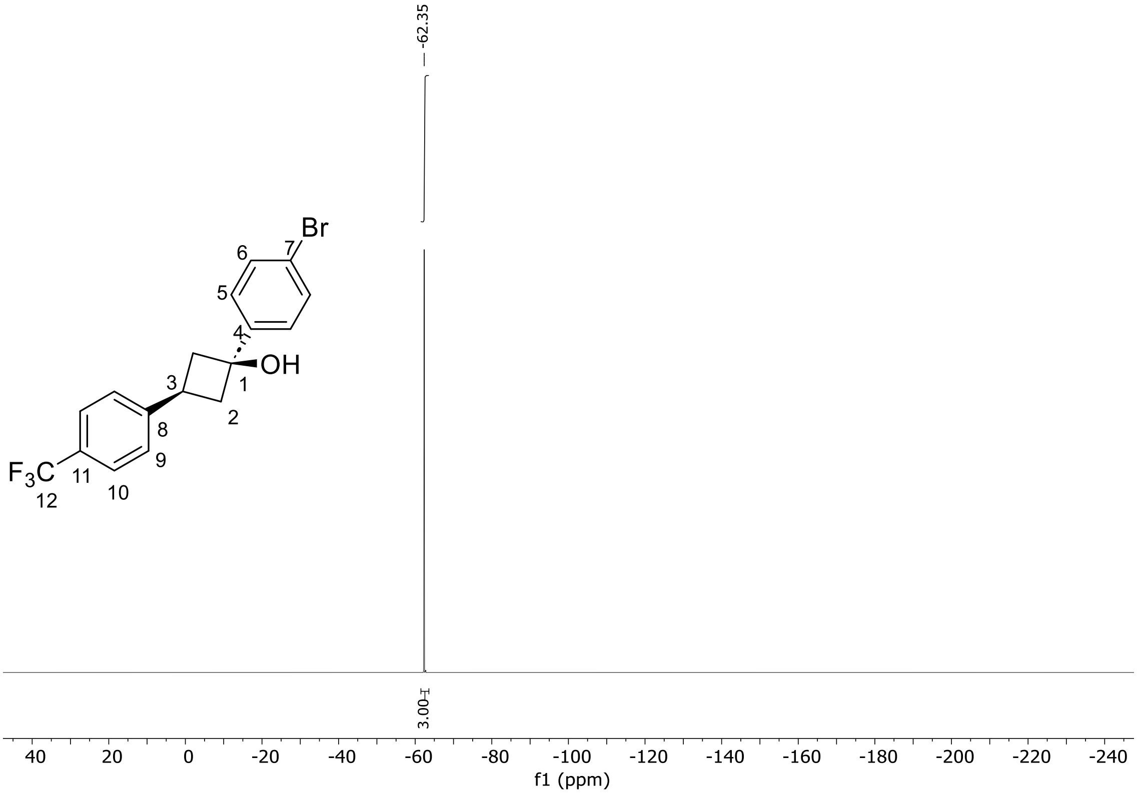


**Supplementary Figure 82.** $^{19}$F{$^1$H} NMR of **1v** (470 MHz, 299 K, $CDCl_3$).

## 4-(3-(4-Bromophenyl)-3-hydroxycyclobutyl)phenyl trifluoromethanesulfonate (1w)

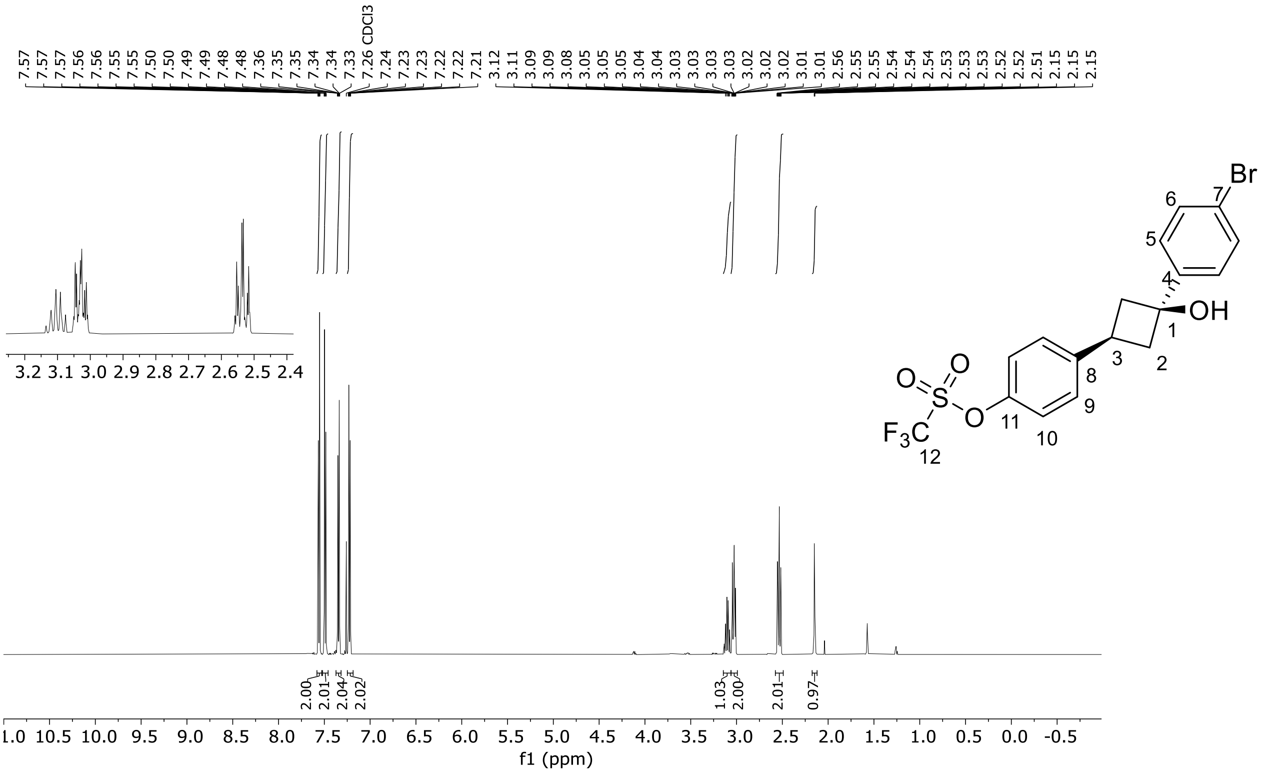


**Supplementary Figure 83.** $^{1}H$ NMR of **1w** (599 MHz, 299 K, $CDCl_3$).

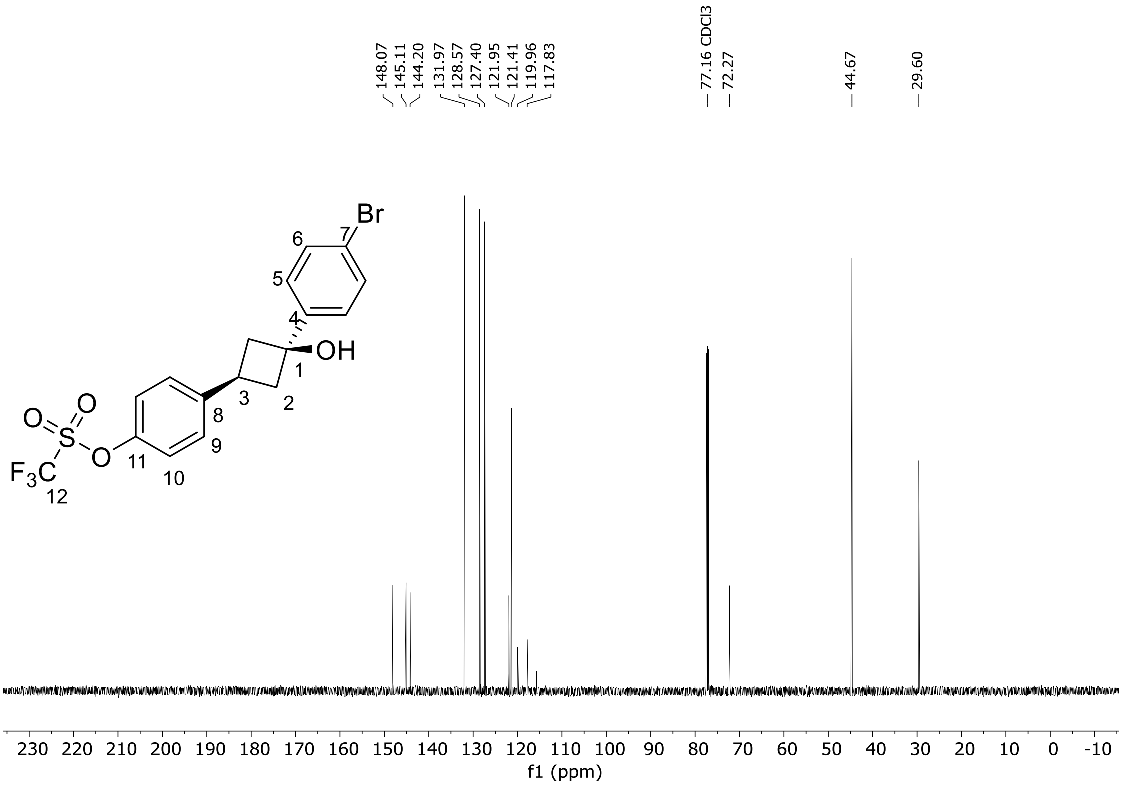


**Supplementary Figure 84.** $^{13}C\{^{1}H\}$ NMR of **1w** (151 MHz, 299 K, $CDCl_3$).

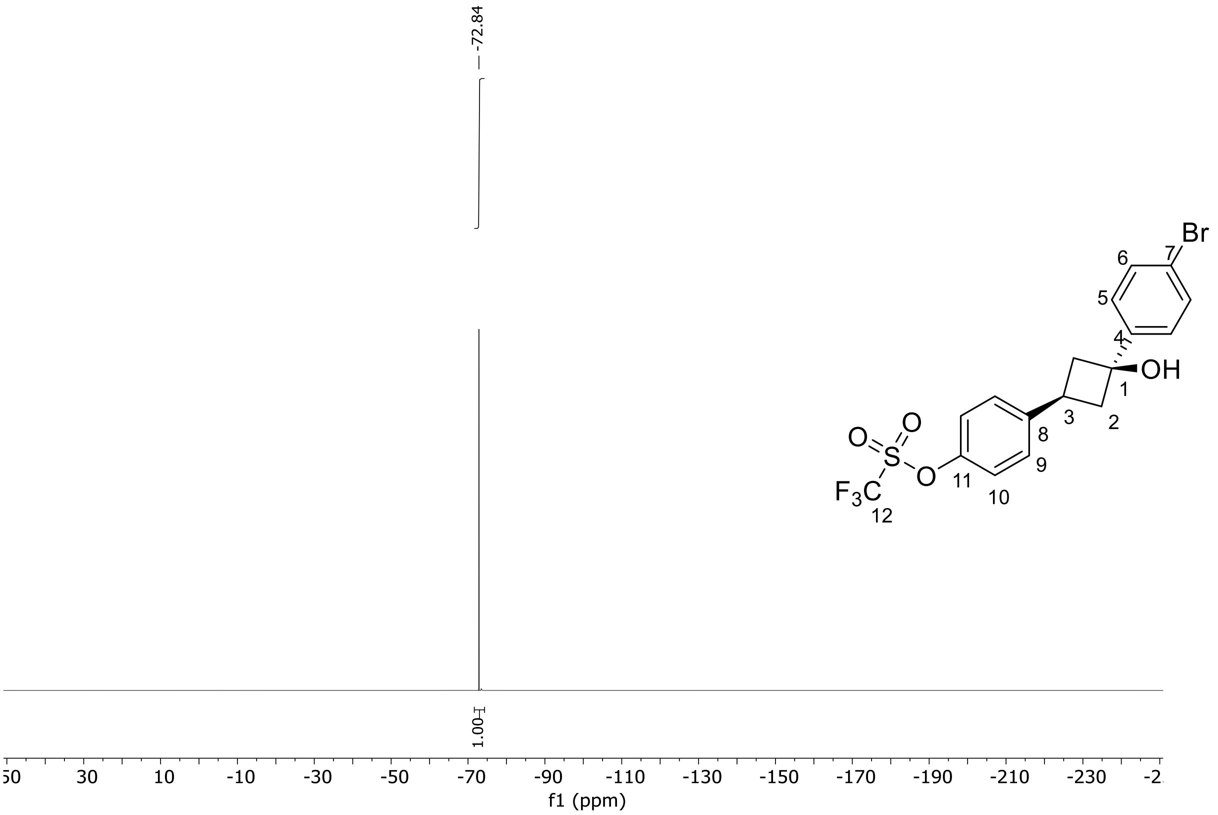


**Supplementary Figure 85.** $^{19}$F NMR of **1w** (376 MHz, 299 K, $CDCl_3$).

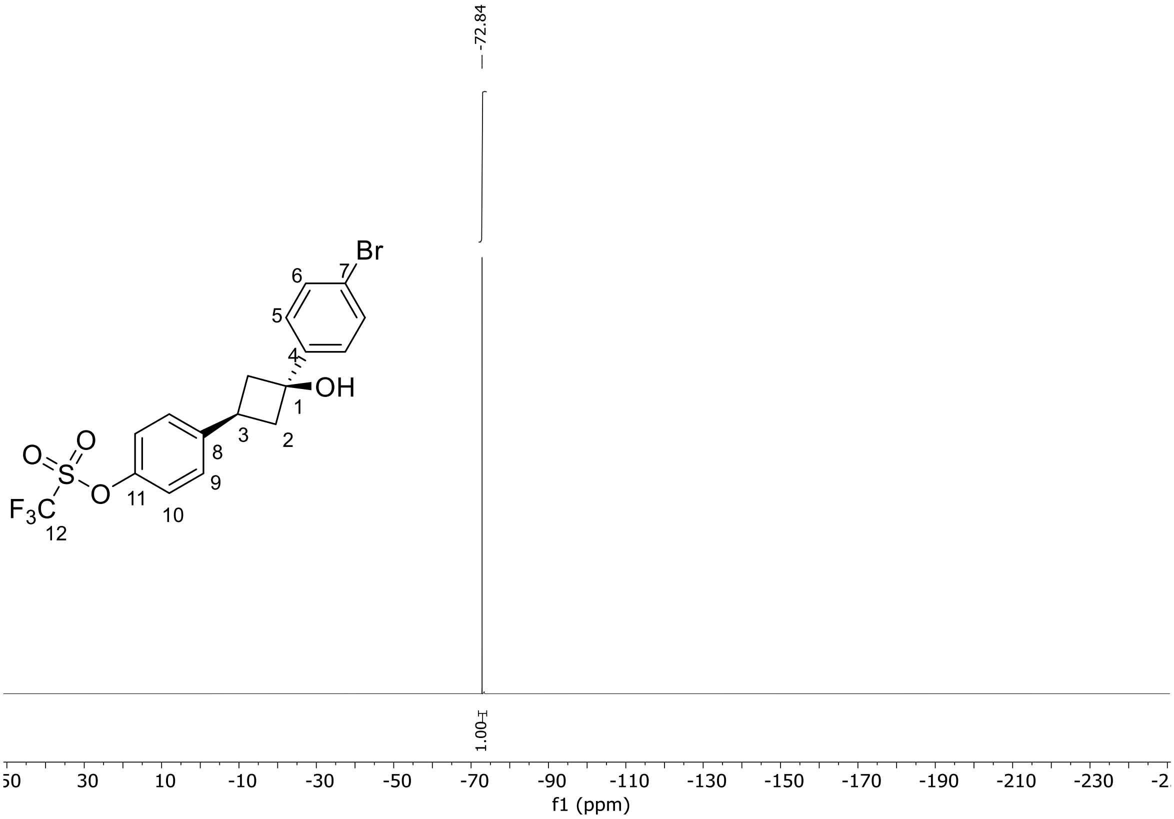


**Supplementary Figure 86.** $^{19}$F{$^1$H} NMR of **1w** (376 MHz, 299 K, $CDCl_3$).

## 1-(4-Bromophenyl)-3-(4-chlorophenyl)-3-methylcyclobutan-1-ol (1x)

*cis*-**1x**

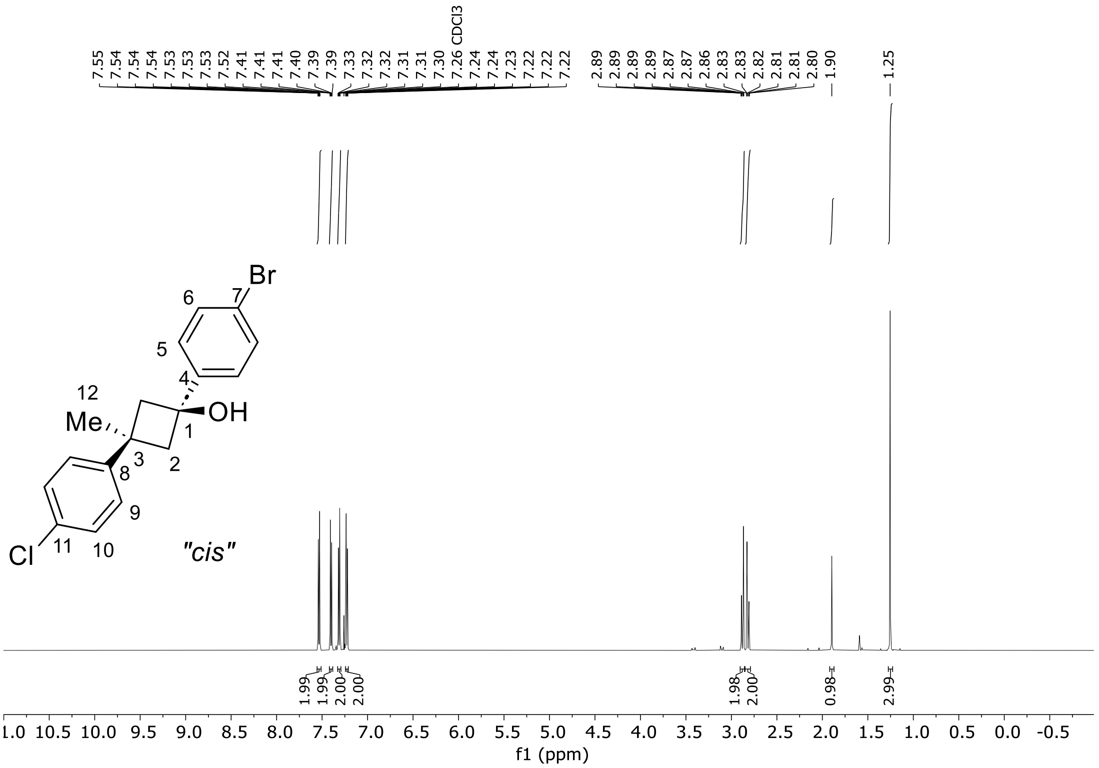


**Supplementary Figure 87.** $^{1}$H NMR of *cis*-**1x** (599 MHz, 299 K, $CDCl_3$).

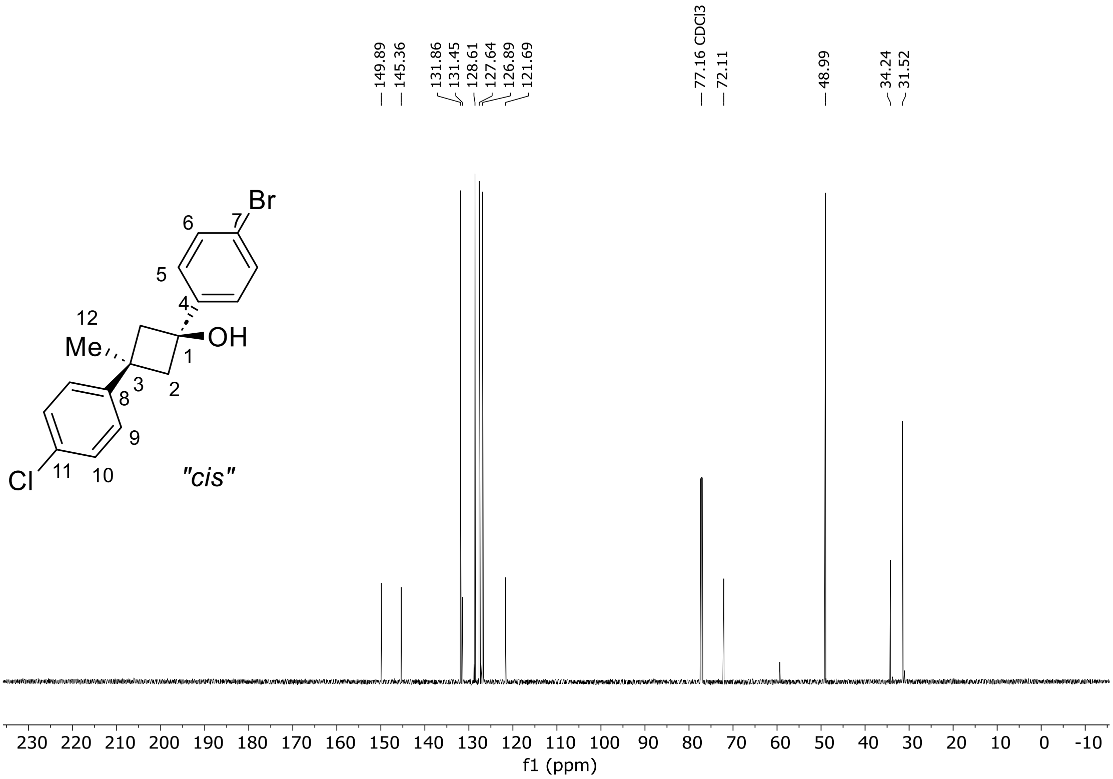


**Supplementary Figure 88.** $^{13}$C{$^{1}$H} NMR of *cis*-**1x** (151 MHz, 299 K, $CDCl_3$).

*trans*-**1x**

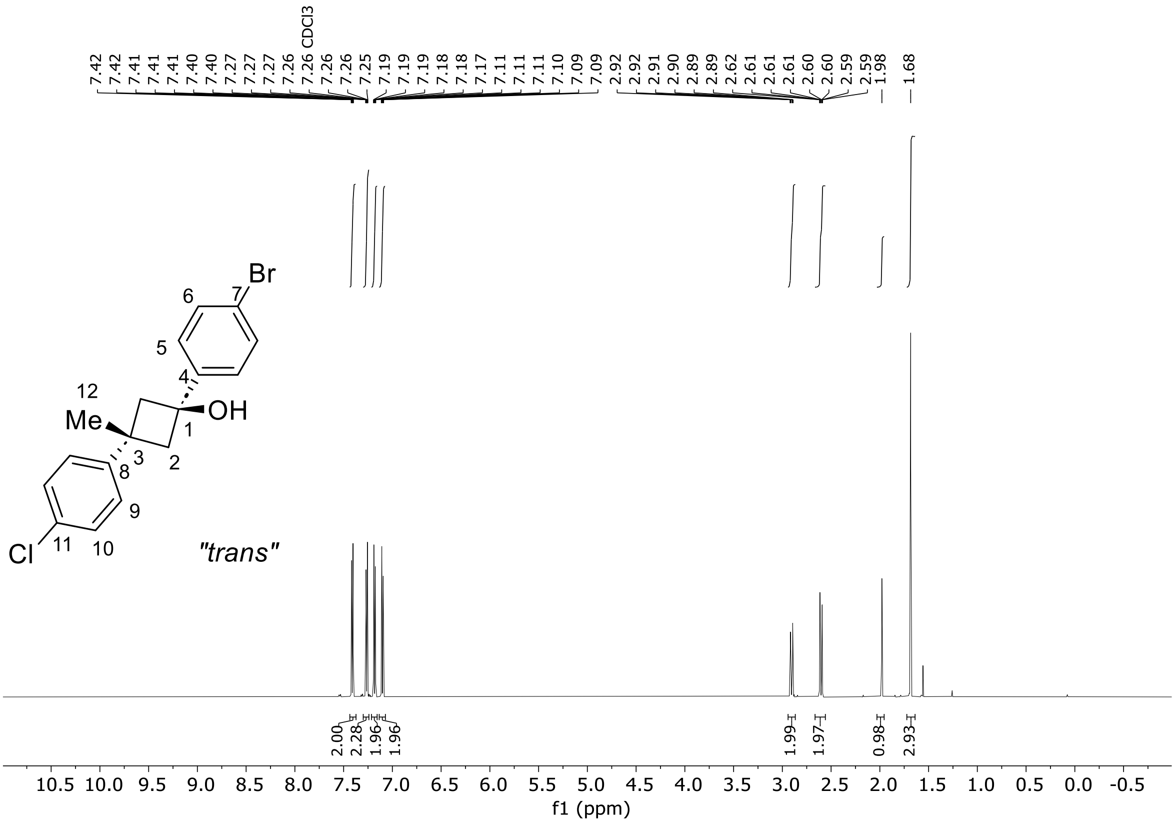


**Supplementary Figure 89.** $^{1}$H NMR of *trans*-**1x** (599 MHz, 299 K, $CDCl_3$).

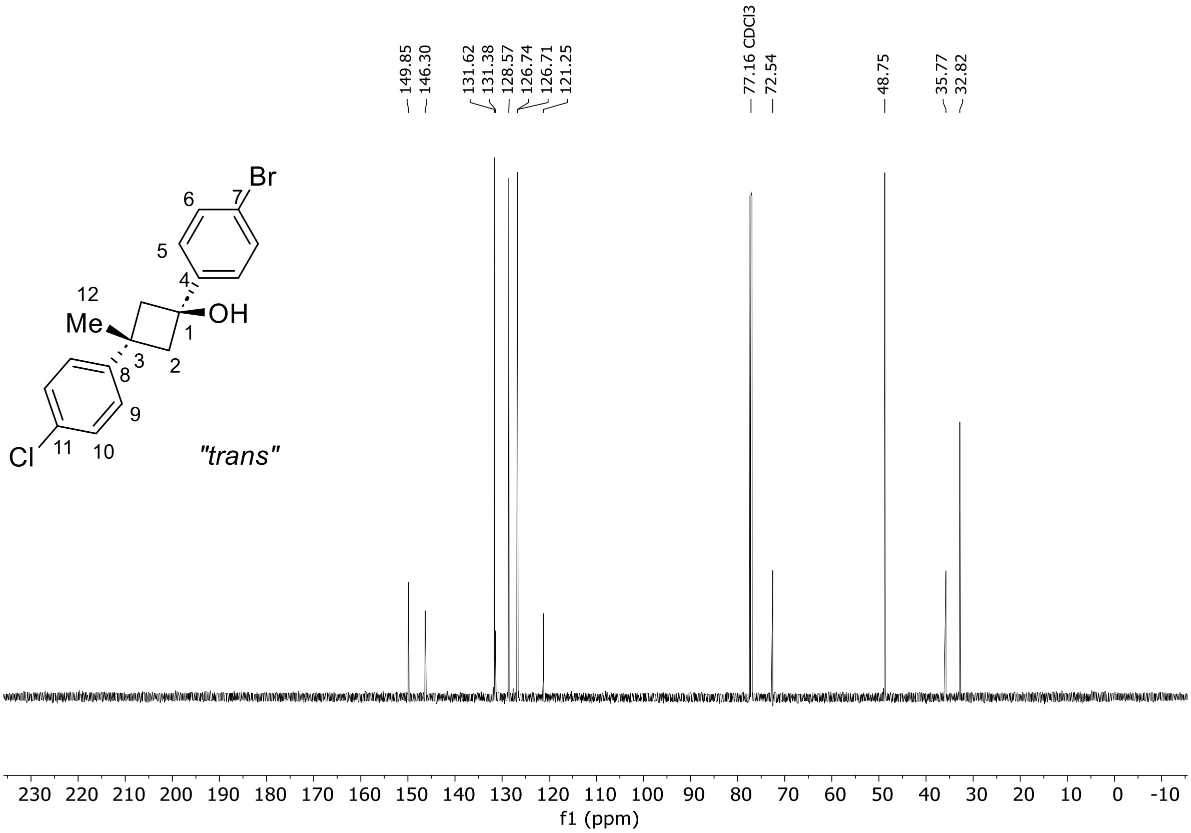


**Supplementary Figure 90.** $^{13}C\{^{1}H\}$ NMR of *trans*-**1x** (151 MHz, 299 K, $CDCl_3$).

**3-Phenyl-1-(4-(pyridin-3-yl)phenyl)cyclobutanol (1y)**

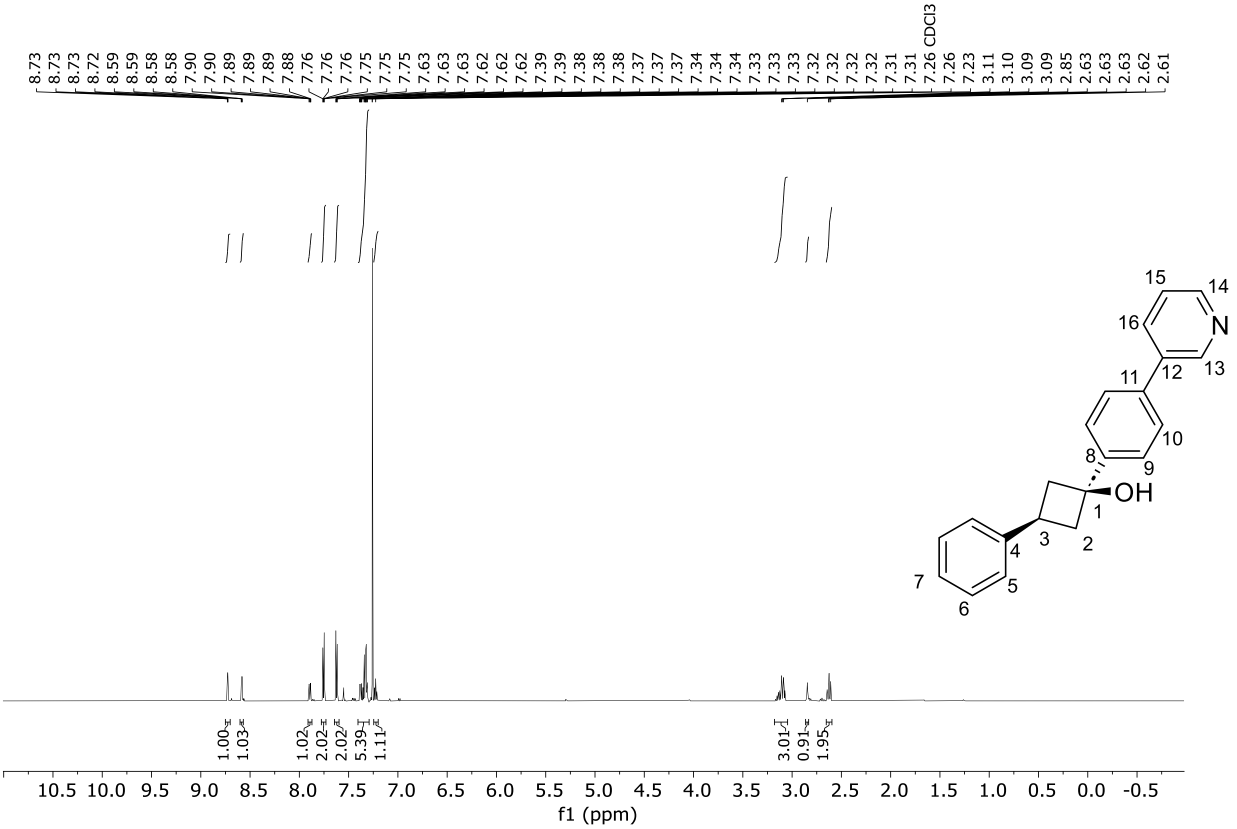


**Supplementary Figure 91**. $^{1}$H NMR of **1y** (500 MHz, 299 K, $CDCl_3$).

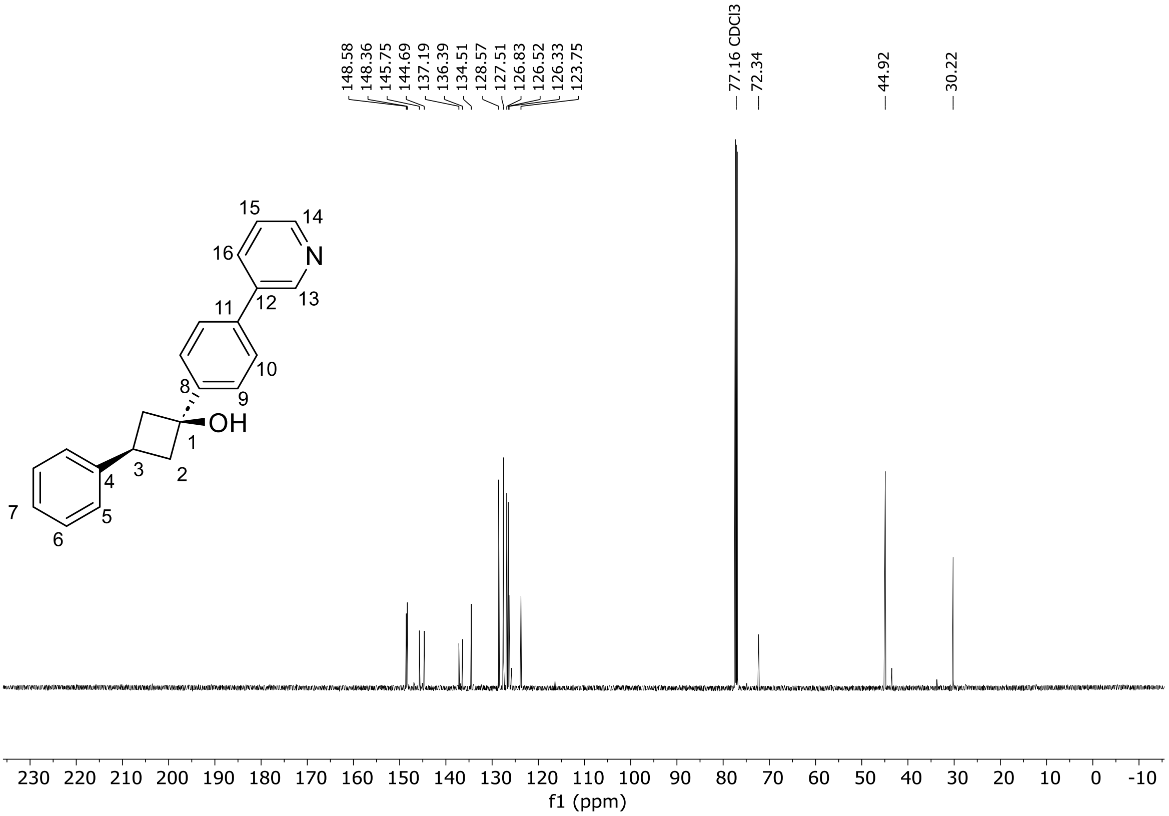


**Supplementary Figure 92.** $^{13}C\{^{1}H\}$ NMR of **1y** (126 MHz, 299 K, $CDCl_3$).

**(1,3,3-Trifluorobutane-1,4-diyl)dibenzene (2a)**

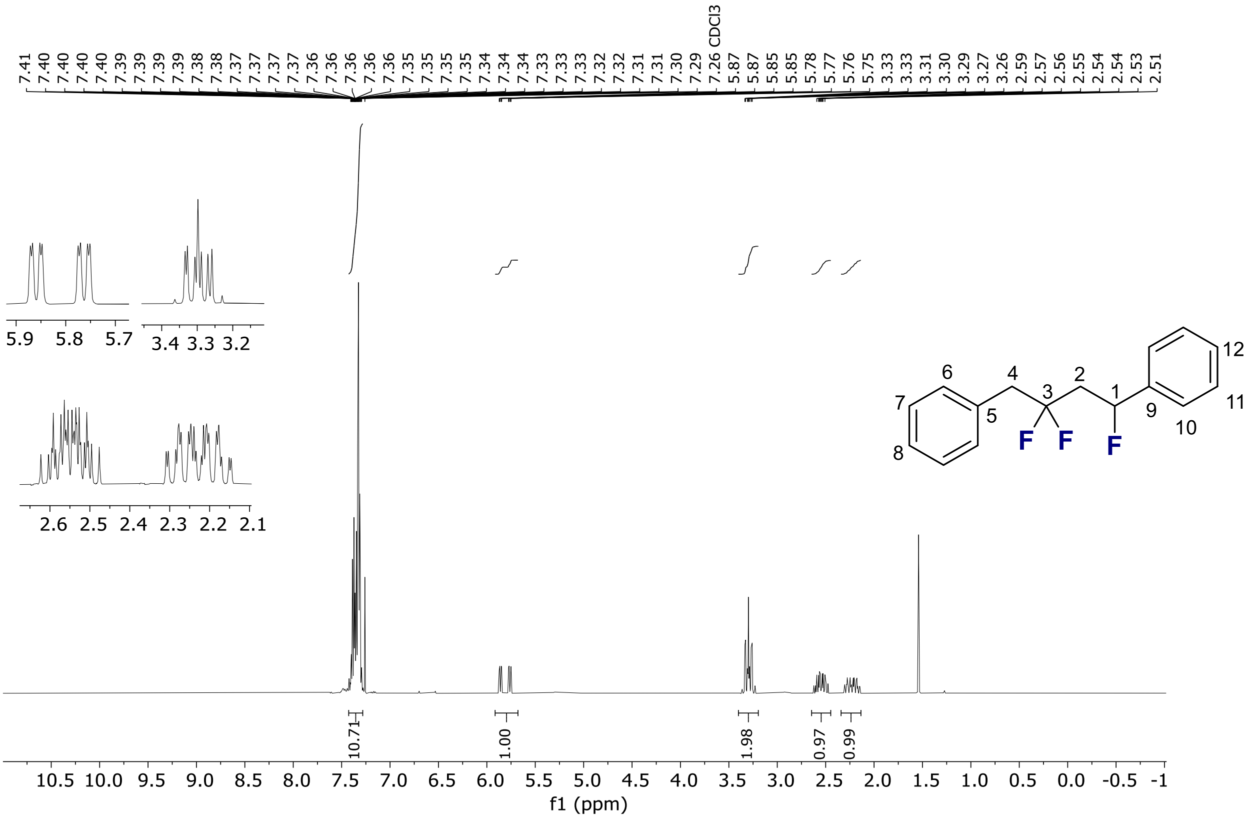


**Supplementary Figure 93.** $^{1}$H NMR of **2a** (500 MHz, 299 K, $CDCl_3$).

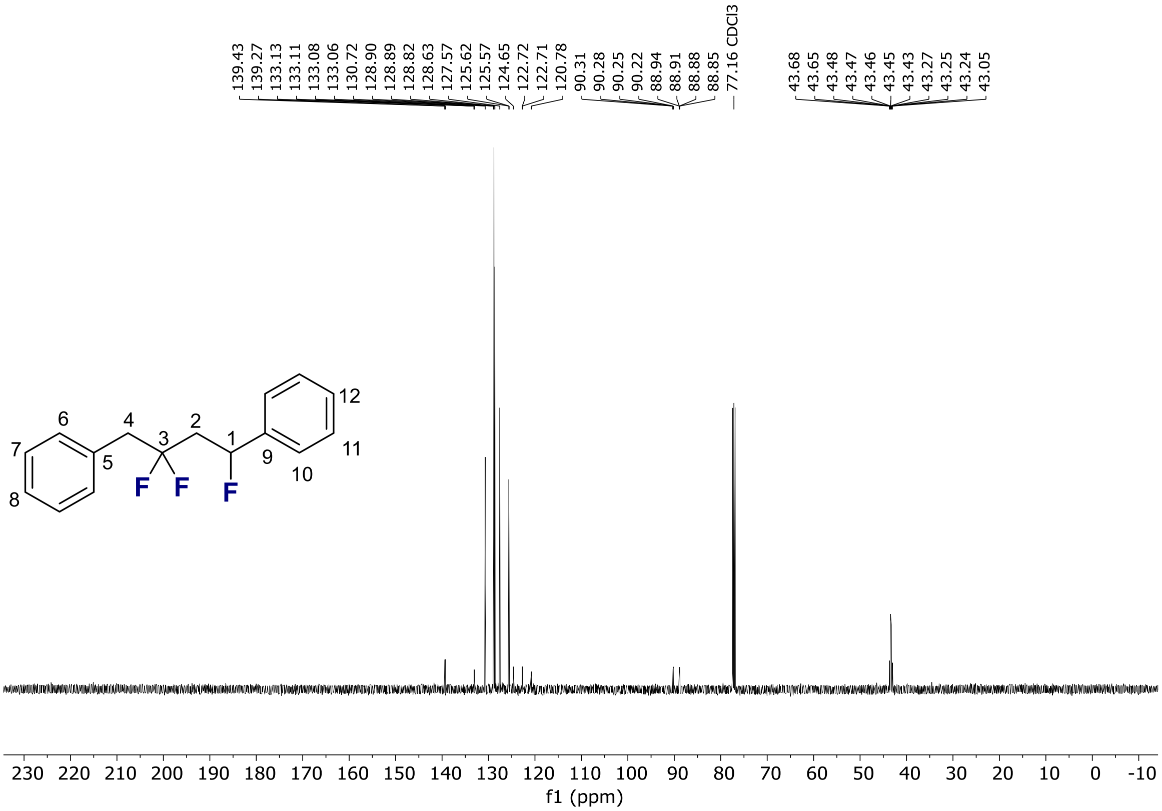


**Supplementary Figure 94.** $^{13}$C{$^{1}$H} NMR of **2a** (126 MHz, 299 K, $CDCl_3$).

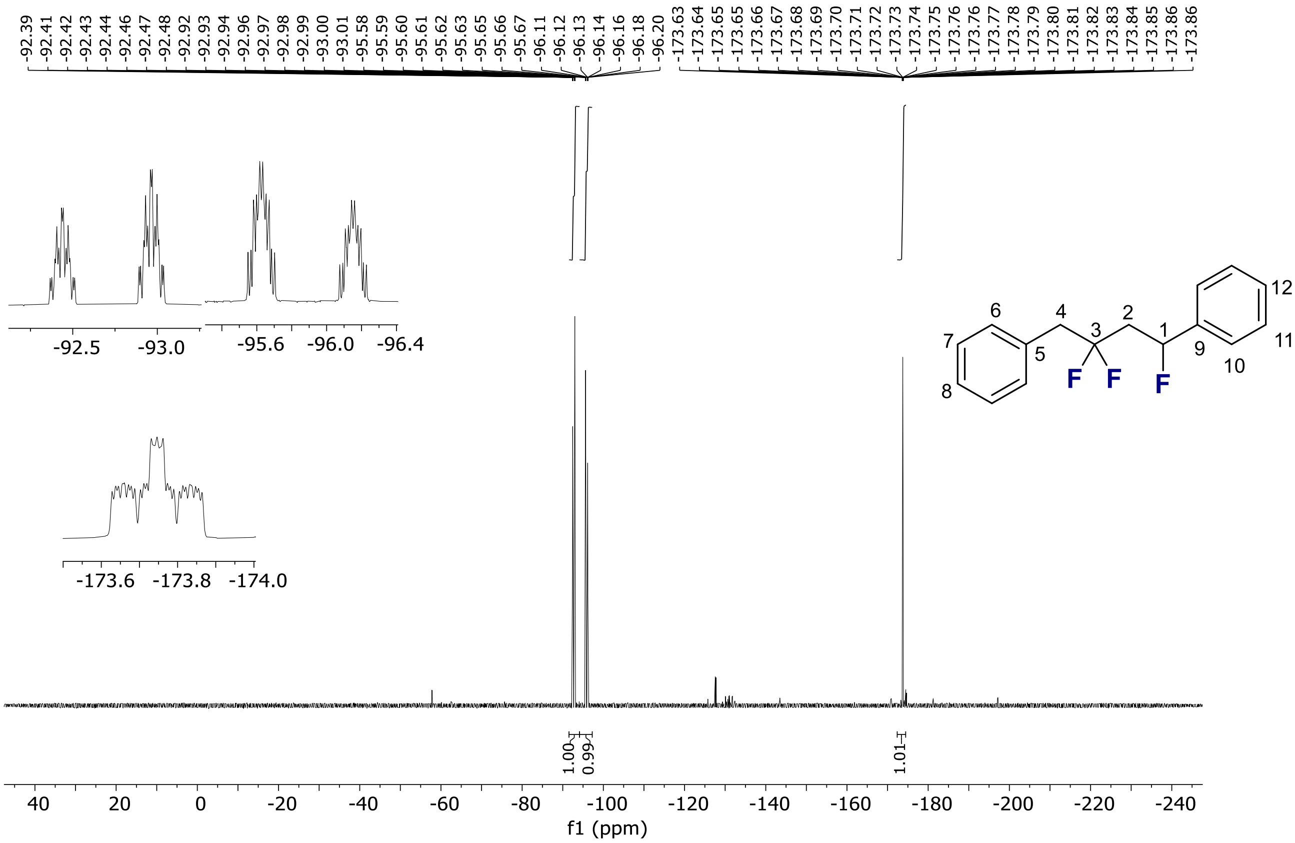

**Supplementary Figure 95.** $^{19}$F NMR of **2a** (470 MHz, 299 K, $CDCl_3$).

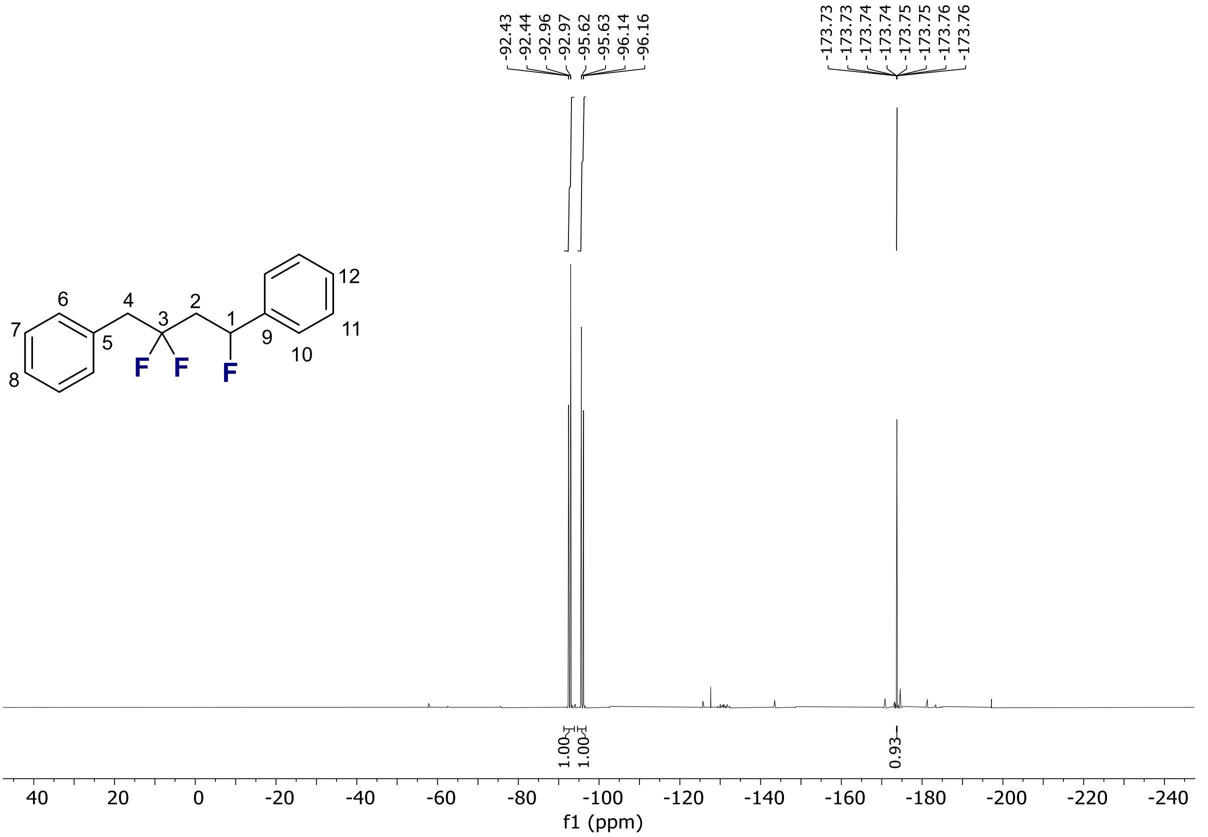

**Supplementary Figure 96.** $^{19}$F{$^1$H} NMR of **2a** (470 MHz, 299 K, $CDCl_3$).

## 4,4'-(1,3,3-Trifluorobutane-1,4-diyl)bis(fluorobenzene) (2b)

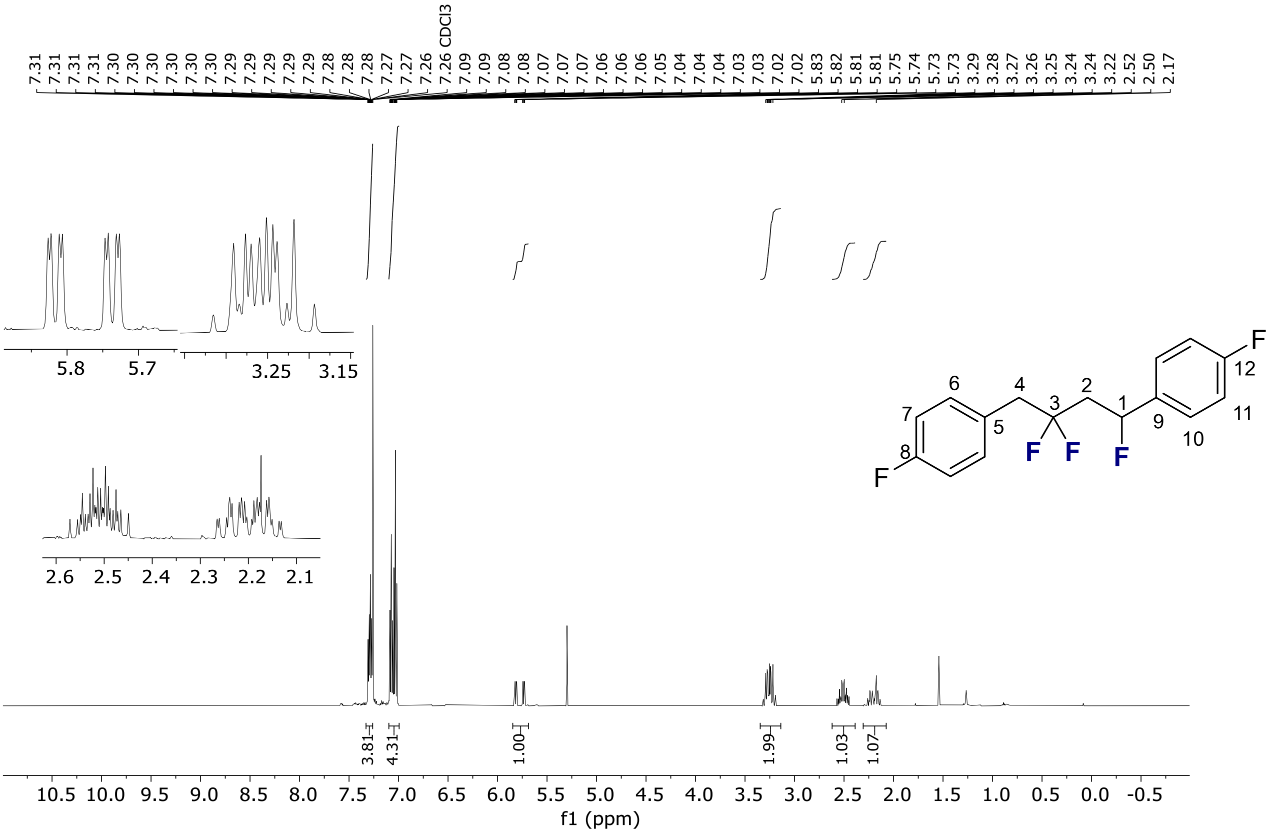


**Supplementary Figure 97.** $^{1}H$ NMR of **2b** (599 MHz, 299 K, $CDCl_3$).

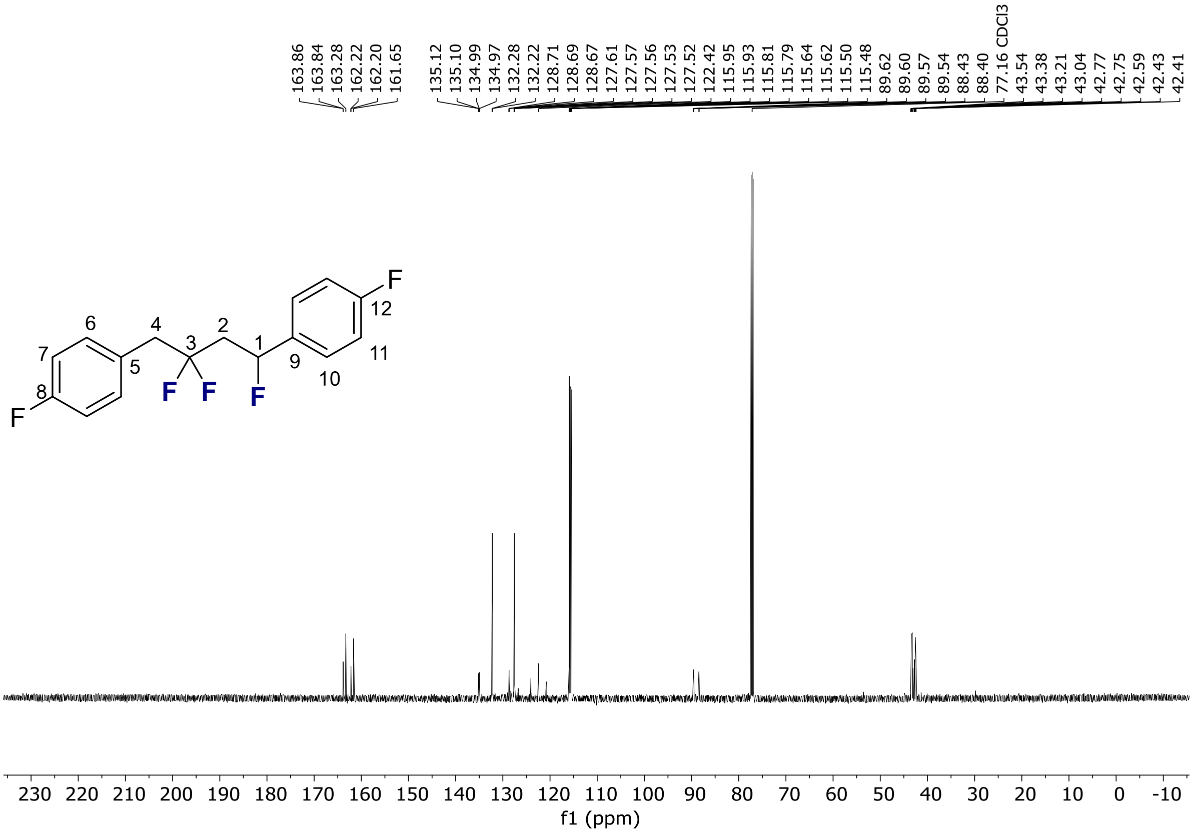


**Supplementary Figure 98.** $^{13}C\{^{1}H\}$ NMR of **2b** (151 MHz, 299 K, $CDCl_3$).

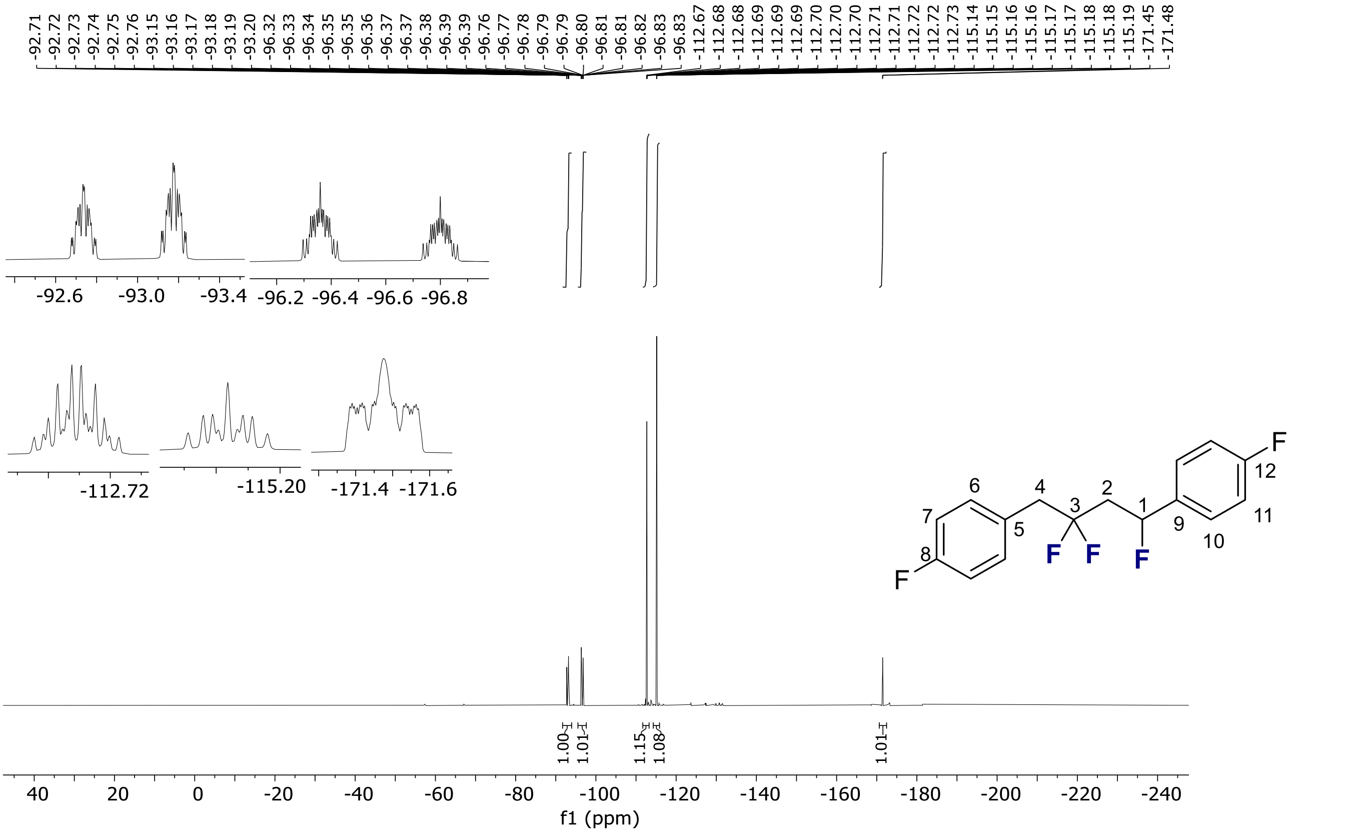

**Supplementary Figure 99.** $^{19}F$ NMR of **2b** (564 MHz, 299 K, $CDCl_3$).

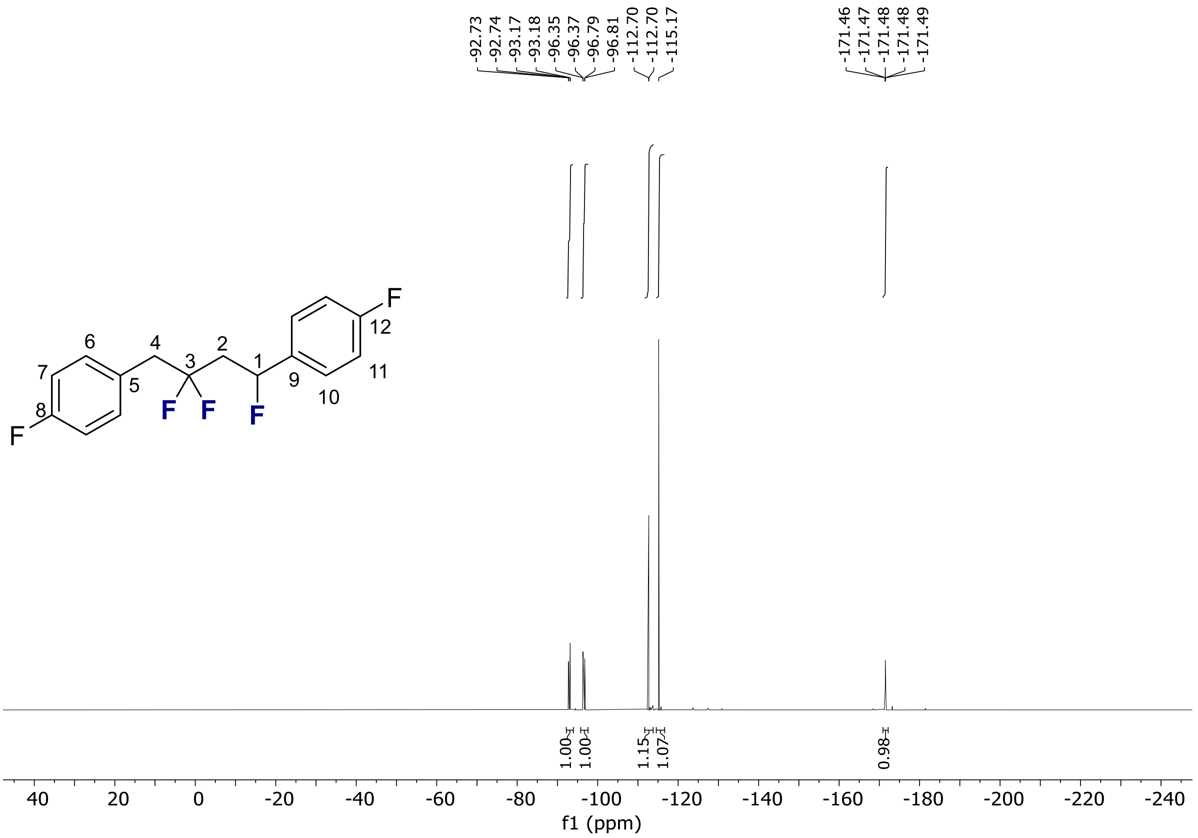

**Supplementary Figure 100.** $^{19}F\{^{1}H\}$ NMR of **2b** (564 MHz, 299 K, $CDCl_3$).

## 4,4'-(1,3,3-Trifluorobutane-1,4-diyl)bis(chlorobenzene) (2c)

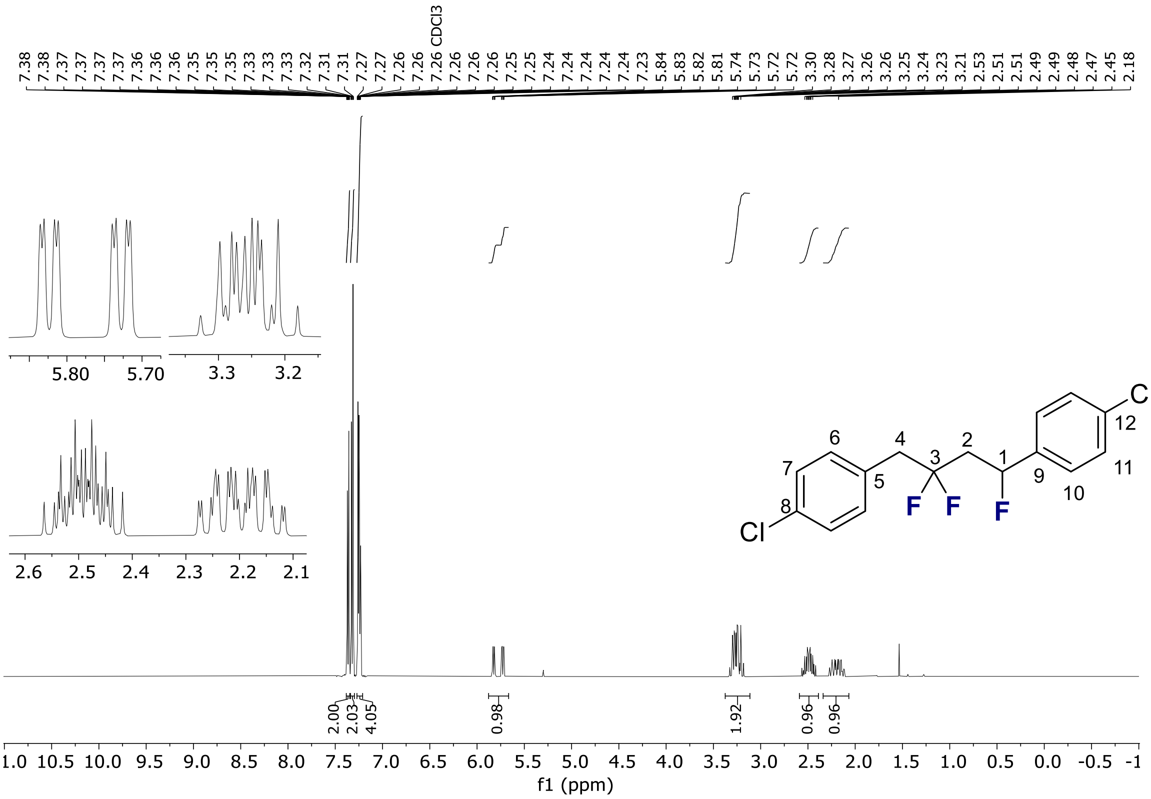


**Supplementary Figure 101.** $^{1}$H NMR of **2c** (500 MHz, 299 K, $CDCl_3$).

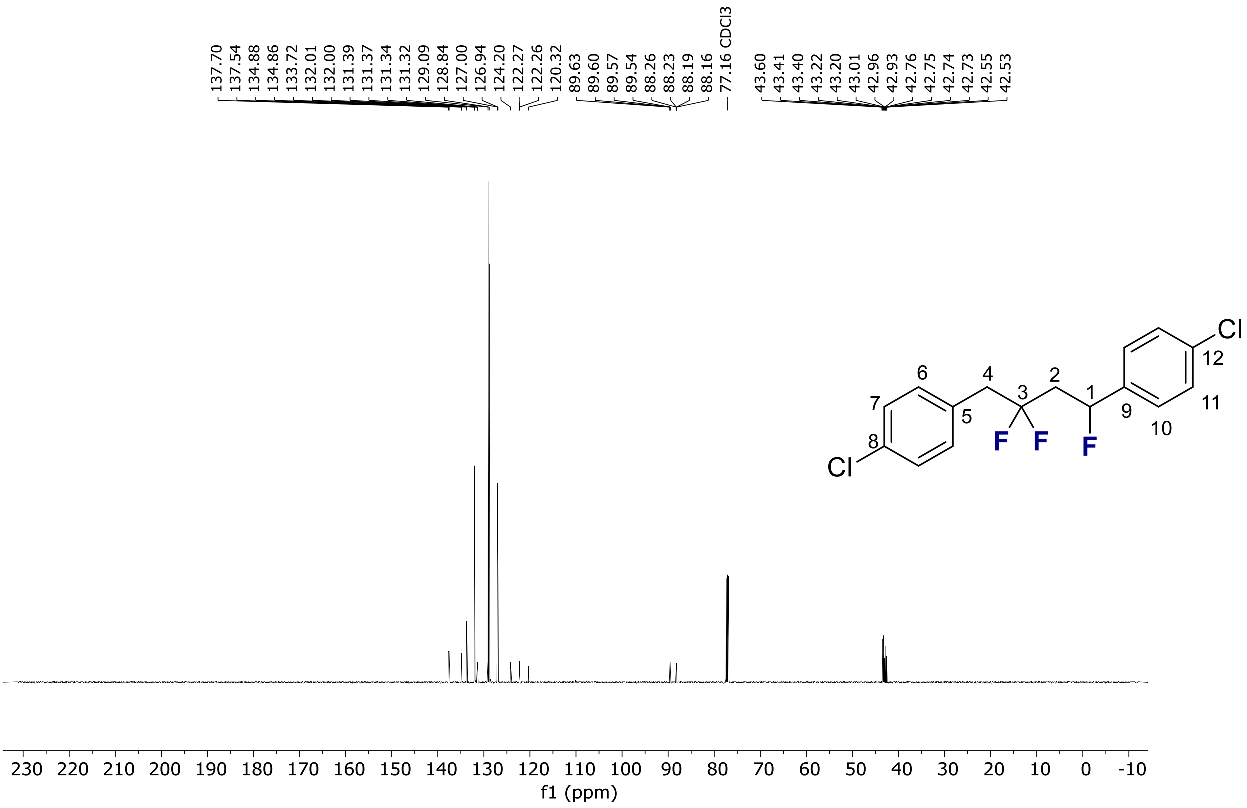


**Supplementary Figure 102.** $^{13}C\{^{1}H\}$ NMR of **2c** (126 MHz, 299 K, $CDCl_3$).

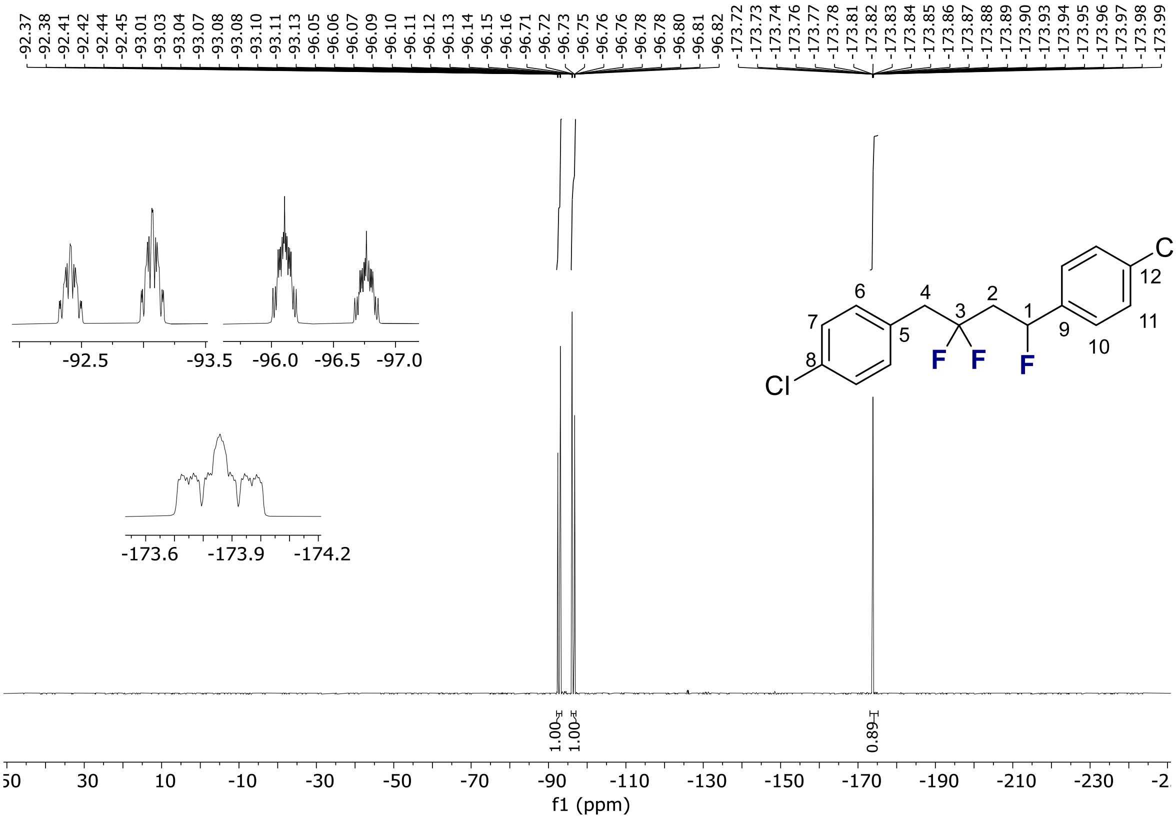

**Supplementary Figure 103.** $^{19}$F NMR of **2c** (376 MHz, 299 K, $CDCl_3$).

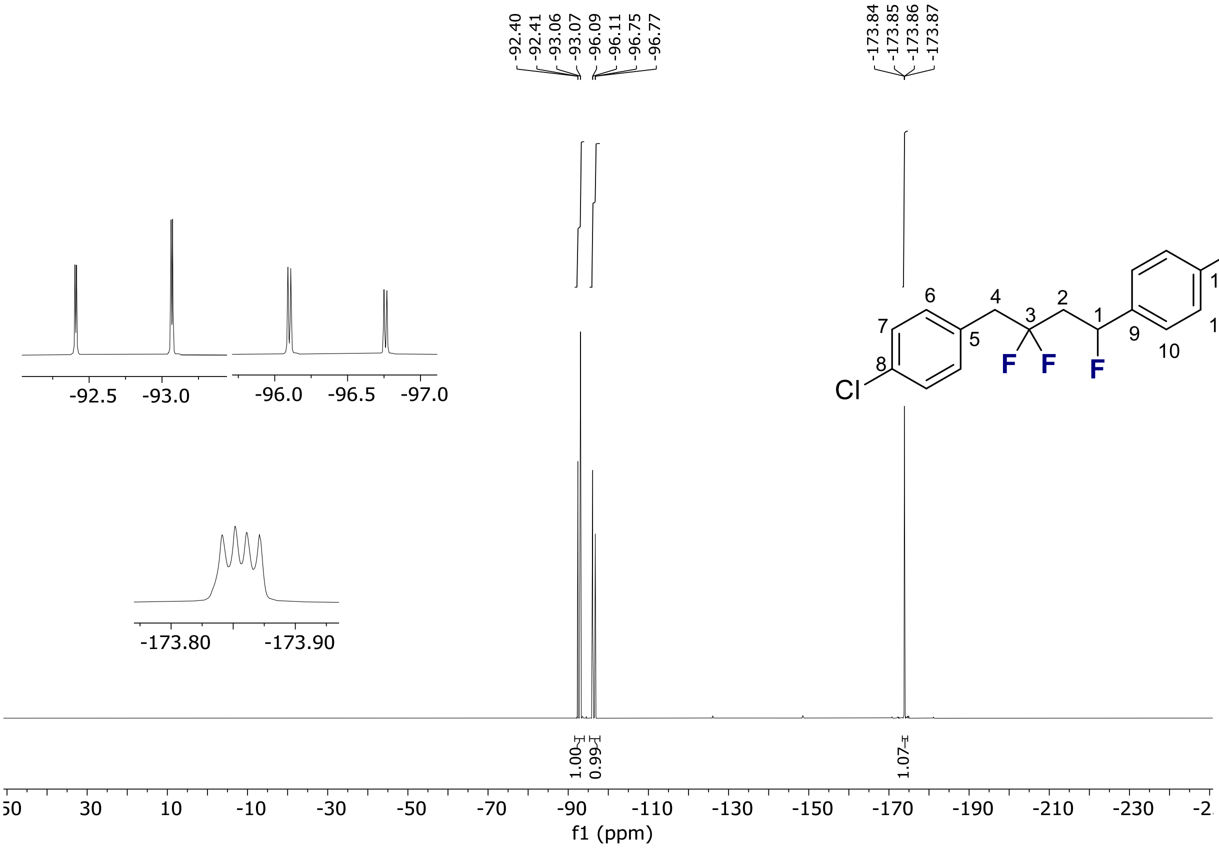

**Supplementary Figure 104.** $^{19}F\{^{1}H\}$ NMR of **2c** (376 MHz, 299 K, $CDCl_3$).

## 4,4'-(1,3,3-Trifluorobutane-1,4-diyl)bis(bromobenzene) (2d)

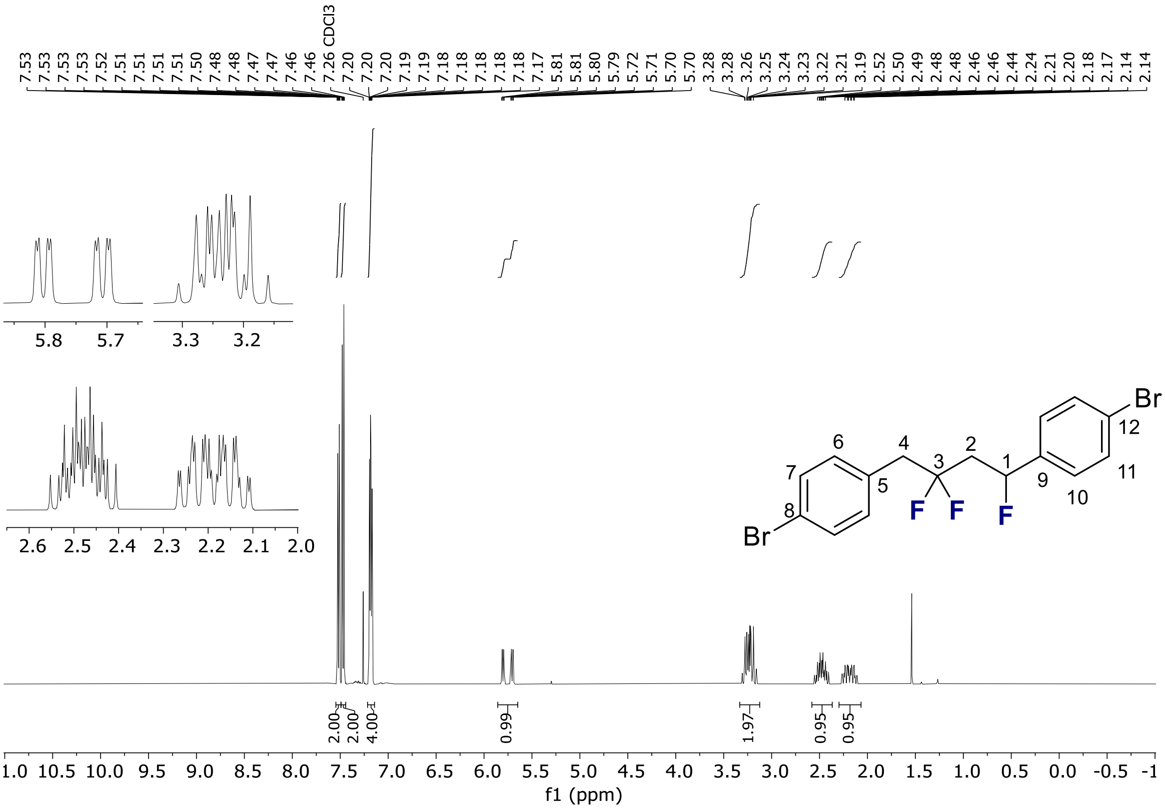


**Supplementary Figure 105.** $^{1}$H NMR of **2d** (500 MHz, 299 K, $CDCl_3$).

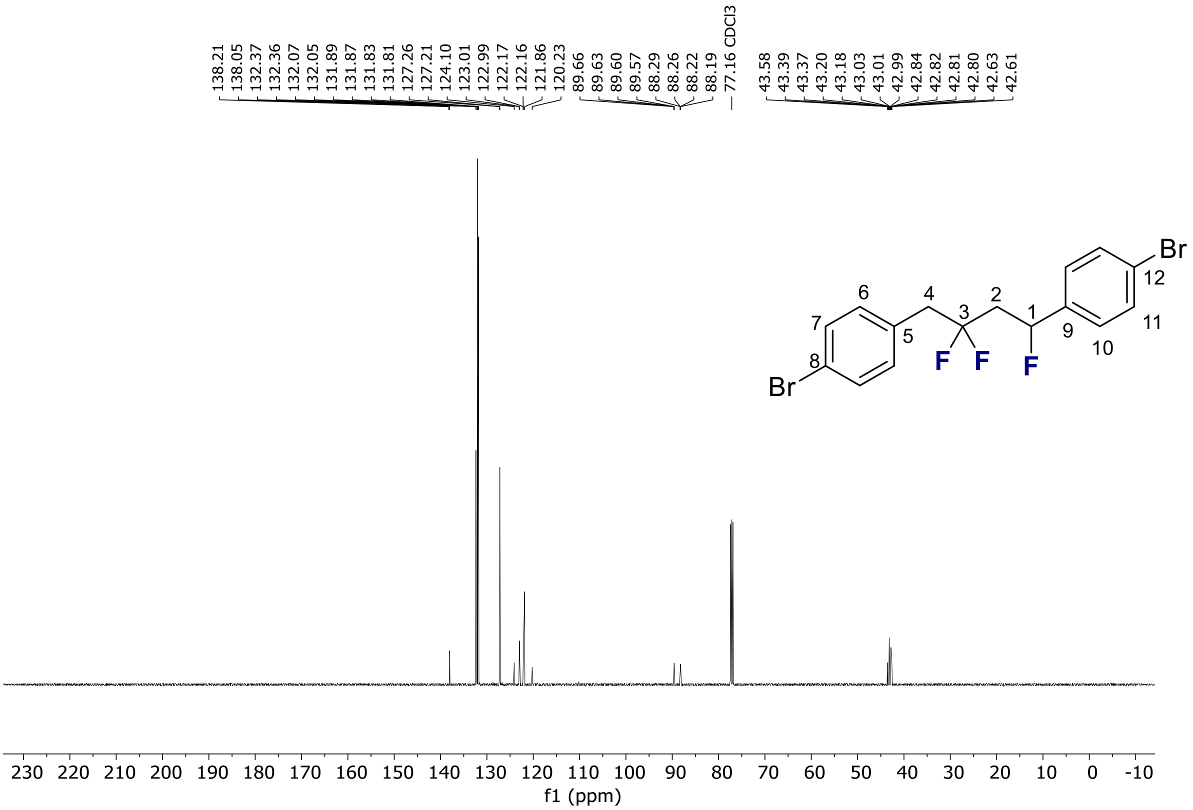


**Supplementary Figure 106.** $^{13}$C{$^{1}$H} NMR of **2d** (126 MHz, 299 K, $CDCl_3$).

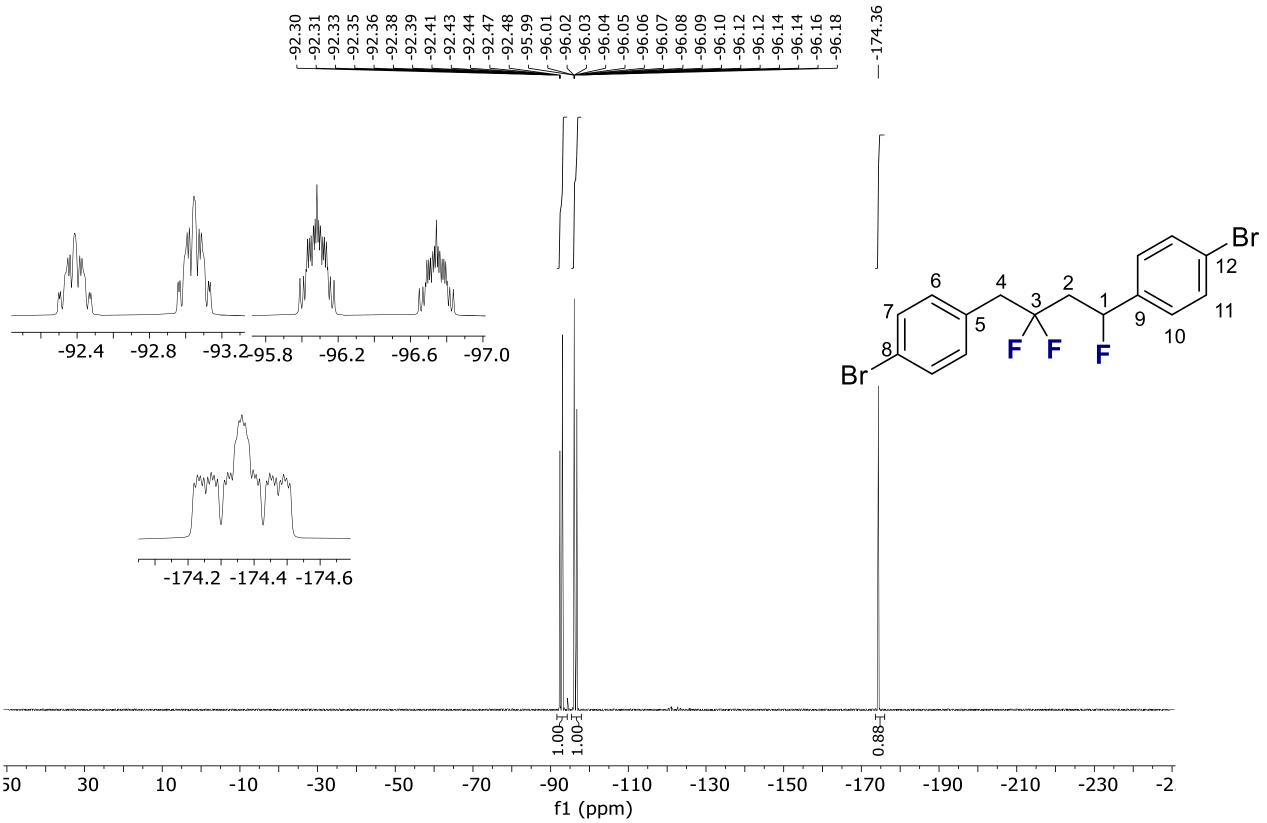


**Supplementary Figure 107.** $^{19}$F NMR of **2d** (376 MHz, 299 K, $CDCl_3$).

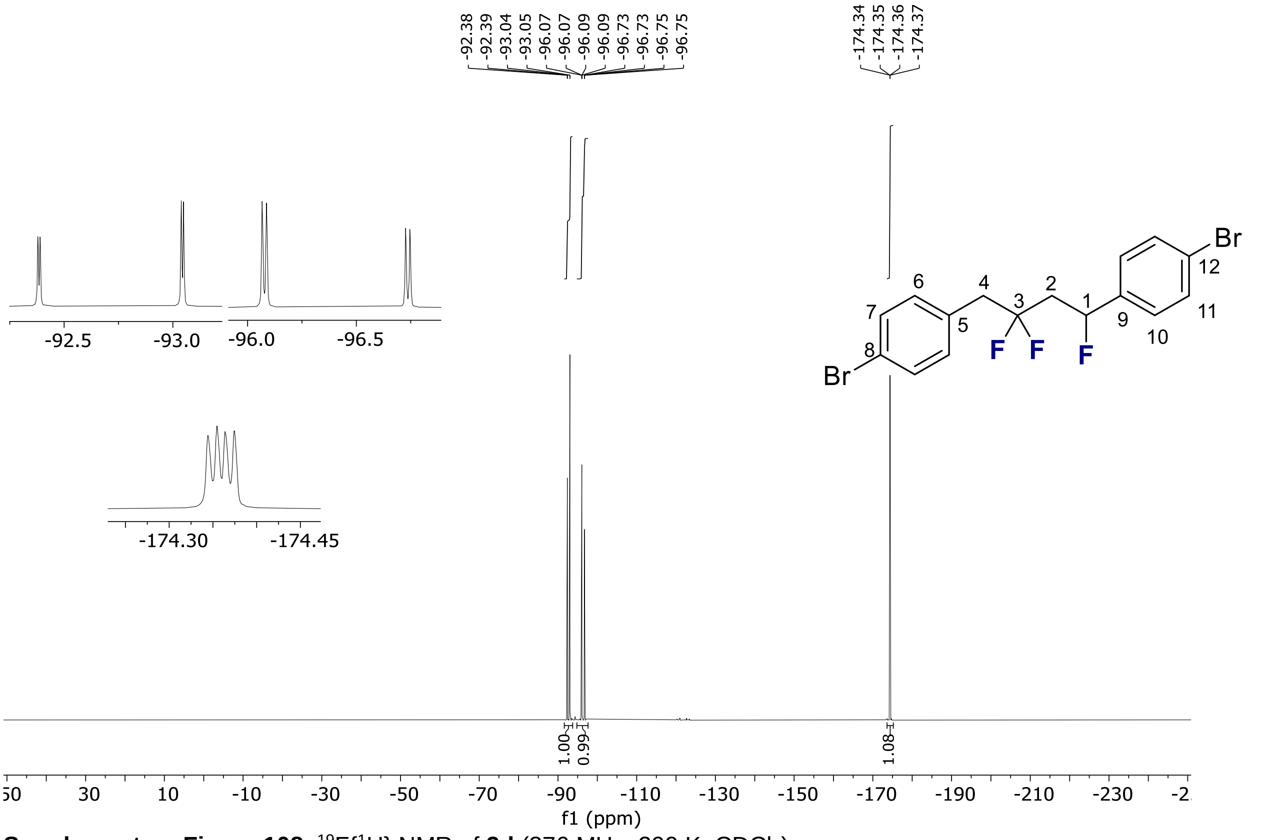


**Supplementary Figure 108.** $^{19}$F{$^{1}$H} NMR of **2d** (376 MHz, 299 K, $CDCl_3$).

## 4,4'-(1,3,3-Trifluorobutane-1,4-diyl)bis((trifluoromethyl)benzene) (2e)

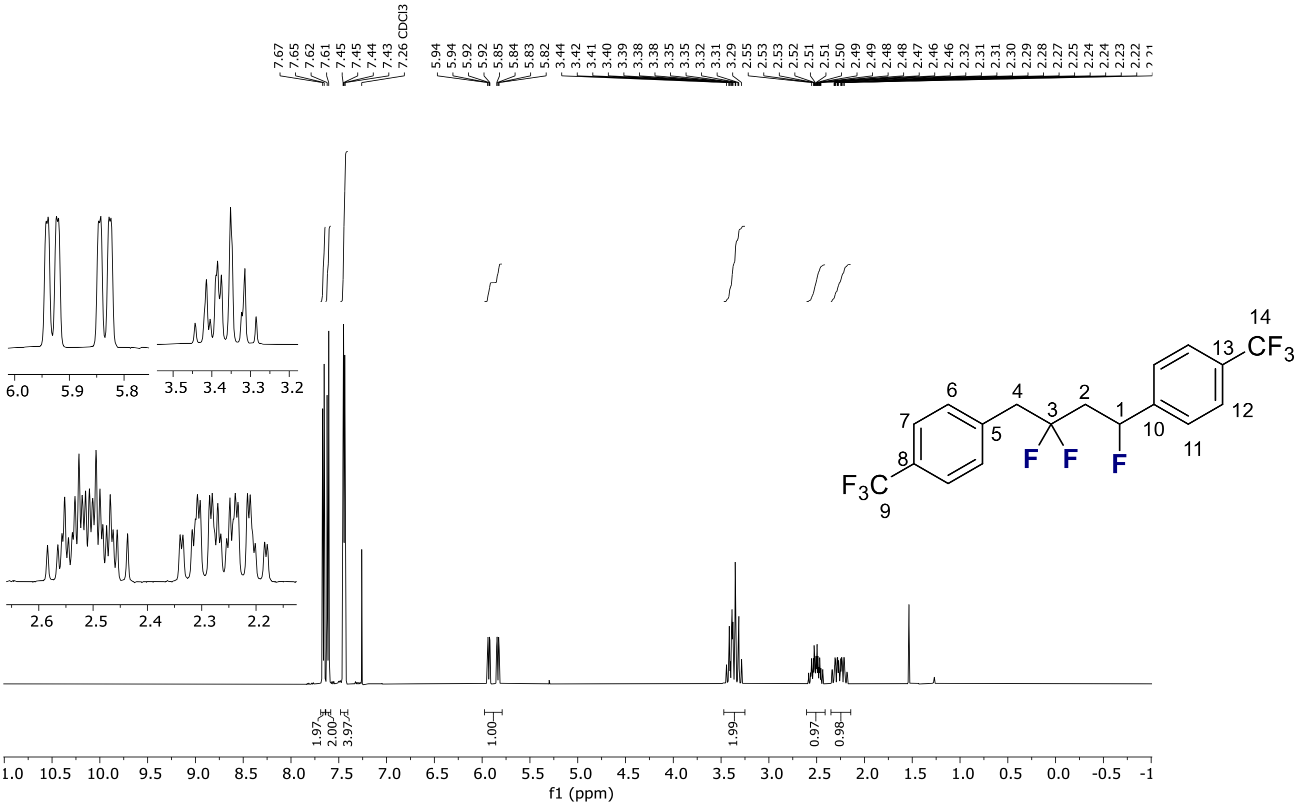


**Supplementary Figure 109.** $^1$H NMR of **2e** (500 MHz, 299 K, $CDCl_3$).

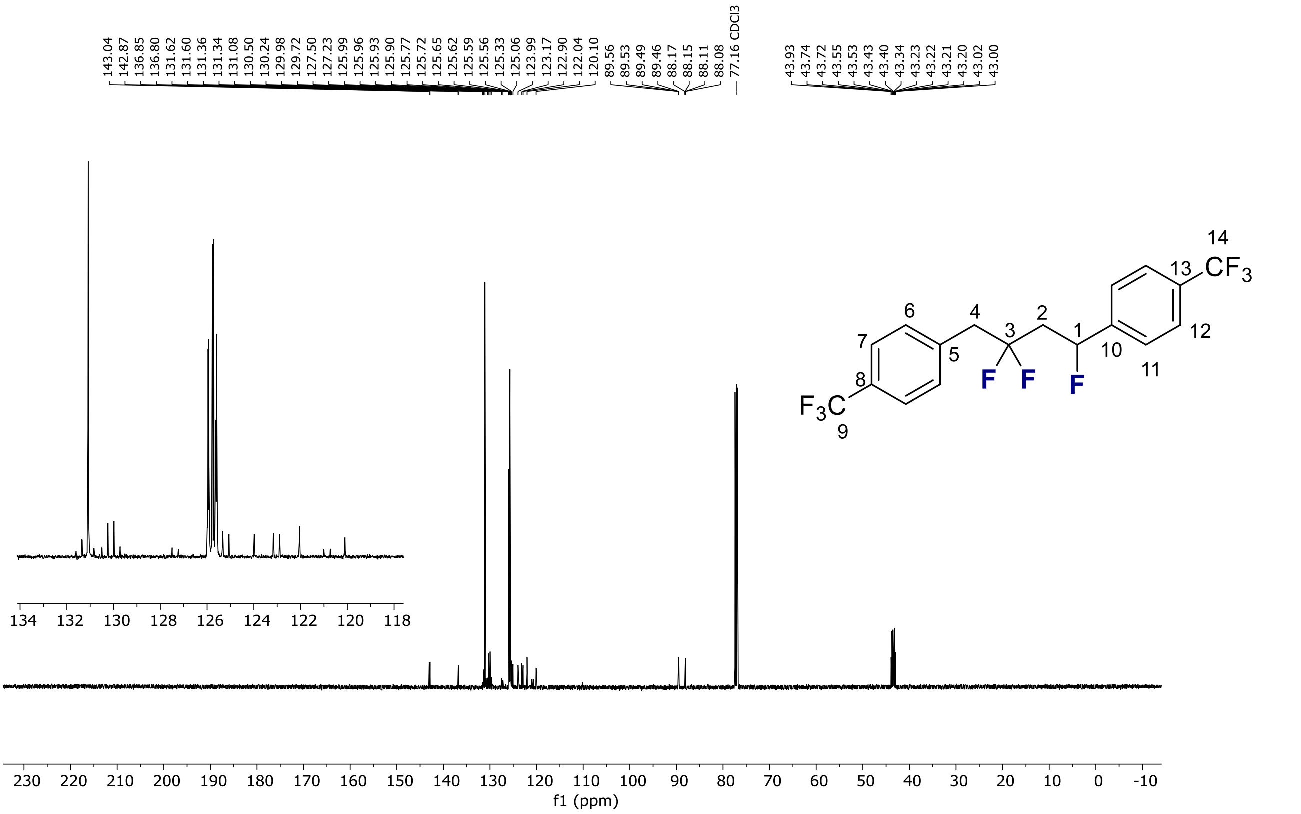


**Supplementary Figure 110.** $^{13}C\{^1H\}$ NMR of **2e** (126 MHz, 299 K, $CDCl_3$).

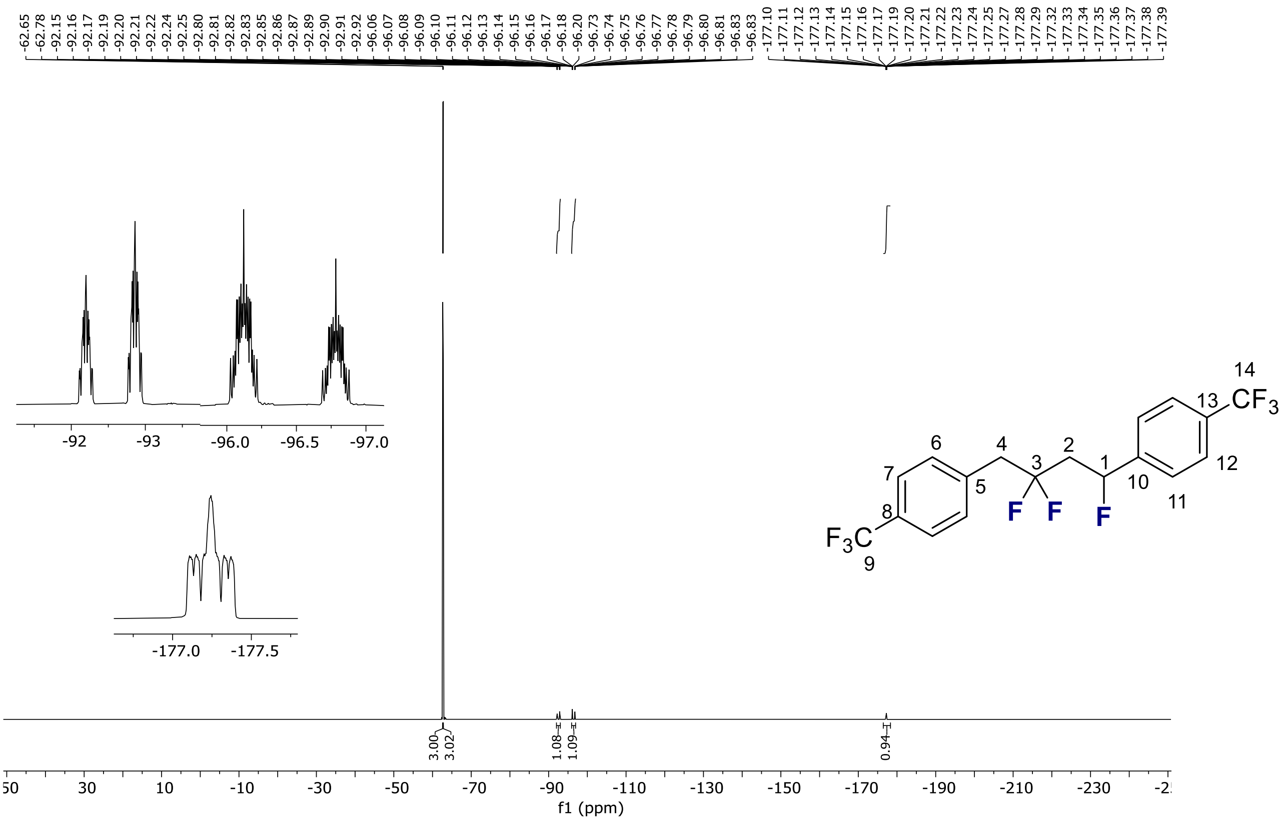


**Supplementary Figure 111.** $^{19}F$ NMR of **2e** (376 MHz, 299 K, $CDCl_3$).

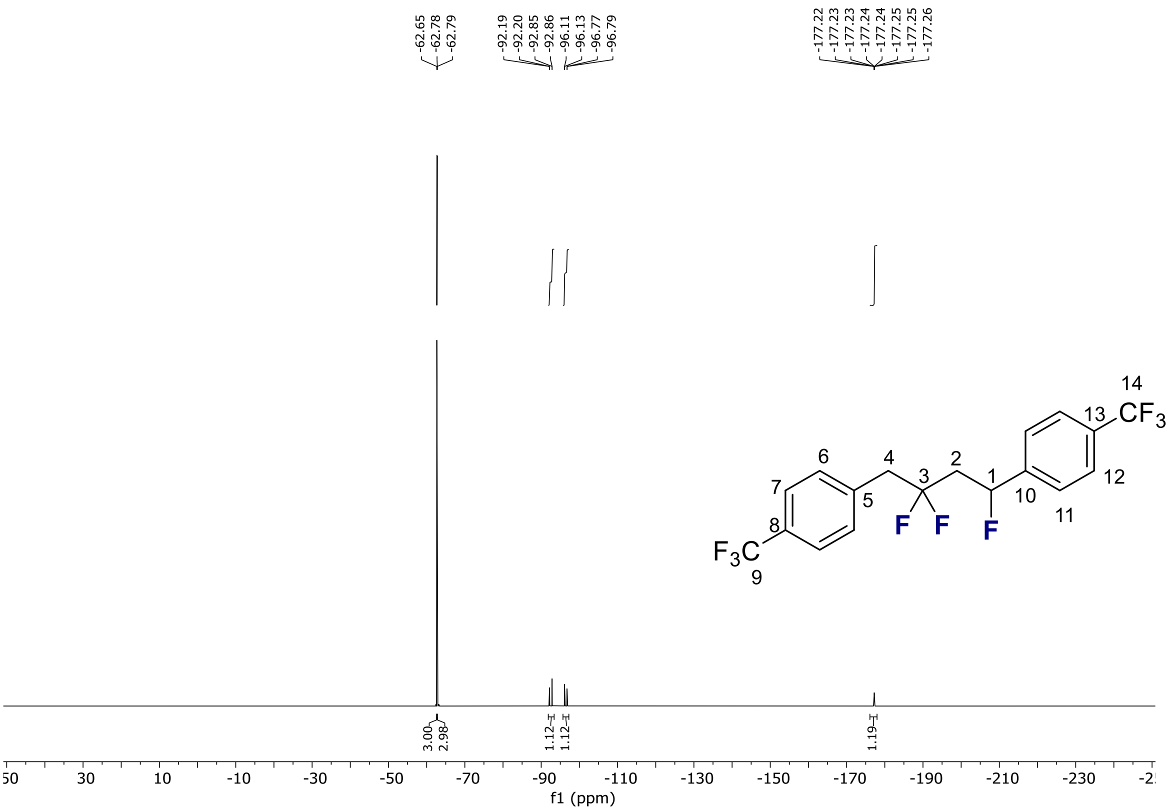


**Supplementary Figure 112.** $^{19}F\{^1H\}$ NMR of **2e** (376 MHz, 299 K, $CDCl_3$).

## 1-Fluoro-4-(2,2,4-trifluoro-4-phenylbutyl)benzene (2f)

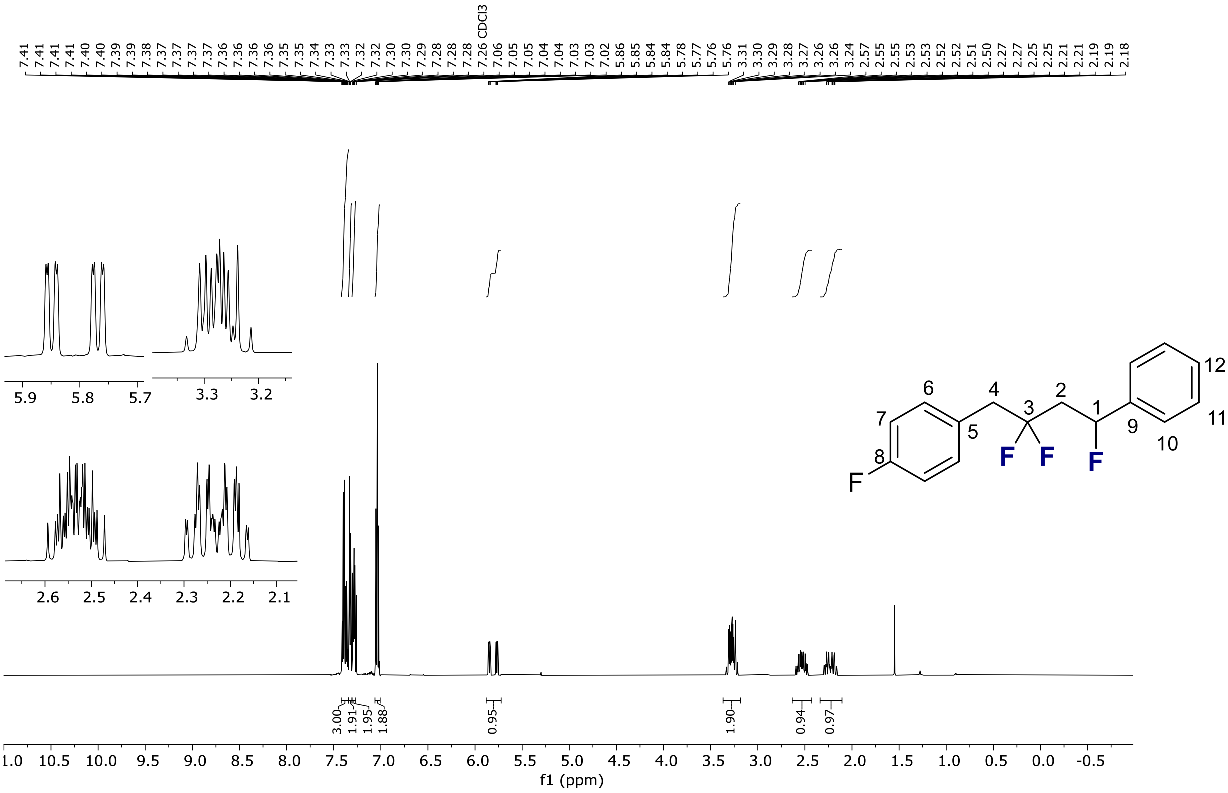


**Supplementary Figure 113.** $^{1}H$ NMR of **2f** (599 MHz, 299 K, $CDCl_3$).

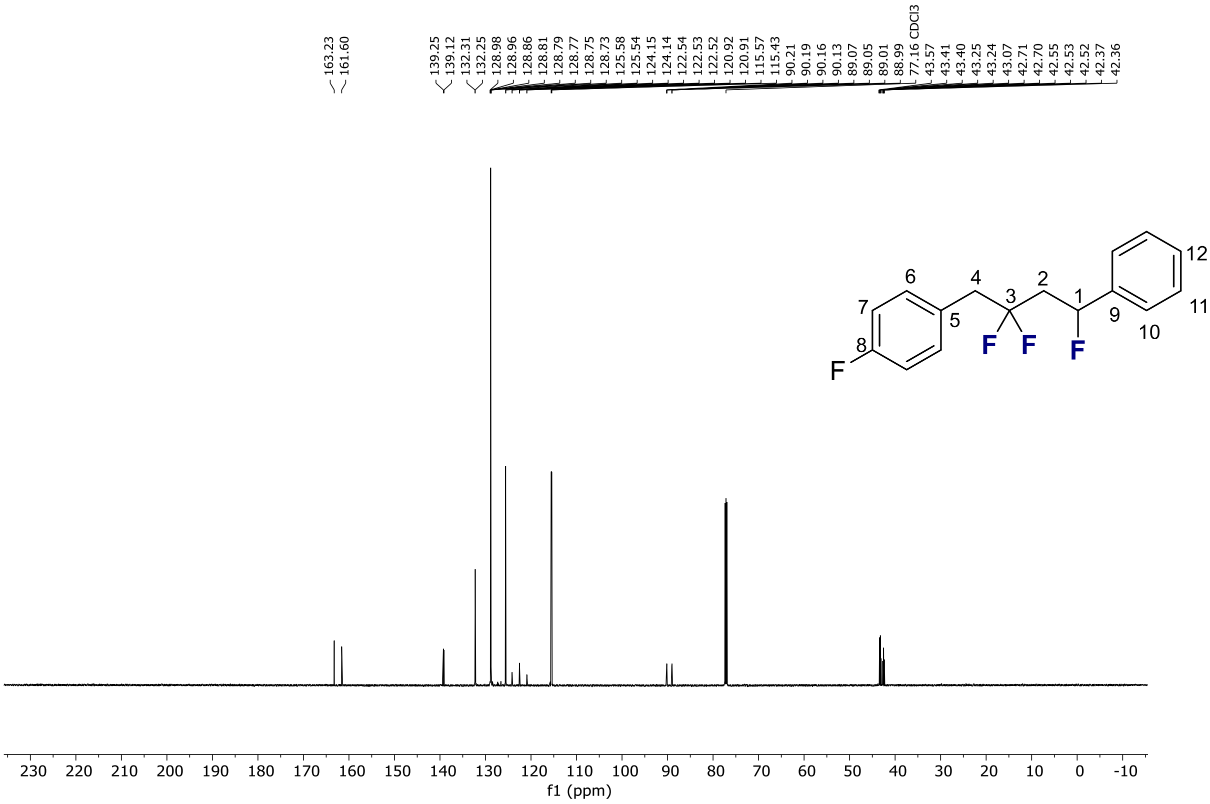


**Supplementary Figure 114.** $^{13}C\{^{1}H\}$ NMR of **2f** (151 MHz, 299 K, $CDCl_3$).

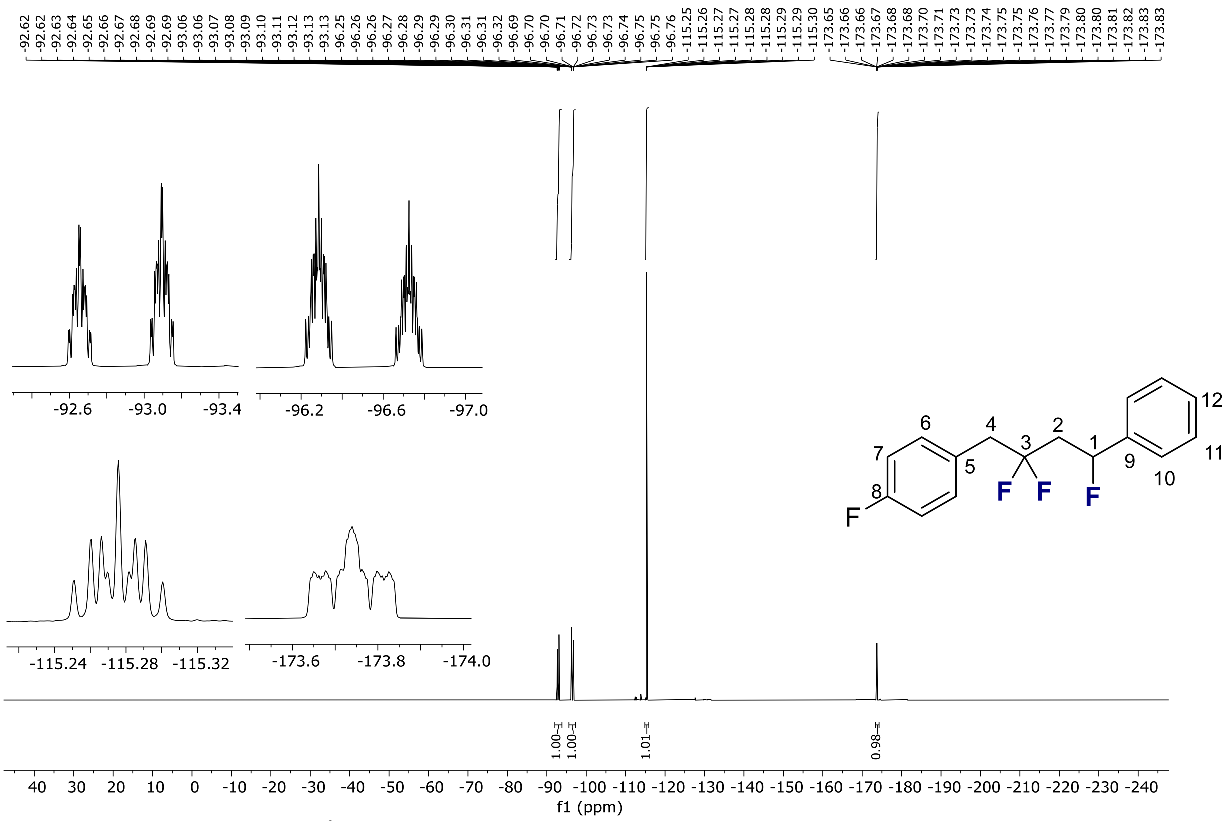


**Supplementary Figure 115.** $^{19}F$ NMR of **2f** (564 MHz, 299 K, $CDCl_3$).

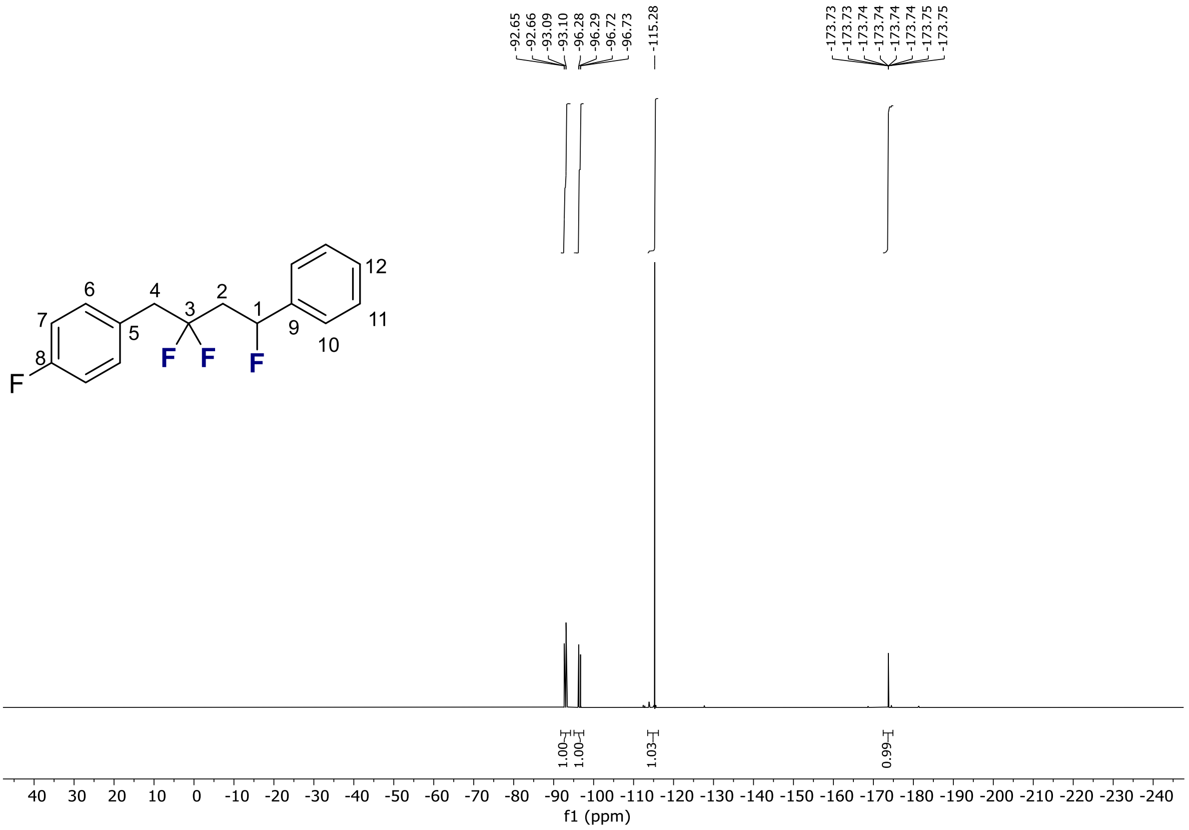


**Supplementary Figure 116.** $^{19}F\{^1H\}$ NMR of **2f** (564 MHz, 299 K, $CDCl_3$).

## 1-Chloro-4-(2,2,4-trifluoro-4-phenylbutyl)benzene (2g)

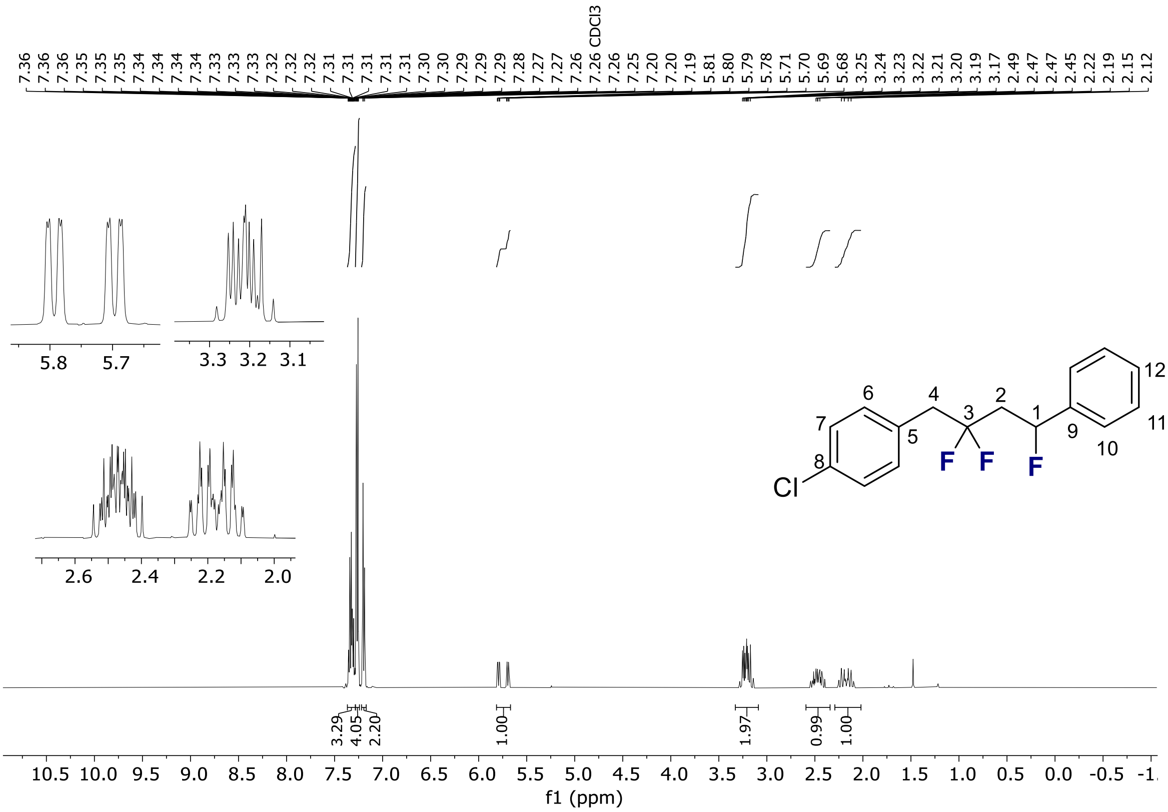


**Supplementary Figure 117.** $^{1}H$ NMR of **2g** (500 MHz, 299 K, $CDCl_3$).

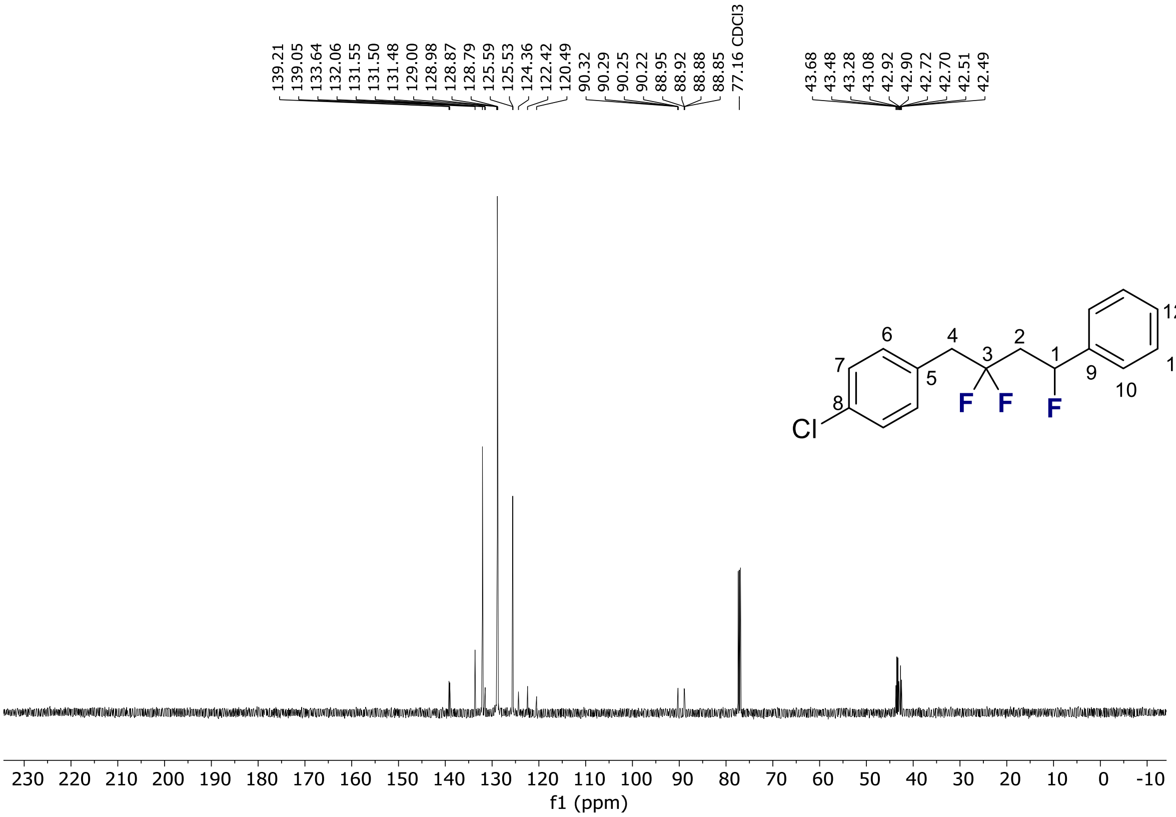


**Supplementary Figure 118.** $^{13}C\{^{1}H\}$ NMR of **2g** (126 MHz, 299 K, $CDCl_3$).

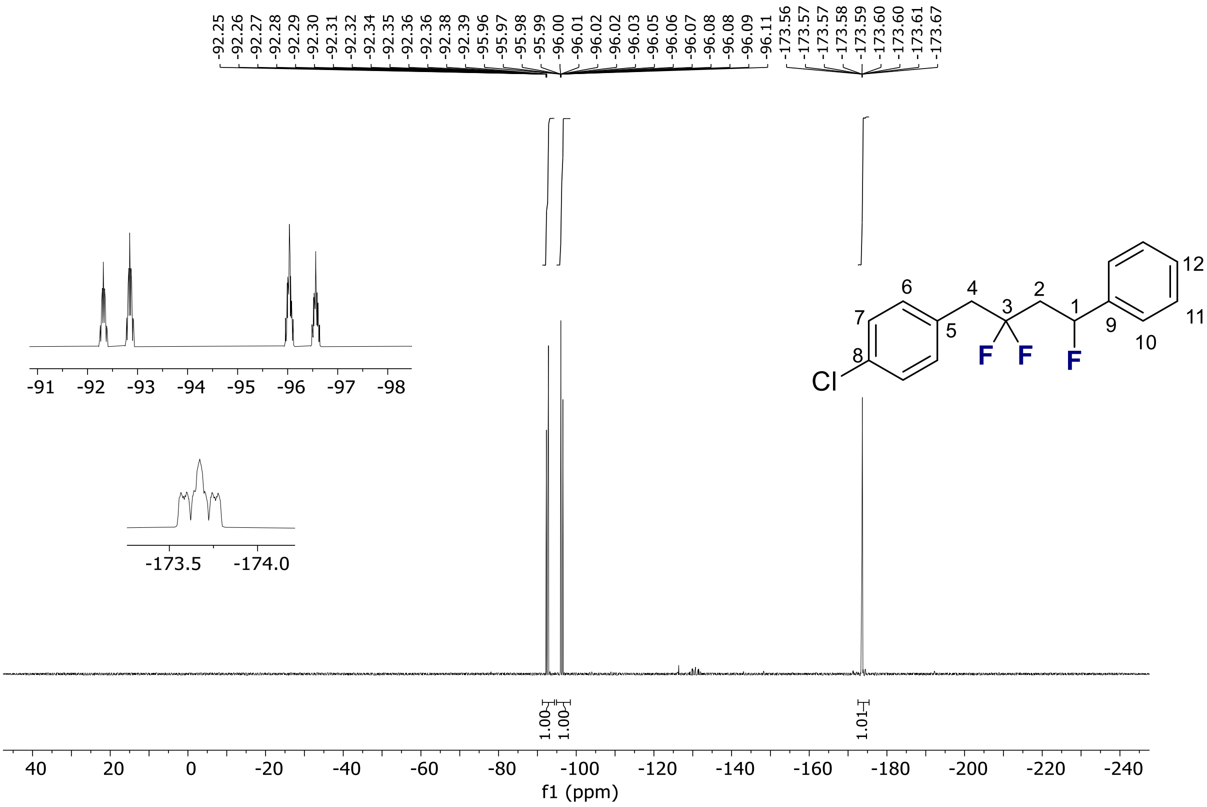


**Supplementary Figure 119.** $^{19}$F NMR of **2g** (470 MHz, 299 K, $CDCl_3$).

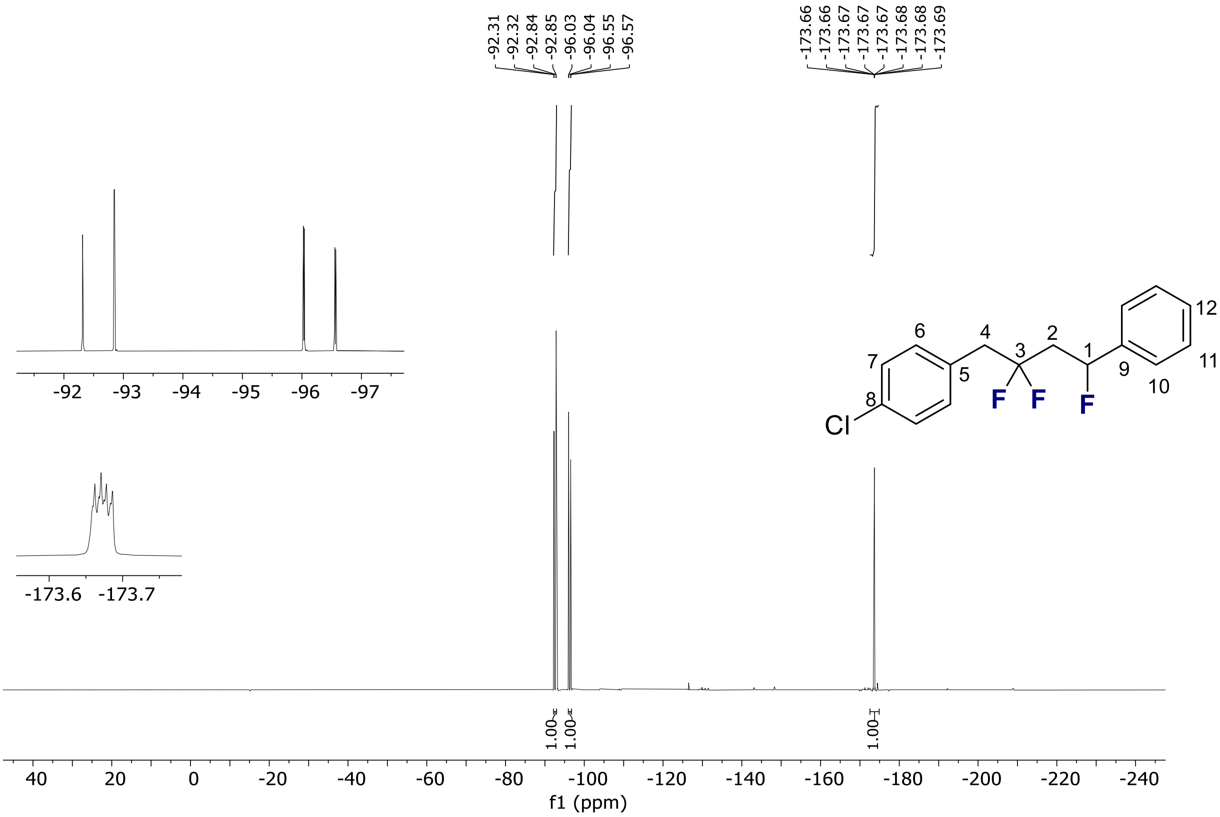


**Supplementary Figure 120.** $^{19}$F{$^{1}$H} NMR of **2g** (470 MHz, 299 K, $CDCl_3$).

**1-Bromo-4-(2,2,4-trifluoro-4-phenylbutyl)benzene (2h)**

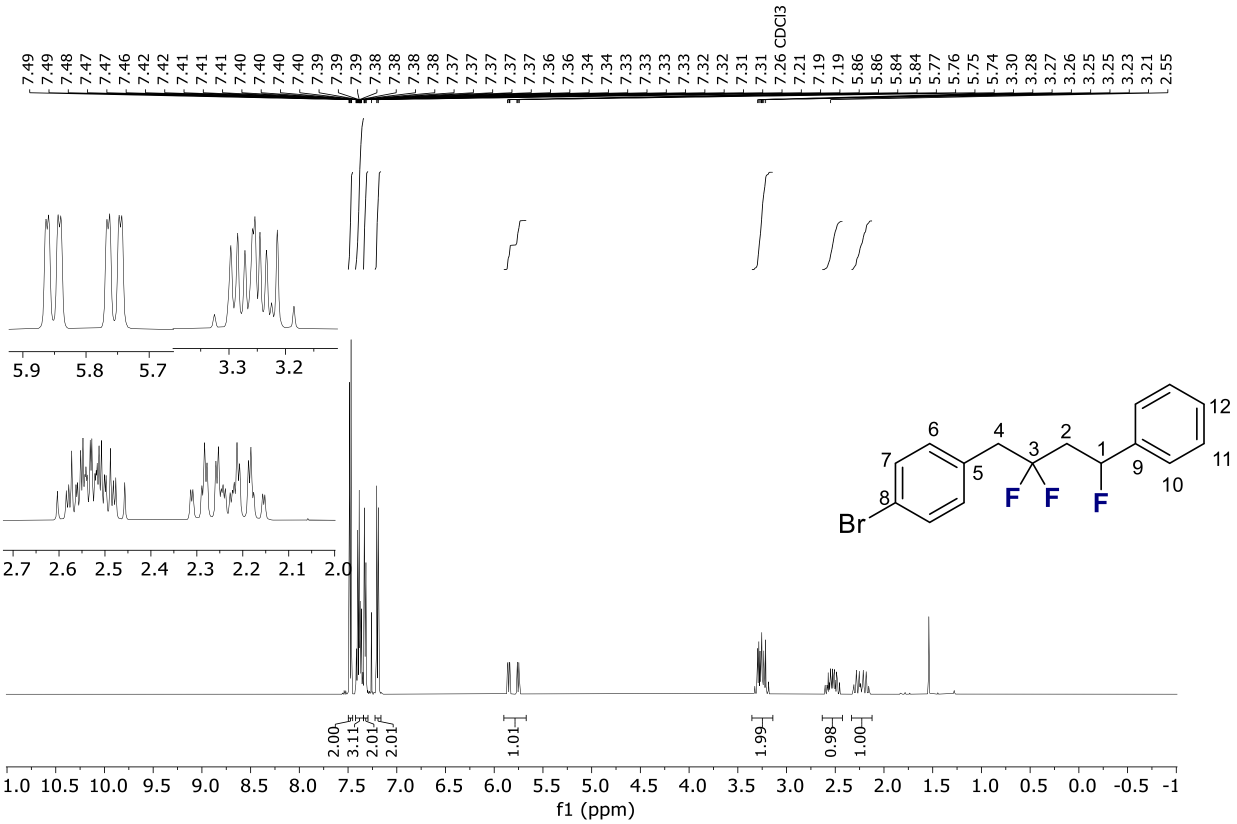


**Supplementary Figure 121.** $^{1}$H NMR of **2h** (500 MHz, 299 K, $CDCl_3$).

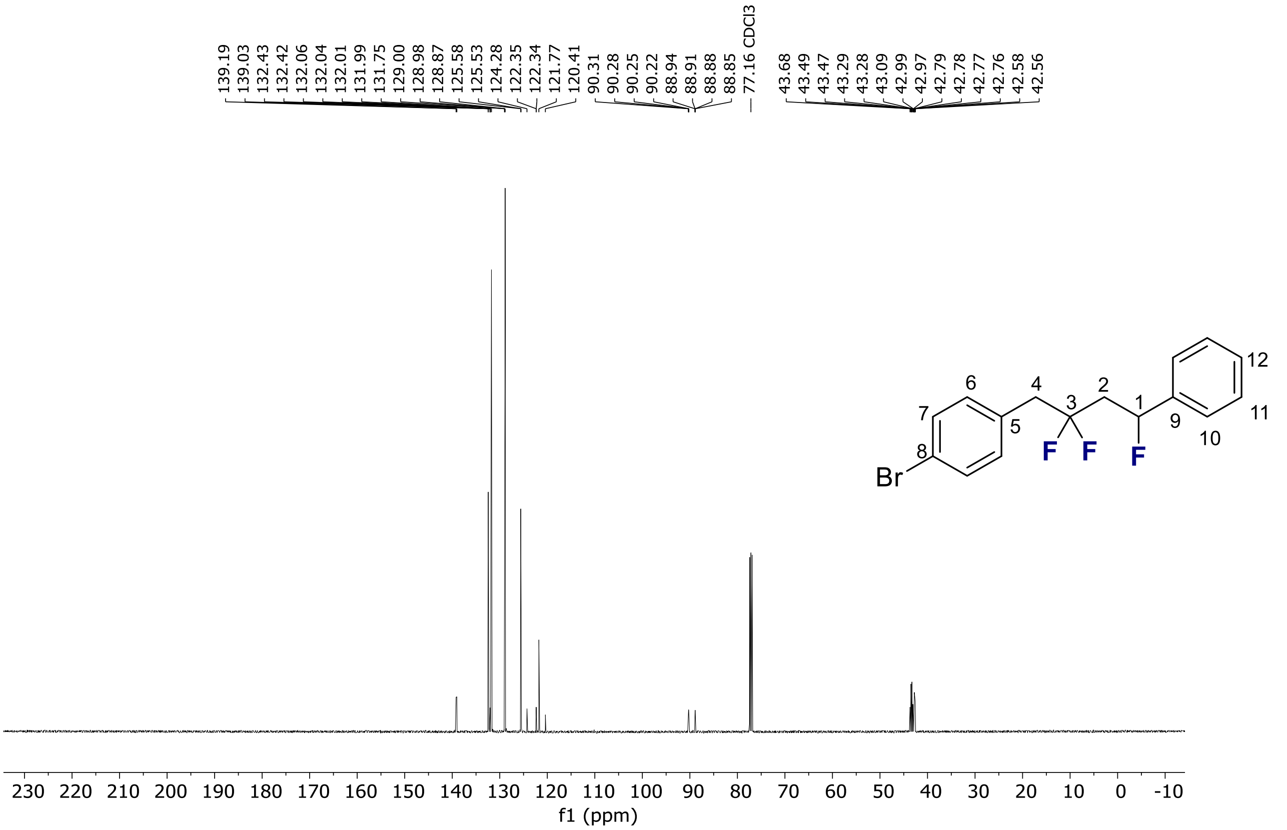


**Supplementary Figure 122.** $^{13}C\{^{1}H\}$ NMR of **2h** (126 MHz, 299 K, $CDCl_3$).

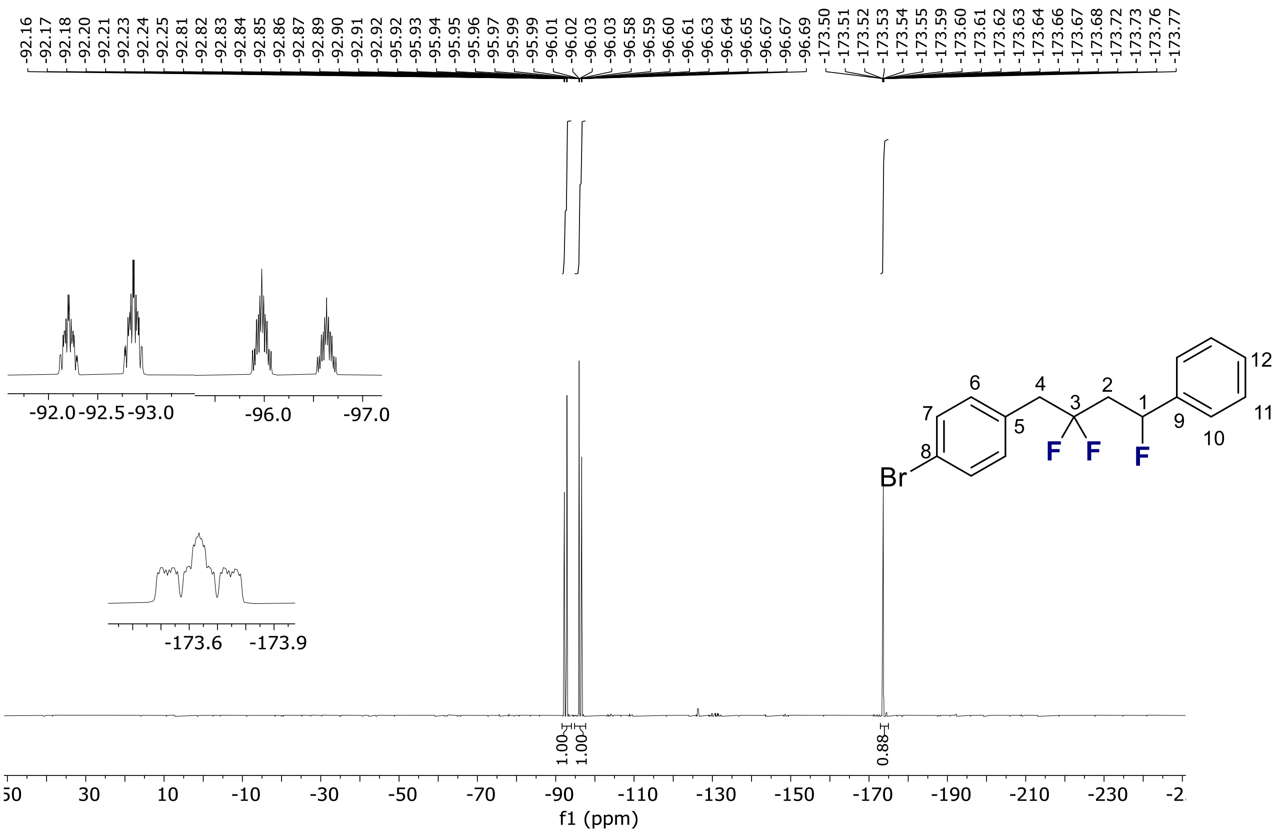


**Supplementary Figure 123.** $^{19}$F NMR of **2h** (376 MHz, 299 K, $CDCl_3$).

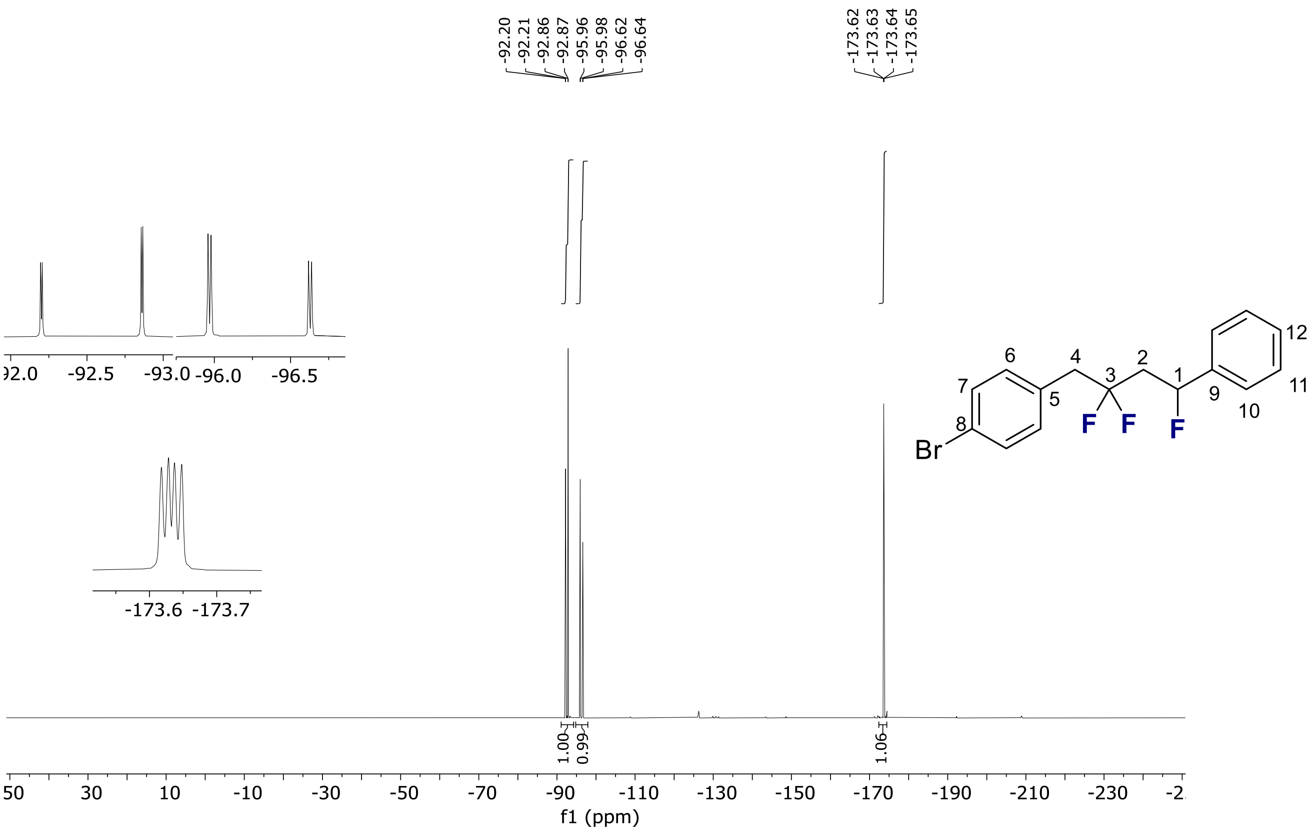


**Supplementary Figure 124.** $^{19}$F{$^1$H} NMR of **2h** (376 MHz, 299 K, $CDCl_3$).

## 1-(2,2,4-Trifluoro-4-phenylbutyl)-4-(trifluoromethoxy)benzene (2i)

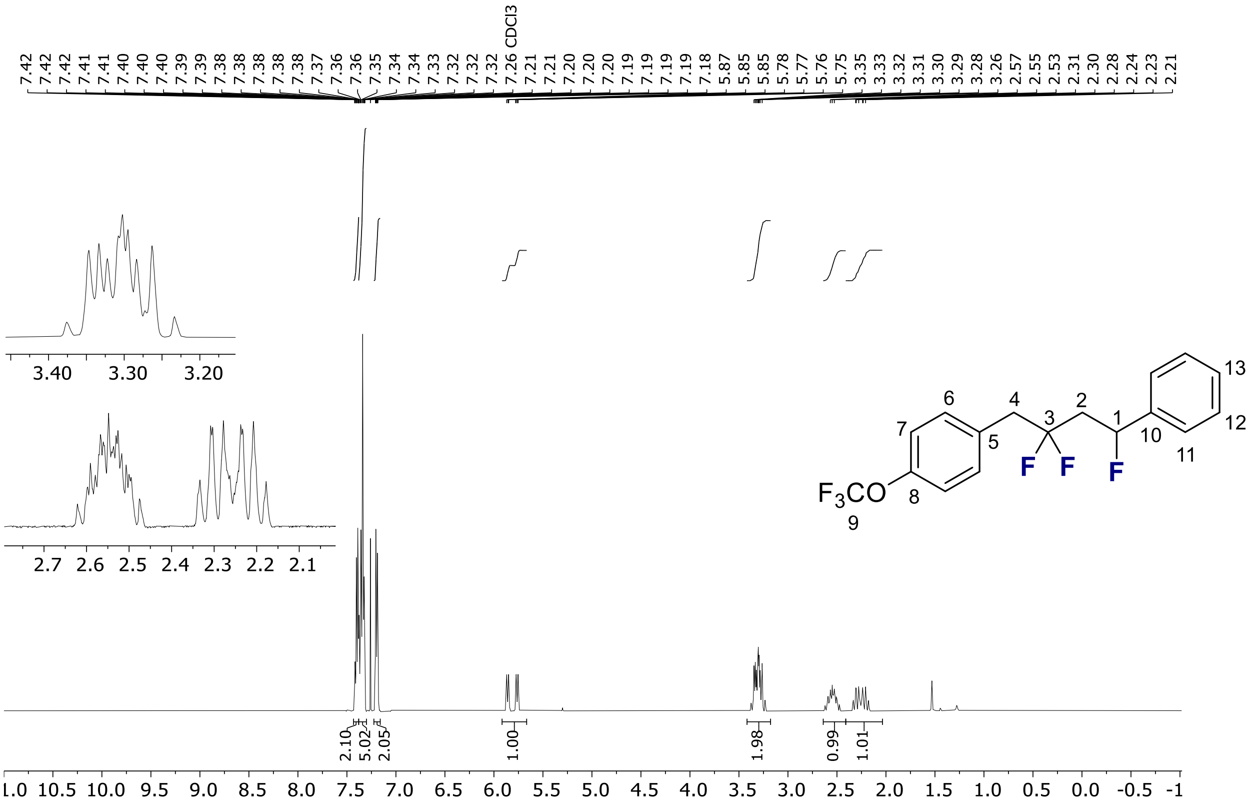


**Supplementary Figure 125.** $^{1}H$ NMR of **2i** (500 MHz, 299 K, $CDCl_3$).

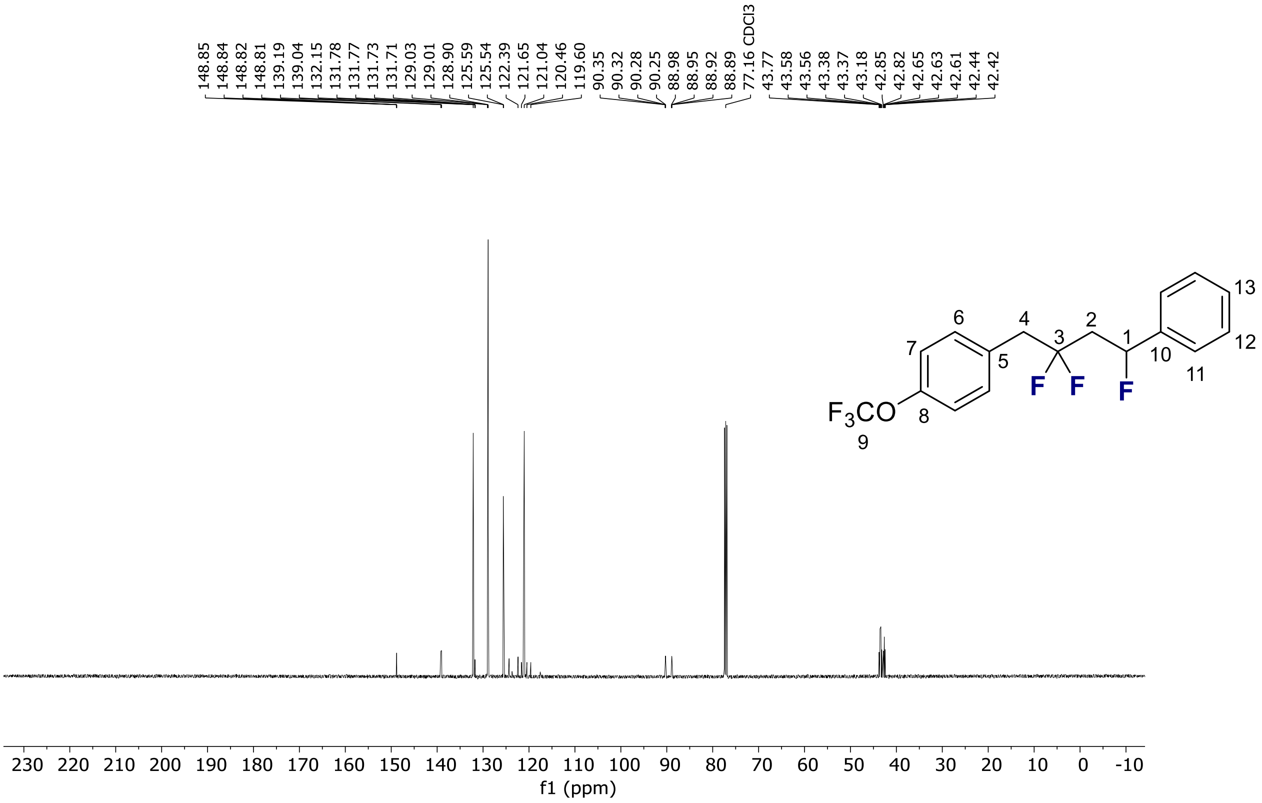


**Supplementary Figure 126.** $^{13}C\{^{1}H\}$ NMR of **2i** (126 MHz, 299 K, $CDCl_3$).

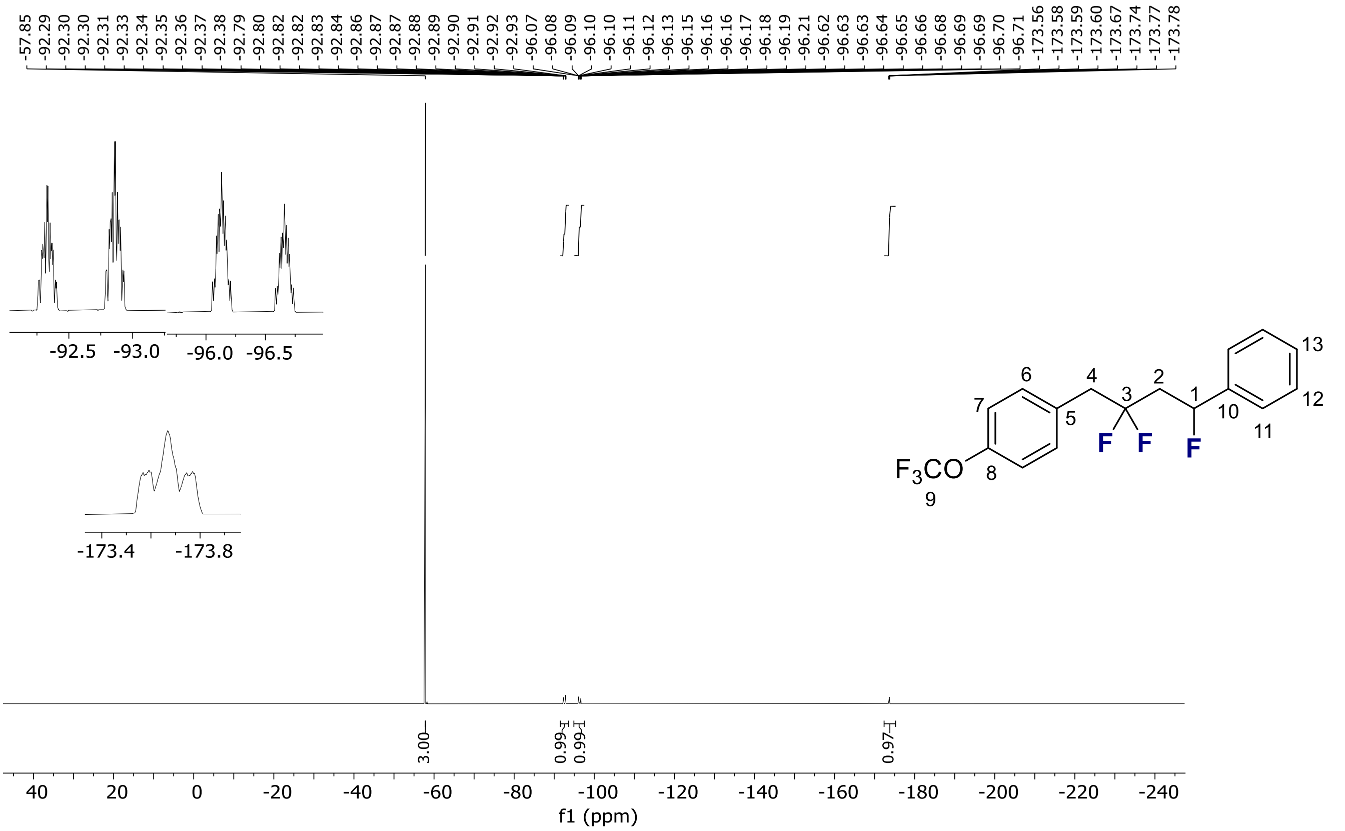


**Supplementary Figure 127.** $^{19}F$ NMR of **2i** (470 MHz, 299 K, $CDCl_3$).

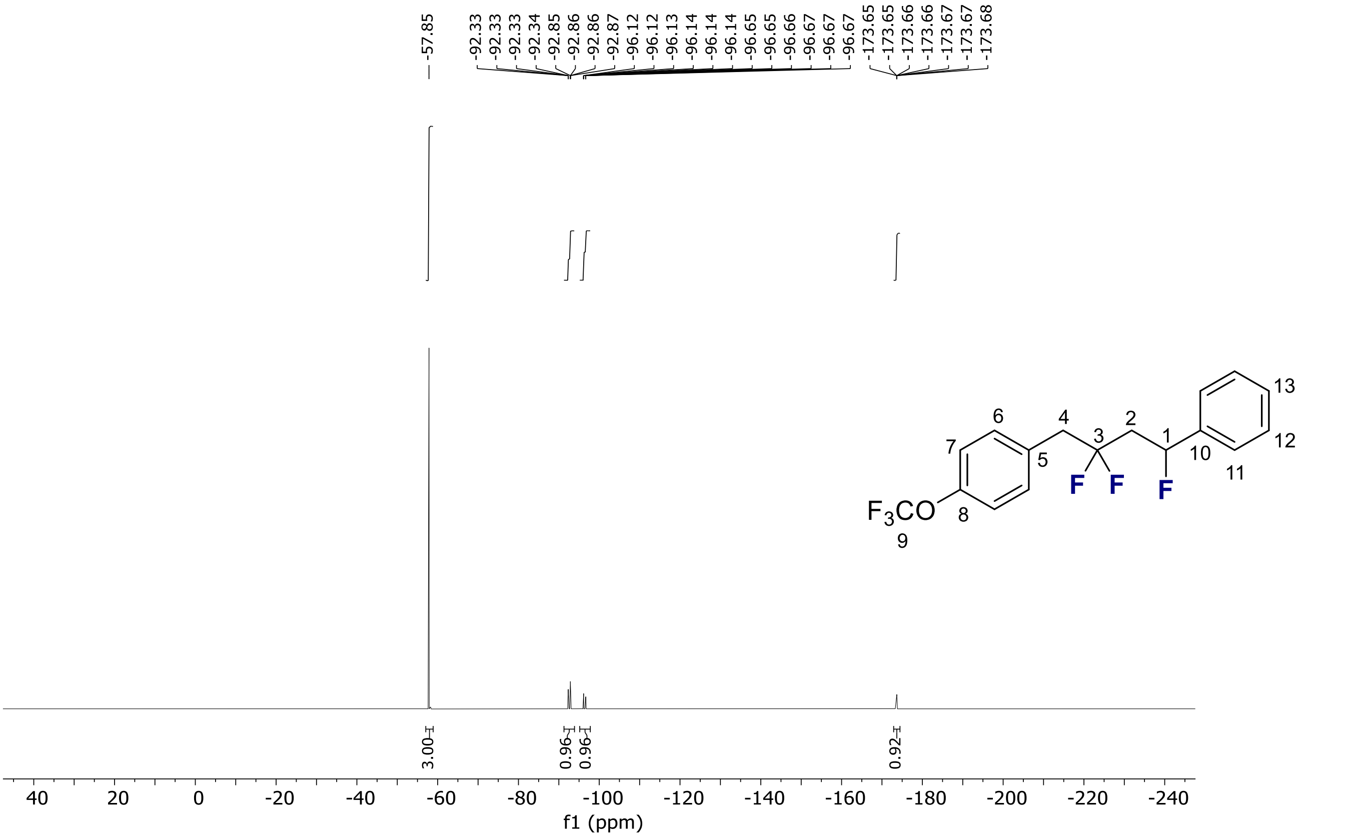


**Supplementary Figure 128.** $^{19}F\{^{1}H\}$ NMR of **2i** (470 MHz, 299 K, $CDCl_3$).

## 1-(2,2,4-Trifluoro-4-phenylbutyl)-4-(trifluoromethyl)benzene (2j)

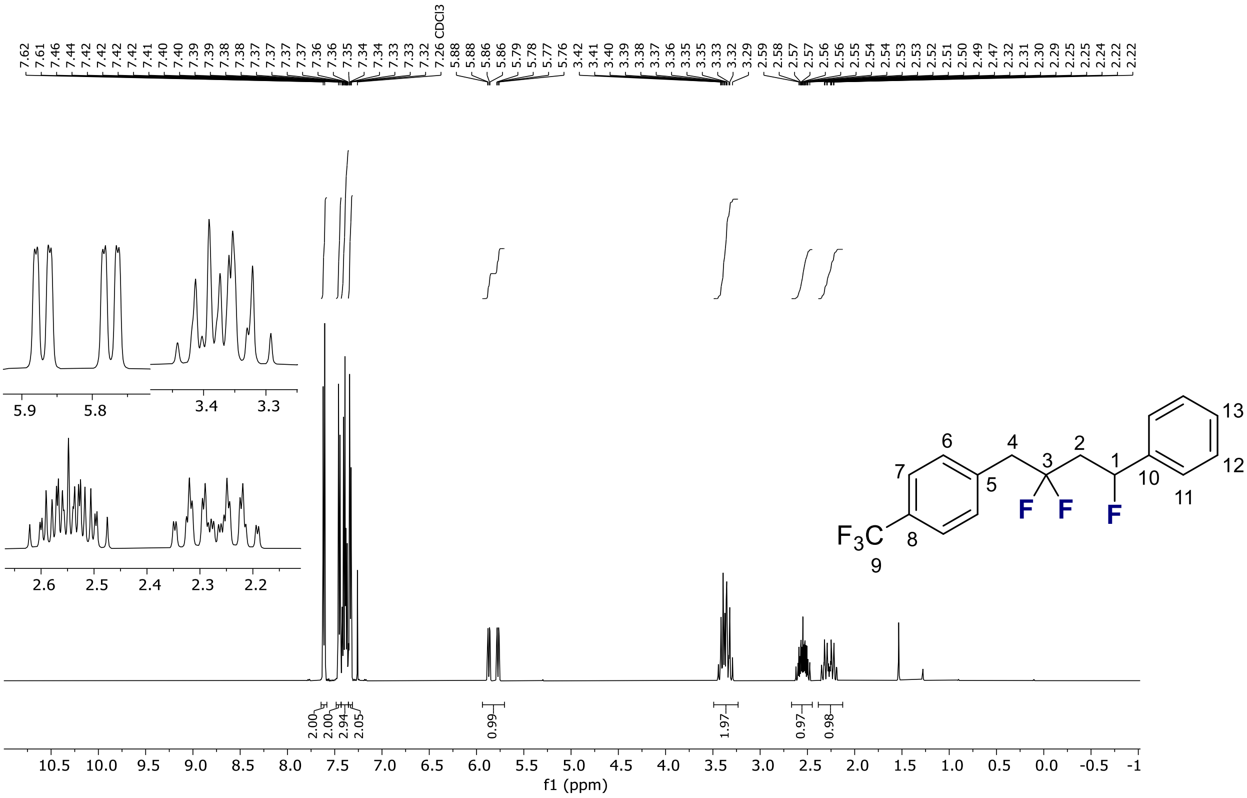


**Supplementary Figure 129.** $^{1}H$ NMR of **2j** (500 MHz, 299 K, $CDCl_3$).

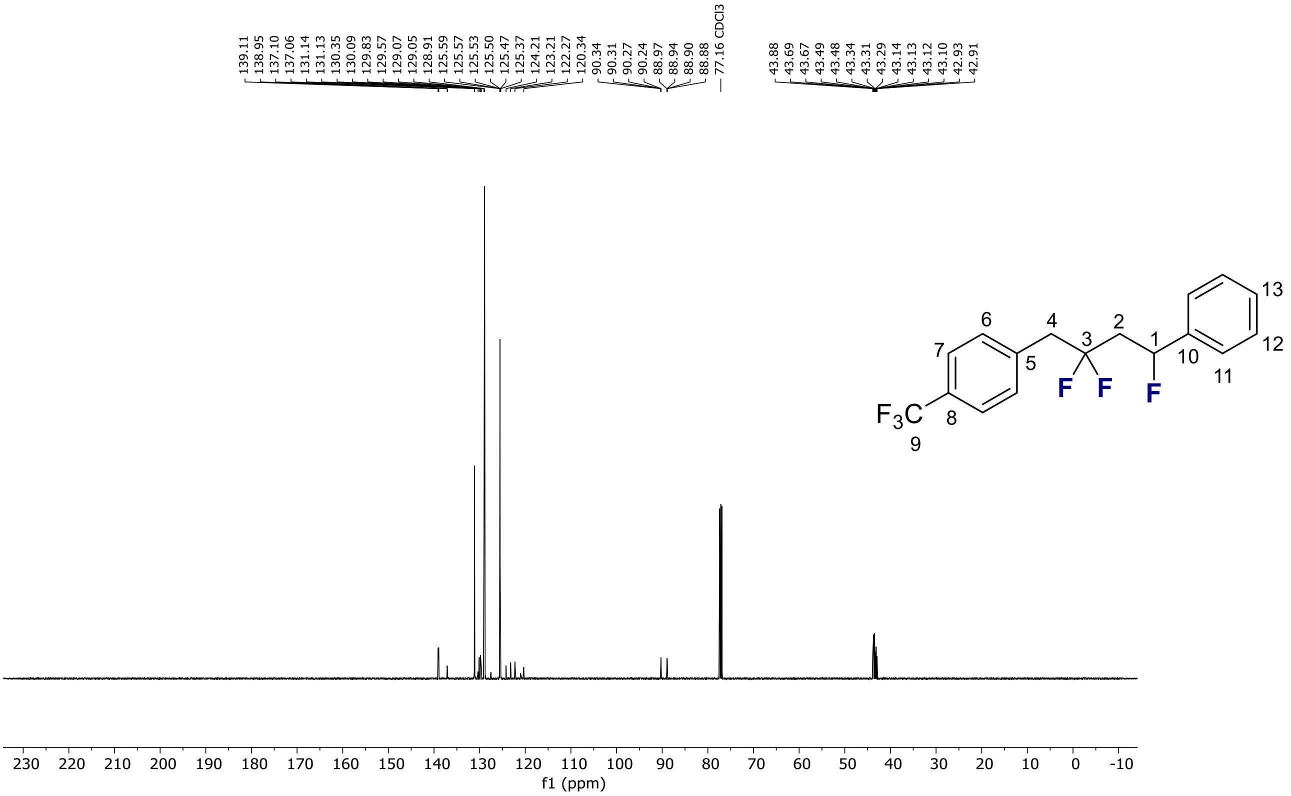


**Supplementary Figure 130.** $^{13}C\{^{1}H\}$ NMR of **2j** (126 MHz, 299 K, $CDCl_3$).

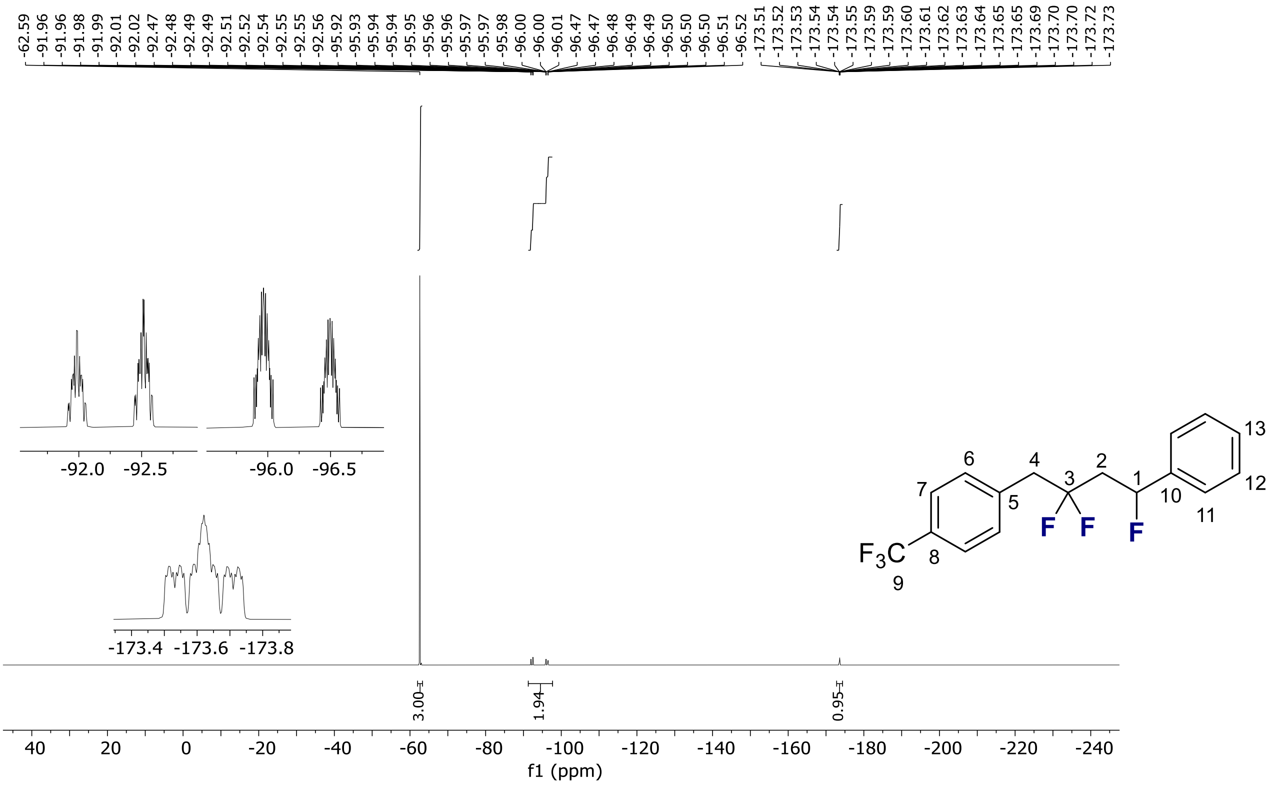


**Supplementary Figure 131.** $^{19}F$ NMR of **2j** (470 MHz, 299 K, $CDCl_3$).

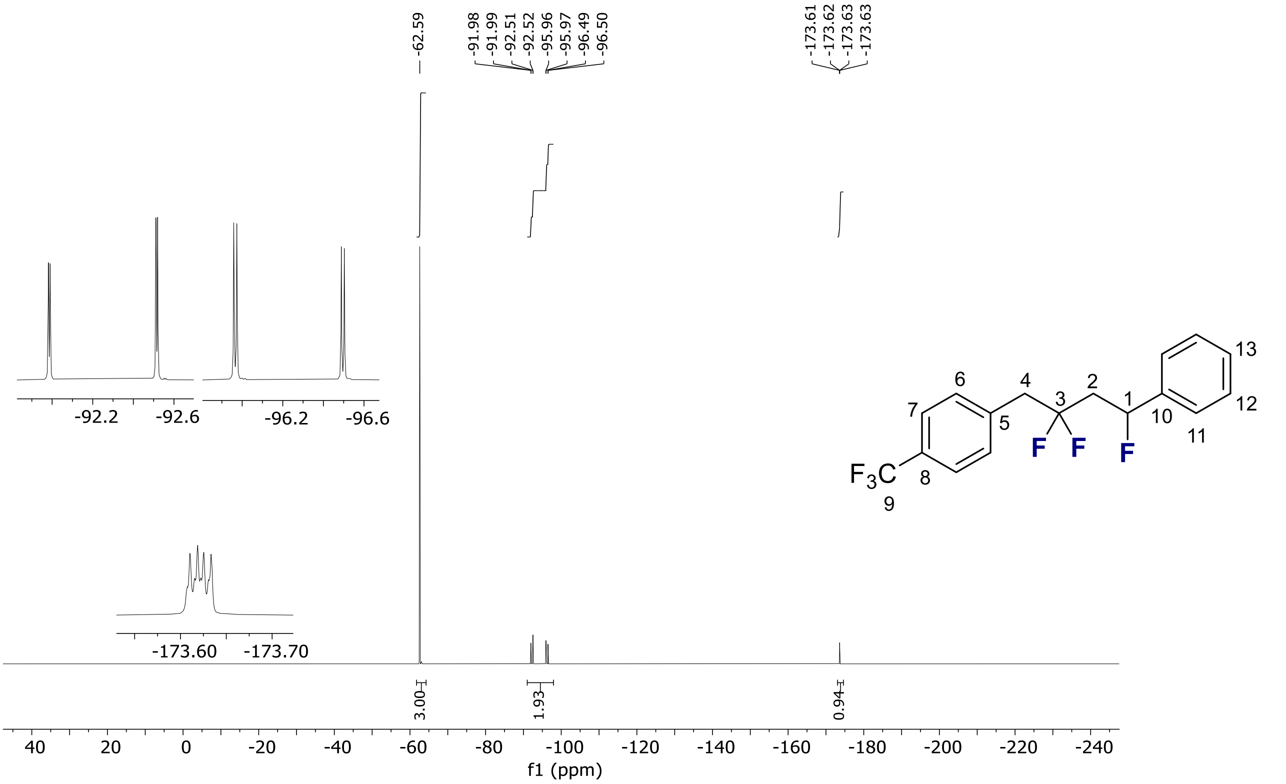


**Supplementary Figure 132.** $^{19}F\{^1H\}$ NMR of **2j** (470 MHz, 299 K, $CDCl_3$).

## 4-(2,2,4-Trifluoro-4-phenylbutyl)benzonitrile (2k)

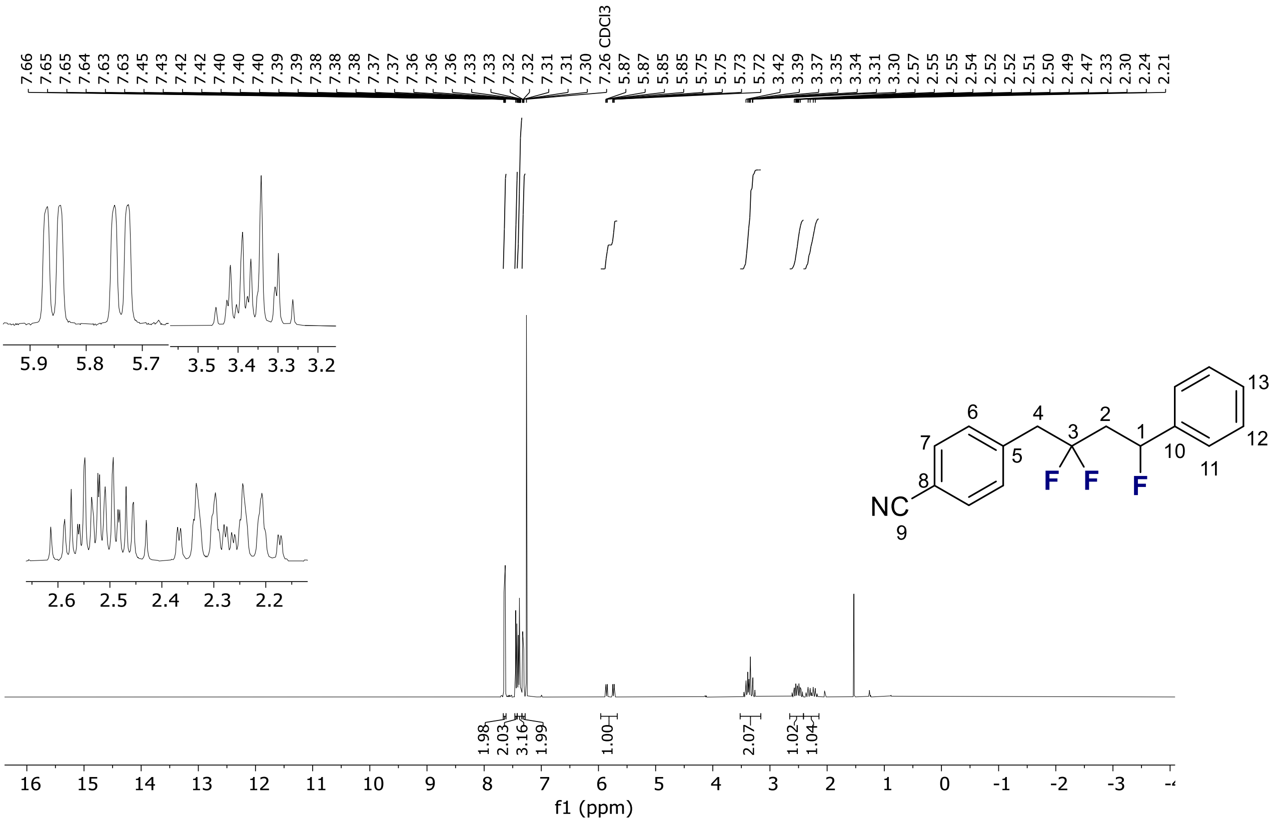


**Supplementary Figure 133.** $^{1}$H NMR of **2k** (500 MHz, 299 K, $CDCl_3$).

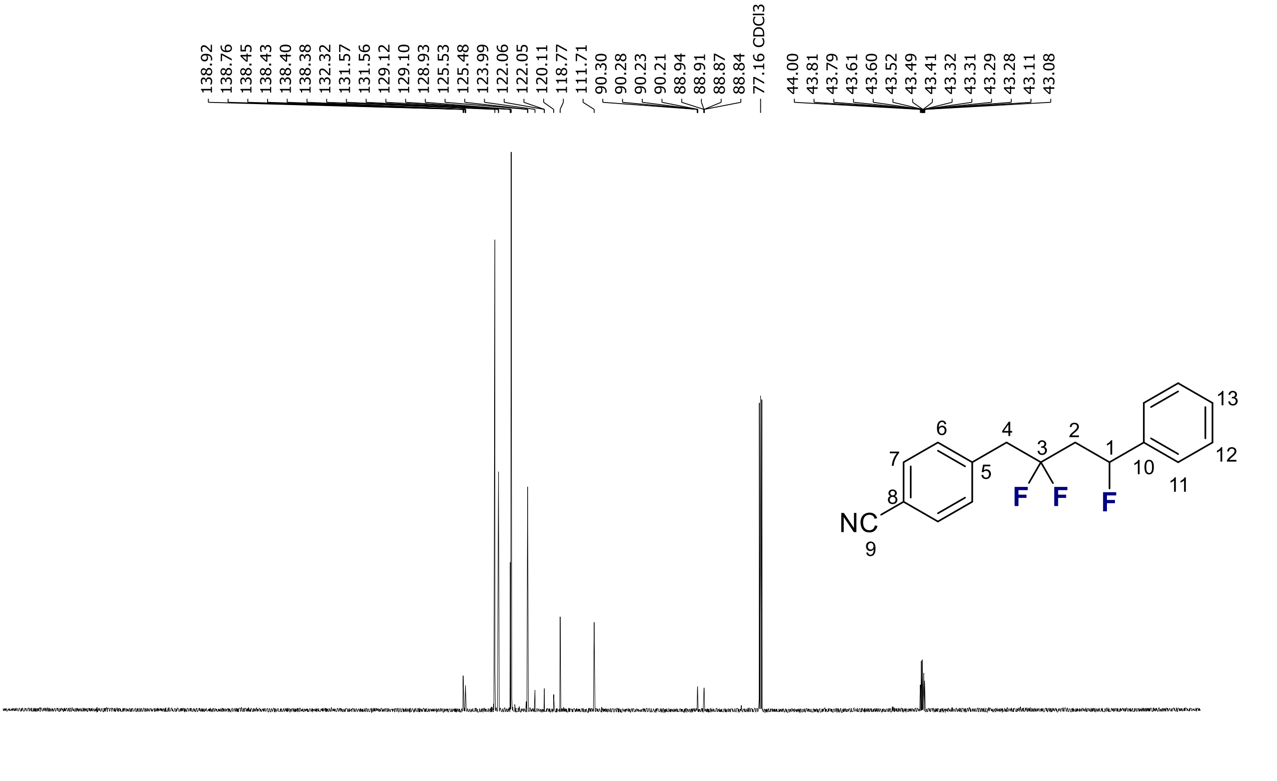


**Supplementary Figure 134.** $^{13}$C{$^{1}$H} NMR of **2k** (126 MHz, 299 K, $CDCl_3$).

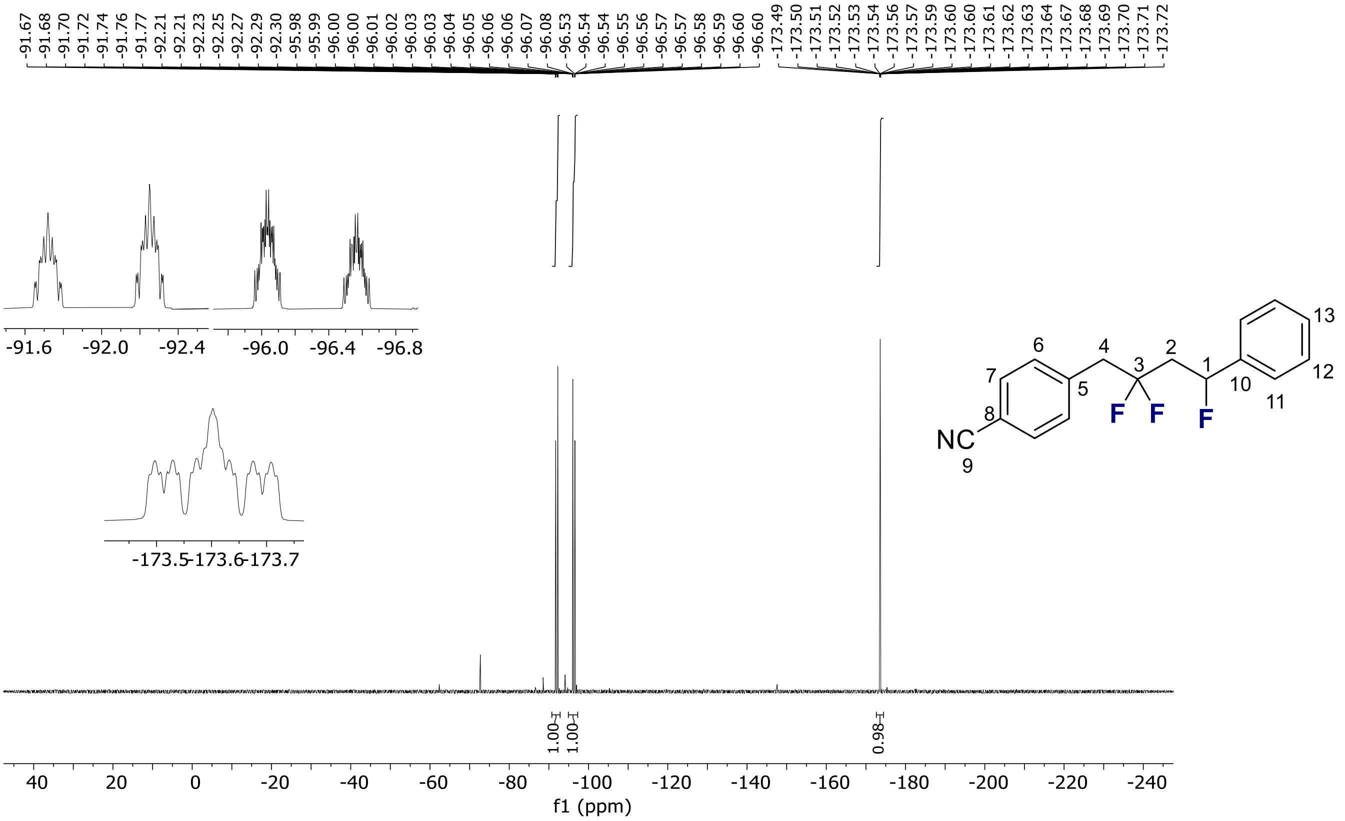


**Supplementary Figure 135.** $^{19}$F NMR of **2k** (470 MHz, 299 K, $CDCl_3$).

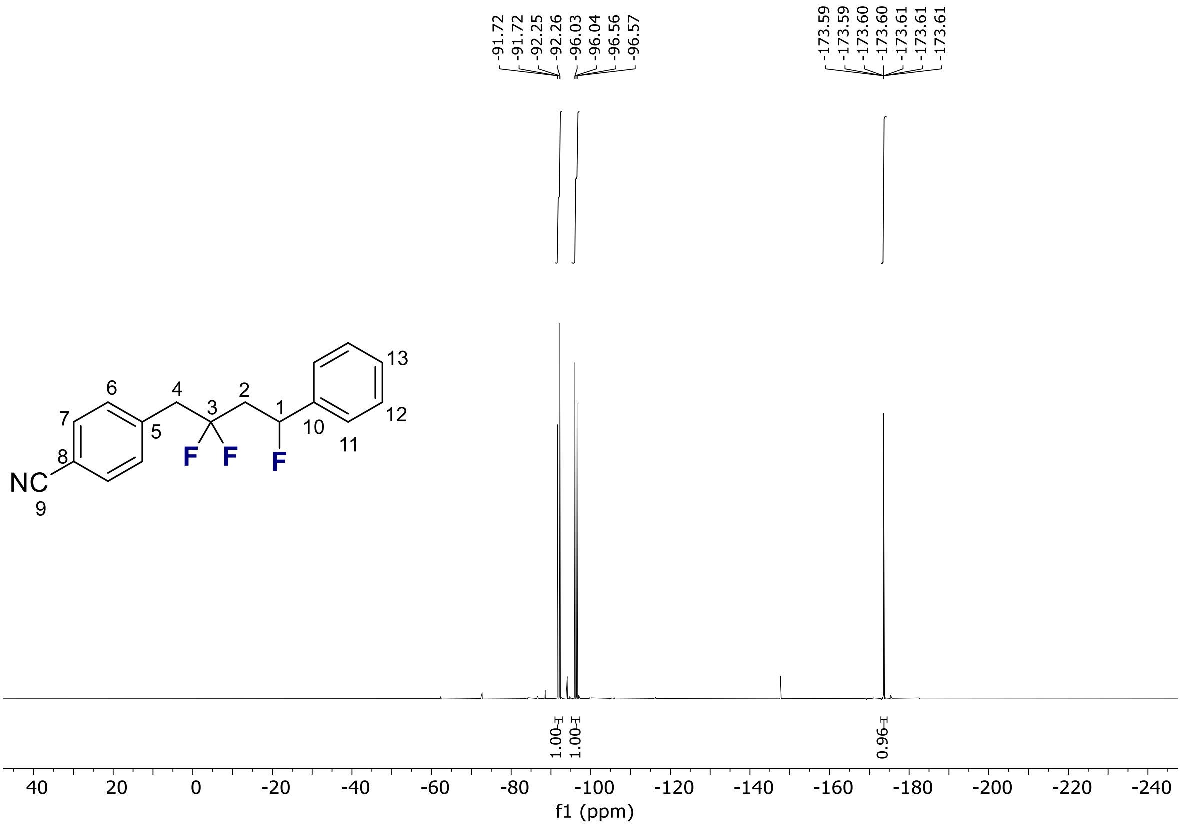


**Supplementary Figure 136.** $^{19}$F{$^{1}$H} NMR of **2k** (470 MHz, 299 K, $CDCl_3$).

## 4-(2,2,4-Trifluoro-4-phenylbutyl)-4'-(trifluoromethyl)-1,1'-biphenyl (2l)

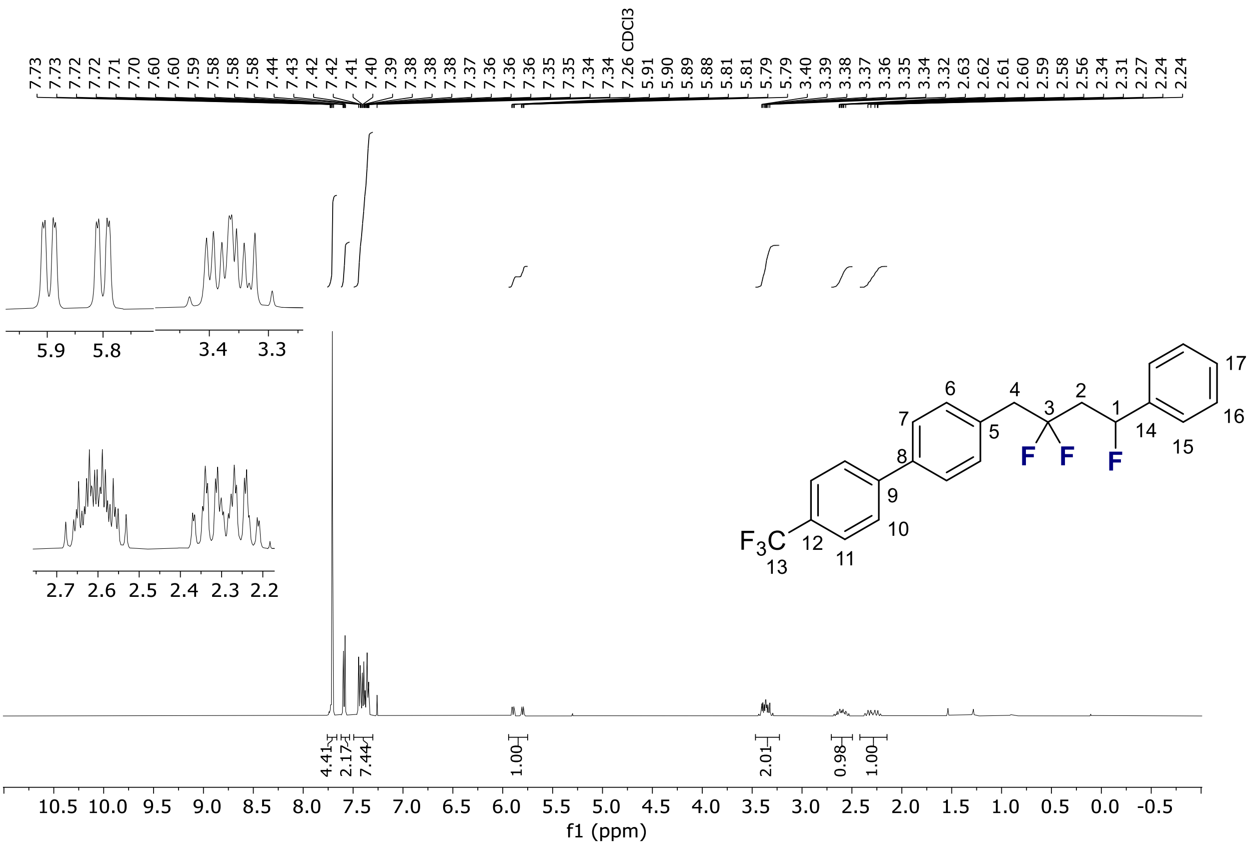


**Supplementary Figure 137.** $^{1}$H NMR of **2l** (500 MHz, 299 K, $CDCl_3$).

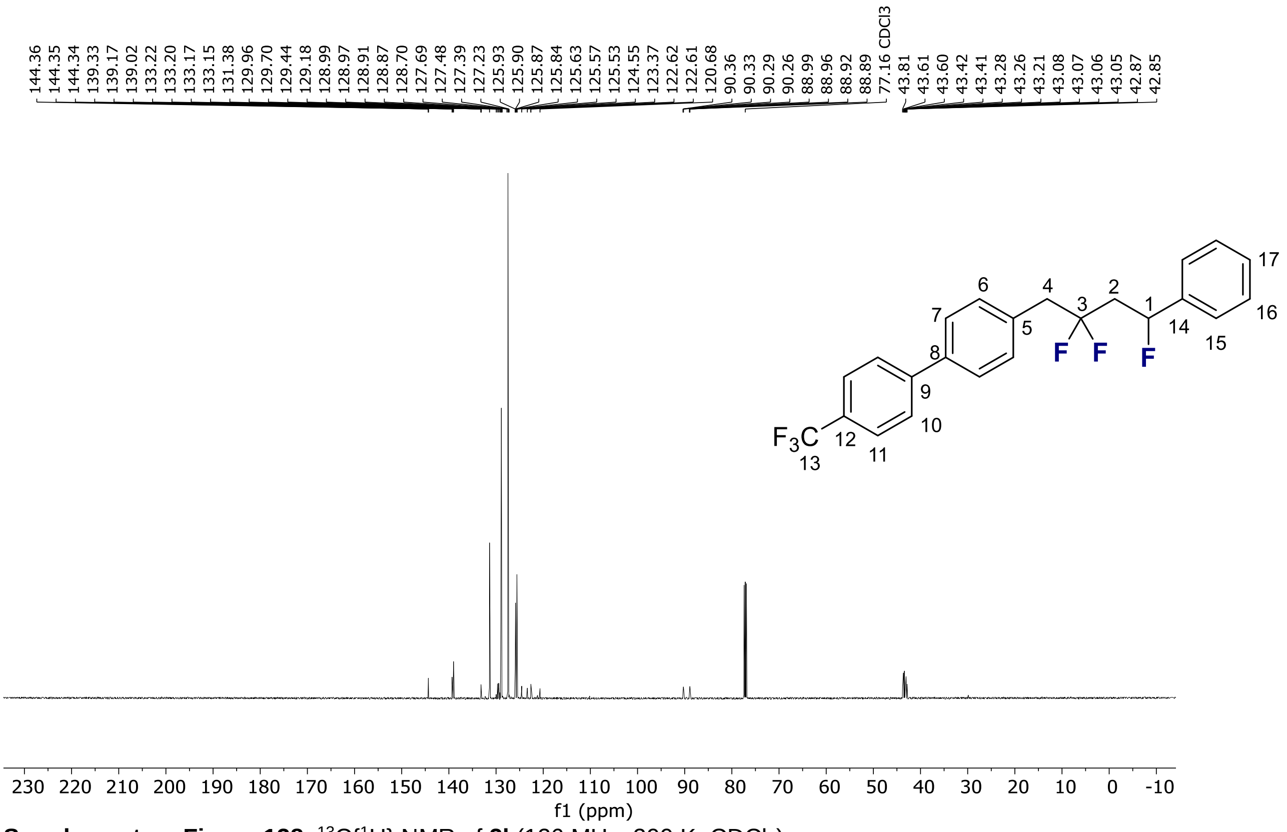


**Supplementary Figure 138.** $^{13}C\{^{1}H\}$ NMR of **2l** (126 MHz, 299 K, $CDCl_3$).

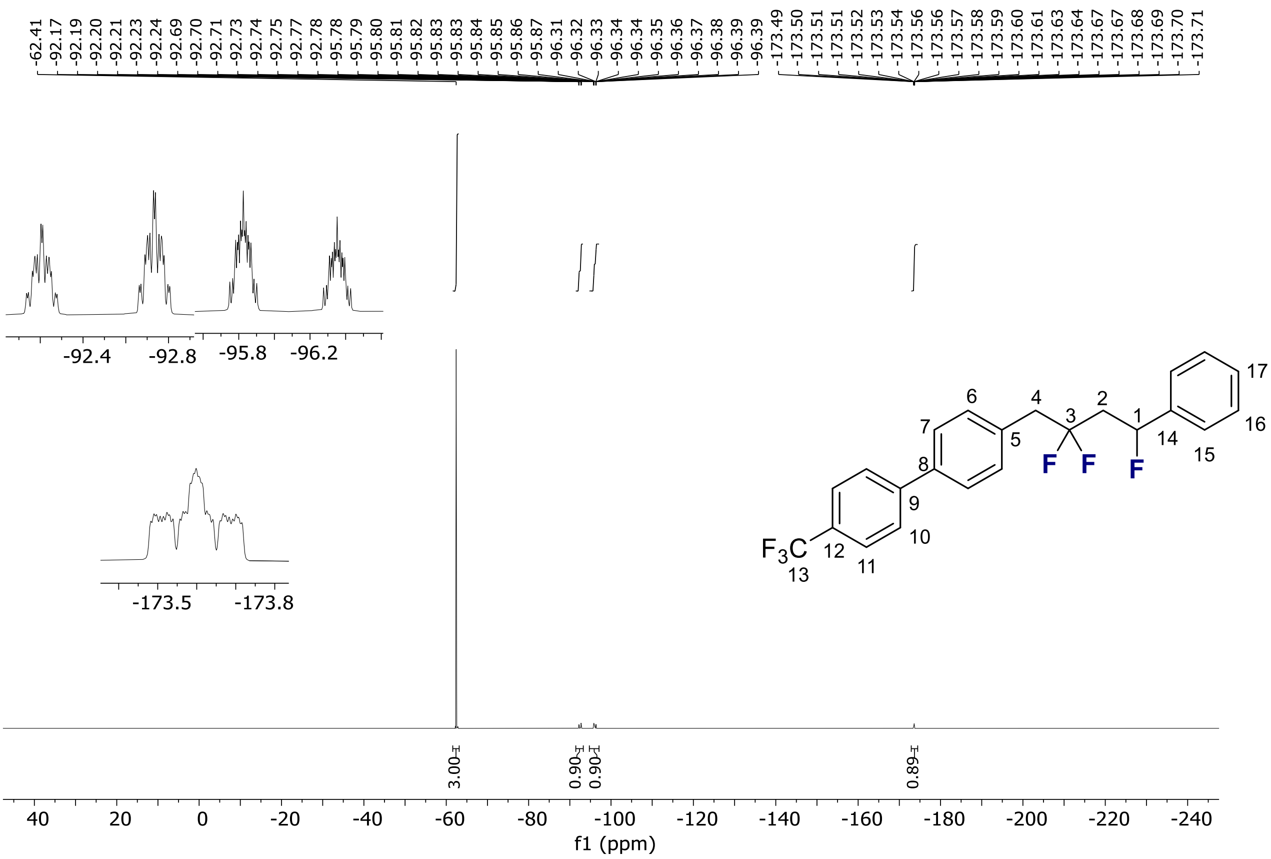


**Supplementary Figure 139.** $^{19}$F NMR of **2l** (470 MHz, 299 K, $CDCl_3$).

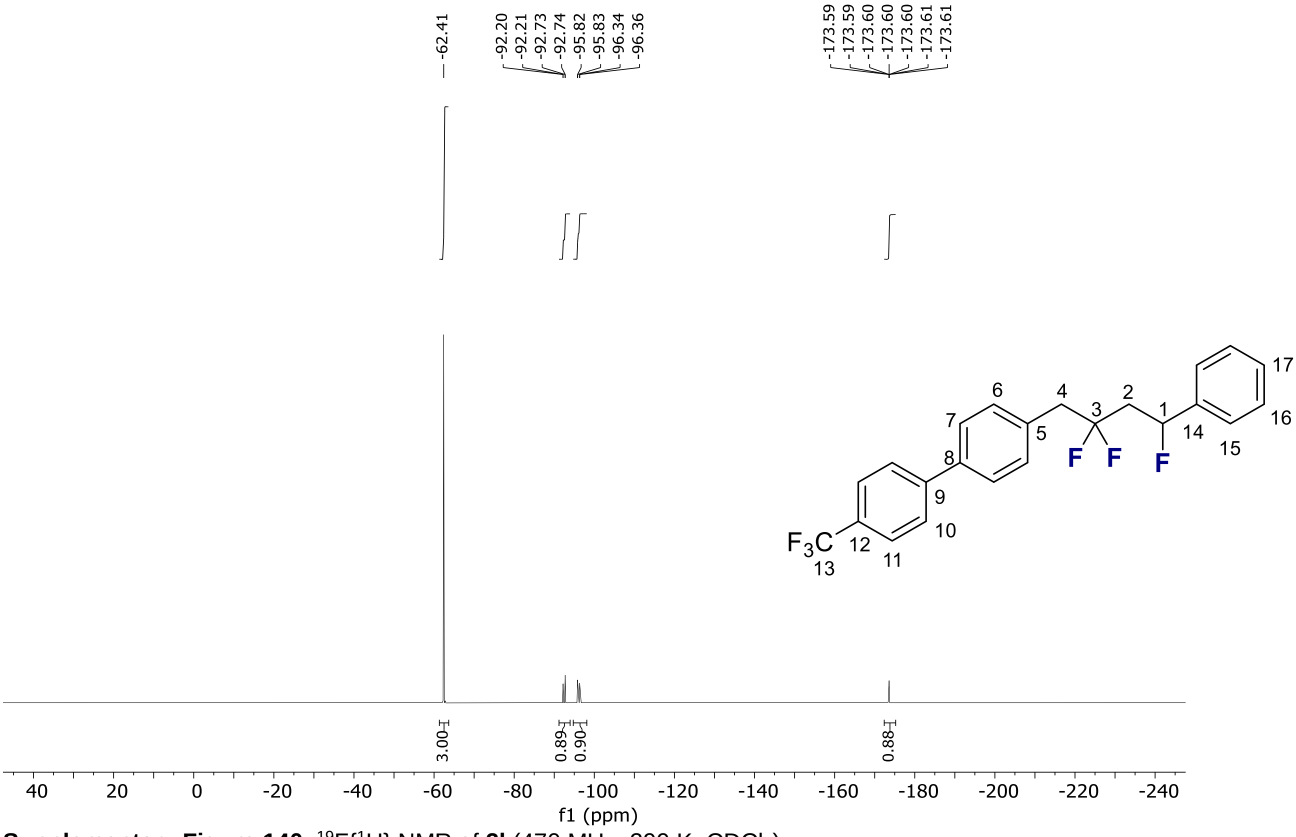


**Supplementary Figure 140.** $^{19}$F{$^{1}$H} NMR of **2l** (470 MHz, 299 K, $CDCl_3$).

## 1-Bromo-3-(2,2,4-trifluoro-4-phenylbutyl)benzene (2m)

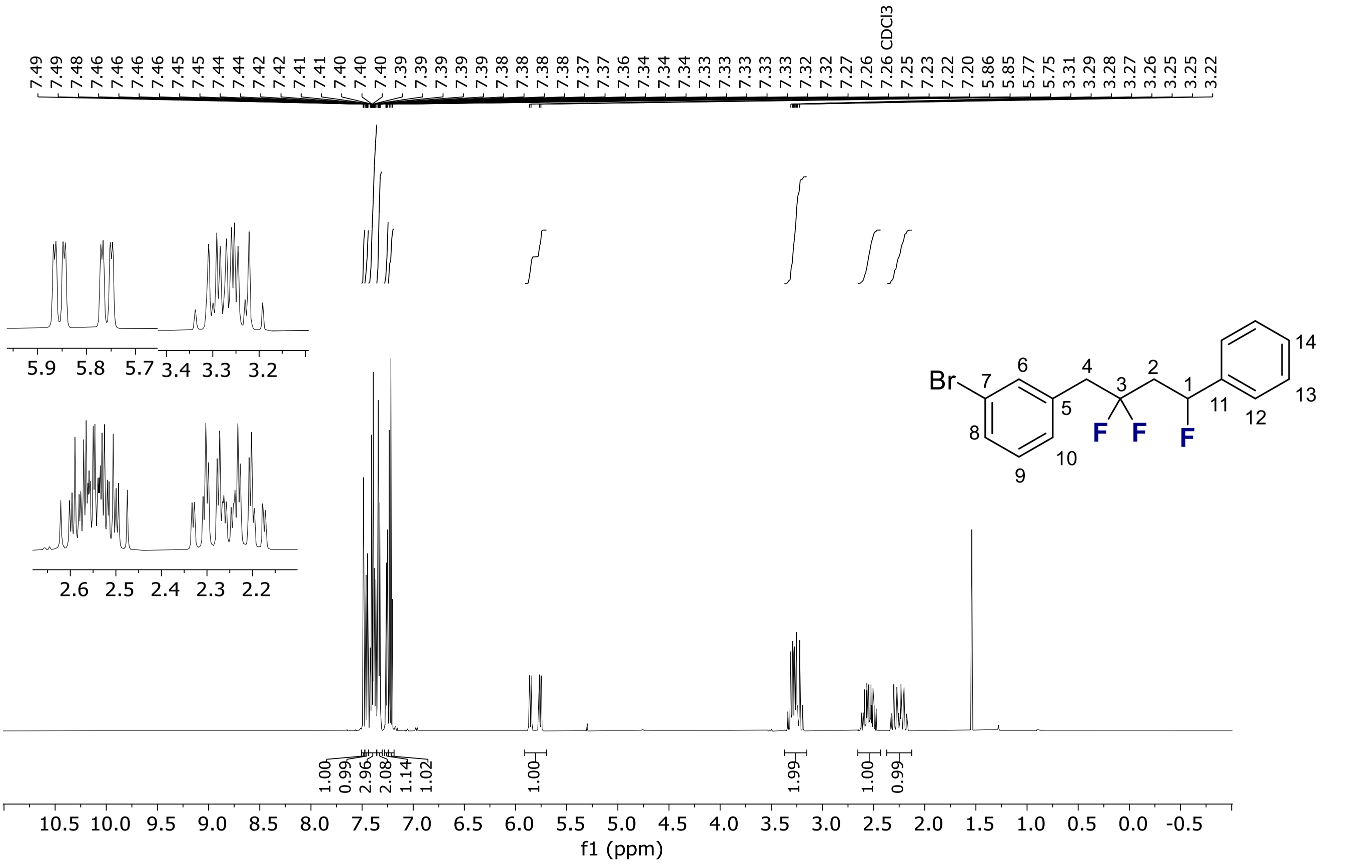


**Supplementary Figure 141.** $^{1}H$ NMR of **2m** (500 MHz, 299 K, $CDCl_3$).

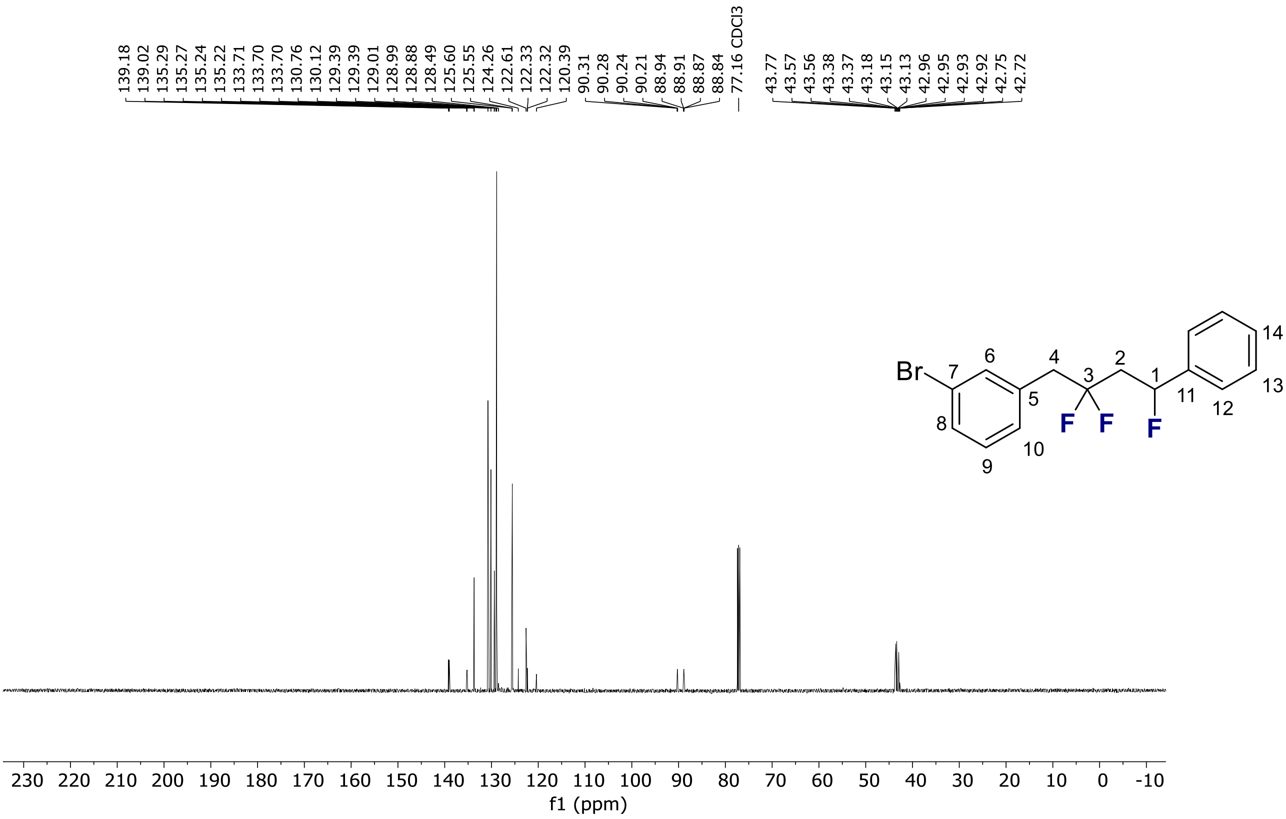


**Supplementary Figure 142.** $^{13}C\{^{1}H\}$ NMR of **2m** (126 MHz, 299 K, $CDCl_3$).

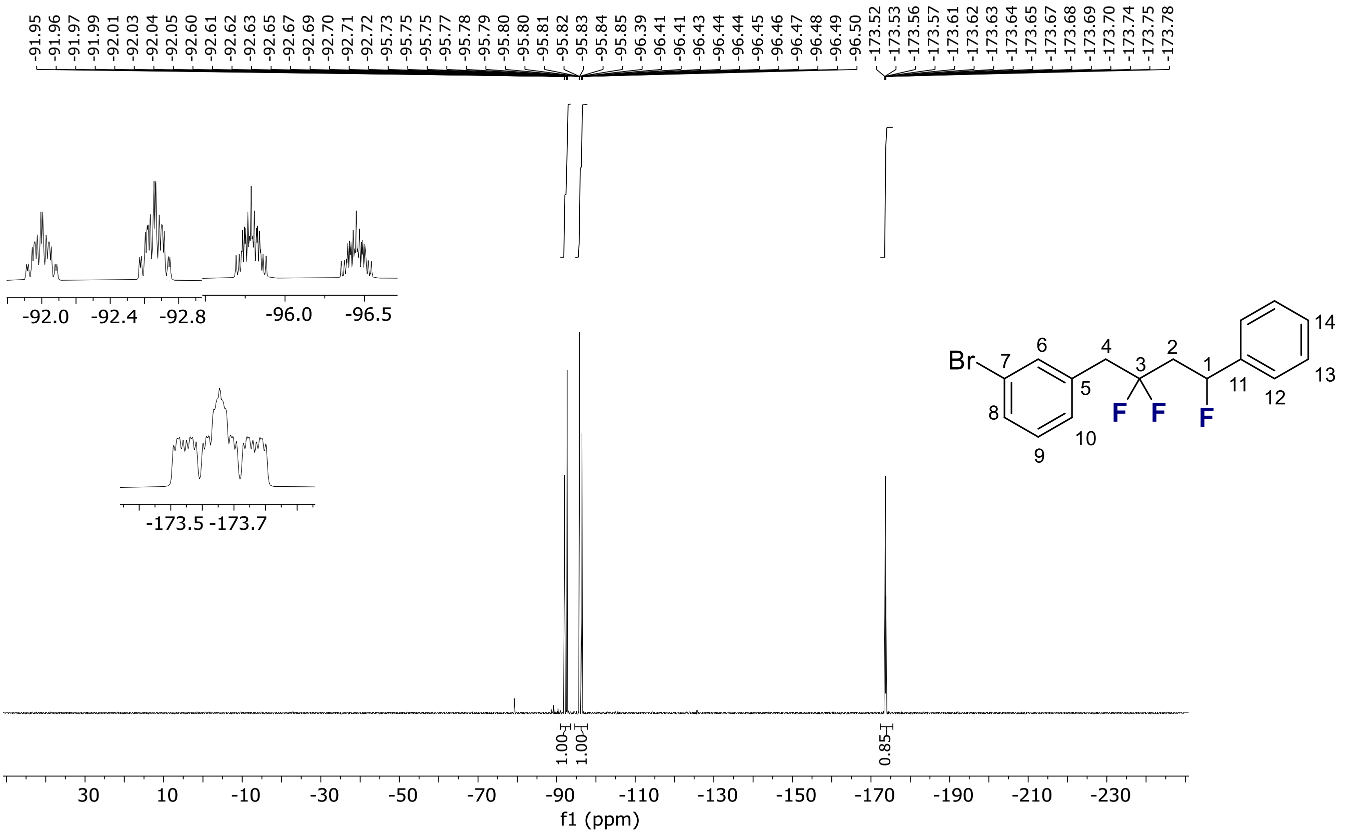


**Supplementary Figure 143.** $^{19}F$ NMR of **2m** (376 MHz, 299 K, $CDCl_3$).

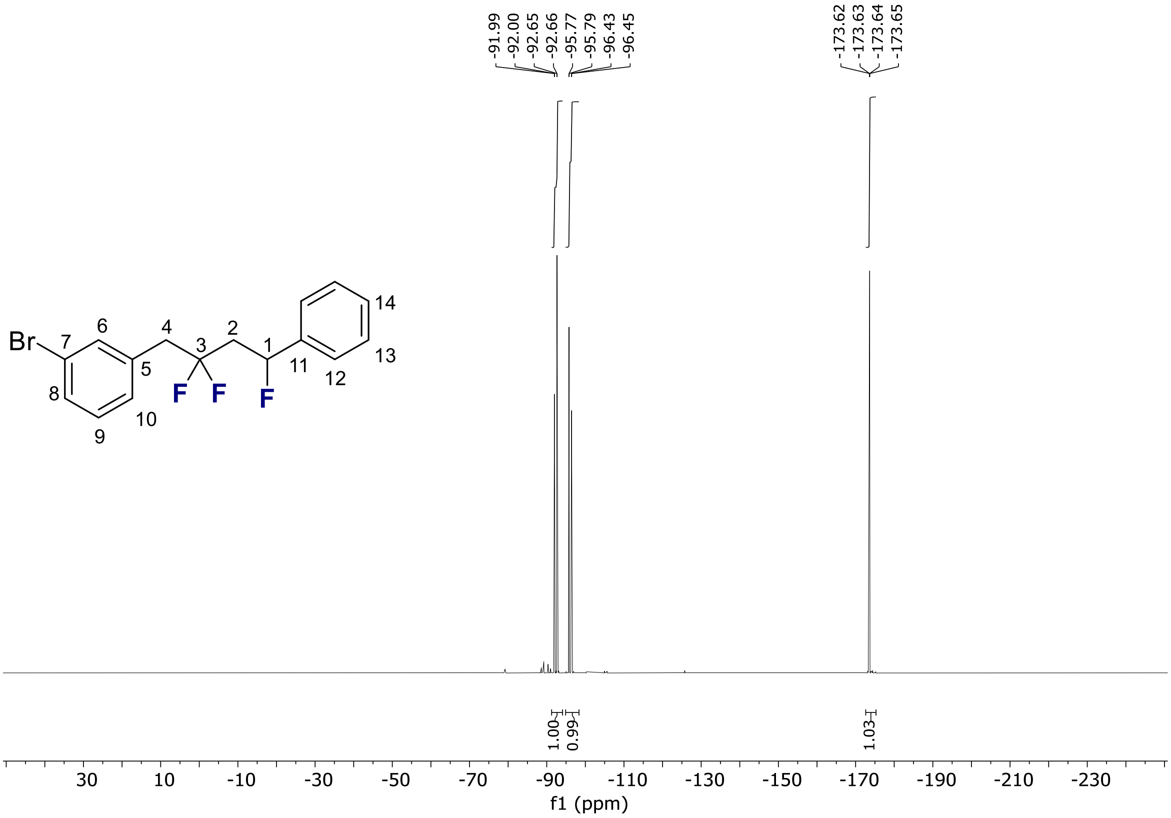


**Supplementary Figure 144.** $^{19}F\{^1H\}$ NMR of **2m** (376 MHz, 299 K, $CDCl_3$).

**1-Bromo-2-(2,2,4-trifluoro-4-phenylbutyl)benzene (2n)**

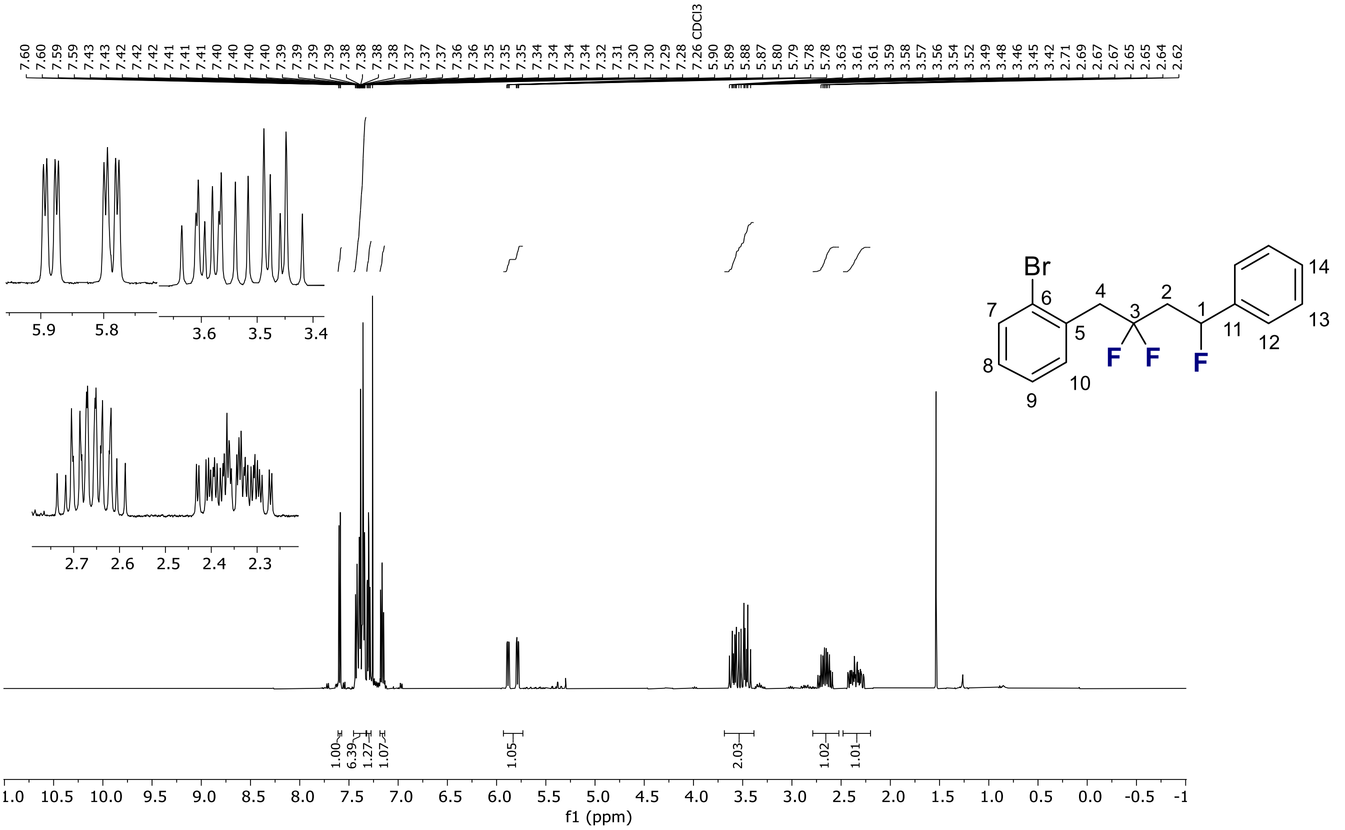

**Supplementary Figure 145.** $^{1}$H NMR of **2n** (500 MHz, 299 K, $CDCl_3$).

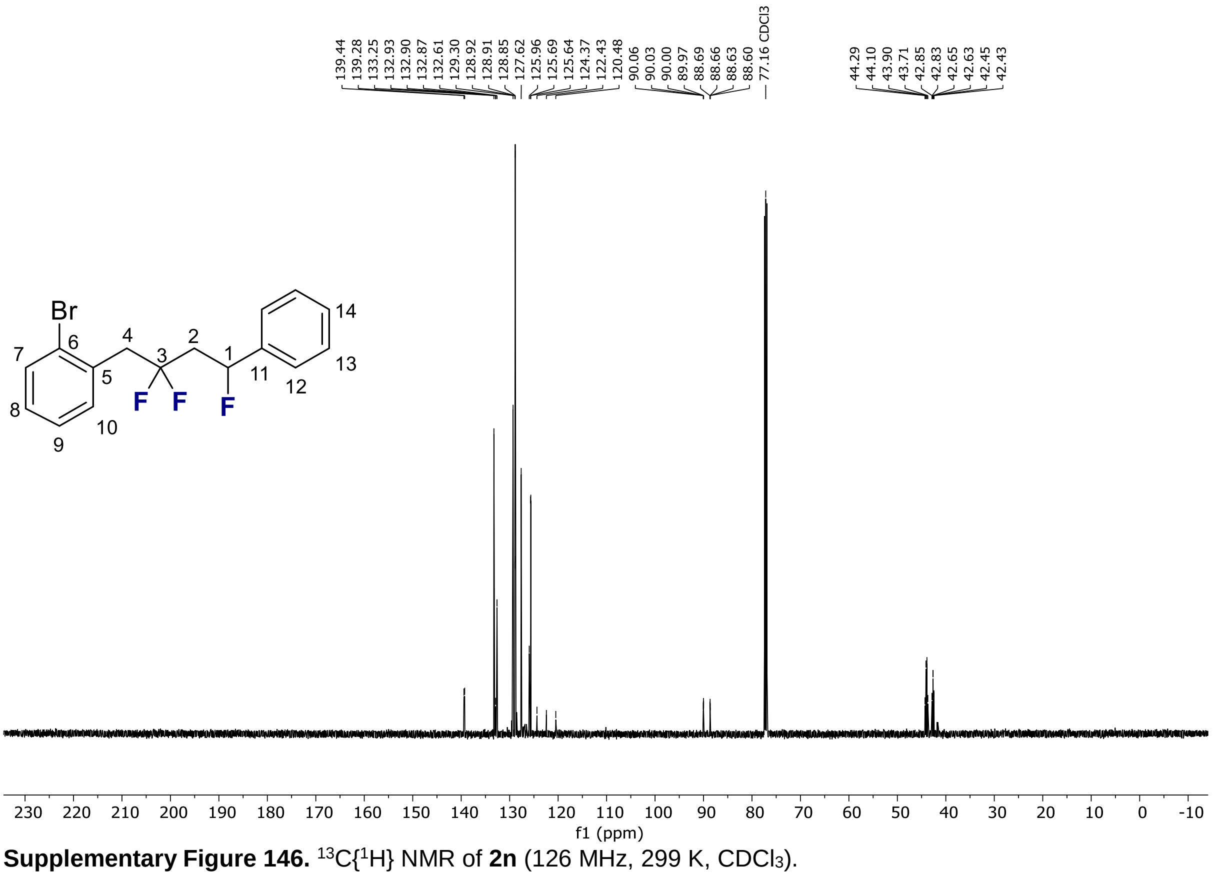

**Supplementary Figure 146.** $^{13}C\{^{1}H\}$ NMR of **2n** (126 MHz, 299 K, $CDCl_3$).

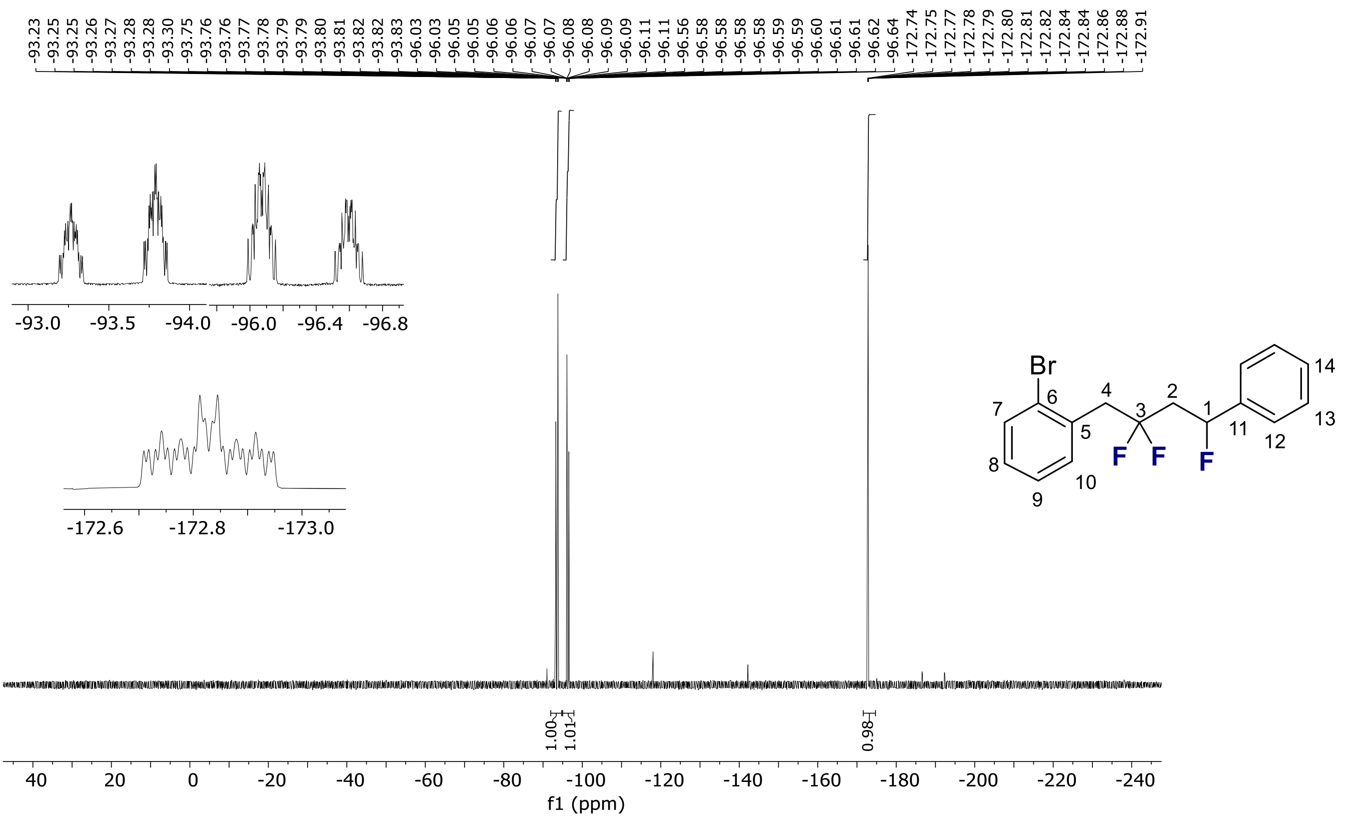


**Supplementary Figure 147.** $^{19}$F NMR of **2n** (470 MHz, 299 K, $CDCl_3$).

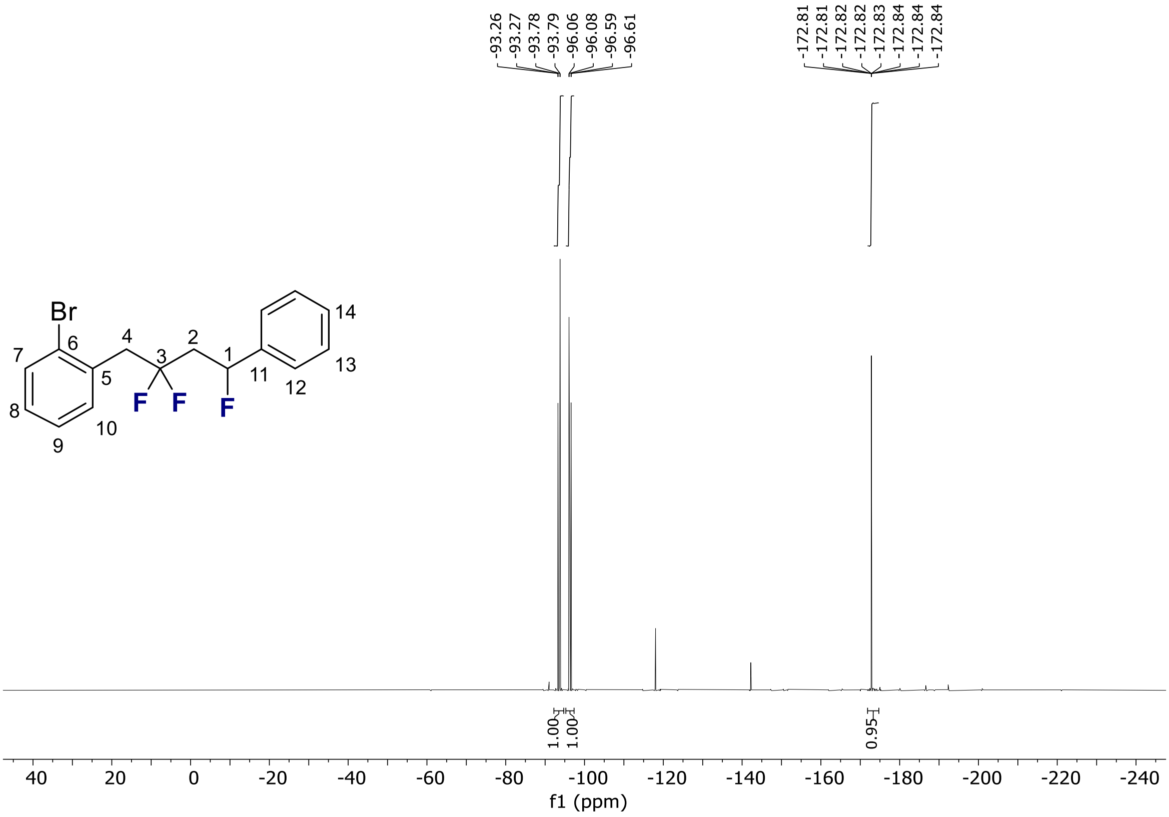


**Supplementary Figure 148.** $^{19}F\{^1H\}$ NMR of **2n** (470 MHz, 299 K, $CDCl_3$).

## 1-Fluoro-4-(1,3,3-trifluoro-4-phenylbutyl)benzene (2o)

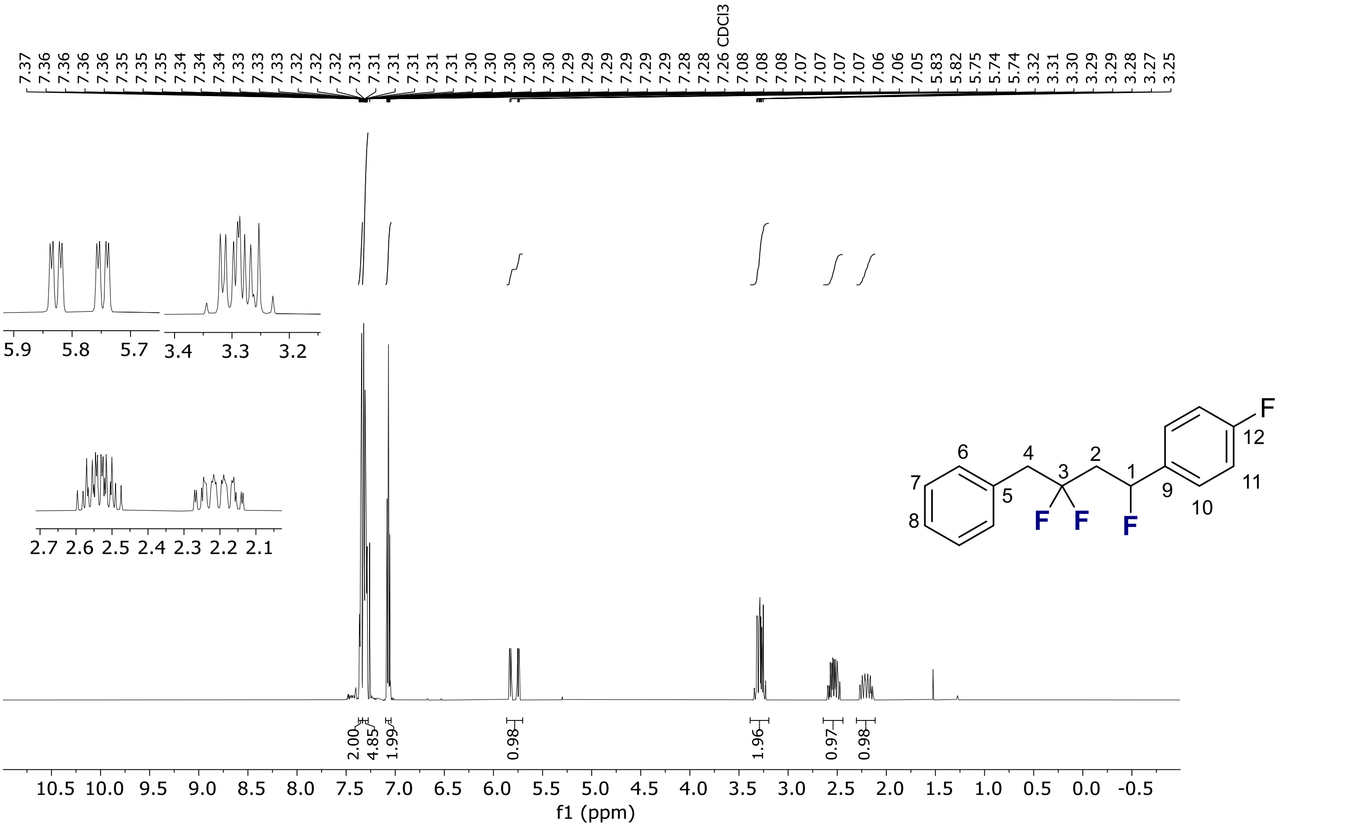


**Supplementary Figure 149.** $^{1}$H NMR of **2o** (599 MHz, 299 K, $CDCl_3$).

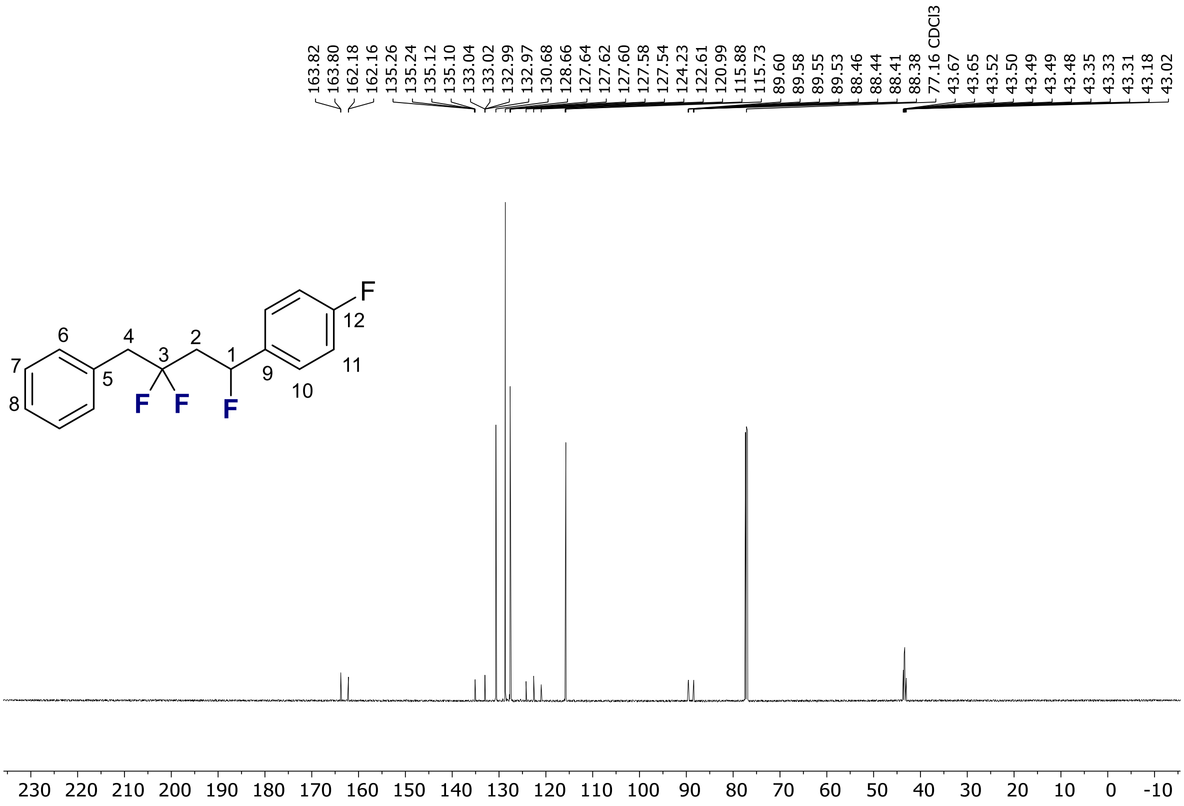


**Supplementary Figure 150.** $^{13}C\{^{1}H\}$ NMR of **2o** (151 MHz, 299 K, $CDCl_3$).

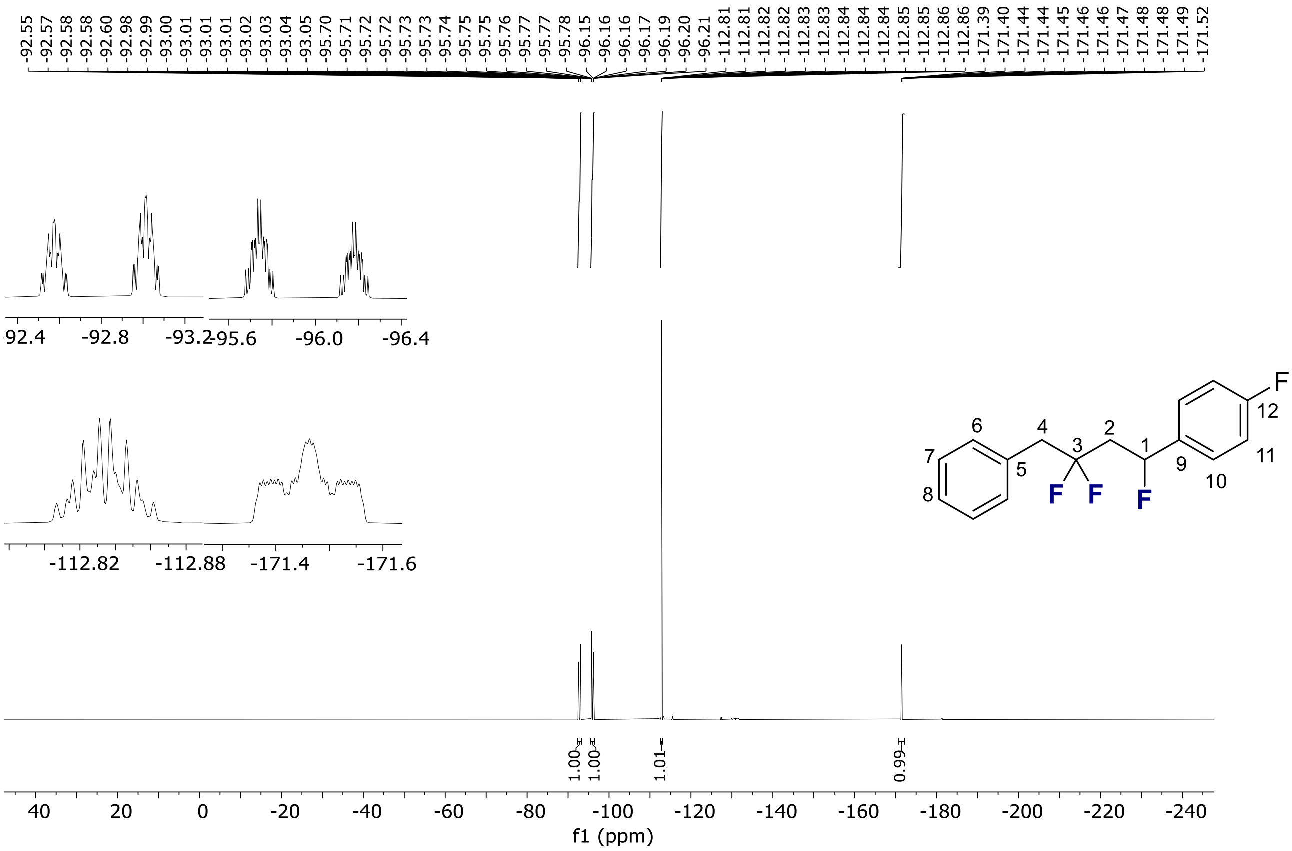

**Supplementary Figure 151.** $^{19}$F NMR of **2o** (564 MHz, 299 K, $CDCl_3$).

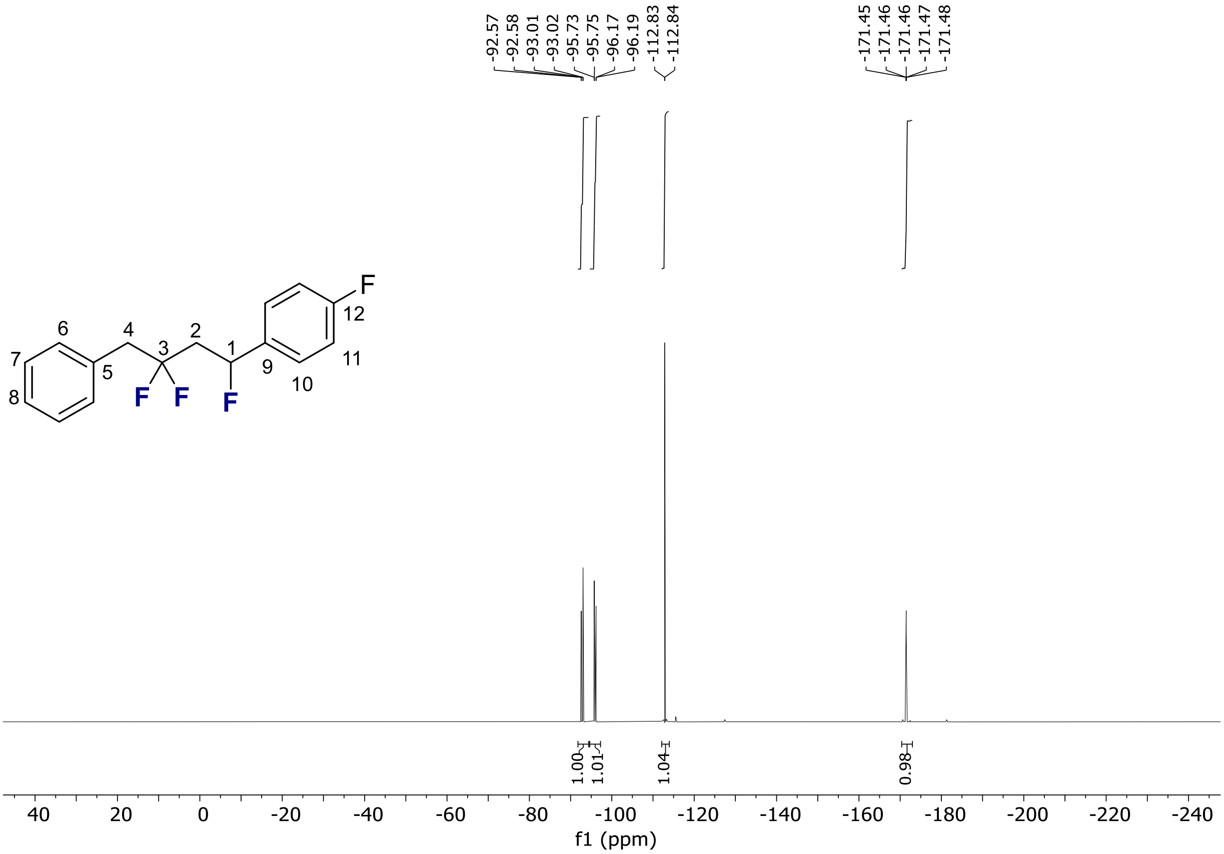

**Supplementary Figure 152.** $^{19}$F{$^1$H} NMR of **2o** (564 MHz, 299 K, $CDCl_3$).

## 1-Chloro-4-(1,3,3-trifluoro-4-phenylbutyl)benzene (2p)

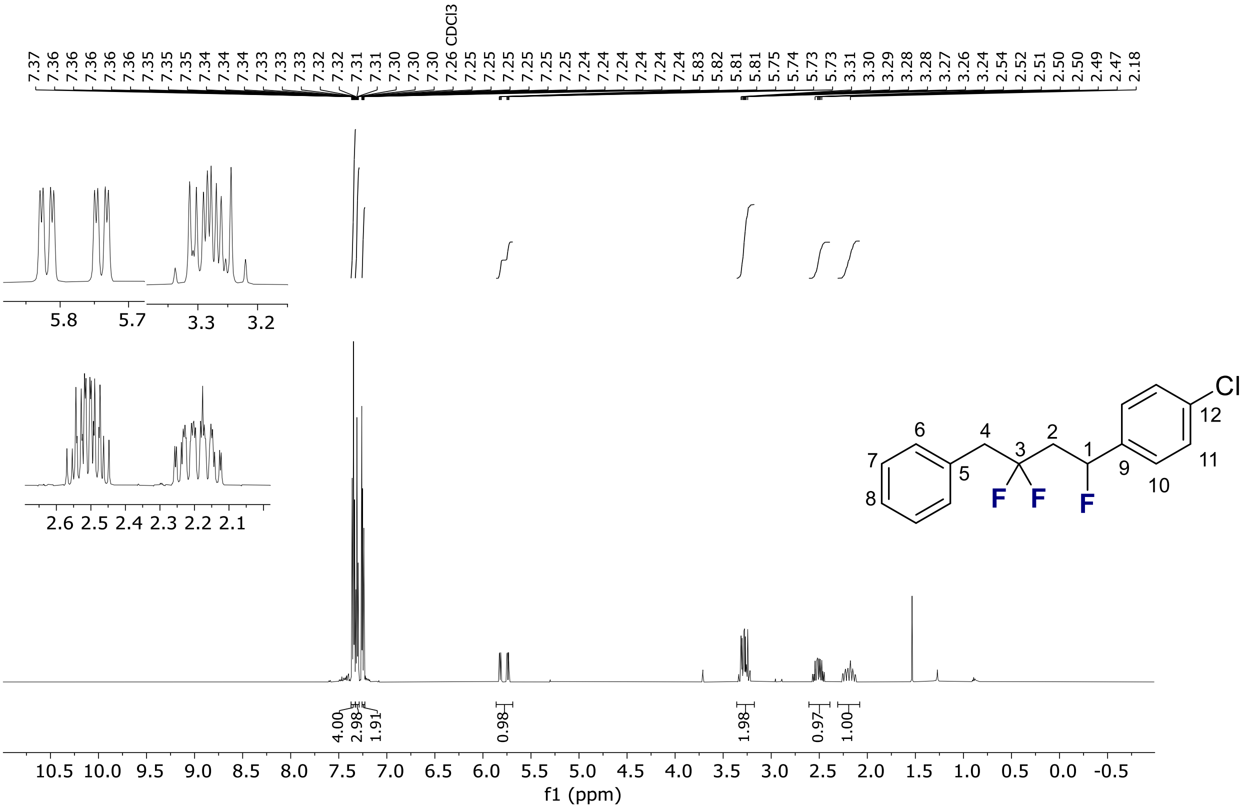


**Supplementary Figure 153.** $^{1}$H NMR of **2p** (599 MHz, 299 K, $CDCl_3$).

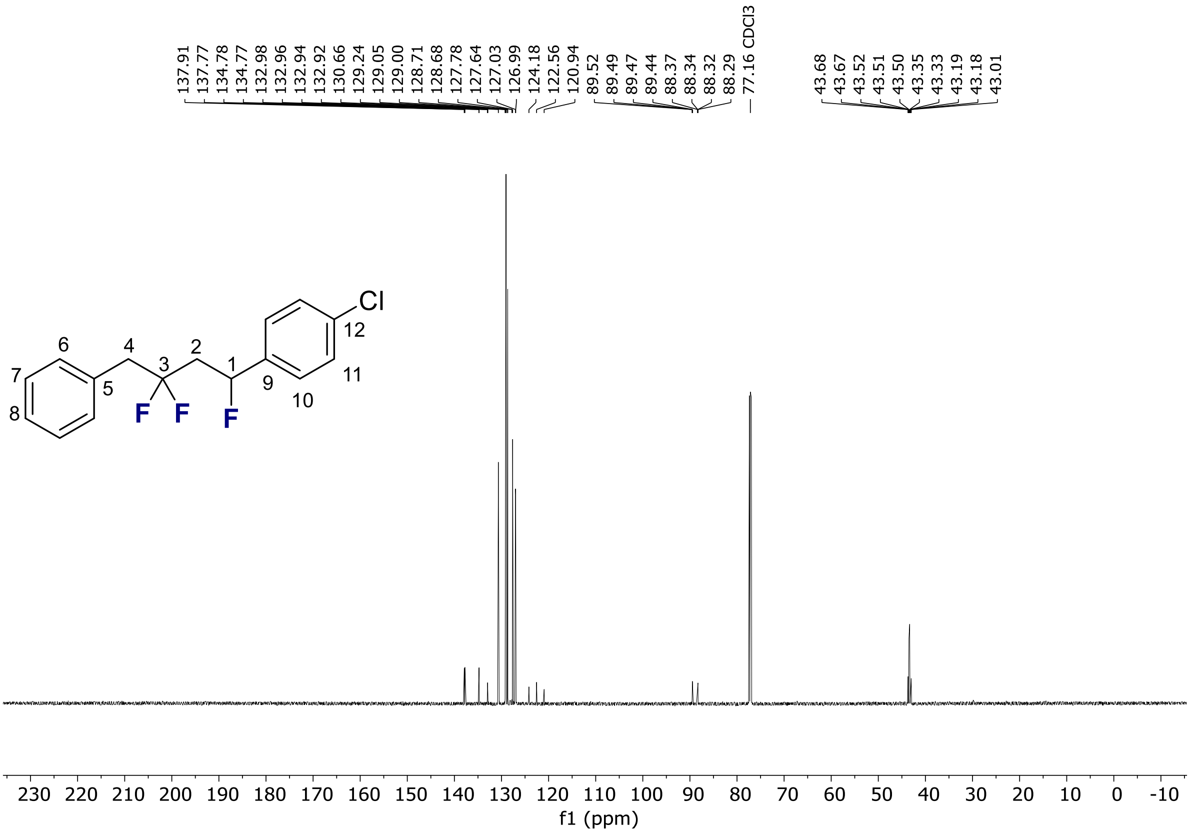


**Supplementary Figure 154.** $^{13}C\{^{1}H\}$ NMR of **2p** (151 MHz, 299 K, $CDCl_3$).

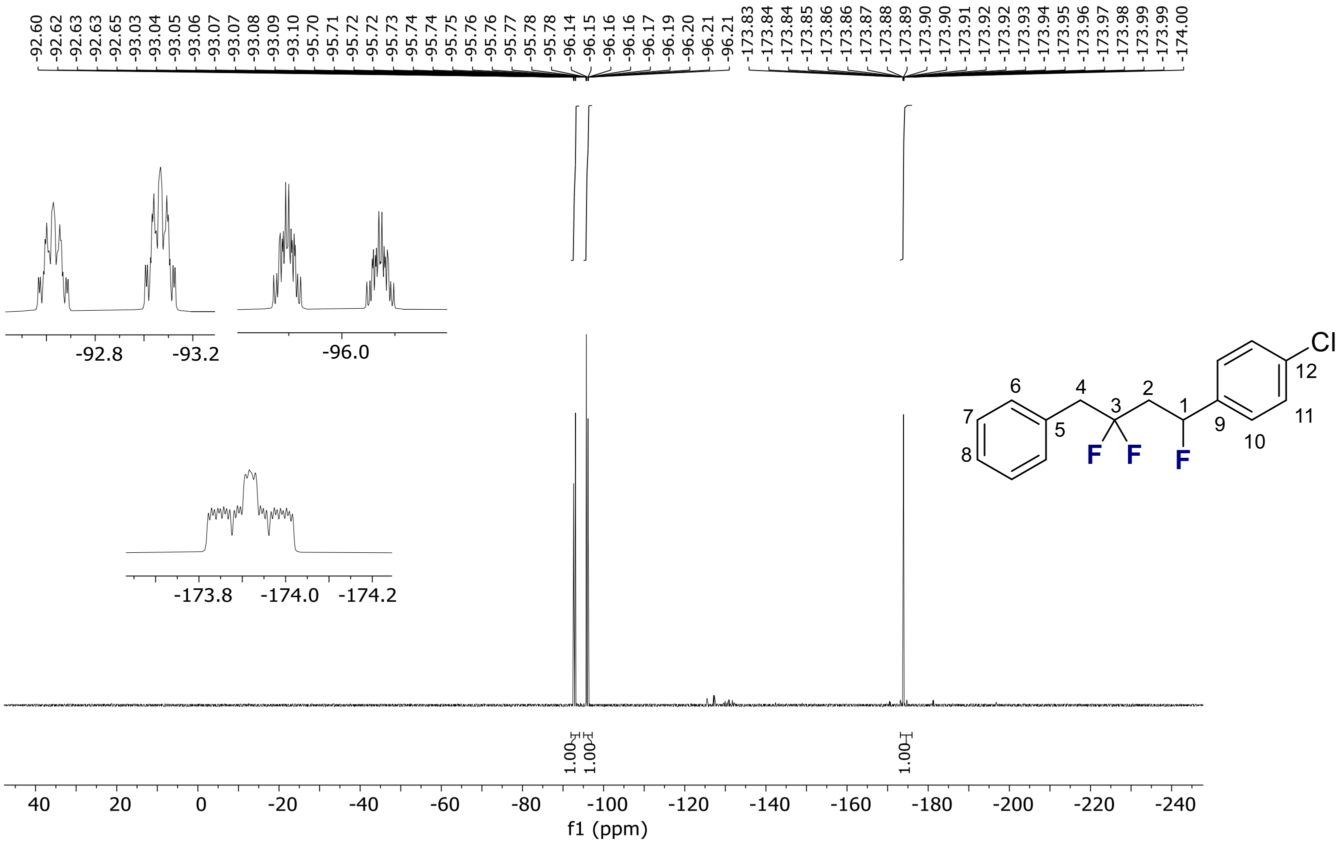


**Supplementary Figure 155.** $^{19}$F NMR of **2p** (564 MHz, 299 K, $CDCl_3$).

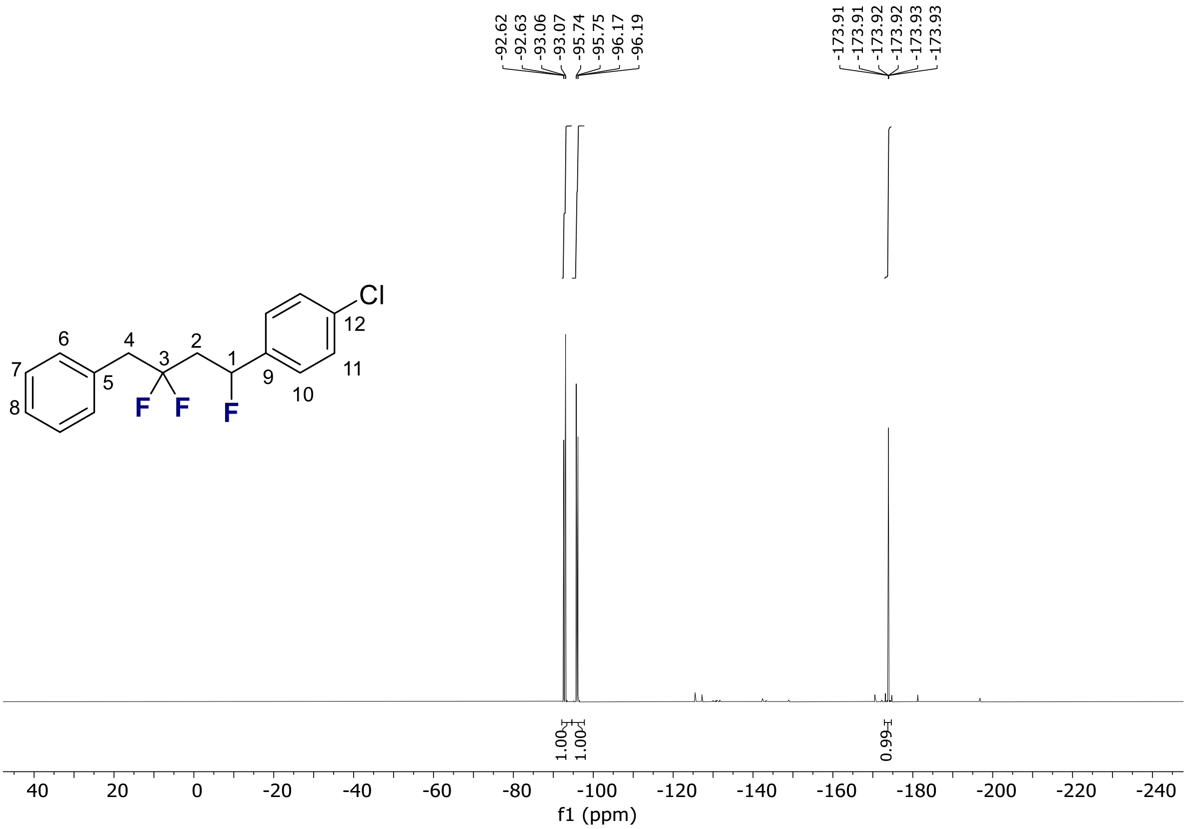


**Supplementary Figure 156.** $^{19}$F{$^1$H} NMR of **2p** (564 MHz, 299 K, $CDCl_3$).

## 1-Bromo-4-(1,3,3-trifluoro-4-phenylbutyl)benzene (2q)

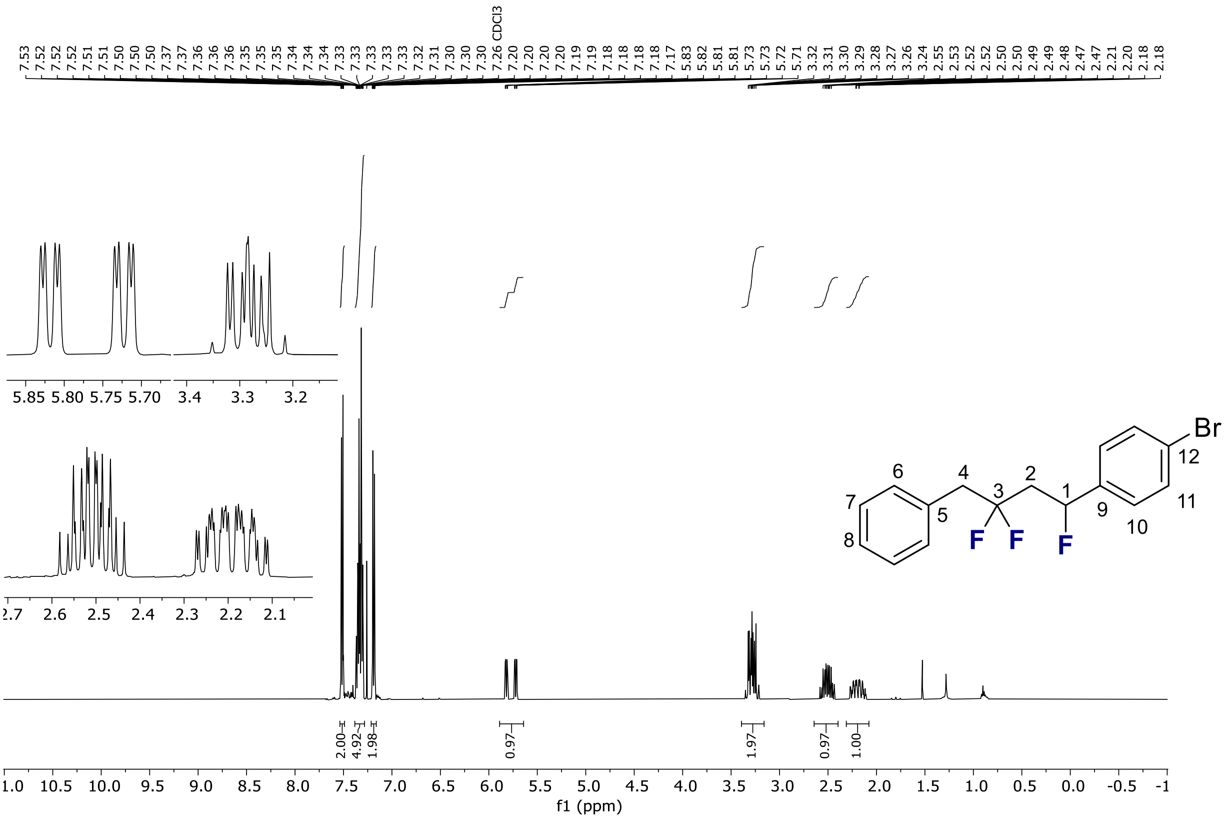


**Supplementary Figure 157.** $^{1}$H NMR of **2q** (500 MHz, 299 K, $CDCl_3$).

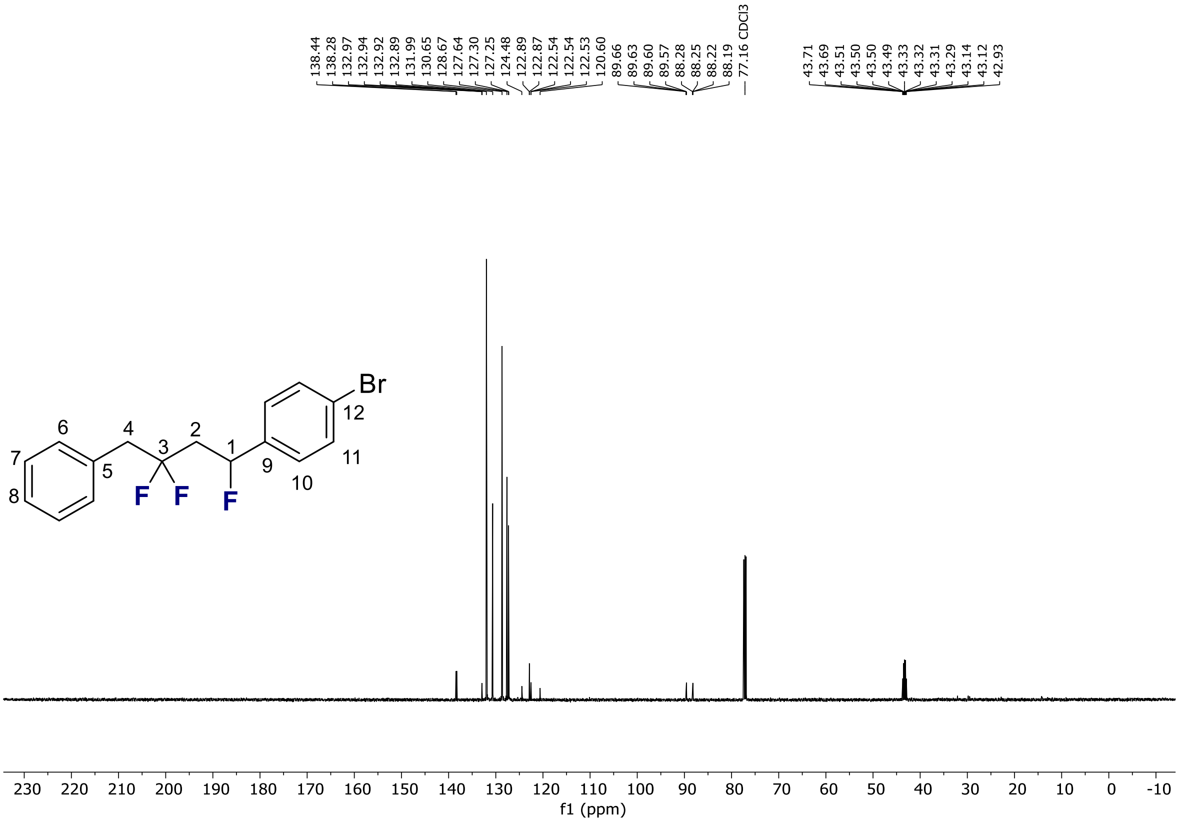


**Supplementary Figure 158.** $^{13}C\{^{1}H\}$ NMR of **2q** (126 MHz, 299 K, $CDCl_3$).

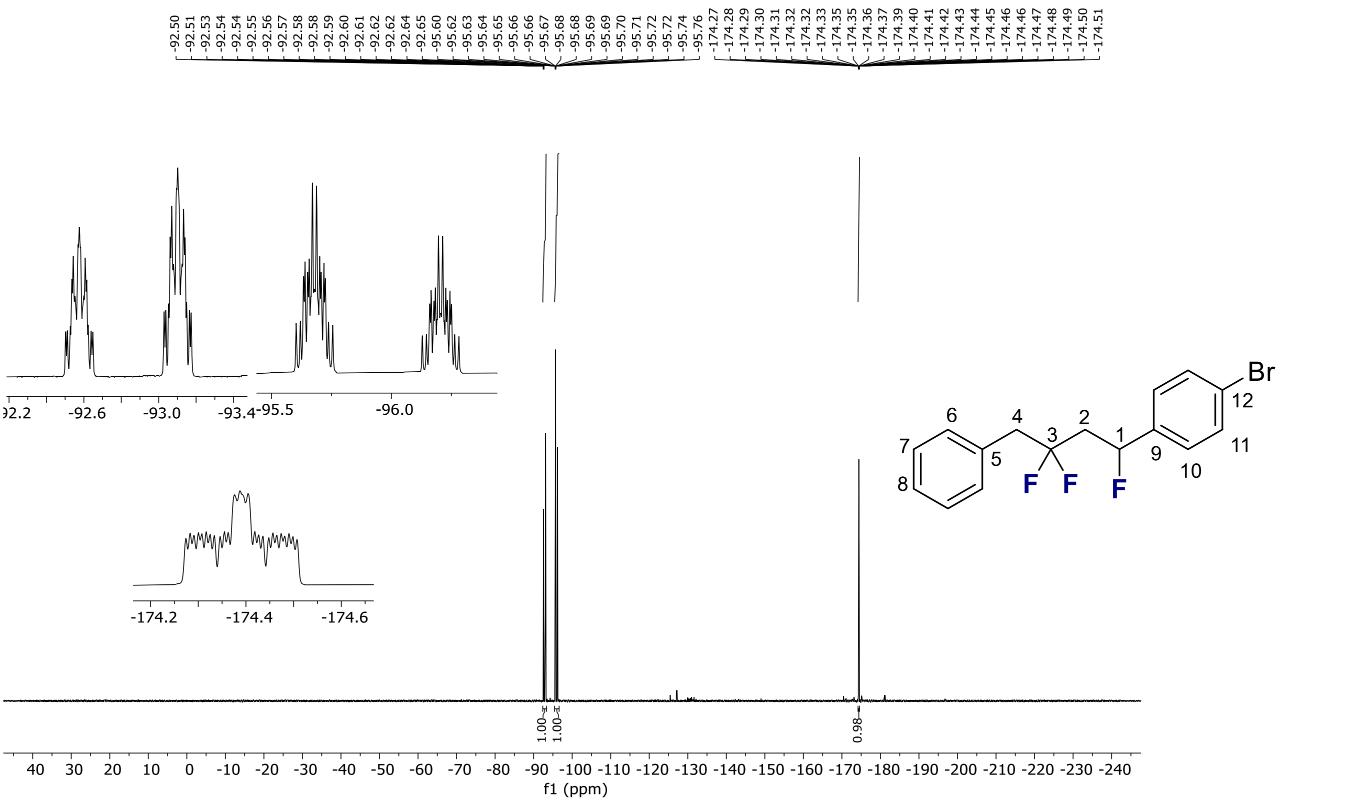

**Supplementary Figure 159.** $^{19}$F NMR of **2q** (470 MHz, 299 K, $CDCl_3$).

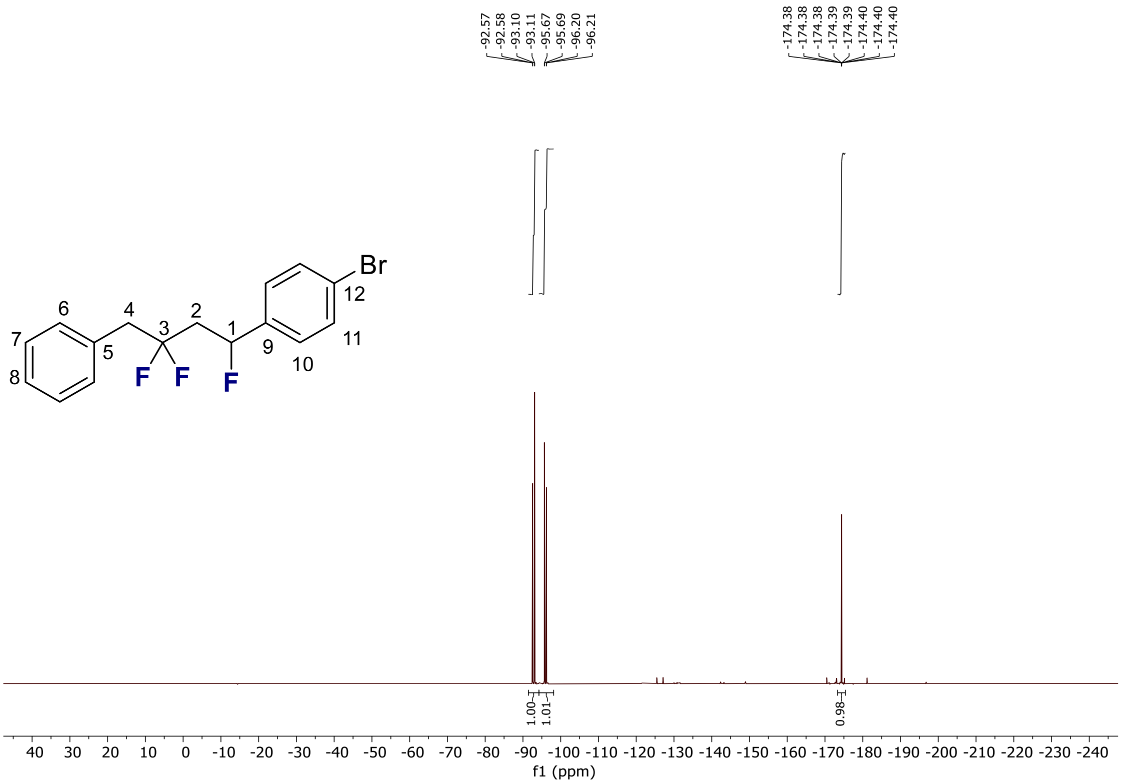

**Supplementary Figure 160.** $^{19}$F{$^{1}$H} NMR of **2q** (470 MHz, 299 K, $CDCl_3$).

## 1-(1,3,3-Trifluoro-4-phenylbutyl)-4-(trifluoromethyl)benzene (2r)

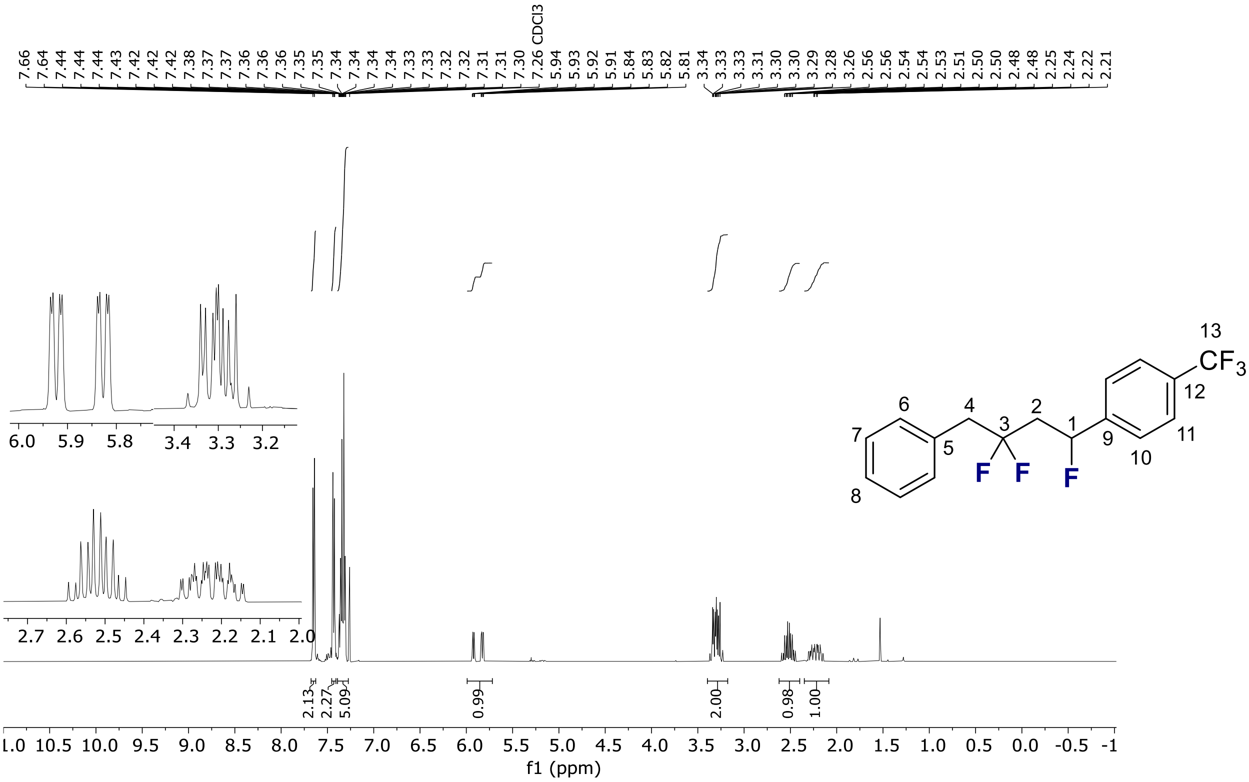


**Supplementary Figure 161.** $^{1}$H NMR of **2r** (500 MHz, 299 K, $CDCl_3$).

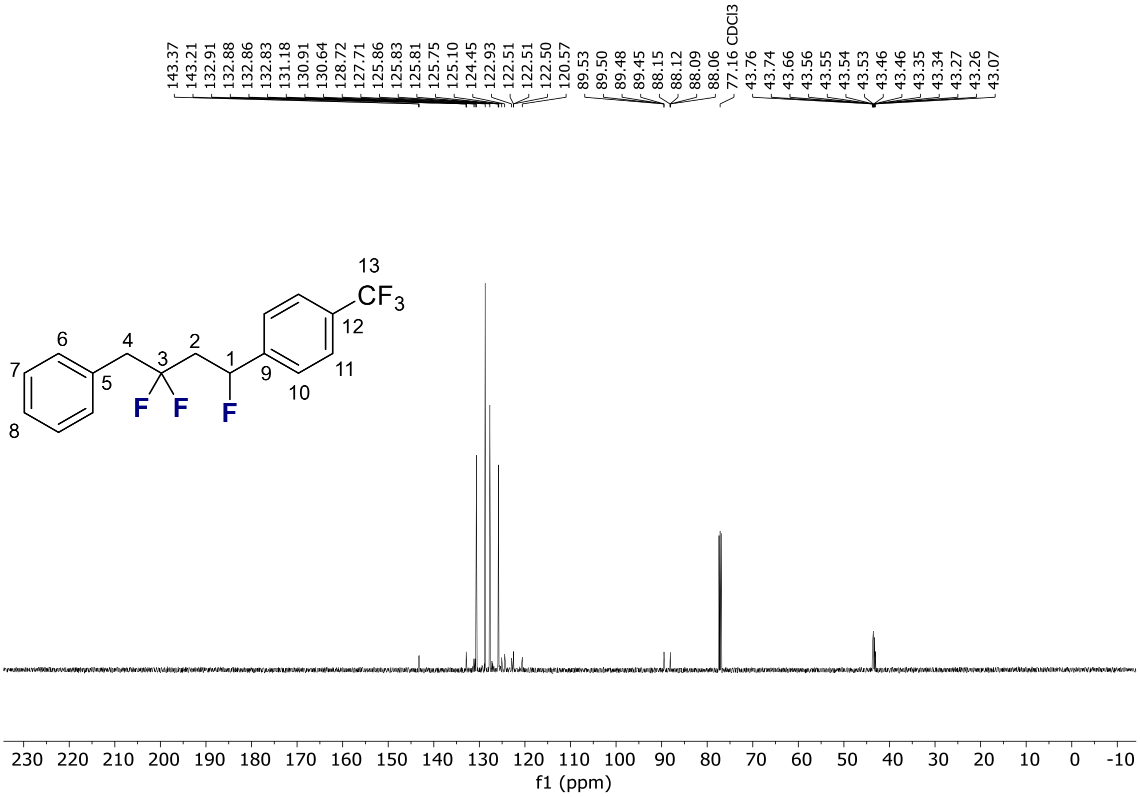


**Supplementary Figure 162.** $^{13}C\{^{1}H\}$ NMR of **2r** (126 MHz, 299 K, $CDCl_3$).

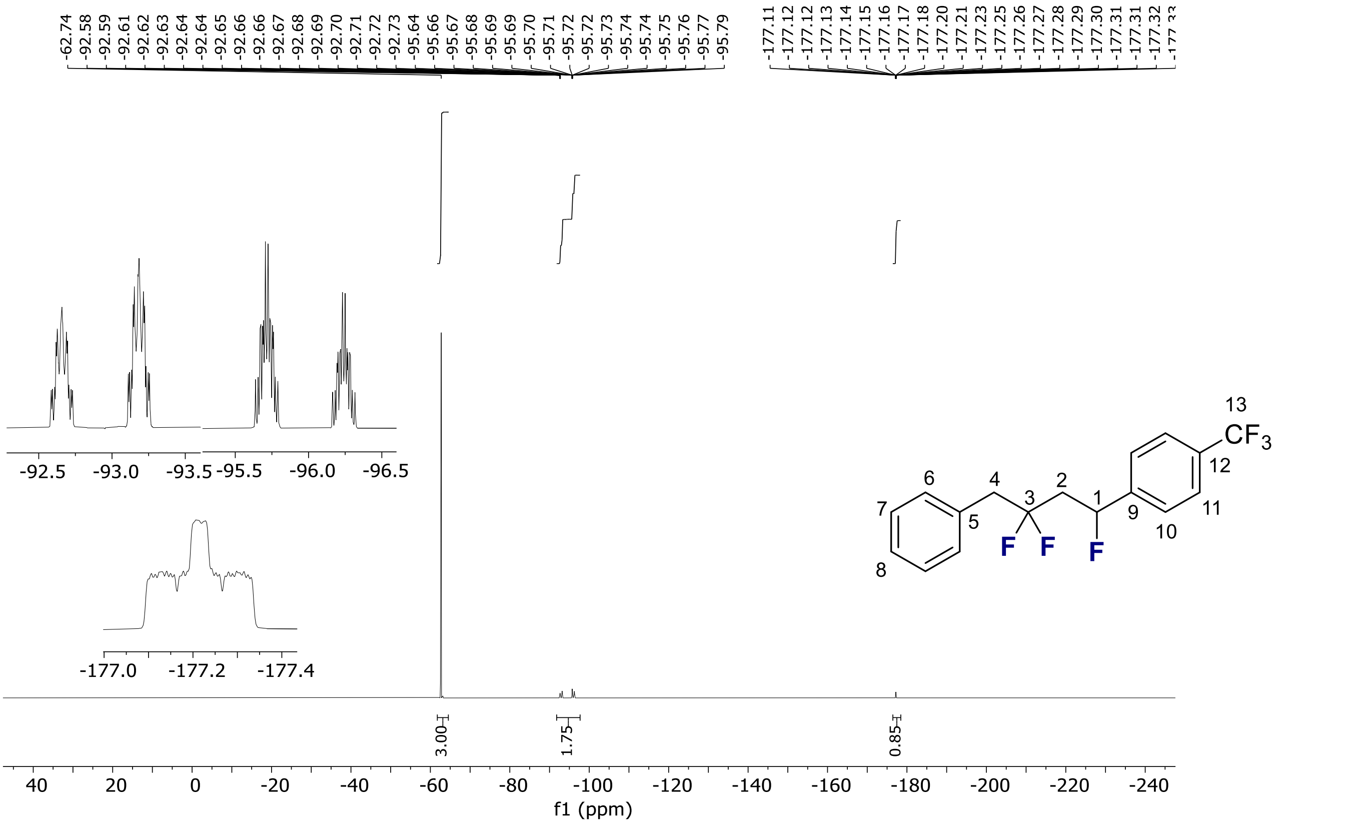

**Supplementary Figure 163.** $^{19}F$ NMR of **2r** (470 MHz, 299 K, $CDCl_3$).

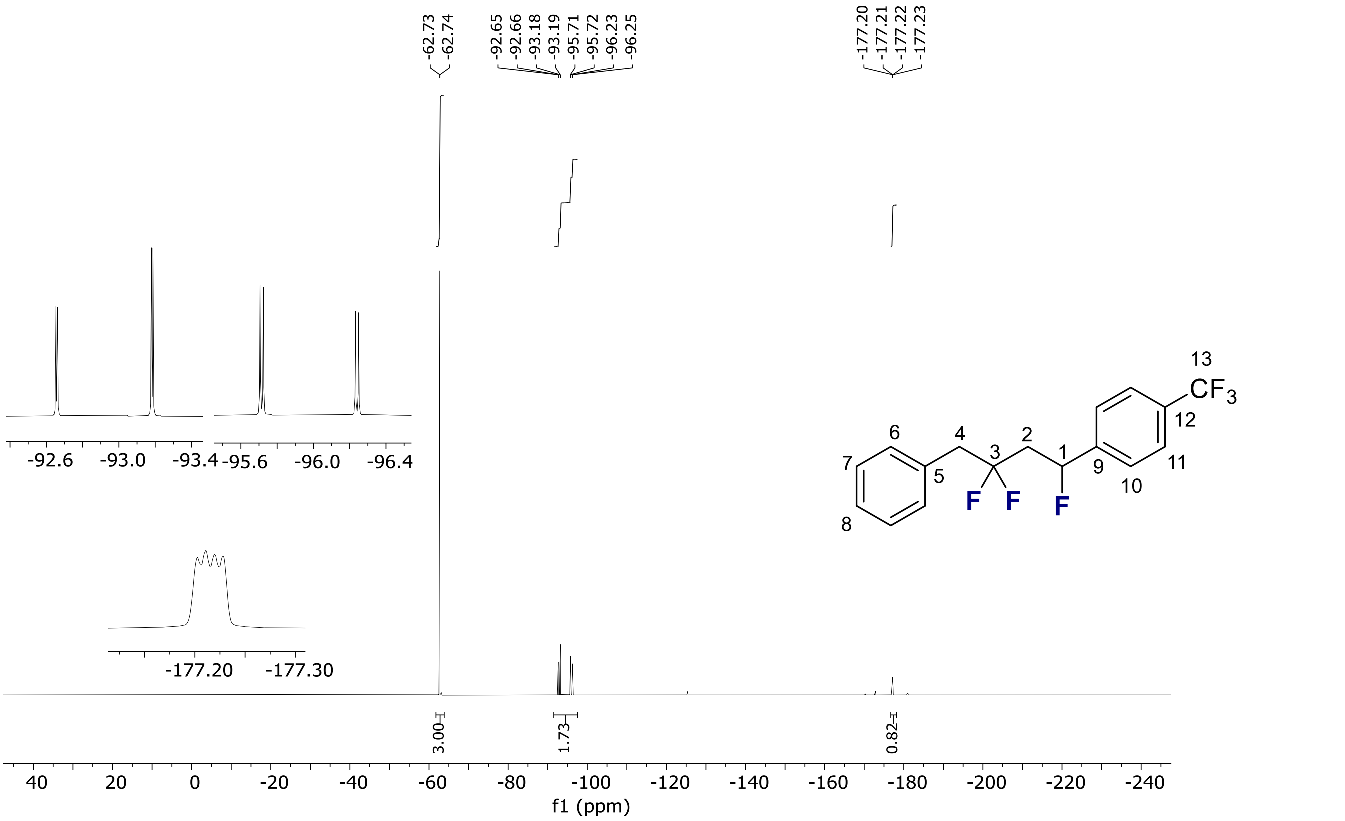

**Supplementary Figure 164.** $^{19}F\{^1H\}$ NMR of **2r** (470 MHz, 299 K, $CDCl_3$).

## 4-(1,3,3-Trifluoro-4-phenylbutyl)phenyl trifluoromethanesulfonate (2s)

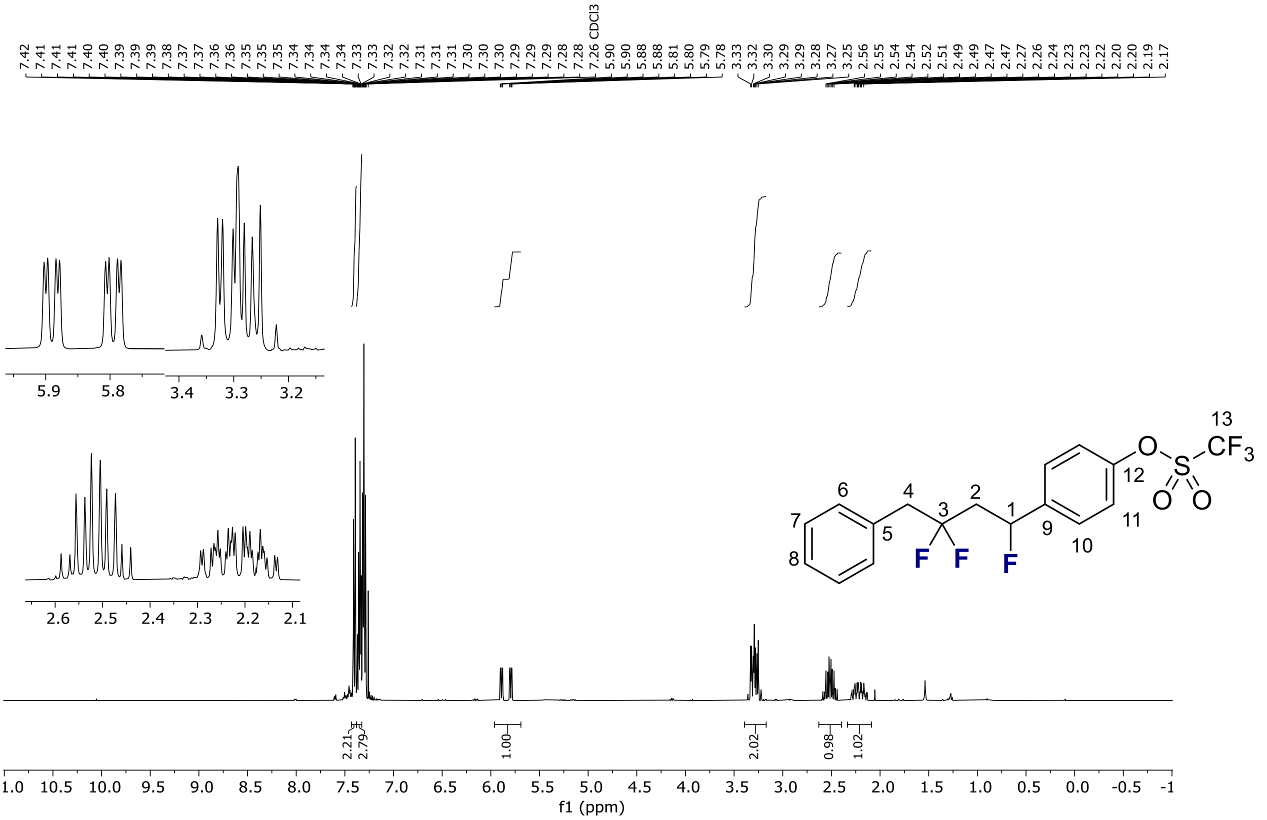


**Supplementary Figure 165.** $^{1}$H NMR of **2s** (500 MHz, 299 K, $CDCl_3$).

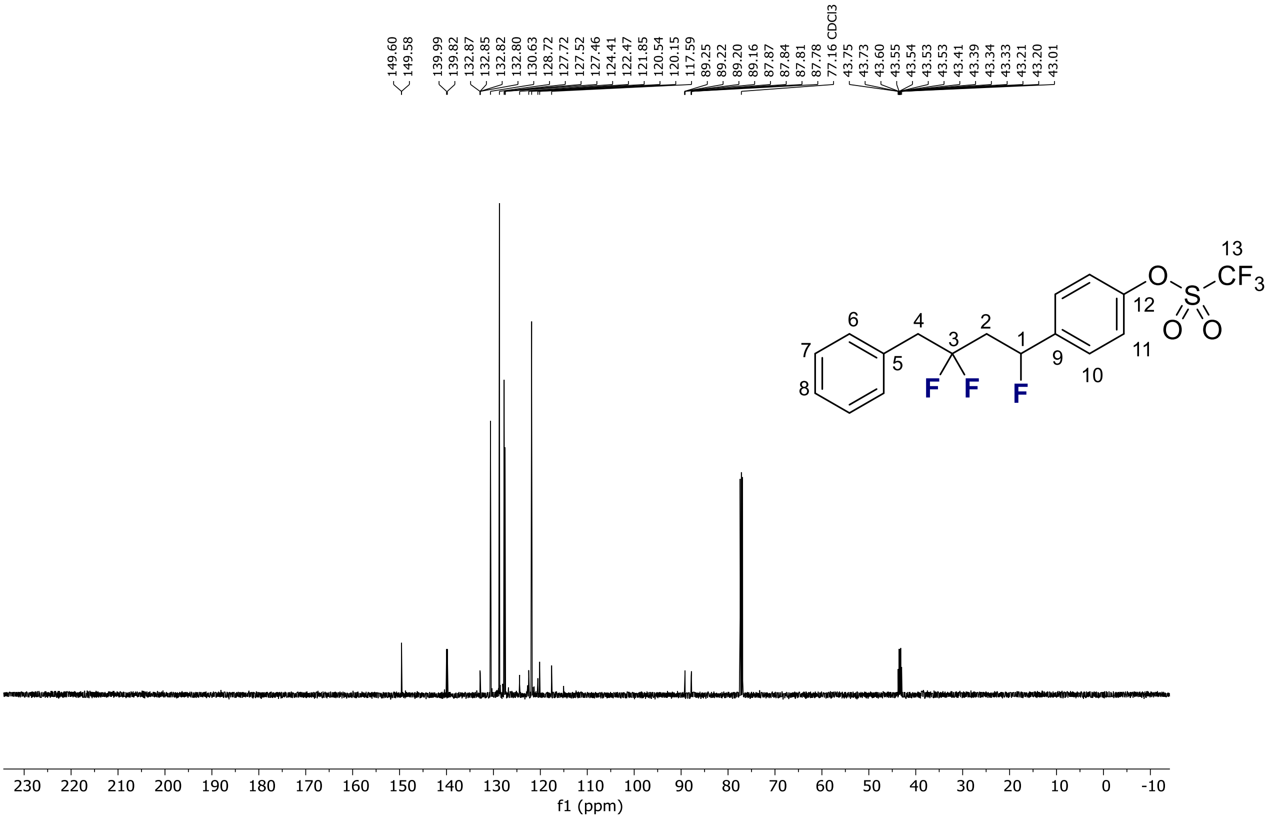


**Supplementary Figure 166.** $^{13}$C{$^{1}$H} NMR of **2s** (126 MHz, 299 K, $CDCl_3$).

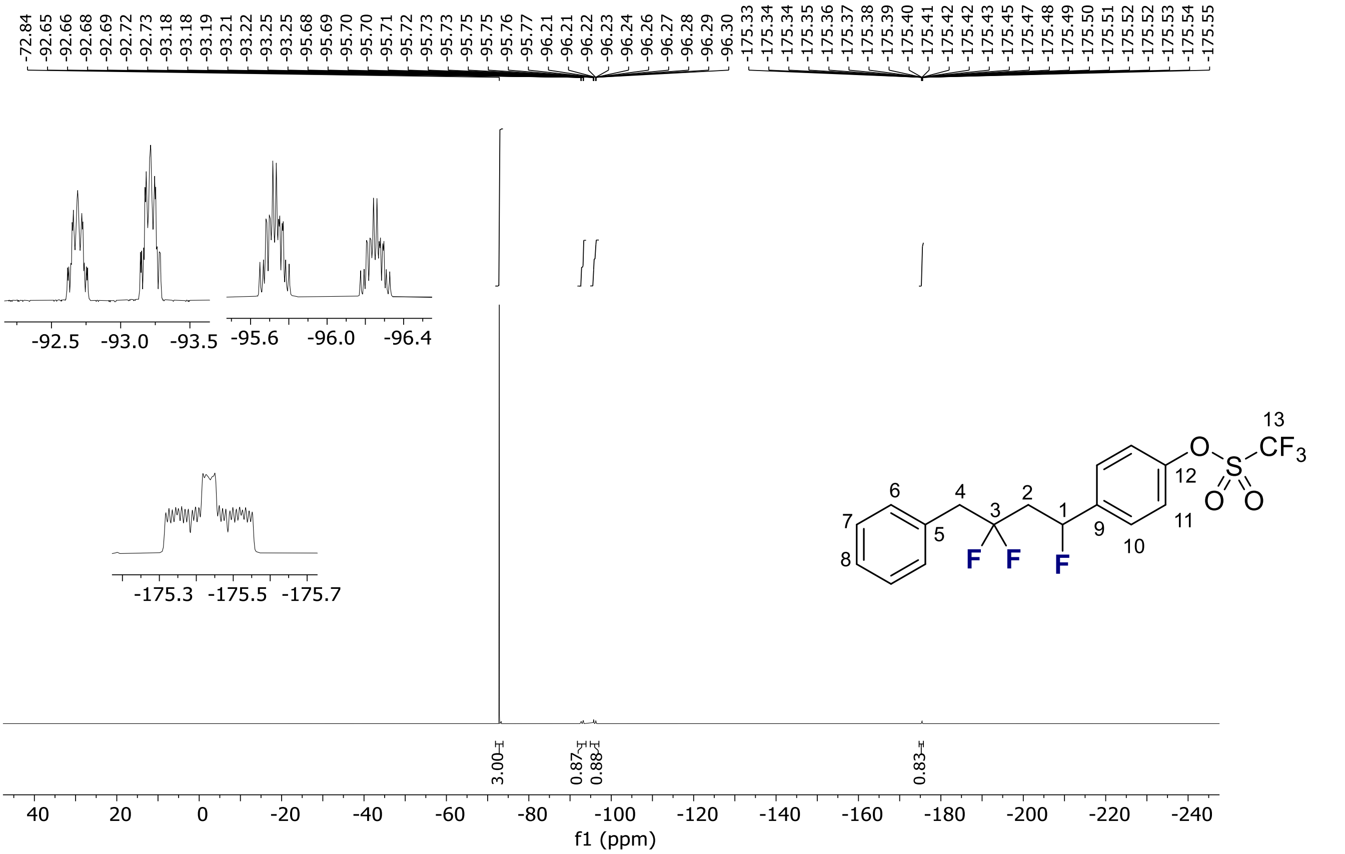

**Supplementary Figure 167.** $^{19}$F NMR of **2s** (470 MHz, 299 K, $CDCl_3$).

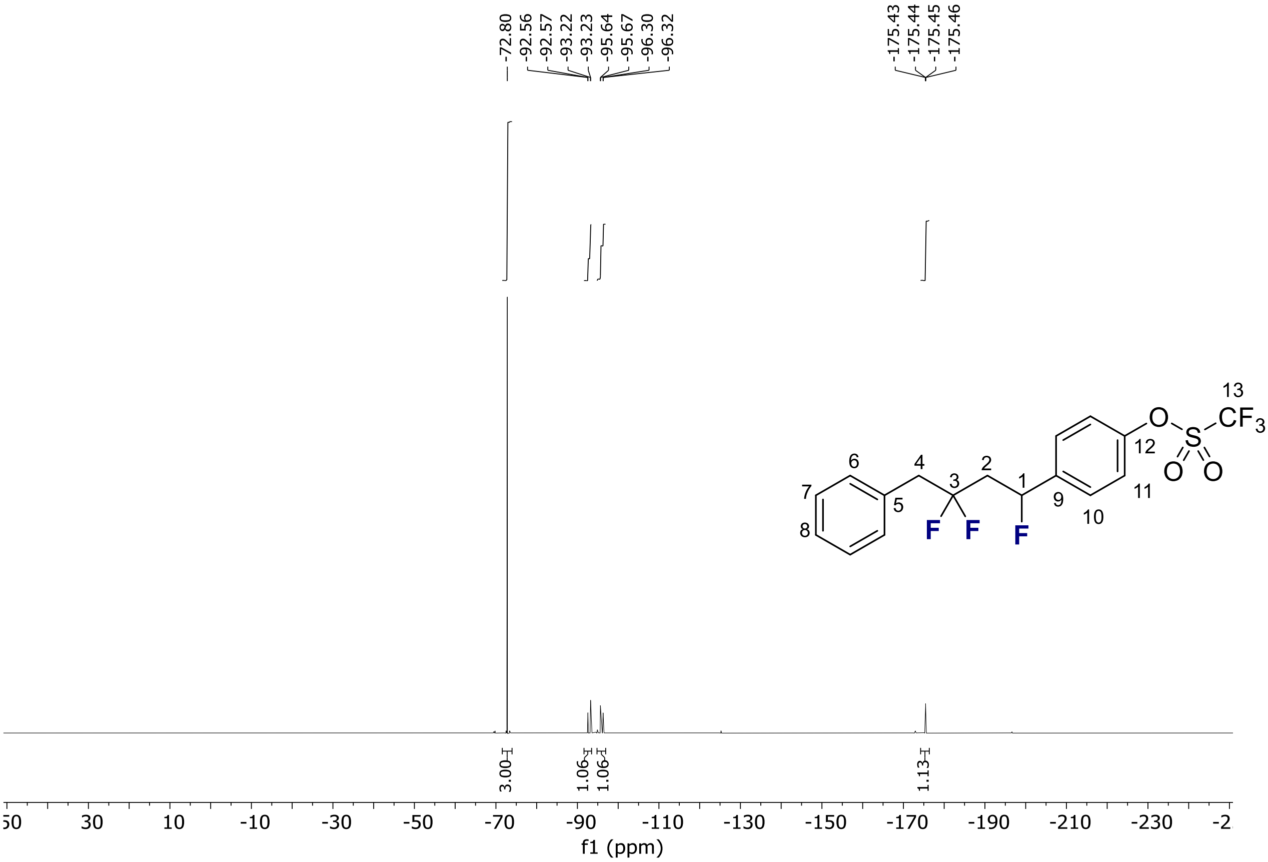

**Supplementary Figure 168.** $^{19}$F{$^{1}$H} NMR of **2s** (376 MHz, 299 K, $CDCl_3$).

## 1-Bromo-4-(2,2,4-trifluoro-4-(4-fluorophenyl)butyl)benzene (2t)

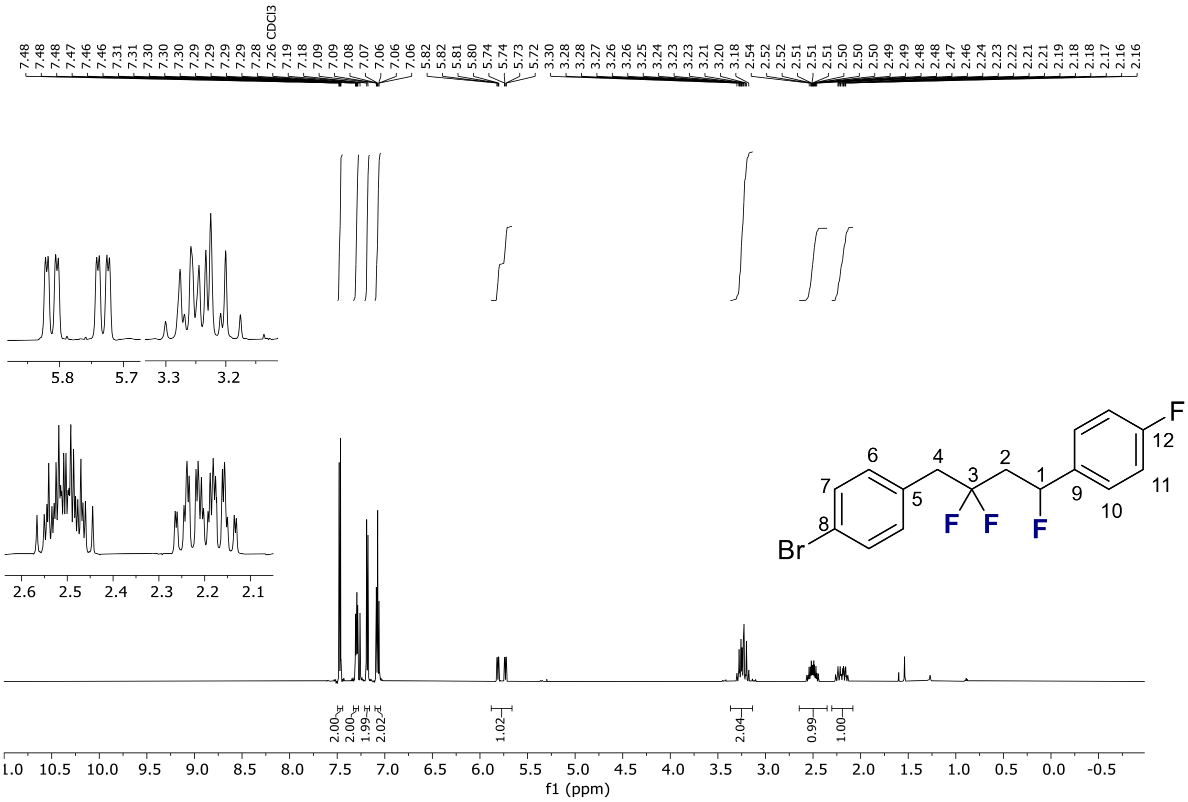


**Supplementary Figure 169.** $^{1}$H NMR of **2t** (599 MHz, 299 K, $CDCl_3$).

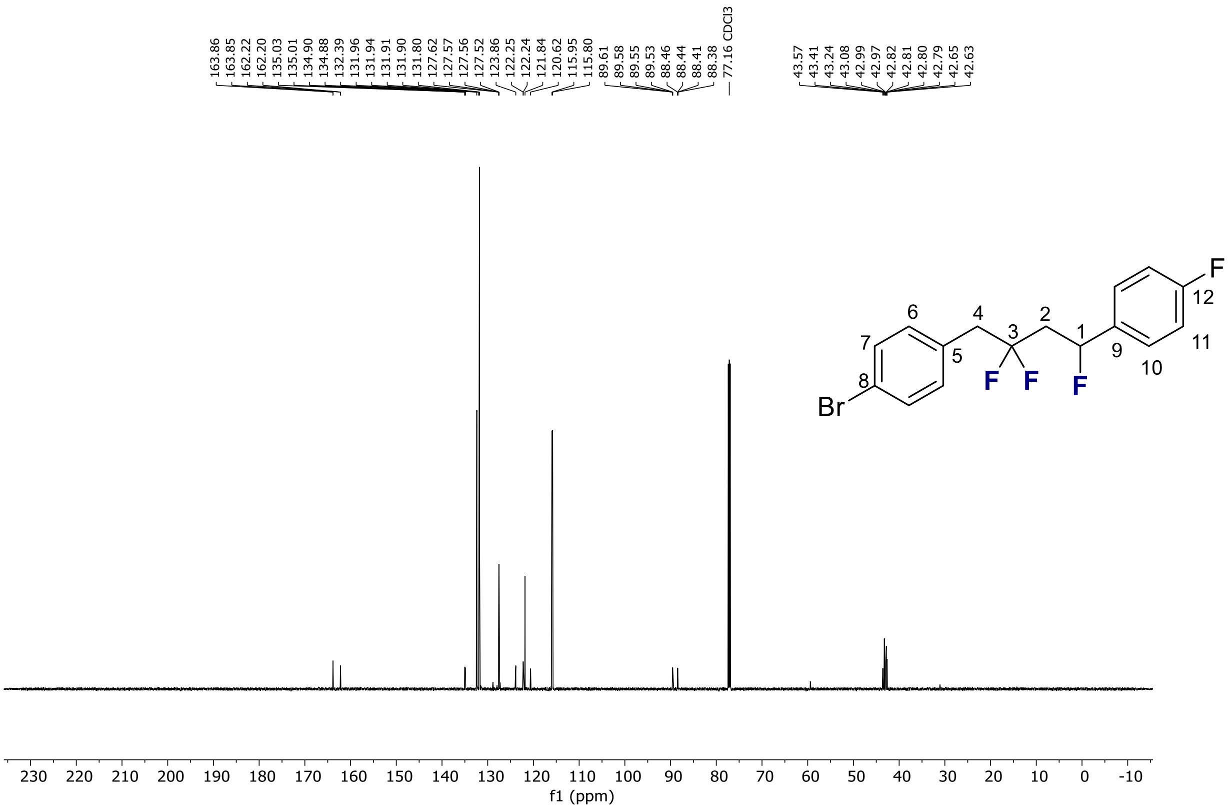


**Supplementary Figure 170.** $^{13}$C{$^{1}$H} NMR of **2t** (151 MHz, 299 K, $CDCl_3$).

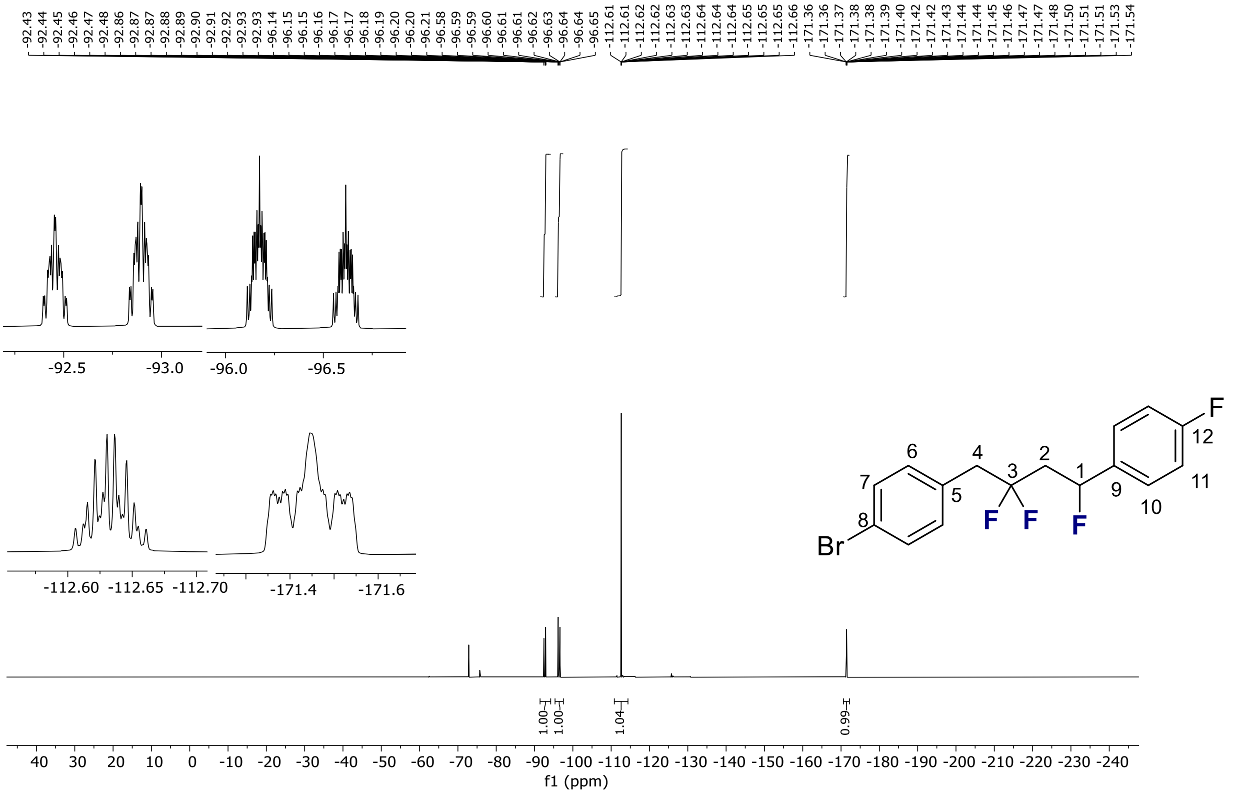


**Supplementary Figure 171.** $^{19}F$ NMR of **2t** (564 MHz, 299 K, $CDCl_3$).

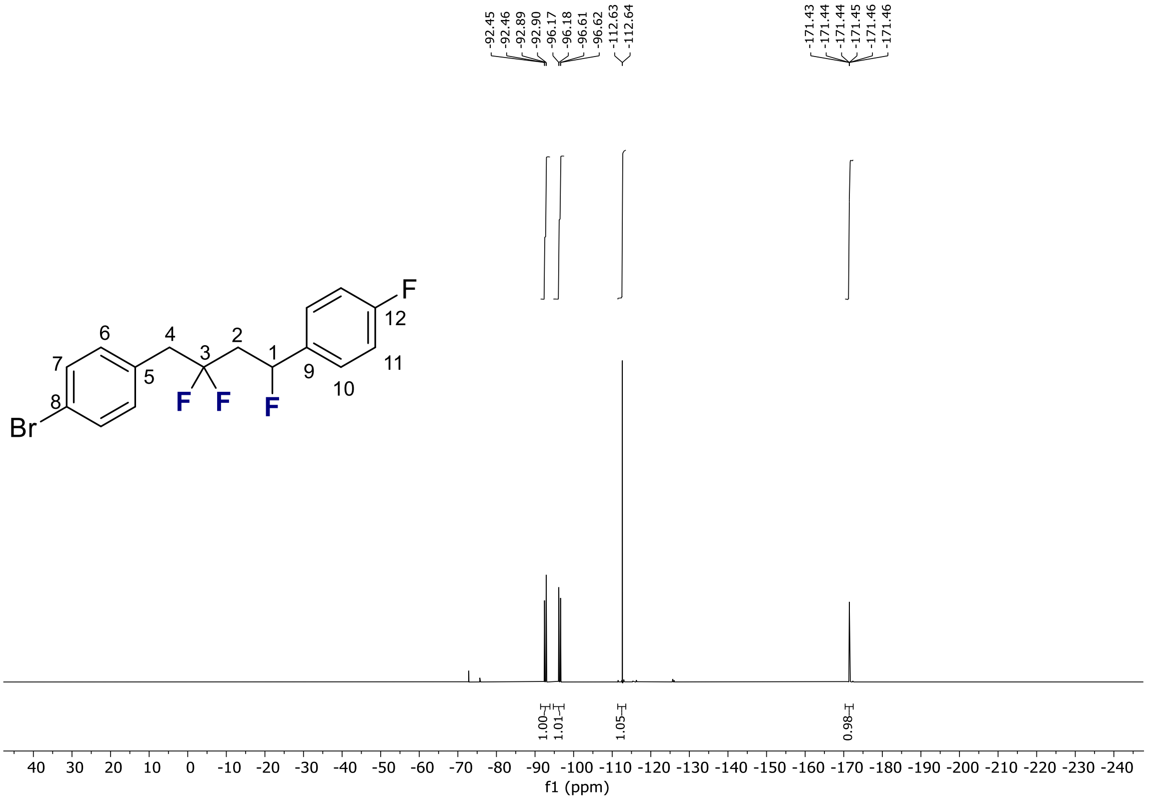


**Supplementary Figure 172.** $^{19}F\{^1H\}$ NMR of **2t** (564 MHz, 299 K, $CDCl_3$).

**1-Bromo-4-(4-(4-chlorophenyl)-2,2,4-trifluorobutyl)benzene (2u)**

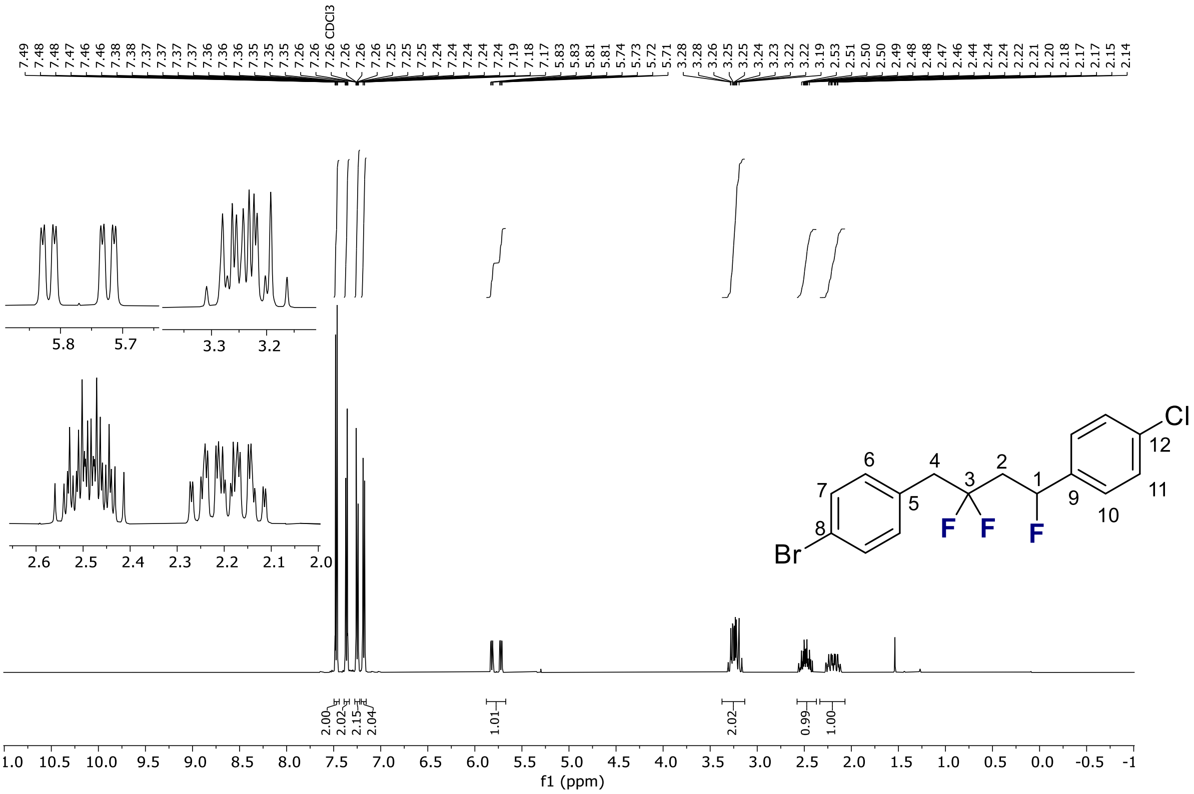

**Supplementary Figure 173.** $^{1}H$ NMR of **2u** (500 MHz, 299 K, $CDCl_3$).

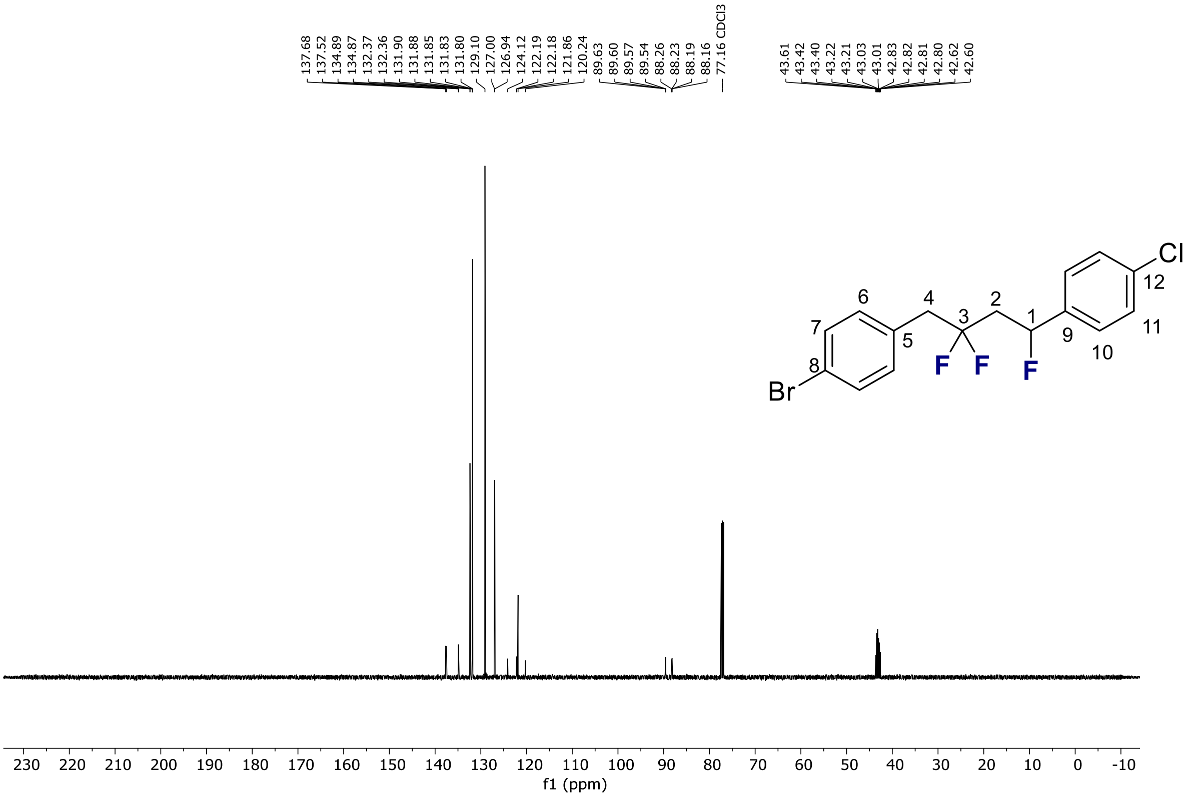

**Supplementary Figure 174.** $^{13}C\{^{1}H\}$ NMR of **2u** (126 MHz, 299 K, $CDCl_3$).

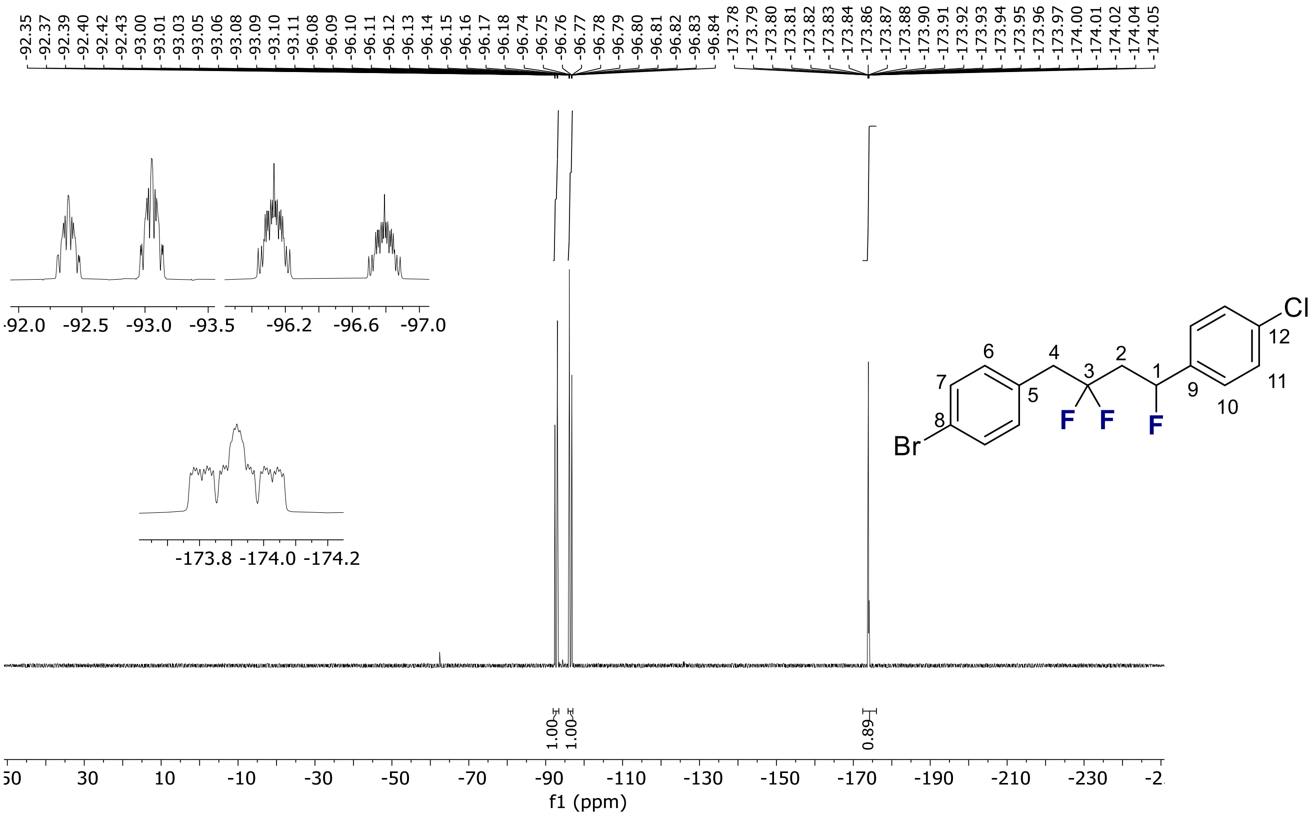

**Supplementary Figure 175.** $^{19}F$ NMR of **2u** (376 MHz, 299 K, $CDCl_3$).

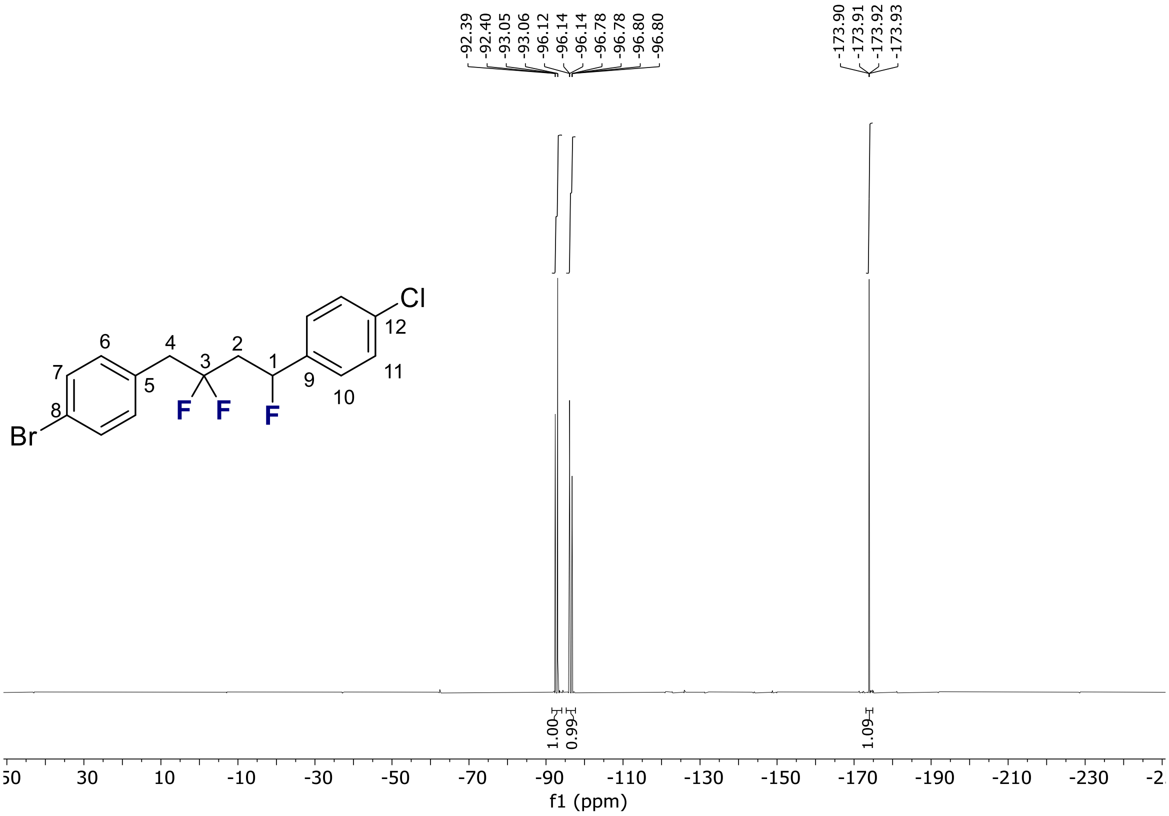

**Supplementary Figure 176.** $^{19}F\{^1H\}$ NMR of **2u** (376 MHz, 299 K, $CDCl_3$).

## 1-Bromo-4-(2,2,4-trifluoro-4-(4-(trifluoromethyl)phenyl)butyl)benzene (2v)

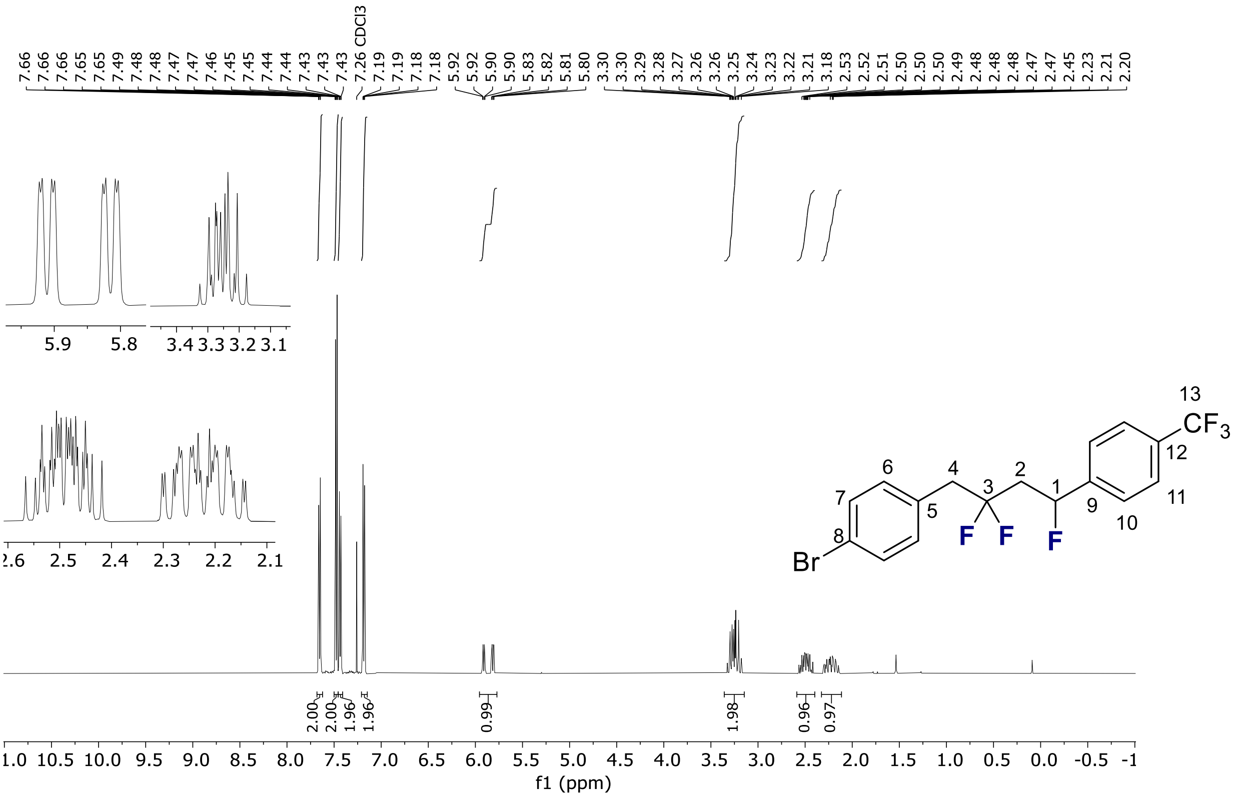


**Supplementary Figure 177.** $^{1}$H NMR of **2v** (500 MHz, 299 K, $CDCl_3$).

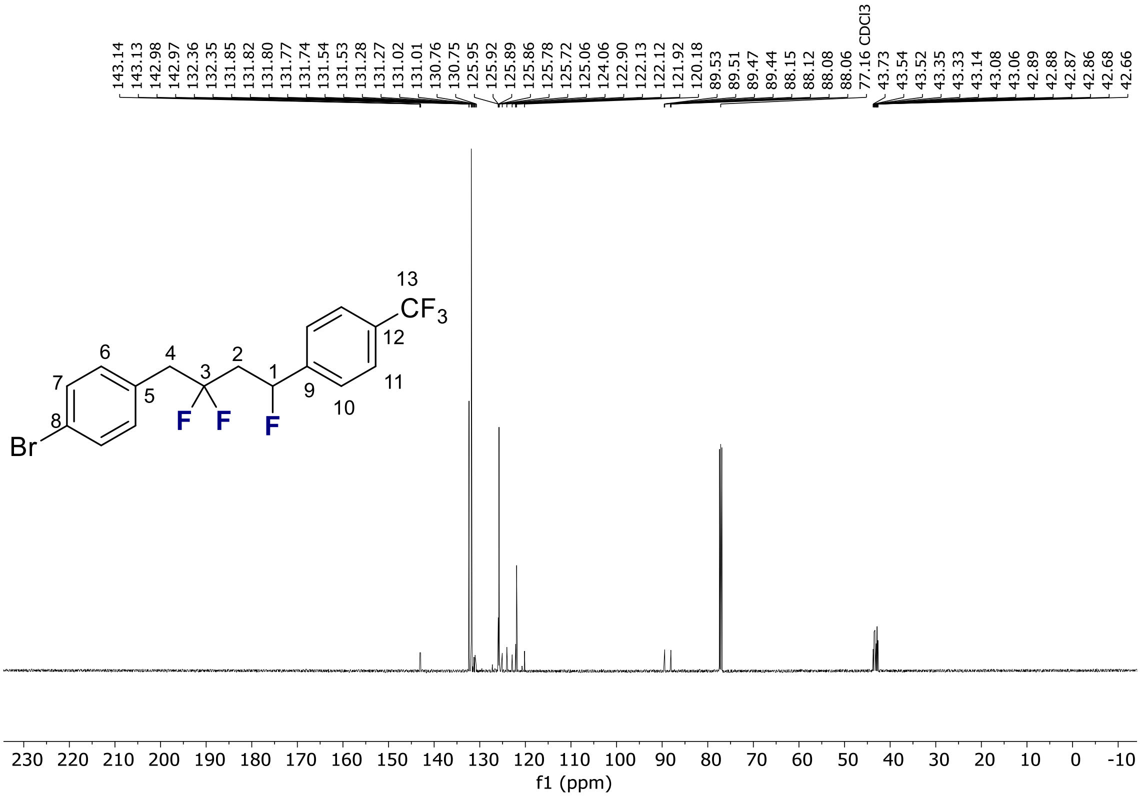


**Supplementary Figure 178.** $^{13}C\{^{1}H\}$ NMR of **2v** (126 MHz, 299 K, $CDCl_3$).

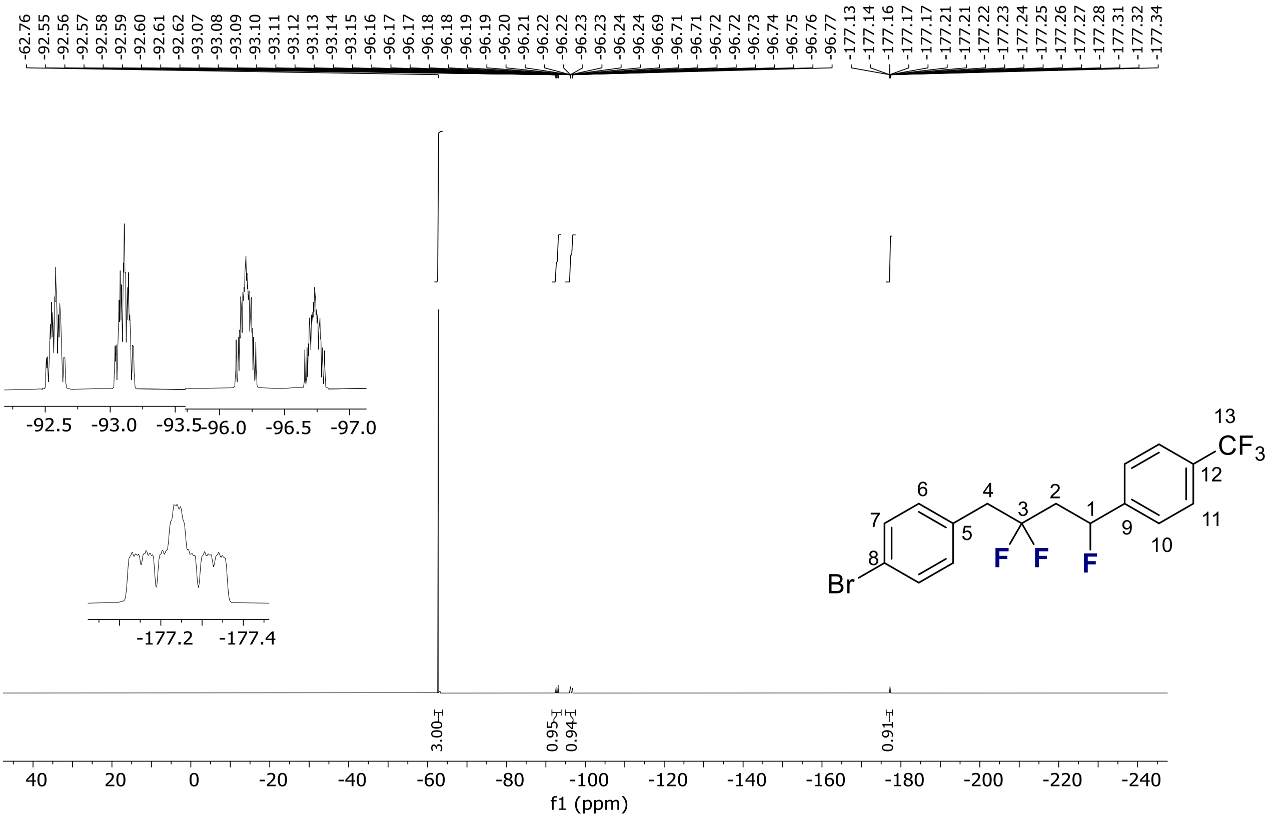


**Supplementary Figure 179.** $^{19}F$ NMR of **2v** (470 MHz, 299 K, $CDCl_3$).

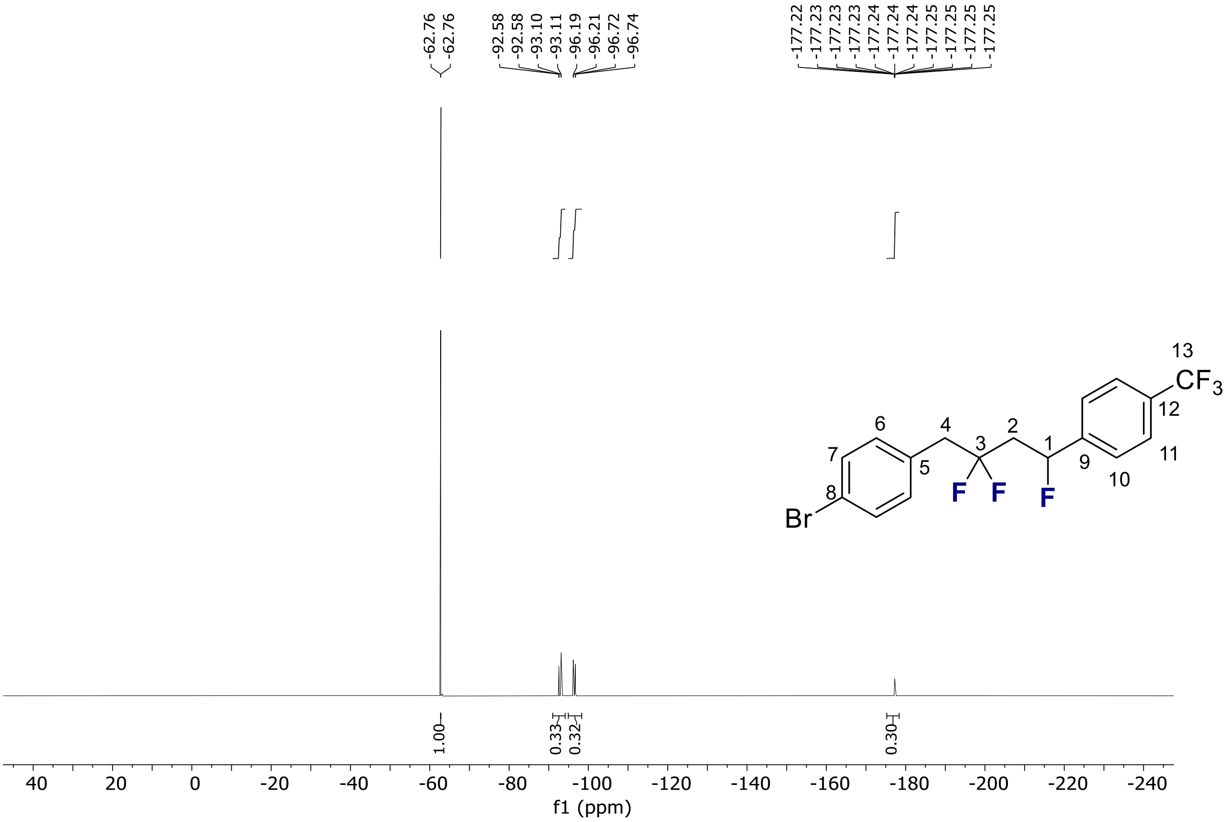


**Supplementary Figure 180.** $^{19}F\{^1H\}$ NMR of **2v** (470 MHz, 299 K, $CDCl_3$).

## 4-(4-(4-Bromophenyl)-1,3,3-trifluorobutyl)phenyl trifluoromethanesulfonate (2w)

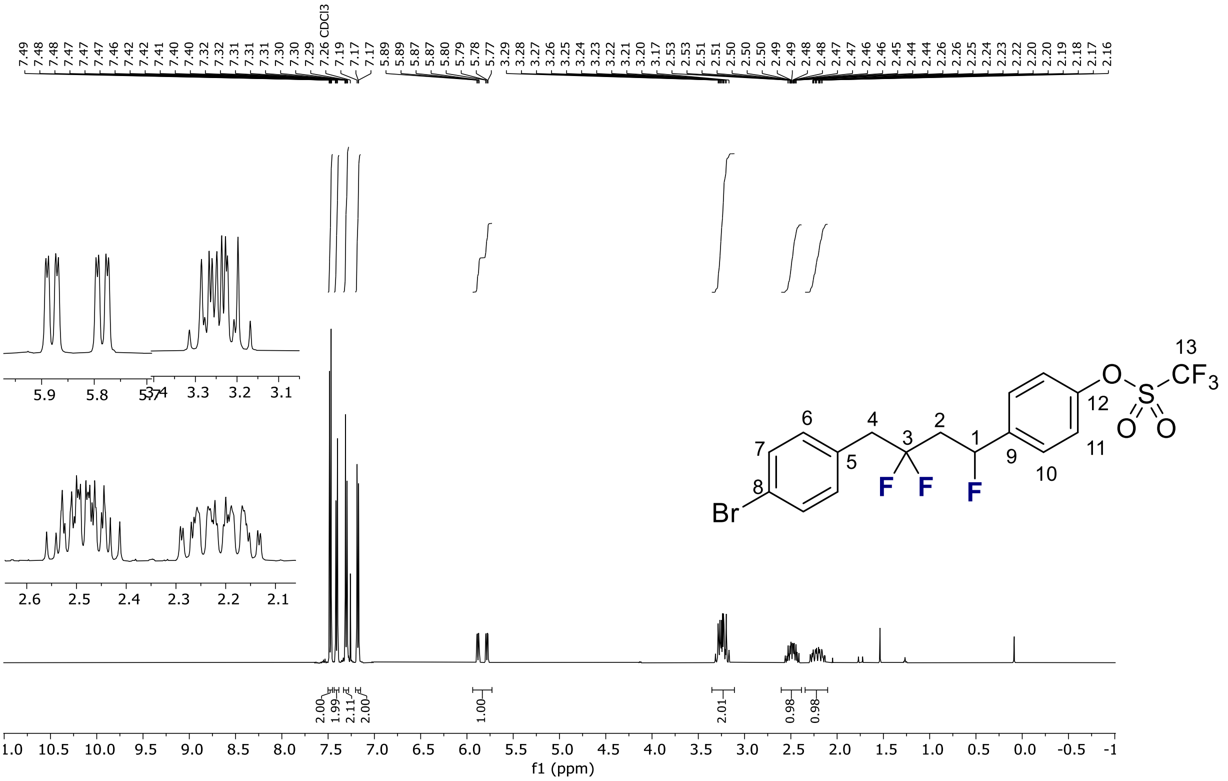


**Supplementary Figure 181.** $^{1}$H NMR of **2w** (500 MHz, 299 K, $CDCl_3$).

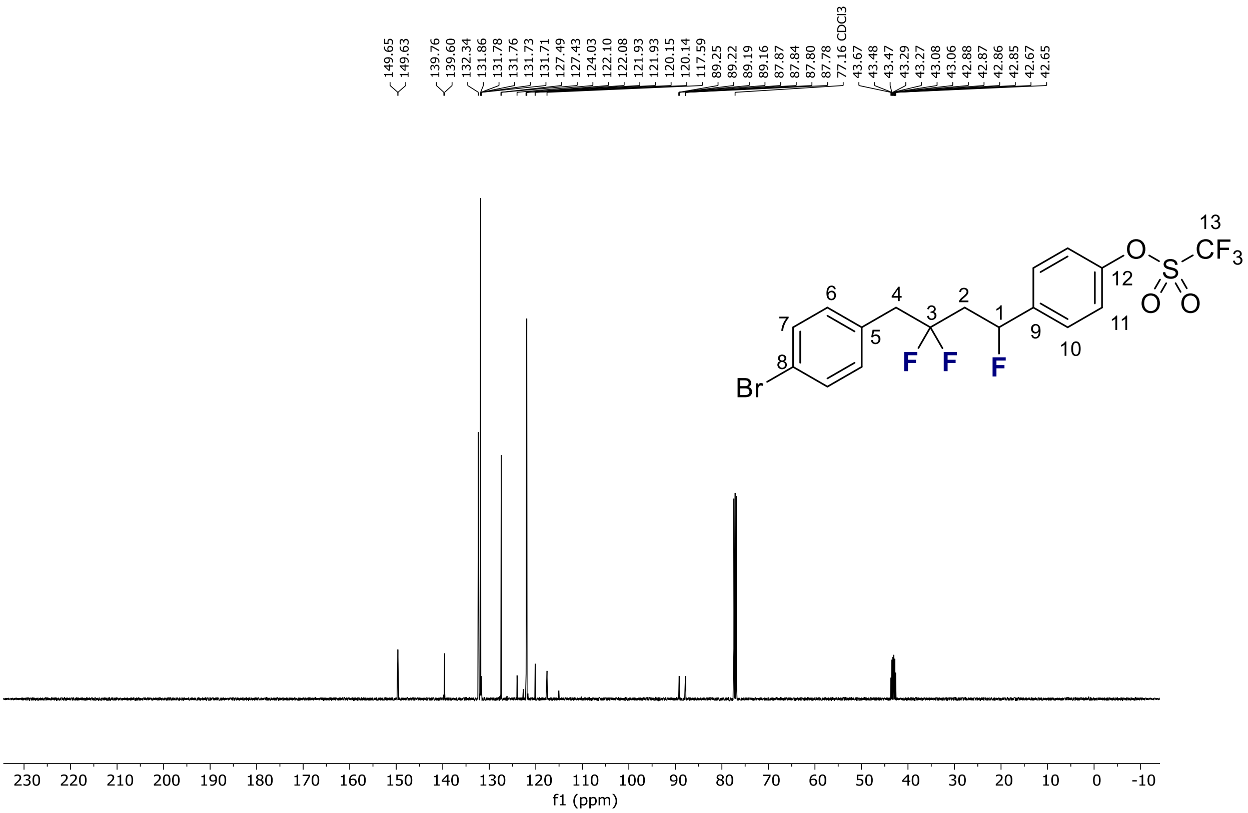


**Supplementary Figure 182.** $^{13}C\{^{1}H\}$ NMR of **2w** (126 MHz, 299 K, $CDCl_3$).

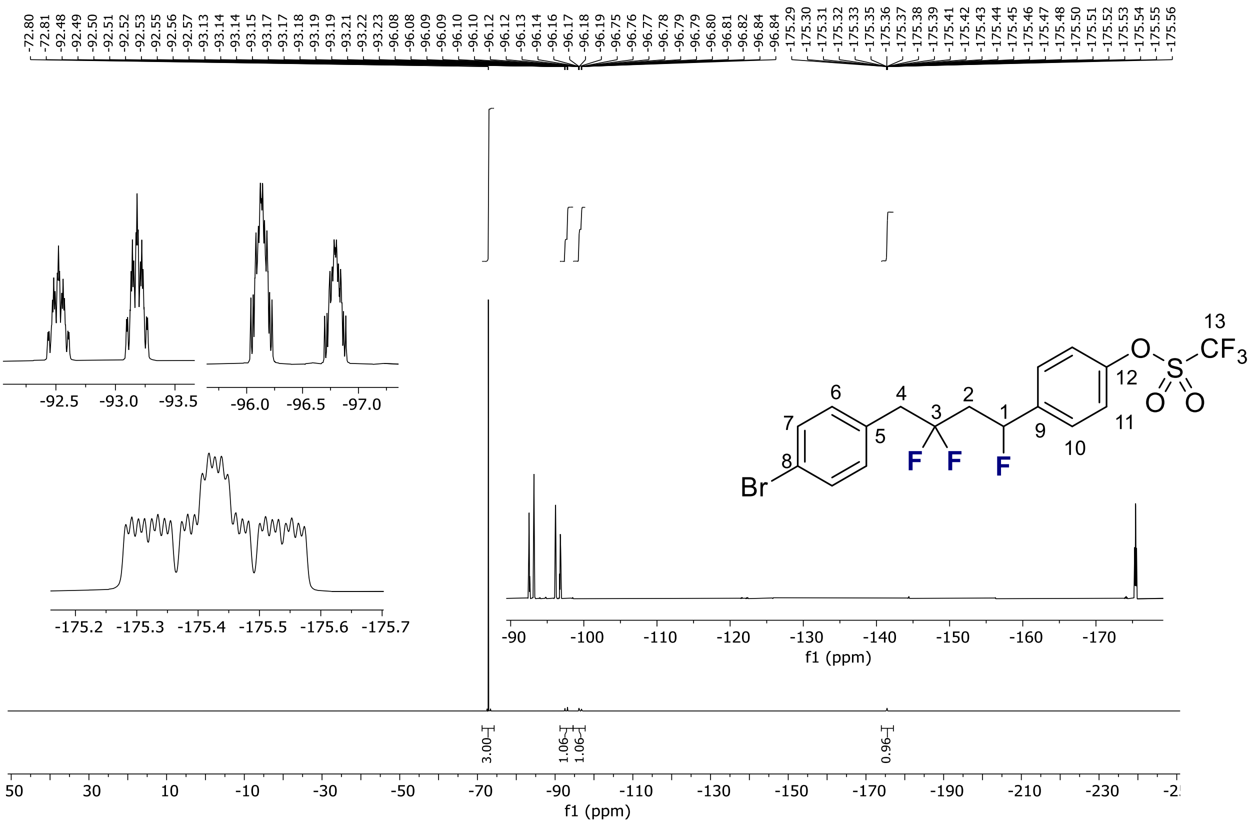


**Supplementary Figure 183.** $^{19}$F NMR of **2w** (376 MHz, 299 K, $CDCl_3$).

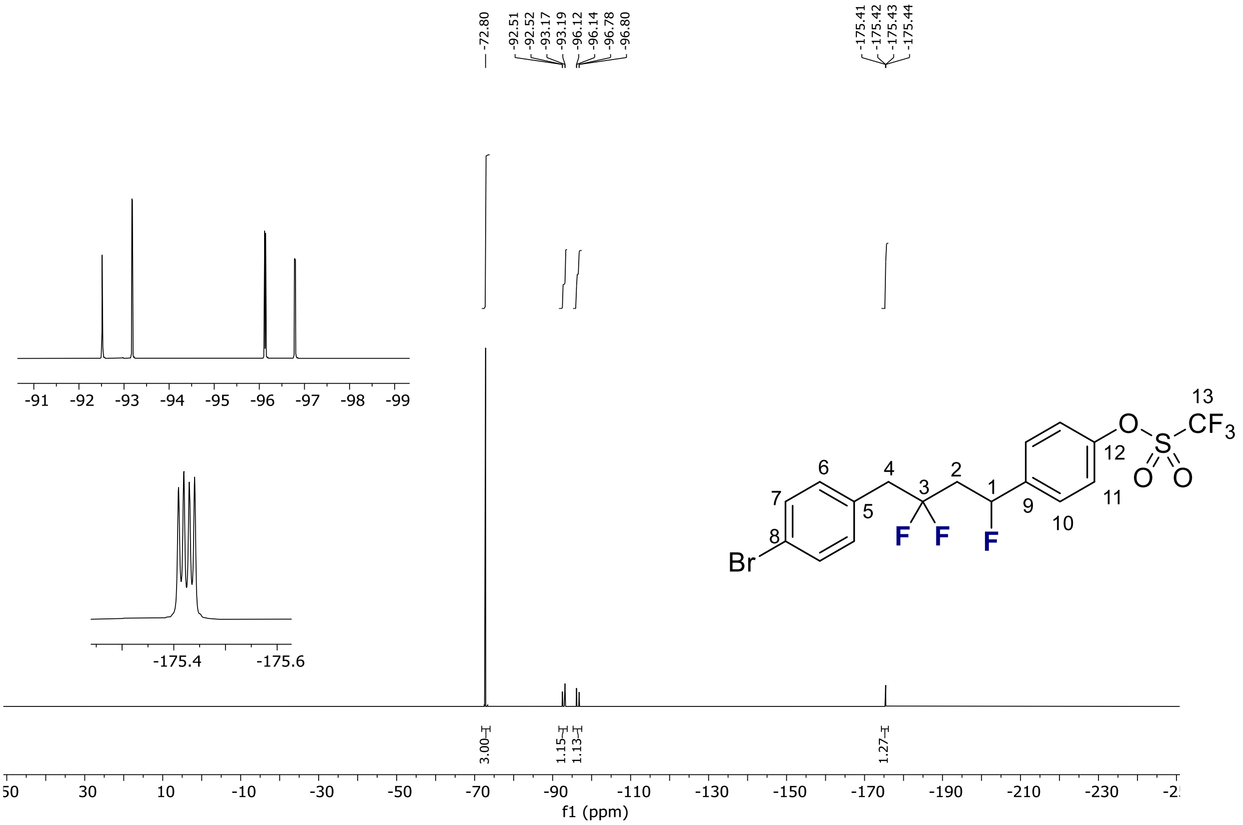


**Supplementary Figure 184.** $^{19}$F{$^{1}$H} NMR of **2w** (376 MHz, 299 K, $CDCl_3$).

## 1-Bromo-4-(4-(4-chlorophenyl)-2,2,4-trifluoropentyl)benzene (2x)

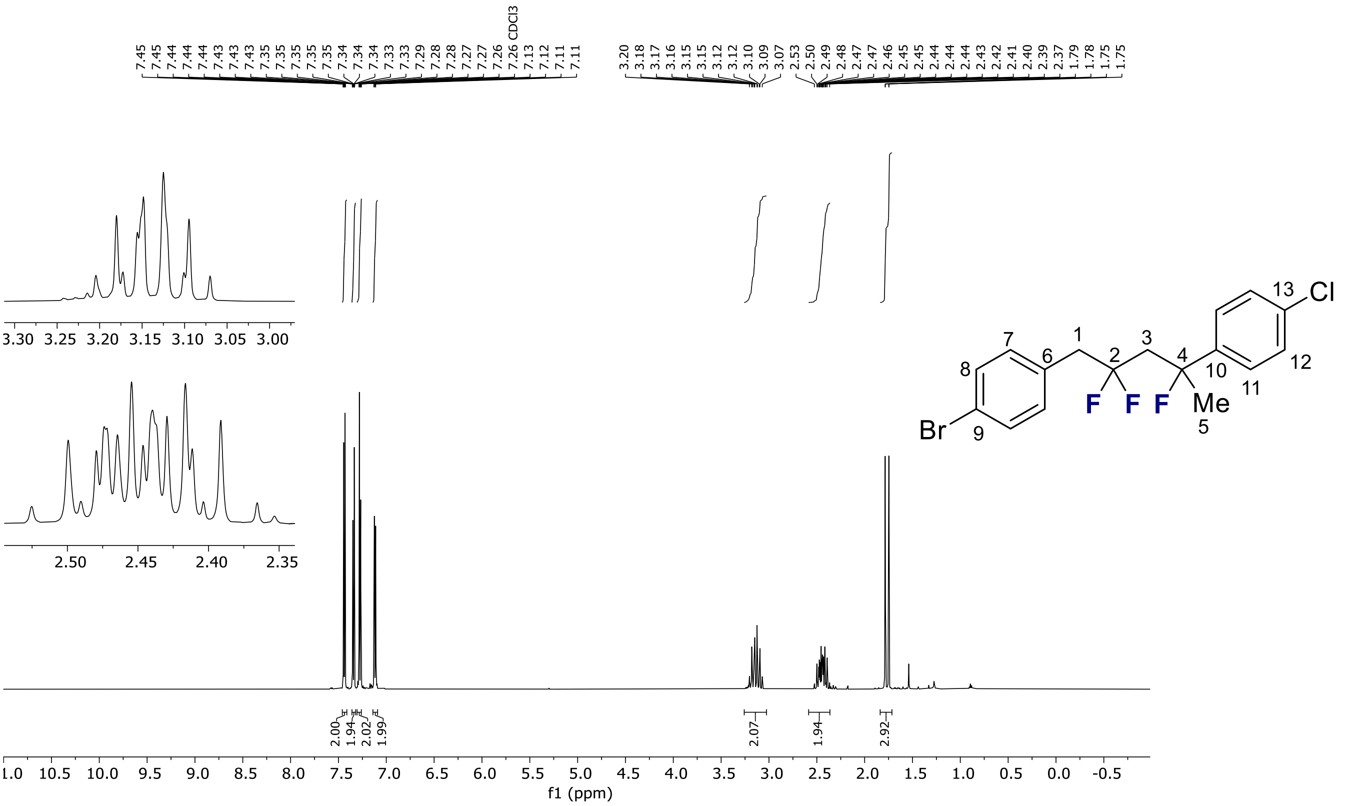


**Supplementary Figure 185.** $^{1}H$ NMR of **2x** (599 MHz, 299 K, $CDCl_3$).

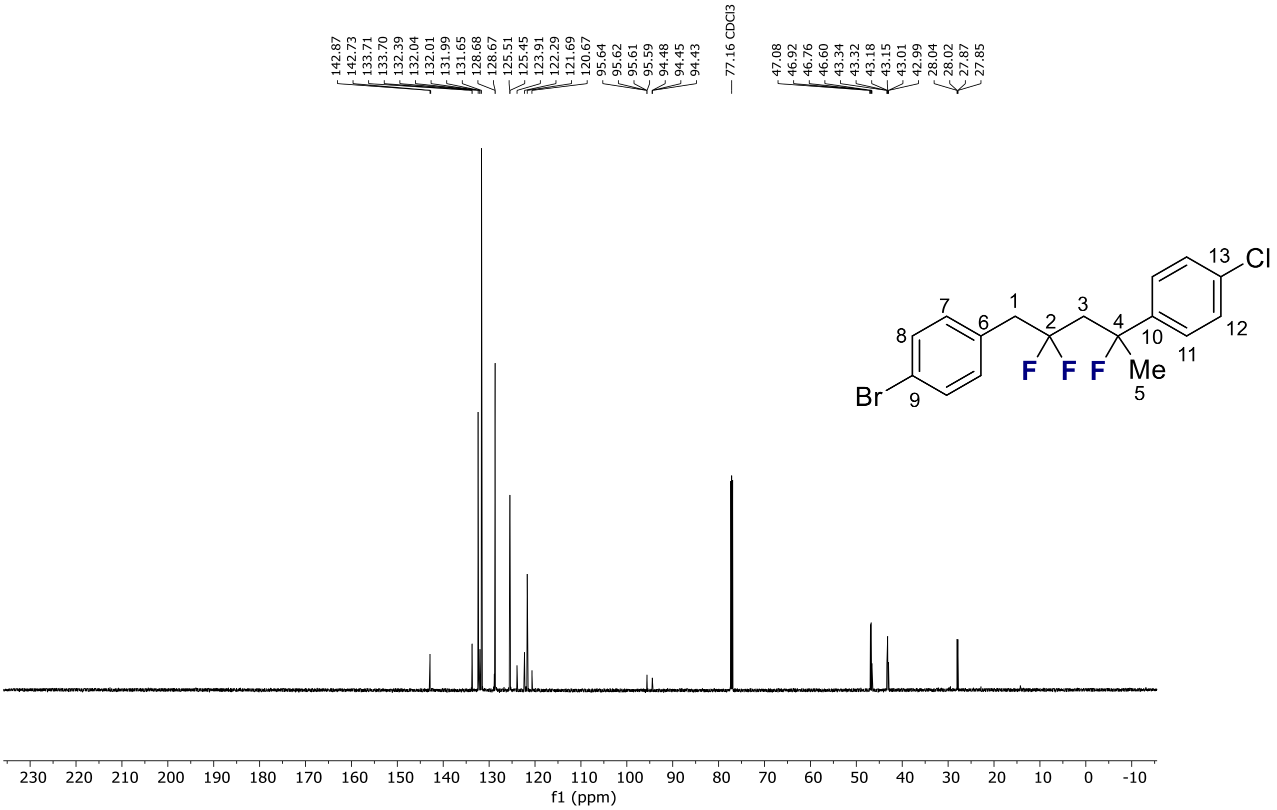


**Supplementary Figure 186.** $^{13}C\{^{1}H\}$ NMR of **2x** (151 MHz, 299 K, $CDCl_3$).

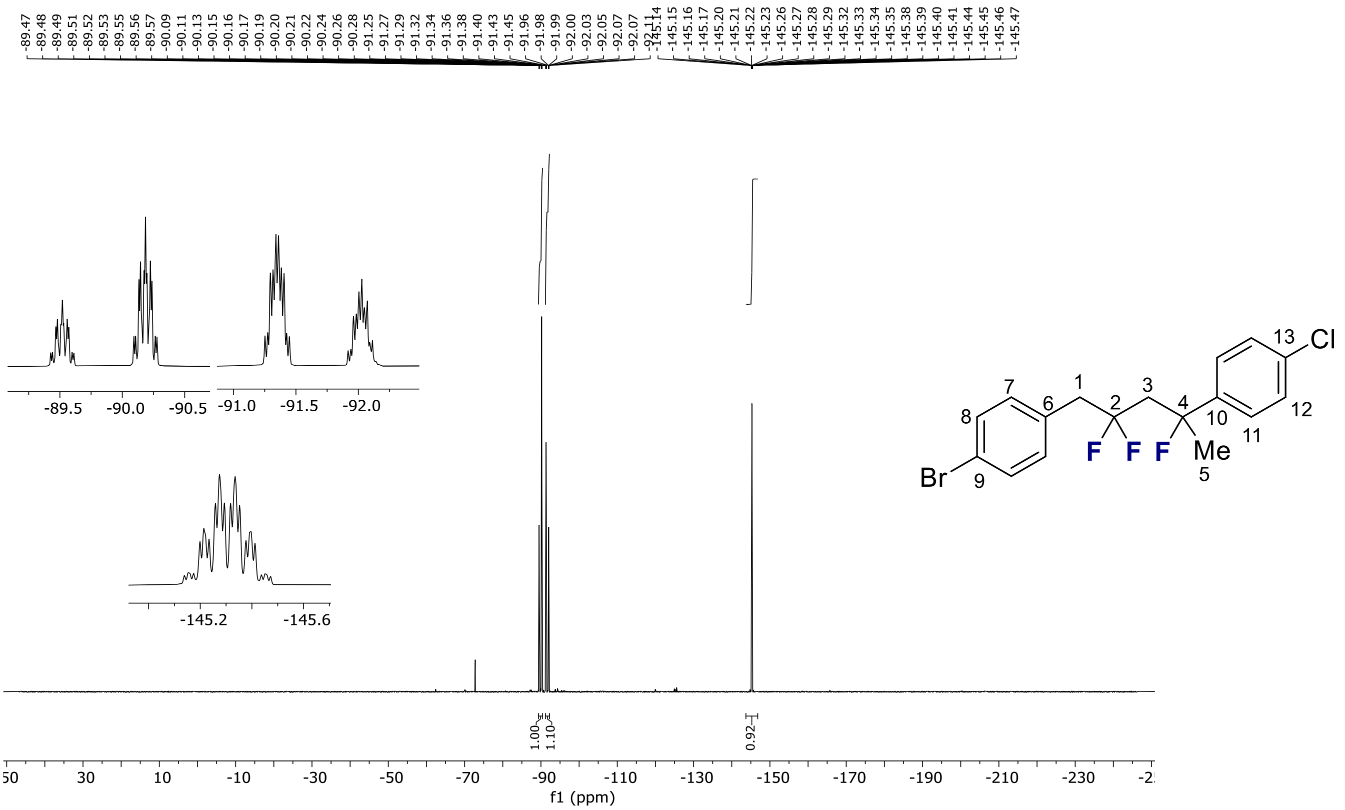


**Supplementary Figure 187.** $^{19}$F NMR of **2x** (376 MHz, 299 K, $CDCl_3$).

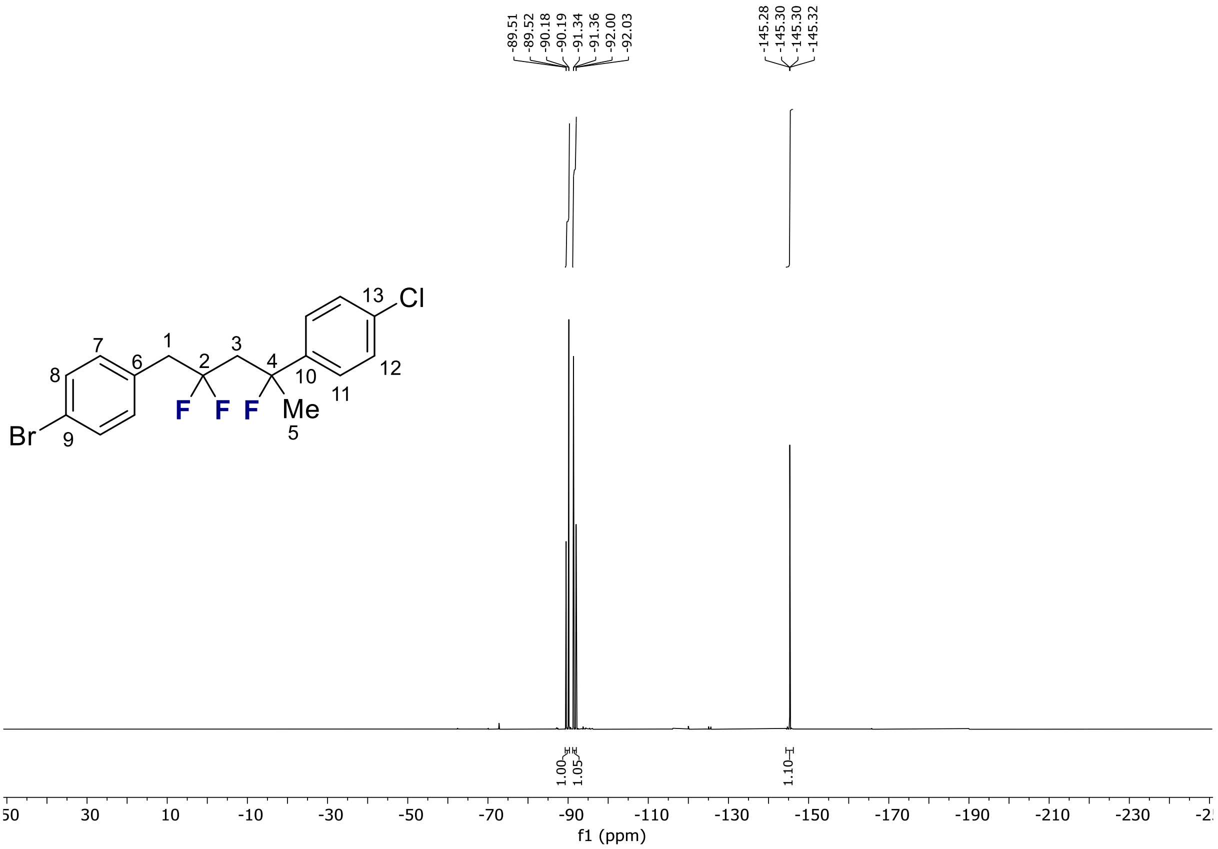


**Supplementary Figure 188.** $^{19}$F{$^{1}$H} NMR of **2x** (376 MHz, 299 K, $CDCl_3$).

## 3-(4-(1,3,3-Trifluoro-4-phenylbutyl)phenyl)pyridine (2y)

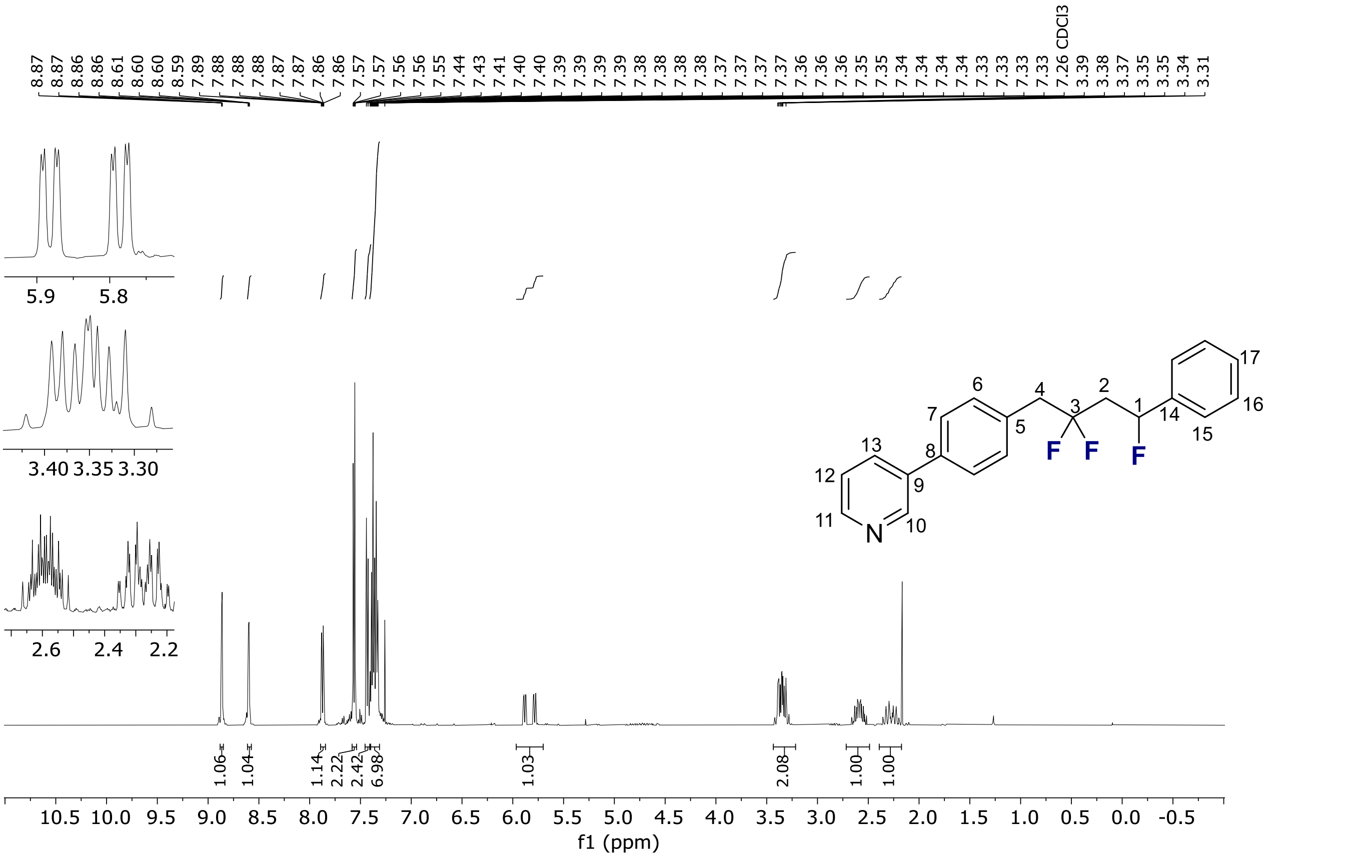


**Supplementary Figure 189.** $^{1}H$ NMR of **2y** (500 MHz, 299 K, $CDCl_3$).

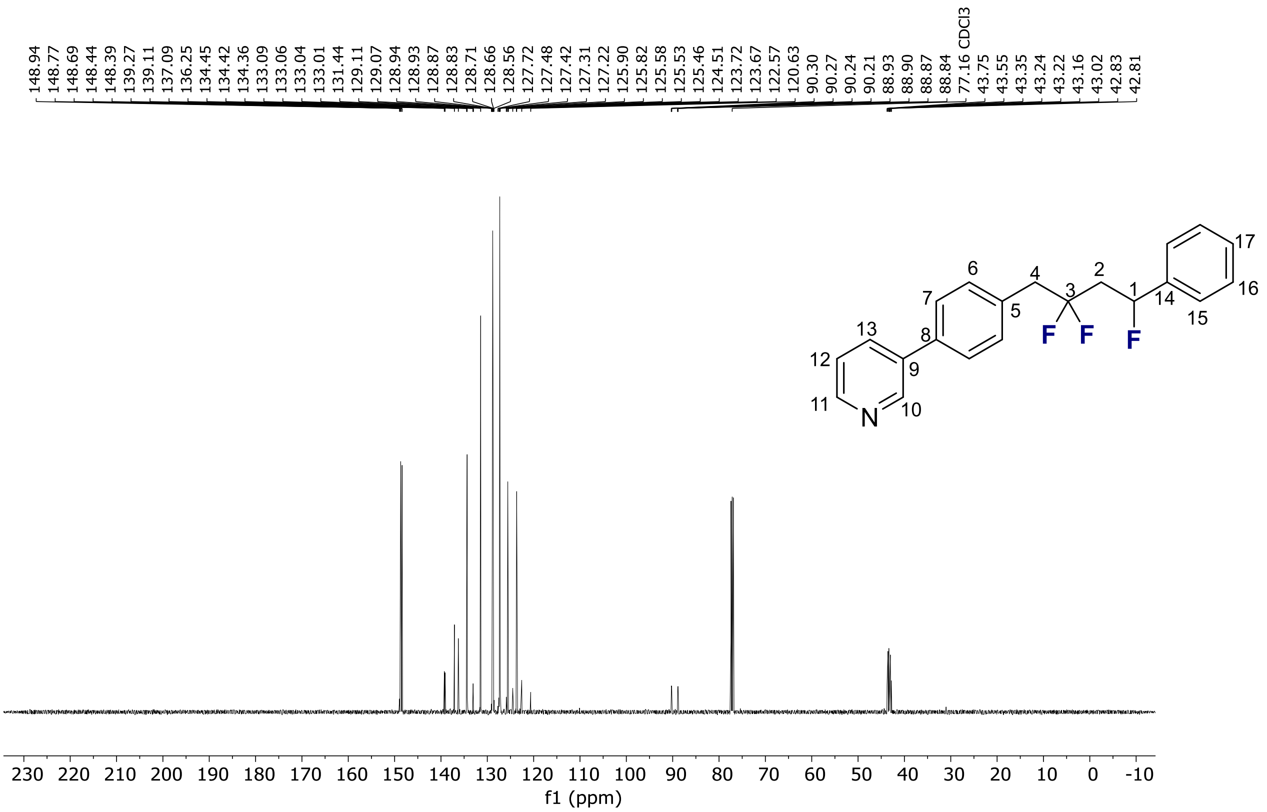


**Supplementary Figure 190.** $^{13}C\{^{1}H\}$ NMR of **2y** (126 MHz, 299 K, $CDCl_3$).

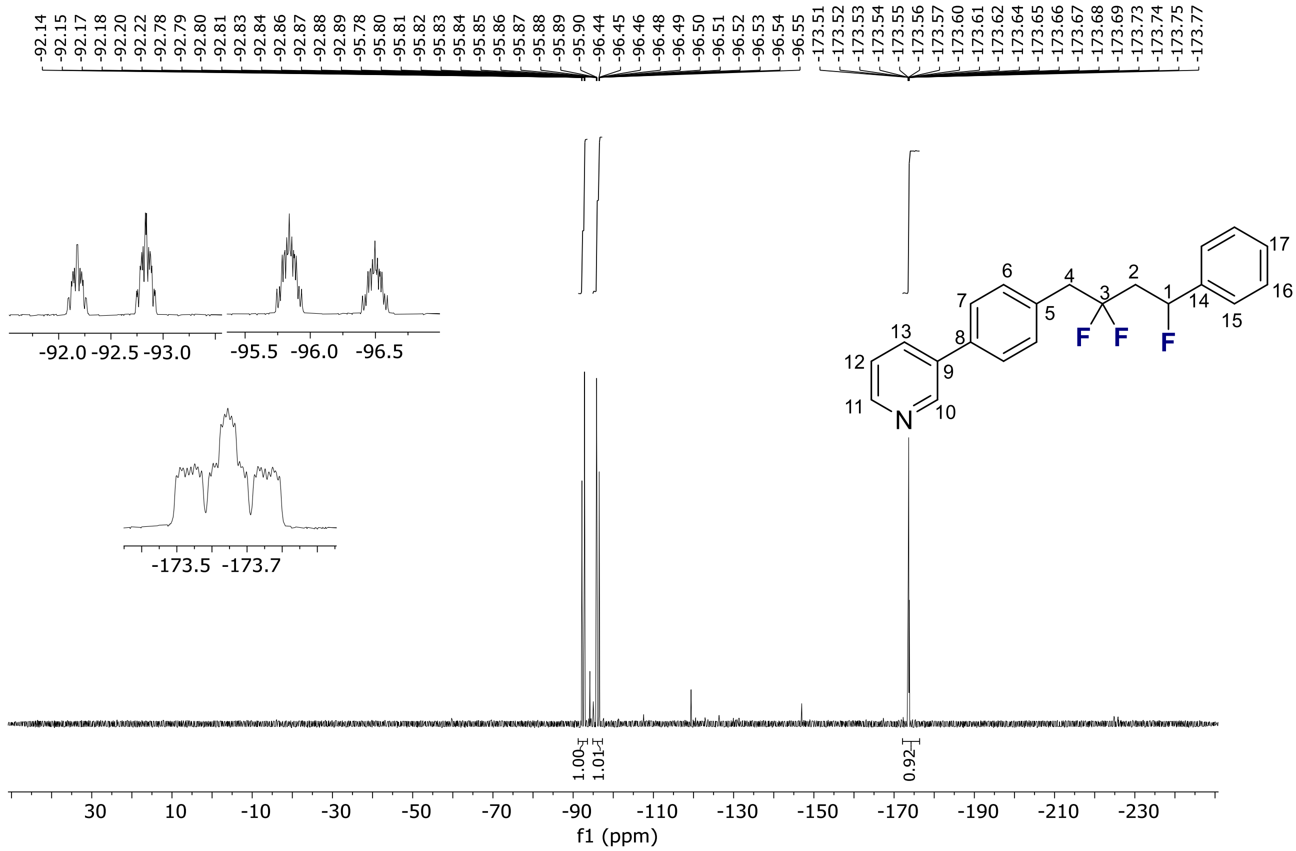

**Supplementary Figure 191.** $^{19}F$ NMR of **2y** (376 MHz, 299 K, $CDCl_3$).

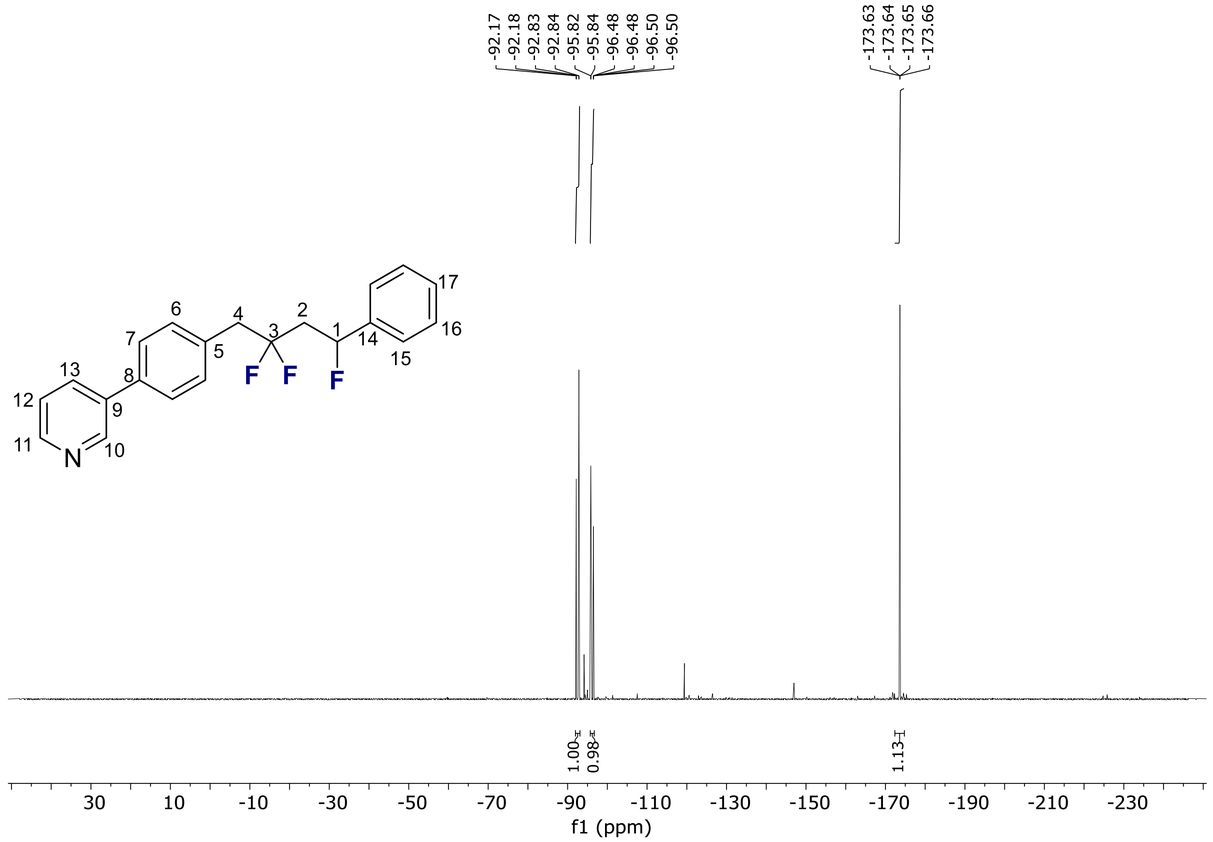

**Supplementary Figure 192.** $^{19}F\{^1H\}$ NMR of **2y** (376 MHz, 299 K, $CDCl_3$).

## 3,3-Difluoro-1-phenyl-1,2,3,4-tetrahydronaphthalene (3a)

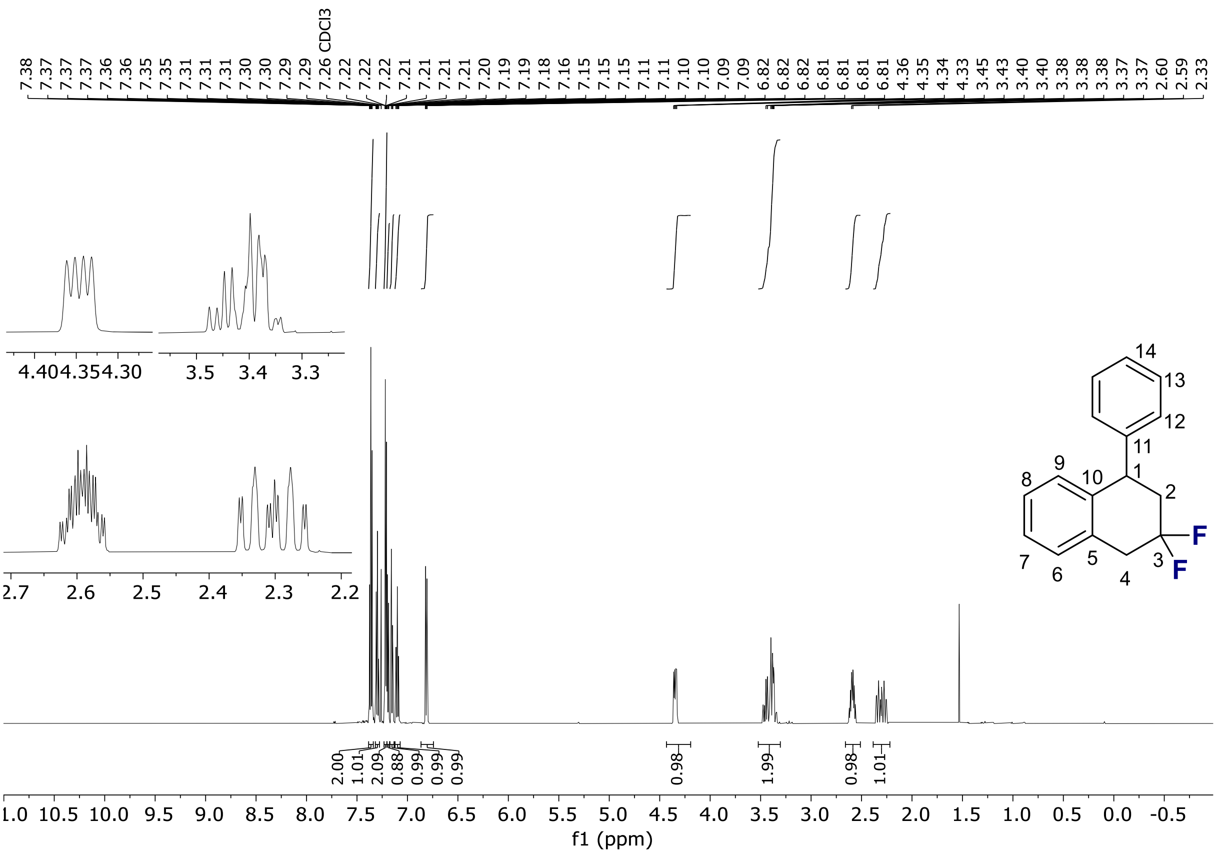

**Supplementary Figure 193.** $^{1}$H NMR of **3a** (599 MHz, 299 K, $CDCl_3$).

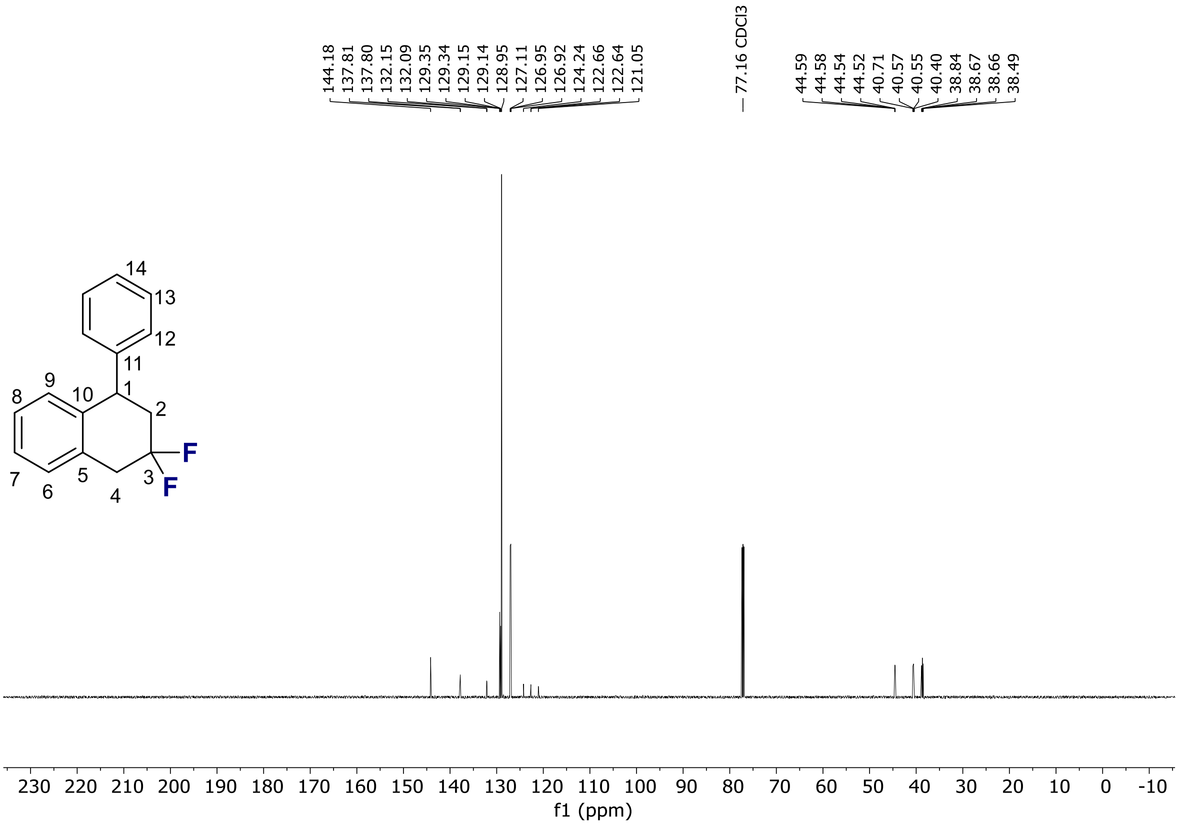

**Supplementary Figure 194.** $^{13}C\{^1H\}$ NMR of **3a** (151 MHz, 299 K, $CDCl_3$).

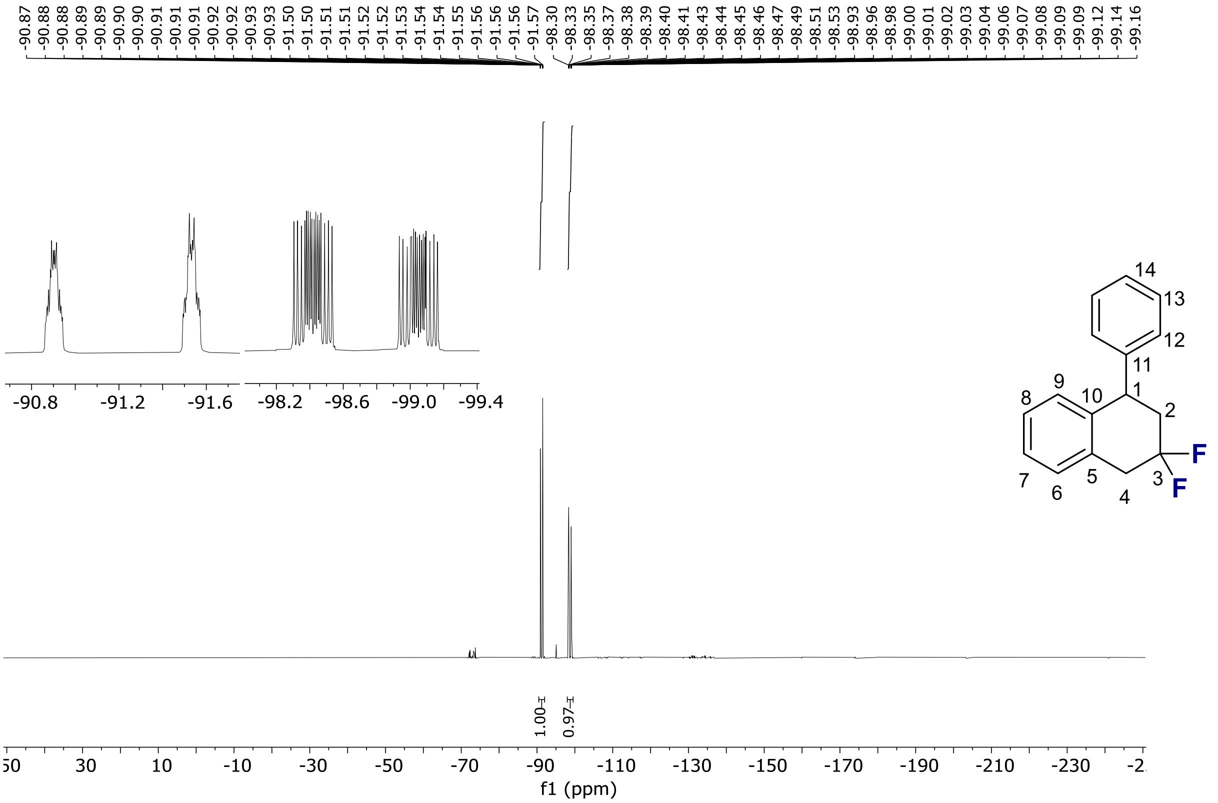


**Supplementary Figure 195.** $^{19}$F NMR of **3a** (376 MHz, 299 K, $CDCl_3$).

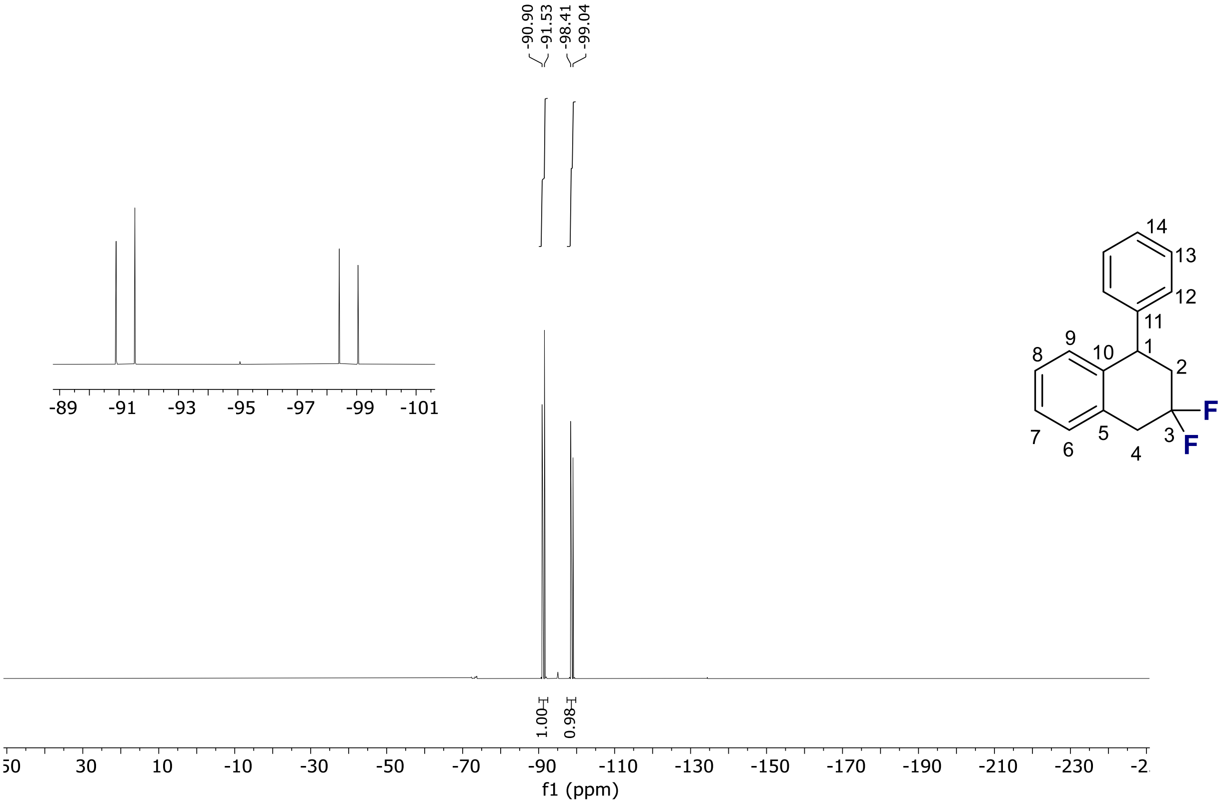


**Supplementary Figure 196.** $^{19}$F{$^{1}$H} NMR of **3a** (376 MHz, 299 K, $CDCl_3$).

**3,3,7-Trifluoro-1-(4-fluorophenyl)-1,2,3,4-tetrahydronaphthalene (3b)**

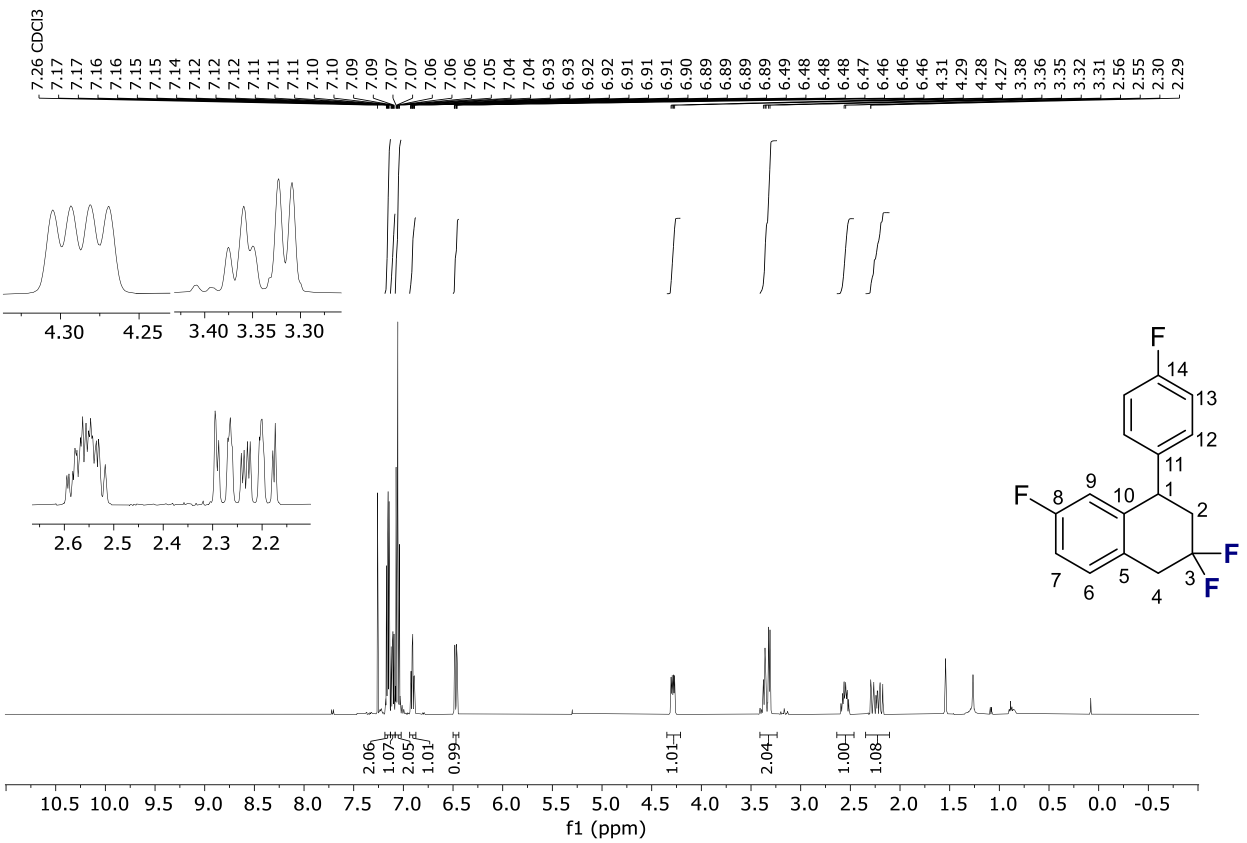

**Supplementary Figure 197.** $^{1}H$ NMR of **3b** (500 MHz, 299 K, $CDCl_3$).

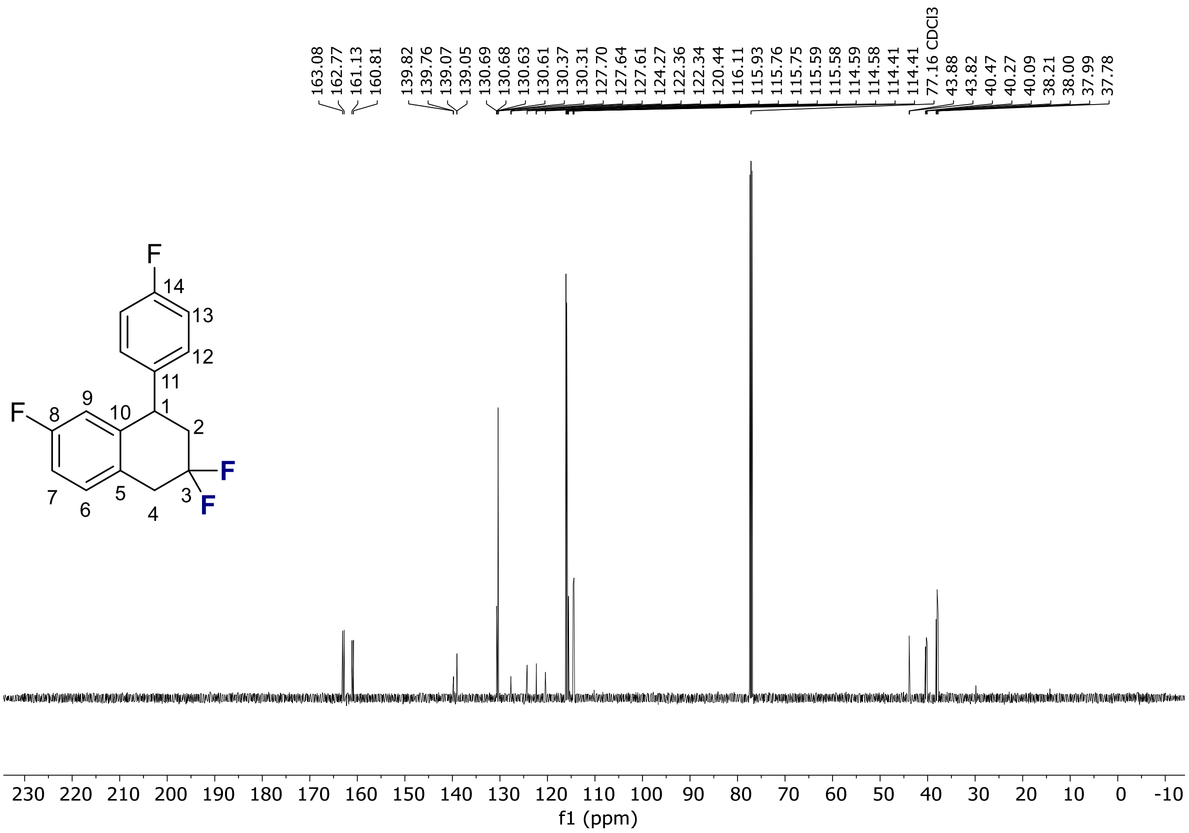

**Supplementary Figure 198.** $^{13}C\{^{1}H\}$ NMR of **3b** (126 MHz, 299 K, $CDCl_3$).

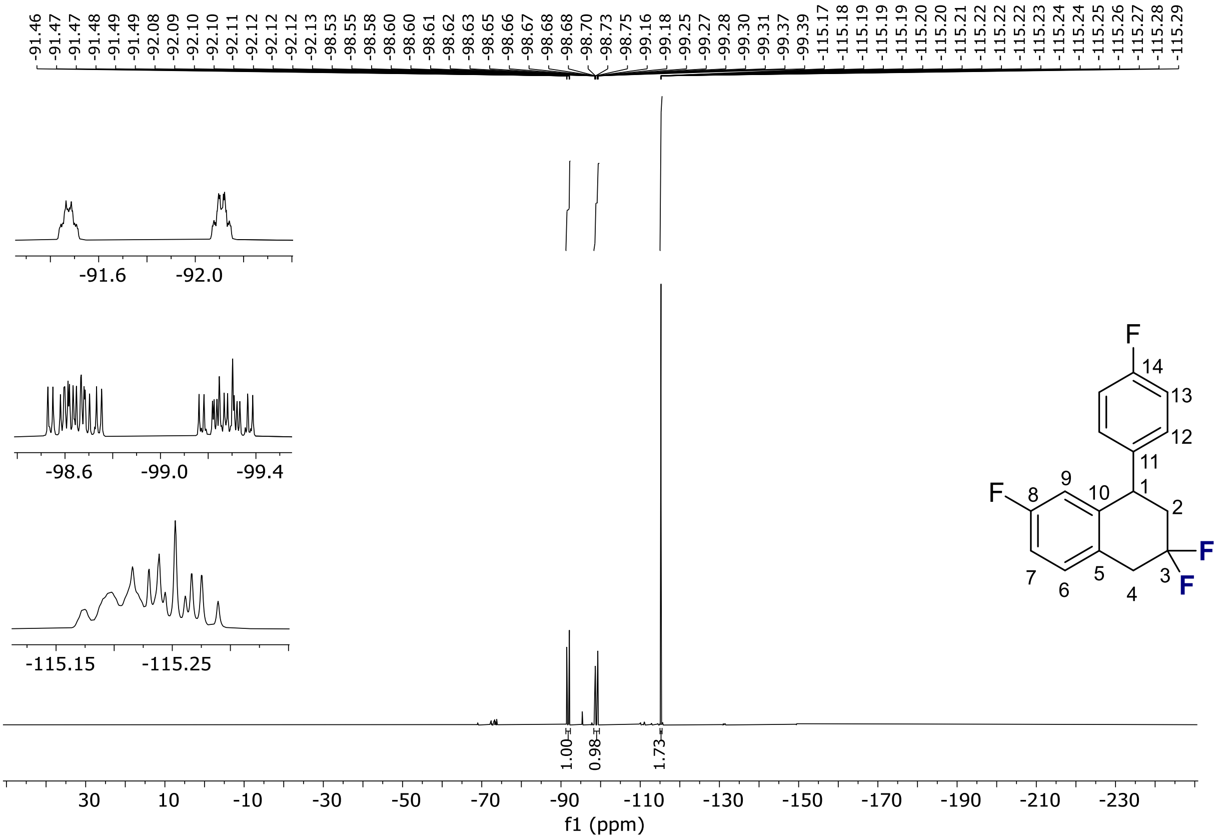

**Supplementary Figure 199.** $^{19}F$ NMR of **3b** (376 MHz, 299 K, $CDCl_3$).

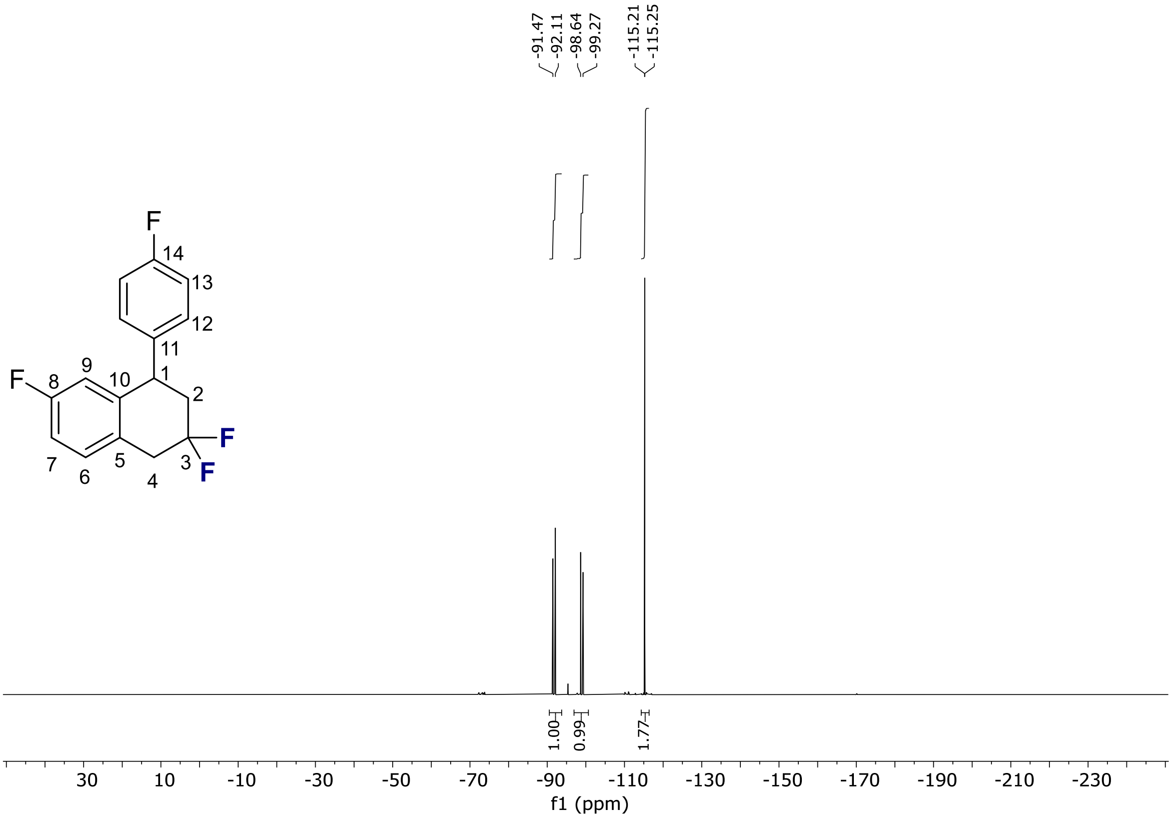

**Supplementary Figure 200.** $^{19}F\{^1H\}$ NMR of **3b** (376 MHz, 299 K, $CDCl_3$).

**7-Chloro-1-(4-chlorophenyl)-3,3-difluoro-1,2,3,4-tetrahydronaphthalene (3c)**

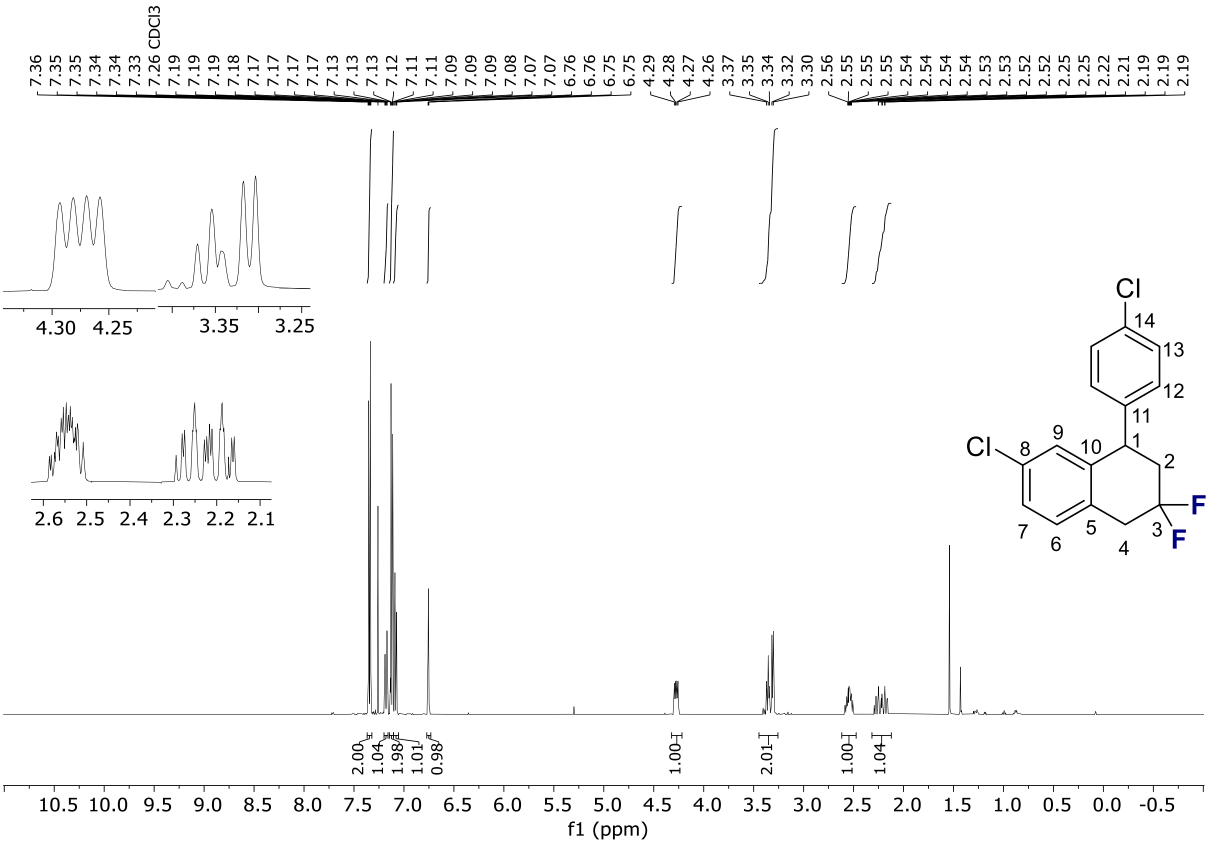

**Supplementary Figure 201.** $^{1}H$ NMR of **3c** (500 MHz, 299 K, $CDCl_3$).

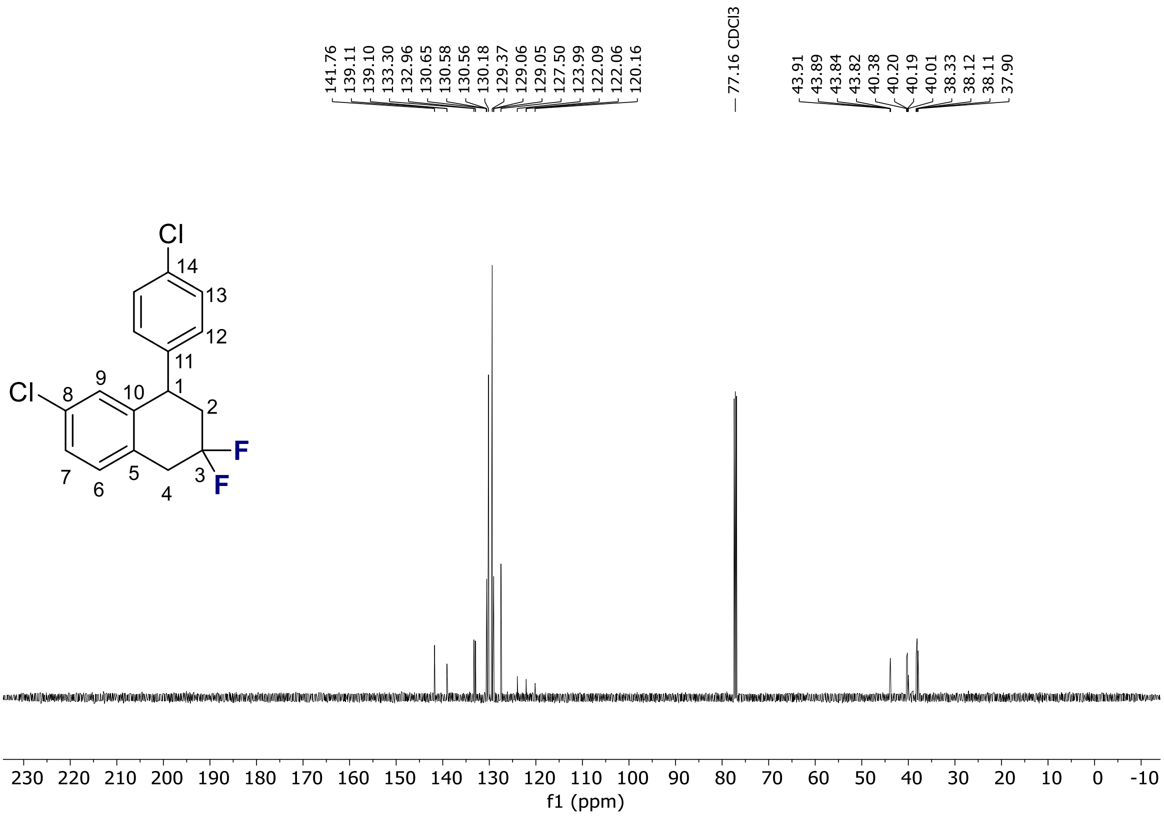

**Supplementary Figure 202.** $^{13}C\{^{1}H\}$ NMR of **3c** (126 MHz, 299 K, $CDCl_3$).

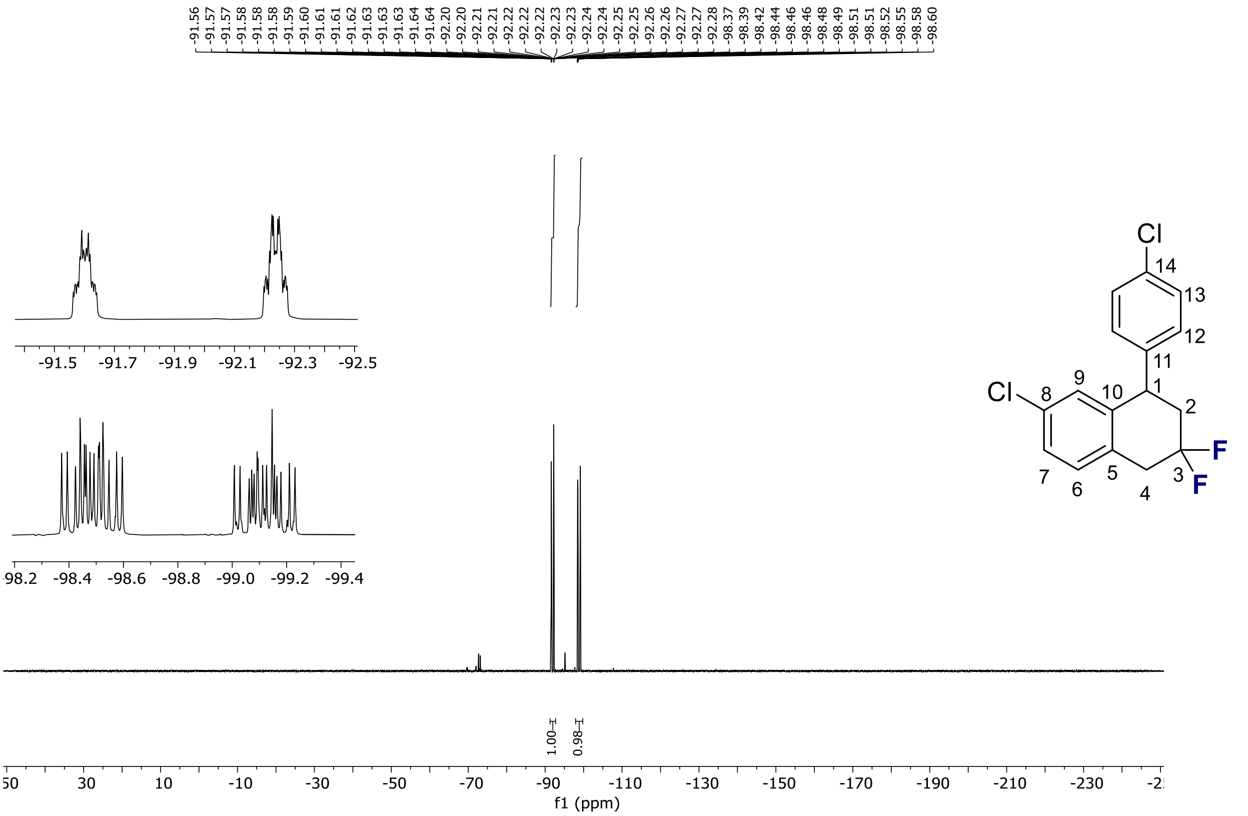


**Supplementary Figure 203.** $^{19}$F NMR of **3c** (376 MHz, 299 K, $CDCl_3$).

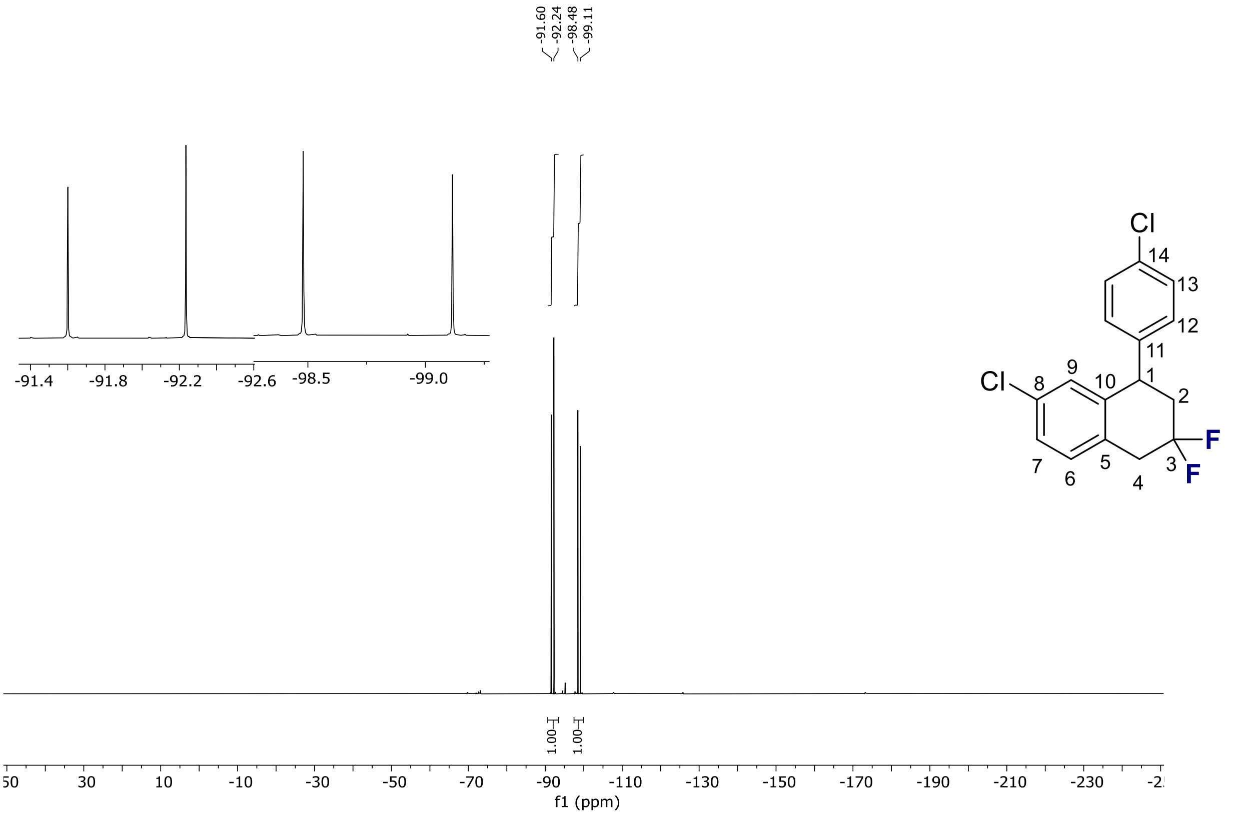


**Supplementary Figure 204.** $^{19}$F{$^{1}$H} NMR of **3c** (376 MHz, 299 K, $CDCl_3$).

## 7-Bromo-1-(4-bromophenyl)-3,3-difluoro-1,2,3,4-tetrahydronaphthalene (3d)

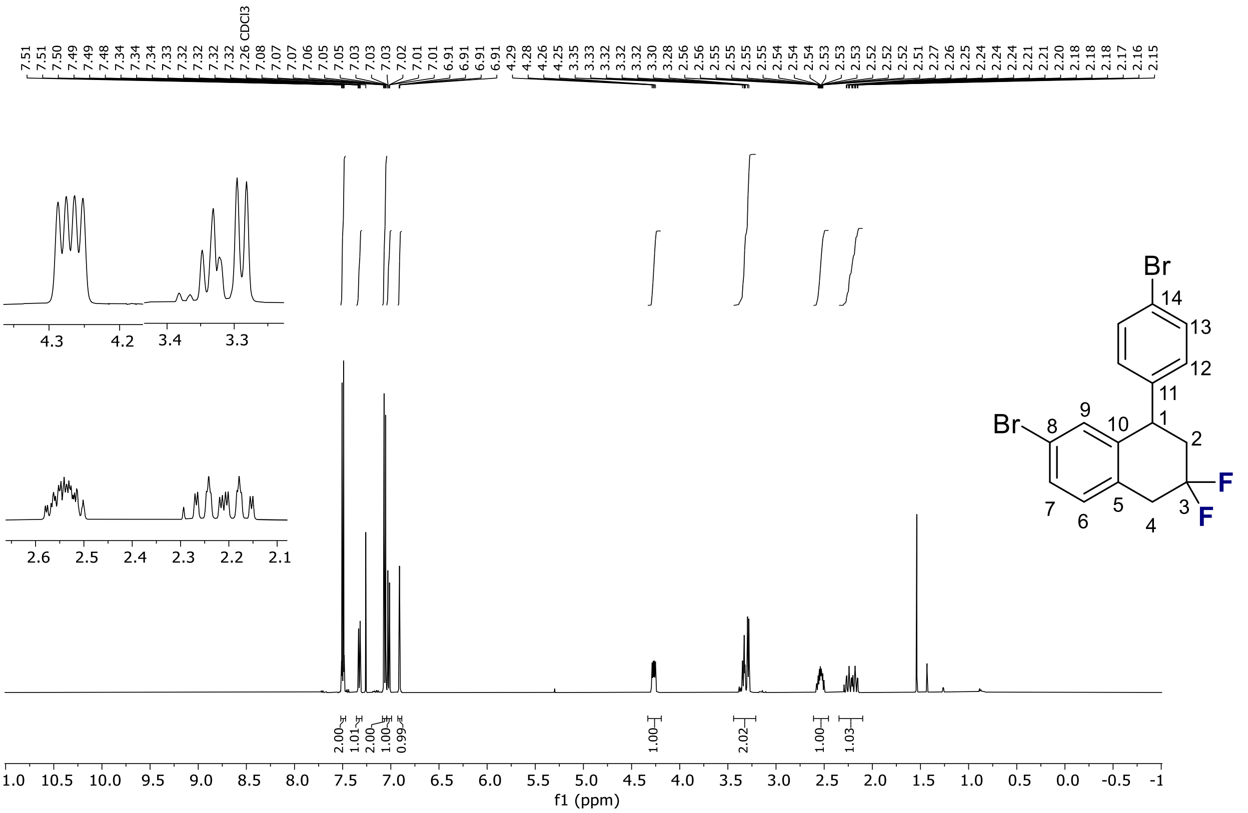


**Supplementary Figure 205.** $^{1}$H NMR of **3d** (500 MHz, 299 K, $CDCl_3$).

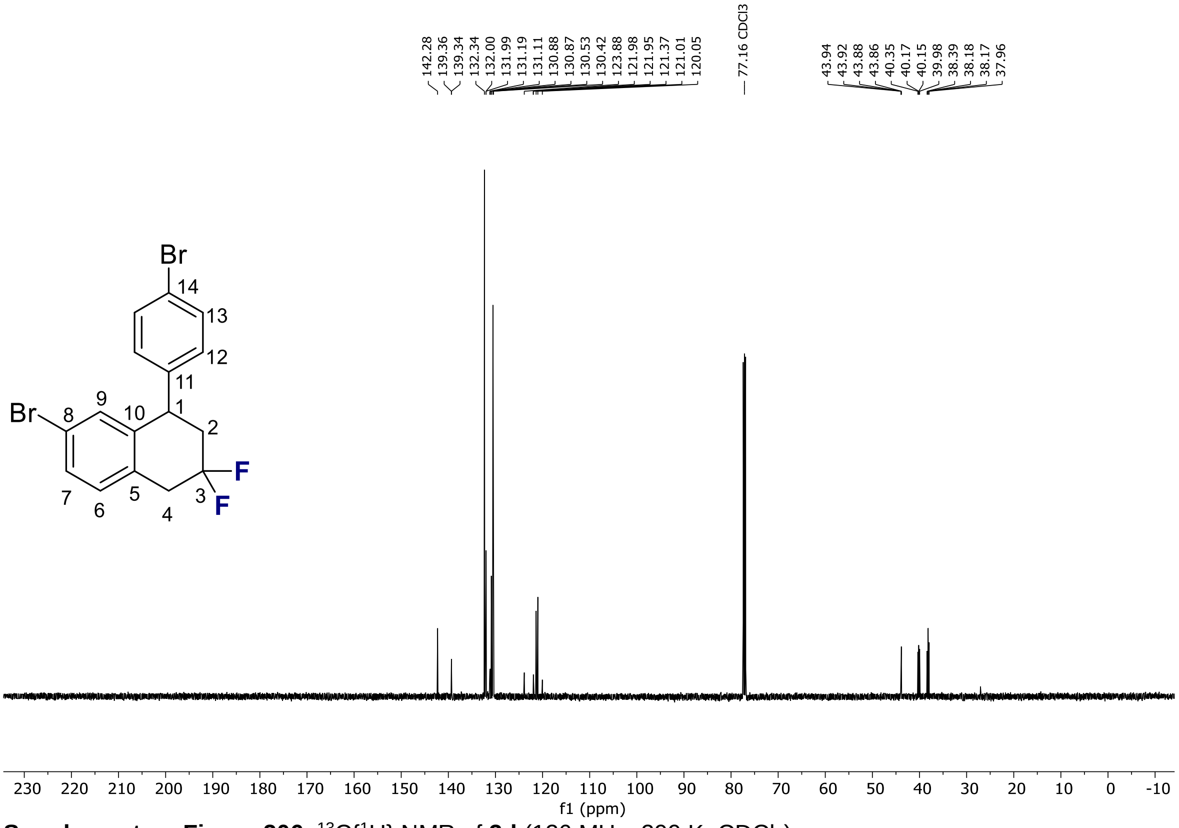


**Supplementary Figure 206.** $^{13}$C{$^{1}$H} NMR of **3d** (126 MHz, 299 K, $CDCl_3$).

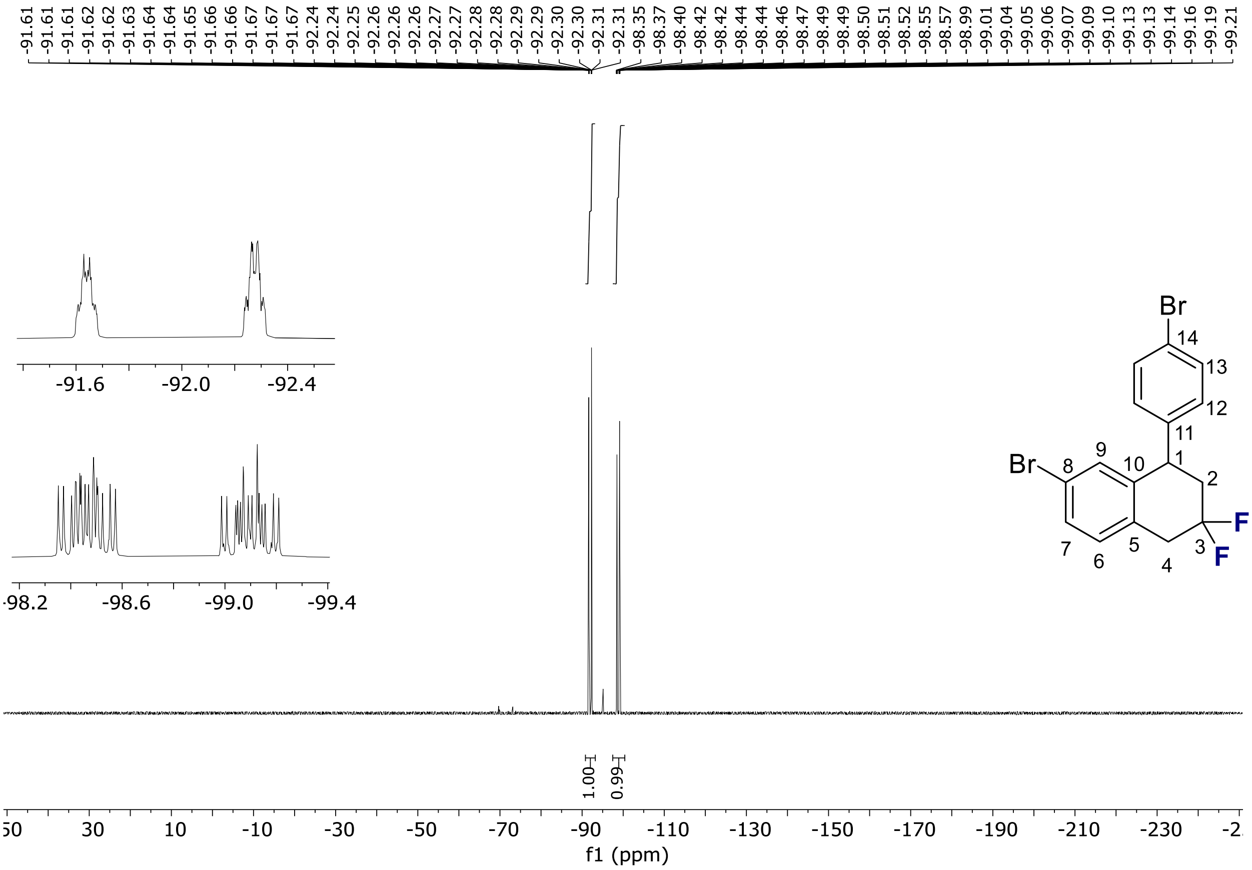


**Supplementary Figure 207.** $^{19}$F NMR of **3d** (376 MHz, 299 K, $CDCl_3$).

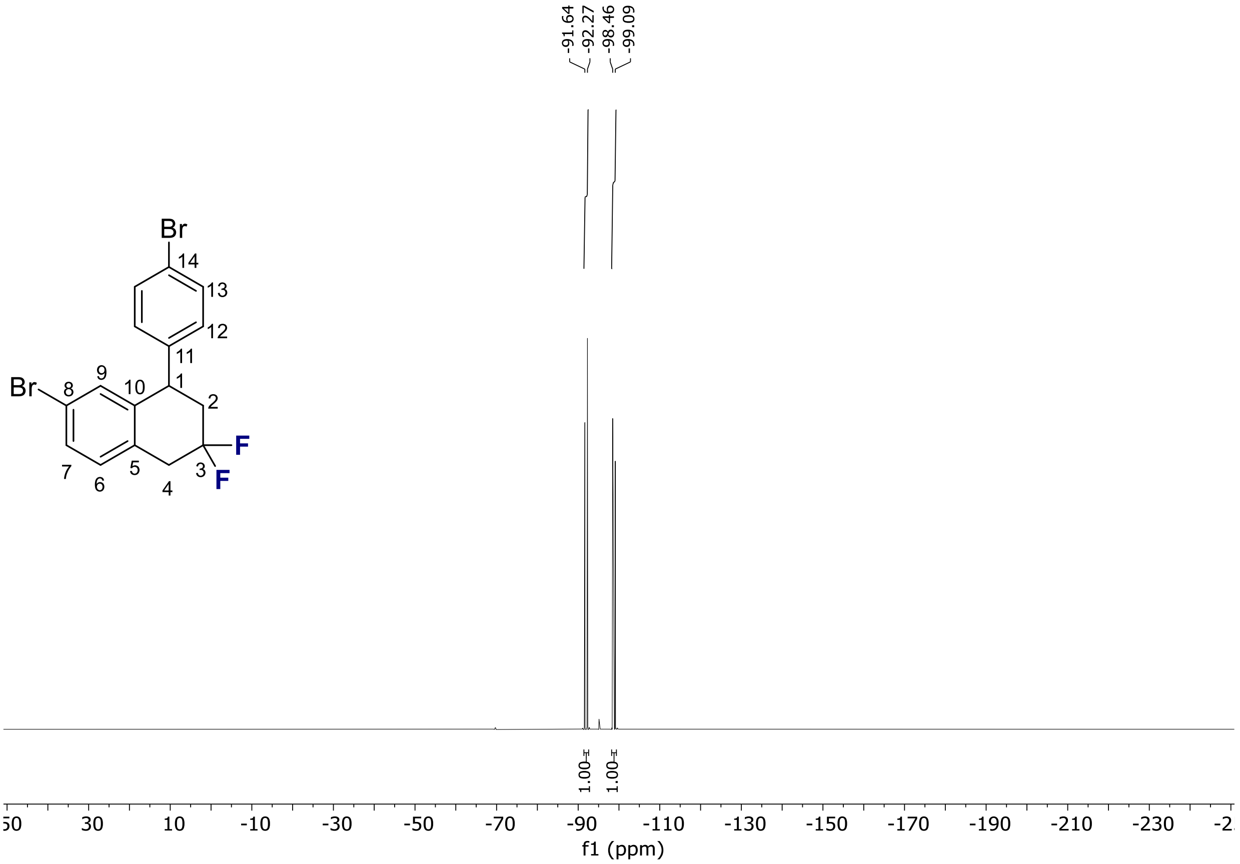


**Supplementary Figure 208.** $^{19}$F{$^1$H} NMR of **3d** (376 MHz, 299 K, $CDCl_3$).

## 3,3-Difluoro-7-(trifluoromethyl)-1-(4-(trifluoromethyl)phenyl)-1,2,3,4-tetrahydronaphthalene (3e)

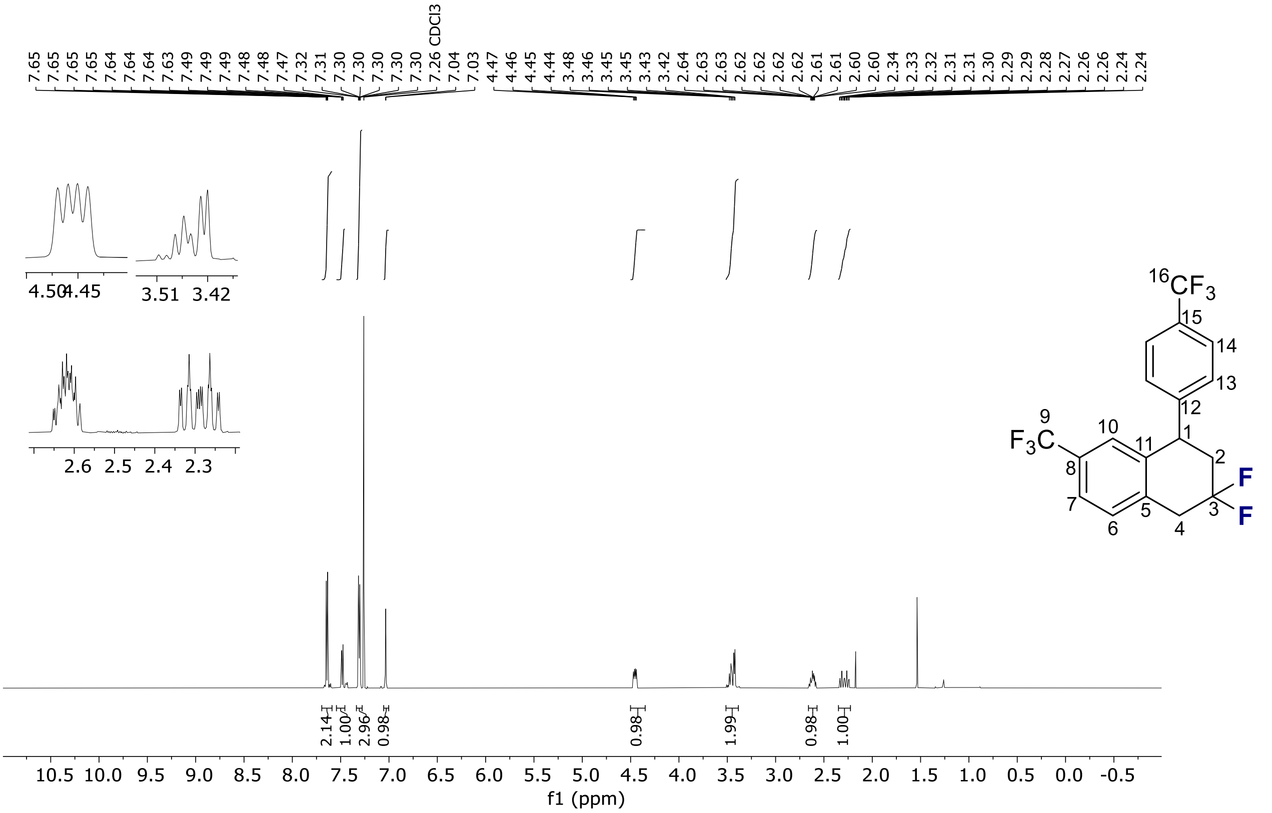


**Supplementary Figure 209.** $^{1}$H NMR of **3e** (500 MHz, 299 K, $CDCl_3$).

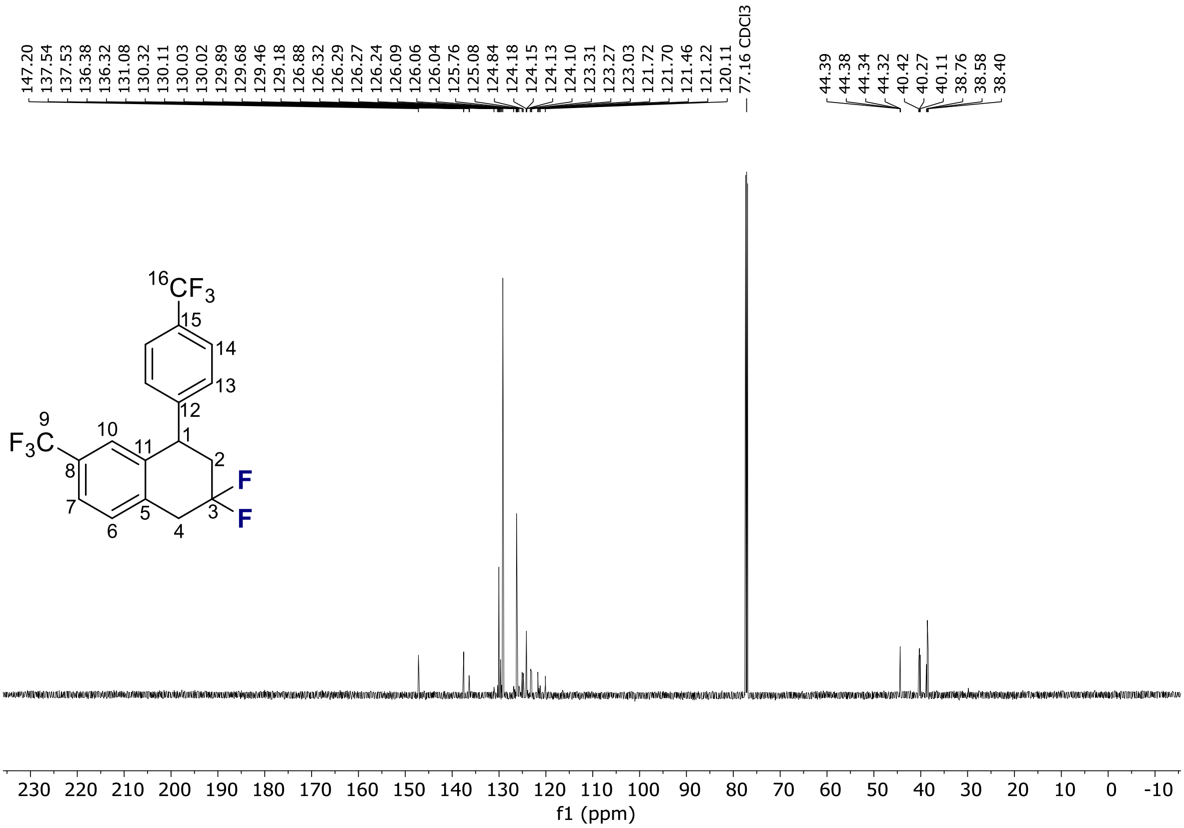


**Supplementary Figure 210.** $^{13}C\{^{1}H\}$ NMR of **3e** (126 MHz, 299 K, $CDCl_3$).

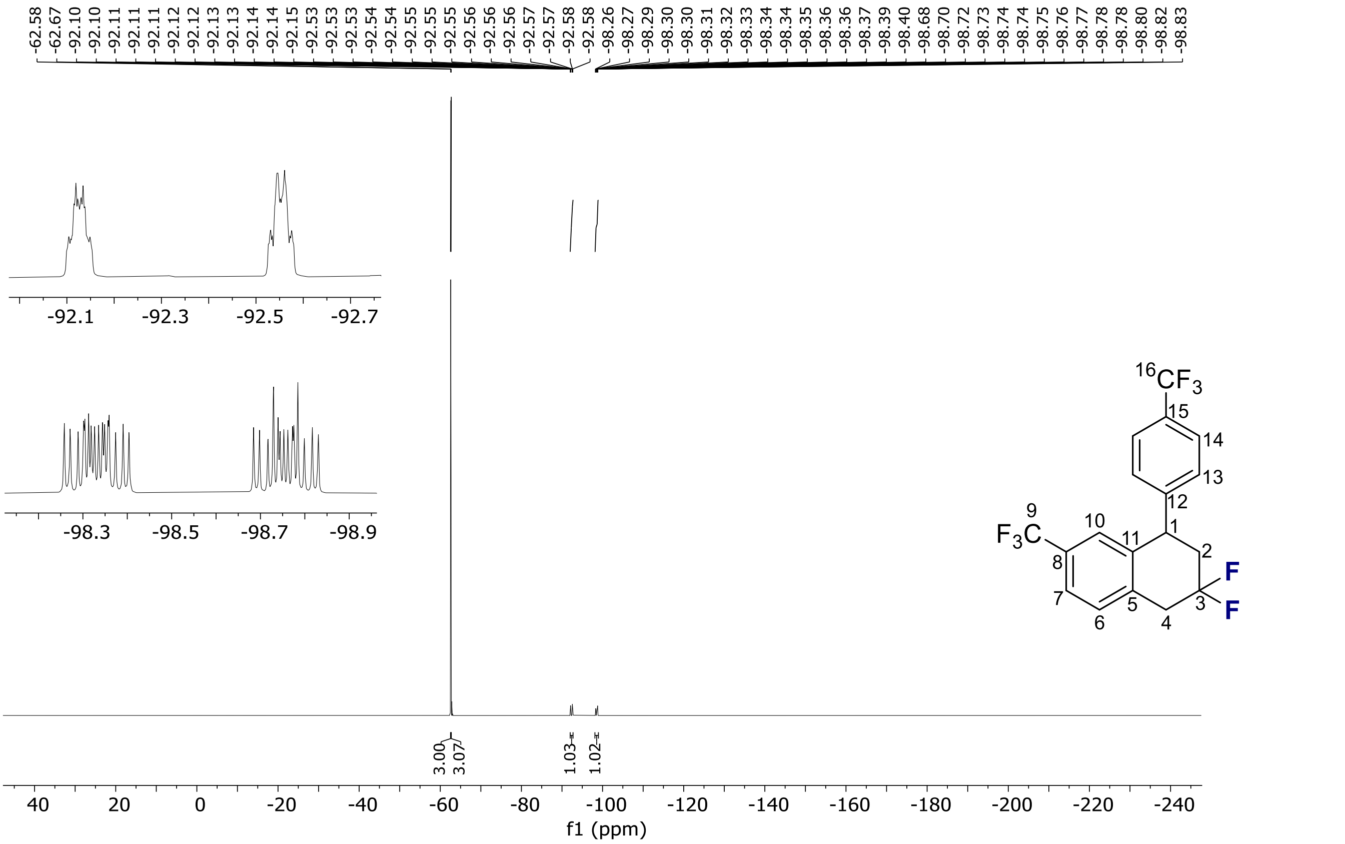

**Supplementary Figure 211.** $^{19}F$ NMR of **3e** (470 MHz, 299 K, $CDCl_3$).

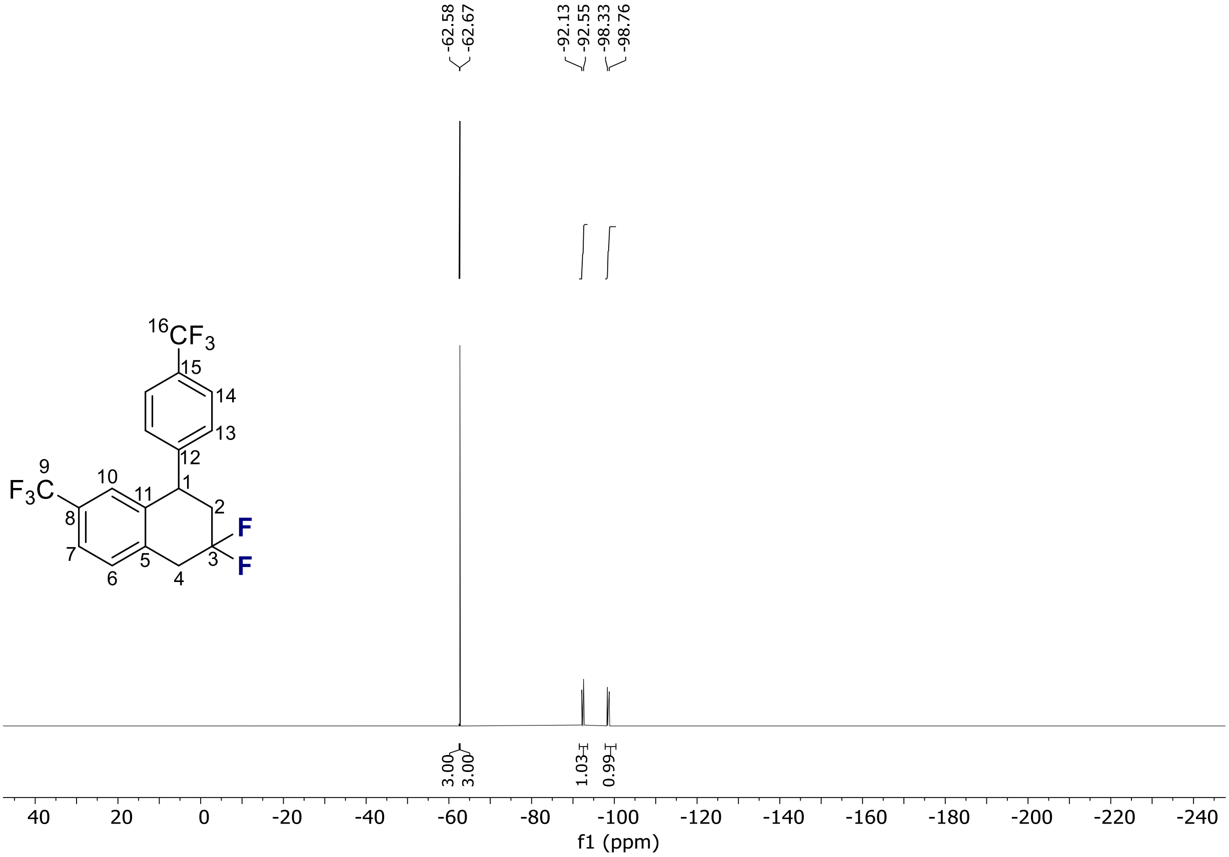

**Supplementary Figure 212.** $^{19}F\{^{1}H\}$ NMR of **3e** (470 MHz, 299 K, $CDCl_3$).

**3,3,7-Trifluoro-1-phenyl-1,2,3,4-tetrahydronaphthalene (3f)**

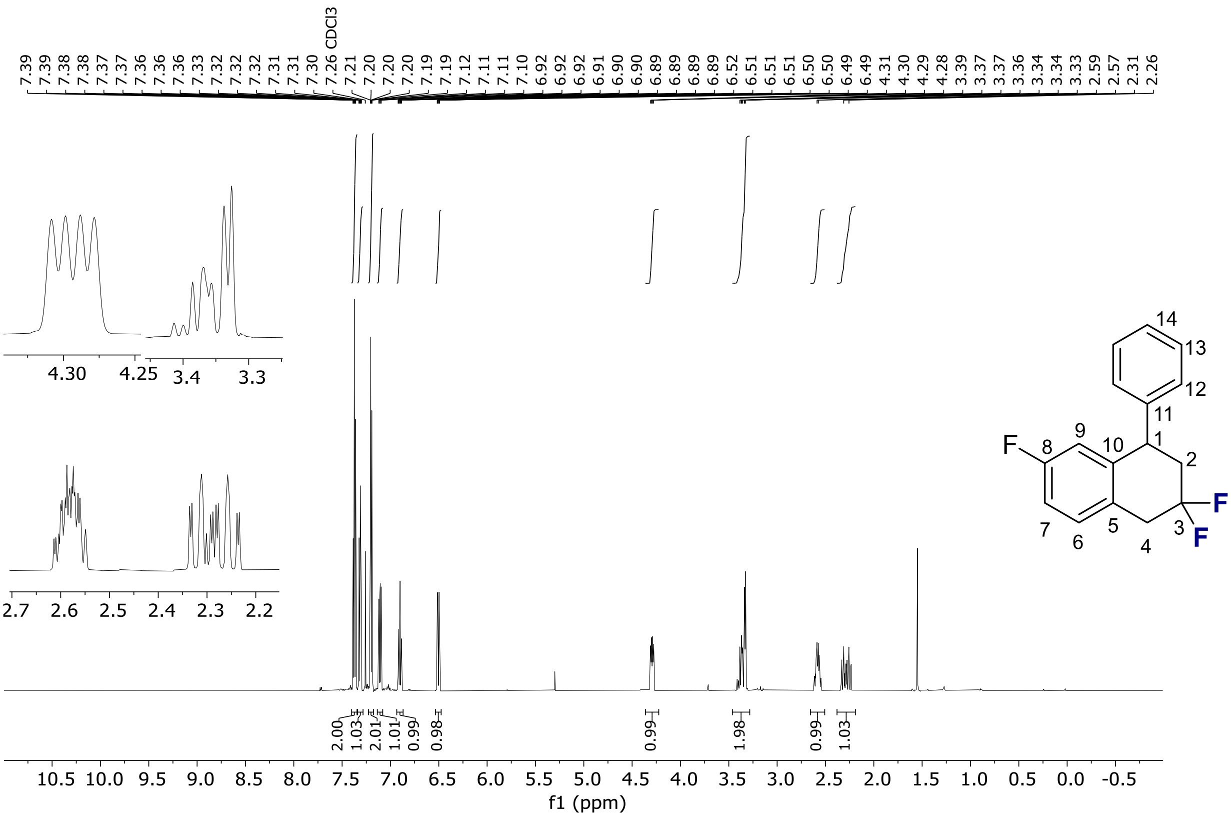

**Supplementary Figure 213.** $^{1}$H NMR of **3f** (599 MHz, 299 K, $CDCl_3$).

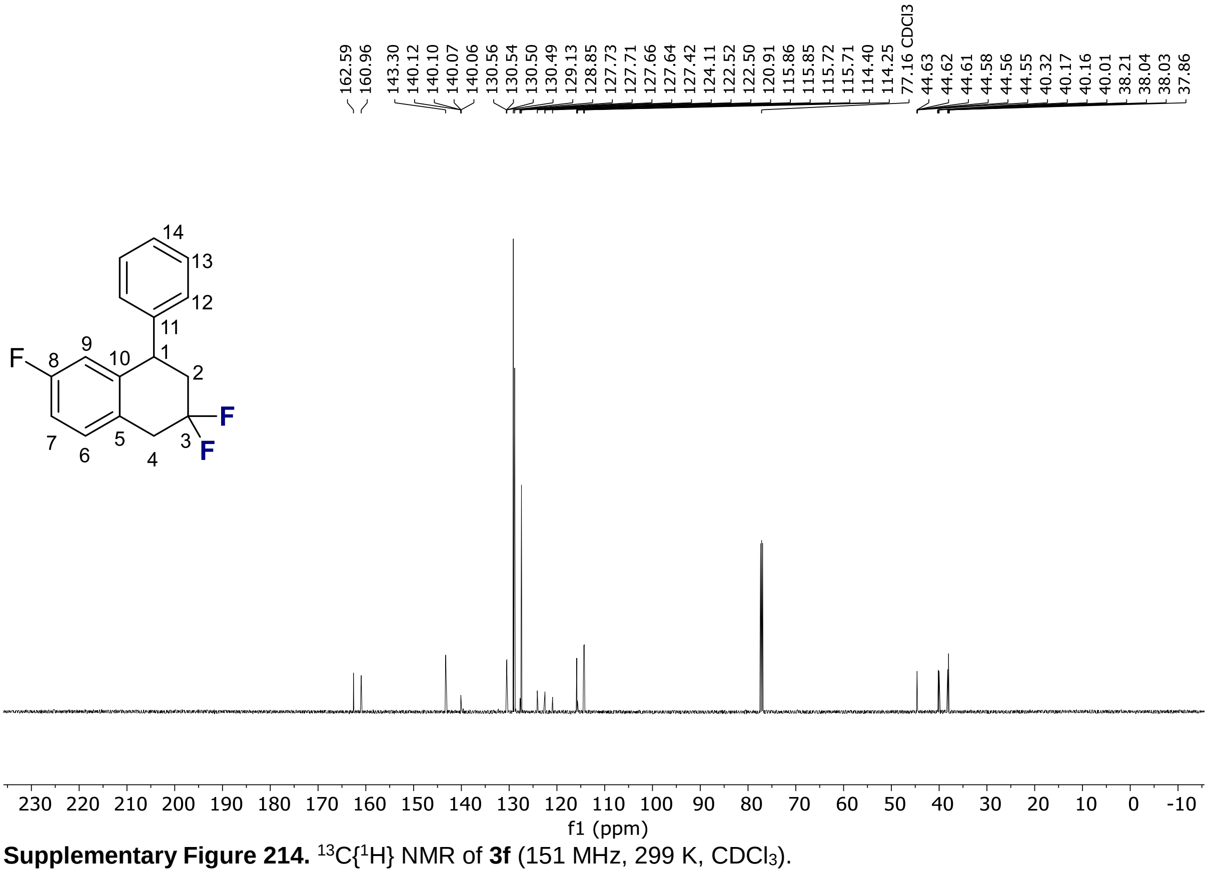

**Supplementary Figure 214.** $^{13}C\{^{1}H\}$ NMR of **3f** (151 MHz, 299 K, $CDCl_3$).

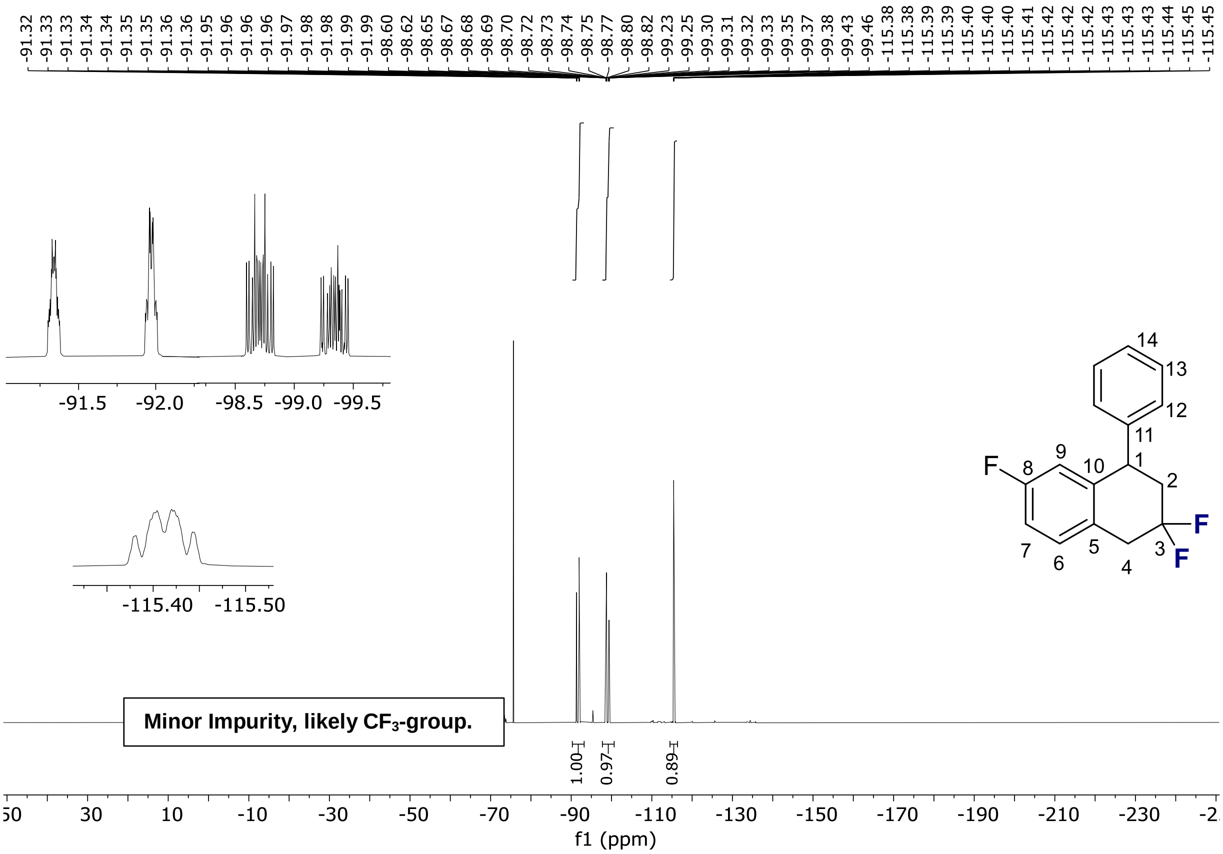


**Supplementary Figure 215.** $^{19}$F NMR of **3f** (376 MHz, 299 K, $CDCl_3$).

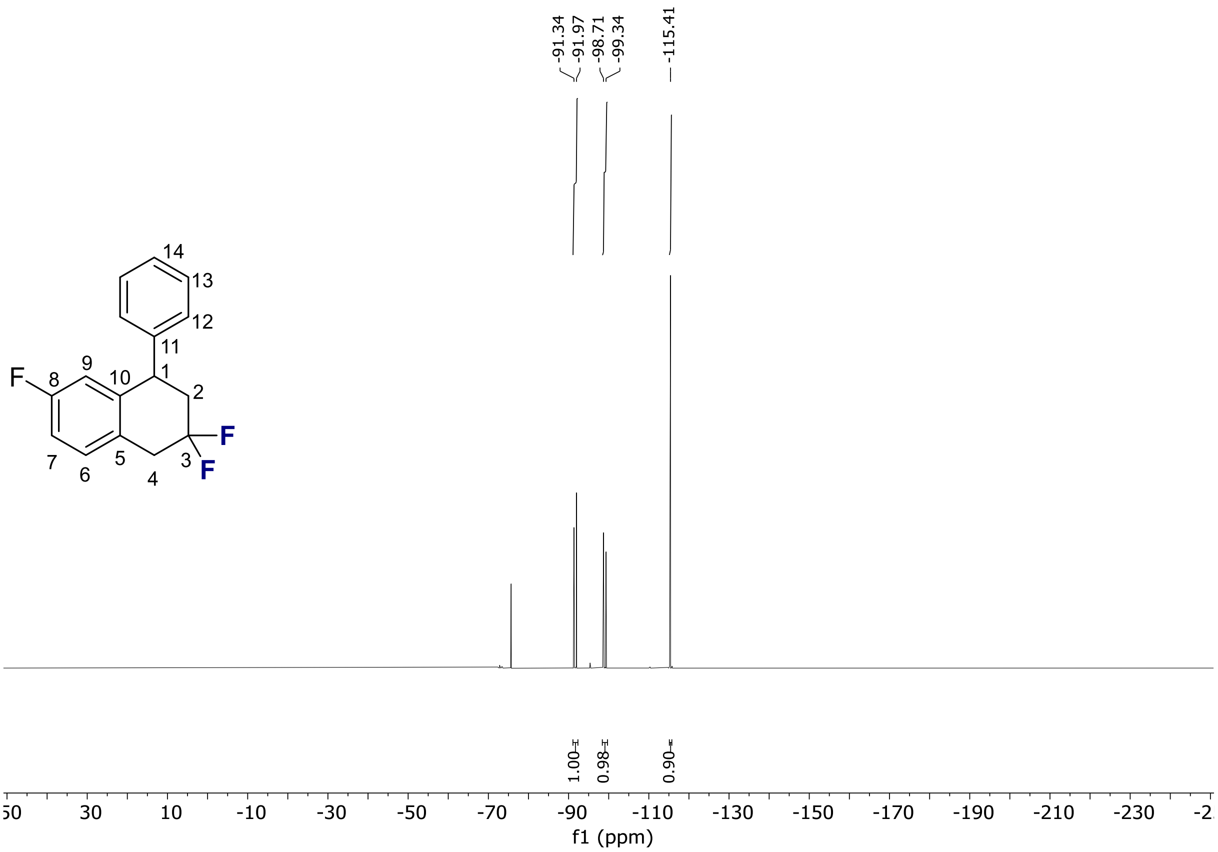


**Supplementary Figure 216.** $^{19}$F{$^1$H} NMR of **3f** (376 MHz, 299 K, $CDCl_3$).

## 7-Chloro-3,3-difluoro-1-phenyl-1,2,3,4-tetrahydronaphthalene (3g)

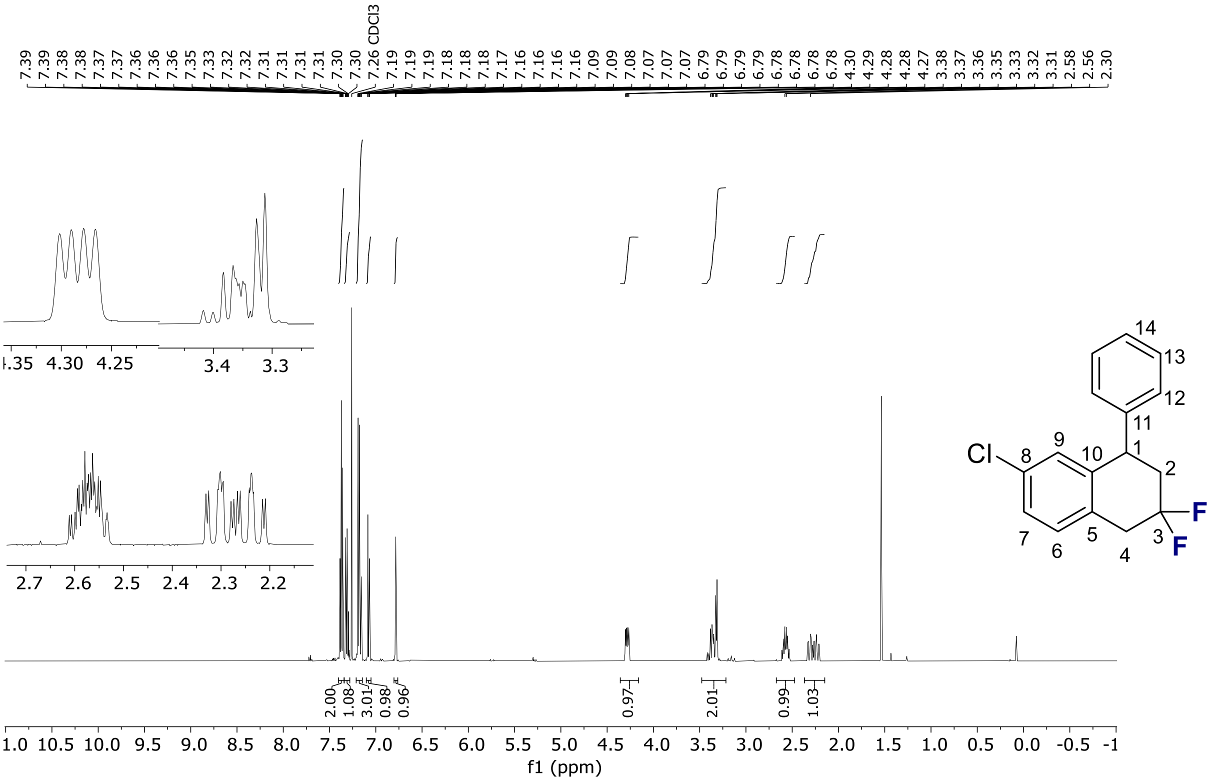


**Supplementary Figure 217.** $^{1}H$ NMR of **3g** (500 MHz, 299 K, $CDCl_3$).

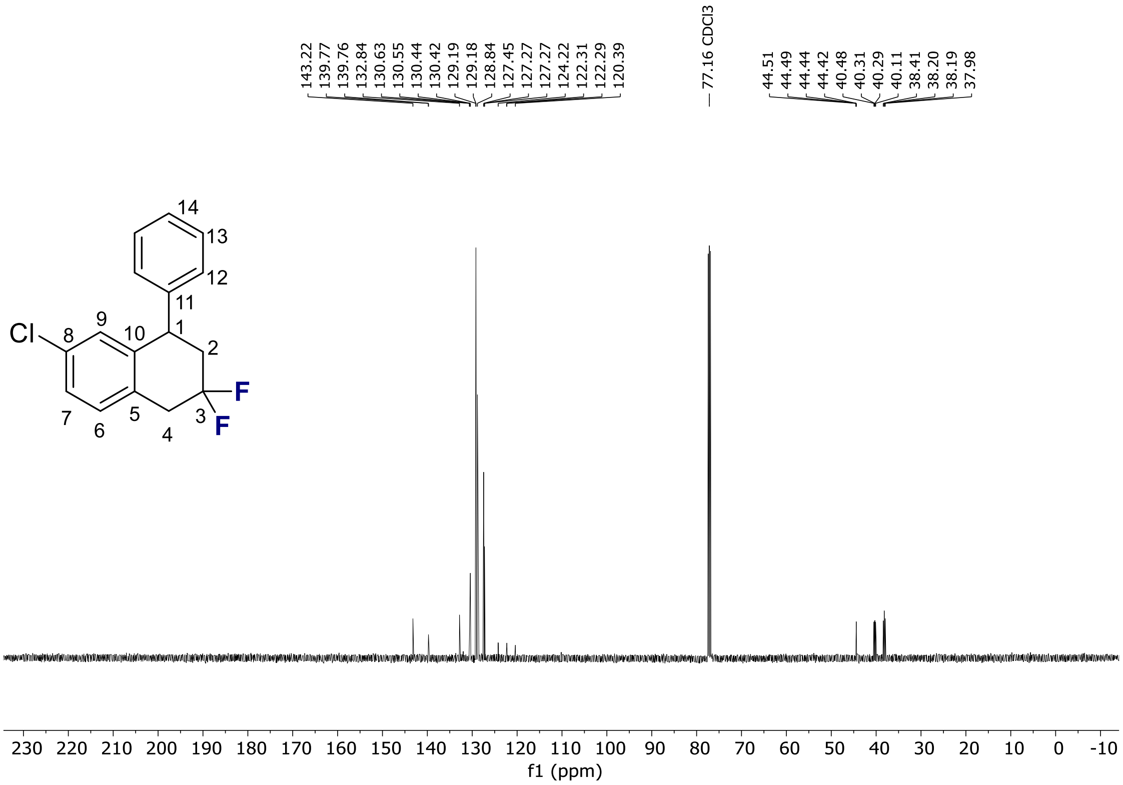


**Supplementary Figure 218.** $^{13}C\{^{1}H\}$ NMR of **3g** (126 MHz, 299 K, $CDCl_3$).

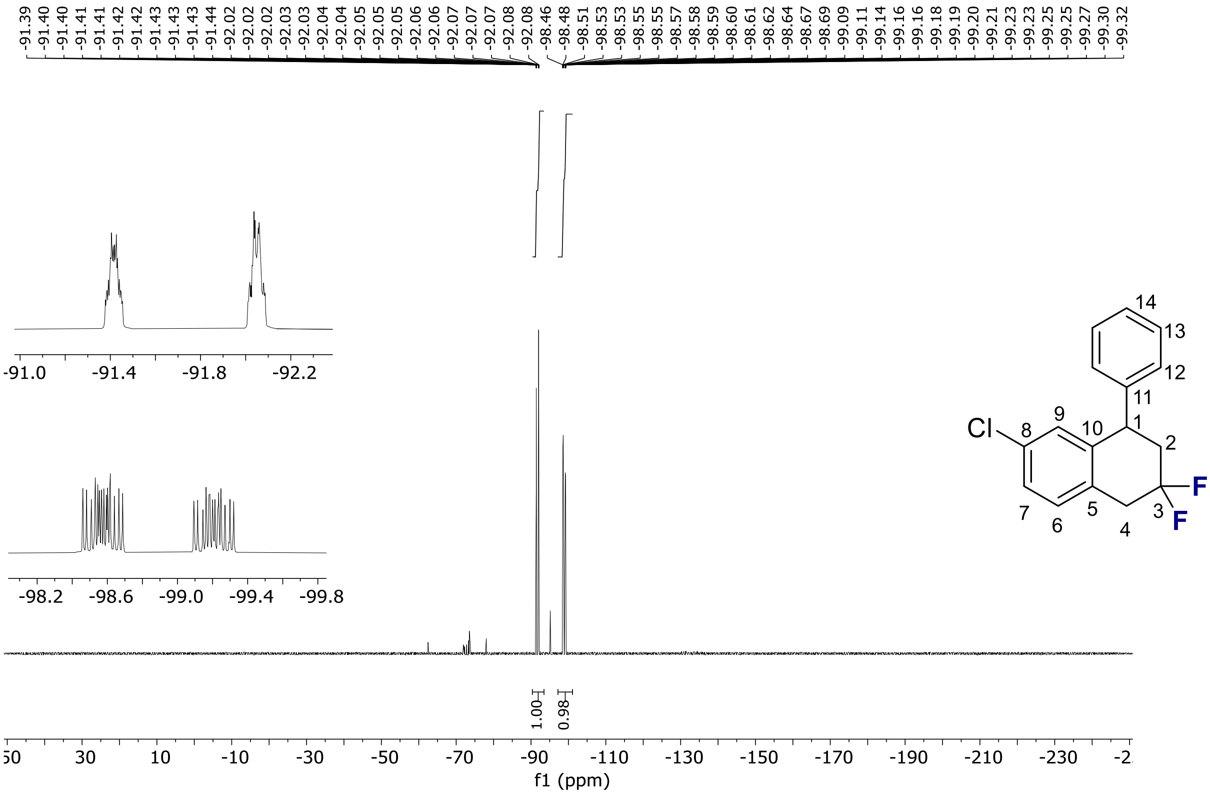


**Supplementary Figure 219.** $^{19}$F NMR of **3g** (376 MHz, 299 K, $CDCl_3$).

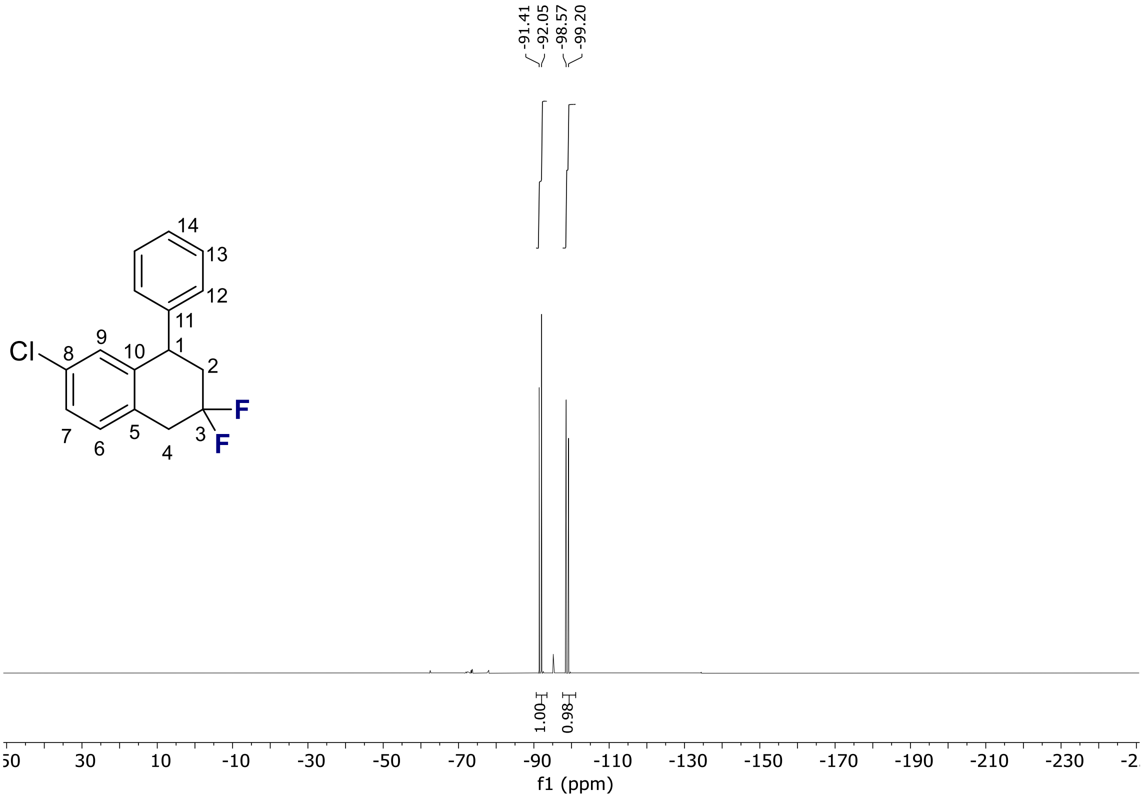


**Supplementary Figure 220.** $^{19}$F{$^1$H} NMR of **3g** (376 MHz, 299 K, $CDCl_3$).

## 7-Bromo-3,3-difluoro-1-phenyl-1,2,3,4-tetrahydronaphthalene (3h)

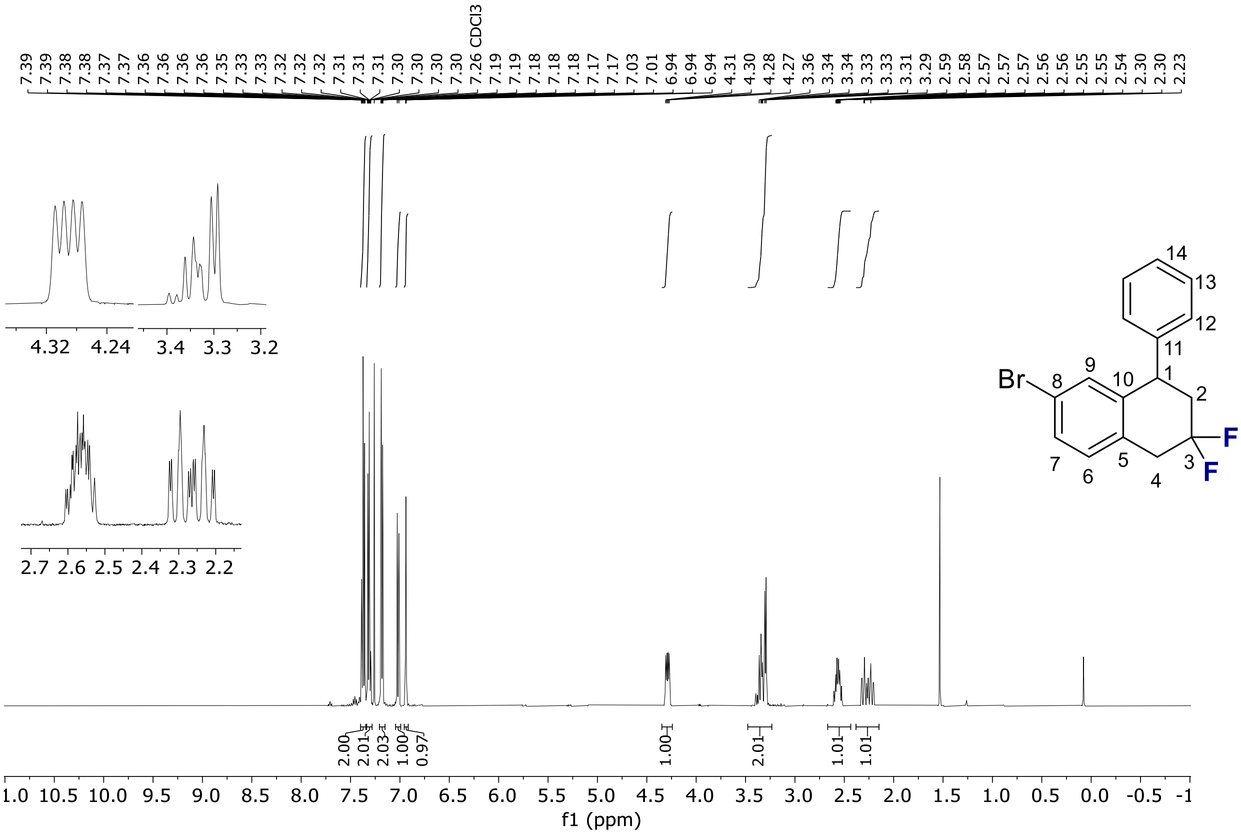


**Supplementary Figure 221.** $^{1}$H NMR of **3h** (500 MHz, 299 K, $CDCl_3$).

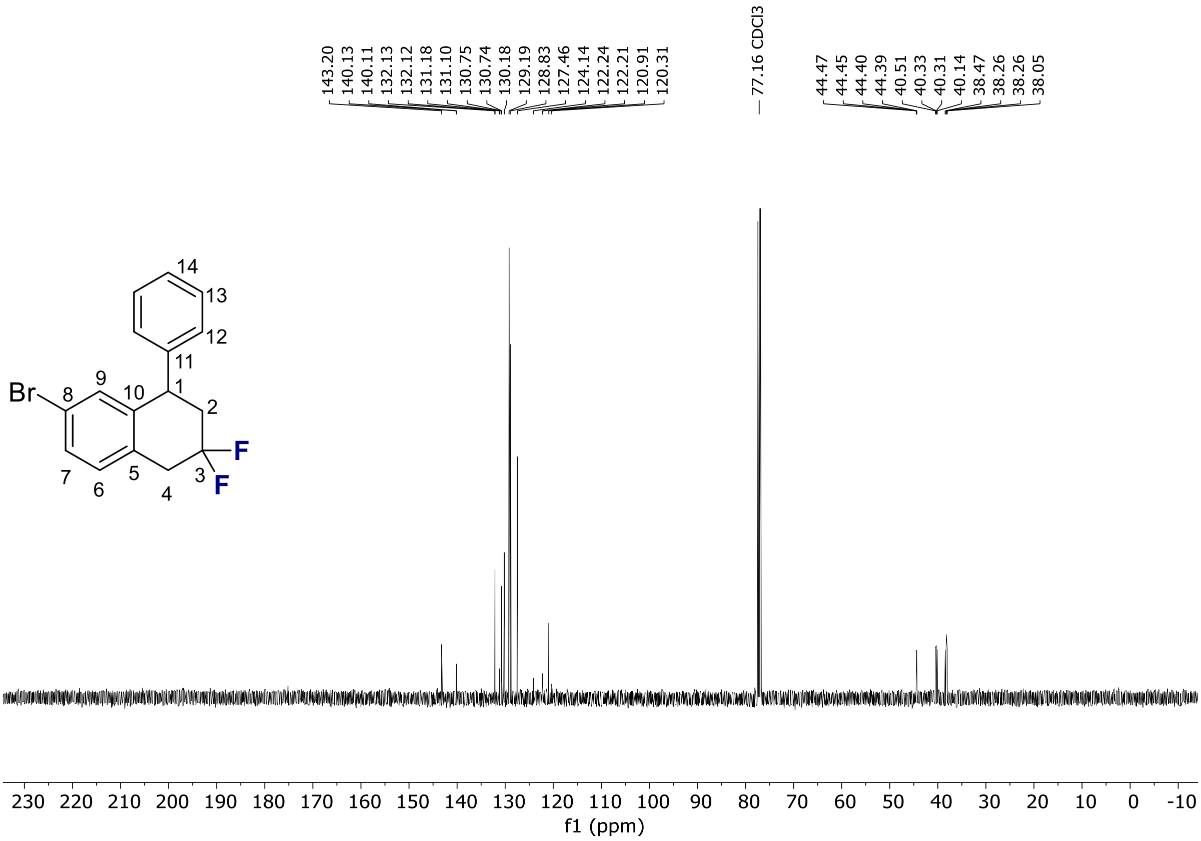


**Supplementary Figure 222.** $^{13}C\{^{1}H\}$ NMR of **3h** (126 MHz, 299 K, $CDCl_3$).

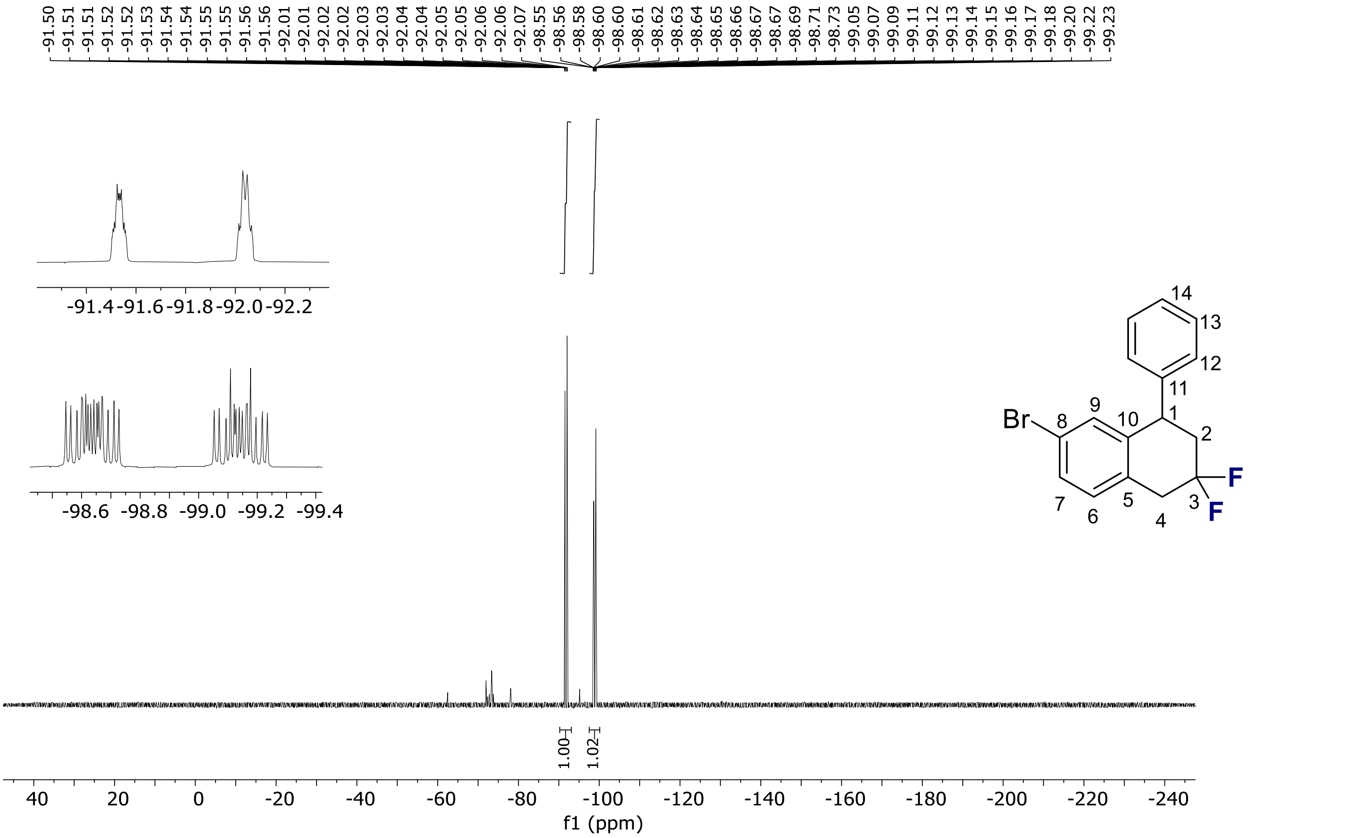

**Supplementary Figure 223.** $^{19}F$ NMR of **3h** (470 MHz, 299 K, $CDCl_3$).

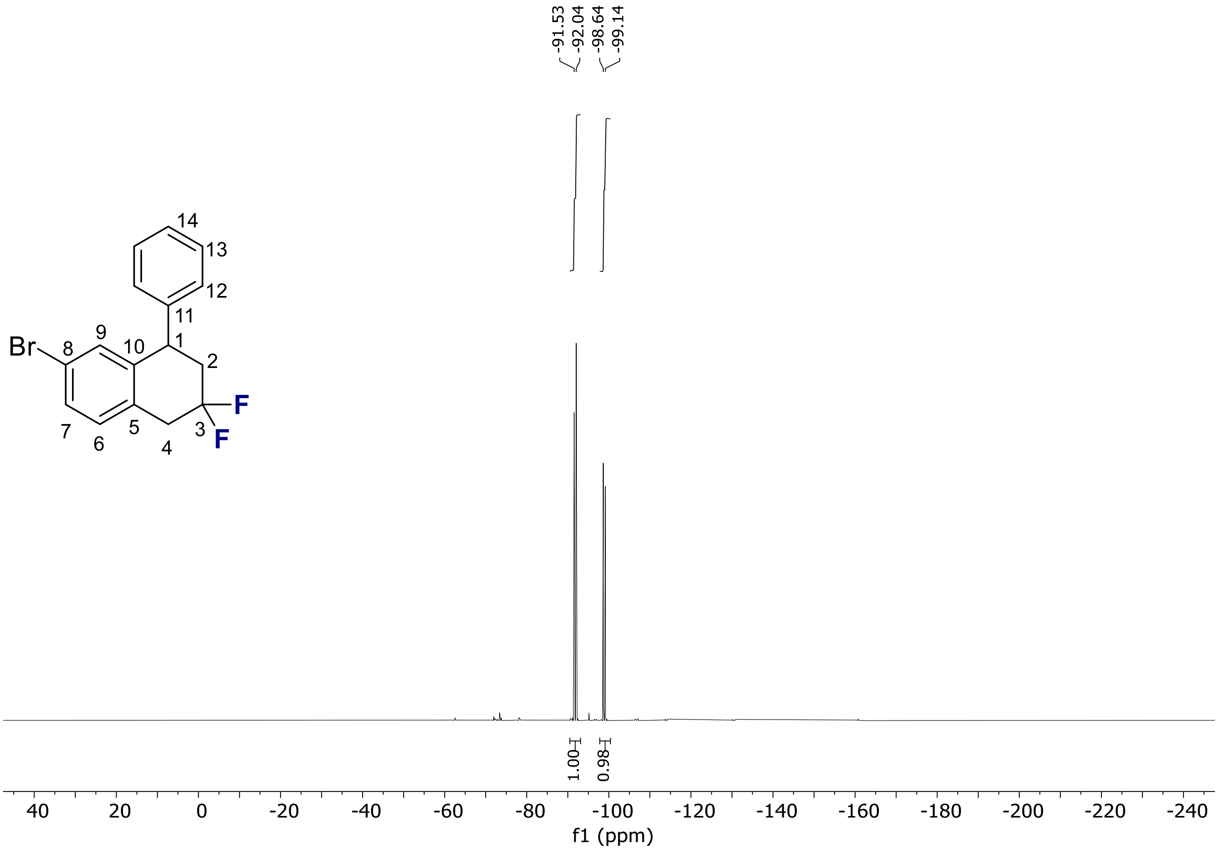

**Supplementary Figure 224.** $^{19}F\{^1H\}$ NMR of **3h** (470 MHz, 299 K, $CDCl_3$).

## 3,3-Difluoro-1-phenyl-7-(trifluoromethoxy)-1,2,3,4-tetrahydronaphthalene (3i)

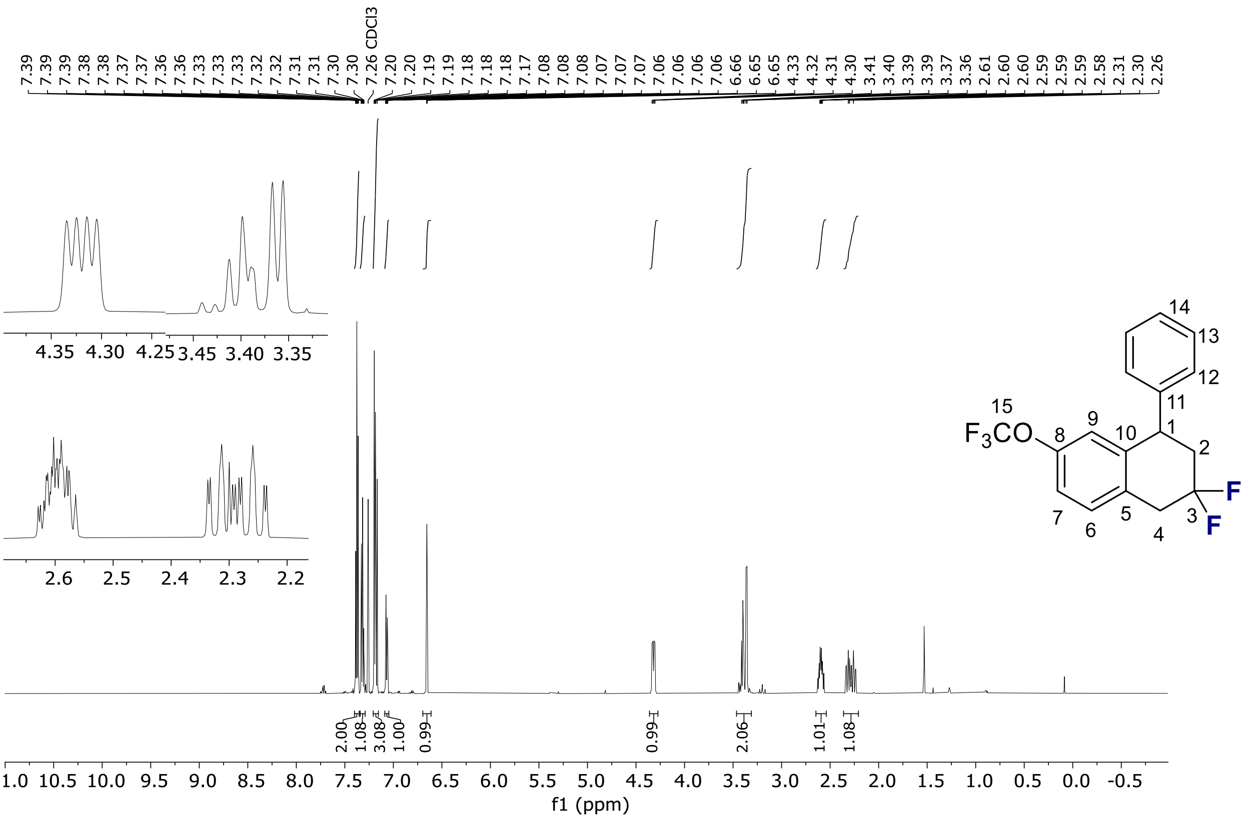


**Supplementary Figure 225.** $^{1}$H NMR of **3i** (599 MHz, 299 K, $CDCl_3$).

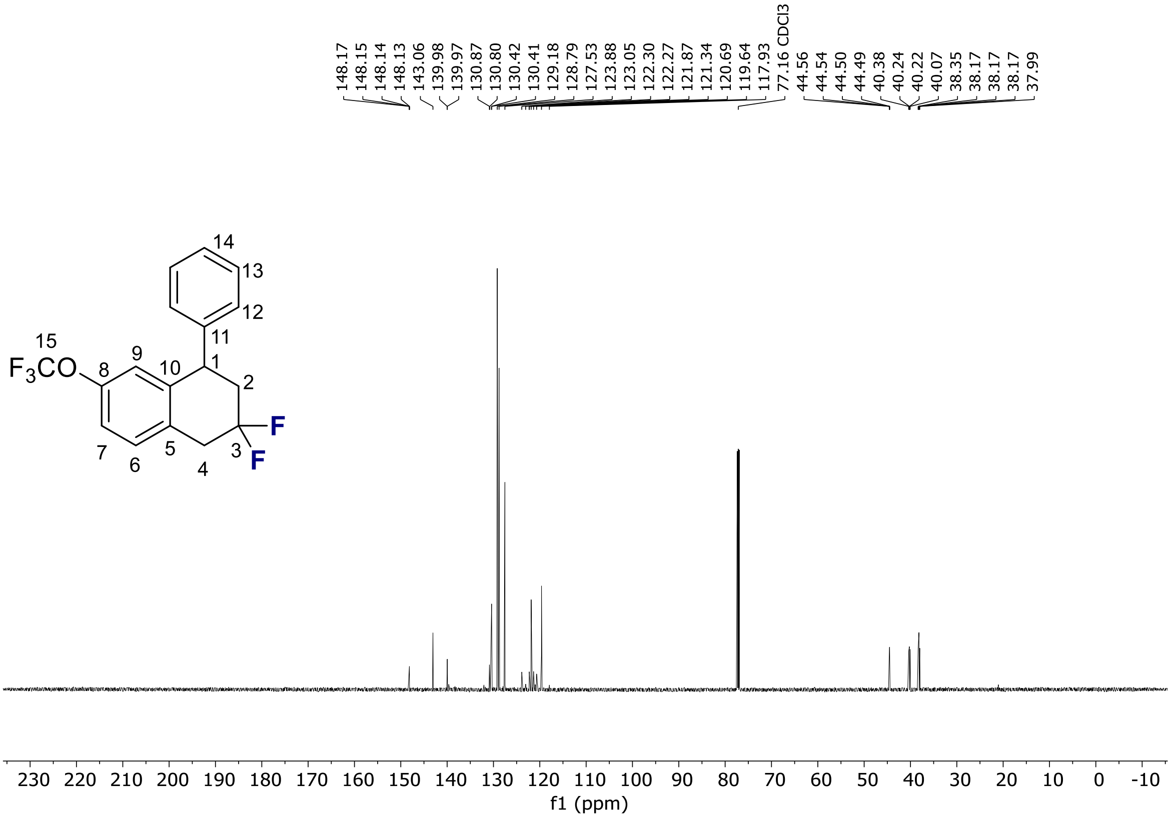


**Supplementary Figure 226.** $^{13}C\{^{1}H\}$ NMR of **3i** (151 MHz, 299 K, $CDCl_3$).

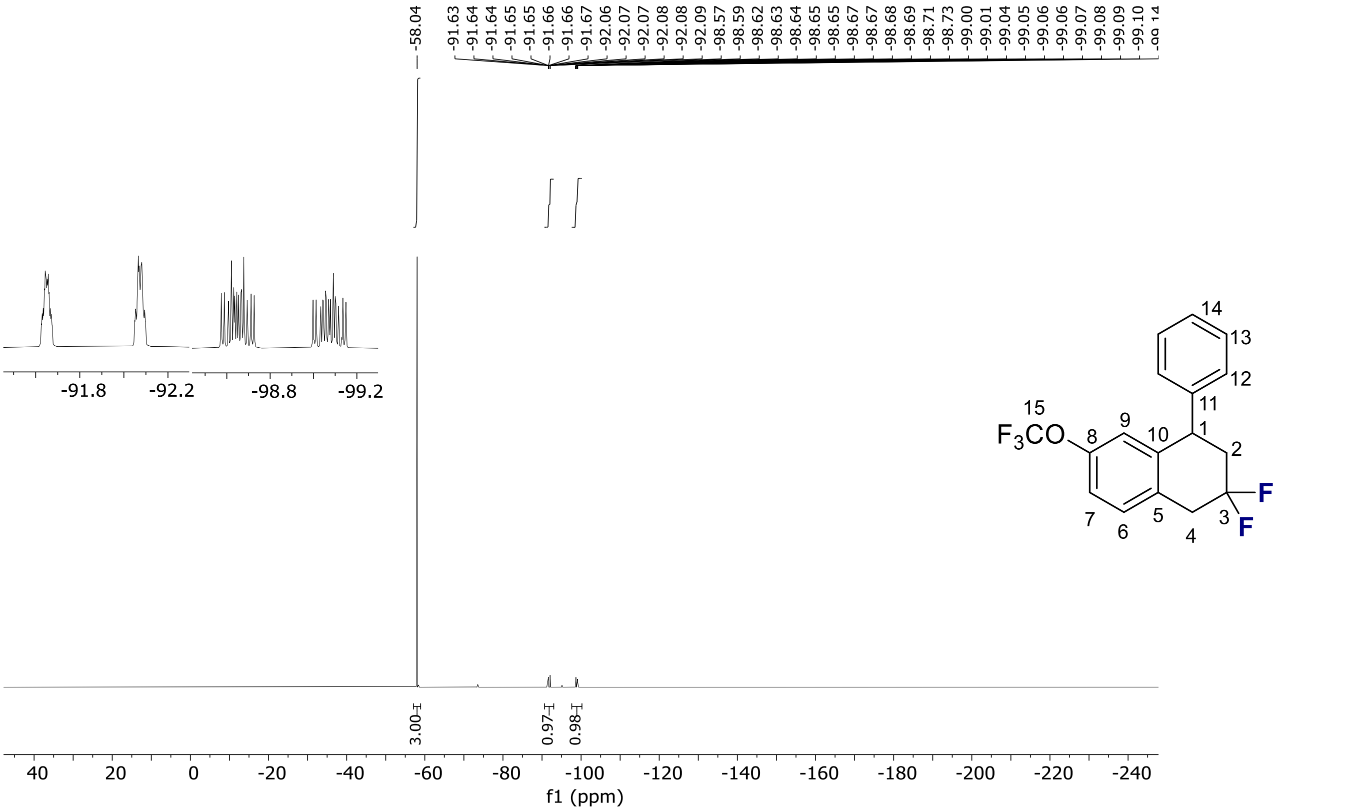

**Supplementary Figure 227.** $^{19}$F NMR of **3i** (564 MHz, 299 K, $CDCl_3$).

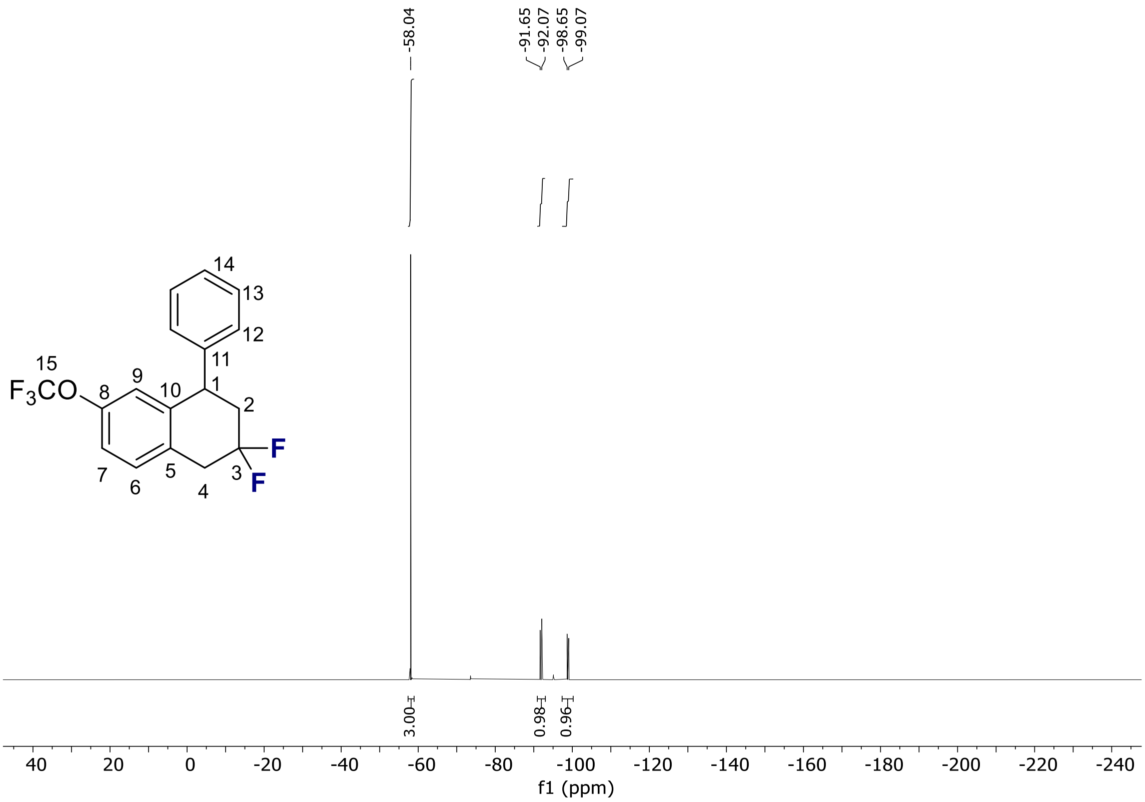

**Supplementary Figure 228.** $^{19}F\{^1H\}$ NMR of **3i** (564 MHz, 299 K, $CDCl_3$).

## 3,3-Difluoro-1-phenyl-7-(trifluoromethyl)-1,2,3,4-tetrahydronaphthalene (3j)

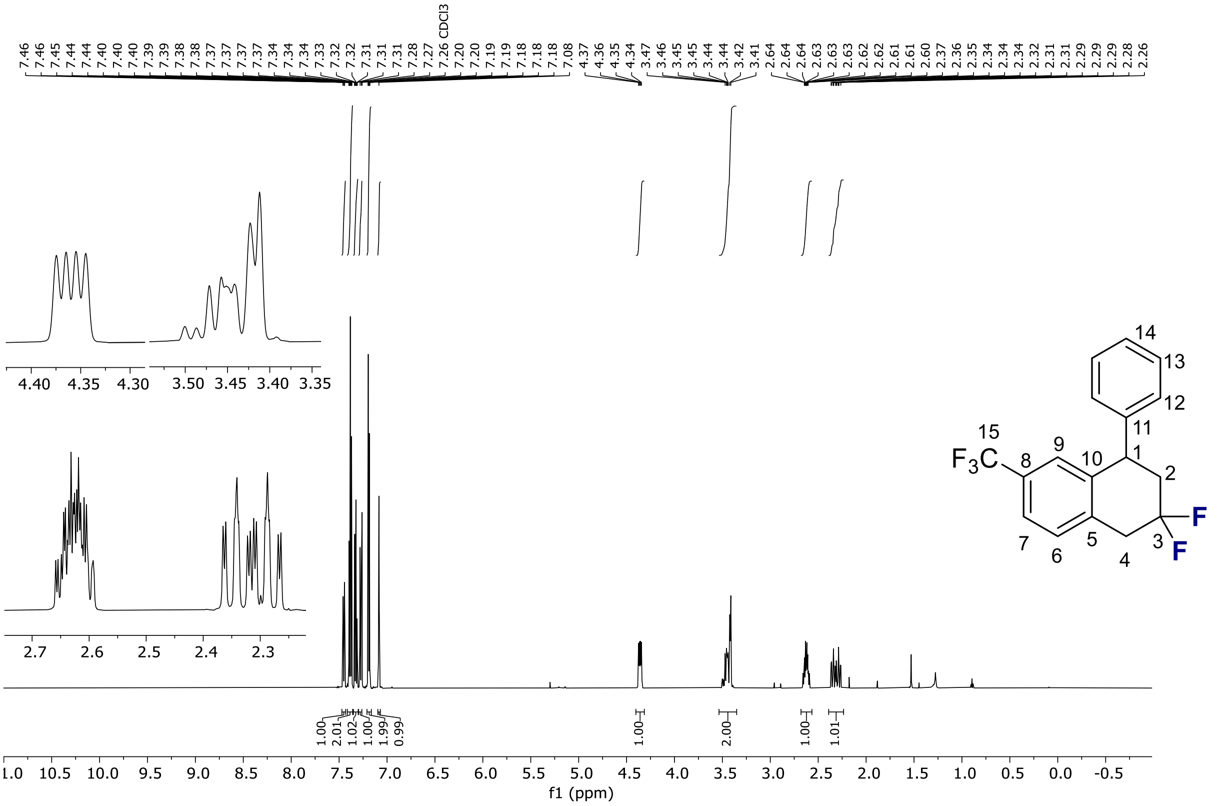


**Supplementary Figure 229.** $^{1}$H NMR of **3j** (599 MHz, 299 K, $CDCl_3$).

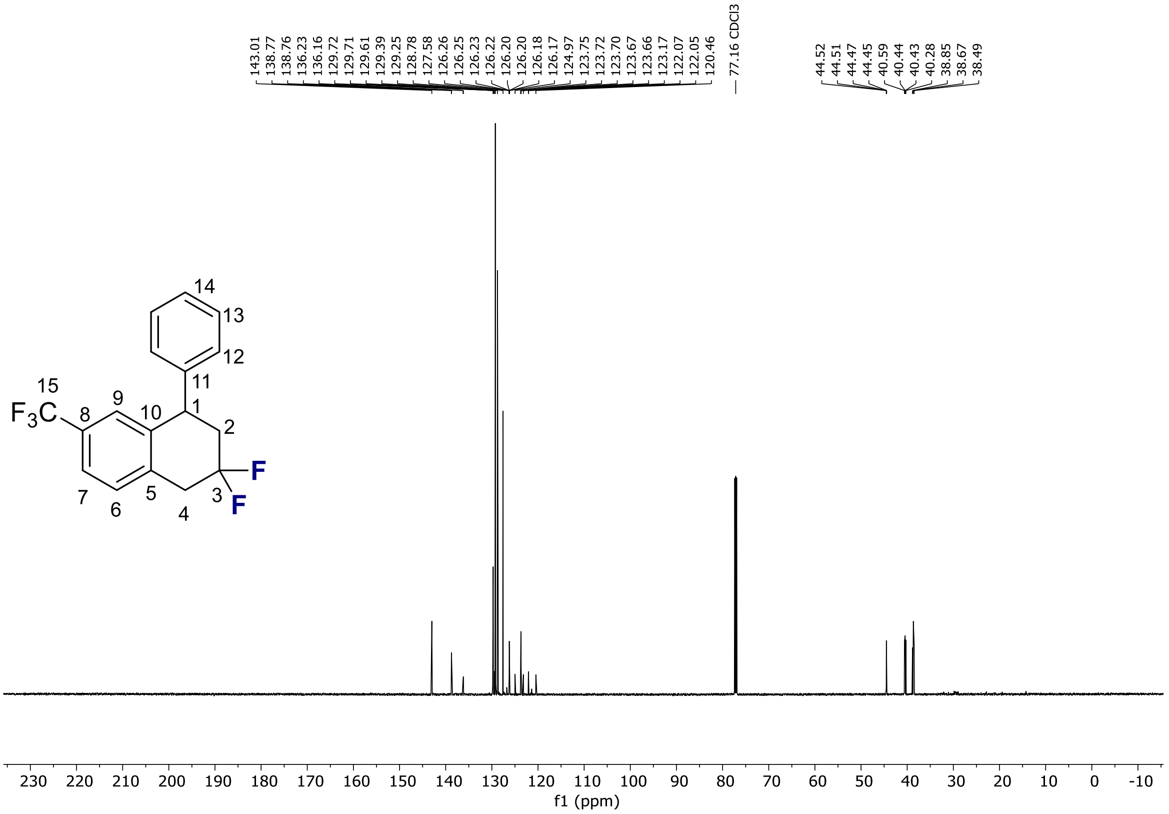


**Supplementary Figure 230.** $^{13}$C{$^{1}$H} NMR of **3j** (151 MHz, 299 K, $CDCl_3$).

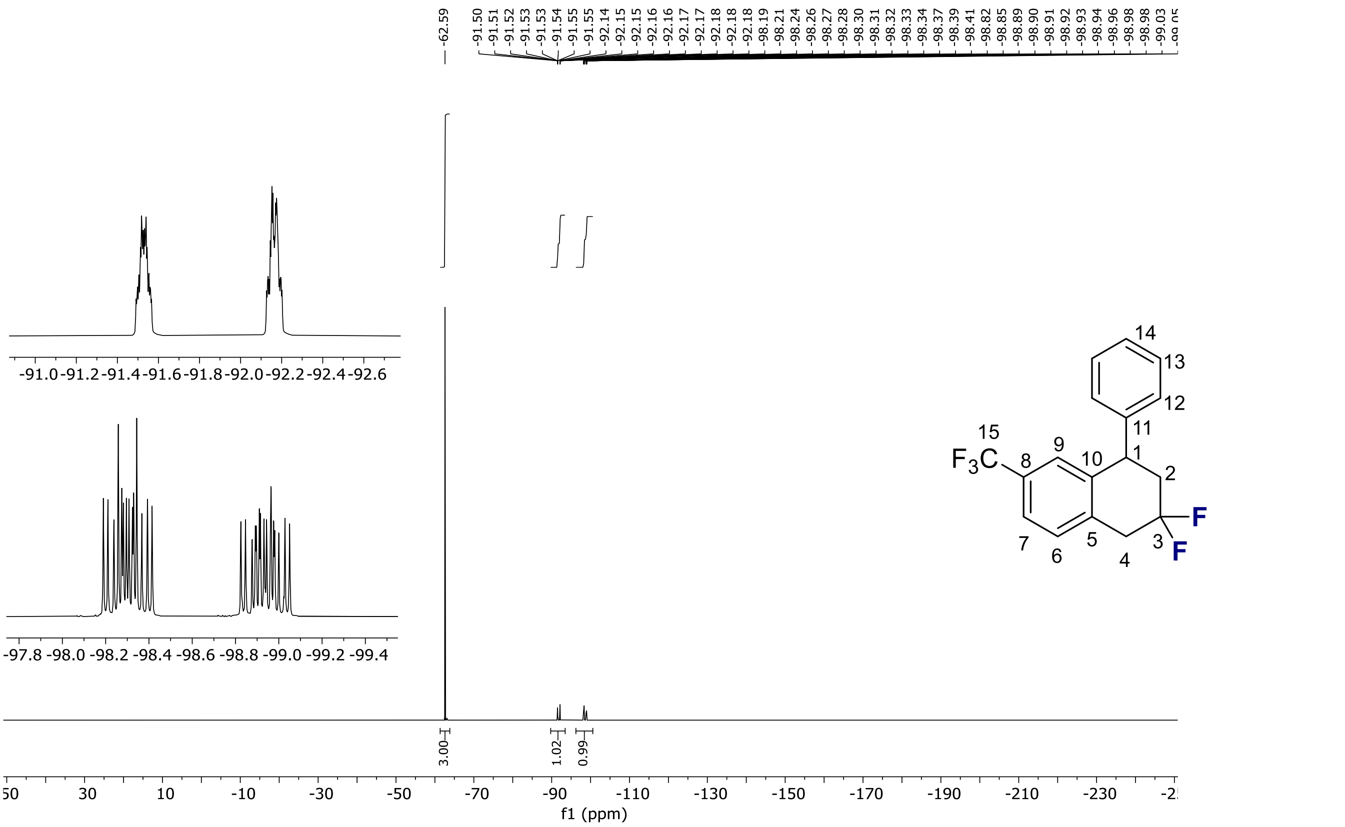


**Supplementary Figure 231.** $^{19}F$ NMR of **3j** (376 MHz, 299 K, $CDCl_3$).

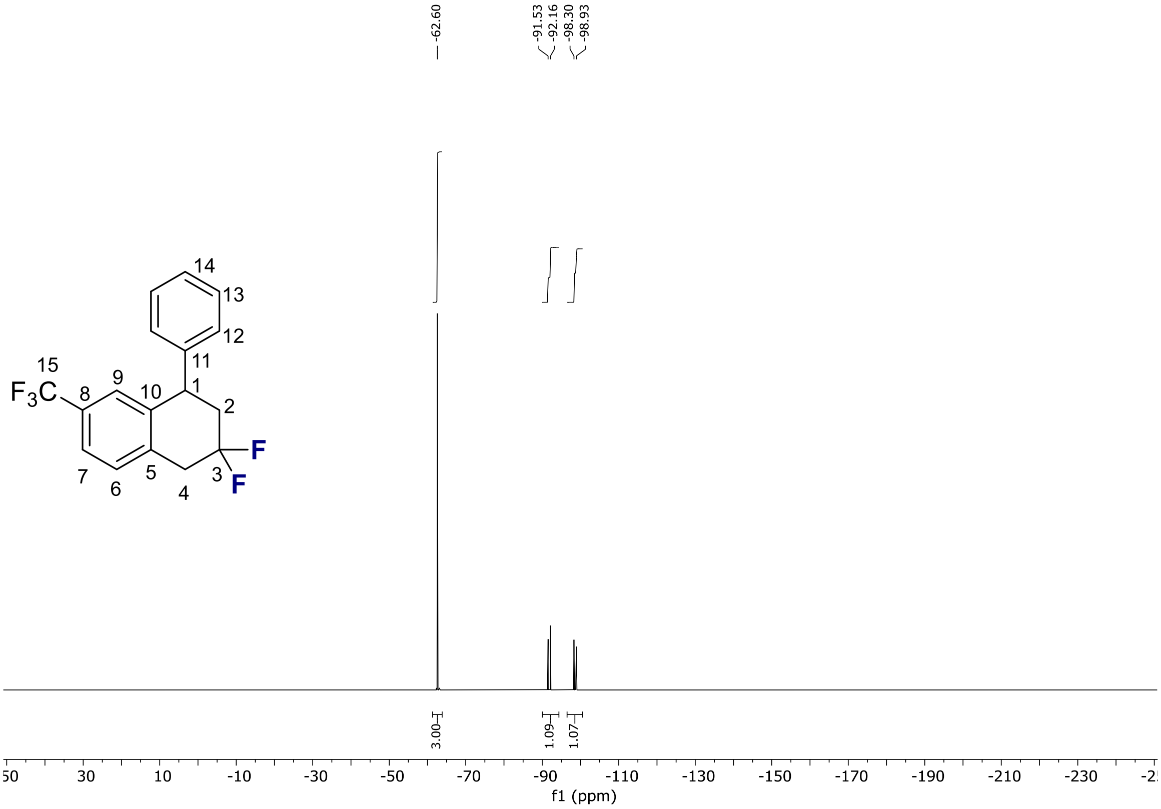


**Supplementary Figure 232.** $^{19}F\{^1H\}$ NMR of **3j** (376 MHz, 299 K, $CDCl_3$).

## 3,3-Difluoro-1-phenyl-7-(4-(trifluoromethyl)phenyl)-1,2,3,4-tetrahydronaphthalene (3l)

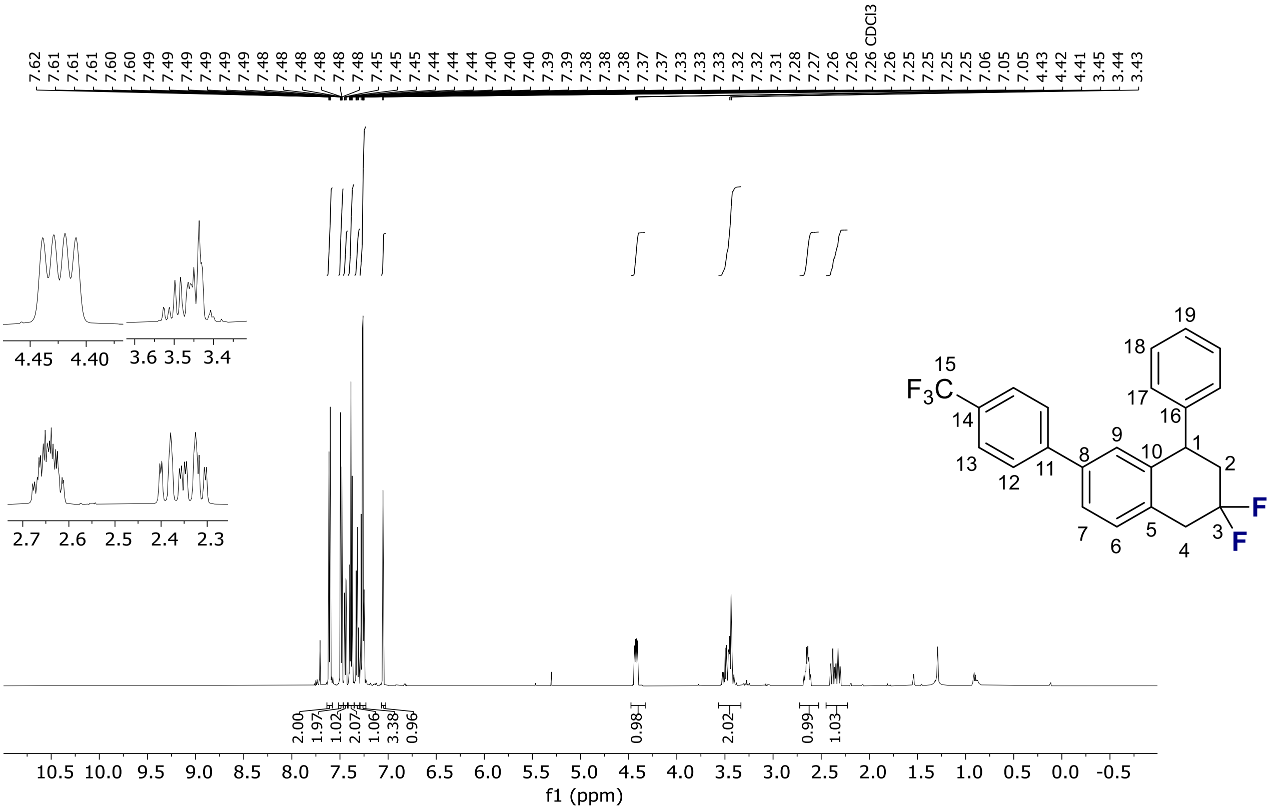


**Supplementary Figure 233.** $^{1}$H NMR of **3l** (599 MHz, 299 K, $CDCl_3$).

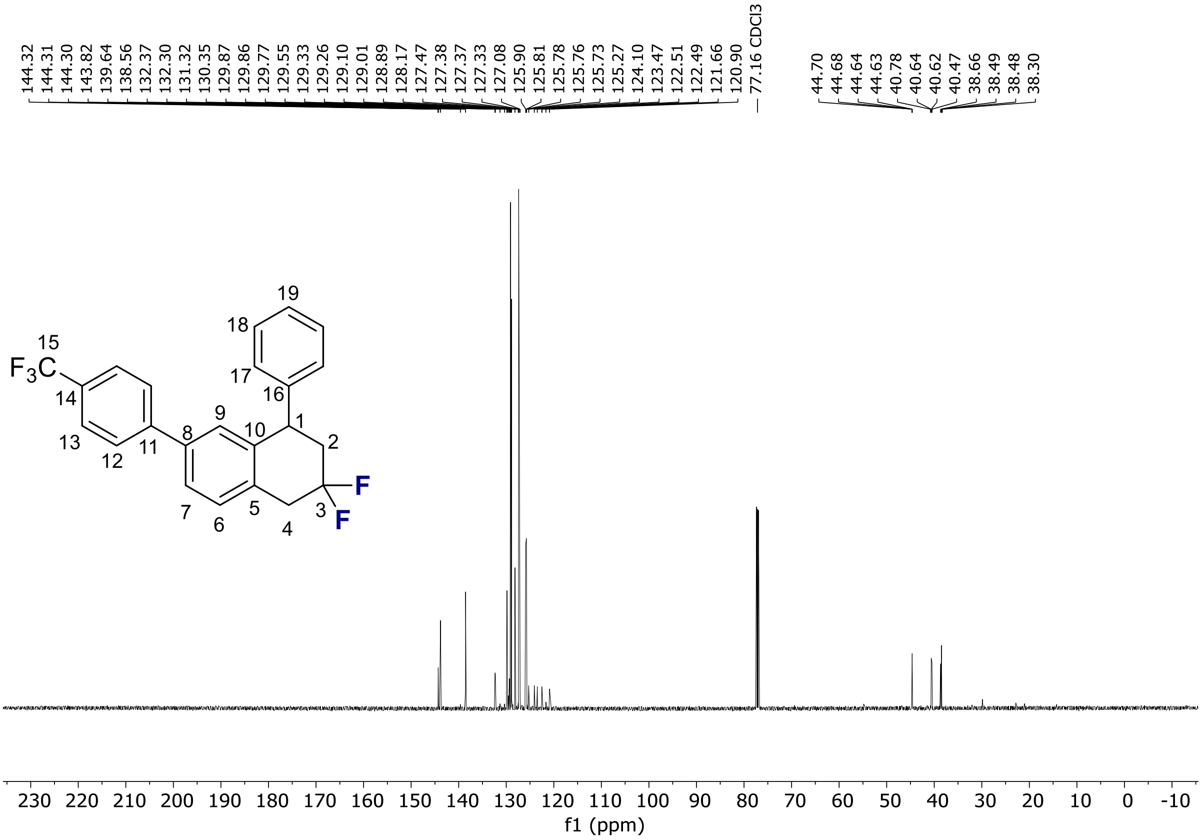


**Supplementary Figure 234.** $^{13}$C{$^{1}$H} NMR of **3l** (151 MHz, 299 K, $CDCl_3$).

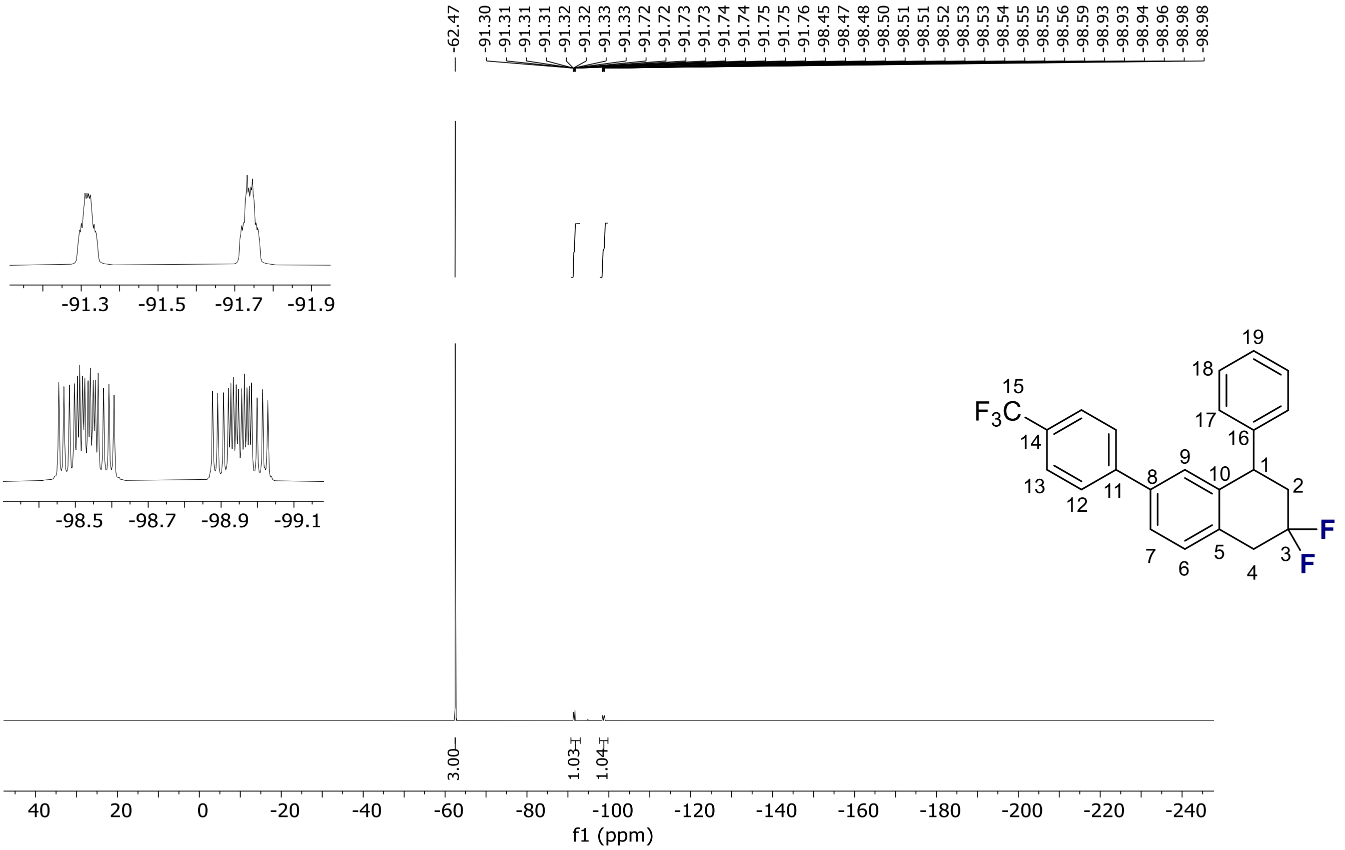


**Supplementary Figure 235.** $^{19}F$ NMR of **3l** (564 MHz, 299 K, $CDCl_3$).

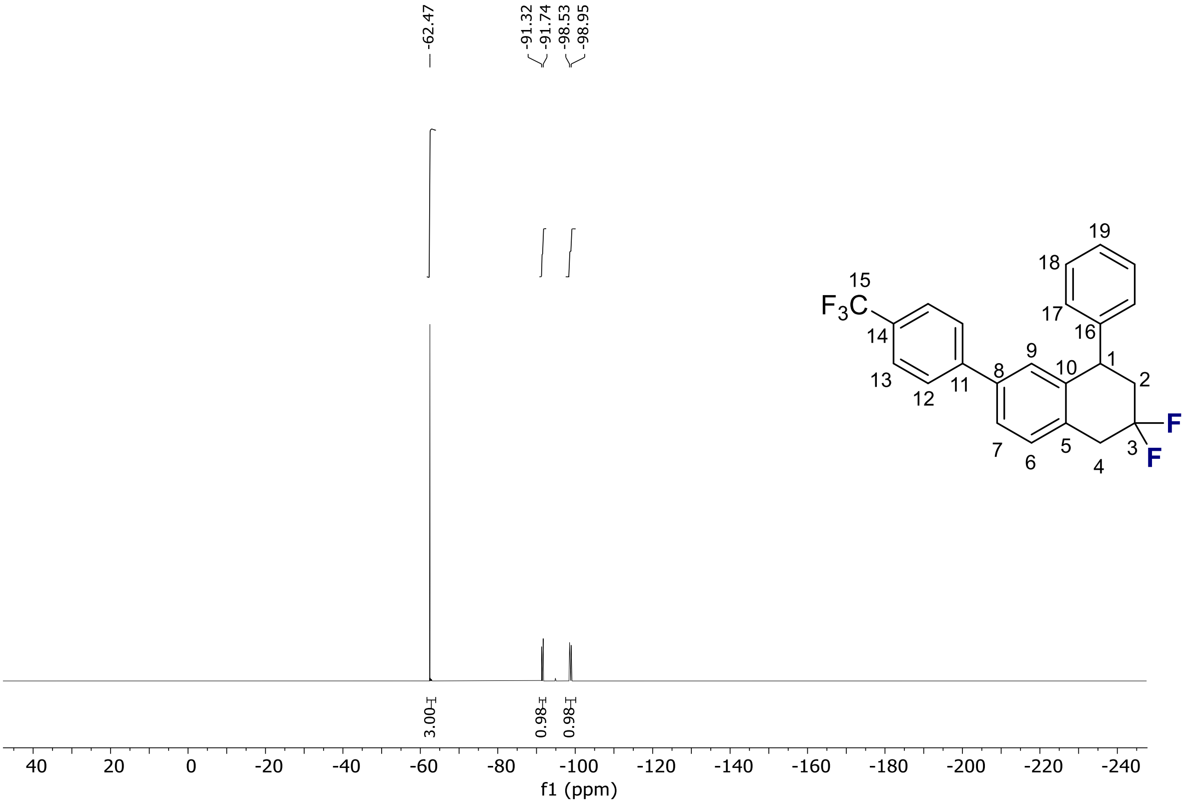


**Supplementary Figure 236.** $^{19}F\{^1H\}$ NMR of **3l** (564 MHz, 299 K, $CDCl_3$).

**6-Bromo-3,3-difluoro-1-phenyl-1,2,3,4-tetrahydronaphthalene (3m)**

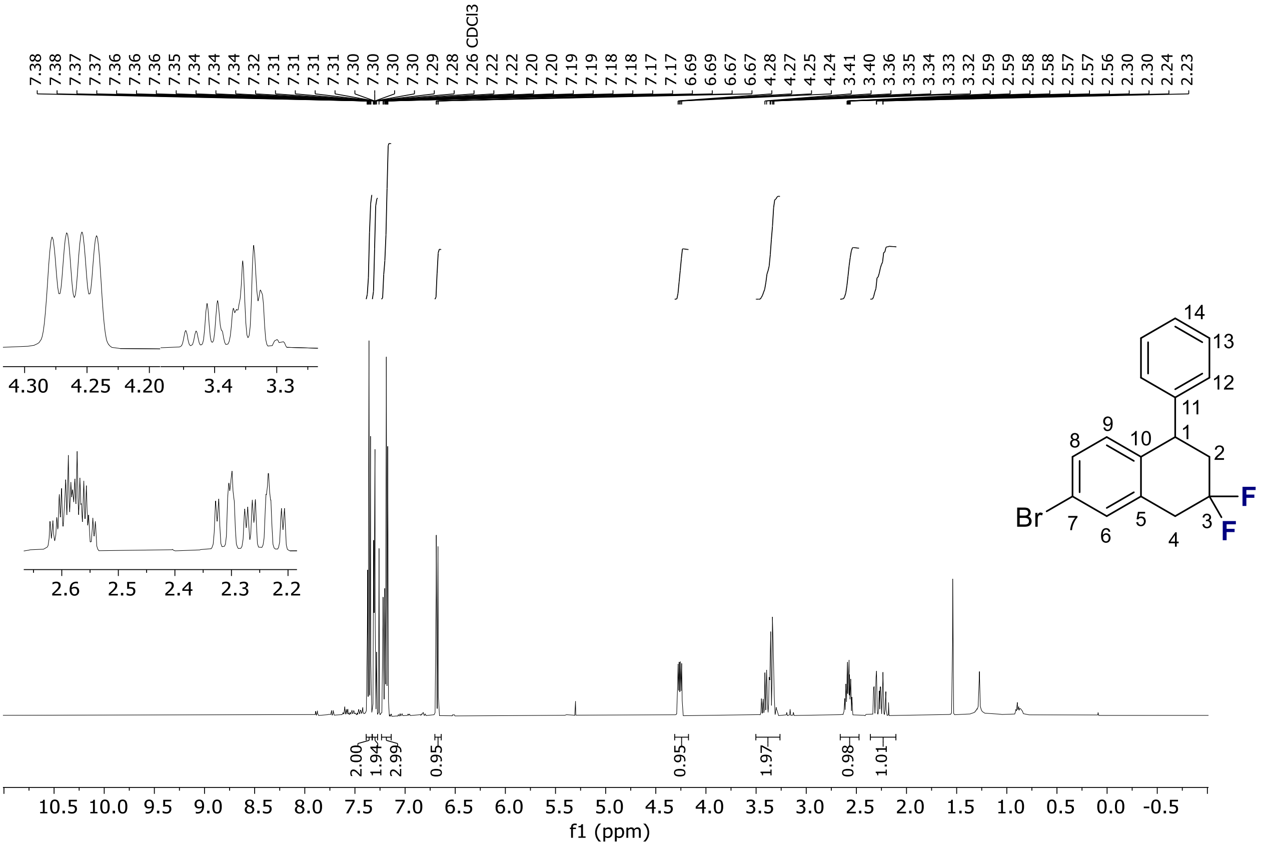

**Supplementary Figure 237.** $^{1}H$ NMR of **3m** (500 MHz, 299 K, $CDCl_3$).

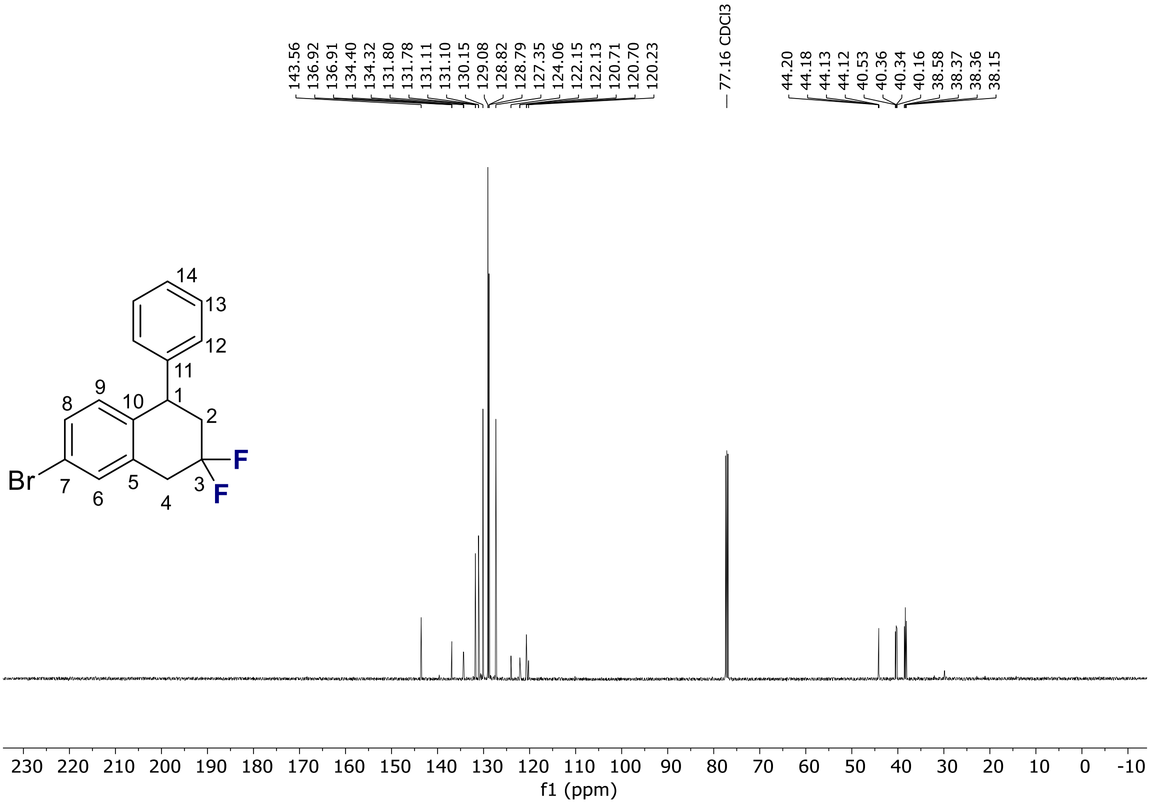

**Supplementary Figure 238.** $^{13}C\{^{1}H\}$ NMR of **3m** (126 MHz, 299 K, $CDCl_3$).

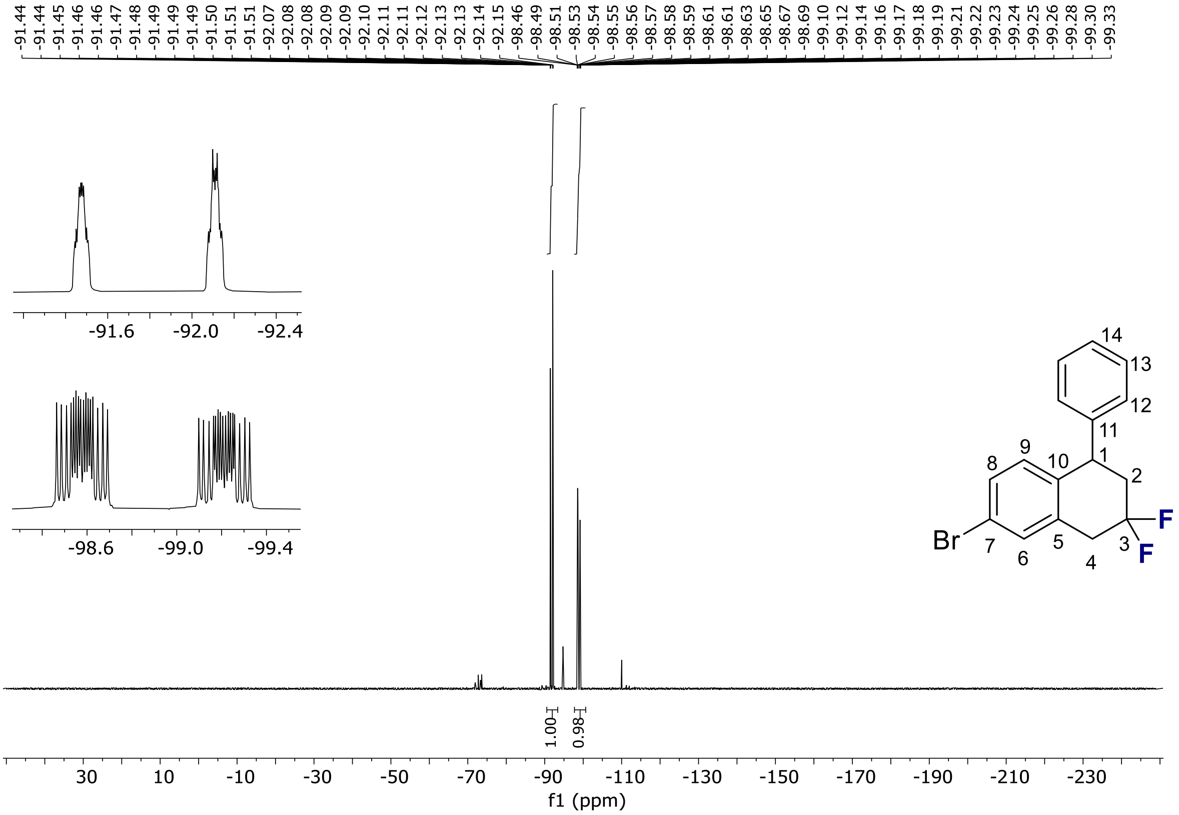


**Supplementary Figure 239.** $^{19}F$ NMR of **3m** (376 MHz, 299 K, $CDCl_3$).

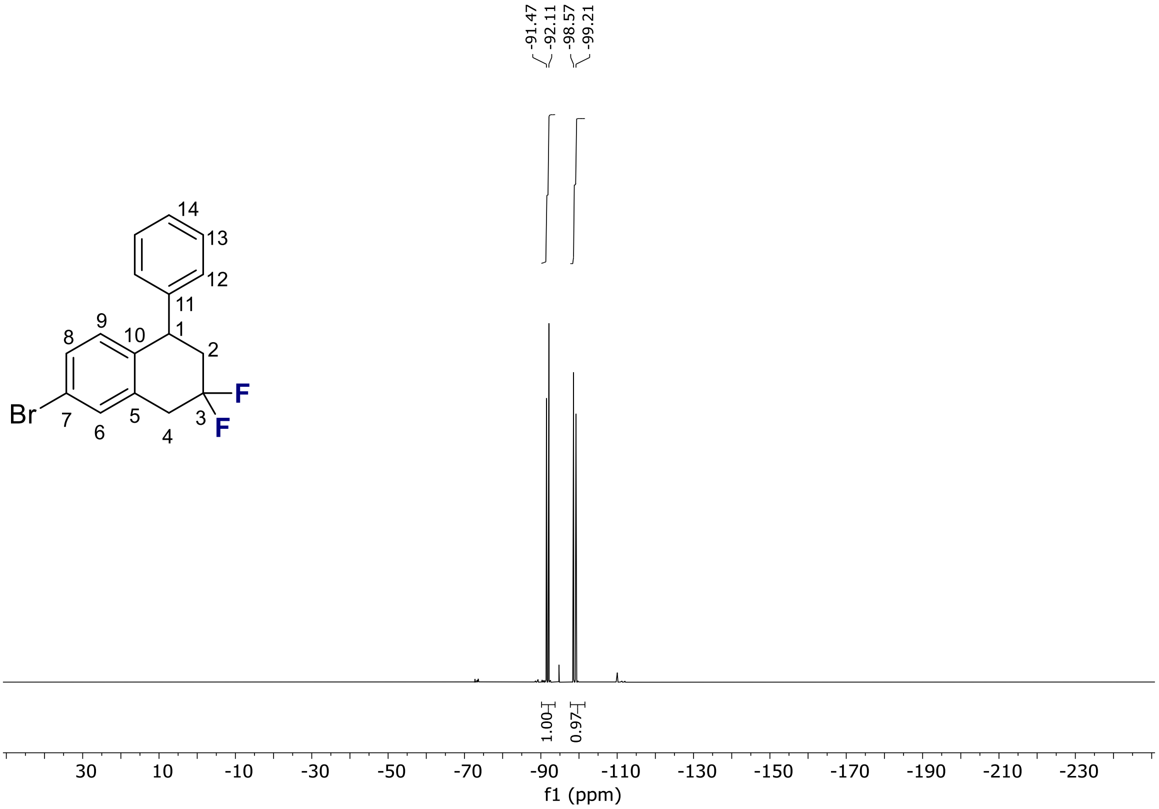


**Supplementary Figure 240.** $^{19}F\{^1H\}$ NMR of **3m** (376 MHz, 299 K, $CDCl_3$).

## 5-Bromo-3,3-difluoro-1-phenyl-1,2,3,4-tetrahydronaphthalene (3n)

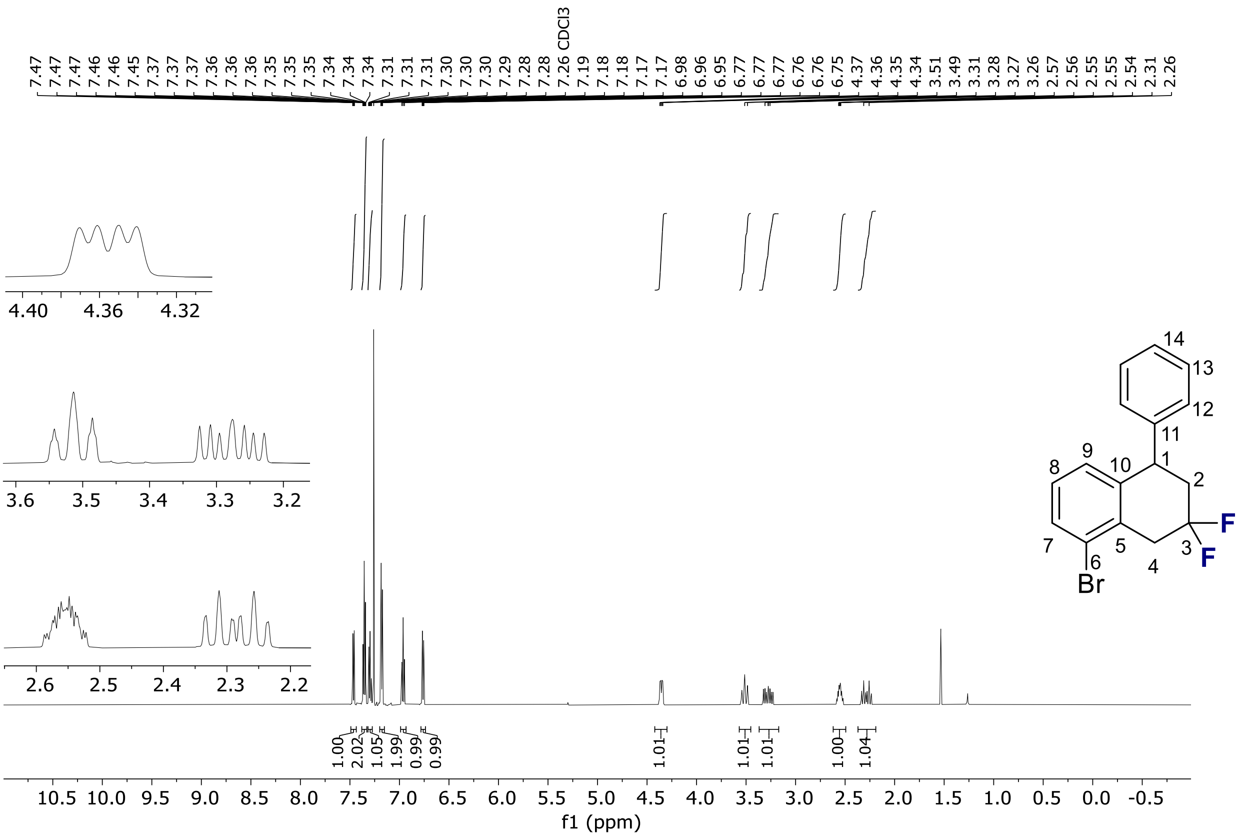


**Supplementary Figure 241.** $^{1}H$ NMR of **3n** (599 MHz, 299 K, $CDCl_3$).

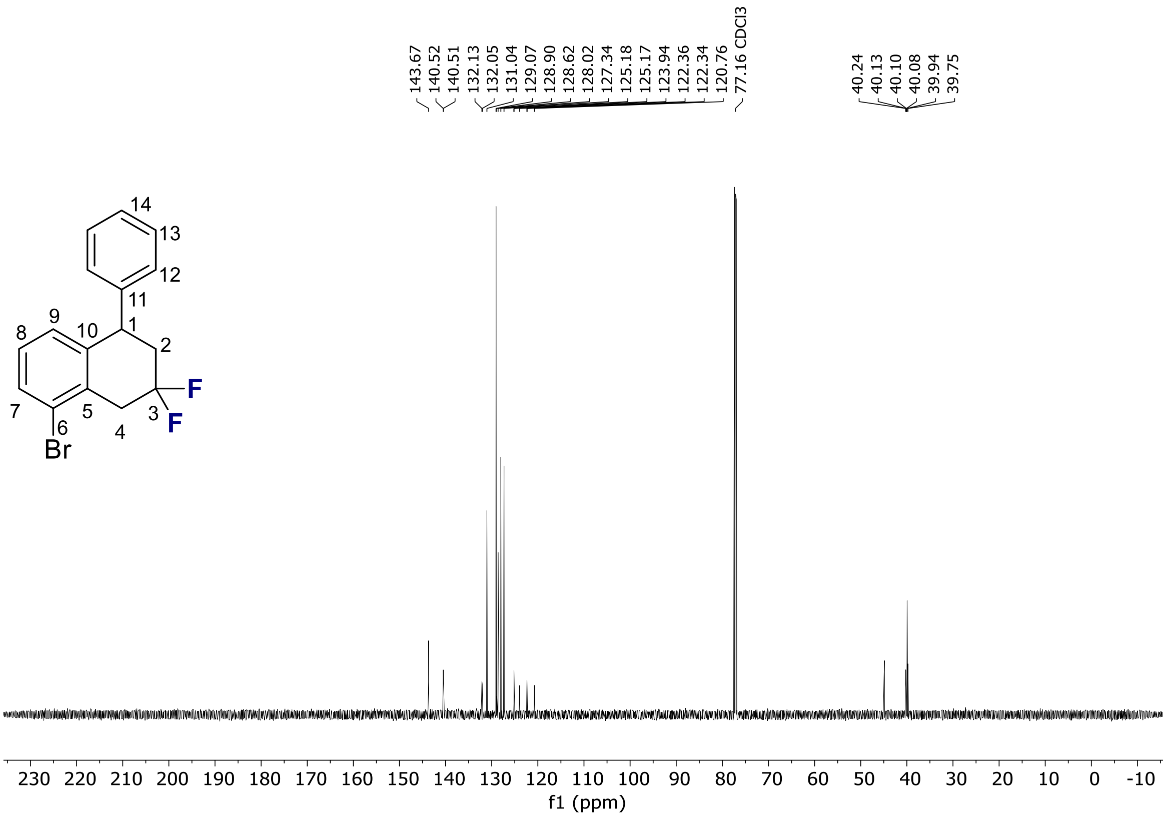


**Supplementary Figure 242.** $^{13}C\{^{1}H\}$ NMR of **3n** (151 MHz, 299 K, $CDCl_3$).

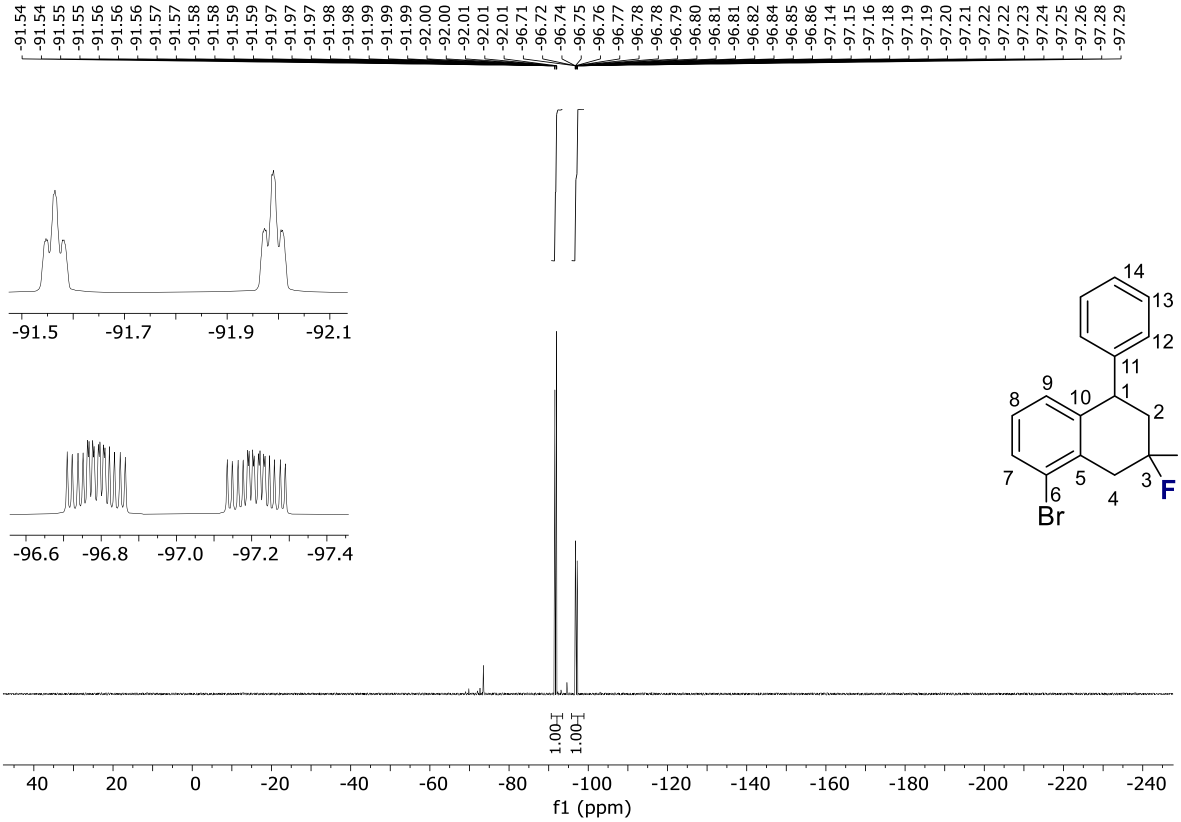


**Supplementary Figure 243.** $^{19}$F NMR of **3n** (564 MHz, 299 K, $CDCl_3$).

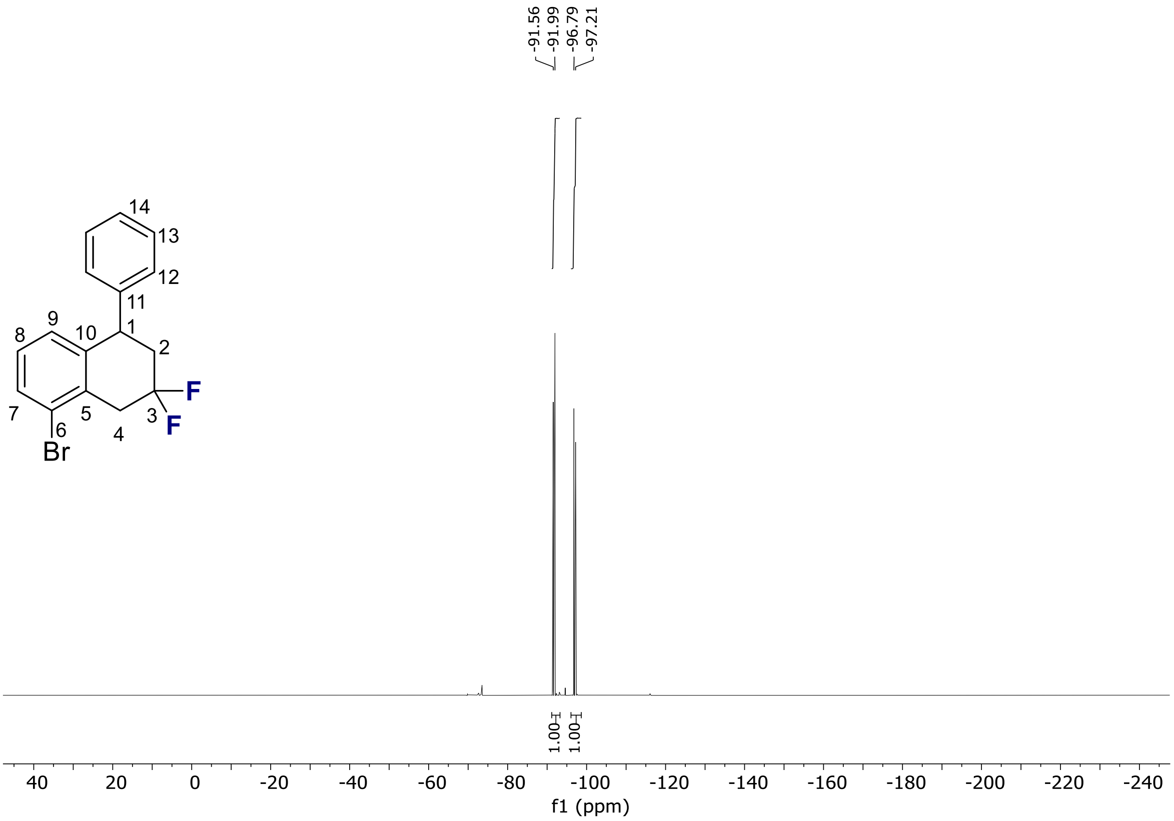


**Supplementary Figure 244.** $^{19}$F{$^{1}$H} NMR of **3n** (564 MHz, 299 K, $CDCl_3$).

**3,3-Difluoro-1-(4-fluorophenyl)-1,2,3,4-tetrahydronaphthalene (3o)**

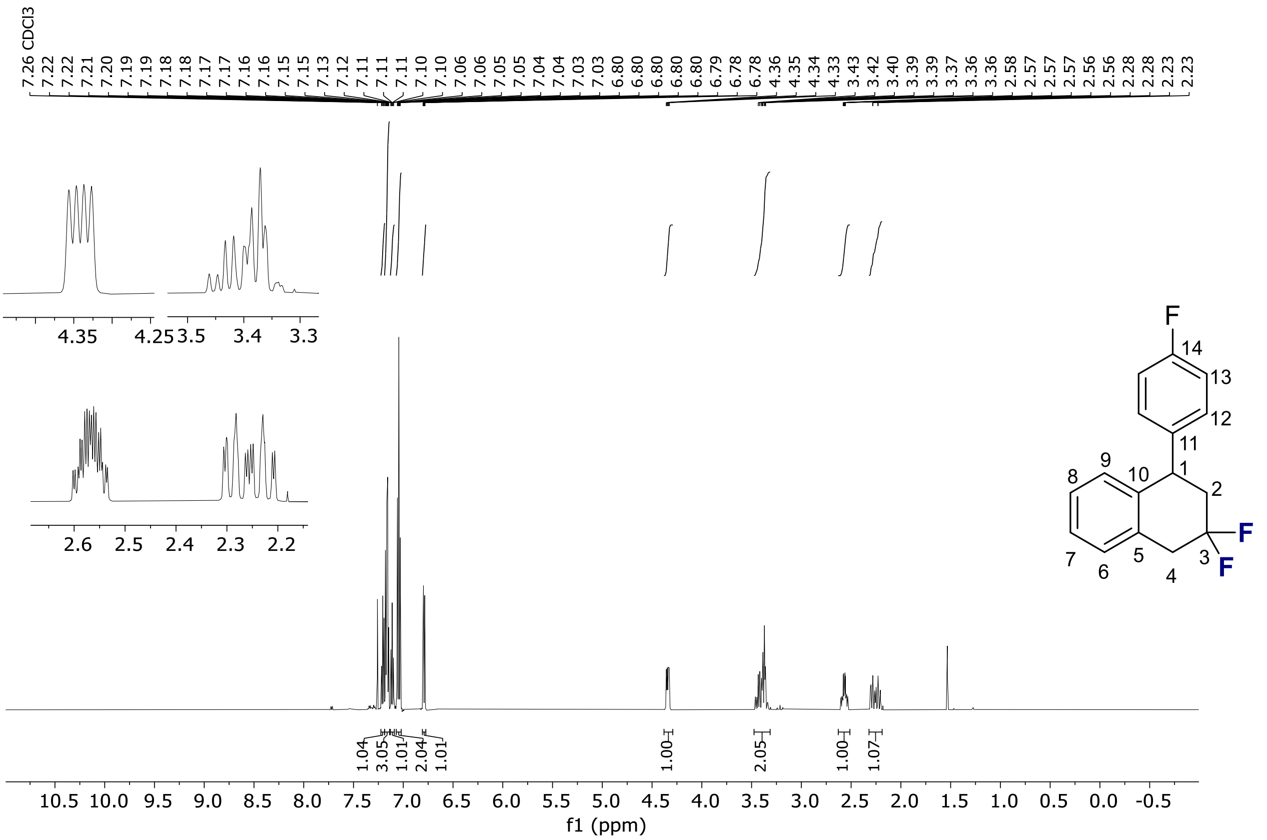


**Supplementary Figure 245.** $^{1}H$ NMR of **3o** (599 MHz, 299 K, $CDCl_3$).

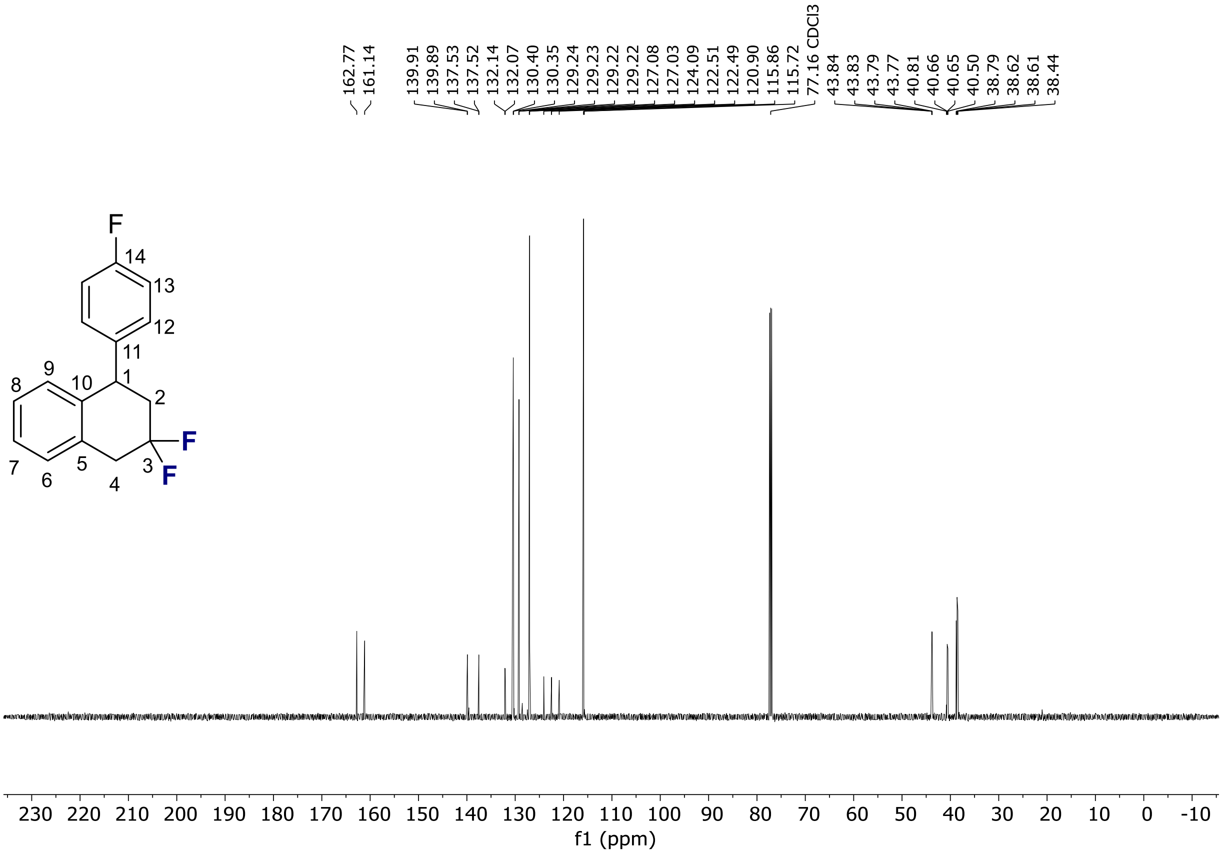


**Supplementary Figure 246.** $^{13}C\{^{1}H\}$ NMR of **3o** (151 MHz, 299 K, $CDCl_3$).

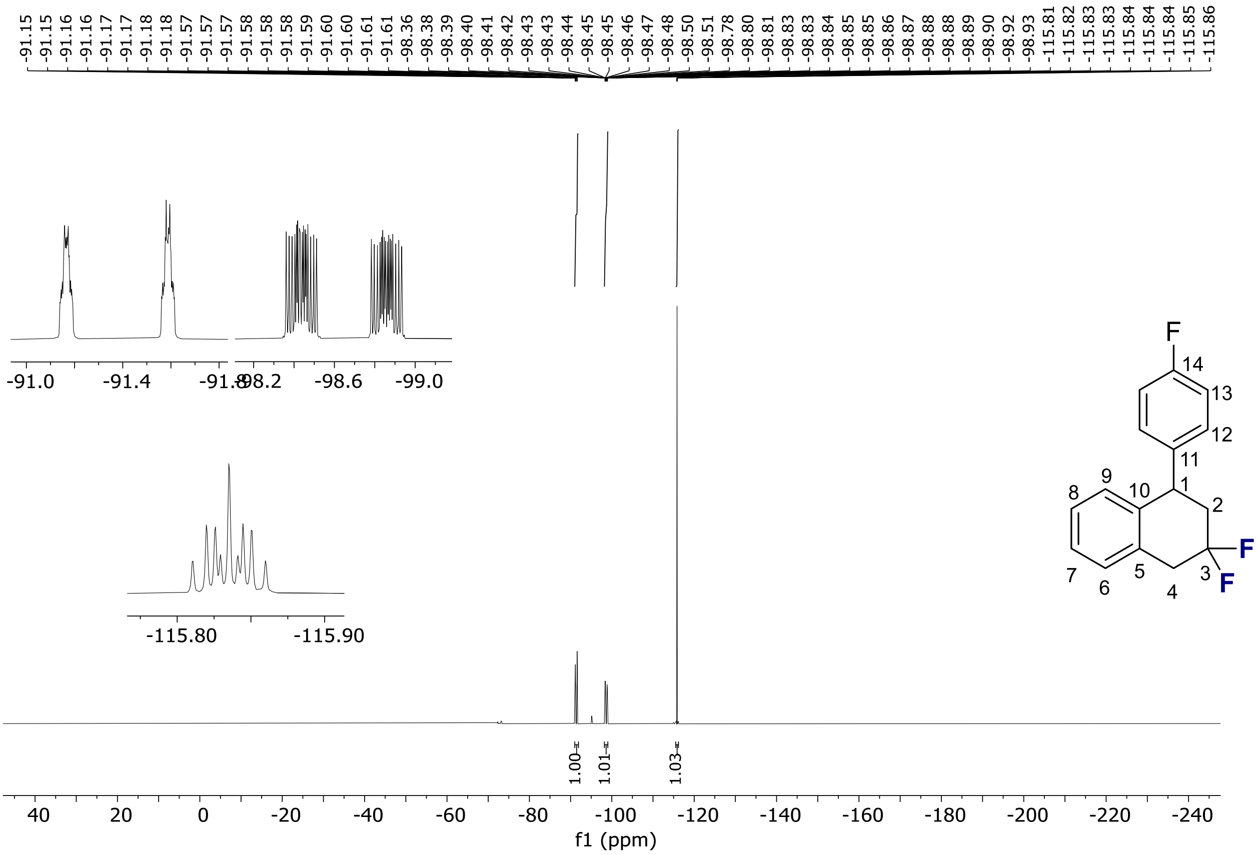


**Supplementary Figure 247.** $^{19}$F NMR of **3o** (564 MHz, 299 K, $CDCl_3$).

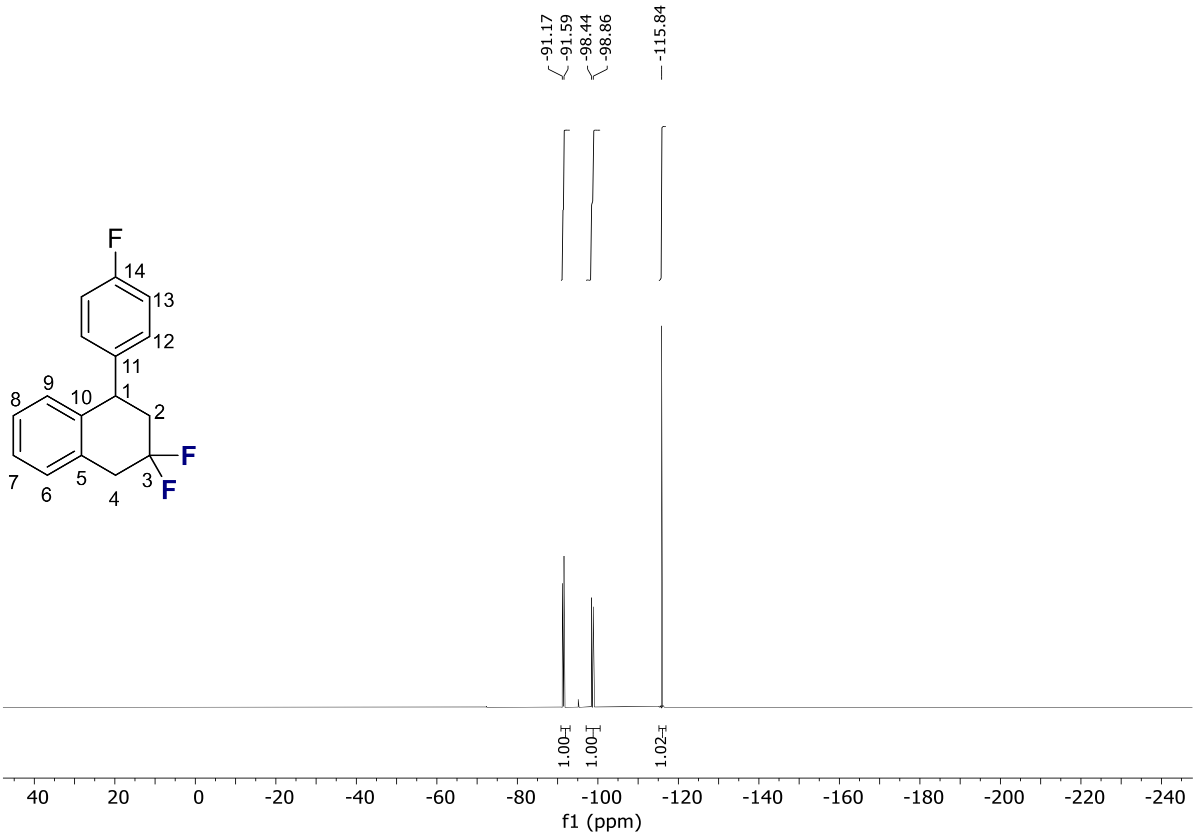


**Supplementary Figure 248.** $^{19}$F{$^{1}$H} NMR of **3o** (564 MHz, 299 K, $CDCl_3$).

## 1-(4-Chlorophenyl)-3,3-difluoro-1,2,3,4-tetrahydronaphthalene (3p)

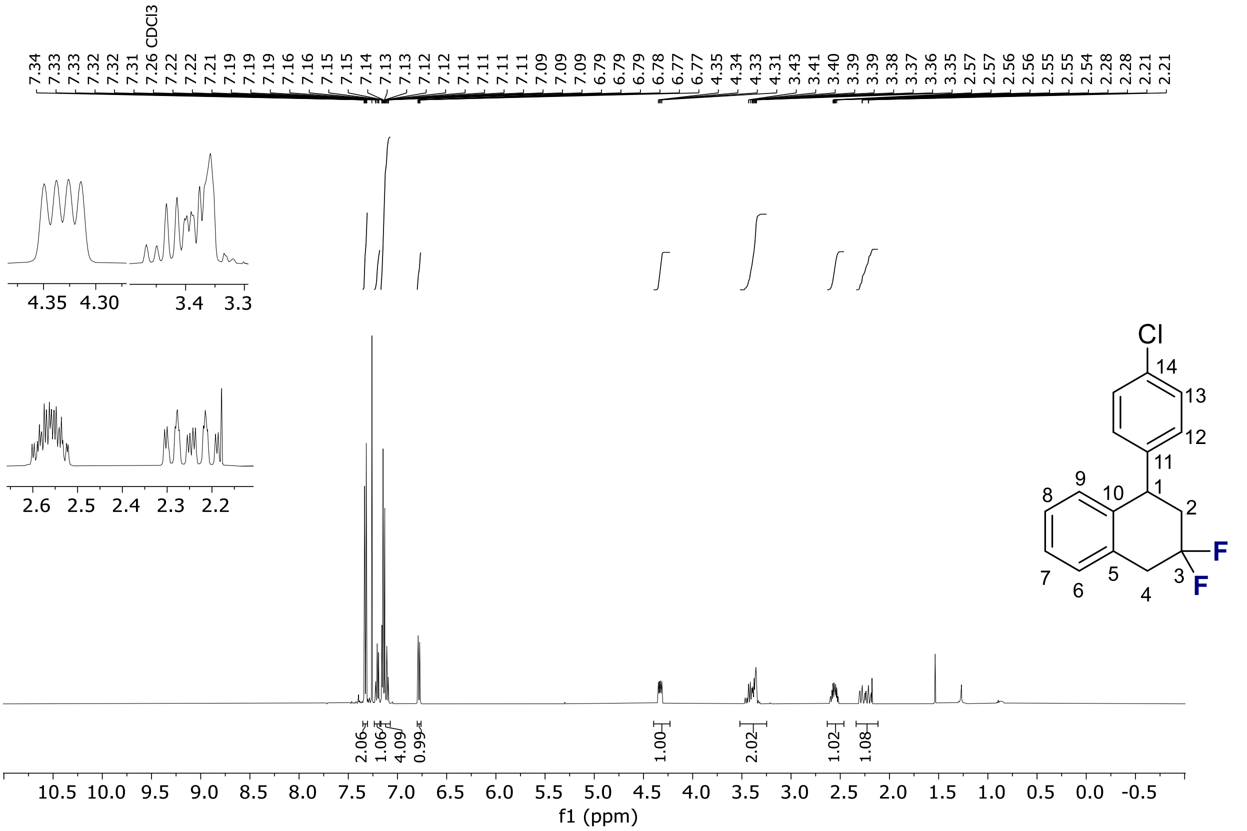


**Supplementary Figure 249.** $^{1}$H NMR of **3p** (500 MHz, 299 K, $CDCl_3$).

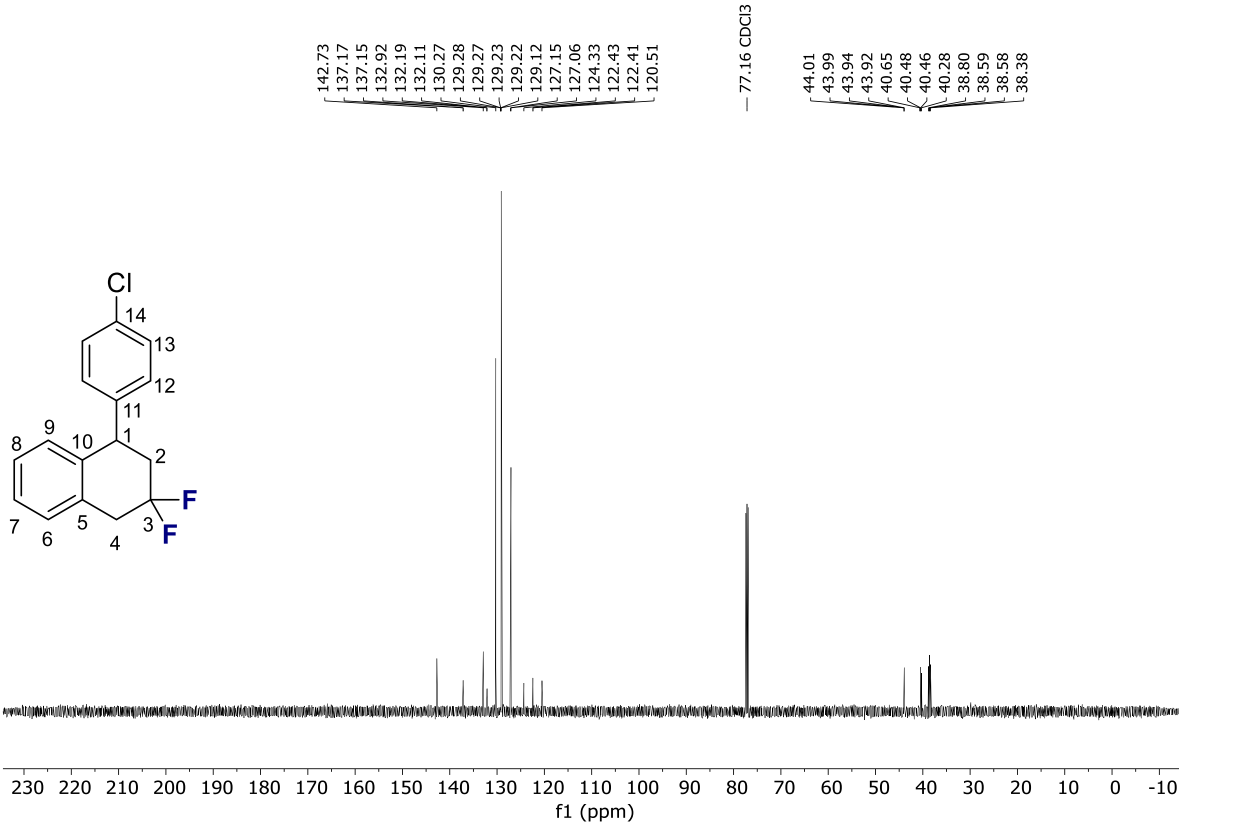


**Supplementary Figure 250.** $^{13}C\{^{1}H\}$ NMR of **3p** (126 MHz, 299 K, $CDCl_3$).

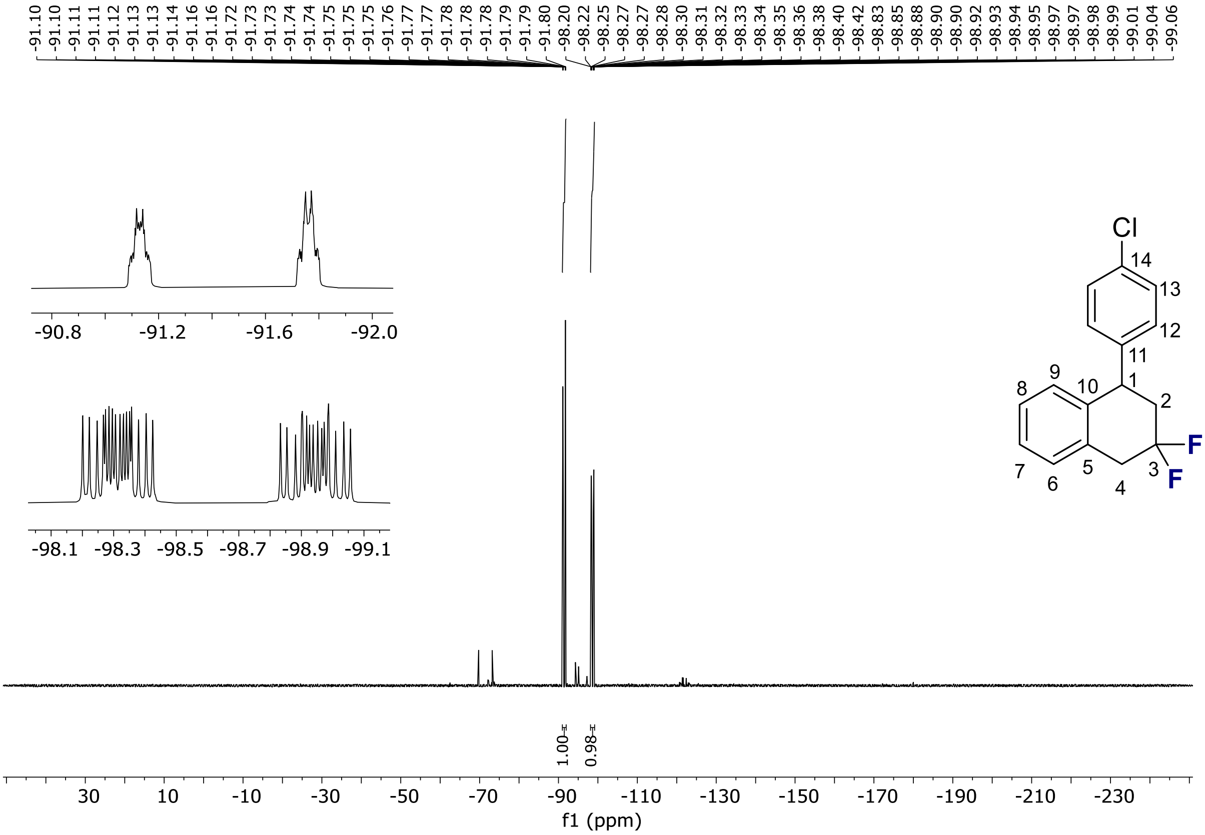


**Supplementary Figure 251.** $^{19}$F NMR of **3p** (376 MHz, 299 K, $CDCl_3$).

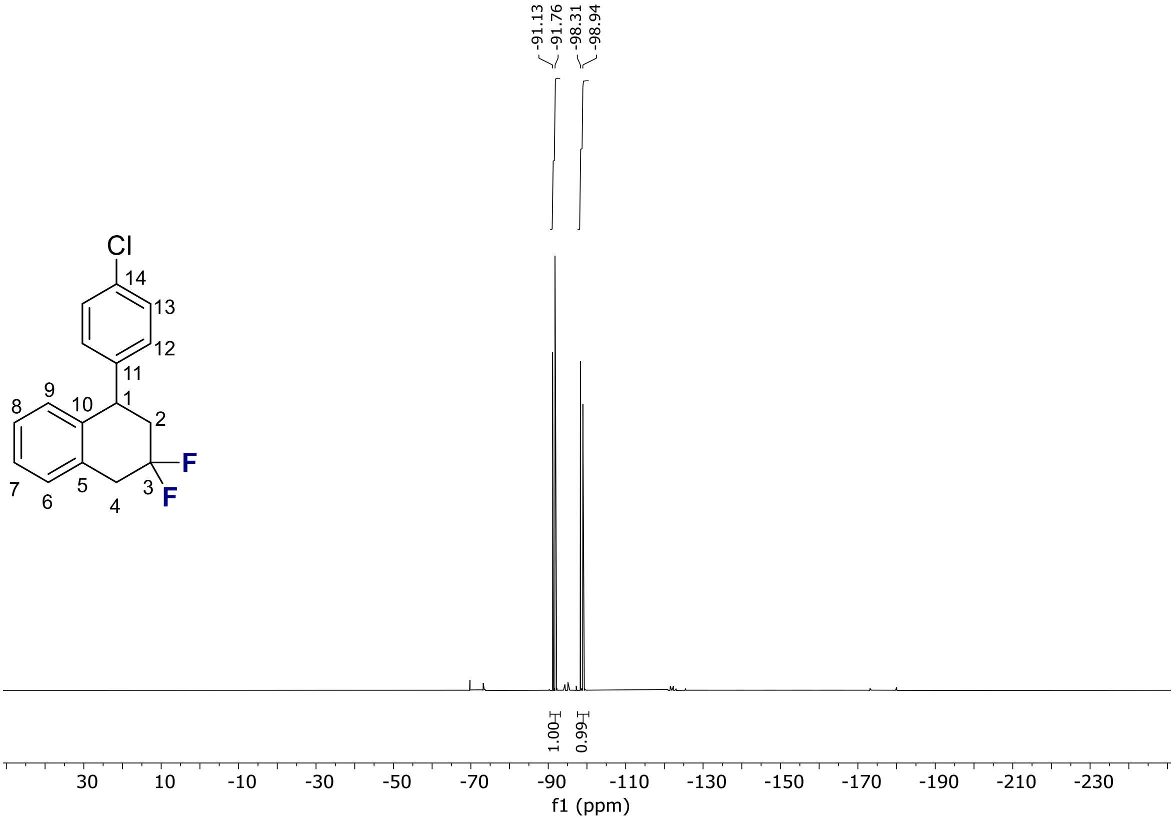


**Supplementary Figure 252.** $^{19}$F{$^1$H} NMR of **3p** (376 MHz, 299 K, $CDCl_3$).

## 1-(4-Bromophenyl)-3,3-difluoro-1,2,3,4-tetrahydronaphthalene (3q)

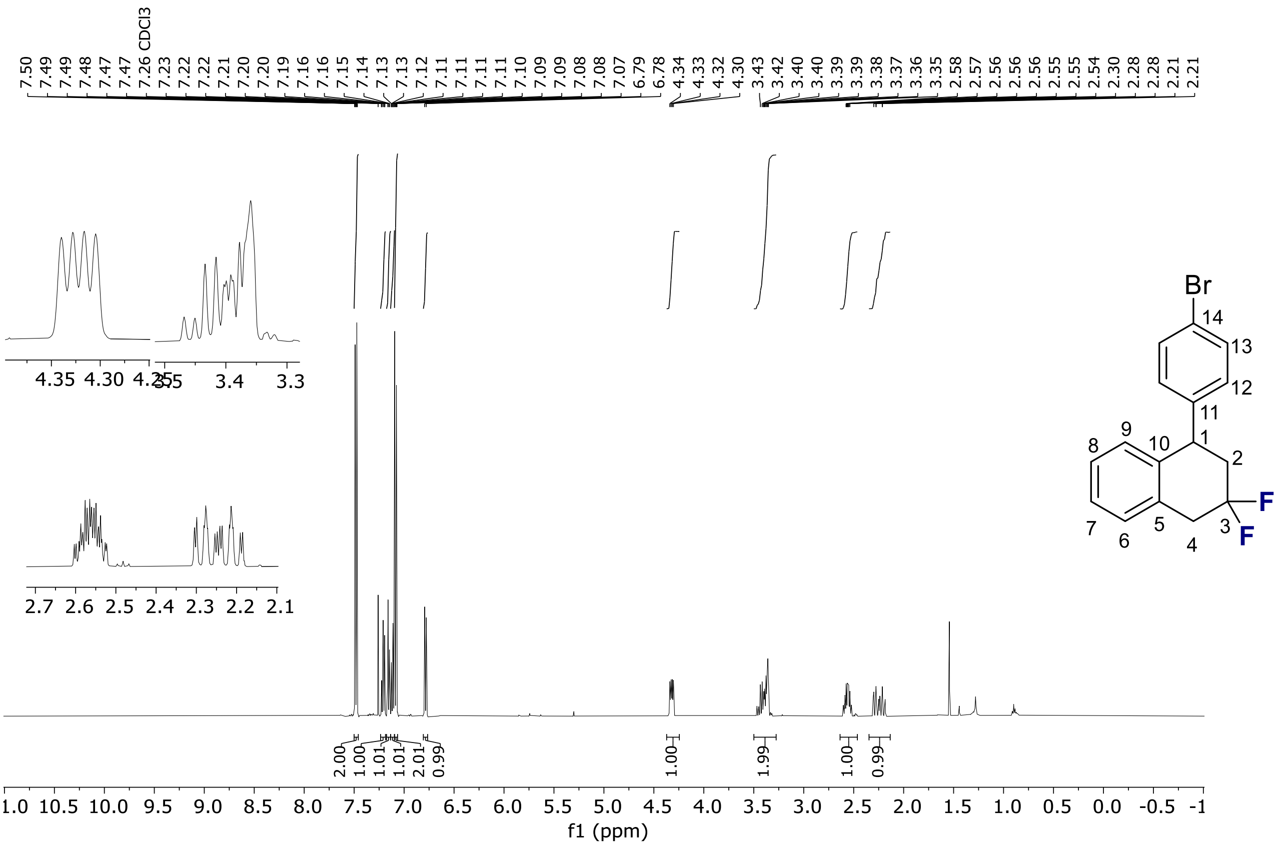


**Supplementary Figure 253.** $^{1}H$ NMR of **3q** (500 MHz, 299 K, $CDCl_3$).

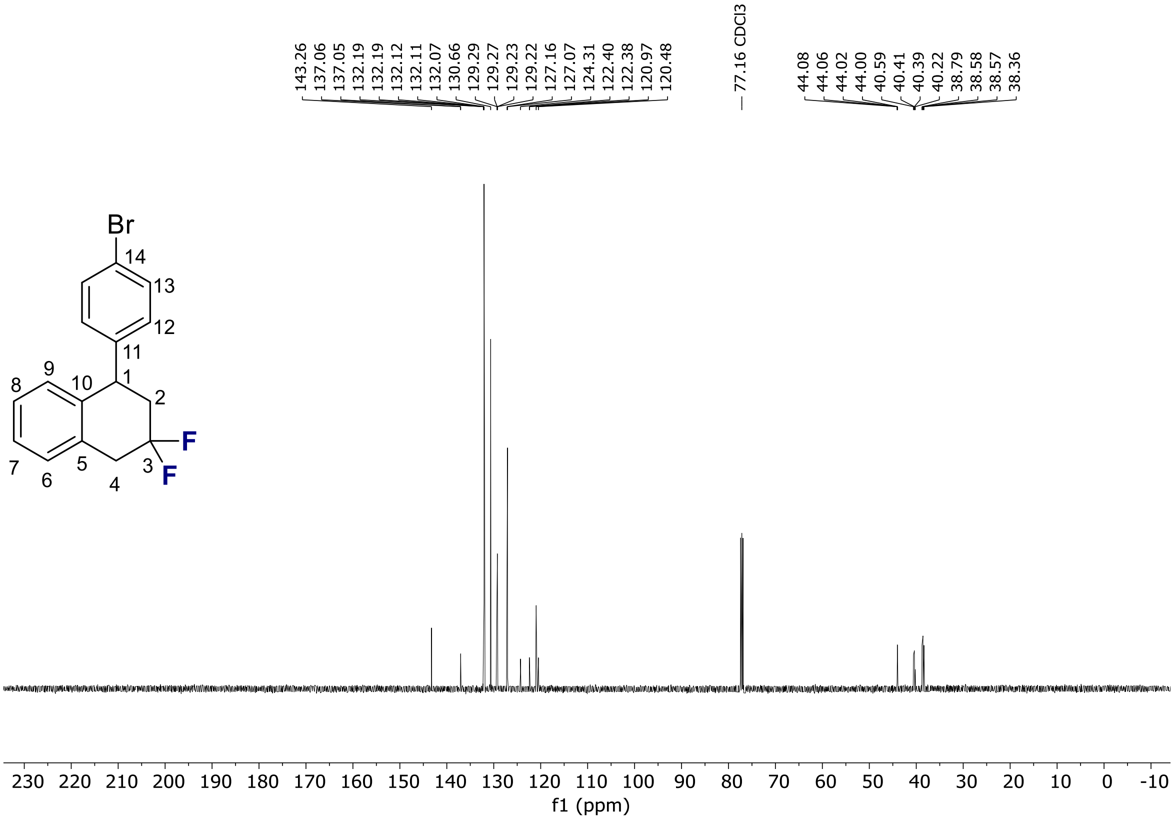


**Supplementary Figure 254.** $^{13}C\{^{1}H\}$ NMR of **3q** (126 MHz, 299 K, $CDCl_3$).

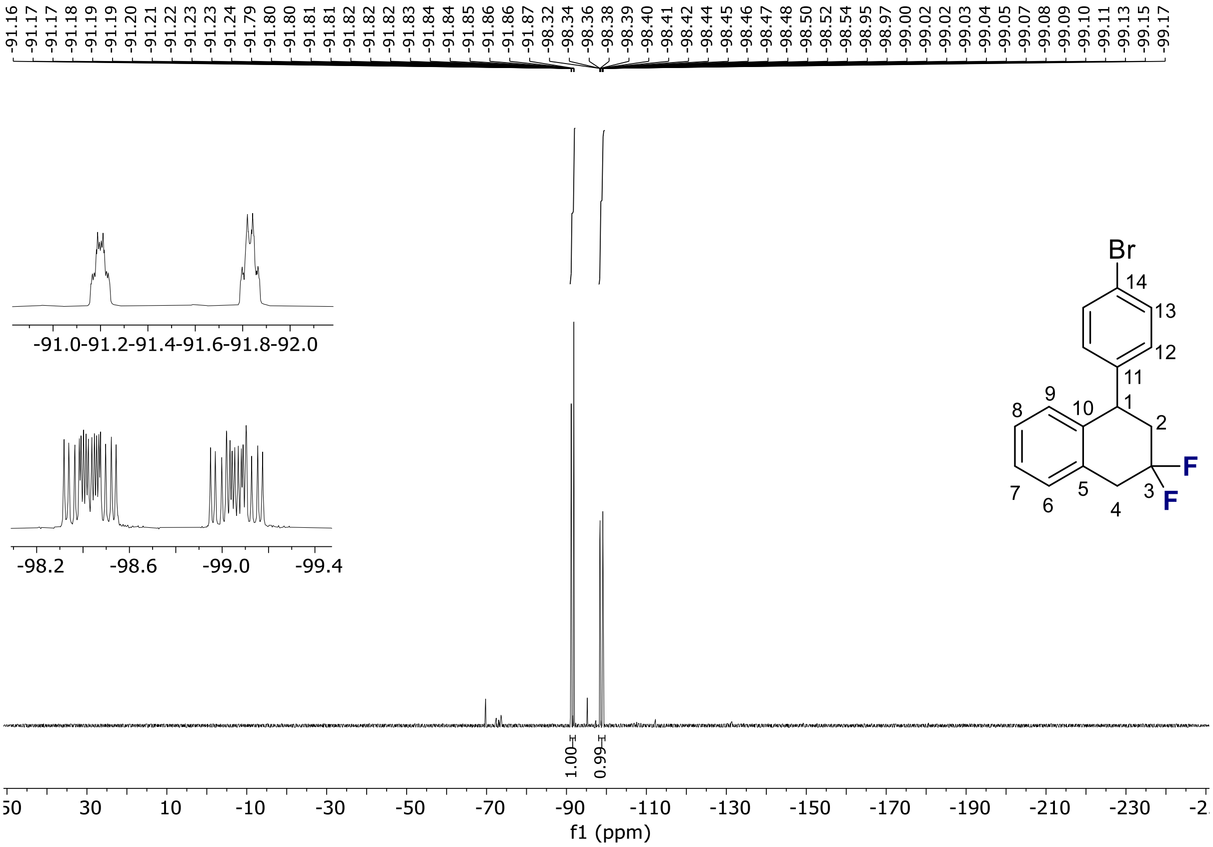


**Supplementary Figure 255.** $^{19}$F NMR of **3q** (376 MHz, 299 K, $CDCl_3$).

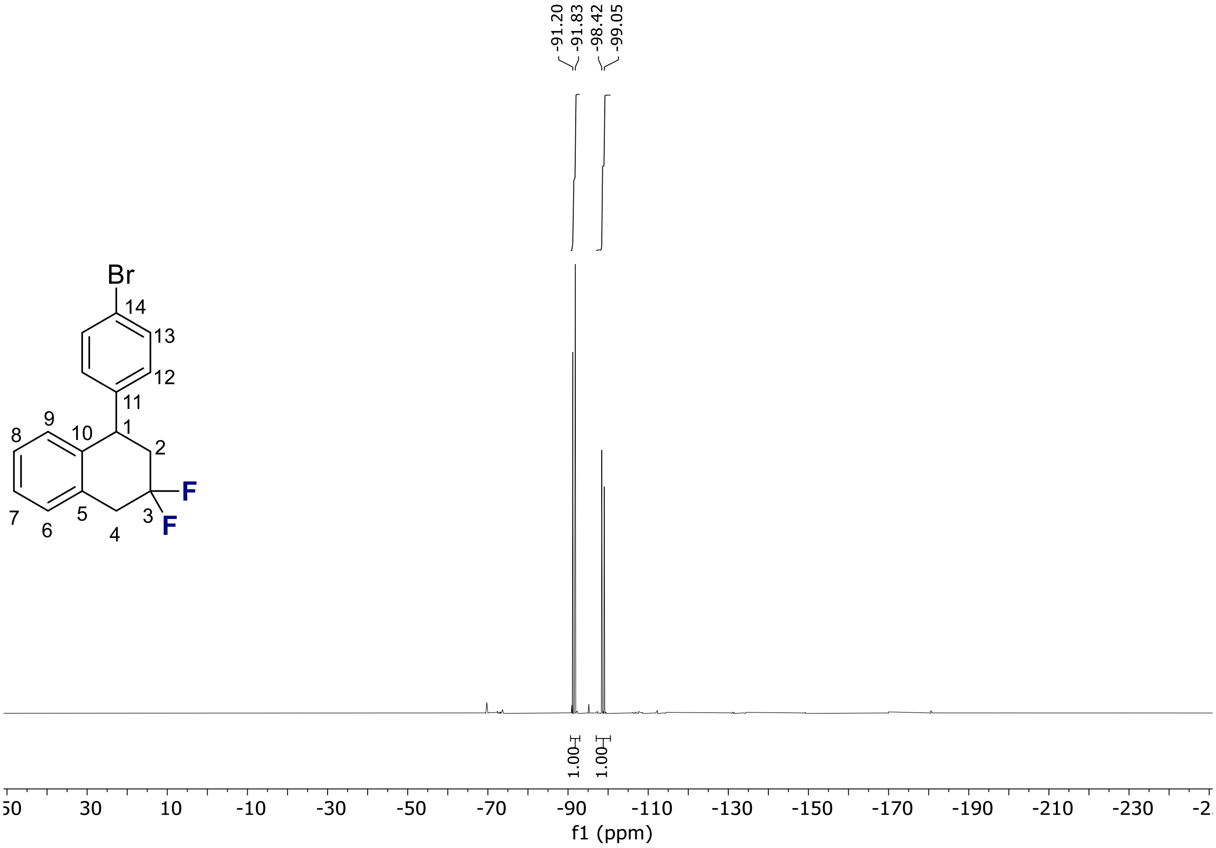


**Supplementary Figure 256.** $^{19}$F{$^1$H} NMR of **3q** (376 MHz, 299 K, $CDCl_3$).

## 3,3-Difluoro-1-(4-(trifluoromethyl)phenyl)-1,2,3,4-tetrahydronaphthalene (3r)

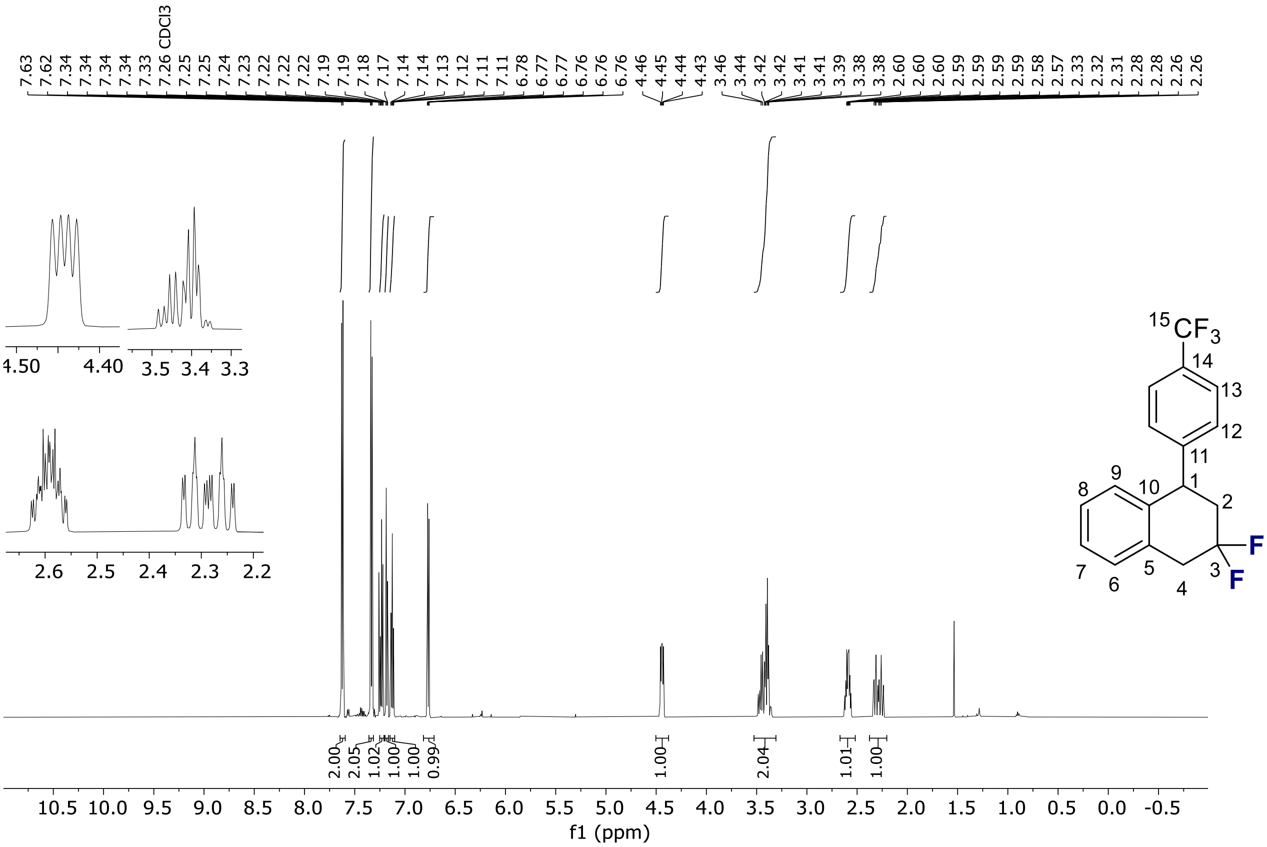


**Supplementary Figure 257.** $^{1}H$ NMR of **3r** (599 MHz, 299 K, $CDCl_3$).

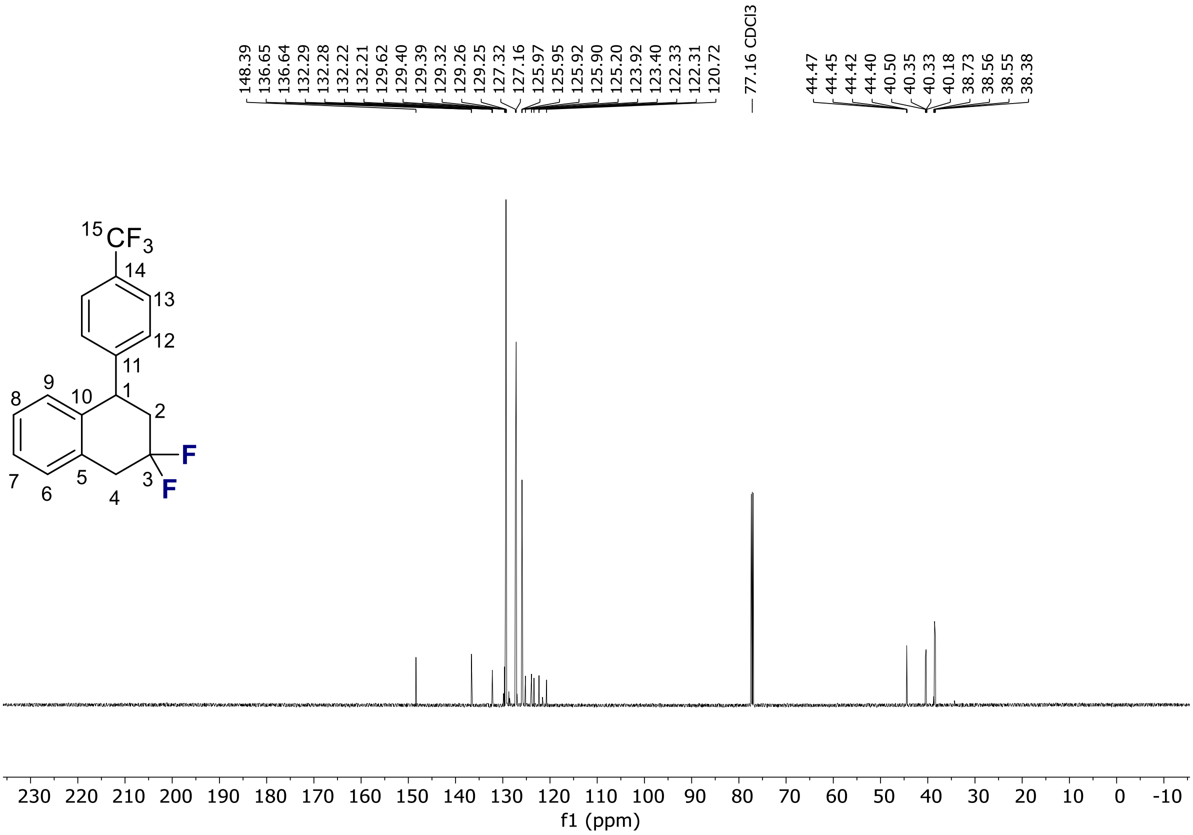


**Supplementary Figure 258.** $^{13}C\{^{1}H\}$ NMR of **3r** (151 MHz, 299 K, $CDCl_3$).

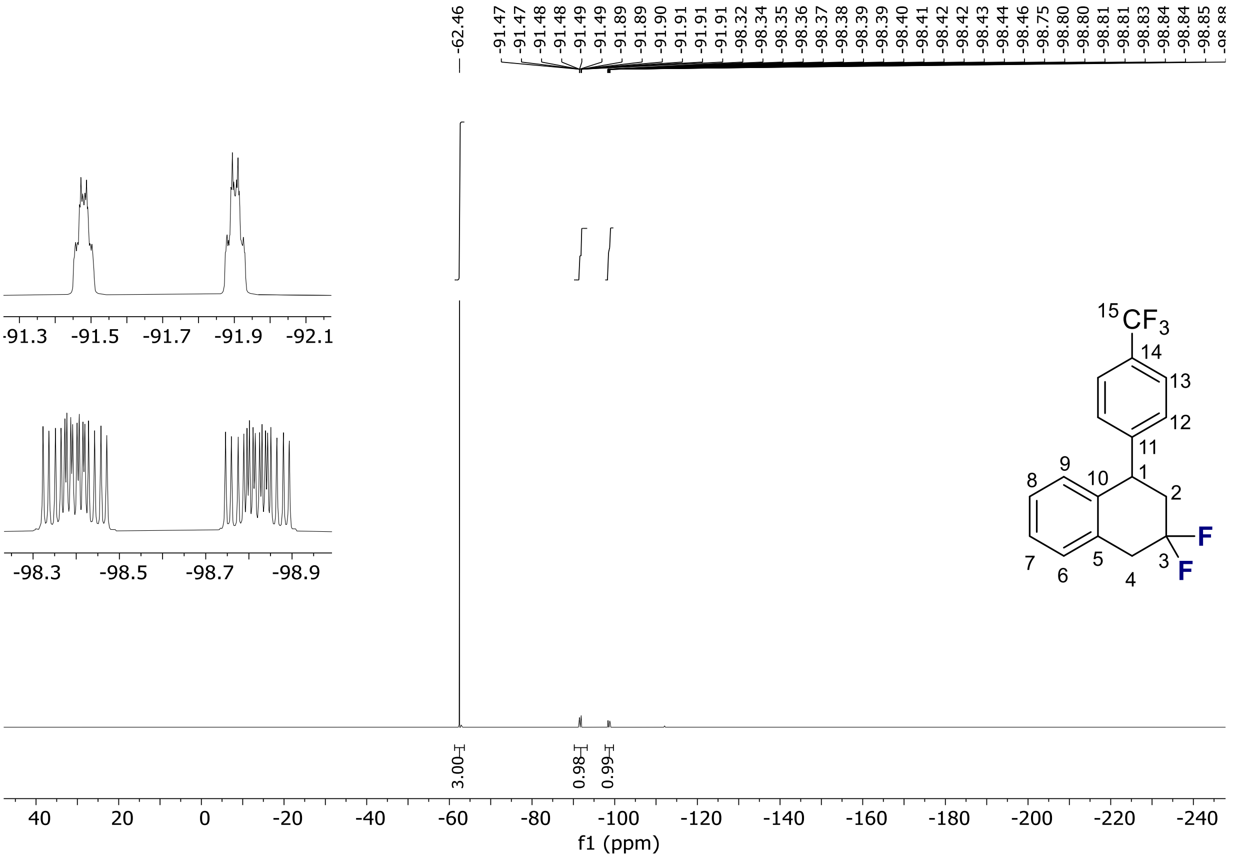


**Supplementary Figure 259.** $^{19}$F NMR of **3r** (564 MHz, 299 K, $CDCl_3$).

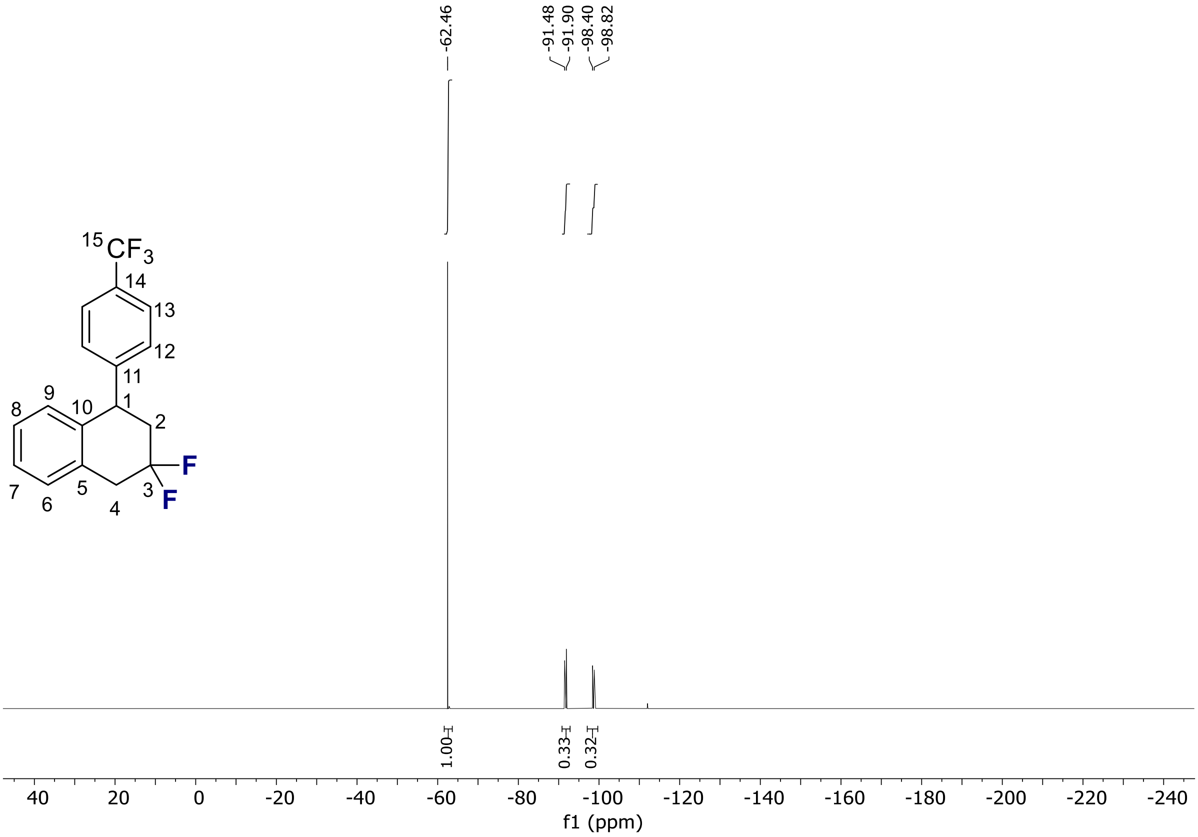


**Supplementary Figure 260.** $^{19}$F{$^1$H} NMR of **3r** (564 MHz, 299 K, $CDCl_3$).

## 4-(3,3-Difluoro-1,2,3,4-tetrahydronaphthalen-1-yl)phenyl trifluoromethane-sulfonate (3s)

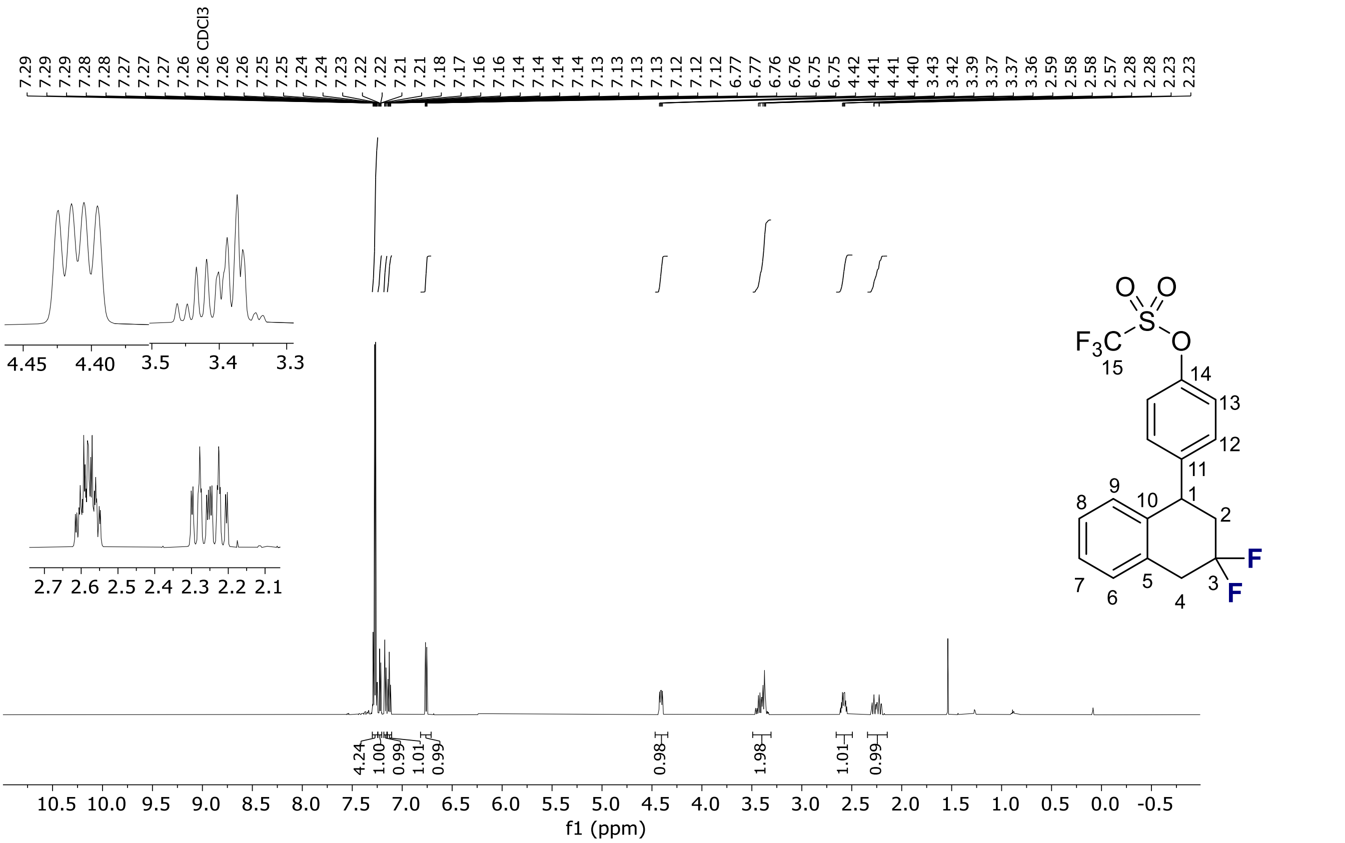


**Supplementary Figure 261.** $^{1}$H NMR of **3s** (599 MHz, 299 K, $CDCl_3$).

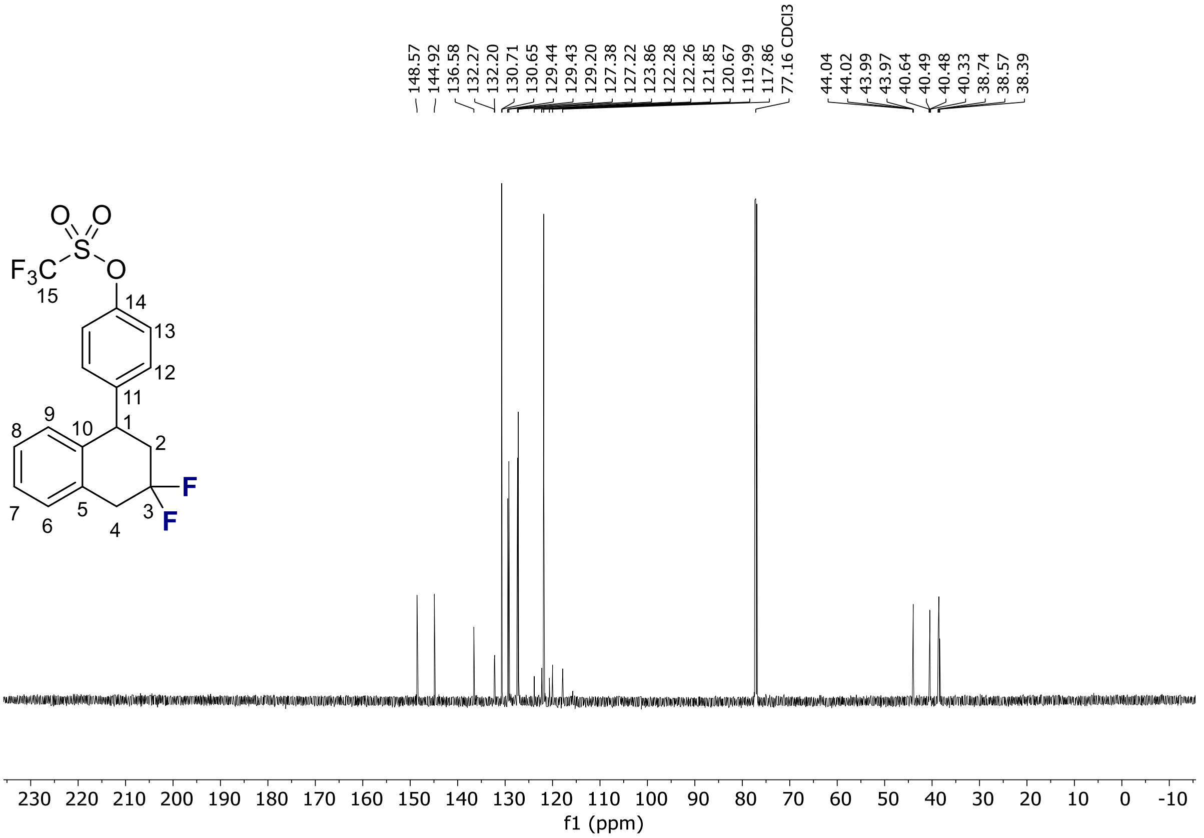


**Supplementary Figure 262.** $^{13}C\{^{1}H\}$ NMR of **3s** (151 MHz, 299 K, $CDCl_3$).

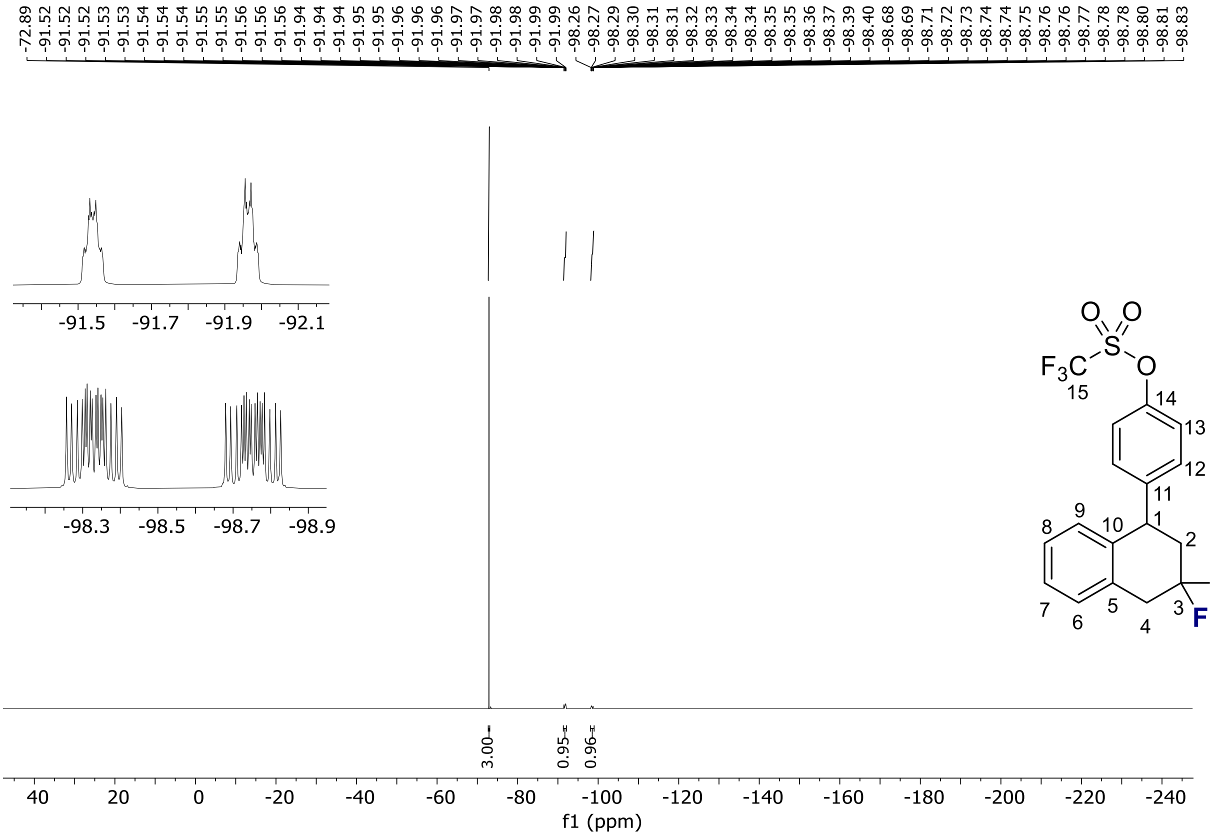


**Supplementary Figure 263.** $^{19}F$ NMR of **3s** (564 MHz, 299 K, $CDCl_3$).

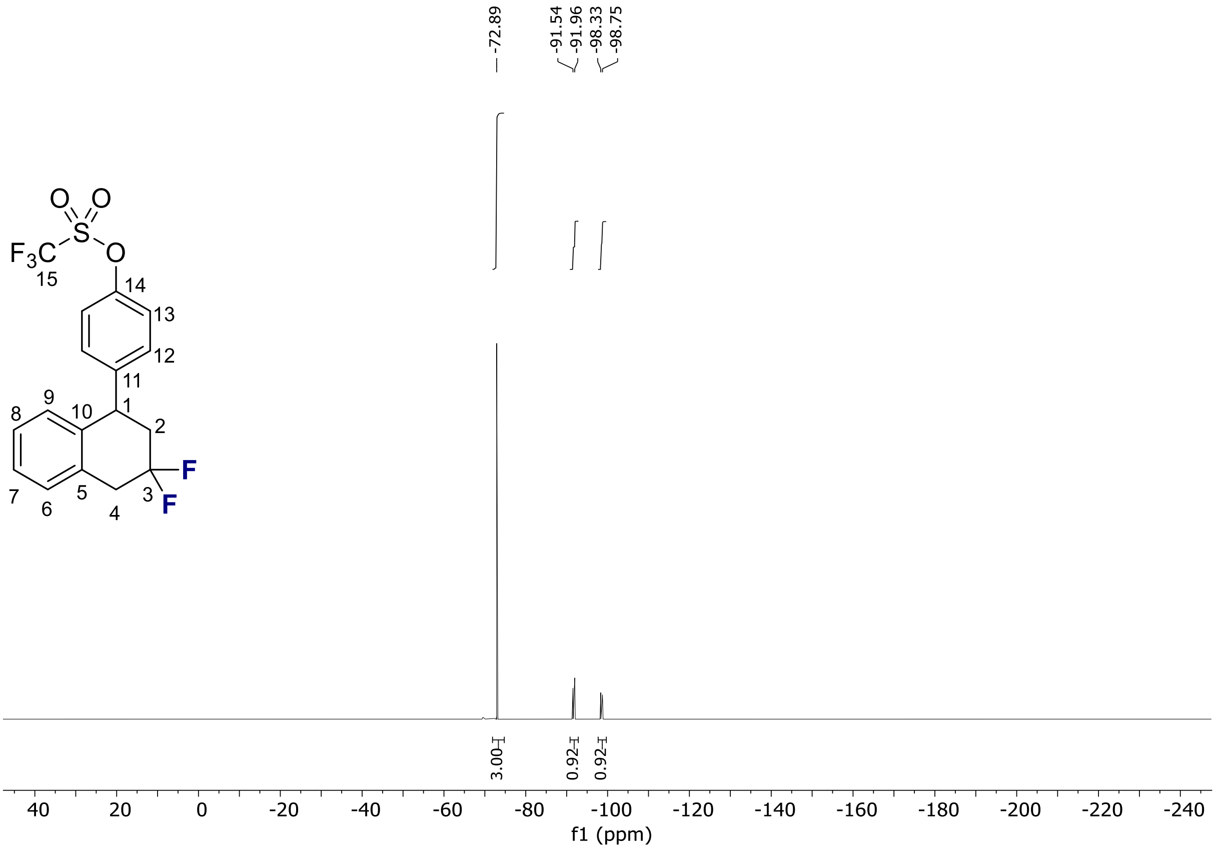


**Supplementary Figure 264.** $^{19}F\{^1H\}$ NMR of **3s** (564 MHz, 299 K, $CDCl_3$).

## 7-Bromo-1-(4-fluorophenyl)-3,3-difluoro-1,2,3,4-tetrahydronaphthalene (3t)

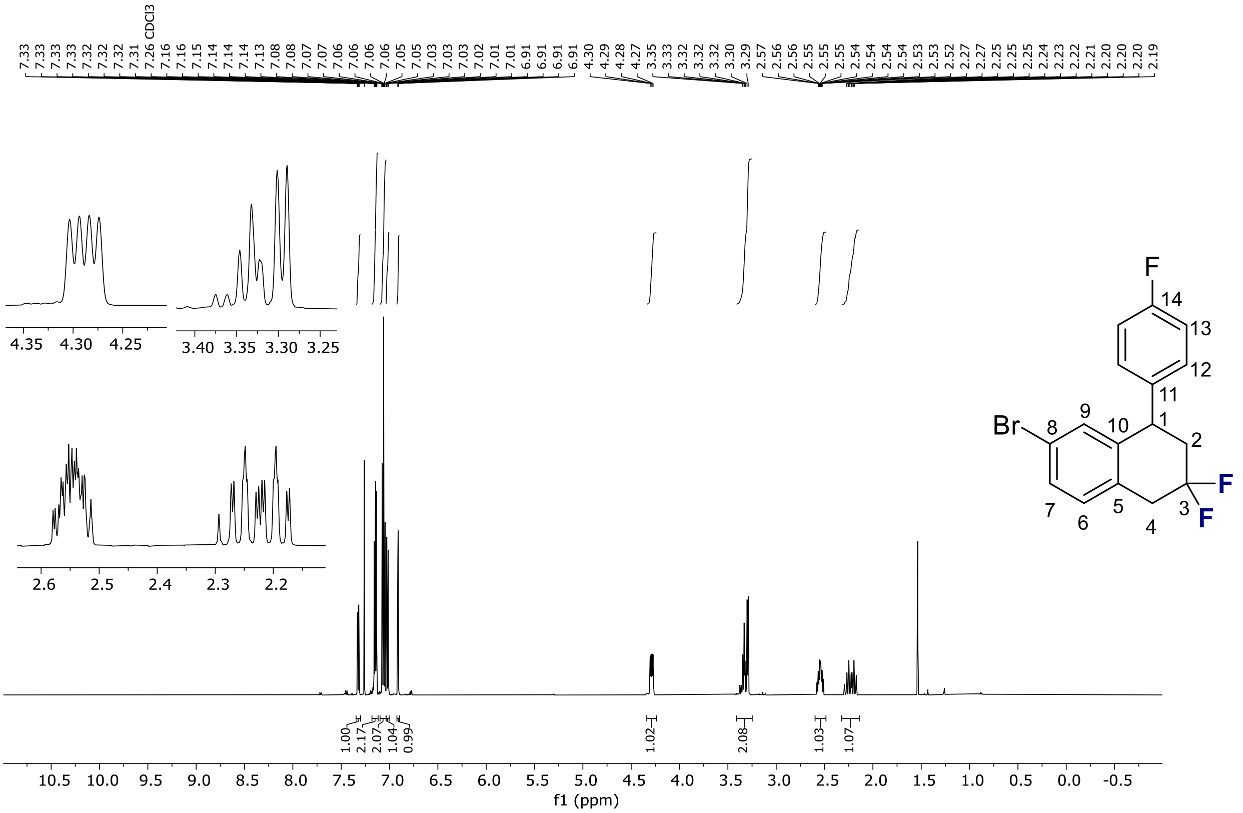


**Supplementary Figure 265.** $^{1}H$ NMR of **3t** (599 MHz, 299 K, $CDCl_3$).

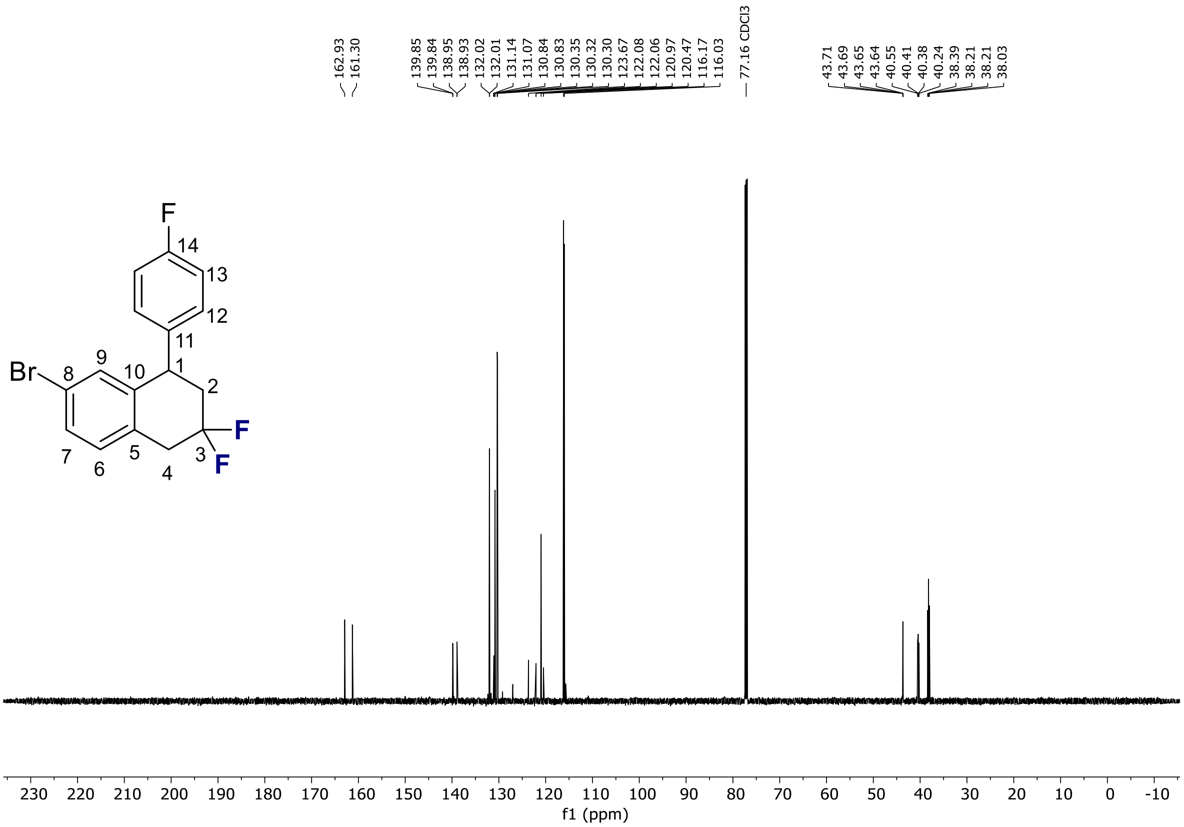


**Supplementary Figure 266.** $^{13}C\{^{1}H\}$ NMR of **3t** (151 MHz, 299 K, $CDCl_3$).

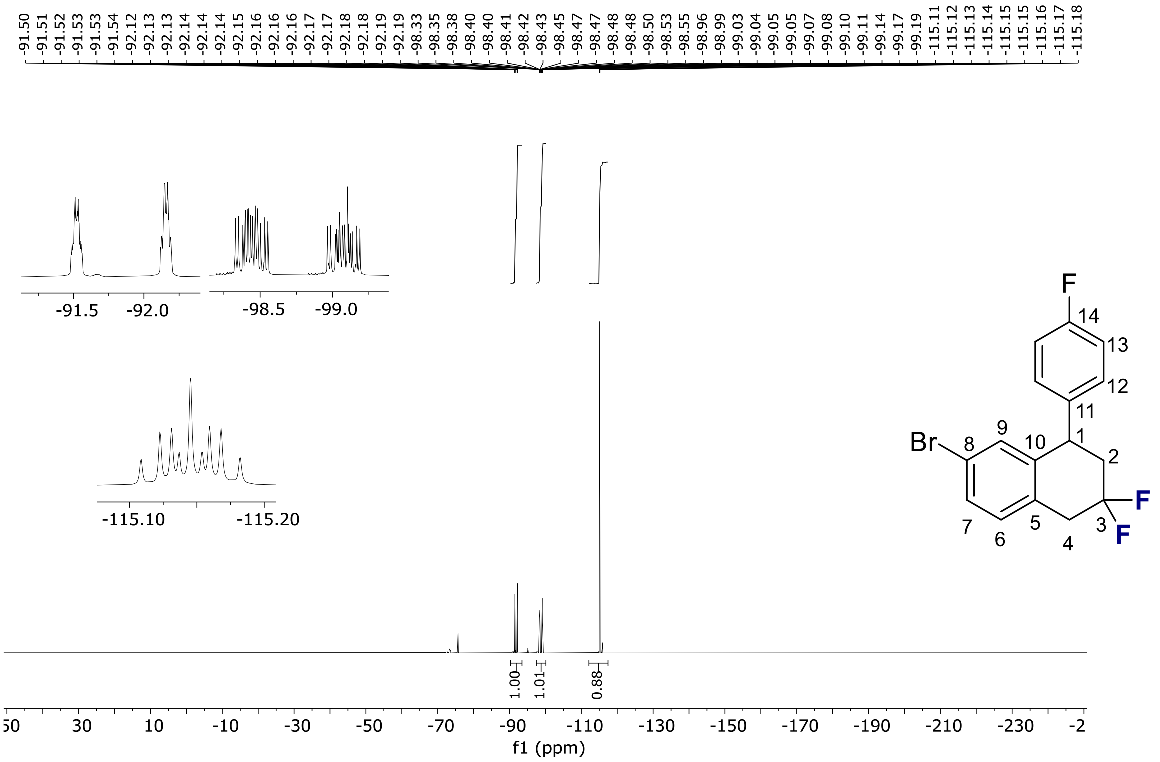


**Supplementary Figure 267.** $^{19}$F NMR of **3t** (376 MHz, 299 K, $CDCl_3$).

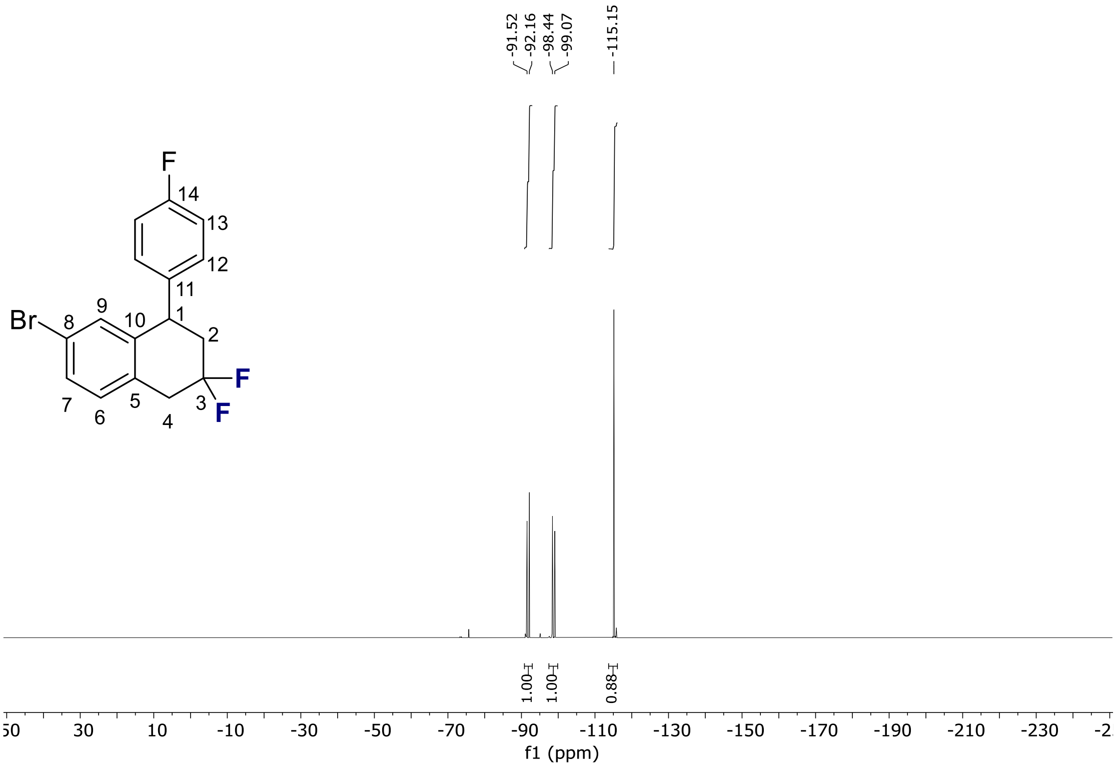


**Supplementary Figure 268.** $^{19}$F{$^1$H} NMR of **3t** (376 MHz, 299 K, $CDCl_3$).

## 7-Bromo-1-(4-chlorophenyl)-3,3-difluoro-1,2,3,4-tetrahydronaphthalene (3u)

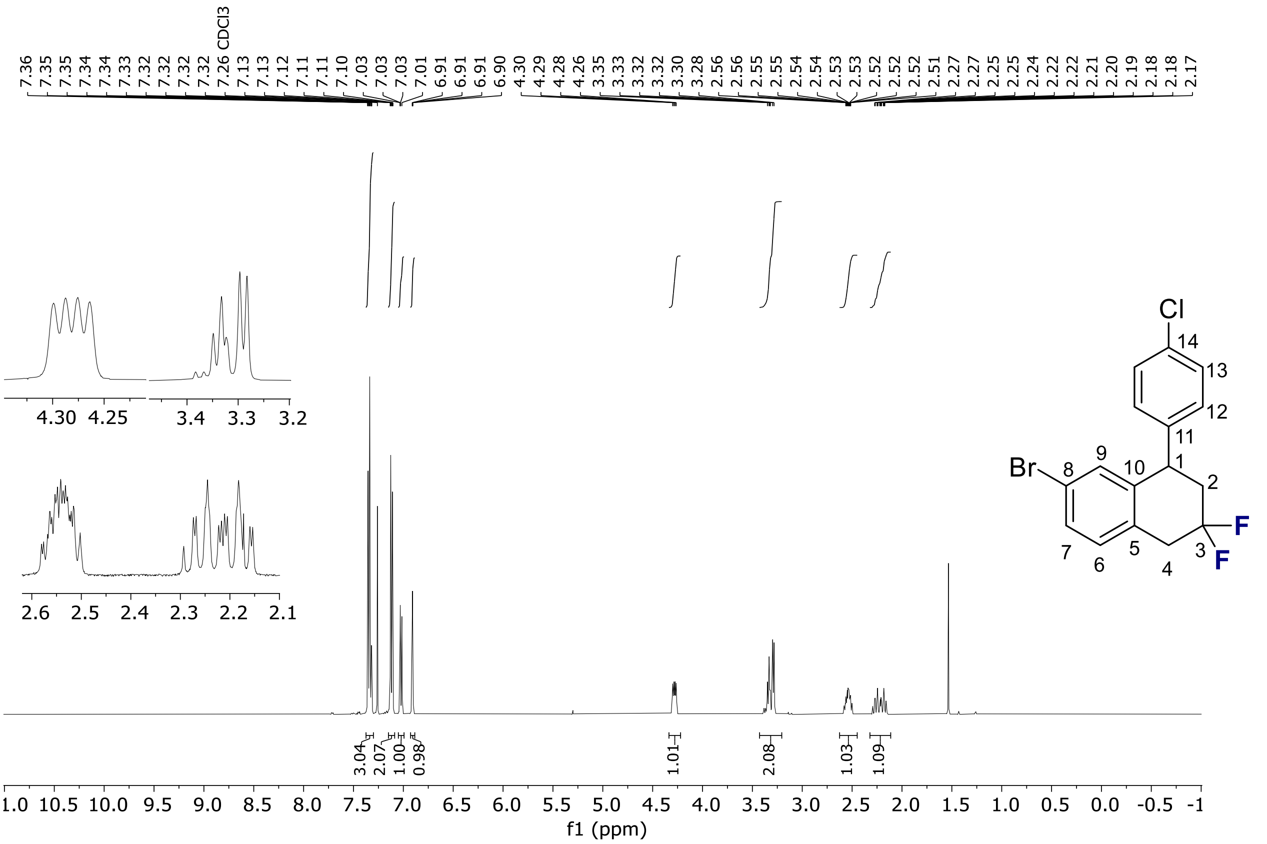


**Supplementary Figure 269.** $^{1}$H NMR of **3u** (500 MHz, 299 K, $CDCl_3$).

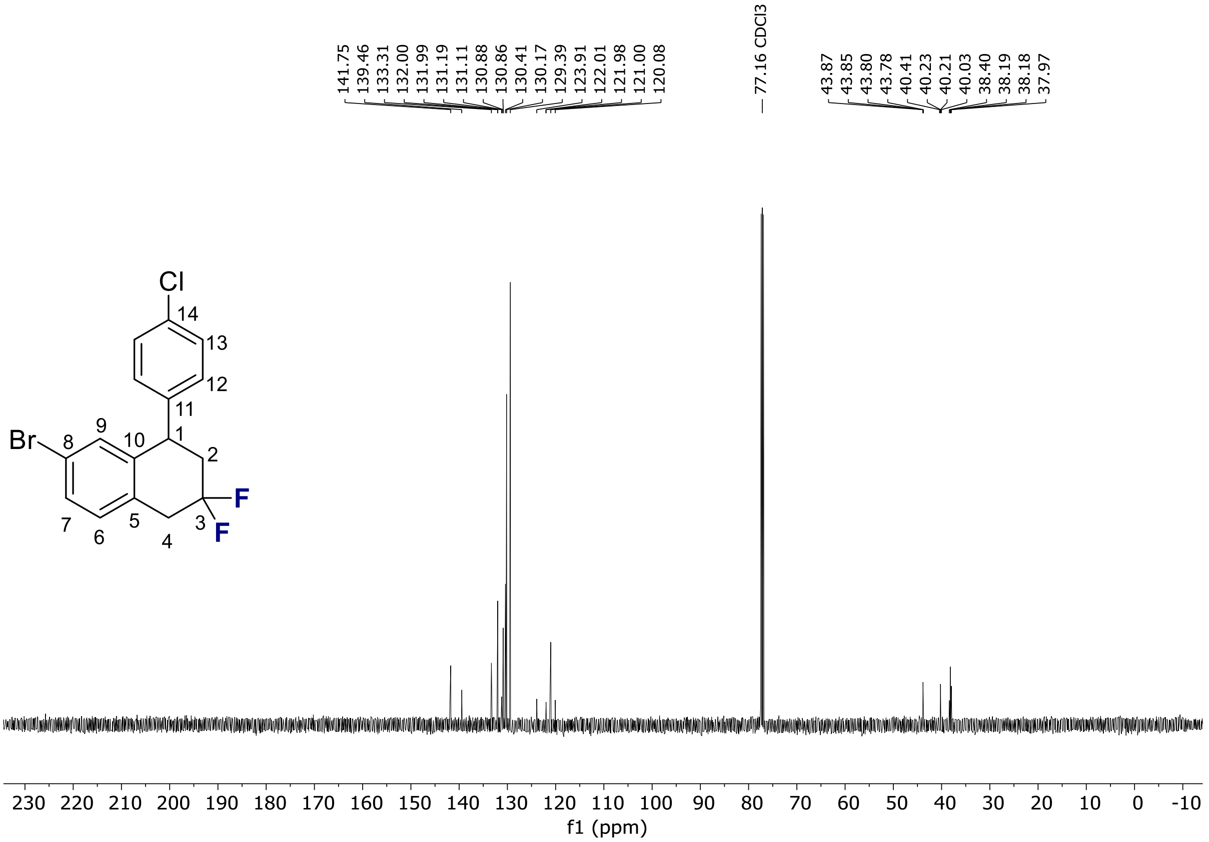


**Supplementary Figure 270.** $^{13}$C{$^{1}$H} NMR of **3u** (126 MHz, 299 K, $CDCl_3$).

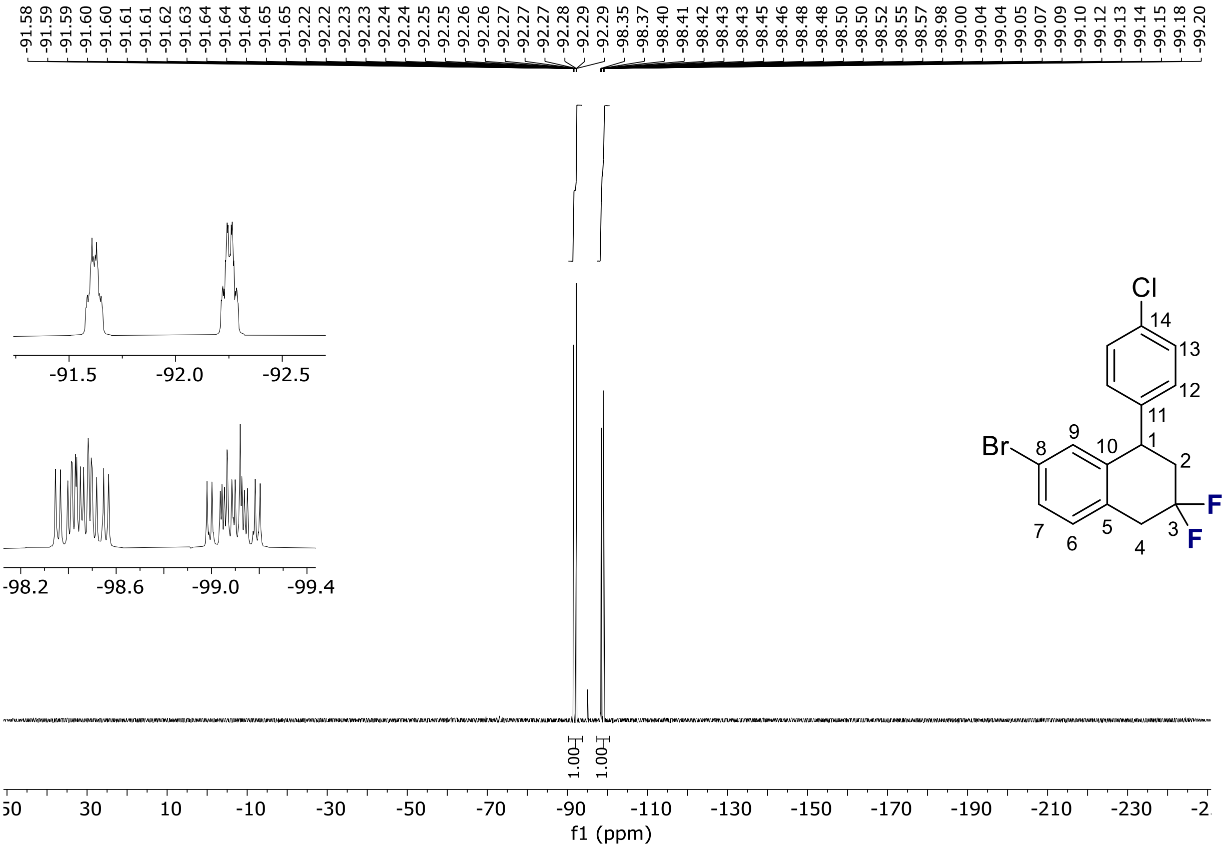


**Supplementary Figure 271.** $^{19}F$ NMR of **3u** (376 MHz, 299 K, $CDCl_3$).

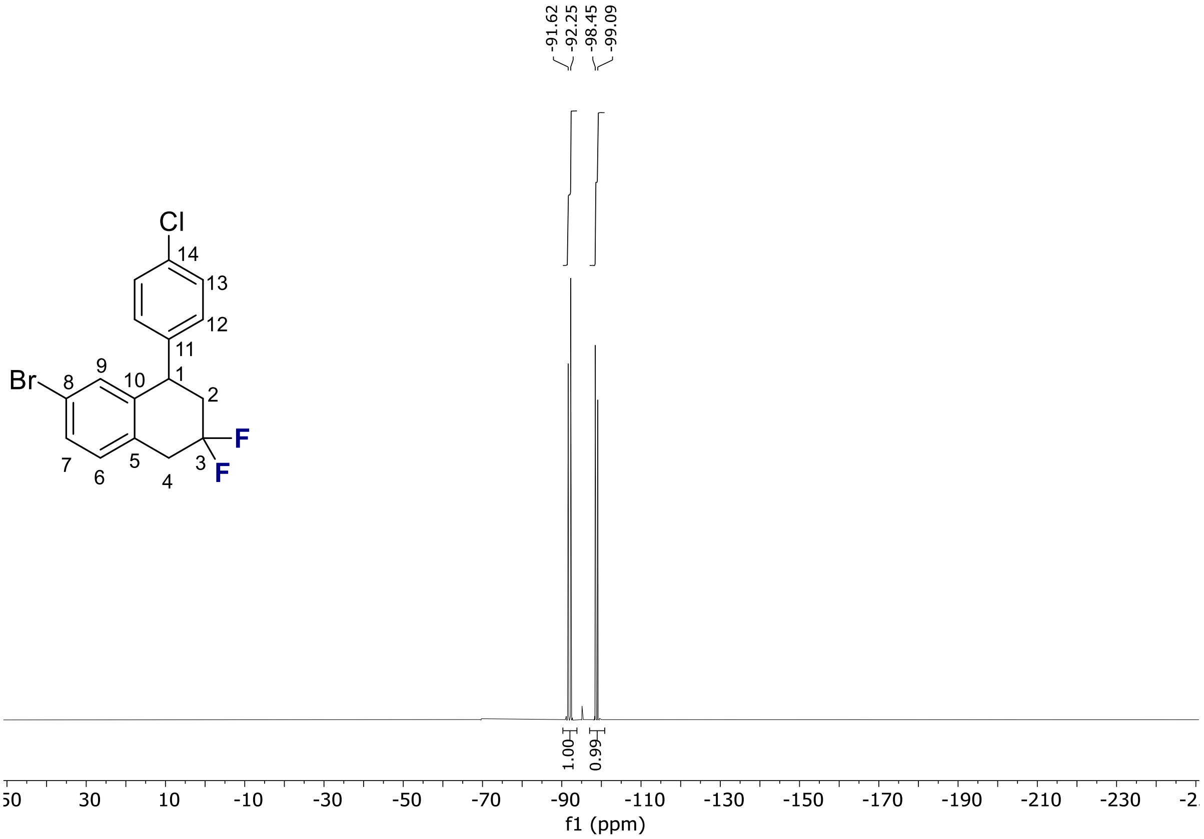


**Supplementary Figure 272.** $^{19}F\{^1H\}$ NMR of **3u** (376 MHz, 299 K, $CDCl_3$).

**7-Bromo-3,3-difluoro-1-(4-(trifluoromethyl)phenyl)-1,2,3,4-tetrahydronaphthalene (3v)**

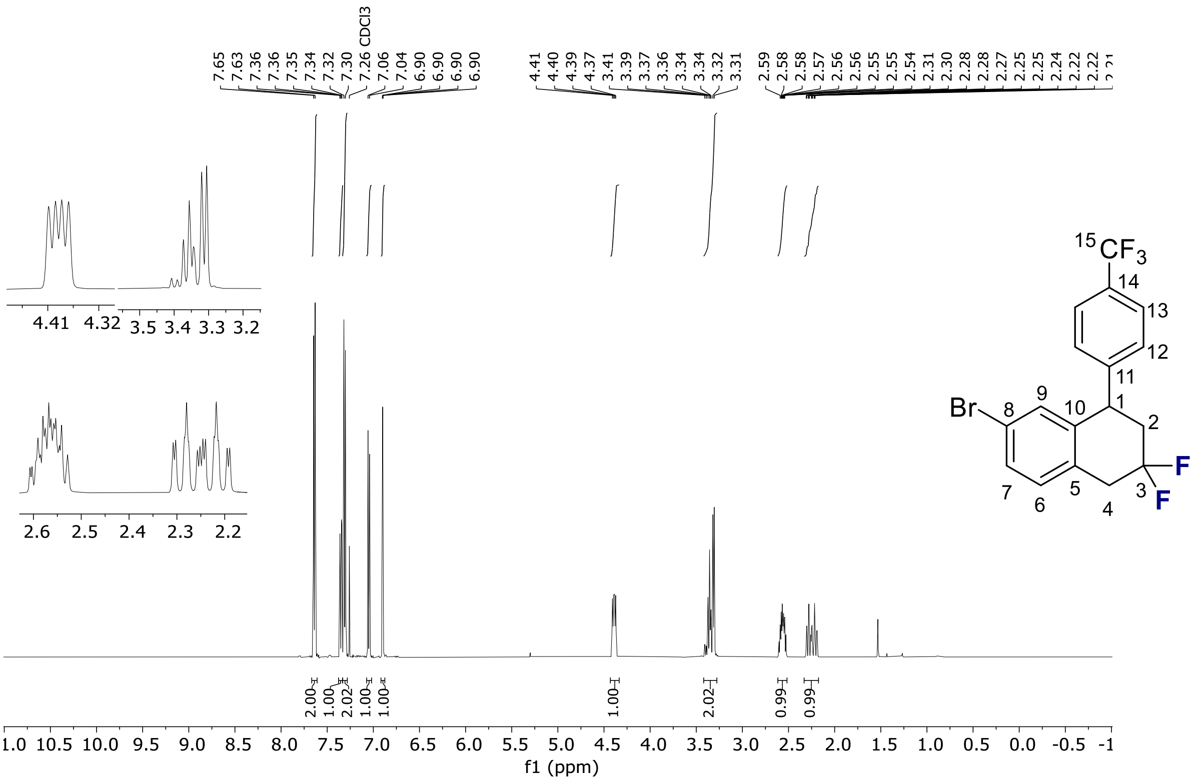


**Supplementary Figure 273.** $^{1}H$ NMR of **3v** (500 MHz, 299 K, $CDCl_3$).

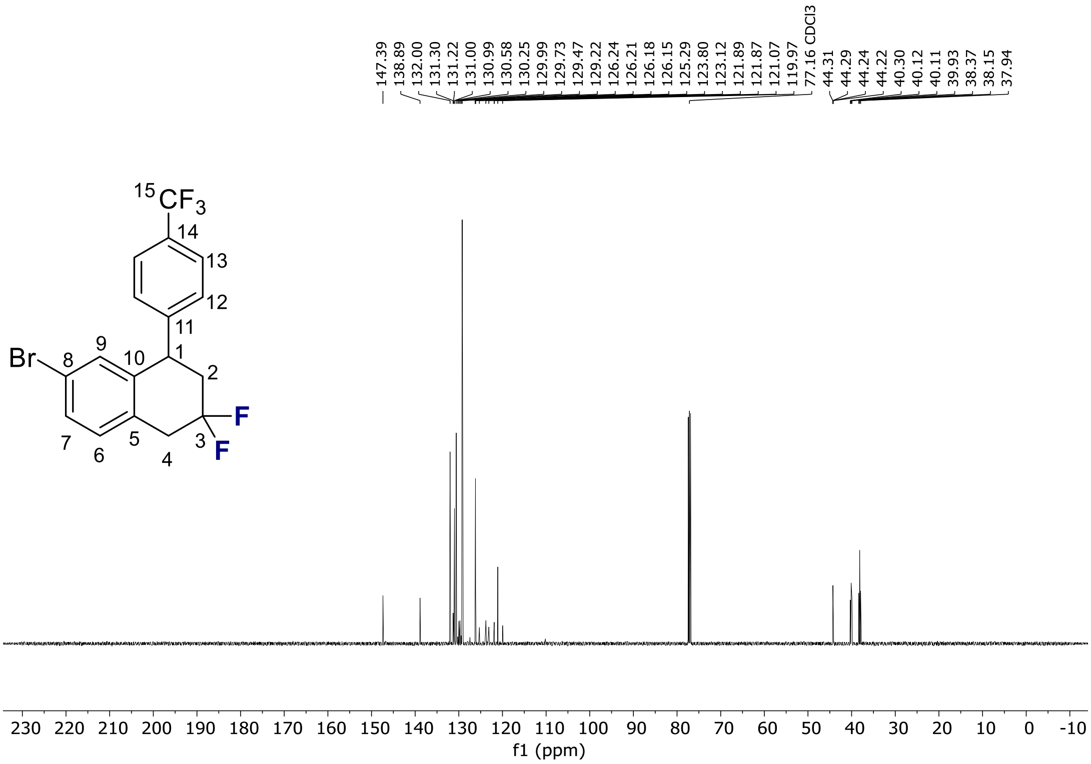


**Supplementary Figure 274.** $^{13}C\{^{1}H\}$ NMR of **3v** (126 MHz, 299 K, $CDCl_3$).

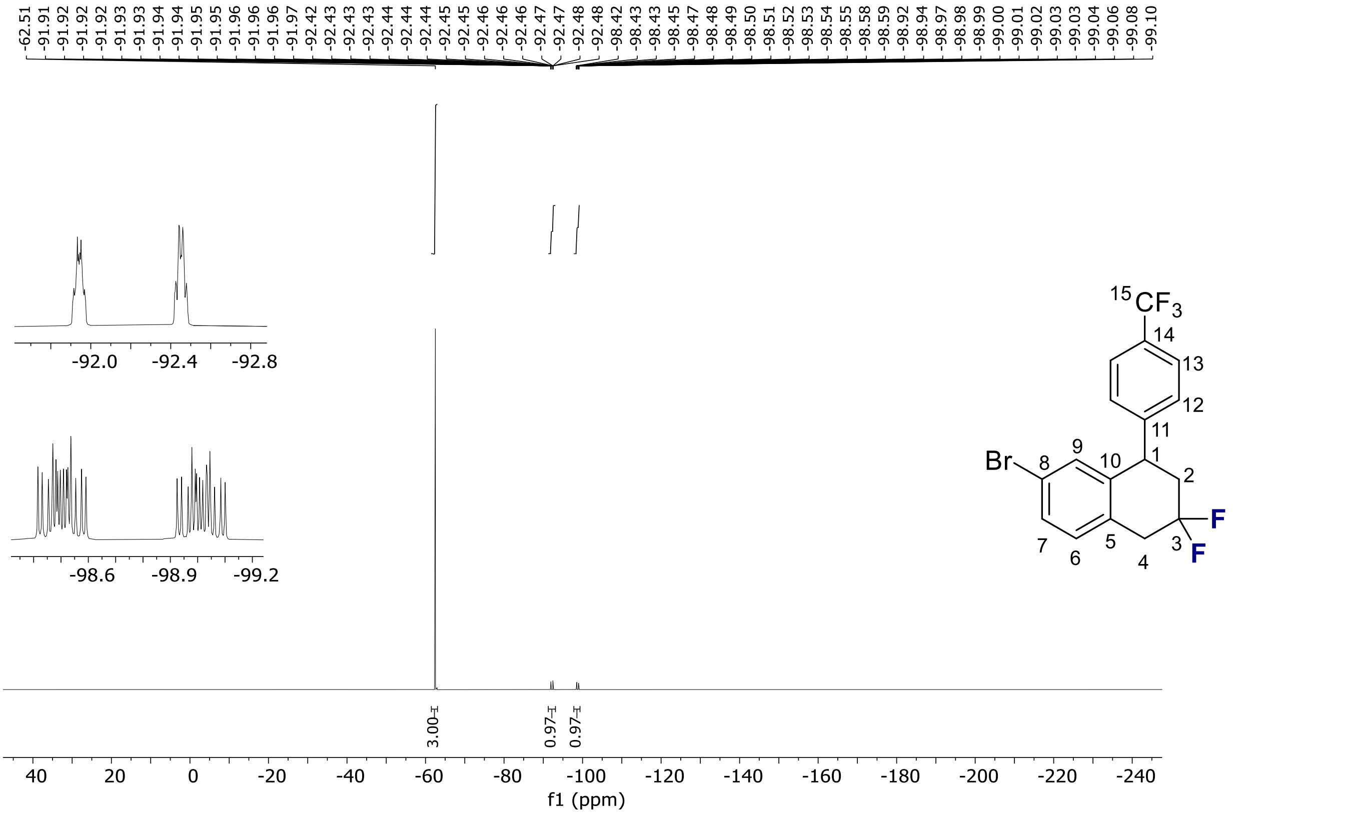


**Supplementary Figure 275.** $^{19}F$ NMR of **3v** (470 MHz, 299 K, $CDCl_3$).

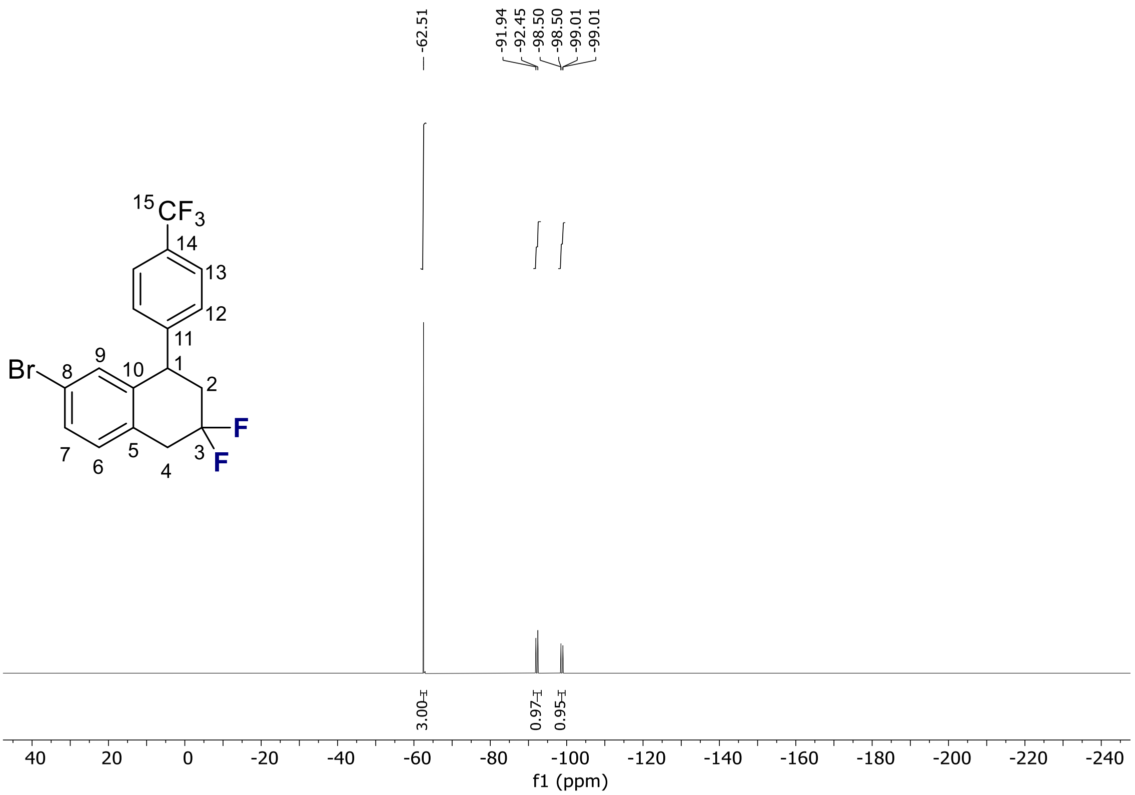


**Supplementary Figure 276.** $^{19}F\{^{1}H\}$ NMR of **3v** (470 MHz, 299 K, $CDCl_3$).

**4-(7-Bromo-3,3-difluoro-1,2,3,4-tetrahydronaphthalen-1-yl)phenyl trifluoromethanesulfonate (3w)**

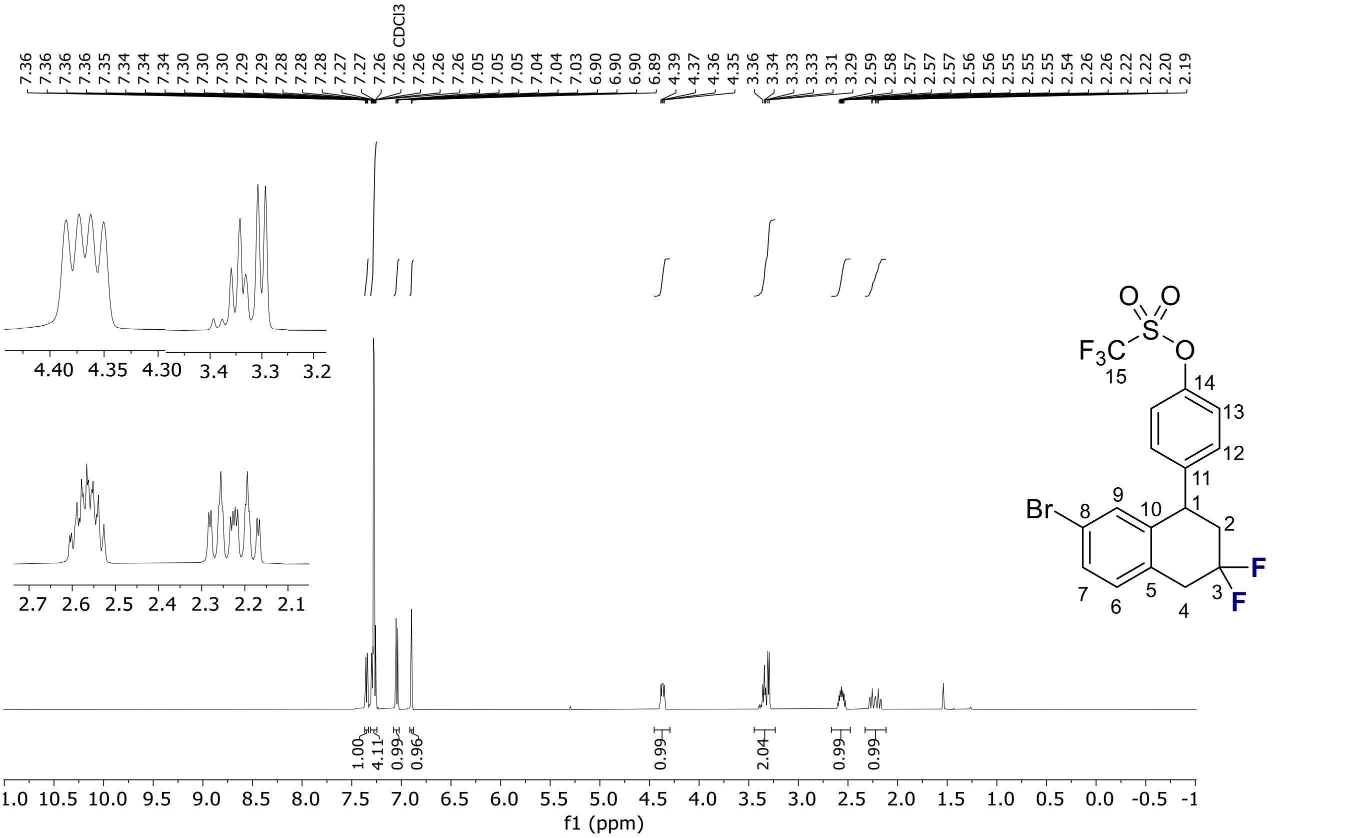


**Supplementary Figure 277.** $^{1}H$ NMR of **3w** (500 MHz, 299 K, $CDCl_3$).

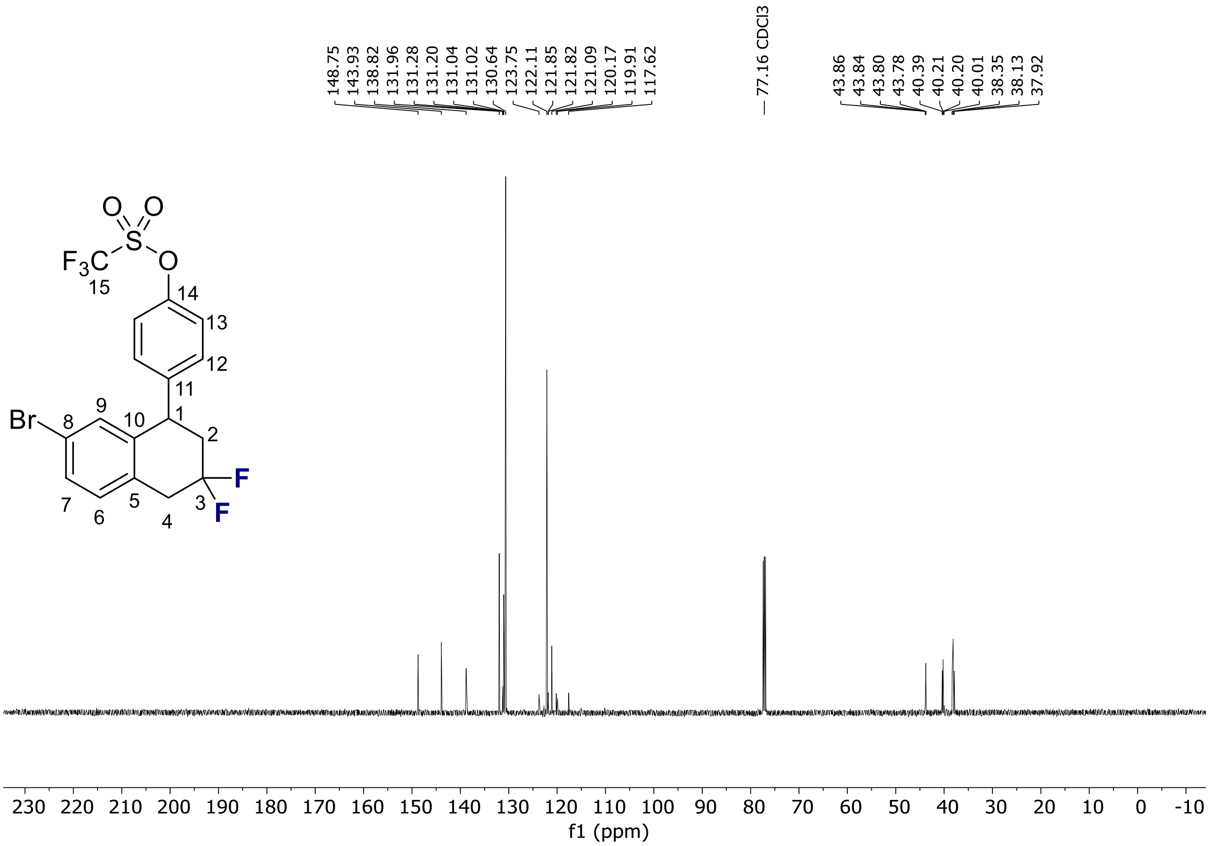


**Supplementary Figure 278.** $^{13}C\{^{1}H\}$ NMR of **3w** (126 MHz, 299 K, $CDCl_3$).

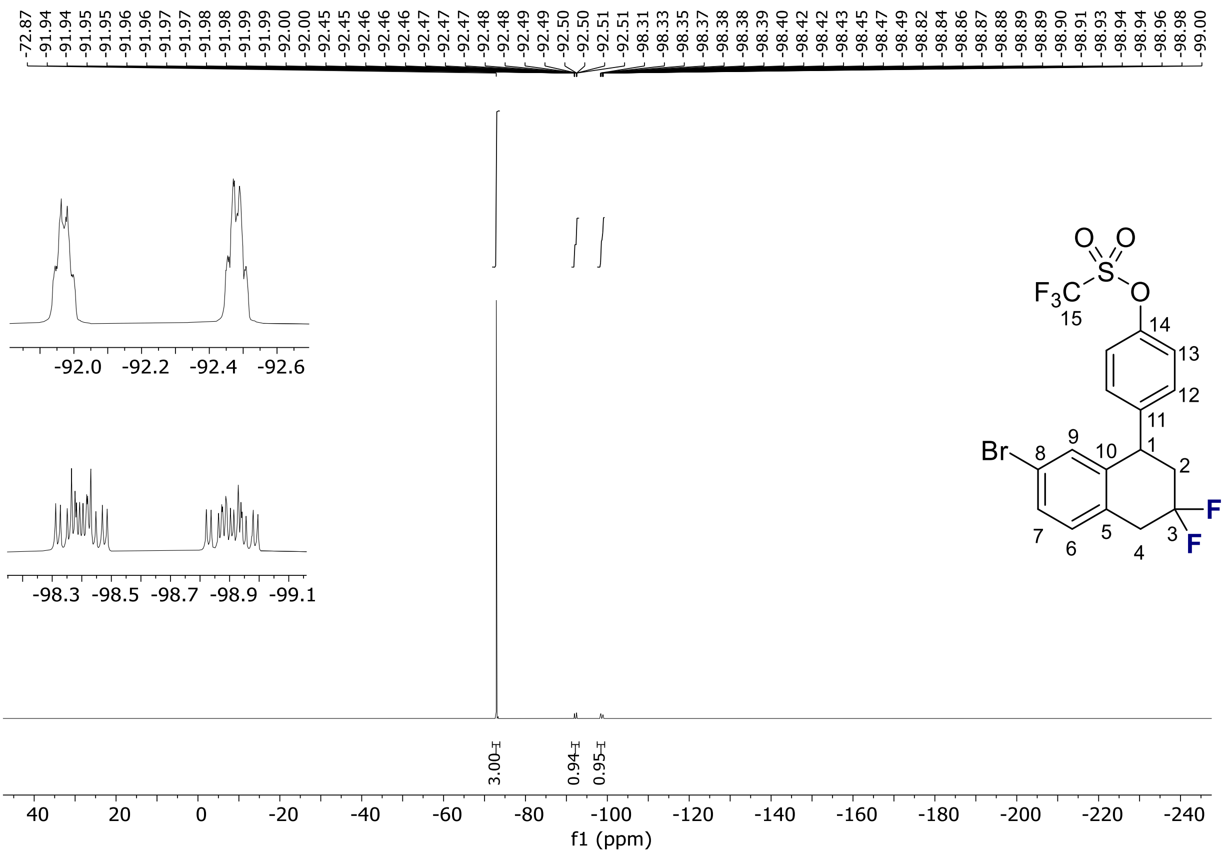

**Supplementary Figure 279.** $^{19}$F NMR of **3w** (470 MHz, 299 K, $CDCl_3$).

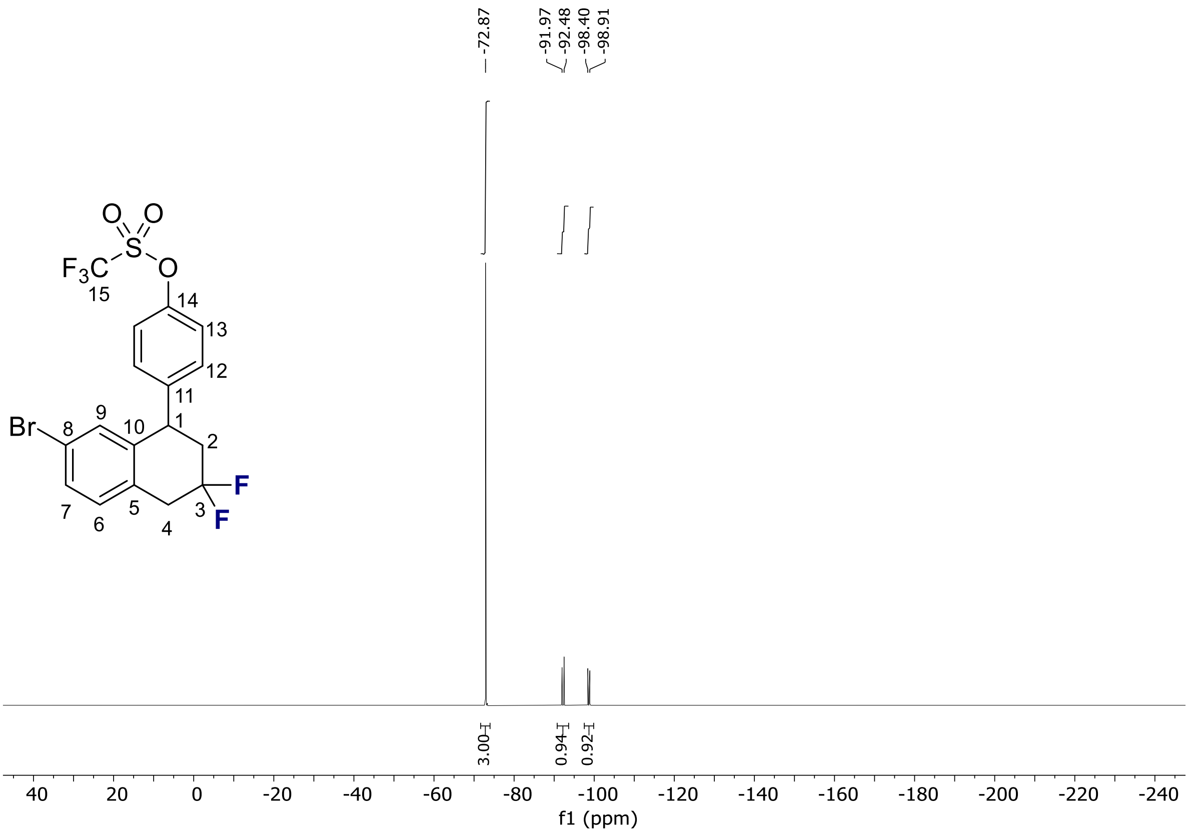

**Supplementary Figure 280.** $^{19}F\{^1H\}$ NMR of **3w** (470 MHz, 299 K, $CDCl_3$).

## 3-(6,6-Difluoro-8-phenyl-5,6,7,8-tetrahydronaphthalen-2-yl)pyridine (3y)

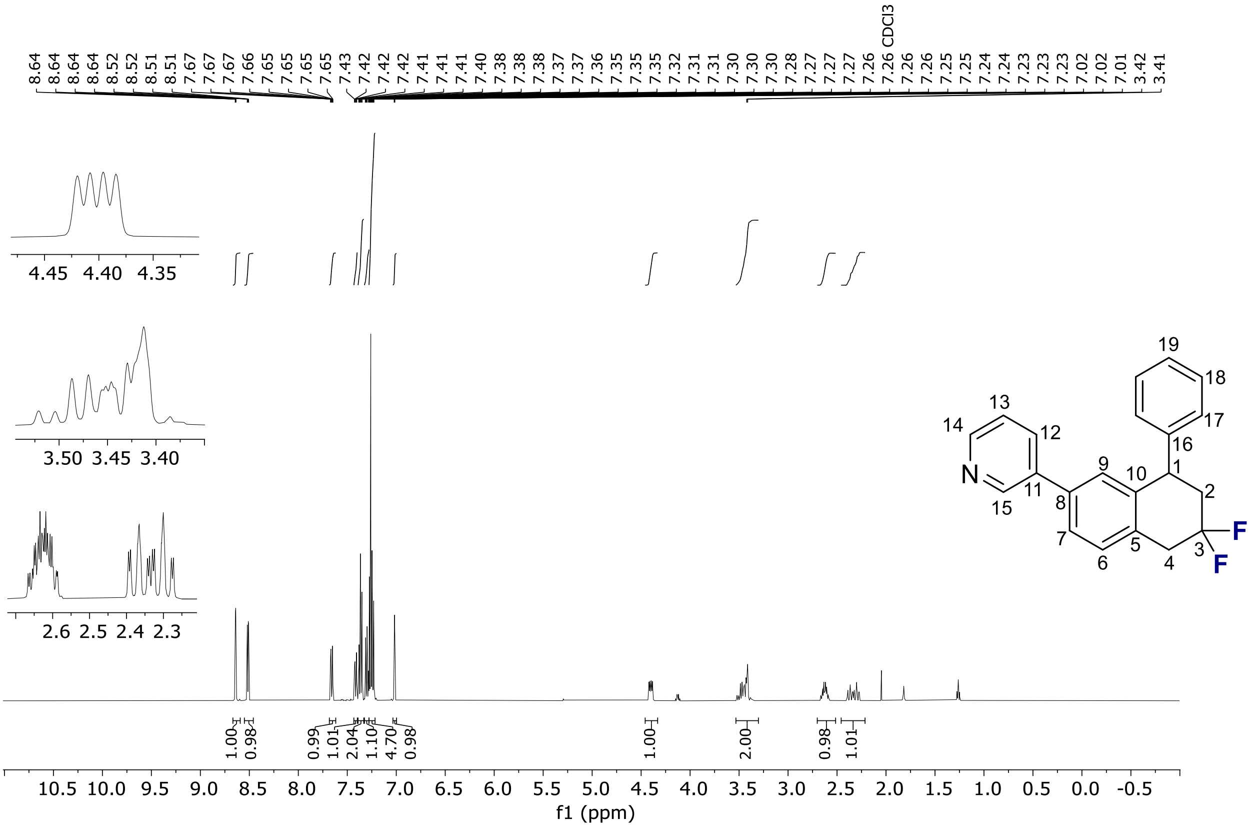


**Supplementary Figure 281.** $^{1}H$ NMR of **3y** (500 MHz, 299 K, $CDCl_3$).

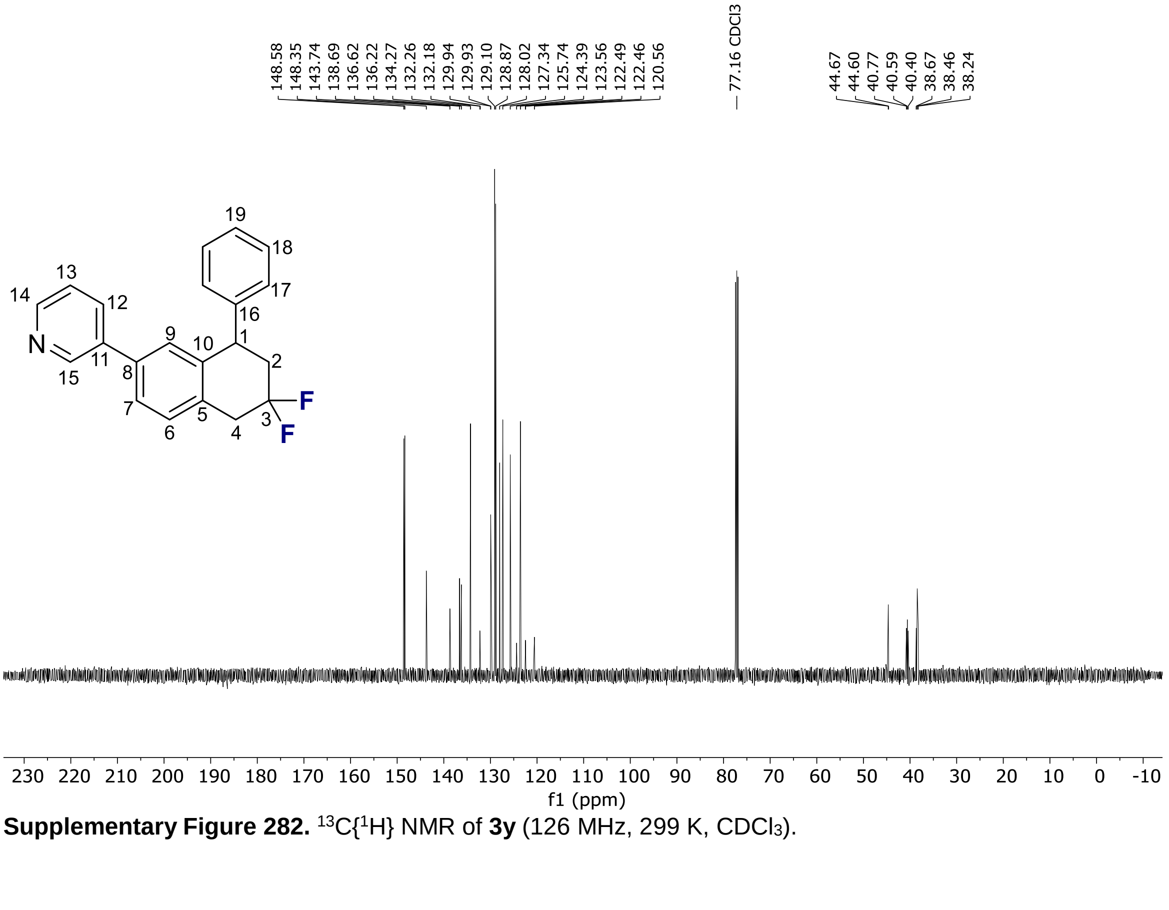


**Supplementary Figure 282.** $^{13}C\{^{1}H\}$ NMR of **3y** (126 MHz, 299 K, $CDCl_3$).

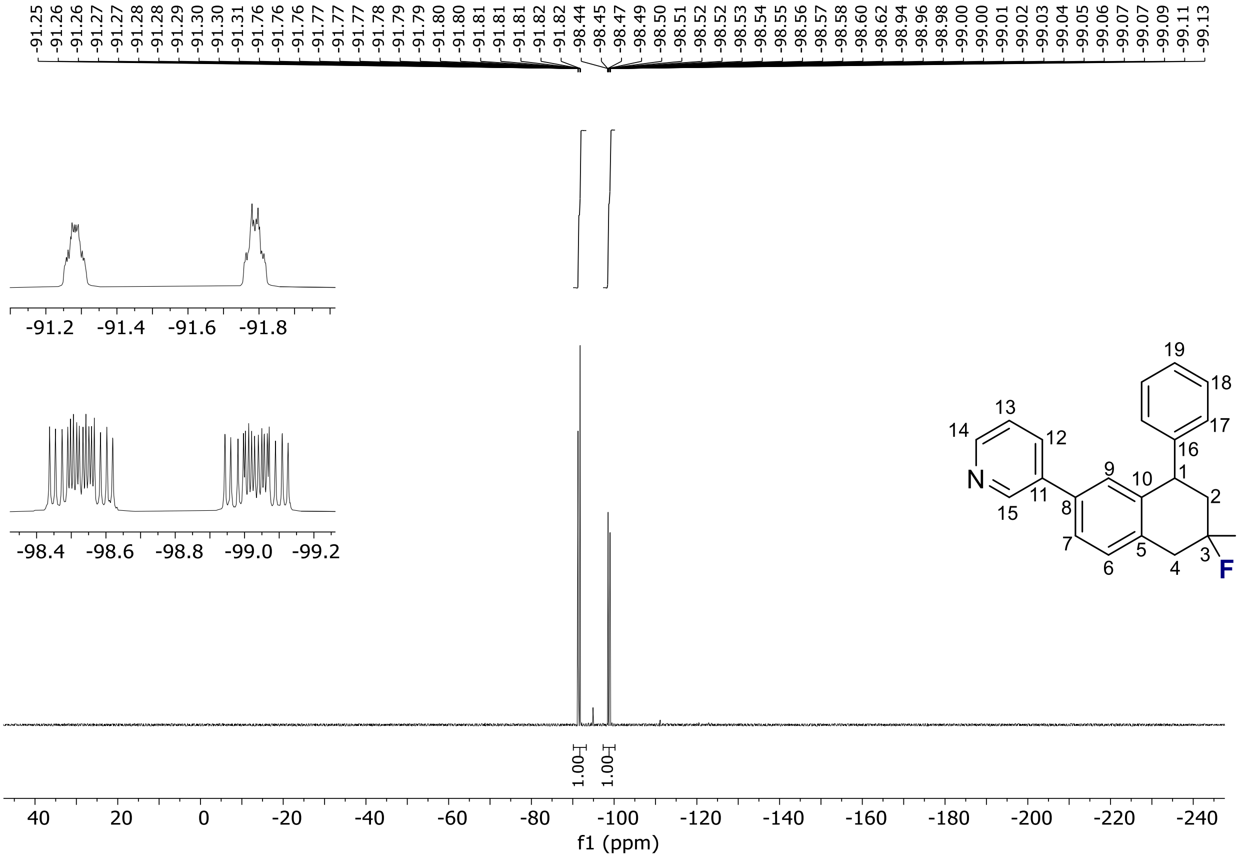


**Supplementary Figure 283.** $^{19}F$ NMR of **3y** (470 MHz, 299 K, $CDCl_3$).

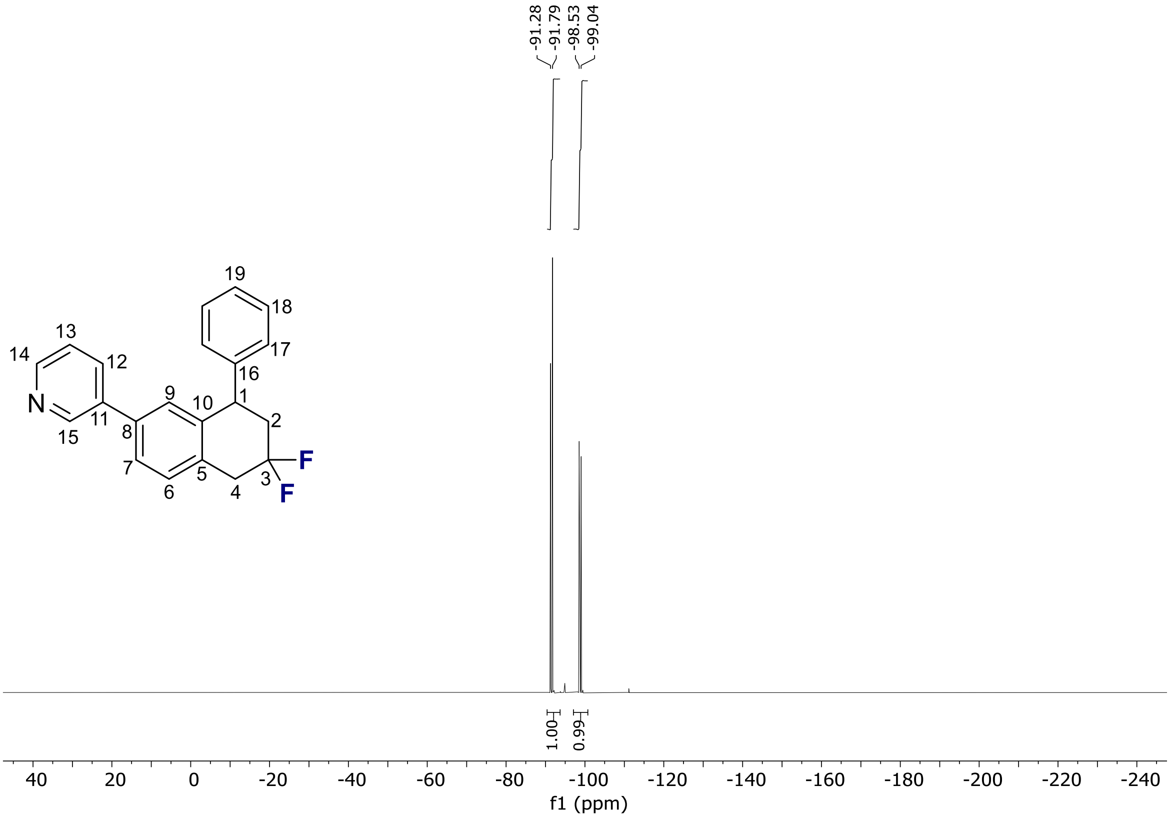


**Supplementary Figure 284.** $^{19}F\{^1H\}$ NMR of **3y** (470 MHz, 299 K, $CDCl_3$).

## 4,4'-(1-Fluorobut-3-ene-1,3-diyl)bis(chlorobenzene) (IIIc)

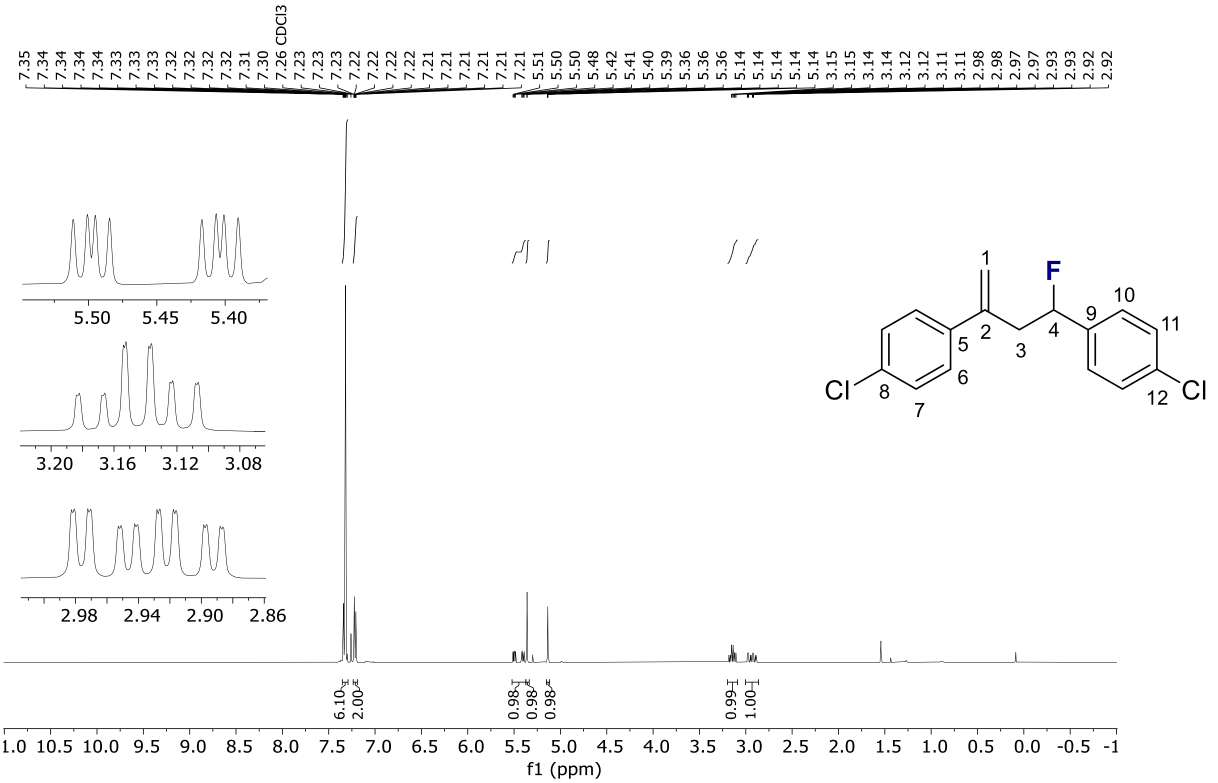


**Supplementary Figure 285.** $^{1}H$ NMR of **IIIc** (500 MHz, 299 K, $CDCl_3$).

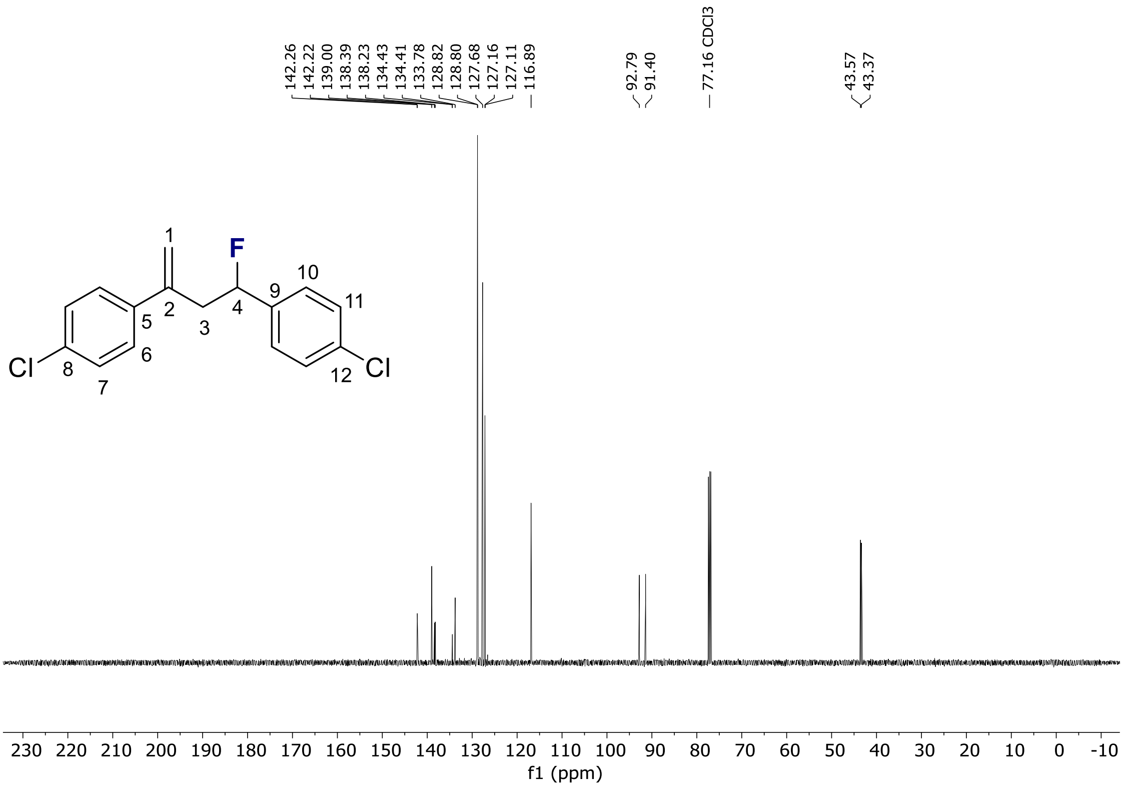


**Supplementary Figure 286.** $^{13}C\{^{1}H\}$ NMR of **IIIc** (126 MHz, 299 K, $CDCl_3$).

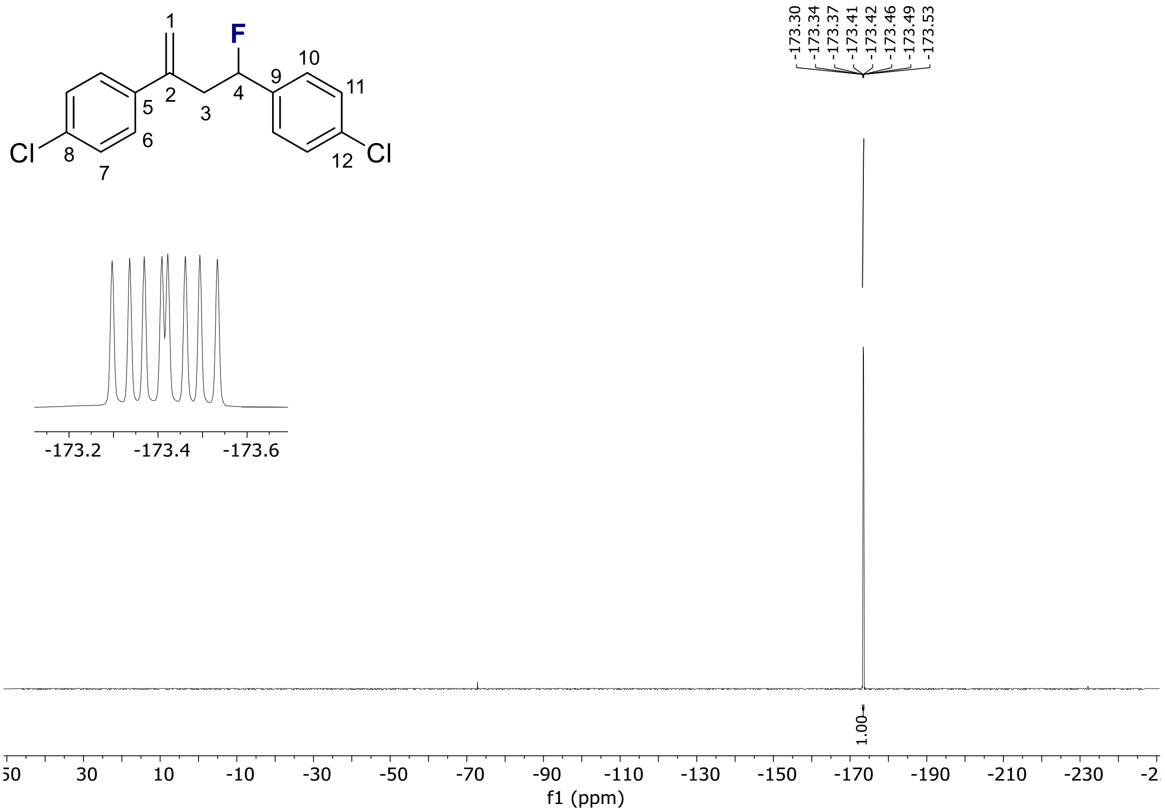


**Supplementary Figure 287.** $^{19}F$ NMR of **IIIc** (376 MHz, 299 K, $CDCl_3$).

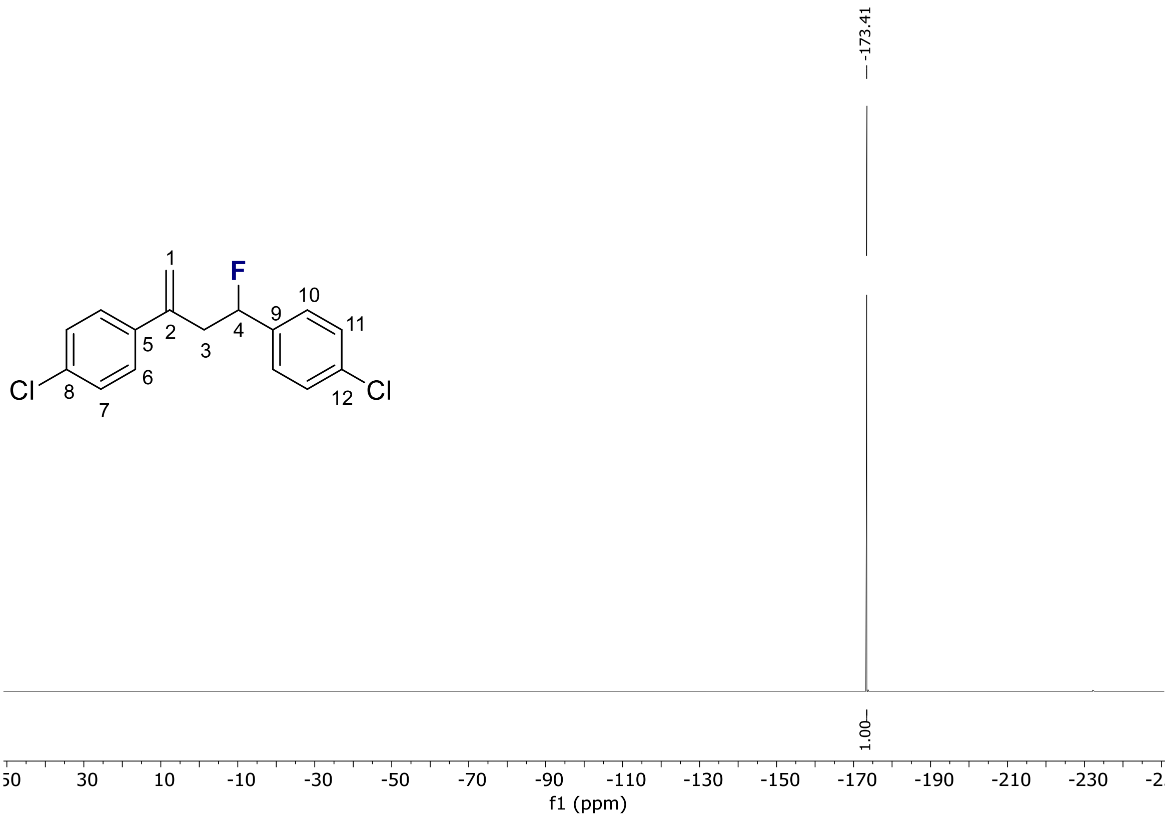


**Supplementary Figure 288.** $^{19}F\{^1H\}$ NMR of **IIIc** (376 MHz, 299 K, $CDCl_3$).

## 1,4-Bis(4-chlorophenyl)-3,3-difluorobutyl acetate (4)

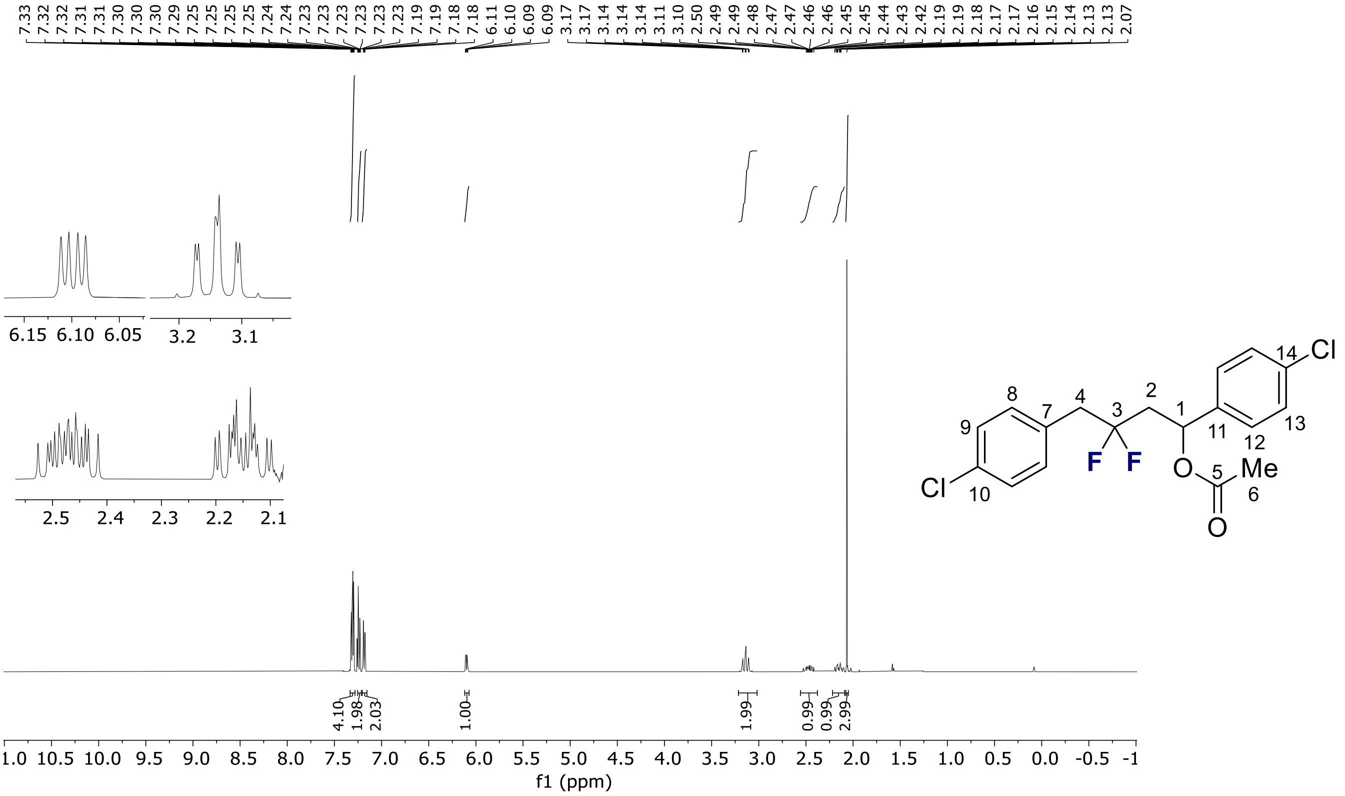


**Supplementary Figure 289.** $^{1}$H NMR of **4** (500 MHz, 299 K, $CDCl_3$).

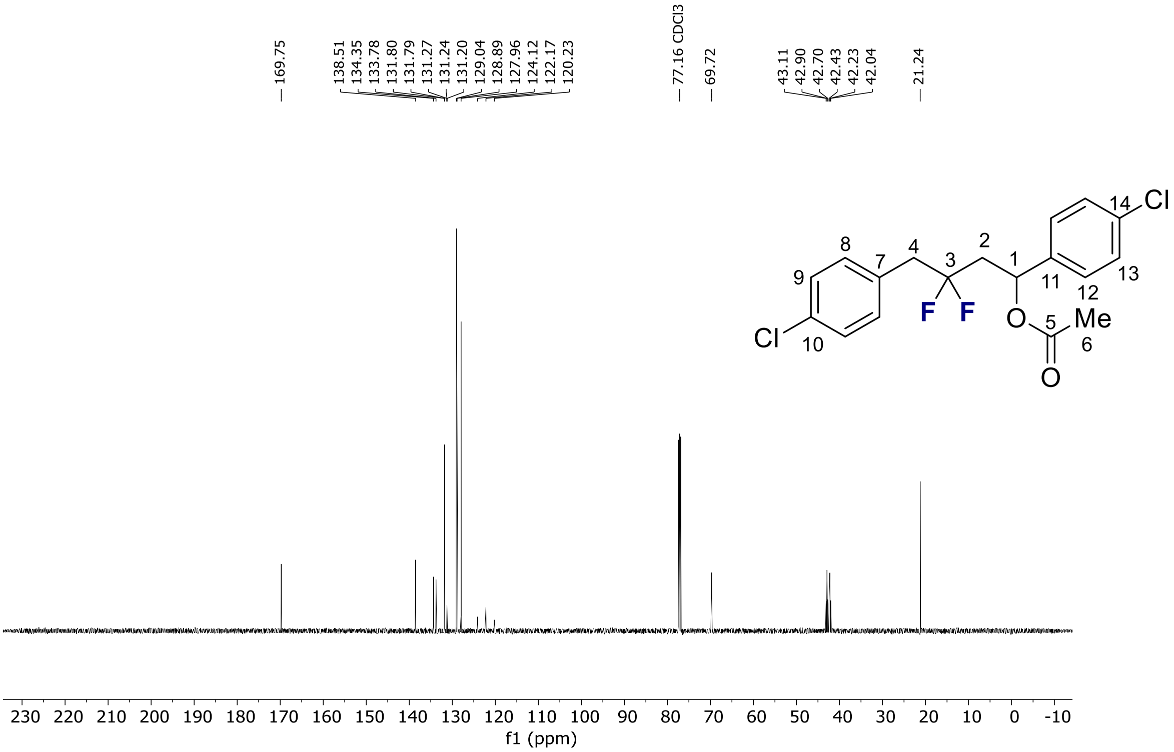


**Supplementary Figure 290.** $^{13}C\{^{1}H\}$ NMR of **4** (126 MHz, 299 K, $CDCl_3$).

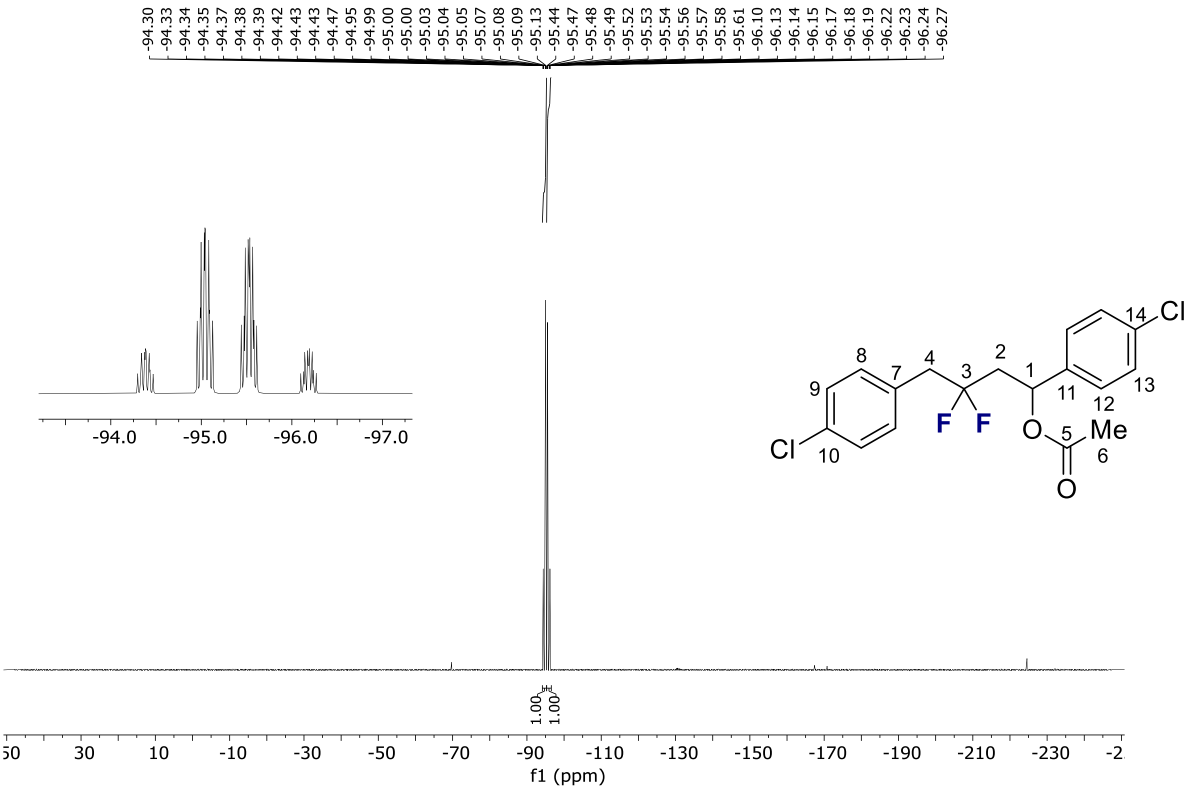


**Supplementary Figure 291.** $^{19}$F NMR of **4** (376 MHz, 299 K, $CDCl_3$).

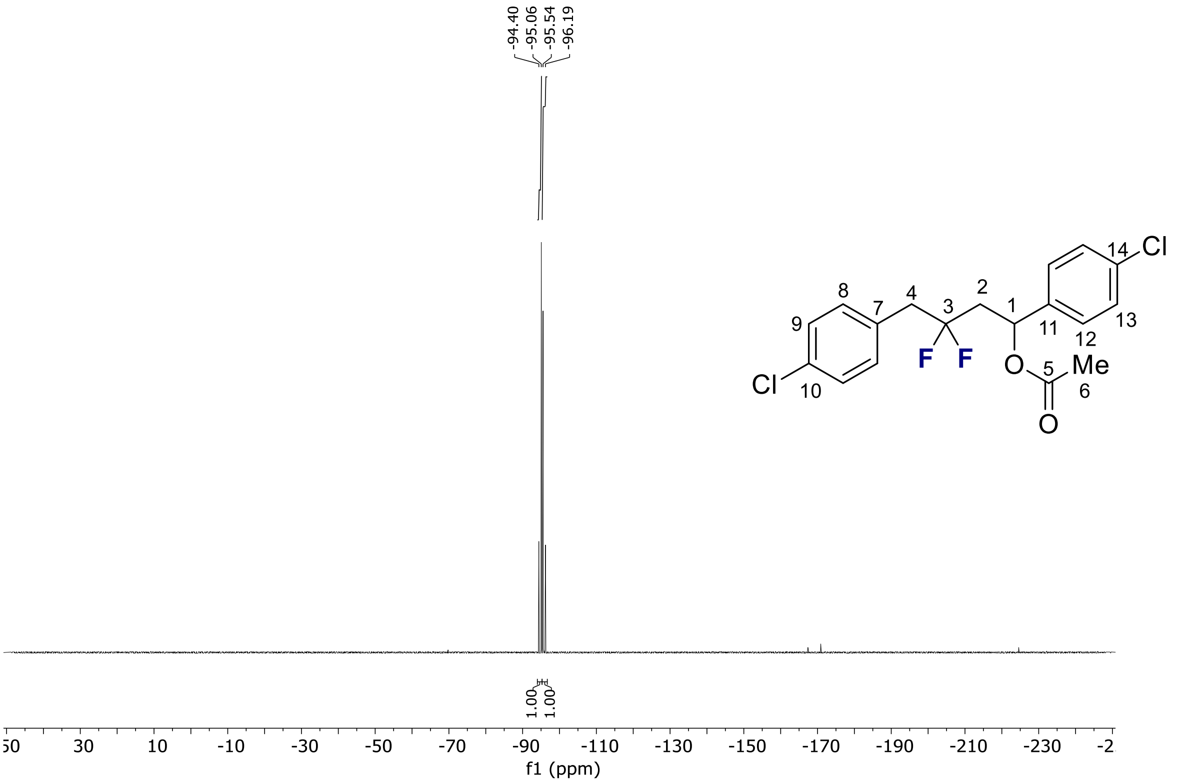


**Supplementary Figure 292.**$^{19}$F{$^1$H} NMR of **4** (376 MHz, 299 K, $CDCl_3$).

**4,4'-(3,3-Difluoro-1-methoxybutane-1,4-diyl)bis(chlorobenzene) (5)**

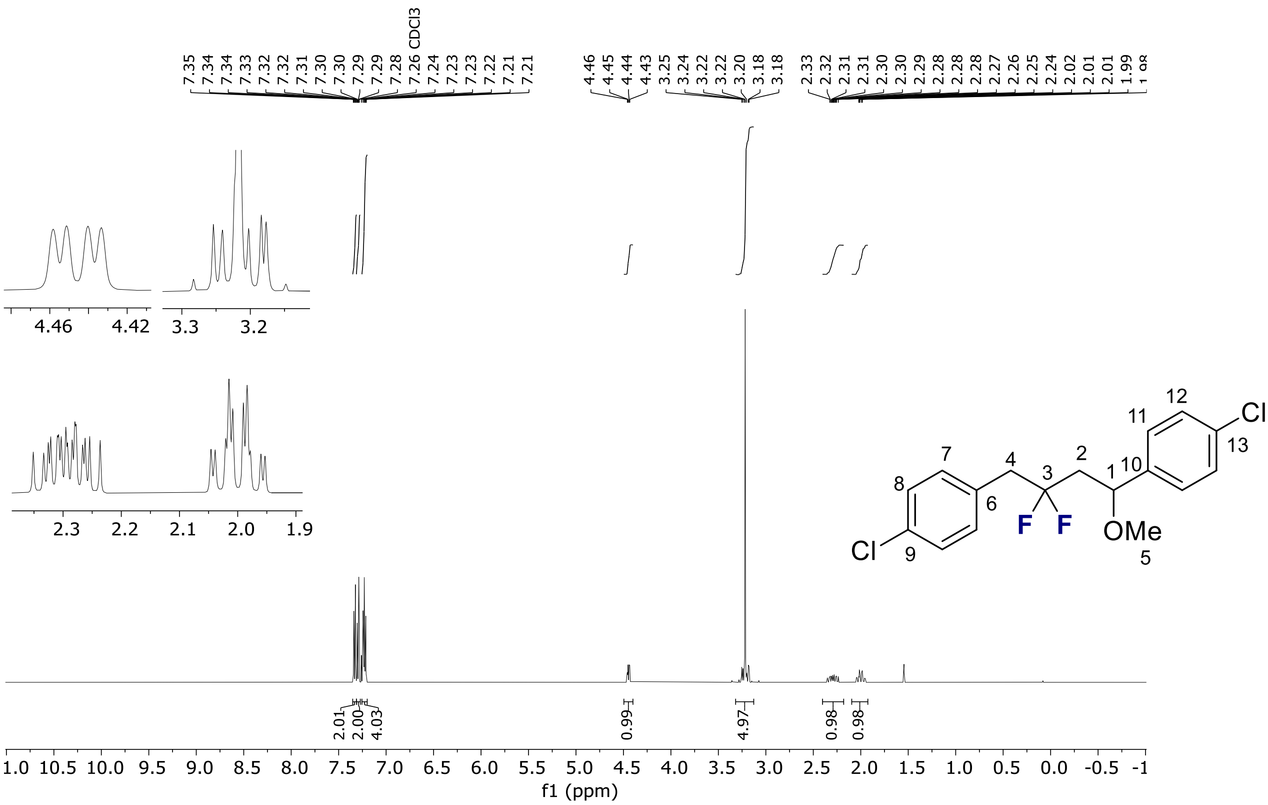


**Supplementary Figure 293.** $^{1}$H NMR of **5** (500 MHz, 299 K, $CDCl_3$).

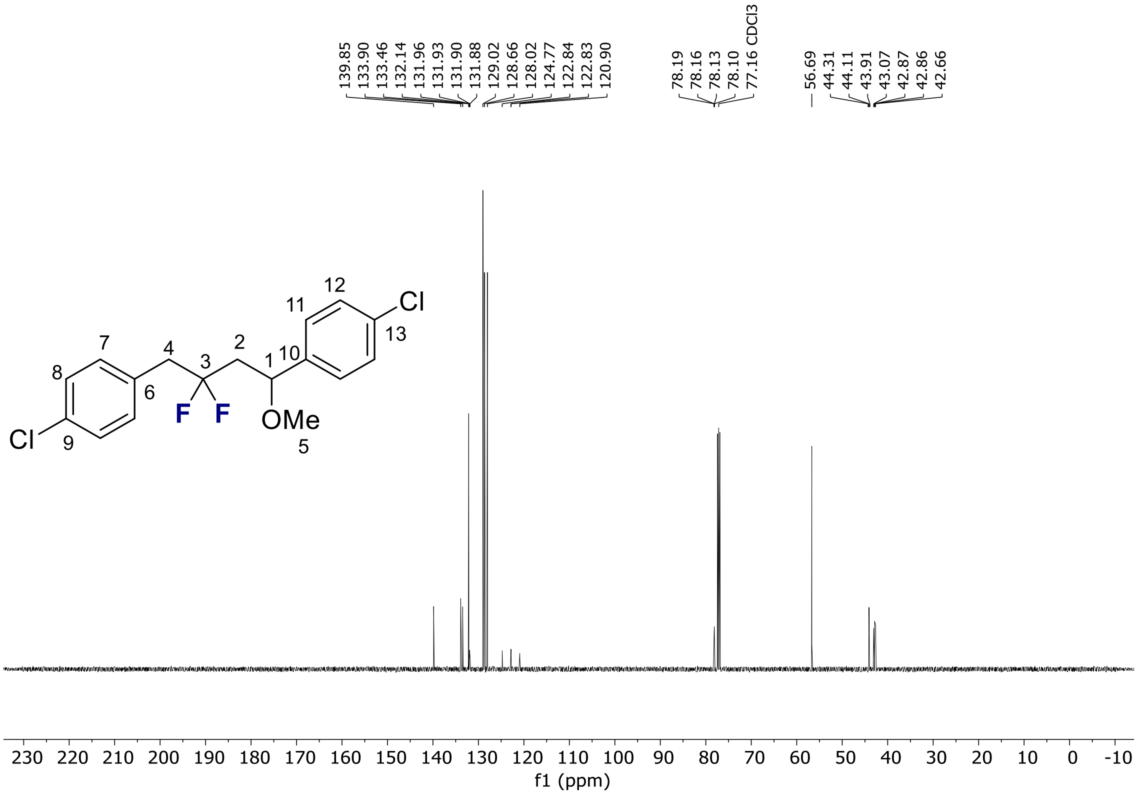


**Supplementary Figure 294.** $^{13}C\{^{1}H\}$ NMR of **5** (126 MHz, 299 K, $CDCl_3$).

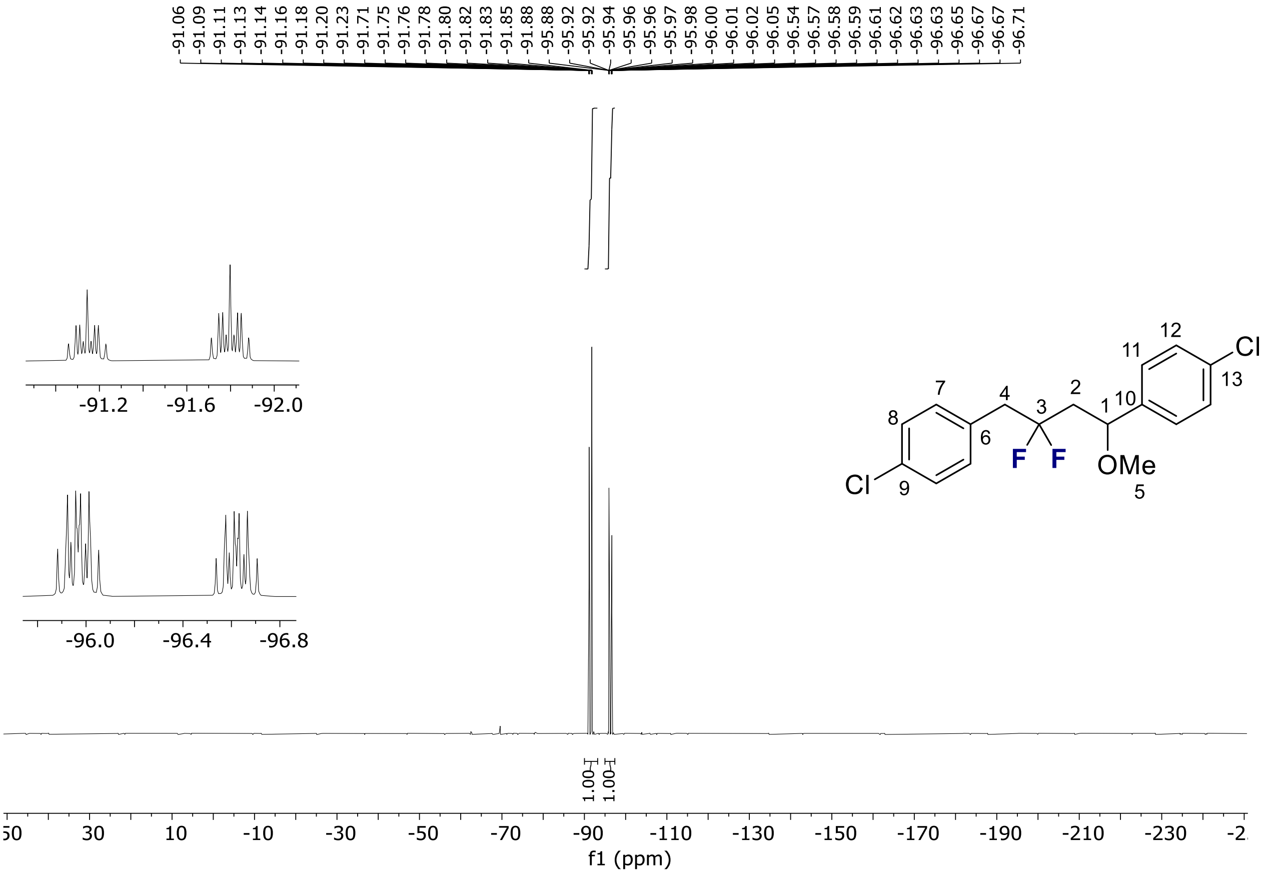

**Supplementary Figure 295.** $^{19}$F NMR of **5** (376 MHz, 299 K, $CDCl_3$).

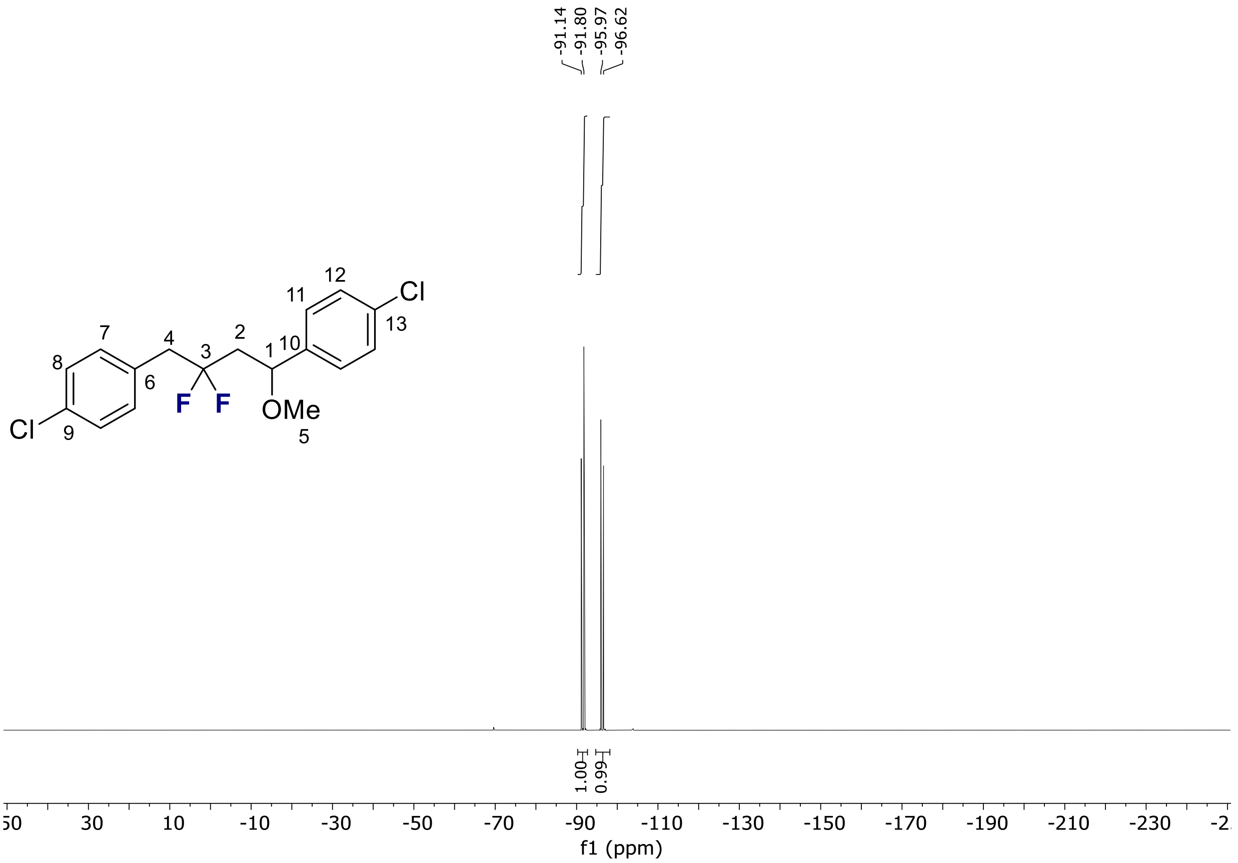

**Supplementary Figure 296.** $^{19}$F{$^{1}$H} NMR of **5** (376 MHz, 299 K, $CDCl_3$).

**4,4'-(1-(2,5-Dimethylphenyl)-3,3-difluorobutane-1,4-diyl)bis(chlorobenzene) (6)**

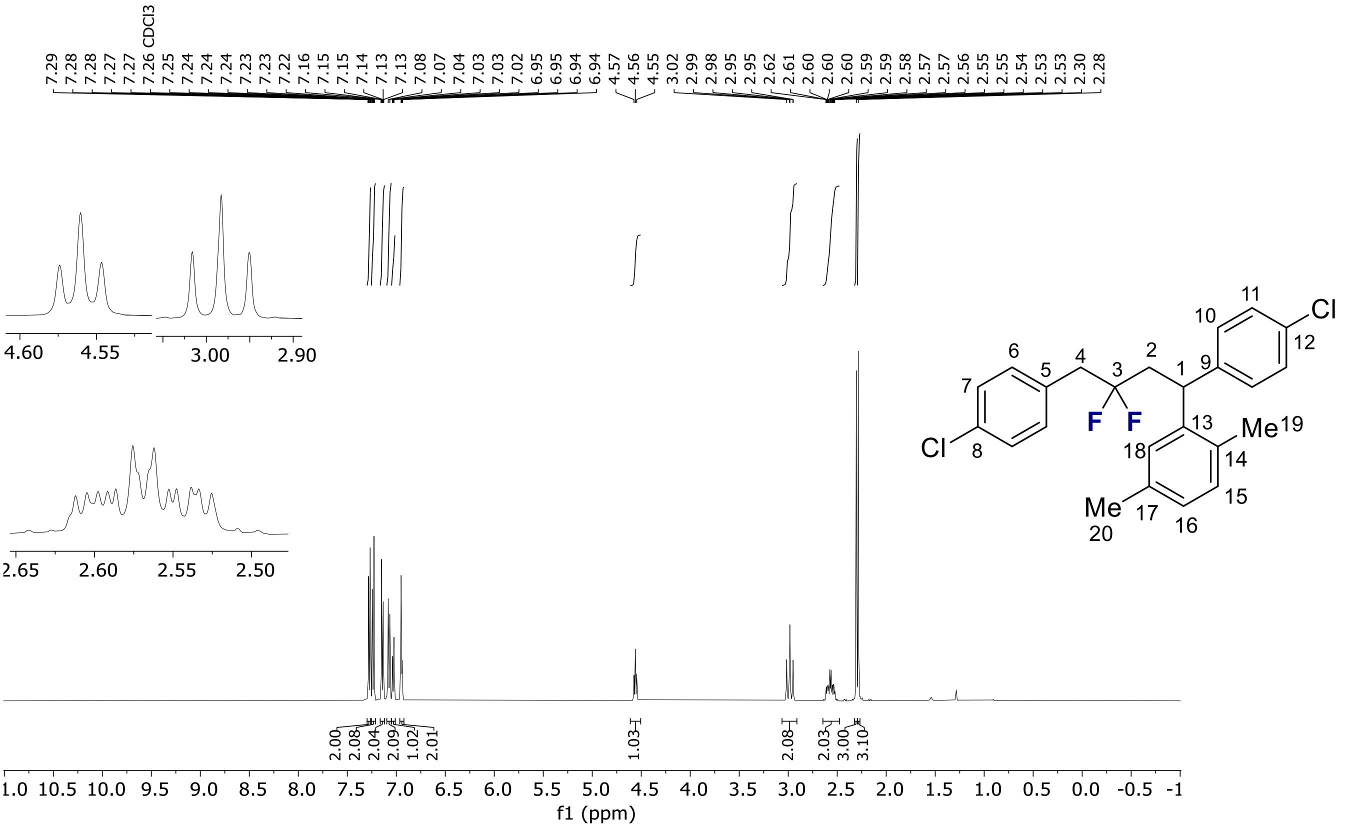


**Supplementary Figure 297.** $^{1}H$ NMR of **6** (500 MHz, 299 K, $CDCl_3$).

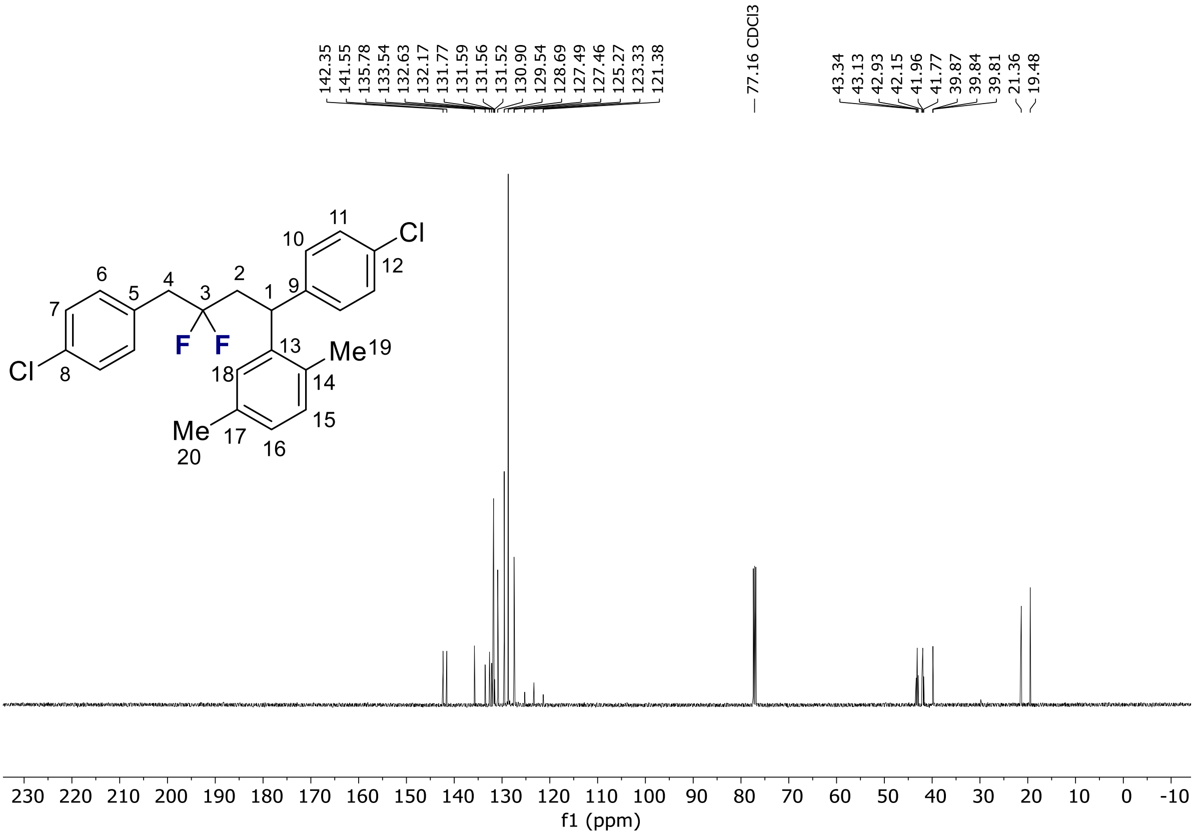


**Supplementary Figure 298.** $^{13}C\{^{1}H\}$ NMR of **6** (126 MHz, 299 K, $CDCl_3$).

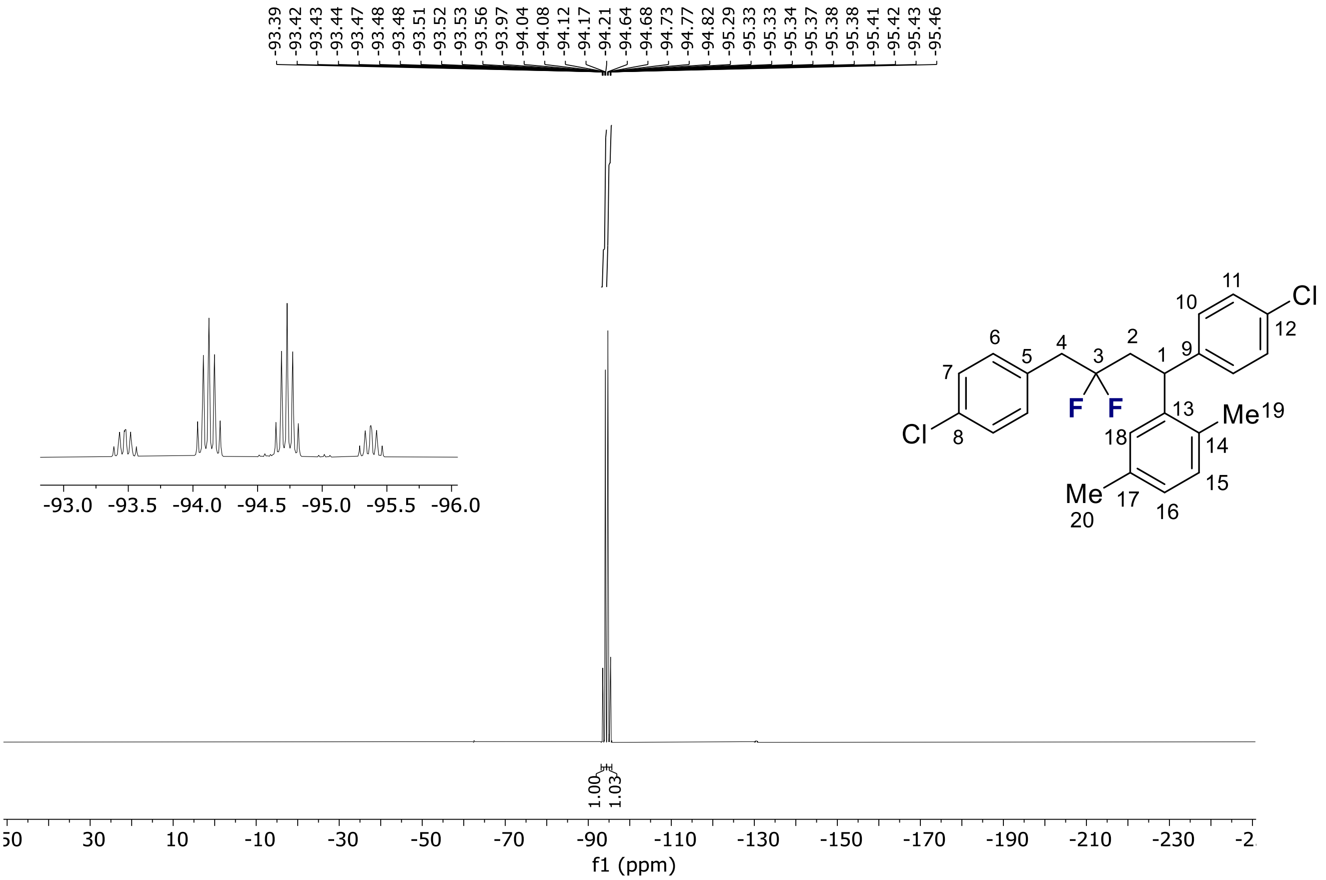


**Supplementary Figure 299.** $^{19}$F NMR of **6** (376 MHz, 299 K, $CDCl_3$).

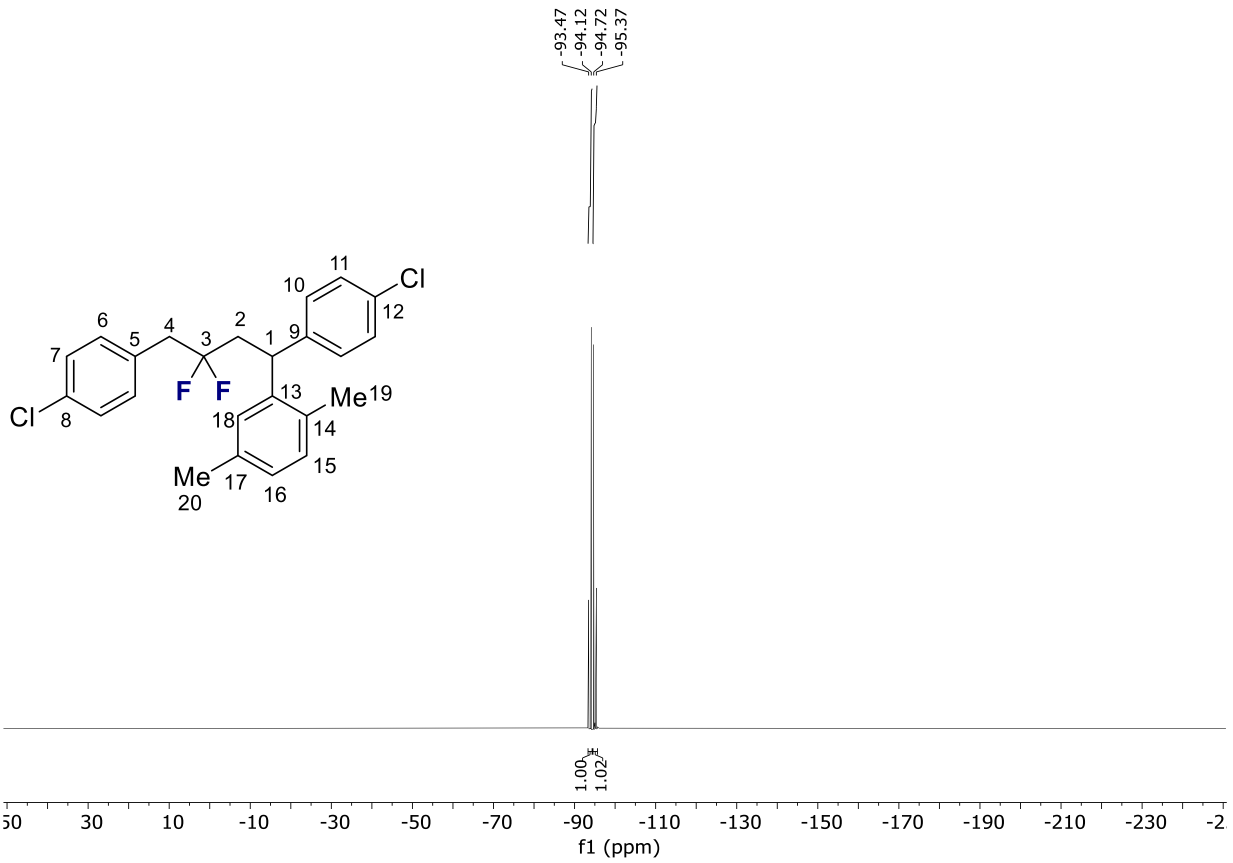


**Supplementary Figure 300.** $^{19}$F{$^1$H} NMR of **6** (376 MHz, 299 K, $CDCl_3$).

**2-((1,4-Bis(4-chlorophenyl)-3,3-difluorobutyl)thio)benzo[d]thiazole (7)**

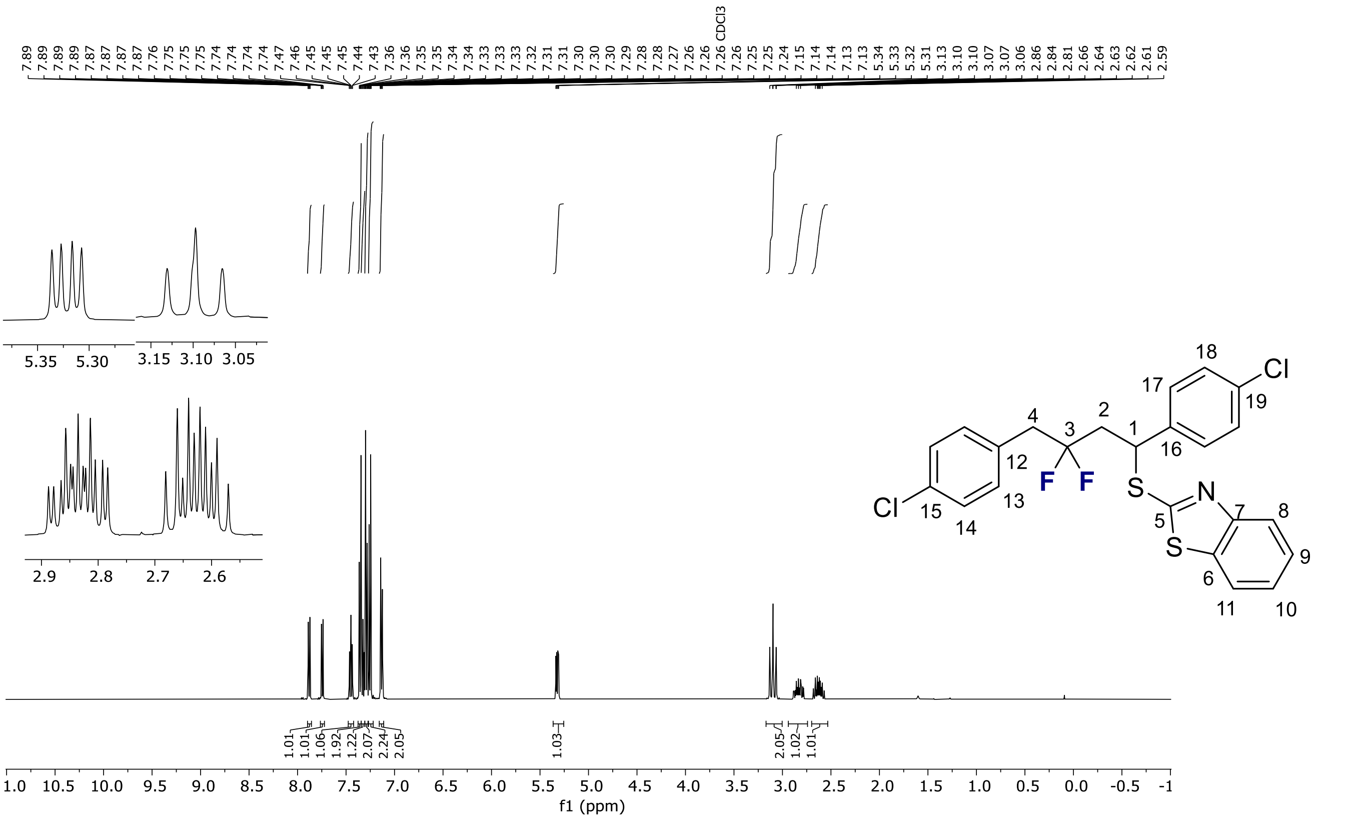


**Supplementary Figure 301.** $^{1}H$ NMR of **7** (500 MHz, 299 K, $CDCl_3$).

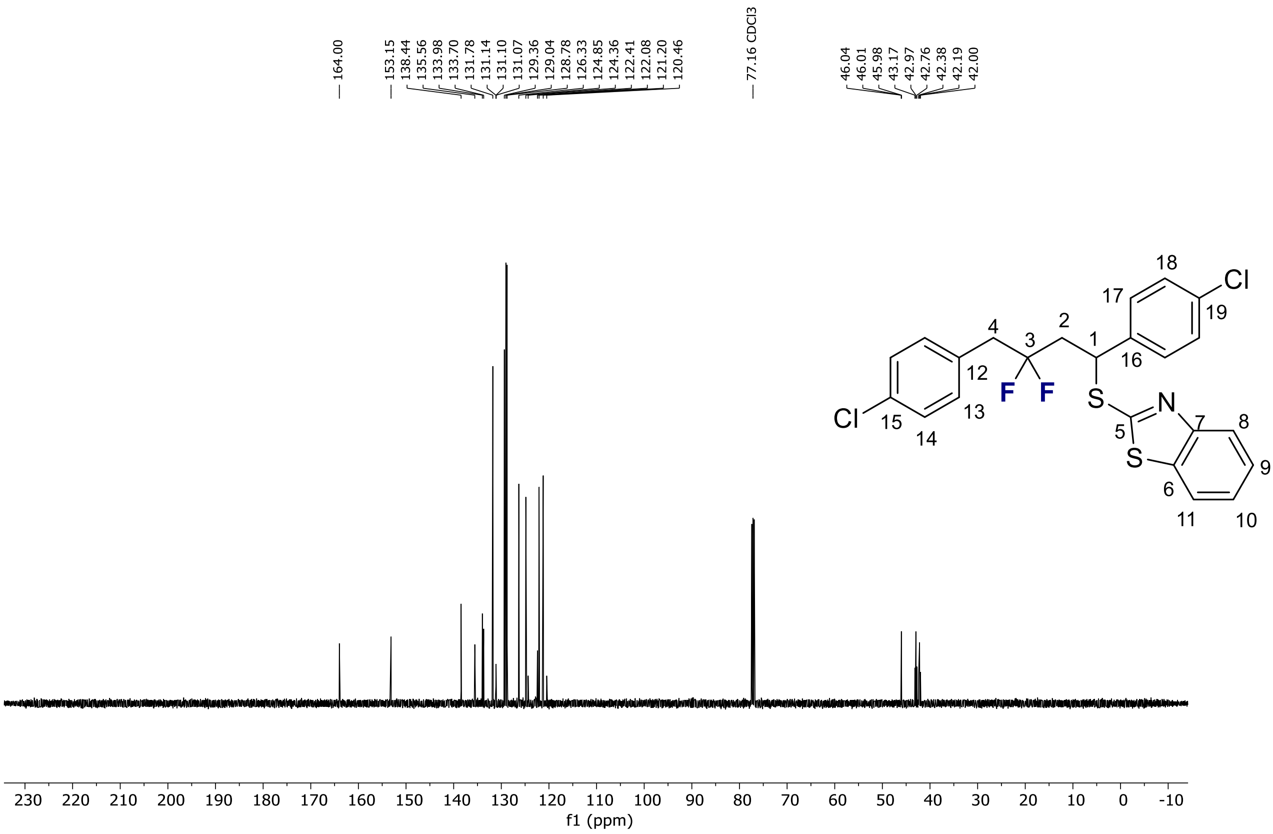


**Supplementary Figure 302.** $^{13}C\{^{1}H\}$ NMR of **7** (126 MHz, 299 K, $CDCl_3$).

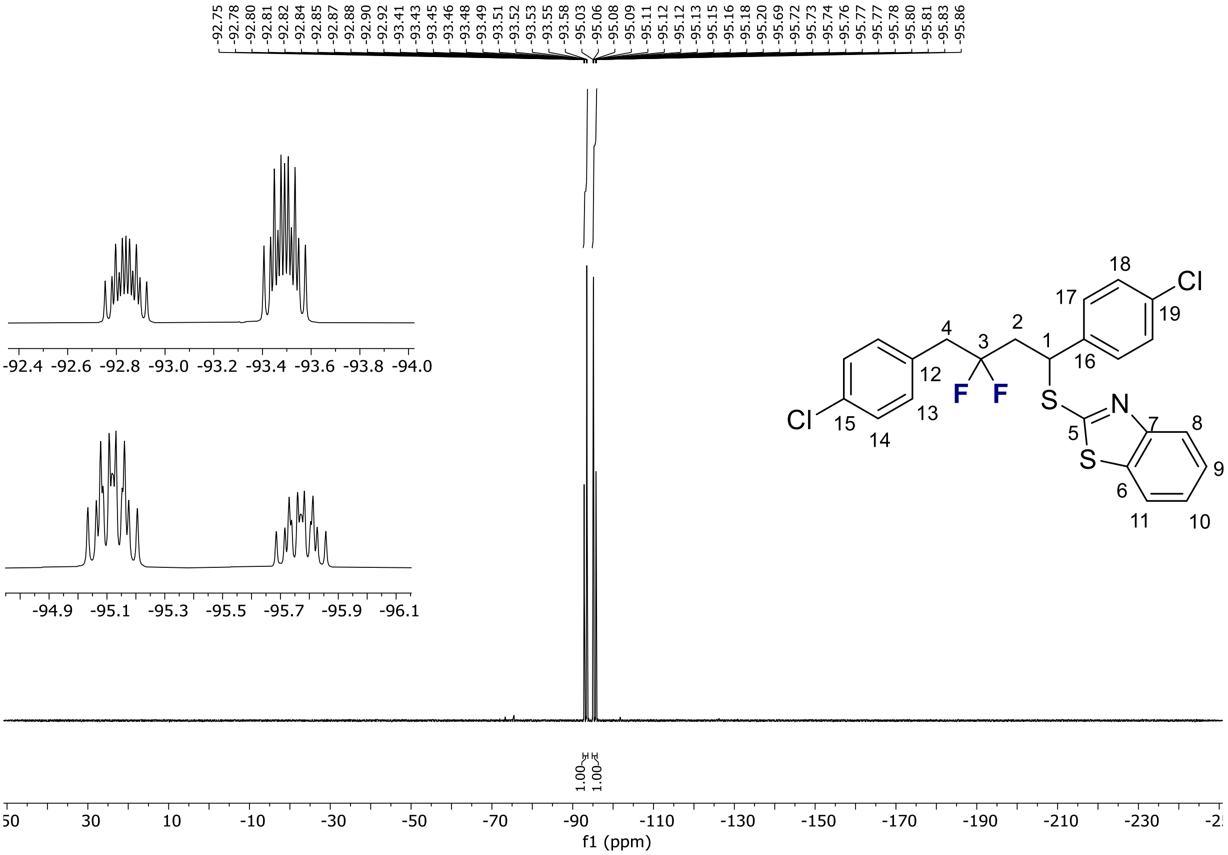

**Supplementary Figure 303.** $^{19}F$ NMR of **7** (376 MHz, 299 K, $CDCl_3$).

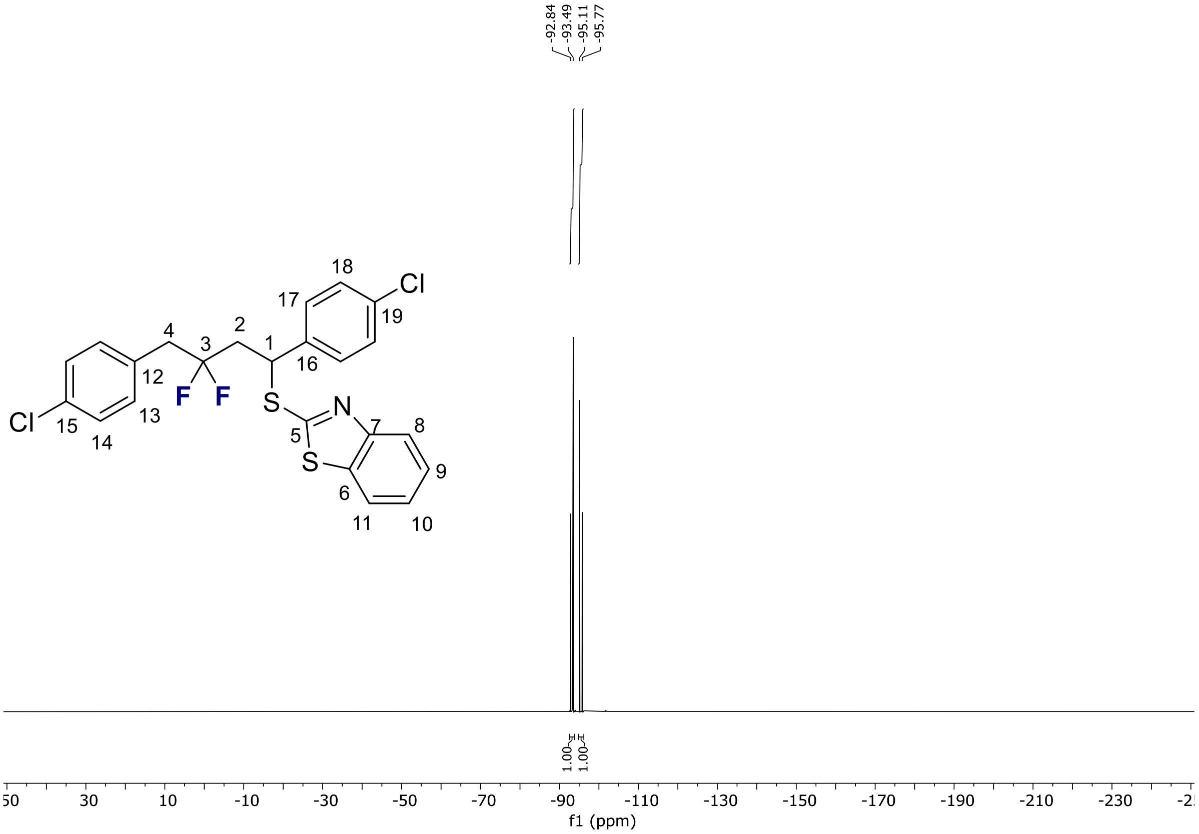

**Supplementary Figure 304.** $^{19}F\{^1H\}$ NMR of **7** (376 MHz, 299 K, $CDCl_3$).

**2-((1,4-Bis(4-chlorophenyl)-3,3-difluorobutyl)sulfonyl)benzo[d]thiazole (8)**

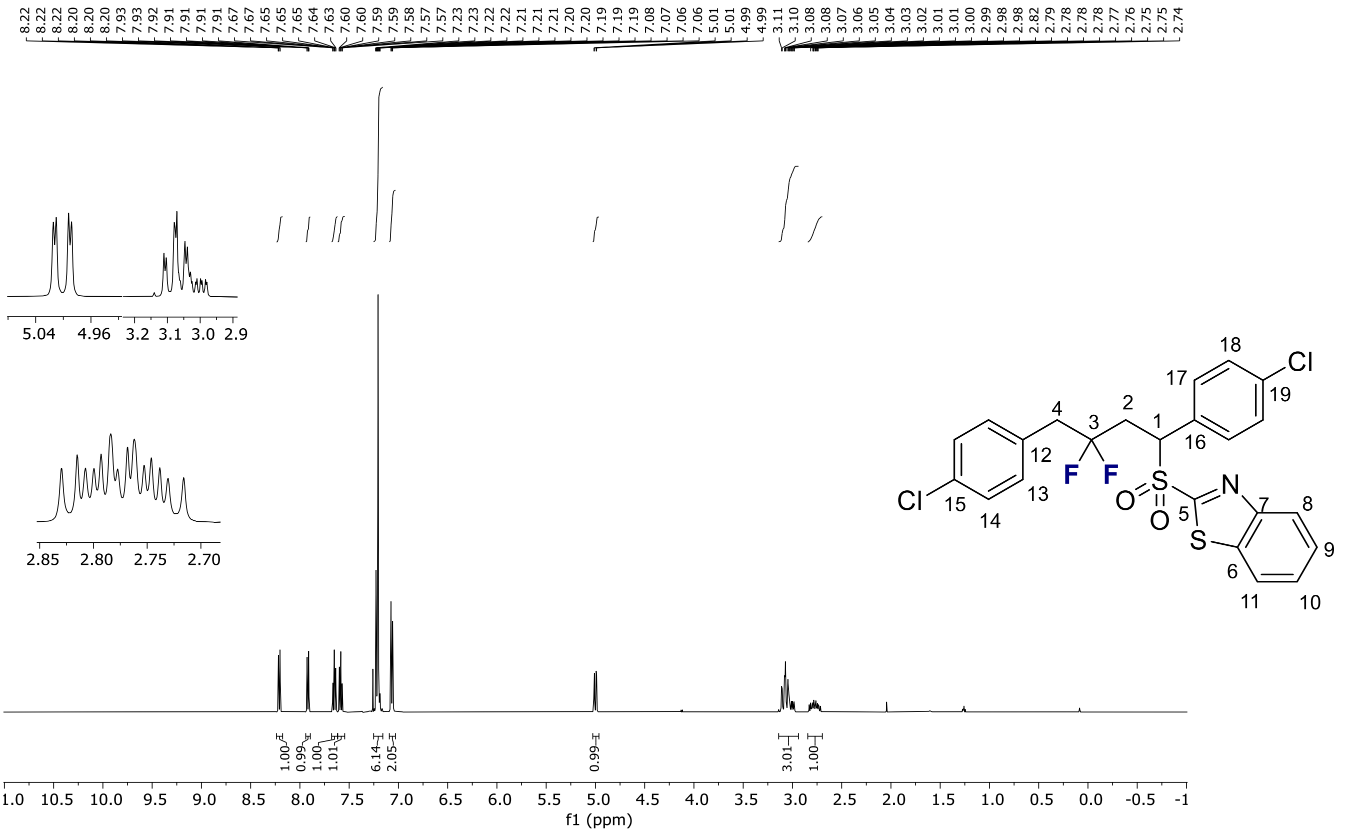

**Supplementary Figure 305.** $^{1}H$ NMR of **8** (500 MHz, 299 K, $CDCl_3$).

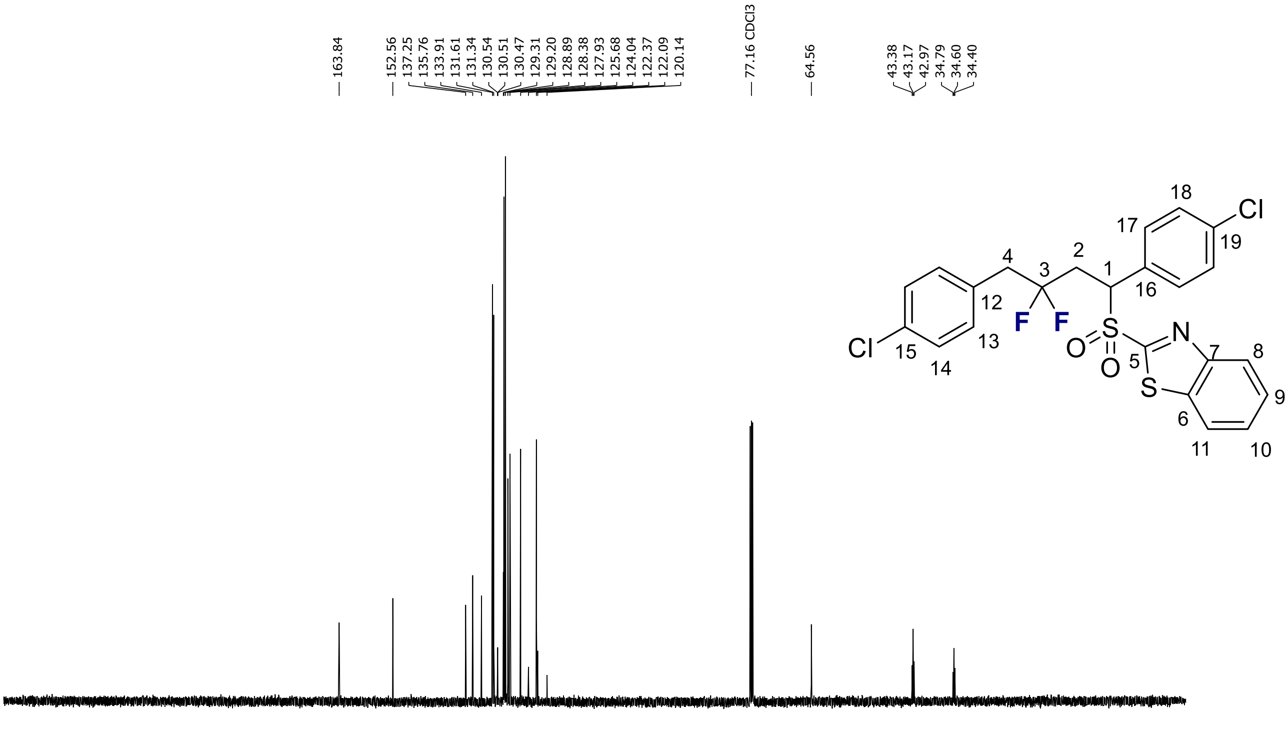

**Supplementary Figure 306.** $^{13}C\{^{1}H\}$ NMR of **8** (126 MHz, 299 K, $CDCl_3$).

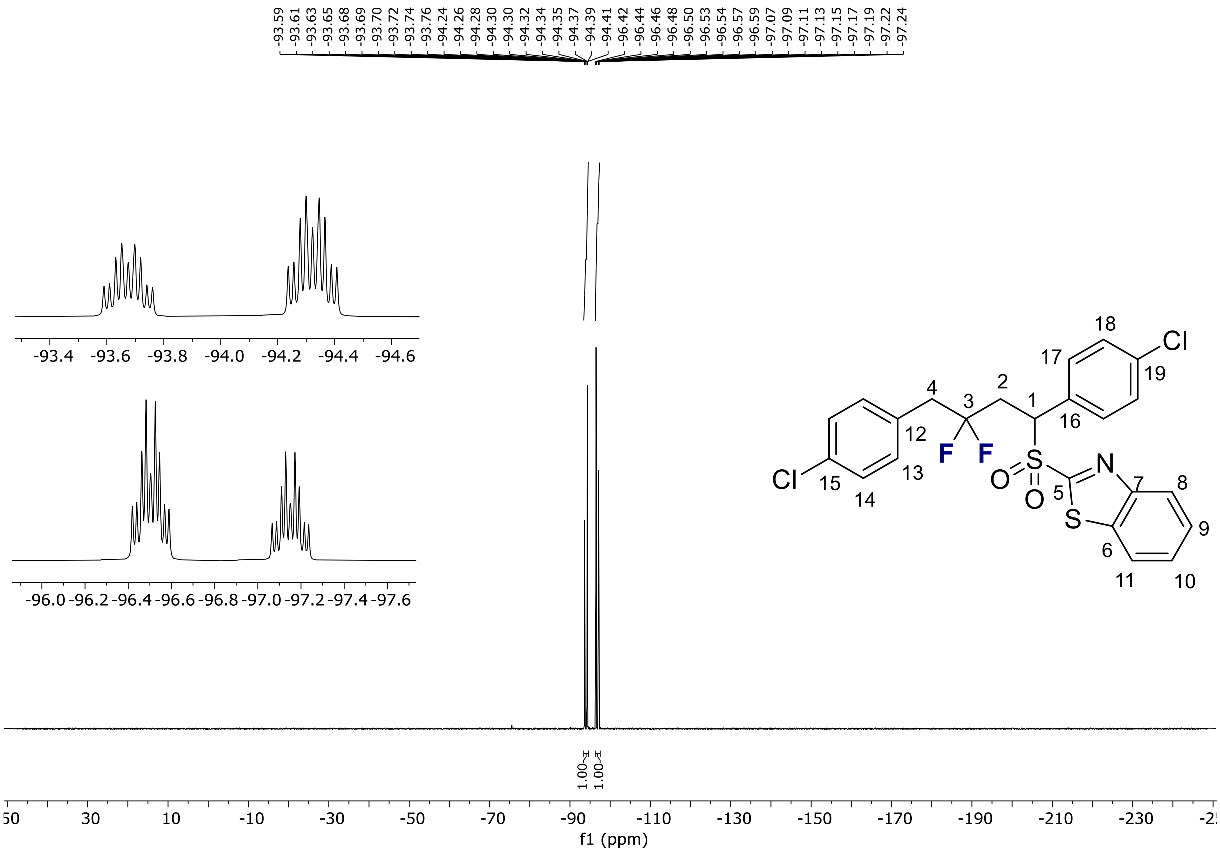

**Supplementary Figure 307.** $^{19}F$ NMR of **8** (376 MHz, 299 K, $CDCl_3$).

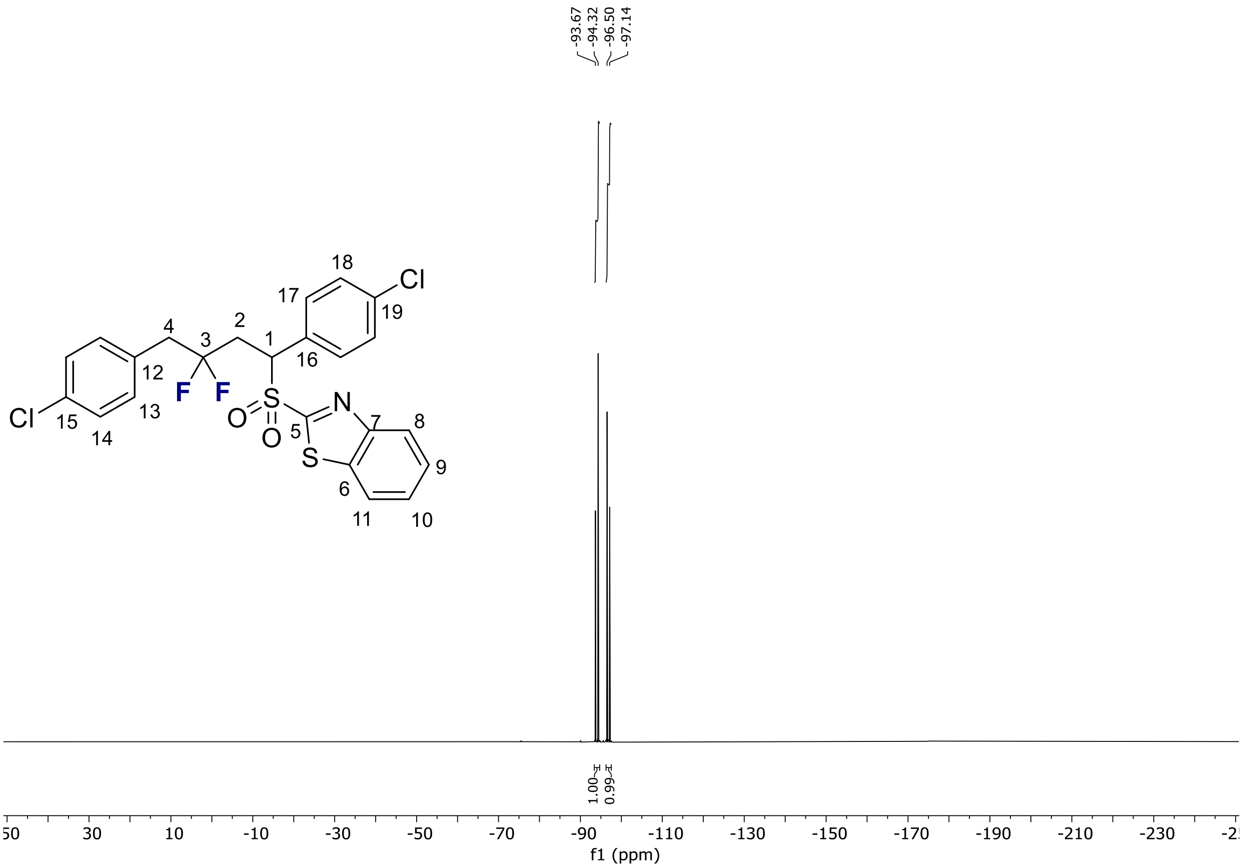

**Supplementary Figure 308.** $^{19}F\{^1H\}$ NMR of **8** (376 MHz, 299 K, $CDCl_3$).

**(1,4-Bis(4-chlorophenyl)-3,3-difluorobutyl)(4-nitrophenyl)sulfane (9)**

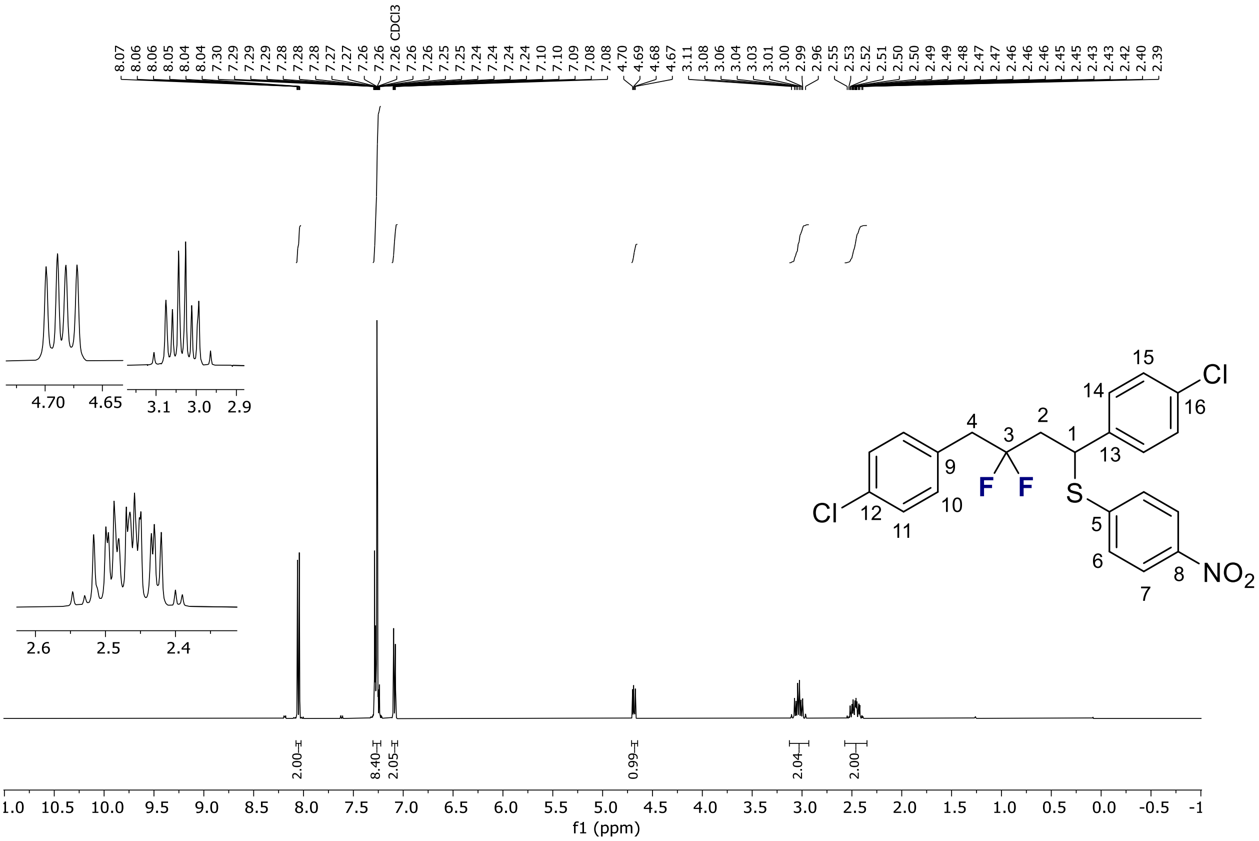


**Supplementary Figure 309.** $^{1}$H NMR of **9** (500 MHz, 299 K, $CDCl_3$).

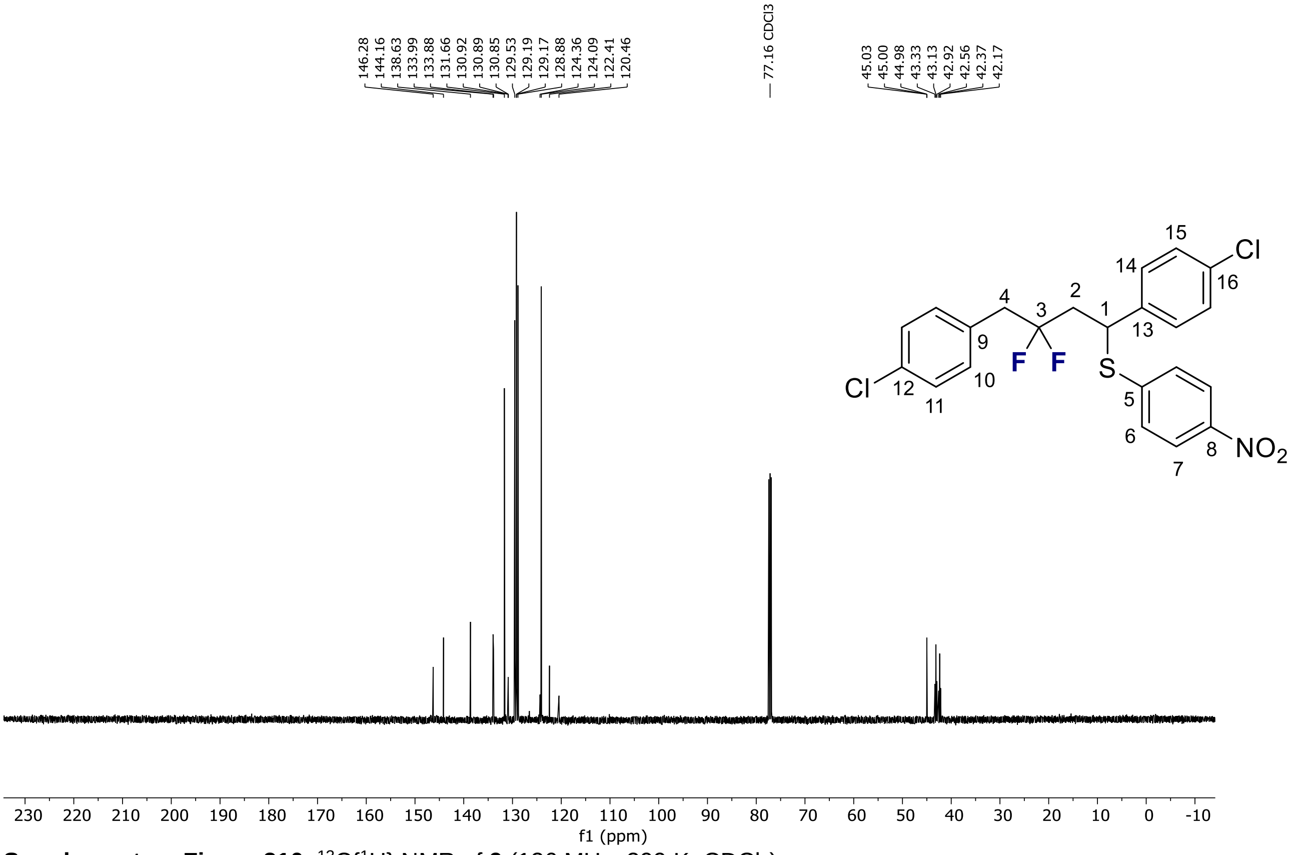


**Supplementary Figure 310.** $^{13}$C{$^{1}$H} NMR of **9** (126 MHz, 299 K, $CDCl_3$).

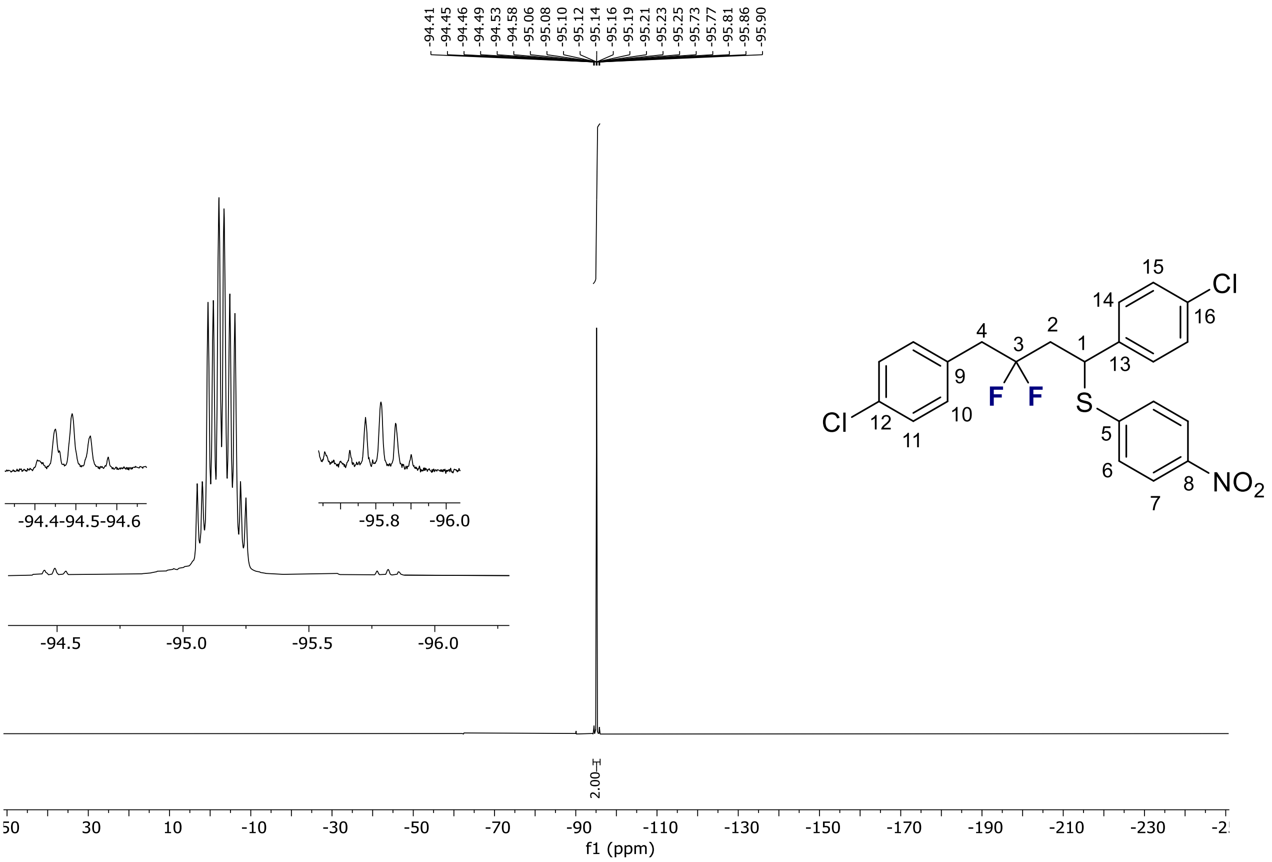

**Supplementary Figure 311.** $^{19}$F NMR of **9** (376 MHz, 299 K, $CDCl_3$).

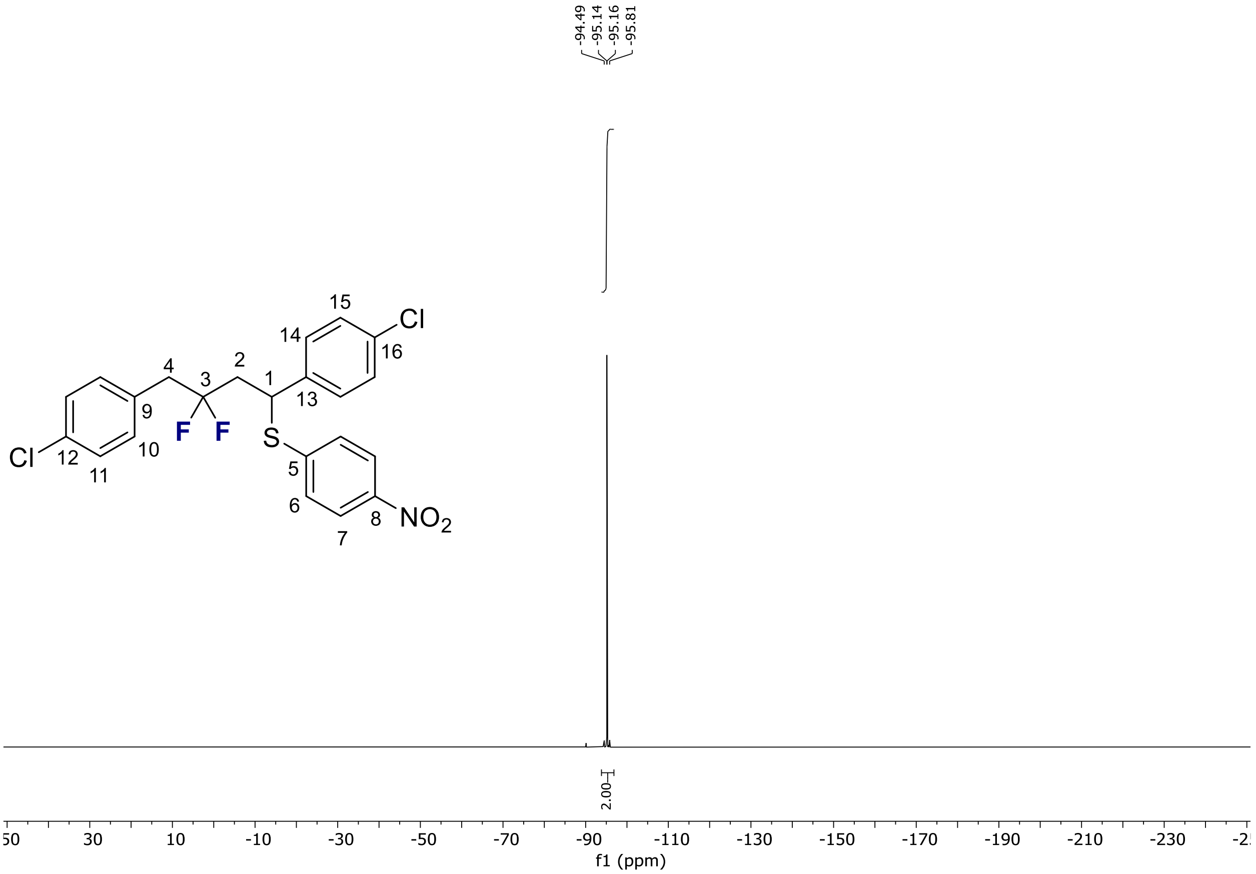

**Supplementary Figure 312.** $^{19}$F{$^1$H} NMR of **9** (376 MHz, 299 K, $CDCl_3$).

## *N*-(1,4-Bis(4-chlorophenyl)-3,3-difluorobutyl)acetamide (10)

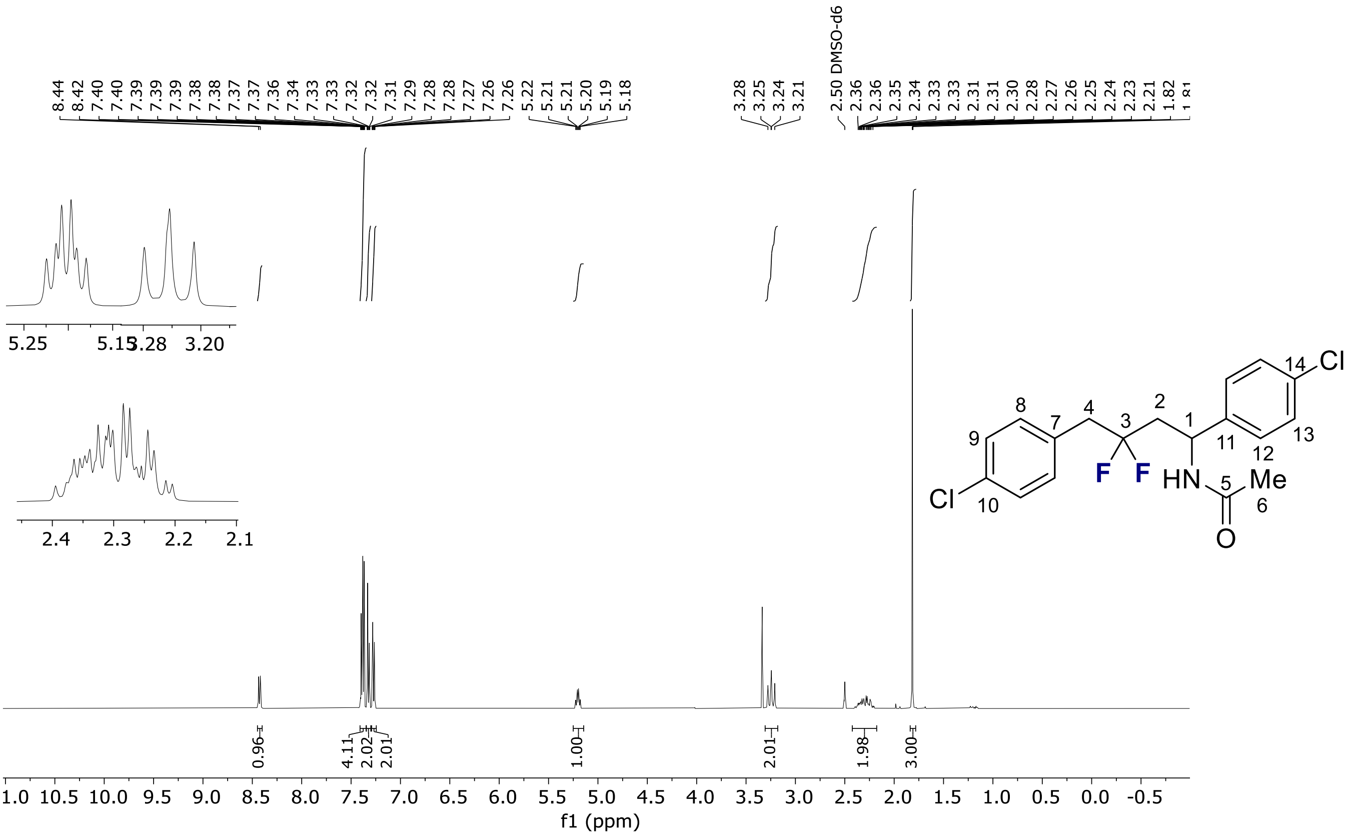


**Supplementary Figure 313.** $^{1}H$ NMR of **10** (500 MHz, 299 K, DMSO-$d_6$).

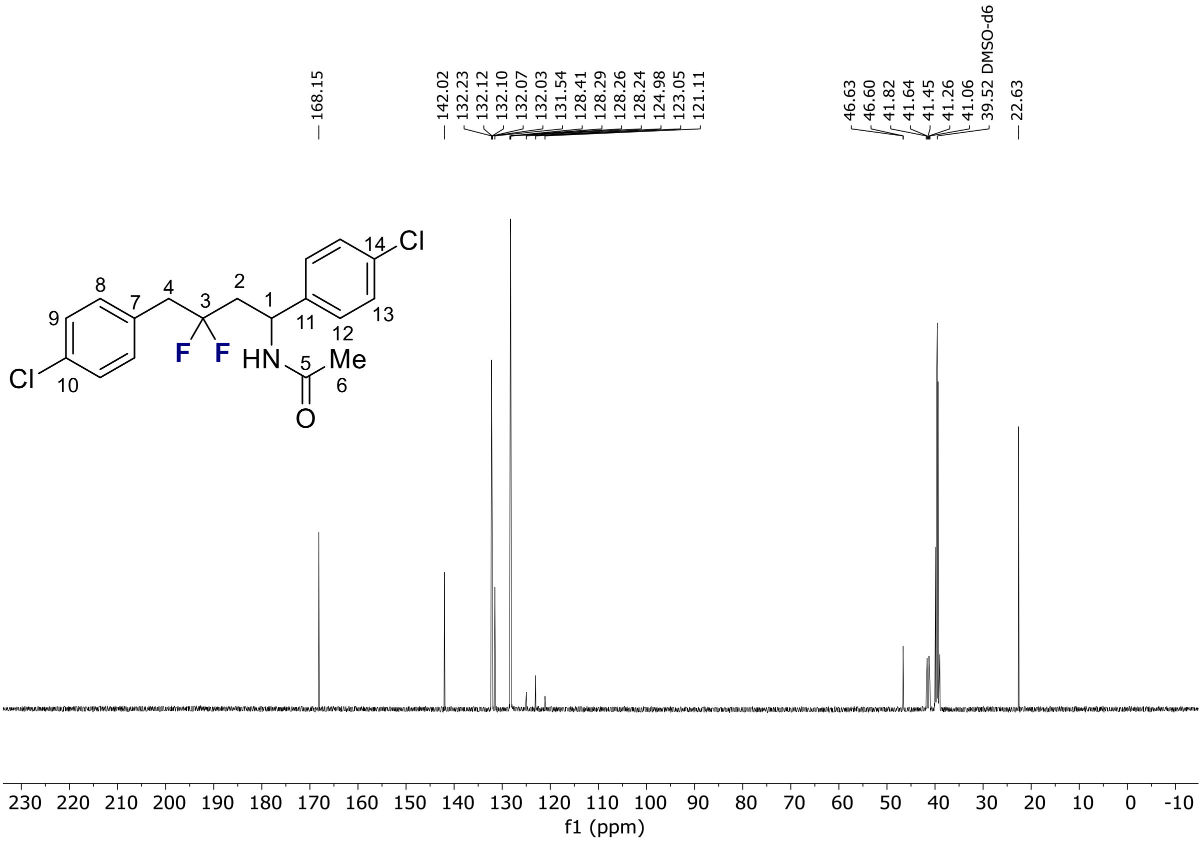


**Supplementary Figure 314.** $^{13}C\{^{1}H\}$ NMR of **10** (126 MHz, 299 K, DMSO-$d_6$).

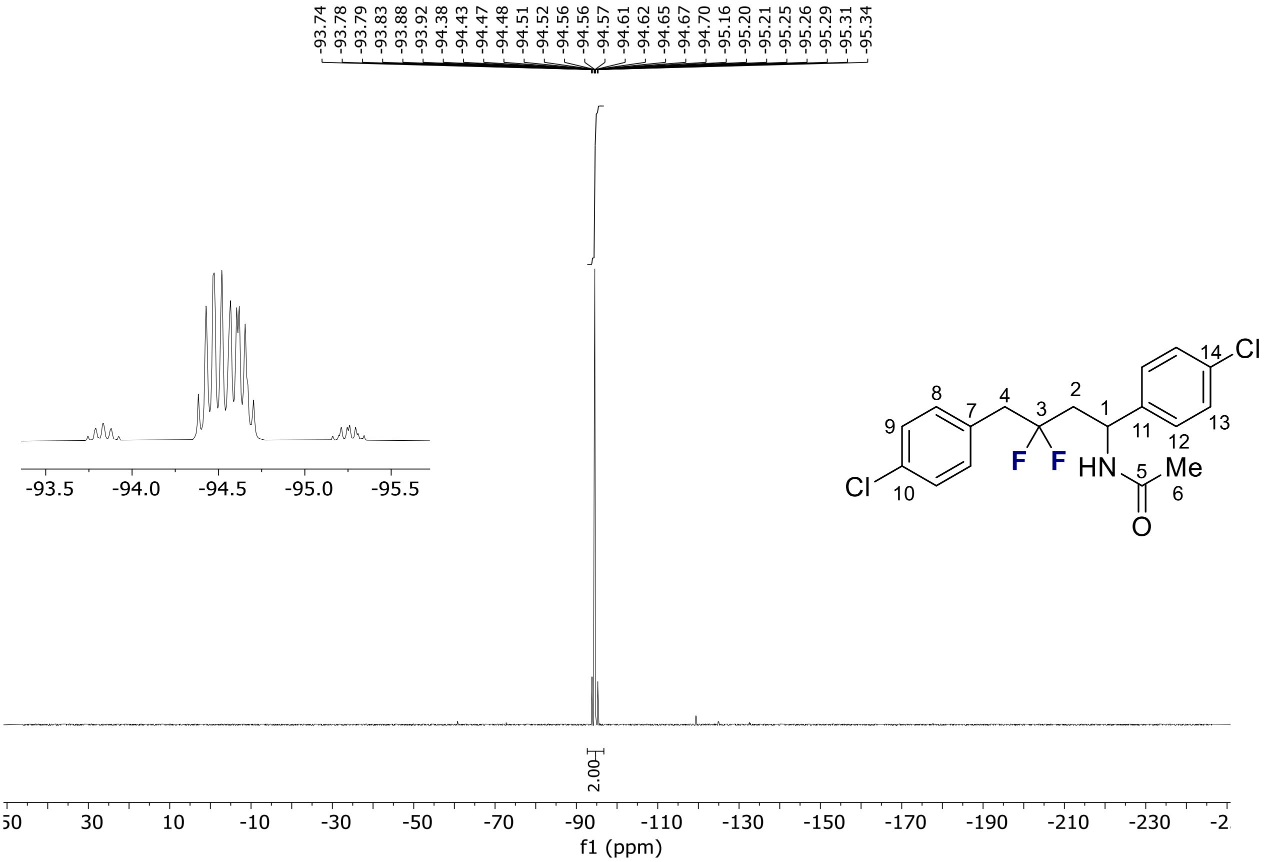


**Supplementary Figure 315.** $^{19}$F NMR of **10** (376 MHz, 299 K, DMSO-$d_6$).

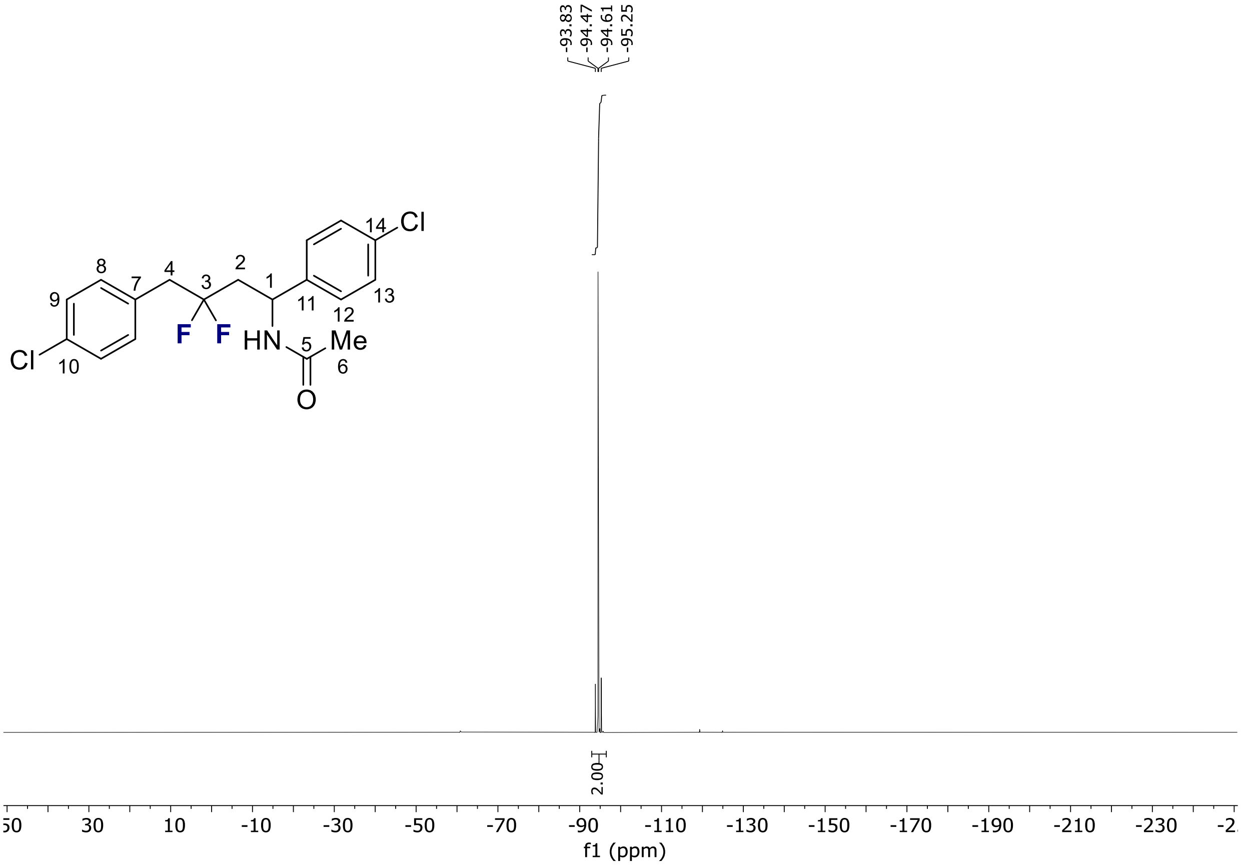


**Supplementary Figure 316.** $^{19}$F{$^1$H} NMR of **10** (376 MHz, 299 K, DMSO-$d_6$).

**(3,3-Difluoro-1,2,3,4-tetrahydronaphthalen-1-yl)phenol (11)**

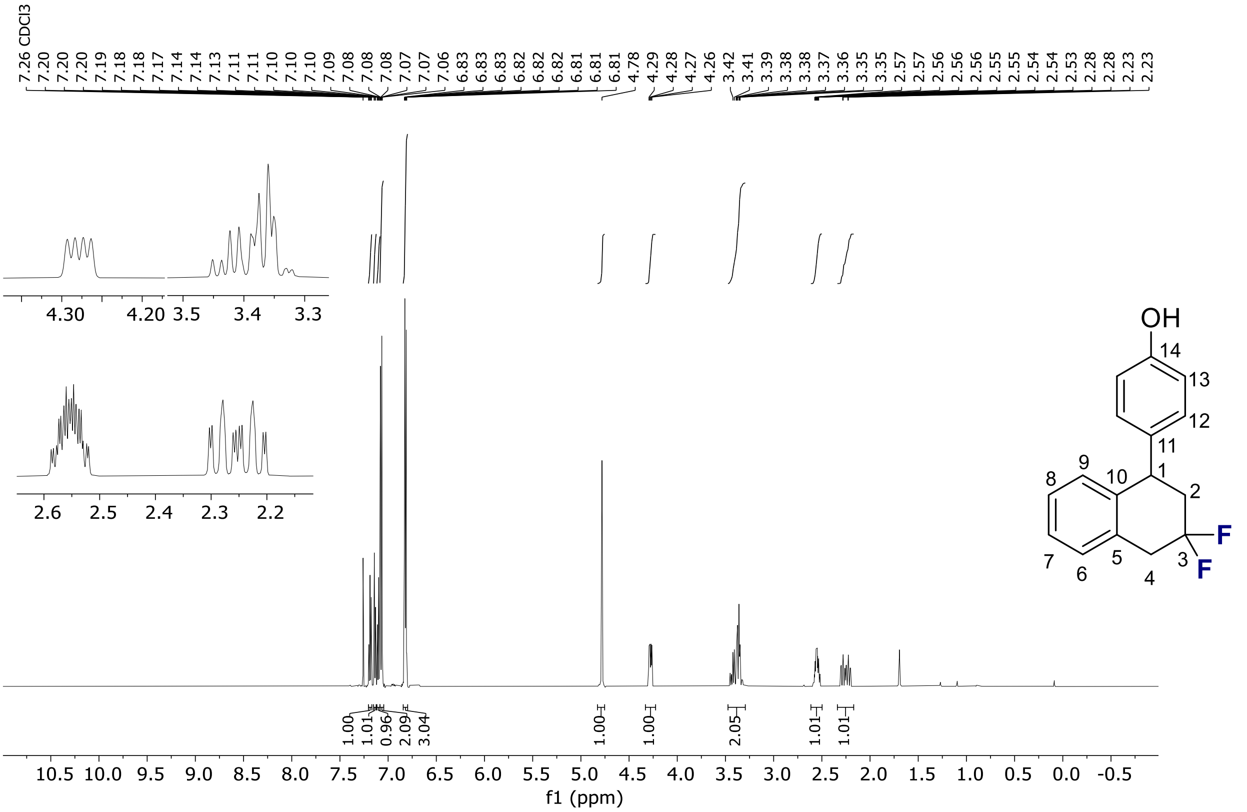


**Supplementary Figure 317.** $^1$H NMR of **11** (599 MHz, 299 K, CDCl$_3$).

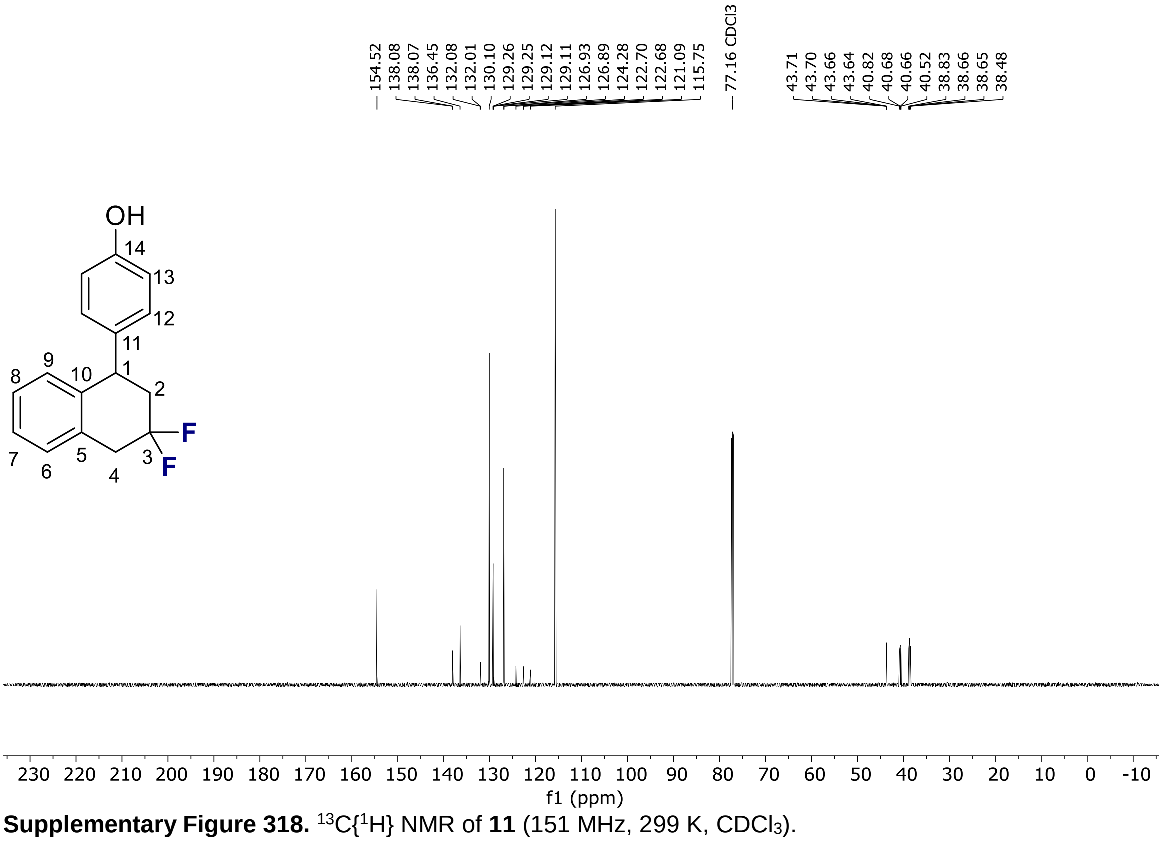


**Supplementary Figure 318.** $^{13}$C{$^1$H} NMR of **11** (151 MHz, 299 K, CDCl$_3$).

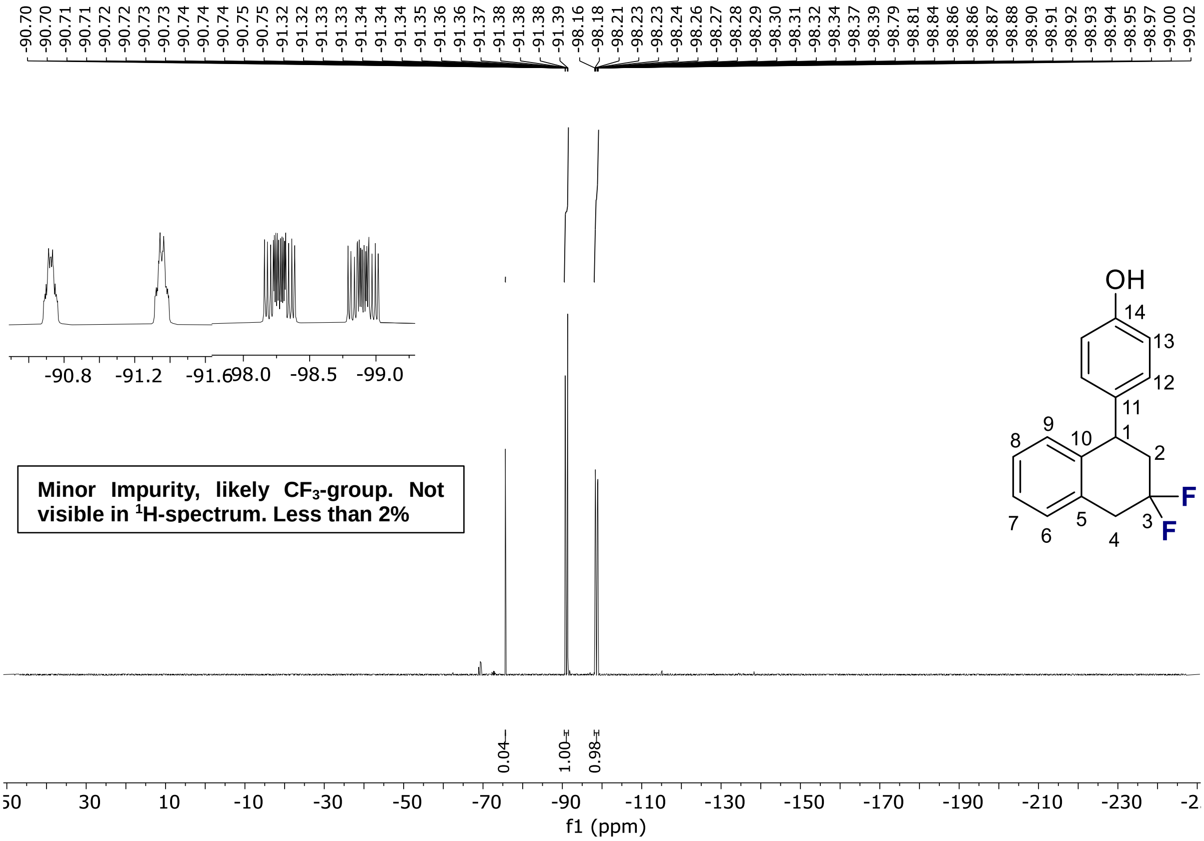


**Supplementary Figure 319.** $^{19}F$ NMR of **11** (376 MHz, 299 K, $CDCl_3$).

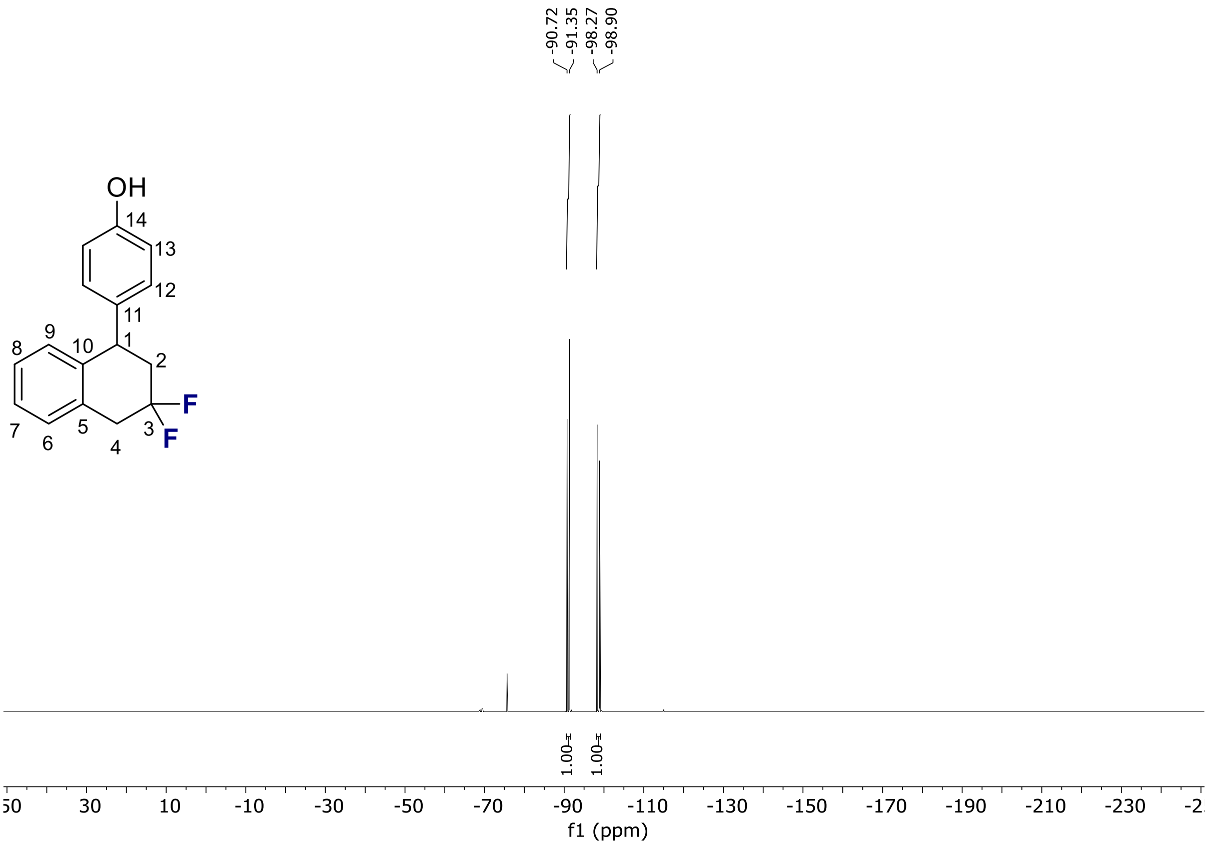


**Supplementary Figure 320.** $^{19}F\{^1H\}$ NMR of **11** (376 MHz, 299 K, $CDCl_3$).

**2-(4-(3,3-Difluoro-1,2,3,4-tetrahydronaphthalen-1-yl)phenoxy)-2-methyl-propanoic acid (12)**

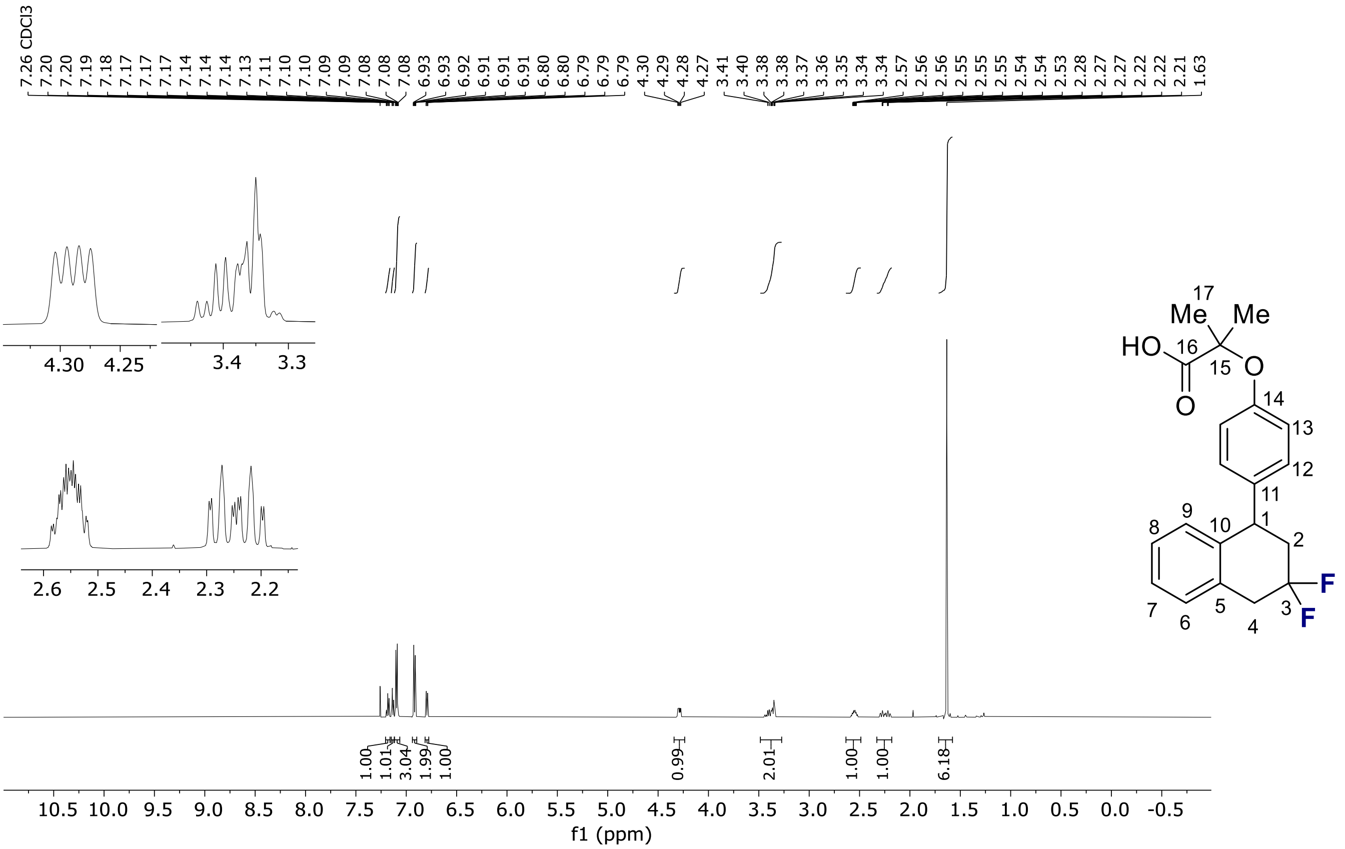

**Supplementary Figure 321.** $^{1}H$ NMR of **12** (599 MHz, 299 K, $CDCl_3$).

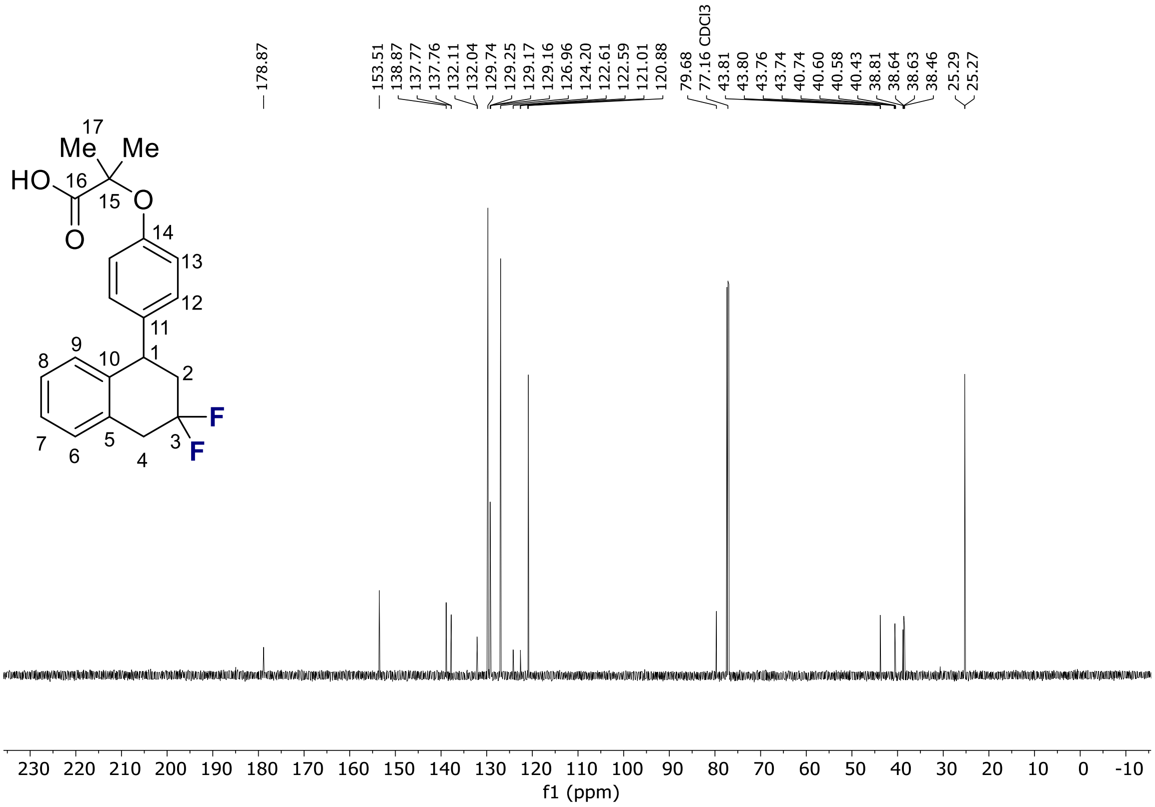

**Supplementary Figure 322.** $^{13}C\{^{1}H\}$ NMR of **12** (151 MHz, 299 K, $CDCl_3$).

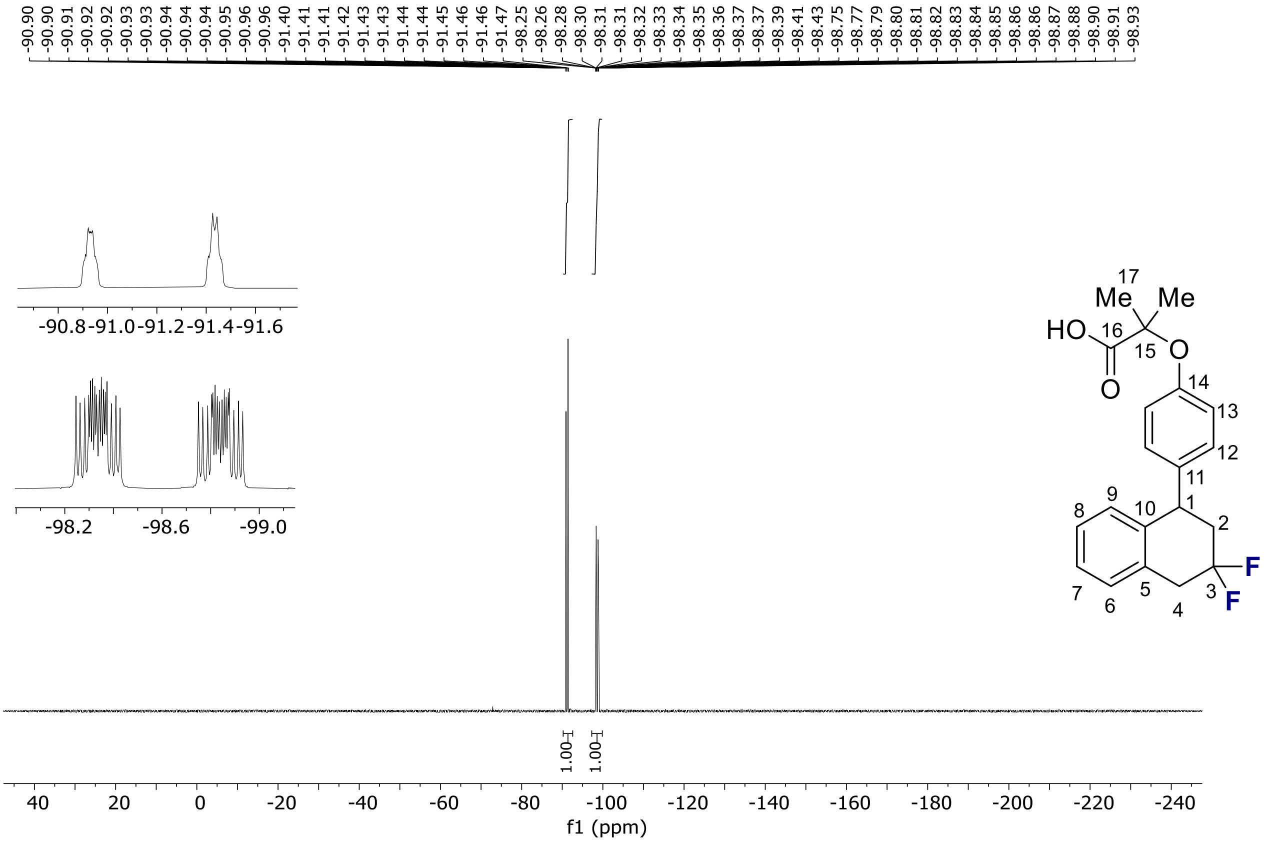


**Supplementary Figure 323.** $^{19}F$ NMR of **12** (564 MHz, 299 K, $CDCl_3$).

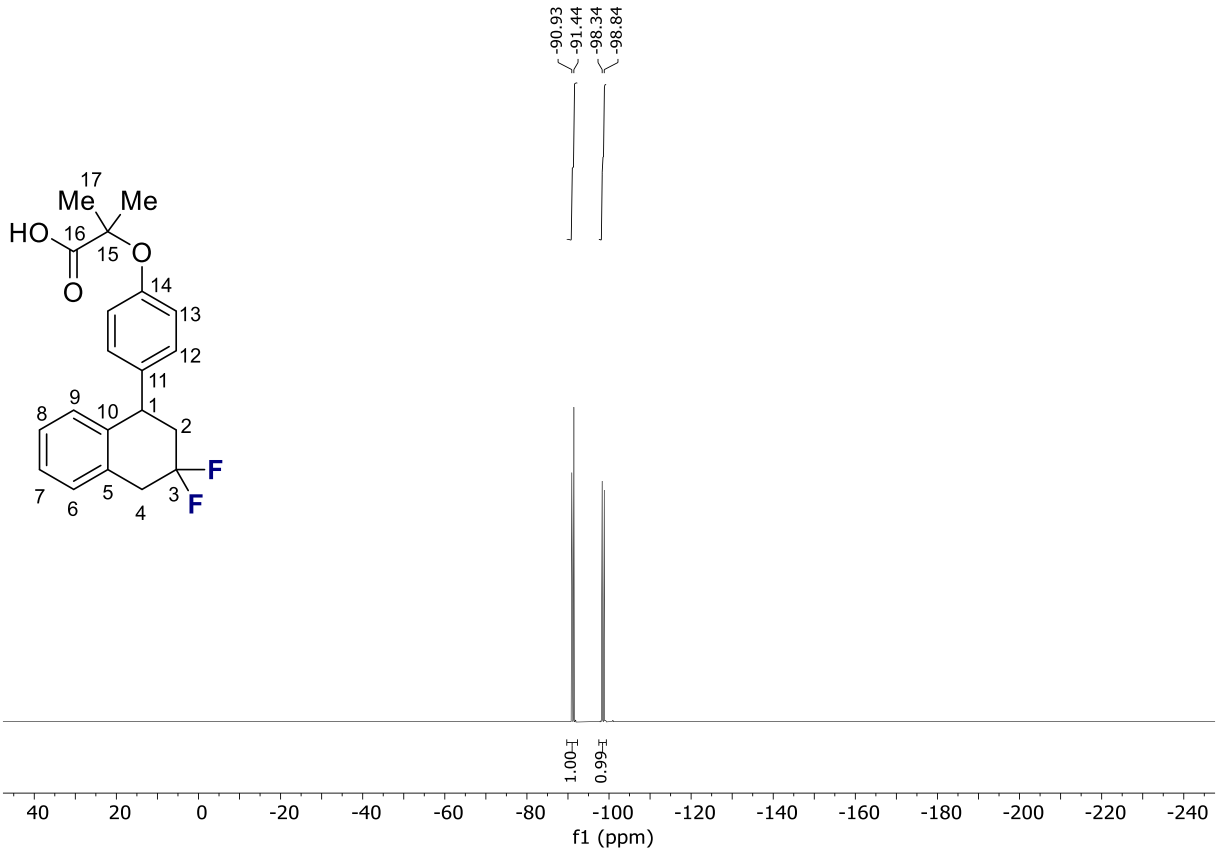


**Supplementary Figure 324.** $^{19}F\{^{1}H\}$ NMR of **12** (564 MHz, 299 K, $CDCl_3$).

## ((4-(3,3-Difluoro-1,2,3,4-tetrahydronaphthalen-1-yl)phenyl)ethynyl)triisopropylsilane (13)

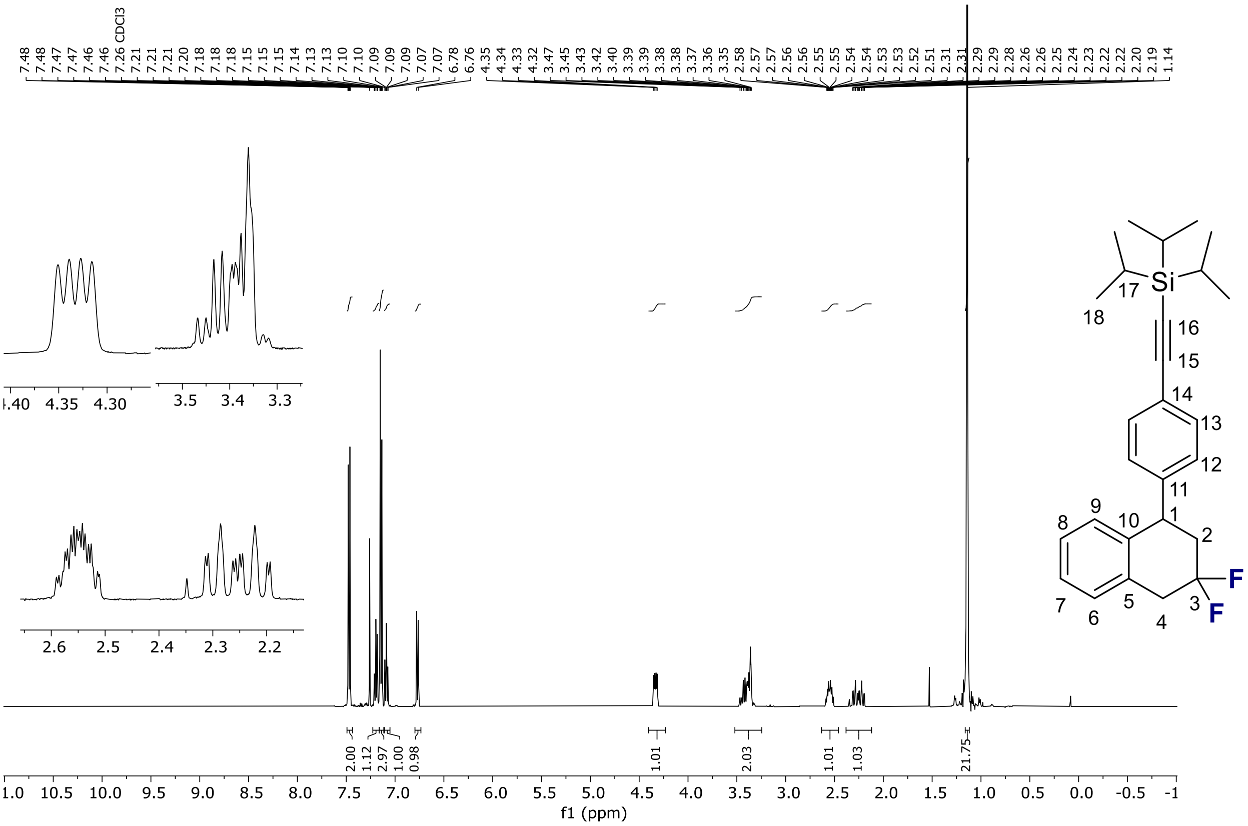


**Supplementary Figure 325.** $^{1}$H NMR of **13** (500 MHz, 299 K, $CDCl_3$).

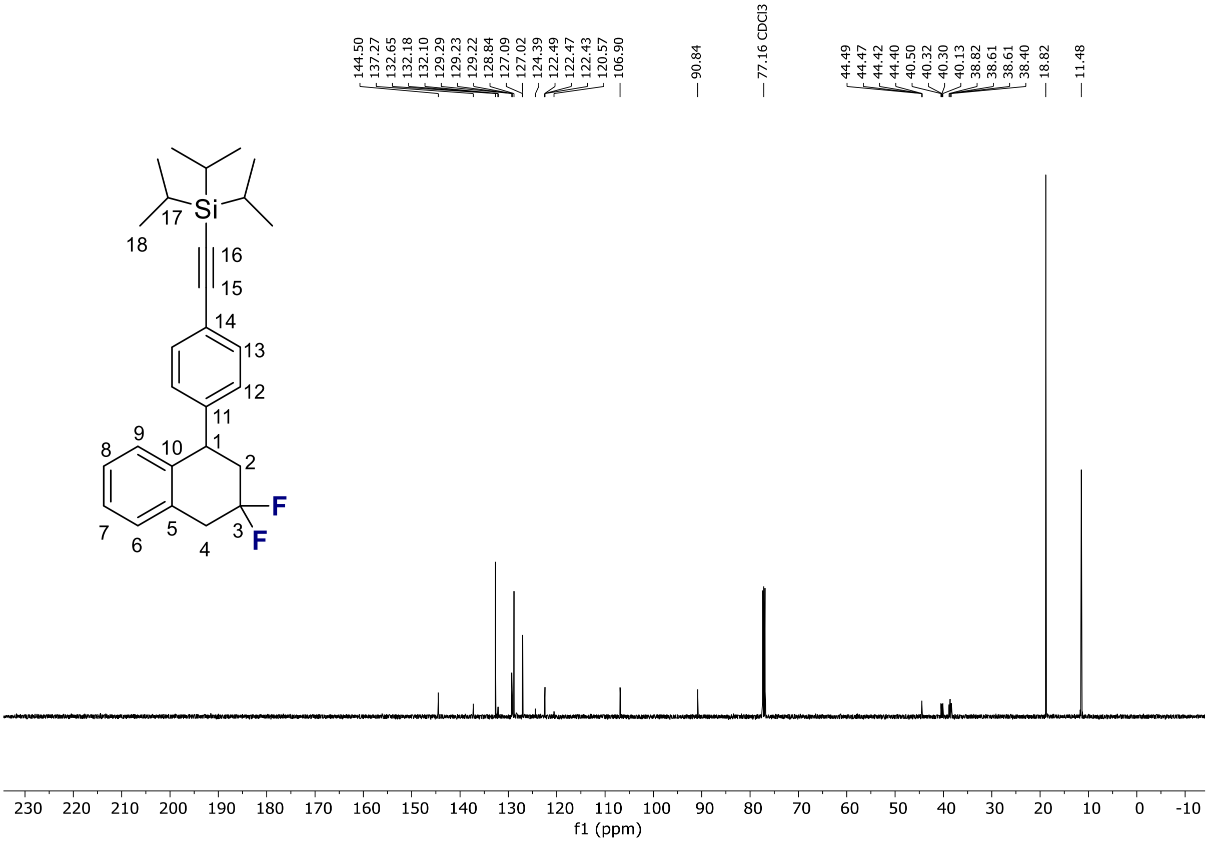


**Supplementary Figure 326.** $^{13}$C{$^{1}$H} NMR of **13** (126 MHz, 299 K, $CDCl_3$).

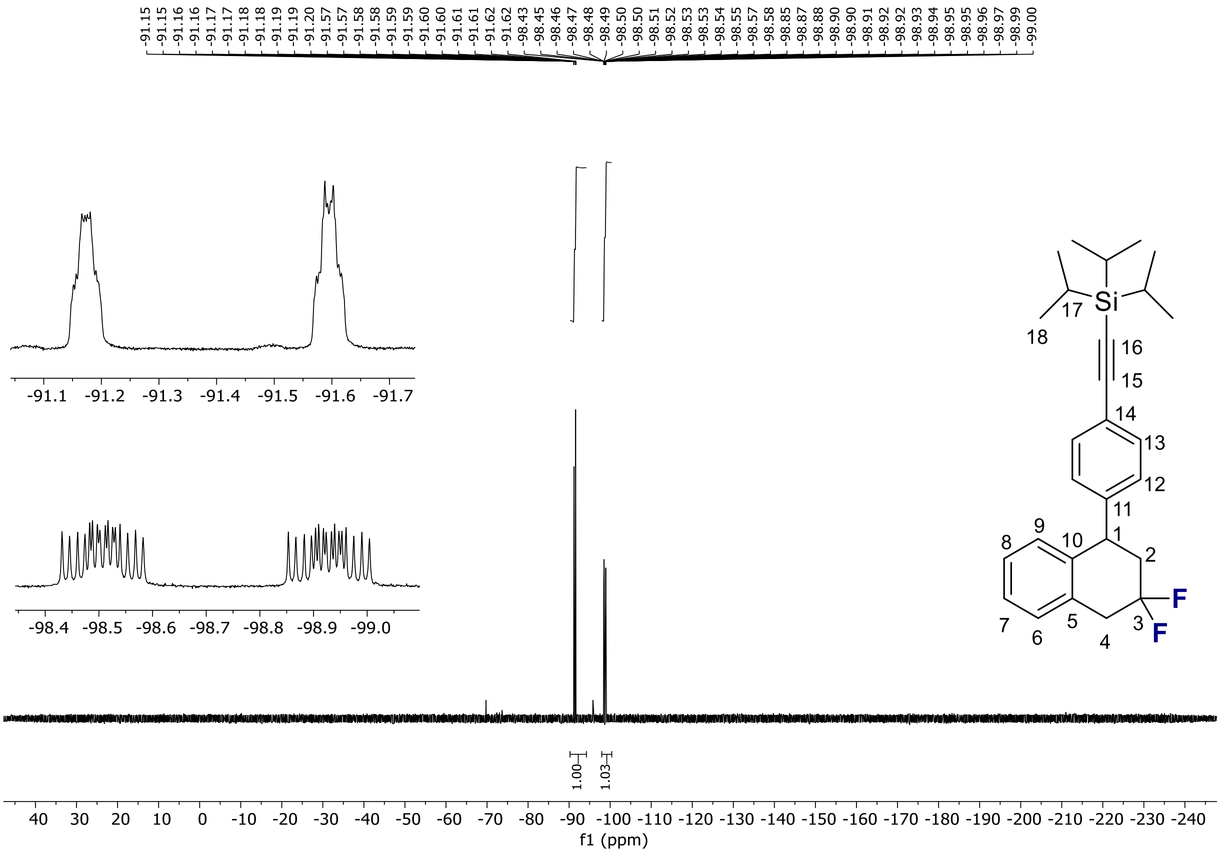


**Supplementary Figure 327.** $^{19}$F NMR of **13** (470 MHz, 299 K, $CDCl_3$).

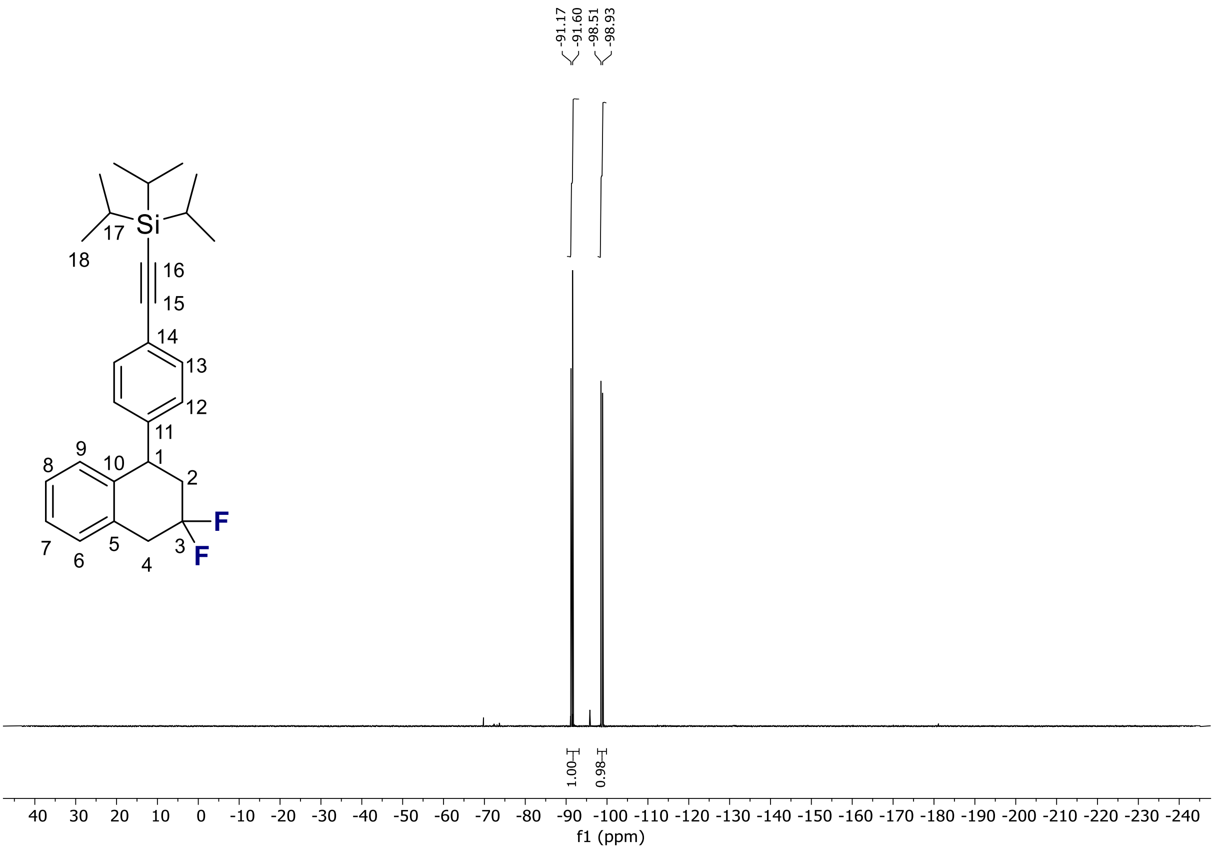


**Supplementary Figure 328.** $^{19}$F{$^1$H} NMR of **13** (470 MHz, 299 K, $CDCl_3$).

## 5-(4-(3,3-Difluoro-1,2,3,4-tetrahydronaphthalen-1-yl)phenyl)benzo[d][1,3]dioxole (14)

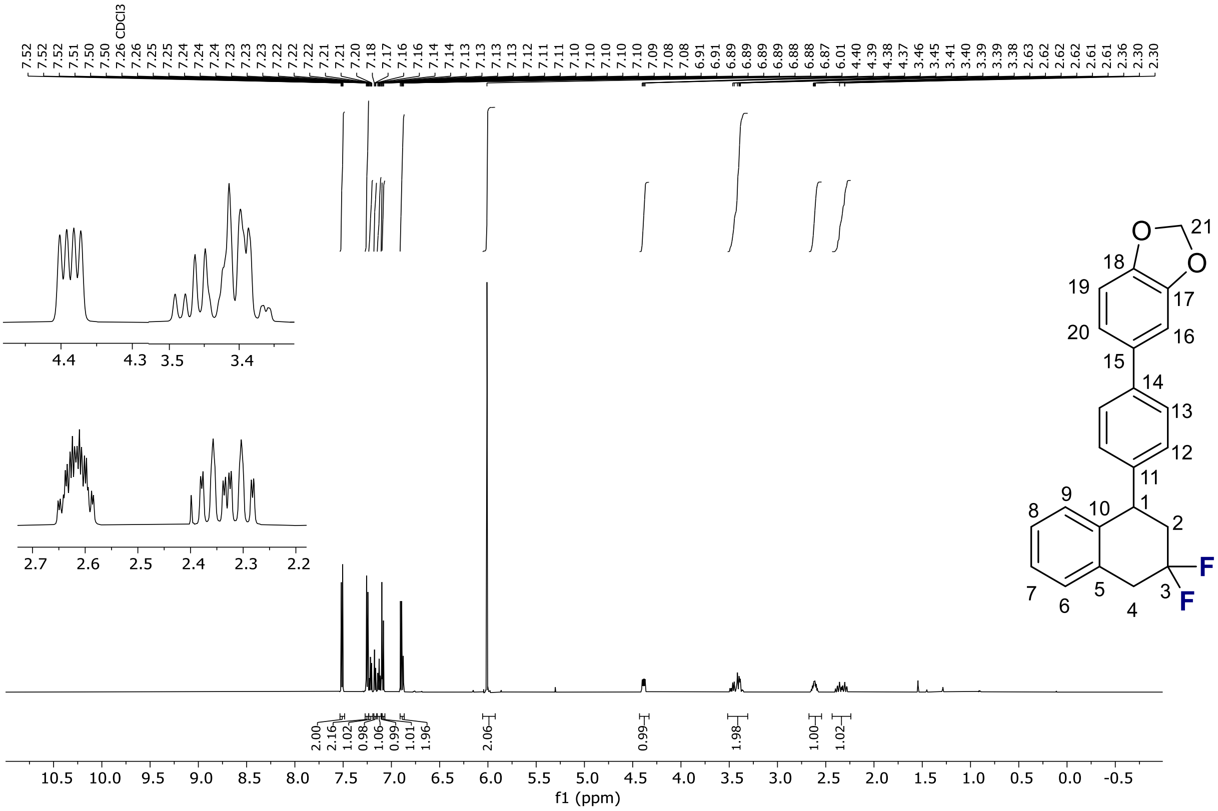


**Supplementary Figure 329.** $^{1}$H NMR of **14** (599 MHz, 299 K, $CDCl_3$).

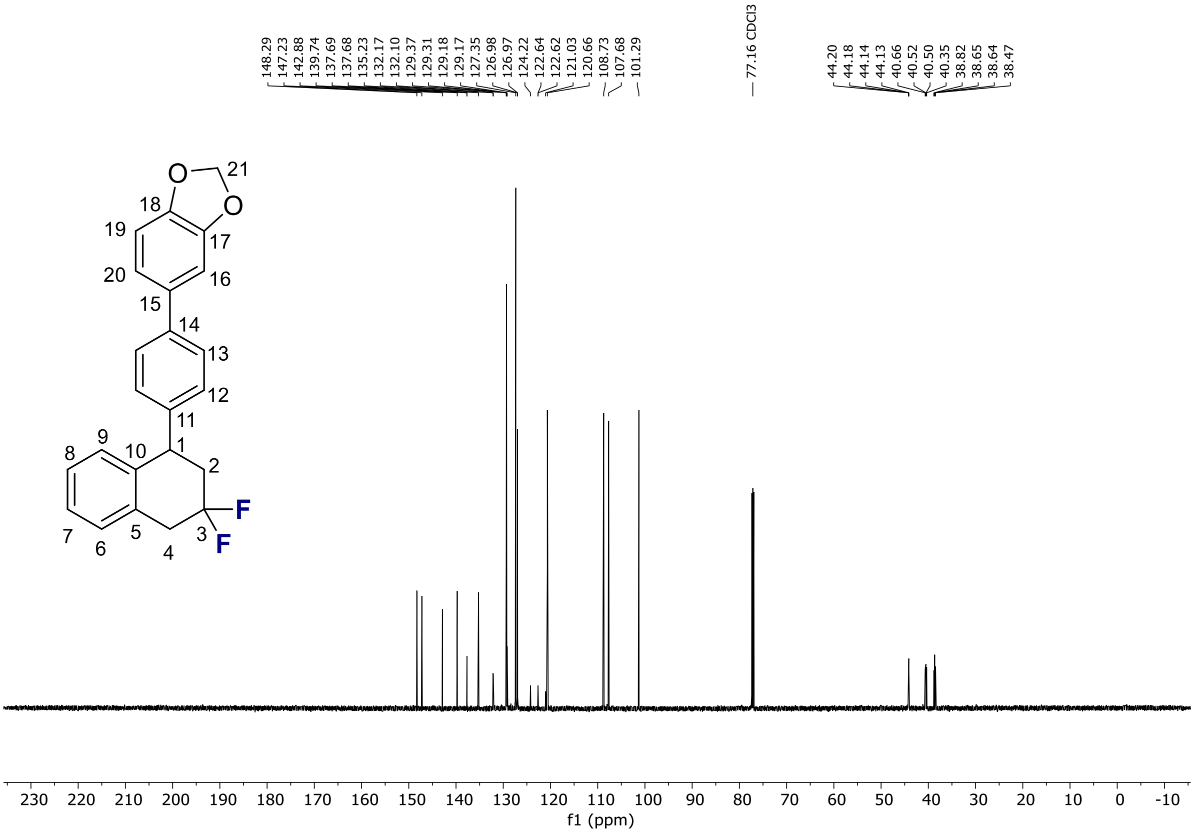


**Supplementary Figure 330.** $^{13}C\{^{1}H\}$ NMR of **14** (151 MHz, 299 K, $CDCl_3$).

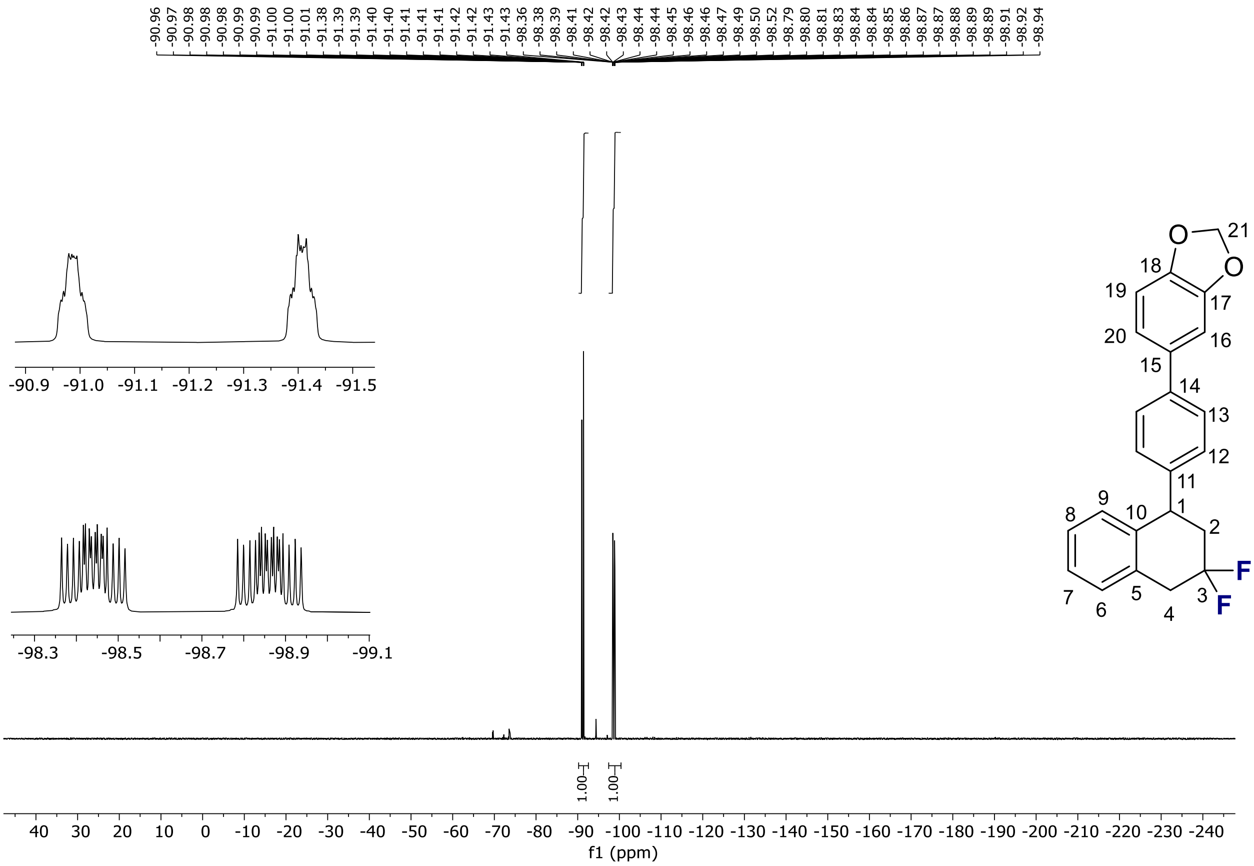


**Supplementary Figure 331.** $^{19}$F NMR of **14** (564 MHz, 299 K, $CDCl_3$).

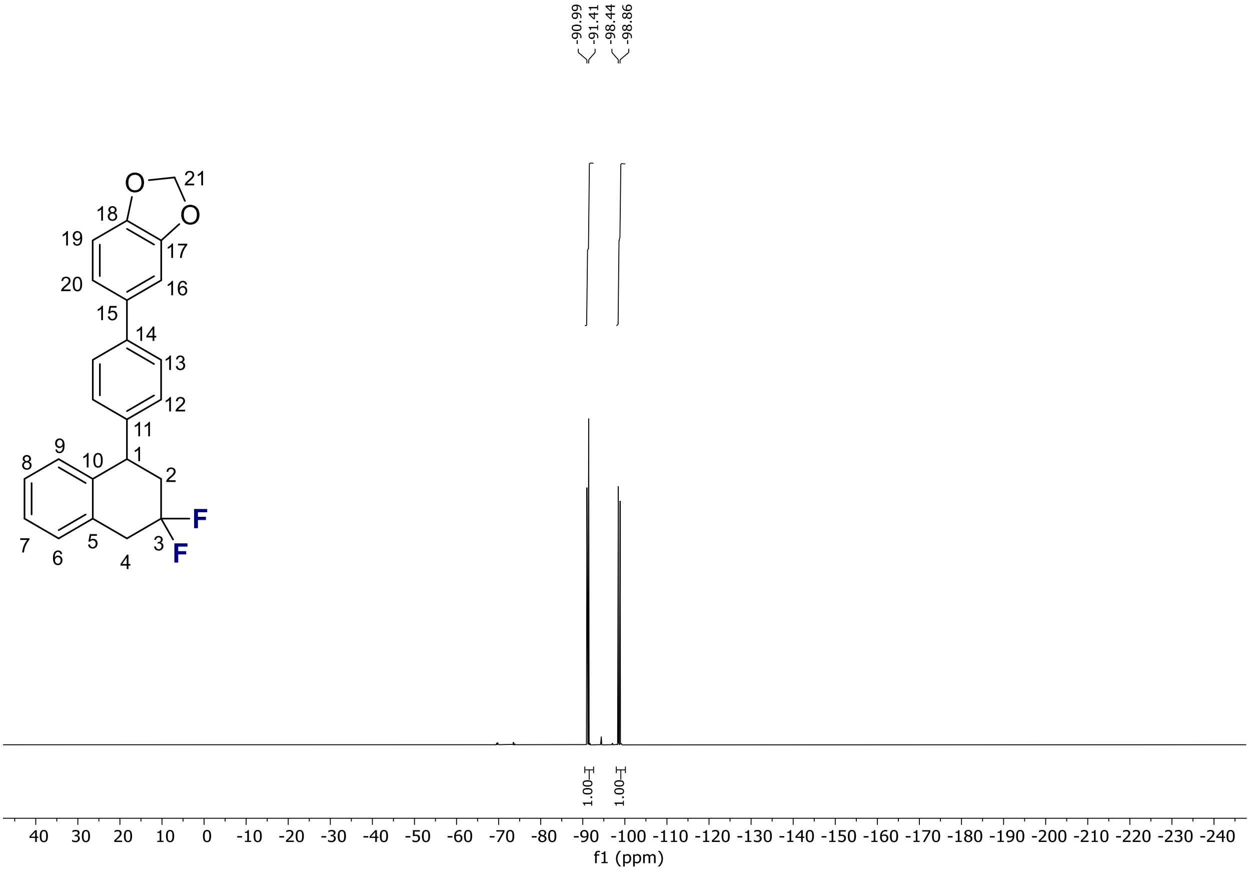


**Supplementary Figure 332.** $^{19}$F{$^1$H} NMR of **14** (564 MHz, 299 K, $CDCl_3$).

## 1-(4-(3,3-Difluoro-1,2,3,4-tetrahydronaphthalen-1-yl)phenyl)-1H-indole (15)

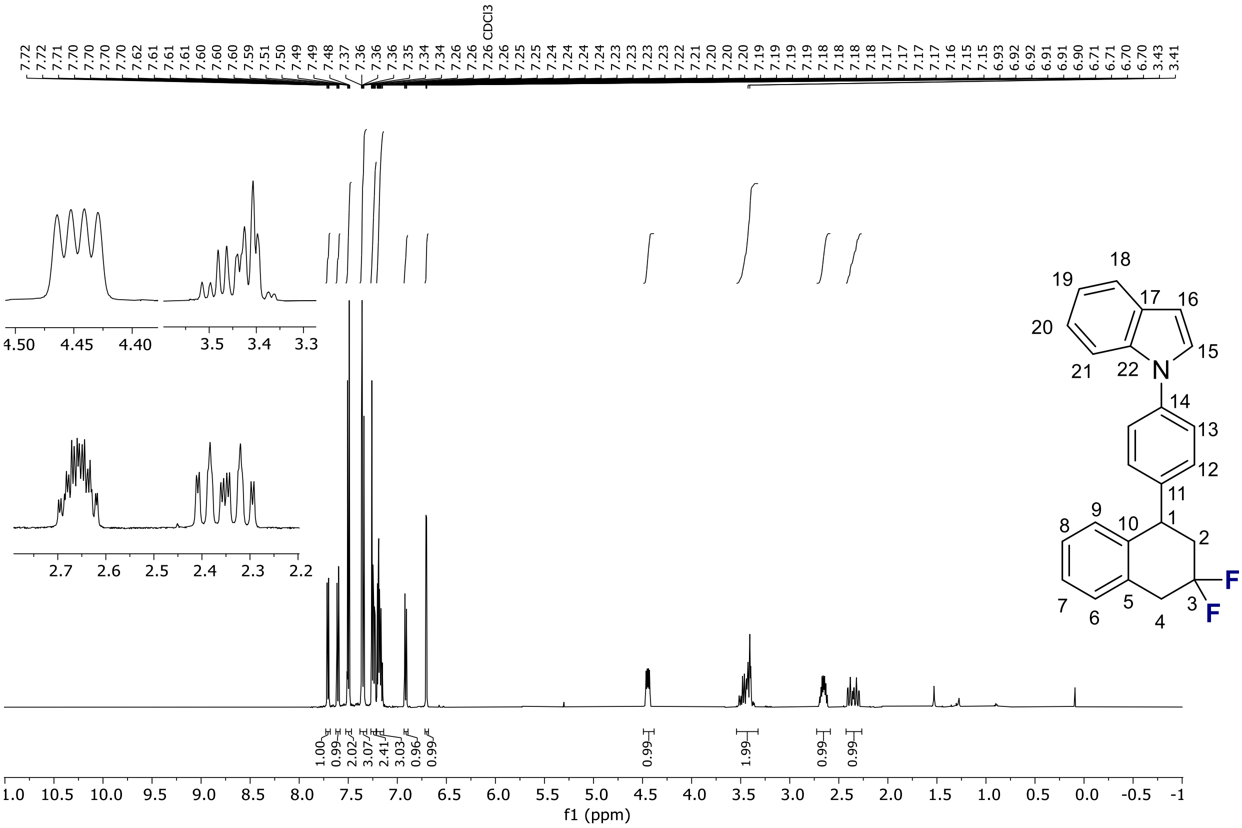


**Supplementary Figure 333.** $^{1}$H NMR of **15** (500 MHz, 299 K, $CDCl_3$).

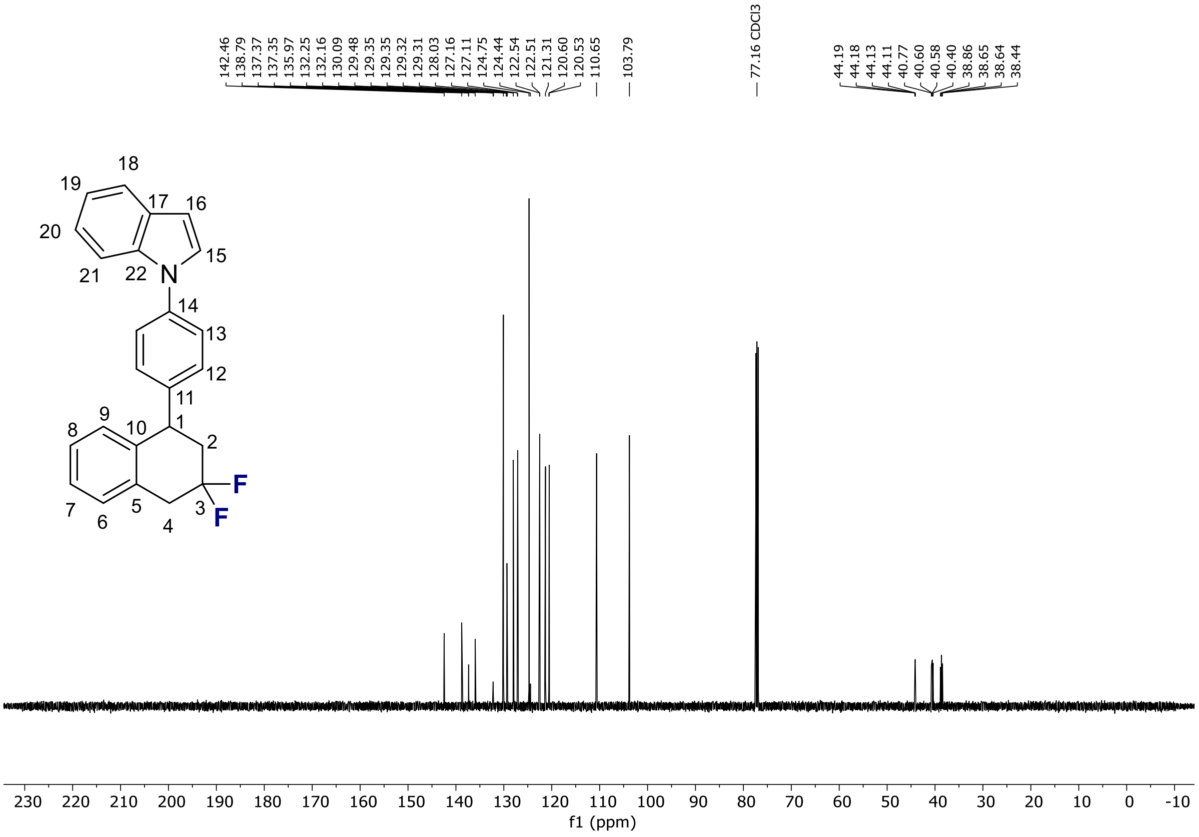


**Supplementary Figure 334.** $^{13}C\{^{1}H\}$ NMR of **15** (126 MHz, 299 K, $CDCl_3$).

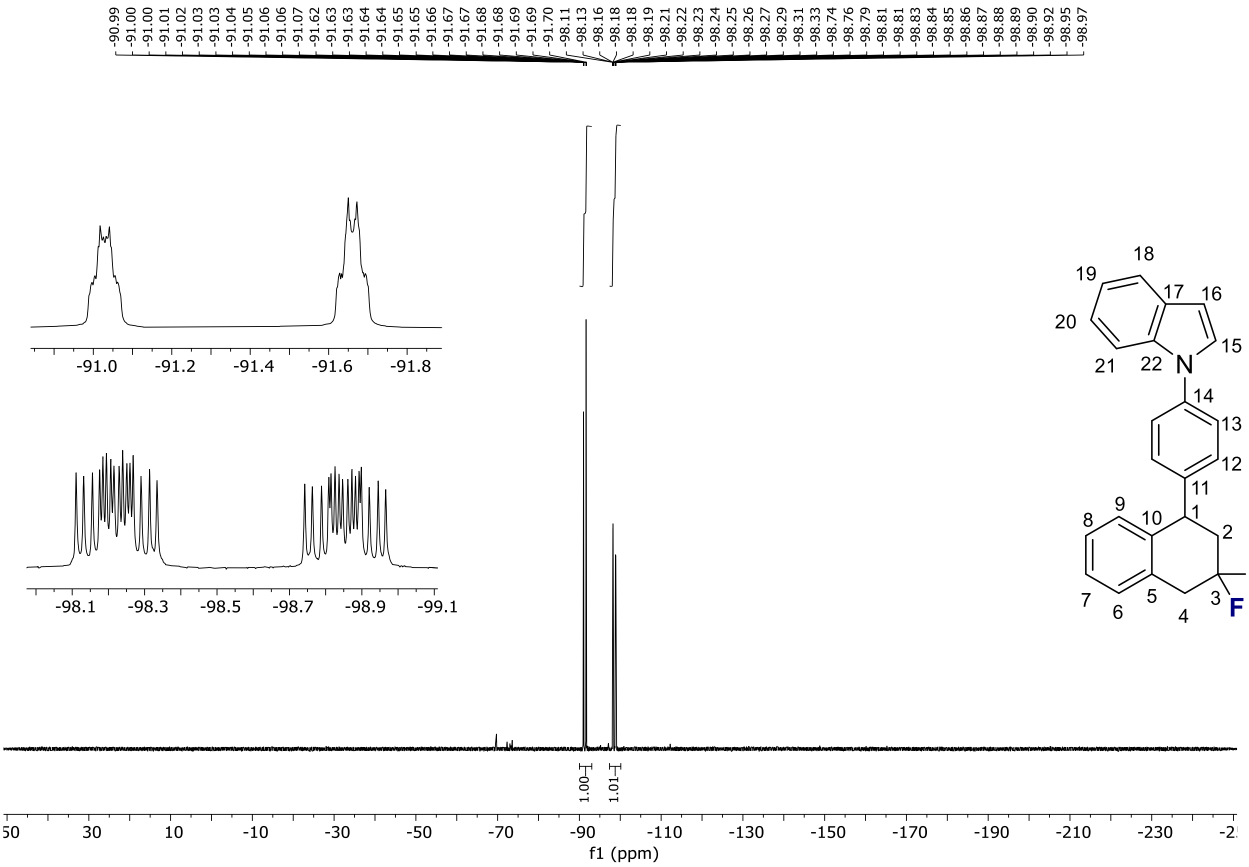


**Supplementary Figure 335.** $^{19}F$ NMR of **15** (376 MHz, 299 K, $CDCl_3$).

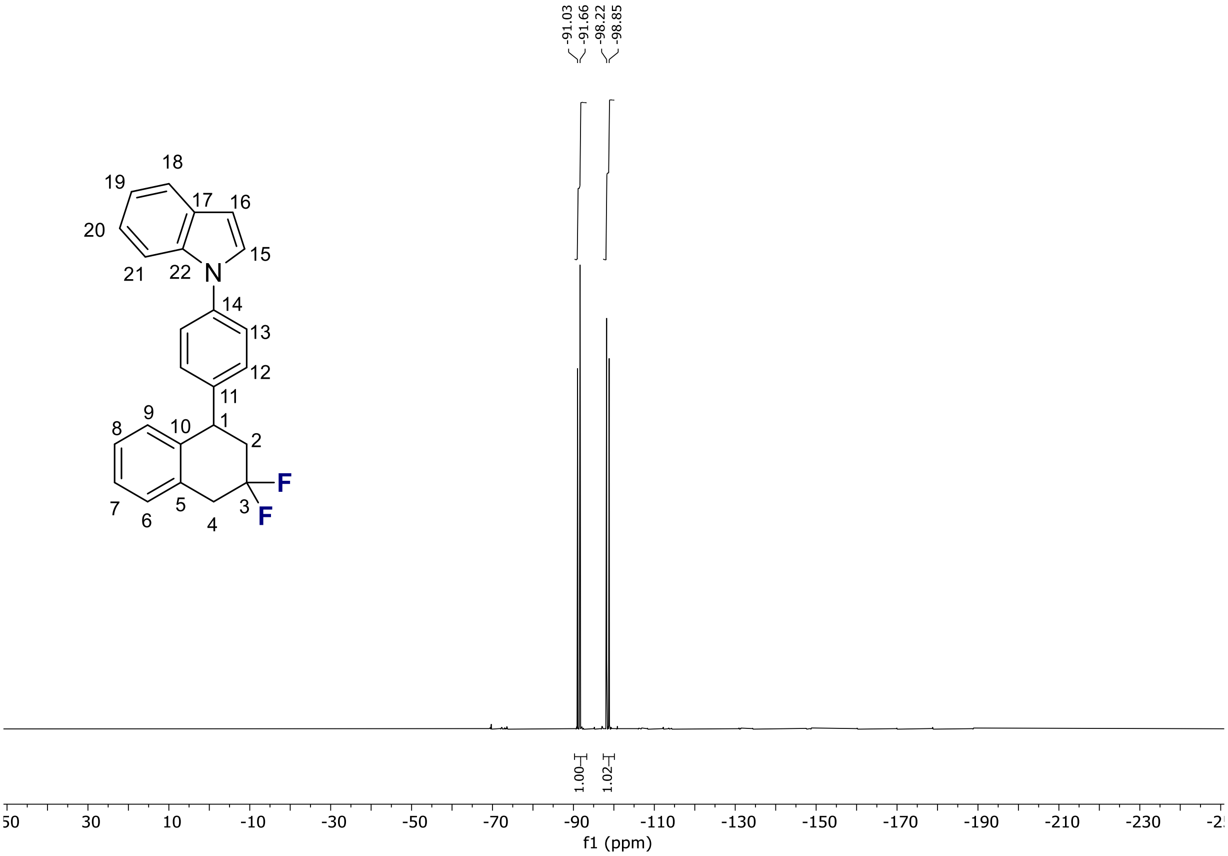


**Supplementary Figure 336.** $^{19}F\{^1H\}$ NMR of **15** (376 MHz, 299 K, $CDCl_3$).

# 2. Supplementary References


[1] Sietmann, J., Ong, M., Mück-Lichtenfeld, C., Daniliuc, C. G. & Wiest, J. M. Desymmetrization of Prochiral Cyclobutanones via Nitrogen Insertion: A Concise Route to Chiral γ-Lactams. *Angew. Chem. Int. Ed. Engl.* **60**, 9719–9723 (2021).

[2] Meyer, S. et al. A chiral pentafluorinated isopropyl group via iodine (I)/(III) catalysis. *Angew. Chem. Int. Ed. Engl.* **60**, 6430–6434 (2021).

[3] Britten, T. K., & McLaughlin, M. G. Brønsted Acid Catalyzed Peterson Olefinations. *J. Org. Chem.* **85**, 301–305 (2020).

[4] Scheidt, F. et al. Enantioselective, catalytic vicinal difluorination of alkenes. *Angew. Chem. Int. Ed. Engl.* **57**, 16431–16435 (2018).

[5] Guo, J. et al. Furfuryl Cation Induced Cascade Formal [3+ 2] Cycloaddition/Double Ring-Opening/Chlorination: An Approach to Chlorine-Containing Complex Triazoles. *Org. Lett.* **20**, 7410–7414 (2018).

[6] Sevov, C. S. & Hartwig, J. F. Iridium-catalyzed oxidative olefination of furans with unactivated alkenes. *J. Am. Chem. Soc.* **136**, 10625-10631 (2014).

[7] Häfliger, J., Livingstone, K., Daniliuc, C. G. & Gilmour, R. Difluorination of α-(bromomethyl) styrenes via I (I)/I (III) catalysis: facile access to electrophilic linchpins for drug discovery. *Chem. Sci.* **12**, 6148–6152 (2021).

[8] Matsuda, T. & Yuihara, I. A rhodium(I)-catalysed formal intramolecular C–C/C–H bond metathesis. *Chem. Commun.* **51**, 7393–7396 (2015).

[9] Yu, H. et al. Conformational Control of a Metallo-Supramolecular Cage via the Dissymmetrical Modulation of Ligands. *Angew. Chem. Int. Ed. Engl.* **60**, 26523-26527 (2021).

[10] Gan, Y., Zhang, N., Huang, S. & Liu, Y. Nickel-Catalyzed Cross-Coupling of Aryl Pivalates with Cyclobutanols Involving C—O and C—C Bond Cleavage. *Chinese J. Chem.* **38**, 1686–1690 (2020).

[11] Matsumura, S., Maeda, Y., Nishimura, T. & Uemura, S. Palladium-Catalyzed Asymmetric Arylation, Vinylation, and Allenylation of tert-Cyclobutanols via Enantioselective C−C Bond Cleavage. *J. Am. Chem. Soc.* **125**, 8862–8869 (2003).

[12] Fyfe, M. C. T. & Teobald, B. J. (Sitryx Therapeutics LTD), Novel Compounds. WO-A1 90724 (2022).

[13] Adcock, A., Das Gupta, B. & Khor, T.-C. Substituent effects by $^{19}$F nuclear magnetic resonance: Polar and π-electron effects. *Aust. J. Chem*. **29**, 2571-2581 (1976).

[14] Banerjee, A. & Sudan Maji, M. A. Brønsted Acid Catalyzed Cascade Reaction for the Conversion of Indoles to α-(3-Indolyl) Ketones by Using 2-Benzyloxy Aldehydes. *Chem. Eur. J.* **25**, 11521-11527 (2019).

[15] Lucarini, S., Bedini, A., Spadoni, G. & Piersanti, G. An improved synthesis of cis-4-phenyl-2-propionamidotetralin (4-P-PDOT): a selective MT2melatonin receptor antagonist. *Org. Biomol. Chem.* **6**, 147-150 (2008).

[16] Ghosh, A. K. & Zajc, B. High-yield synthesis of fluorinated benzothiazolyl sulfones: General synthons for fluoro-Julia olefinations. *Org. Lett.* **8**, 1553–1556 (2006).

[17] Ohgiya, T. & Nishiyama, S. A simple deprotection of triflate esters of phenol derivatives. *Tet. Lett.* **45**, 6317–6320 (2004).

[18] Bering, L., Jeyakumar, K & Antonchick, A. P. Metal-free C–O bond functionalization: Catalytic intramolecular and intermolecular benzylation of arenes. *Org. Lett.* **20**, 3911–3914 (2008).

[19] Kumar, A. S. et al. Design and synthesis of biaryl aryl stilbenes/ethylenes as antimicrotubule agents. *Eur. J. Med. Chem.* **60**, 305–324 (2013).

[20] Antilla, J. C., Klapars, A. & Buchwald, S. L. The Copper-Catalyzed N-Arylation of Indoles. *J. Am. Chem. Soc.* **124**, 11684–11688 (2002).

[21] Bruker AXS (**2021**) APEX4 Version 2021.4-0, SAINT Version 8.40B and SADABS Bruker AXS area detector scaling and absorption correction Version 2016/2, Bruker AXS Inc., Madison, Wisconsin, USA.

[22] Sheldrick, G. M. SHELXT – Integrated space-group and crystal-structure determination. Acta Cryst. **A71**, 3-8 (2015).

[23] Sheldrick, G. M. Crystal structure refinement with SHELXL. Acta Cryst. **C71**, 3-8 (2015).

[24] Bruker AXS (**1998**) XP – Interactive molecular graphics, Version 5.1, Bruker AXS Inc., Madison, Wisconsin, USA.
